# Cytological observation and RNA-seq analysis reveal novel miRNAs high expression associated with the pollen fertility of neo-tetraploid rice

**DOI:** 10.1186/s12870-023-04453-y

**Published:** 2023-09-18

**Authors:** Xiang Li, Xu Huang, Minsi Wen, Wei Yin, Yuanmou Chen, Yuanlong Liu, Xiangdong Liu

**Affiliations:** 1 Guangdong Provincial Key Laboratory of Utilization and Conservation of Food and Medicinal Resources in Northern Region, Shaoguan University, Shaoguan, China; 2 Guangdong Laboratory for Lingnan Modern Agriculture, South China Agricultural University, Guangzhou, China; 3 State Key Laboratory for Conservation and Utilization of Subtropical Agro-Bioresources, South China Agricultural University, Guangzhou, China; 4 Guangdong Provincial Key Laboratory of Plant Molecular Breeding, South China Agricultural University, Guangzhou, China; 5 College of Biology and Agriculture, Shaoguan University, Shaoguan, China

**Keywords:** Neo-tetraploid rice, fertility, microRNAs, Transcriptome, Degradome

## Abstract

**Background:**

Neo-tetraploid rice lines exhibit high fertility and strong heterosis and harbor novel specific alleles, which are useful germplasm for polyploid rice breeding. However, the mechanism of the fertility associated with miRNAs remains unknown. In this study, a neo-tetraploid rice line, termed Huaduo21 (H21), was used. Cytological observation and RNA-sequencing were employed to identify the fertility-related miRNAs in neo-tetraploid rice.

**Results:**

H21 showed high pollen fertility (88.08%), a lower percentage of the pollen mother cell (PMC) abnormalities, and lower abnormalities during double fertilization and embryogenesis compared with autotetraploid rice. A total of 166 non-additive miRNAs and 3108 non-additive genes were detected between H21 and its parents. GO and KEGG analysis of non-additive genes revealed significant enrichments in the DNA replication, Chromosome and associated proteins, and Replication and repair pathways. Comprehensive multi-omics analysis identified 32 pairs of miRNA/target that were associated with the fertility in H21. Of these, *osa-miR408-3p* and *osa-miR528-5p* displayed high expression patterns, targeted the phytocyanin genes, and were associated with high pollen fertility. Suppression of *osa-miR528-5p* in Huaduo1 resulted in a low seed set and a decrease in the number of grains. Moreover, transgenic analysis implied that *osa-MIR397b-p3*, *osa-miR5492*, and *osa-MIR5495-p5* might participate in the fertility of H21.

**Conclusion:**

Taken together, the regulation network of fertility-related miRNAs-targets pairs might contribute to the high seed setting in neo-tetraploid rice. These findings enhance our understanding of the regulatory mechanisms of pollen fertility associated with miRNAs in neo-tetraploid rice.

**Supplementary Information:**

The online version contains supplementary material available at 10.1186/s12870-023-04453-y.

## Introduction

Rice (*Oryza sativa* L.) is one of the main food crops in the world. Because of the world’s continuing population growth, rice breeders have focused on yield and quality for decades. However, increasing the continuous yield of rice is a great challenge. A polyploid plant with superior biotic/abiotic resistance and yield potential is a candidate for future breeding [1–5]. Autotetraploid rice, derived from diploid rice by doubling its chromosomes, has great biological advantages, such as larger vegetative organs and higher stress resistance than diploid progenitors [6–8]. Even so, the low fertility of autotetraploid rice is still the major commercial production barrier.

Previous studies have shown that abnormal pollen, embryo sac, embryogenesis, and endosperm development were involved in the low fertility of autotetraploid rice [9–15]. Abnormal chromosome behavior and pairing were among the main reasons for pollen sterility in autotetraploid rice [12, 16]. The meiosis-related genes and small RNAs may be associated with the pollen sterility in autotetraploid rice, including *PAIR2*, *OsDMC1B*, *OsMTOPVI*, *osa-miR167h-3p*, *osa-miR159a.1*, and et al. [8, 12–14, 16]. Moreover, 24nt-phasiRNAs triggered by *osa-miR2275d*, and lncRNA57811 were found to be associated with the low pollen fertility of autotetraploid rice [13, 17]. With many years of effort, Chinese scientists bred tetraploid rice with high fertility, including polyploid meiosis stability rice (PMeS) and neo-tetraploid rice (NTR), which could overcome the low fertility of autotetraploid rice [18–20].

More than 20 lines of tetraploid rice were developed in 2009 by our research group [4, 19, 21–24]. Neo-tetraploid lines display over 80% seed set and high heterosis, harbor wide-compatibility and neutral pollen fertility genes, and novel specific alleles [19, 21, 24–27]. So far, more than 10 genes have been found to participate in the fertility of neo-tetraploid rice, including *kin7l*, *bzr3*, *nrfg4*, *MOF1a*, *HSP101–1*, *NY1*, and *NY2* [23, 25, 26, 28, 29]. However, little information about fertility regulation by microRNAs (miRNAs) is available in neo-tetraploid rice.

MicroRNAs, typically with 21–24 nucleotides, were involved in various biological processes of plants, including development, meiosis, and stress response [30–32]. Plant miRNAs typically functioned by base-pairing with target mRNAs, resulting in either translational repression or mRNA degradation [33]. miRNAs have emerged as important fertility regulators in rice and played critical roles during rice pollen development [34–36]. Previous studies proved that *OsmiR408* and *OsmiR528* could positively regulate rice pollen fertility by cutting the phytocyanin-related target gene [37]. In addition, reproductive phasiRNAs triggered by *OsmiR2118*, targeting the downstream gene (*MEL1*), were required for meiotic progression and pollen fertility in rice [38]. Altered expression of *OsmiR159a* affected rice yield traits and caused pollen defects [39]. Recently, *OsmiR156*, *OsmiR5488*, and *OsmiR399* were reported to play important roles in the regulation of male sterility in photoperiod- and thermo-sensitive genic male sterile rice [40].

In this study, a neo-tetraploid rice, Huaduo21 (H21), was used to evaluate the ability to overcome autotetraploid rice’s low fertility. Moreover, WE-CLSM (whole mount eosin B confocal laser scanning microscopy) was employed to examine the fertility of H21, and microRNAome, transcriptome, and degradome were used to identify the fertility-related miRNAs in the neo-tetraploid rice. The results of this study may provide insights into the underlying molecular mechanism of miRNAs-targets in the regulation of fertility in neo-tetraploid rice.

## Result

### Huaduo21 shows high fertility and yield potential compared to autotetraploid rice

Huaduo21 (H21) was developed by the self-crossing of autotetraploid rice lines, Jackson-4x (T45), crossed with 96025-4x (T44) in our lab. H21 displayed excellent agronomic traits compared with its autotetraploid rice parents, especially in yield-related traits, including seed setting (76.85%), the number of filled grains per plant, and 1000-seed weight (Supplementary Information Fig. S1 and Table S1). To evaluate the ability of H21 hybrids in overcoming the sterility, we developed hybrids by crossing H21 with the low fertility autotetraploid rice, T410, T431, and T45. The F_1_ hybrids not only had normal fertility with the seed set of 80.55% (T410×H21), 76.60% (T431×H21), and 75.97% (T45×H21), but also showed obvious heterosis, especially in the traits of effective panicle number, the number of filled grains per plant, the number of grains per plant and grain weight per plant (Supplementary Information Table S2). These results indicated that H21 had the potential to overcome the sterility of autotetraploid rice hybrids and be applied for polyploid rice breeding.

### Huaduo21 shows lower abnormal chromosome behavior and embryo sac fertility compared to autotetraploid rice

**Fig. 1:**
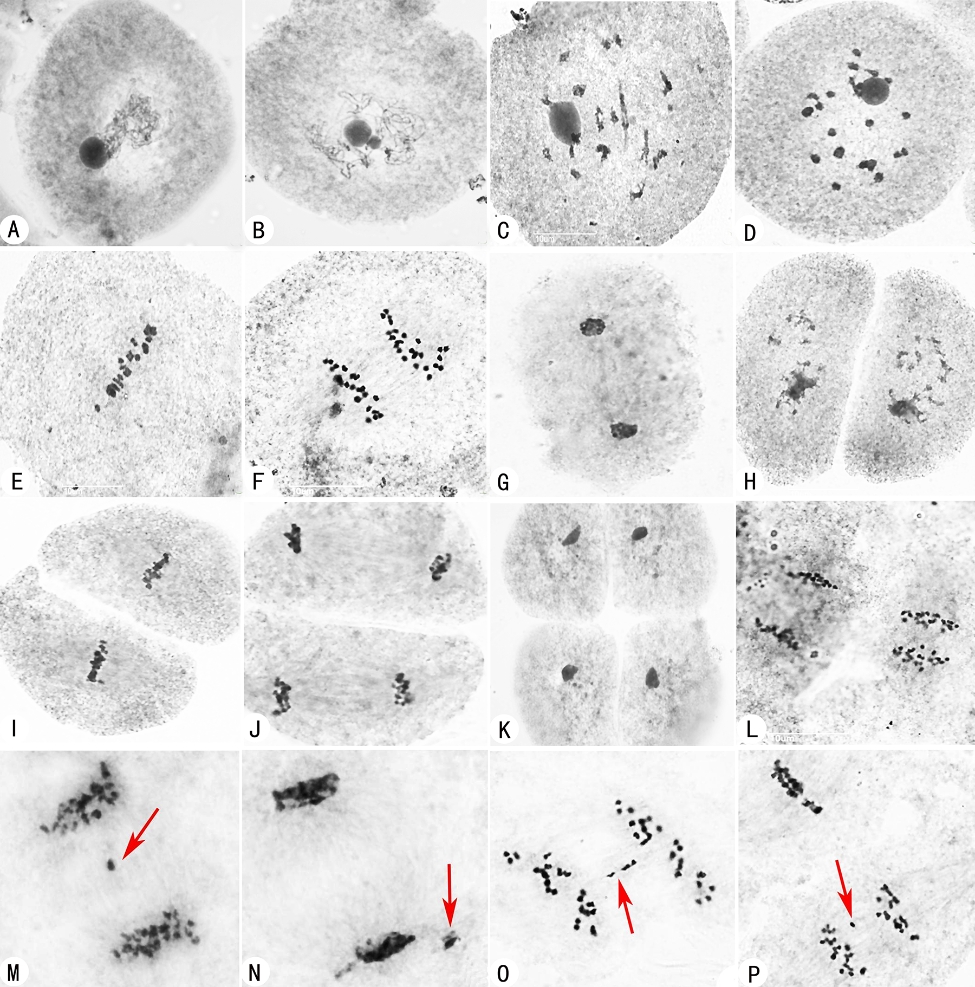
Observation of chromosome behaviors in Huaduo21 (H21) and its parents during meiosis (×3000). Similar and stage divisions were classified among H21, T44, and T45, including (**A**) zygotene, (**B**) pachytene, (**C**) diplotene, (**D**) diakinesis, (**E**) metaphase I, (**F**) anaphase I, (**G**) telophase I, (**H**) prophase II, (**I**) metaphase II, anaphase II, (**J**) telophase II and (**K**) tetrad. (**L**) asynchrony of the dyad at anaphase II in T44. (**M**, **N**) lagged chromosome (red arrow) and micronucleus (red arrow) at telophase I in T44. (**O**) chromosome lagging (red arrow) at anaphase I in T45. (**P**) asynchrony of the dyad with one at metaphase II and another at anaphase II with chromosome lagging in T45. ‘T44’ and ‘T45’ indicated the autotetraploid rice 96025-4x and Jackson-4x, respectively. ‘H21’ indicated the neo-tetraploid rice Huaduo21

The pollen fertility of H21 was 88.08%, which was significantly higher than T44 and T45 (Table 1). Furthermore, chromosomal behavior observation of pollen mother cells (PMCs) was performed in H21 and its parents during pollen development. Similar processes and stage divisions during meiosis were detected in H21 and its parents (Fig. 1). Many abnormalities of PMCs were found in the autotetraploid rice parents of T44 and T45, such as asynchrony of the dyad, lagged chromosome and micronucleus at telophase I in T44 (Fig. 1L-N), chromosomes lagging at anaphase I (Fig. 1O), and asynchrony of the dyad (one at metaphase II, while another at anaphase II) (Fig. 1P) in T45. In contrast, H21 exhibited fewer abnormal PMCs than T44 and T45. Only 4.16% and 7.87% abnormal PMC were identified at metaphase I and anaphase I in H21 (Table 1). The abnormalities of PMCs at metaphase I decreased by 72.77% and 69.34% in H21 compared to T44 and T45 (72.45% and 68.03% at anaphase I), respectively.

**Table 1:** Pollen fertility and abnormalities chromosome behavior in Huaduo21 (H21)

Materials	Metaphase I	Anaphase I	Metaphase II	Anaphase II	Tetrad	Pollen fertility/%	Seed setting/%
H21	4.16%	7.87%	7.87%	46.63%	1.68%	88.08 ± 1.31	76.85 ± 9.17
T45	13.57%	24.62%	5.83%	48.68%	2.23%	59.96 ± 3.82**	39.95 ± 13.93**
T44	15.28%	28.57%	6.67%	53.33%	1.78%	54.31 ± 1.81**	29.83 ± 9.26**

Note: ** indicates a significant difference in pollen fertility between neo-tetraploid rice and its parents at *p* < 0.01

**Fig. 2:**
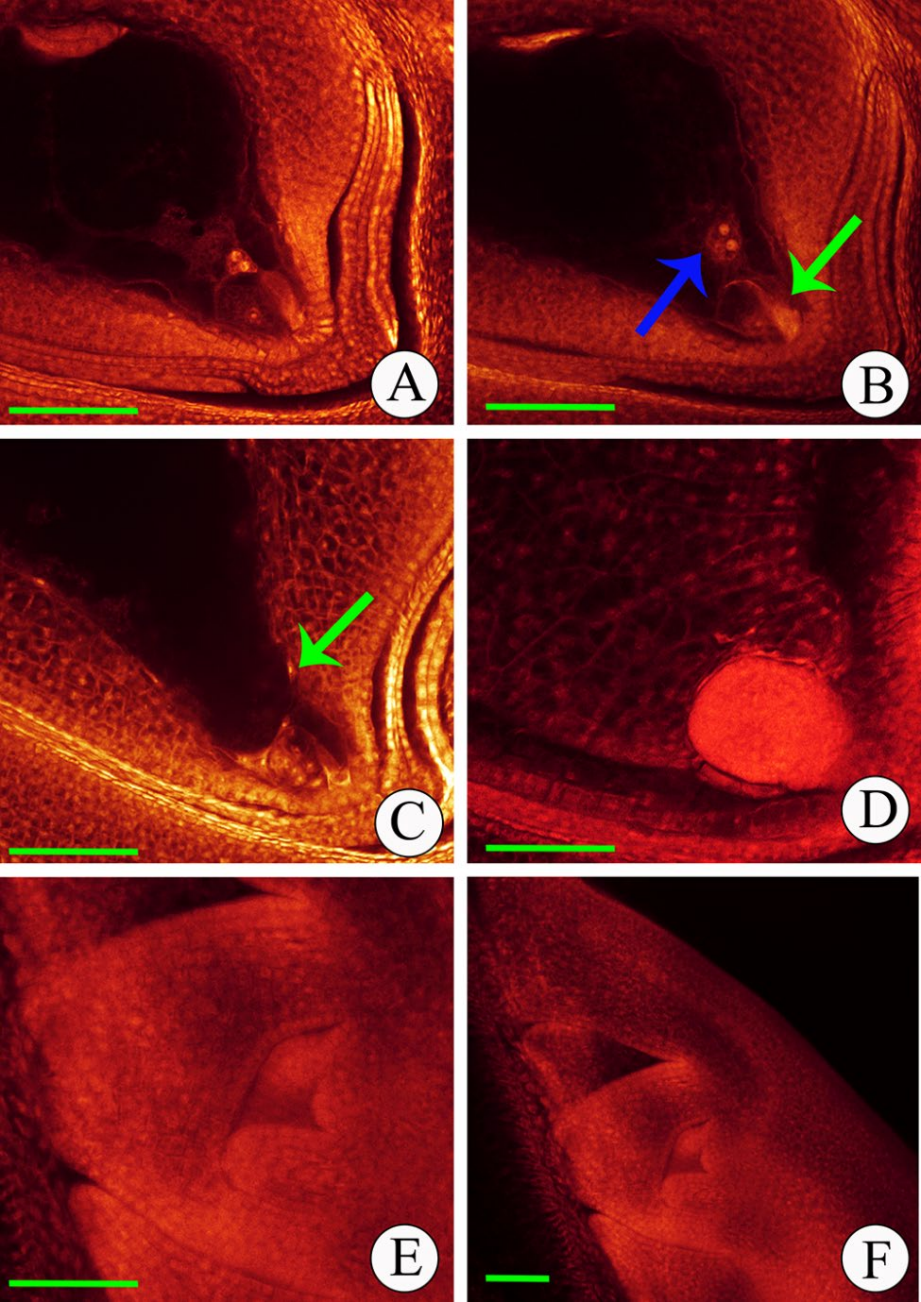
Fertilization and embryo development of Huaduo21 (H21). (**A**) At 5 min after flowering. Pollens were just landed on the stigma; the pollen tube had not been reaching the micropyle pole for double-fertilization. (**B**) At 1 h after flowering. The pollen tube with a bright horn-like structure (green arrow) passed through the micropylar pole. One of the released sperm cells moved toward the egg cell, and the other toward polar nuclei (blue arrow). (**C**) At 1d after flowering. The primary endosperm nucleus was formed from fertilizing the sperm nucleus and polar nuclei. Then, the primary endosperm nucleus started its free nuclei division, and the free nuclei (green arrow) were distributed around the embryo sac wall. The zygote was formed by the fertilization of the sperm nucleus and egg nucleus and underwent cell division at this time. (**D**) At 3d after flowering. The endosperm cell wall was formatted, and a globular embryo was identified in the micropyle pole. (**E-F**) At 5d after flowering. Scutellum, coleoptile, and plumule were formed in the embryo. The picture of E was caught in the x20 objective, while F was in x10. Bar = 100 μm

Moreover, the fertilization and embryo development (5 min, 1 h, 1 day, 3 days, and 5 days) of the neo-tetraploid rice H21 were observed by WE-CLSM. The development of fertilization and embryo in H21 and autotetraploid rice (Fig. 2 and Supplementary Information Fig. S2) were consistent with the previous studies [41, 42]. However, abnormalities were observed in autotetraploid rice, including abnormal polar nuclei in number and position, single-fertilization, unfertilization, and embryo degeneration (Supplementary Information Fig. S2). In contrast, the endosperm and embryo development after double-fertilization in the neo-tetraploid rice showed fertility similar to the diploid rice compared to autotetraploid rice. The abnormalities rate of 1, 3, and 5 days after flowering decreased by 43.52%, 51.74%, and 44.88% in H21 compared to the autotetraploid rice (Supplementary Information Fig. S3). Taken together, the high seed setting rate of the neo-tetraploid rice showed a strong relationship to the normal double fertilization of embryo sac, pollen, and embryo sac development.

### Identification of differentially expressed miRNAs in Huaduo21 during pollen development

**Fig. 3:**
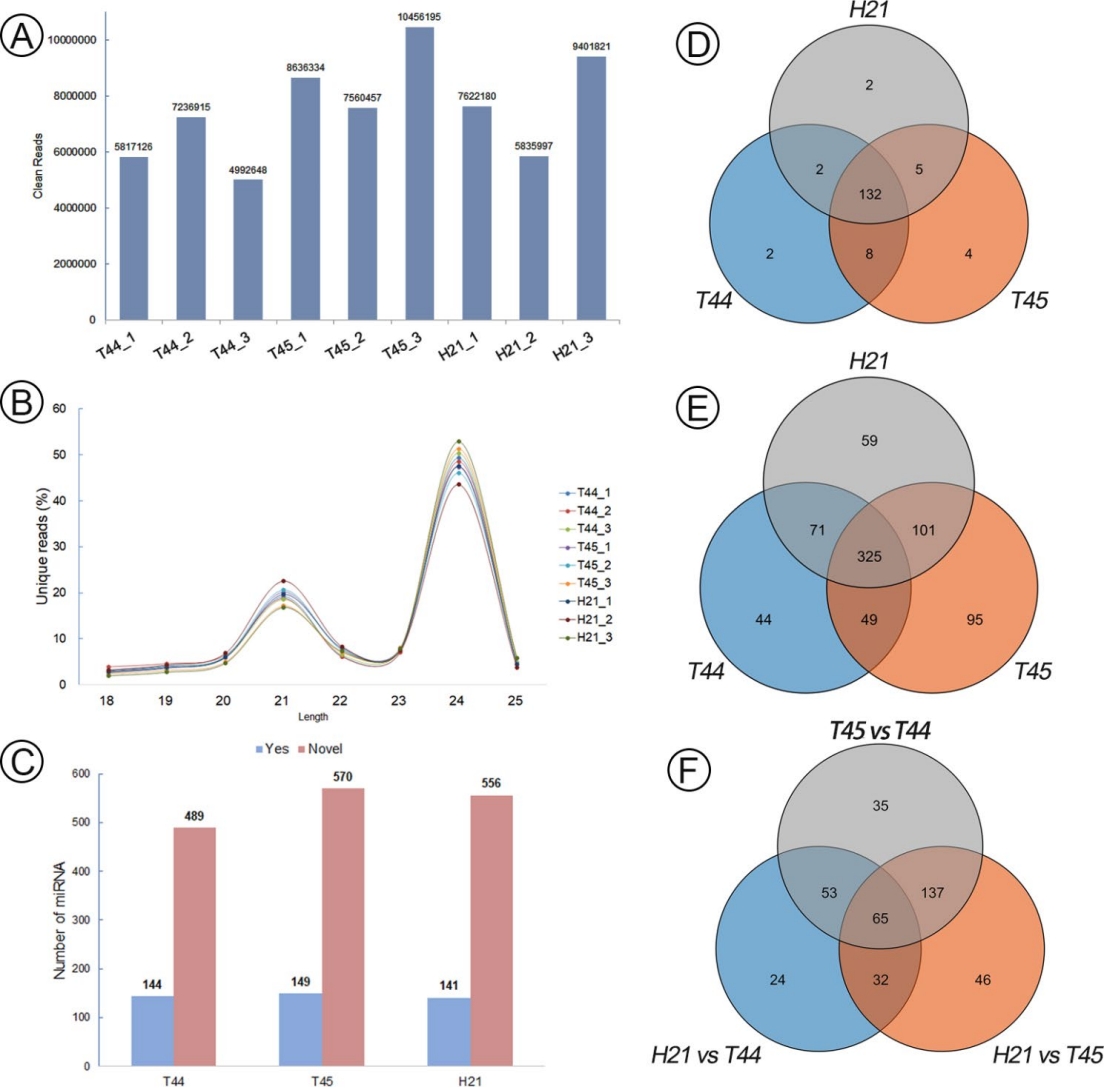
Identification and characterization of miRNAs in neo-tetraploid rice and autotetraploid rice. (**A**) Unique clean reads in T44, T45, and Huaduo21 (H21). (**B**) Length distribution of unique sRNAs in this study. (**C**) Numbers of known and novel miRNAs in T44, T45, and H21. (**D**, **E**) The specific known (**D**) and novel (**E**) miRNAs in T44, T45, and H21. (**F**) Venny analysis of differentially expressed miRNAs among the H21, T45, and T44

To identify fertility associated miRNAs in H21, nine sRNA libraries (three replicated experiments) were constructed from anthers at the meiosis stage of the three lines, including T44, T45 and H21, and sequenced using Illumina sequencing technology. After removing adaptors and low-quality reads, deep sequencing yielded 4.9 to 10.4 million clean reads in the nine sRNA libraries (Fig. 3A). The sRNAs were dominated in 21–24nt sequences, where 24nt unique sequences represent the most prevalent size class (more than 40%) (Fig. 3B). A total of 899 unique miRNAs were identified in T44, T45, and H21 at the meiosis stage of the anther. Among these miRNAs, 155 miRNAs were known, while 744 were novel (Fig. 3C). Venn analysis detected two, four, and two known miRNAs explicitly to T44, T45, and H21, respectively (Fig. 3D). In addition, numbers 44, 95, and 59 novel miRNAs were explicitly found in T44, T45, and H21, respectively (Fig. 3E). Compared to the T44 and T45, 174 and 280 differentially expressed miRNAs (DEM) were detected in H21, respectively. Additionally, a total of 290 miRNAs displayed differential expression between T44 and T45. We further defined DEM between the hybrid (H21) and its parents (T44 and T45) as DEM_FP_ and those between the parental lines as DEM_PP_. Venny’s result revealed that 102 (24 + 32 + 46) DEM were categorized as DEM_FPU_ because they were unique to the groups of H21 against T44 and H21 vs. T45 (Supplementary Information Table S3). These DEM might associate with phenotypic changes in H21 (Fig. 3F).

Additive and non-additive expressions were classified based on the expression level of miRNAs in the H21 relative to the mid-parent value (MPV = 1/2(T44 + T45)) [22]. Based on the expression level of miRNAs in H21 and the MPV (|log2 (Fold Change ratio)| > 1 and *q-value* < 0.05), 166 miRNAs were obtained for further non-additive expression pattern analysis, and they were designated as non-additive miRNAs (NAM) (Supplementary Information Table S4). The NAM was classified into five groups, including (1) significantly higher than both parents (NAM_HP_), (2) significantly lower than both parents (NAM_LP_), (3) statistically close to T44 (defined as NAM_CT44_), or (4) close to T45 (defined as NAM_CT45_), and (5) the expression level of H21 between T44 and T45 (NAM_BP_). According to these classifications, 34 NAM_HP_, 18 NAM_LP_, 39 NAM_CT45_, 72 NAM_CT44_, and 3 NAM_BP_ were identified from the NAM during meiosis in H21.

**Fig. 4:**
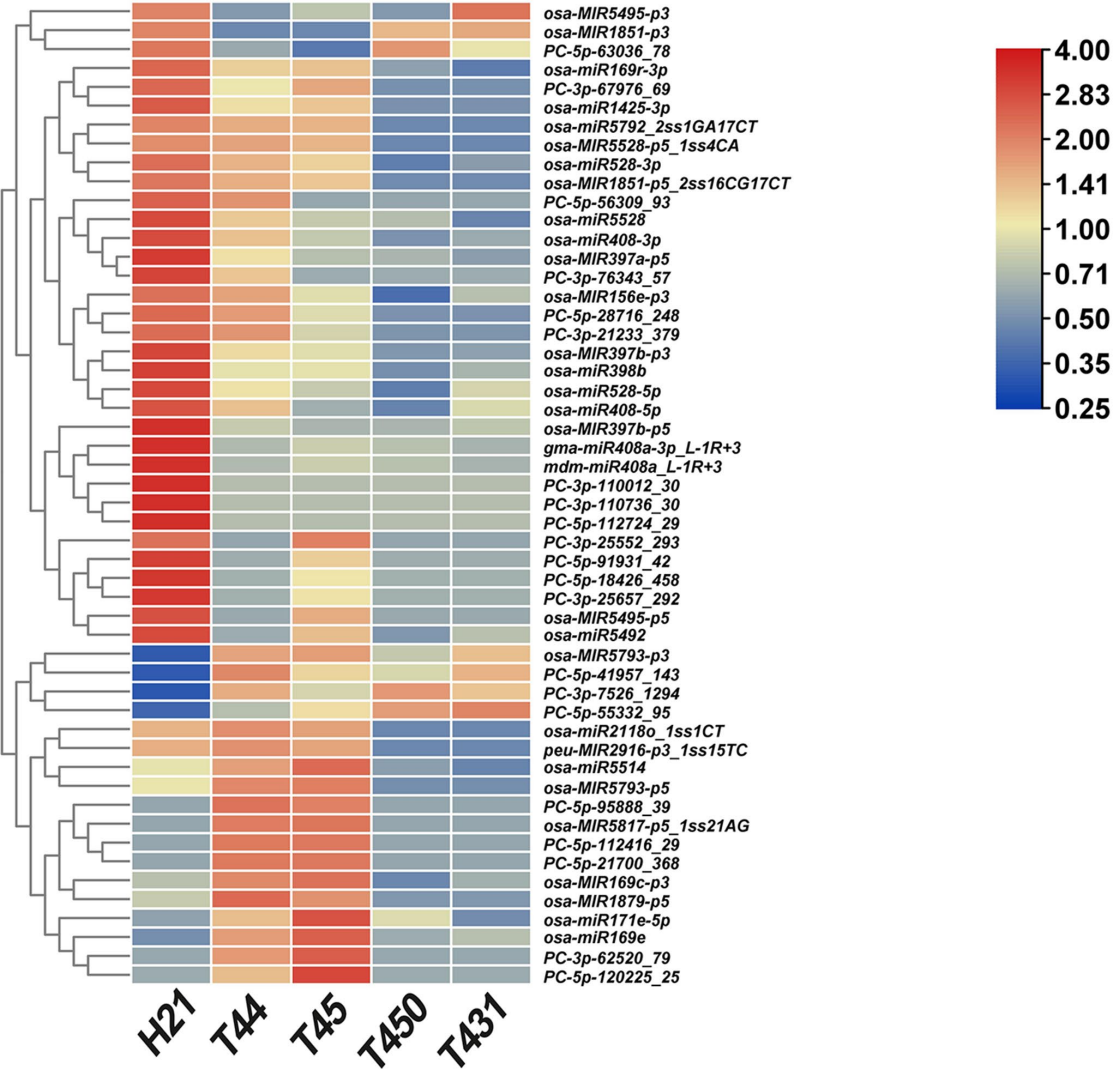
Heatmap of the part of the non-additive miRNAs between Huaduo21 (H21) and four autotetraploid rice (T44, T45, T450, and T431). The scale bar indicates the relative expression levels of miRNAs (log2)

**Fig. 5:**
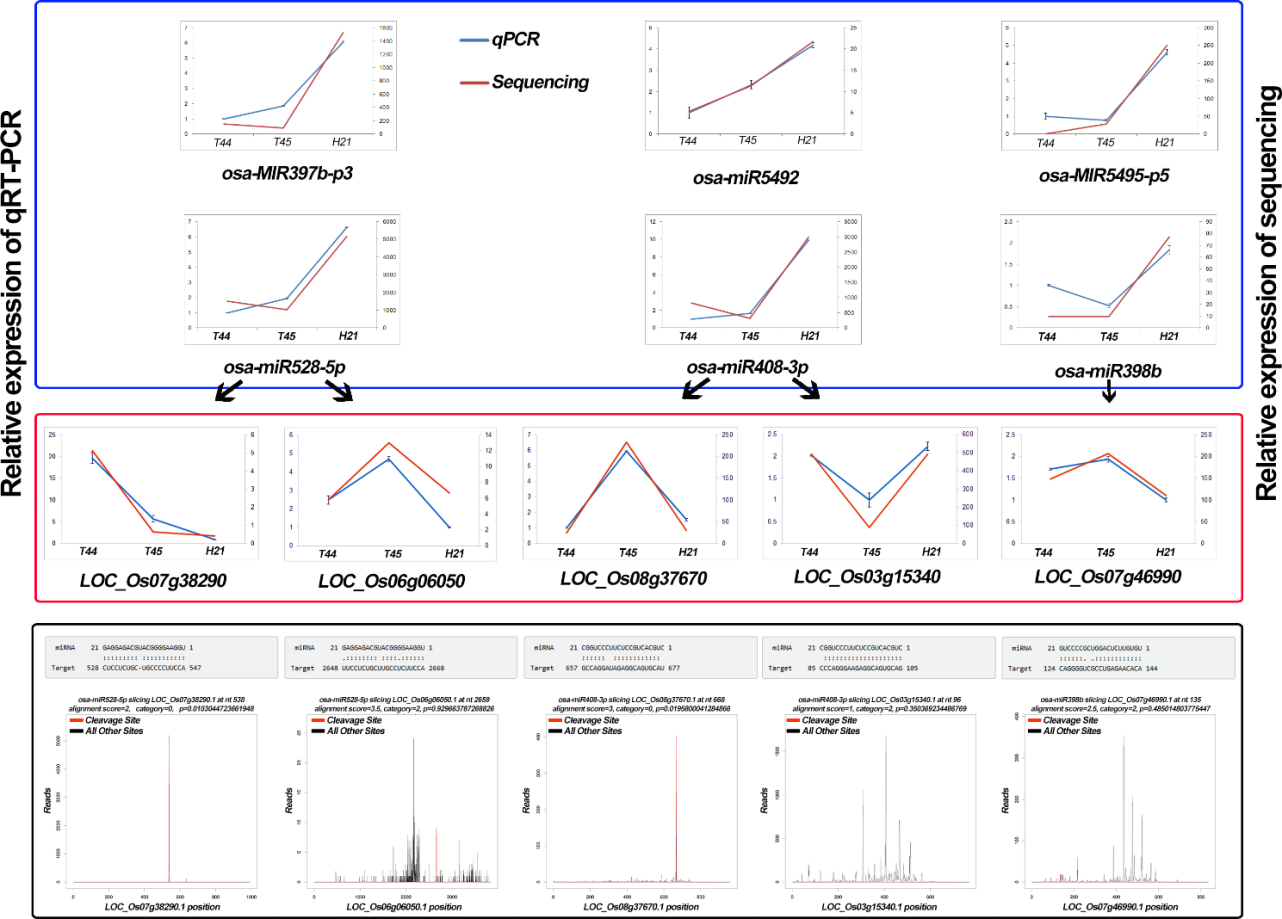
Validation of differentially expressed miRNAs and their corresponding target genes. The blue and red box content represents the qRT-PCR results of miRNAs and target genes, respectively. The blue and red lines represent the relative expression levels of qRT-PCR and Sequencing date (miRNA/RNA), respectively. qRT-PCR was performed in three biological replicates and three technical replicates. Error bars represent the standard deviation (SD). The black arrow indicates the miRNA-target pairs. The content within the black box represents the *t*-plot and cleavage site between miRNAs and their targets. The alignment between representative miRNAs and their targets is shown below the corresponding *t*-plot. Red lines indicate the signature produced by miRNA-directed cleavage in the *t*-plot

Furthermore, cluster analysis of the 34 NAM_HP_ and 18 NAM_LP_ expression patterns was performed among H21, T44, and T45, which integrated with the miRNAome data of 02428-4x (T450) and Taichung65-4x (T431) at the meiosis stage during pollen development [8, 13]. Most NAM_HP_ showed the highest expression levels in neo-tetraploid rice H21, while lower expression patterns in the low fertility autotetraploid rice (T44, T45, T450, and T431), such as *OSA-miR5792_2ss1GA17CT*, *osa-MIR397b-p3*, and *osa-miR528-3p.* Similar expression patterns were found among the NAM_LP_, including *osa-MIR5793-p3, PC-3p-7526_1294*, and *PC-5p-55332_95* (Fig. 4). These miRNAs could associate with the high fertility of neo-tetraploid rice. In addition, 25 DEM were selected for qRT-PCR validation, of which 22 were NAM. The expression patterns of the miRNAs obtained through qRT-PCR were consistent with the microRNAomes (Fig. 5, Supplementary Information Fig. S4 and Fig. S5).

### Identification of differentially expressed genes in Huaduo21 during pollen development

To investigate the target gene expression changes associated with fertility in H21, transcriptome libraries were constructed using the same samples as for miRNA sequencing. After the removal of low-quality reads, an average of 47 million high-quality clean reads were obtained from each sample. The Q30 base percentage in all samples ranged from 96.29 to 97.71%. Clean reads of each sample were aligned with the reference genome, and the alignment efficiency ranged from 93.17 to 94.97% (Supplementary Information Table S5).

**Fig. 6:**
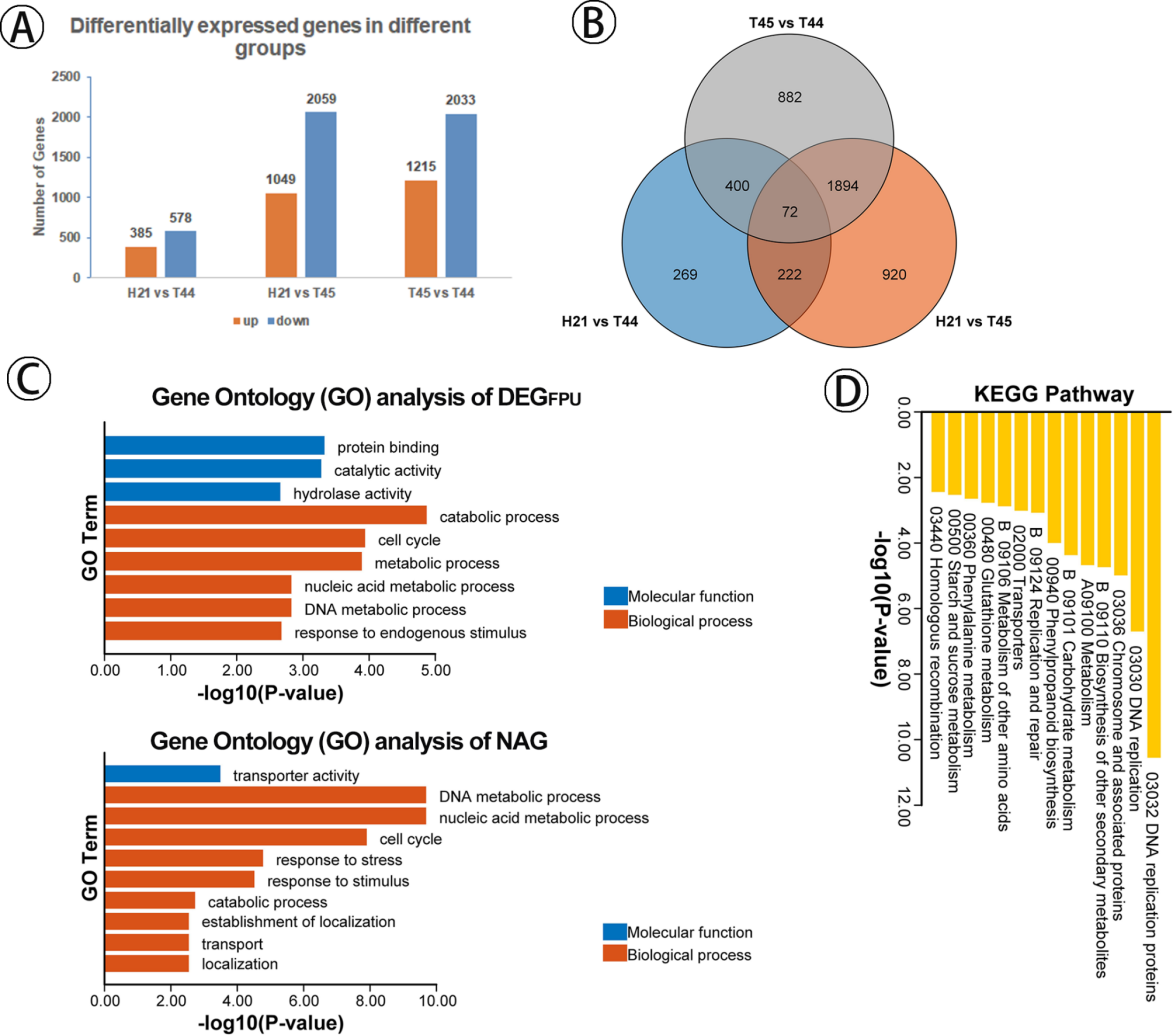
Identification and characterization of differentially expressed genes (DEG) in Huaduo21 (H21). (**A**) The DEG in H21 compared to T44, H21 compared to T45, and T44 compared to T45. (**B**) Venny analysis of differentially expressed miRNAs among the H21, T45, and T44. (**C**) Gene Ontology (GO) analysis of DEG_FPU_ (DEG were uniquely belonged to H21 compared to T44 and T45) and NAG (non-additive genes) in H21. Genes are classified into three major categories: biological process and molecular function. The X-axis indicates the -log10 (P-value) of GO terms and the Y-axis indicates the significance of GO terms (*p*-value < 0.05 and *q-value* < 0.05). (**D**) Kyoto Encyclopedia of Genes and Genomes (KEGG) enrichment analysis of NAG. The X-axis indicates the -log10 (P-value) of KEGG pathways, and the Y-axis represents the pathway name (*p*-value < 0.05 and *q-value* < 0.05)

A total of 963 differentially expressed genes (DEG) were identified in H21 compared to T44, of which 385 and 578 were up- and down-regulated in H21, respectively. A total of 3108 DEG were found in H21 compared with T45, of which 1049 and 2059 showed up- and down-regulation in H21. We also found 3248 DEG between T44 and T45 (Fig. 6A). We defined DEG between the hybrid (H21) and its parents (T44 and T45) as DEG_FP_ and those between the parental lines as DEG_PP_. Venny’s result showed that a total of 1411 (269 + 222 + 920) DEG uniquely belonged to H21 compared to T44 and T45, defined as DEG_FPU_ (Fig. 6B and Supplementary Information Table S6). The DEG_FPU_ may be relevant to phenotypic differences between H21 and its parents (T44 and T45); therefore, we specifically focused on the DEG_FPU_ to explore genes related to the fertility/heterosis in H21. The Gene Ontology (GO) analysis of DEG_FPU_ was performed, and 6 and 3 GO terms were significantly enriched in biological processes and molecular function, respectively (Fig. 6C). Biological processes associated with “catabolic process”, “cell cycle”, “metabolic process”, “nucleic acid metabolic process”, “DNA metabolic process”, and “response to endogenous stimulus” were significantly enriched in H21. Among the molecular function, “protein binding”, “catalytic activity”, and “hydrolase activity” were particularly prominent in H21. Kyoto Encyclopedia of Genes and Genomes (KEGG) analysis of DEG_FPU_ showed no significant pathways in H21 with *p*-value < 0.05 and *q*-value < 0.05. The pathway of “DNA replication proteins” with *p*-value < 0.05 and *q*-value = 0.098724565 was identified in H21.

Similar to the analysis of additive and non-additive expression of miRNAs, a total of 3448 non-additive genes (NAG) were obtained between H21 and its parents (T44 and T45), including 163 NAG_HP_ (significantly higher than both parents), 98 NAG_LP_ (significantly lower than both parents), 1432 NAG_CT44_ (statistically close to T44), 439 NAG_CT45_ (statistically close to T45), and 1316 NAG_BP_ (the expression level of H21 between T44 and T45) (Supplementary Information Fig. S6 and Table S7). GO analysis for NAG revealed that 10 GO terms were significantly enriched, including “transporter activity”, “DNA metabolic process”, “nucleic acid metabolic process” and “cell cycle” related to biological process, respectively (Fig. 6C). In addition, the NAG were significantly enriched in the pathway of “DNA replication proteins”, “DNA replication”, ‘Chromosome and associated proteins”, “Carbohydrate metabolism”, “Replication and repair” and “Homologous recombination” by KEGG analysis (Fig. 6D). Together, GO and KEGG analysis showed that DEG was associated with fertility in H21.

### Degradome sequencing and targets prediction of miRNAs in tetraploid rice

To determine the regulatory relationship between miRNAs and their target genes, the mixed anther samples of H21, T44, and T45 were collected for degradome sequencing. In total, 29,137,433 raw reads and 6,768,733 unique raw reads were obtained, of which 6,729,491 reads were mapped to the rice genome, and the unique mappable reads were greater than 99%. The total number of input transcripts was 103,427, and the covered transcripts were 81,229, accounting for 78.54%. The results indicated that degradation sequencing yielded a high coverage of degradation fragments (Supplementary Information Table S8).

In total, 2623 miRNA-target pairs (including 265 unique miRNAs and 1344 unique targets) were detected by degradome sequencing (Supplementary Information Table S9). Based on the CleaveLand pipeline, the target transcripts were classified as 0, 1, 2, 3, and 4, according to the relative abundance of tags at the target sites [43]. Types 0, 1, 2, 3, and 4 had 556, 57, 1168, 72, and 770 cleavage events, respectively. The alignment between representative miRNAs and their target transcripts is shown below the corresponding *t*-plots (Fig. 5). The *t*-plots of miRNA-target pairs confirmed that degradome sequencing occurred in tetraploid rice. In addition, GO enrichment analysis showed that the targets were significantly involved in “flower development”, “reproduction”, “nucleus” and “transcription factor activity” (Supplementary Information Fig. S7). Eight KEGG pathways were significantly detected among the targets, such as “Ribosome”, “Plant hormone signal transduction” and “RNA transport” (Supplementary Information Fig. S7). These results showed that the miRNAs might play a big part in how their targets control the network that controls fertility in H21.

To validate the degradome sequencing, ten miRNA-target pairs were validated by using qRT-PCR (Fig. 5 and Supplementary Information Fig. S5). The expression patterns of *LOC_Os01g59660* (MYB family transcription factor) and *LOC_Os01g47530* (CGMC_MAPKCMGC_2.6 - CGMC includes CDA, MAPK, GSK3, and CLKC ki-ses) obtained by qRT-PCR differed from those obtained by RNA-Seq. This could be because the expression levels of these two genes were not significantly different between H21 and its two parents in RNA-Seq. Although *LOC_Os03g15340* (plastocyanin-like domain containing protein) and *LOC_Os04g30610* (disease resistance protein RGA2) were not showed opposite expression patterns to its regulator miRNA, *osa-miR408-3p* and *osa-miR2118p*, the expression patterns were similar to RNA-sequencing data; implied that the expression patterns of these targets might not only be regulated by miRNA cleavage but also by other pathways. Meanwhile, the relative expression levels of the remaining targets displayed the opposite expression patterns to their miRNAs, including *LOC_Os07g38290* (plastocyanin-like domain containing protein) targeted by *osa-miR528-5p*, *LOC_Os08g37670* (plastocyanin-like domain containing protein) targeted by *osa-miR408-3p*, and *LOC_Os07g46990* (copper/zinc superoxide dismutase) targeted by *osa-miR398b*. Taken together, the results indicated that the relative expression patterns by the qRT-PCR were similar to those produced by high-throughput sequencing, confirming that the sequencing results were reliable in the present study.

### Comprehensive analysis of miRNA expression profiles and target genes related to fertility in Huaduo21

To further determine the miRNAs related to fertility in H21, we integrated miRNAome, degradome, and transcriptome and compared the target genes with the most important meiosis-related targets reported in the previous studies [26, 44–46]. In total, 11 and 32 candidate miRNA-target pairs were found in H21 compared to T44 and T45, respectively (Supplementary Information Table S10). Of this list, six targets were identified that are associated with pollen development, meiosis, fertility, and grain yield, including *LOC_Os03g50140* (*OsUCL8*) [47] targeted by *osa-miR408-3p*, *LOC_Os06g40550* (*OsABCG15*) [48] targeted by *osa-miR5504_R-3*, *LOC_Os10g35240* (*Rf4|PPR782a*) [49] and *LOC_Os10g35640* (*Rf1b*) [50] targeted by *osa-miR1425-5p*, *LOC_Os12g29980* (*OsGRF7*) [51] targeted by *osa-miR396d* and *LOC_Os10g35550* (*Du1*) [52] targeted by *osa-MIR5489-p3*. Furthermore, five pollen mother cells-specific genes and four meiosis stage-specific genes were detected [44–46], such as *LOC_Os03g50160* (plastocyanin-like domain containing protein) targeted by *osa-miR528-5p*, *LOC_Os11g38810* (mannose-6-phosphate isomerase) targeted by *osa-miR5487* and *LOC_Os01g11430* (expressed protein) targeted by *osa-miR159a.1* that showed pollen mother cells-specific patterns in rice, and *LOC_Os07g08530* (auxin response factor) targeted by *osa-miR3979-3p* that displayed the meiosis stage-specific pattern in rice. In addition, two genes, *LOC_Os11g35030* (growth regulating factor protein) targeted by *osa-miR396c-5p* and *osa-miR396e-5p* and *LOC_Os11g34910* (expressed protein) targeted by *osa-MIR812r-p3_1ss18CT*, were found to be the neo-tetraploid rice unique genes [26]. The findings revealed that the differentially expressed miRNAs regulated H21 fertility via targeting pollen and meiosis-related genes.

### Transgenic analysis of fertility-related miRNAs reduced seed set in rice

**Fig. 7:**
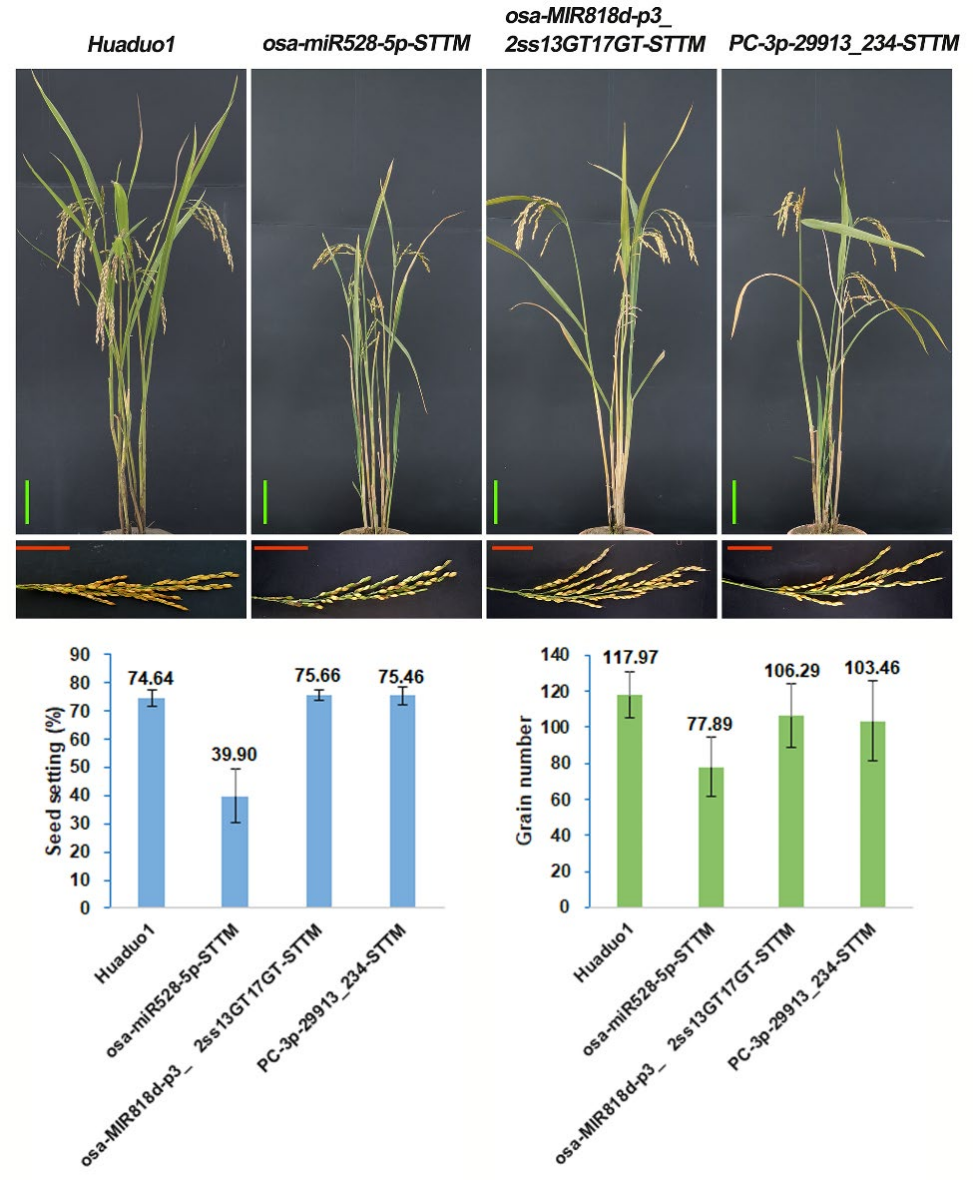
The phenotype of transgenic plants of fertility-related miRNAs in the T1 generation (Neo-tetraploid rice Huaduo1 background). *osa-miR528-5p*, non-additive miRNA, showed high expression in Huaduo21 (H21). *osa-MIR818d-p3_2ss13GT17GT* was the non-additive miRNA in H21. *PC-3p-29913_234* was not the differentially expressed miRNA in H21 compared to its parents. Green Bar: 10 cm. Red Bar: 5 cm

The functions of *osa-miR528* [37], *osa-miR408* [53], and *osa-miR398* [54] have already been reported in diploid rice, and they have indeed been shown to participate in the regulation of pollen fertility and rice yield-related traits. To test the potential functions of the fertility-related miRNAs, *osa-miR528-5p*, *osa-MIR397b-p3*, *osa-miR5492*, and *osa-MIR5495-p5* were selected for further analysis, which were the non-additive miRNAs and displayed high expression levels in H21 compared to the autotetraploid rice (Fig. 4). Short Tandem Target Mimic (STTM) has provided effective tools to block endogenous mature miRNA activity, making it technically possible to undertake large-scale genome-wide studies model and crop plants [55, 56]. The STTM vectors of *osa-miR528-5p*, *osa-MIR397b-p3*, *osa-miR5492*, and *osa-MIR5495-p5* were constructed and transformed *osa-miR528-5p-*STTM into neo-tetraploid rice Huaduo1 and *osa-MIR397b-p3-*STTM, *osa-miR5492-*STTM, and *osa-MIR5495-p5-*STTM into the *japonica* variety Nipponbare. The transgene mutants were planted in T1 (*osa-miR528-5p-*STTM)/T2-generation (*osa-MIR397b-p3-*STTM, *osa-miR5492-*STTM, and *osa-MIR5495-p5-*STTM). The seed set and grain number of the transgene plants *osa-miR528-5p-*STTM were deceased compared to Huaduo1 (Fig. 7). Moreover, the transgene plants of *osa-MIR397b-p3*-STTM, *osa-miR5492*-STTM, and *osa-MIR5495-p5*-STTM showed the distinctly low seed set with 32.40%, 42.85%, and 19.46% compared to wild type in T2 generation (Supplementary Information Fig. S8). These findings suggested that the miRNAs with high expression patterns in H21 played a key role in fertility.

## Discussion

### Huaduo21 shows fertility and exhibits the potential utilization in production compared to autotetraploid rice

Autotetraploid rice has considerable advantages compared to its diploid counterpart, except the fertility [12, 13, 57]. Poor fertility was the major hindrance in using autotetraploid rice at the commercial level. The abnormal pollen development, abnormal meiotic chromosome behavior, and abnormal embryo sac development could lead to pollen and embryo sac abortion in autotetraploid rice [13, 14]. So far, several neo-tetraploid rice lines with high fertility have been successfully bred [19, 21, 24–27]. High pollen fertility associated with lower abnormalities at PMC cells was found in neo-tetraploid rice when compared to autotetraploid rice [21, 24]. Here, the pollen fertility of H21 was 88.08%, which was higher than its two parents (T44 and T45). Moreover, only 4.16% and 7.87% abnormalities of chromosome behavior were detected at metaphase I and anaphase I of PMCs in Huaduo21 (H21), respectively, which is similar to Huaduo1, Huaduo3, 66, and 134 [21, 22, 24]. Furthermore, during meiosis, most bivalents and quadrivalents were found at the diakinesis and metaphase I stage related to high pollen fertility in Huaduo1 [22, 24]. These results suggest that higher fertility of neo-tetraploid lines was related to the high pollen fertility and the few abnormalities of chromosome behavior.

In addition, H21 showed a normal double fertilization process similar to diploid rice, while autotetraploid rice exhibited many abnormalities during the double fertilization development. Multi-types of abnormalities during the double fertilization and embryogenesis were identified in autotetraploid rice, resulting in a low seed set, including the embryo sac without endosperm/embryo, unfertilization, and embryo degeneration [10, 58]. These results also demonstrated that H21 with normal fertility might associate with lower abnormalities during double fertilization and embryogenesis. Interestingly, the giant and dull endosperm was only found in H21 among the neo-tetraploid rice (Supplementary Information Fig. S9). When the seeds matured, the glumes were dehiscence due to the puffed endosperm in H21.

Previous studies reported that neo-tetraploid rice produced high-yield heterosis when crossed with *indica* autotetraploid rice [25]. Similarly, heterosis analysis of F_1_ hybrids (Huaduo3 crossed with 26 *indica* and 14 *japonica* autotetraploid rice lines) demonstrated that the hybrids of Huaduo3 crossed with *indica* autotetraploid rice showed high yield heterosis than that crossed with *japonica* autotetraploid rice [19]. Moreover, Huaduo8, the wide compatibility germplasm of neo-tetraploid rice, had been reported could overcome the intersubspecific autotetraploid hybrid rice sterility caused by pollen sterility loci [27]. Chen et al. found that F_1_ hybrids generated by crossing the autotetraploid with neo-tetraploid rice exhibited high heterosis [22]. In the present study, we reported another neo-tetraploid rice, H21, which showed high fertility and yield potential, and produced high-yield heterosis. Like the other neo-tetraploid rice lines, H21 also displayed the potential ability to overcome the sterility of autotetraploid rice. The F_1_ hybrids developed by H21 (crossed with the low fertility autotetraploid rice) displayed high fertility (~ 77.70%) and positive heterosis associated with yield-related traits. These results indicated that H21 could improve fertility, produce high-yield vigor in tetraploid rice, and be the important germplasm for the breeding of polyploid rice. Taken together, all of these results demonstrate that H21 showed obvious heterosis and higher fertility than the autotetraploid rice.

### Fertility-related miRNAs might involve in the regulation of high fertility in neo-tetraploid rice Huaduo21

High-throughput sequencing technology has become an effective tool to identify the potential miRNAs related to rice fertility, such as the miRNAs participate in pollen development, embryo sac development, and grain filling [13, 40, 59]; however, there are still limitations to understand the fertility mechanism associated with miRNAs in neo-tetraploid rice. Here, we identified 166 non-additive miRNAs (NAM) in H21 during pollen development compared to its parents. Interestingly, of these NAM, the NAM_HP_ (significantly higher than both parents) and NAM_LP_ (significantly lower than both parents) showed similar expression patterns in H21 compared to autotetraploid rice (T44, T45, T450, and T431), indicating that the high fertility of neo-tetraploid rice may be related to these microRNAs.

Generally, miRNAs negatively regulate the target genes in plants by cutting or inhibiting the translation of target transcripts [60]. We identified 1411 differentially expressed genes (DEG) uniquely belonging to H21 compared to its two parents by transcriptomics. Gene Ontology results revealed these DEG were mainly involved in “DNA metabolic process”, “cell cycle”, “catalytic activity” and “hydrolase activity”. Meanwhile, a total of 3448 non-additive genes (NAG) were identified between H21 and its two parents. Gene Ontology showed that NAG was significantly enriched in “transporter activity”, “DNA metabolic process”, and “cell cycle”. Excitingly, the NAG was found to be related to the pathway of “DNA replication”, ‘Chromosome and associated proteins”, and “Replication and repair” by KEGG analysis. Taken together, function prediction indicated that the differentially expressed genes in H21 were related to the high pollen fertility and the few abnormalities of chromosome behavior.

In addition, we identified 2623 miRNA-target pairs by using degradome sequencing. Several fertility-related miRNAs-targets pairs related to high fertility in H21 were identified by comprehensive analysis of miRNAome, degradome, and transcriptome in the present study. MiR408 has been reported to target various blue copper protein members, including those in the phytocyanin family [53]. *OsmiR408* could positively regulate rice grain yield by increasing panicle branching and grain number by negatively regulating its target, a plantacyanin gene called *OsUCL8* [53]. Additionally, the overexpression of *OsUCL8* led to pollen tube abortions that resulted in low seed set in rice; however, knock-out of *OsUCL8* and overexpressing *miR408*, displayed vigorous pollens with a higher germination rate [47], which demonstrated that *OsmiR408*-*OsUCL8* regulatory network mainly affects pollen intine formation in rice. In the present study, we found *osa-miR408-3p* and *osa-miR408-5p*, the NAM_HP_, were highly expressed in H21 compared to autotetraploid rice. Three plastocyanin genes that target by *osa-miR408-3p* were identified by degradome sequencing, including *LOC_Os03g15340*, *LOC_Os08g37670*, and *LOC_Os03g50140* (*OsUCL8*). The negative expression patterns between *osa-miR408-3p* and *LOC_Os08g37670* were also confirmed in H21. These results illustrated that the dominant *osa-miR408-3p* negatively regulated the plastocyanin gene that might play a critical role in the high pollen fertility in H21. Recently, *OsmiR528* was found to be the key regulator for forming the pollen intine and contributes to male fertility in rice [37]. Knockout of *OsmiR528* causes aborted pollen development at the late binucleate pollen stage that results in low seed-setting in rice. Molecular function analysis showed that *OsmiR528* affected pollen development by directly targeting *OsUCL23*. *OsUCL23* was a uclacyanin protein that belonged to the phytocyanin family. In this study, *osa-miR528-5p* displayed high expression patterns in H21 compared to its parents. *LOC_Os07g38290*, annotated as a plastocyanin-like domain-containing protein that belonged to the phytocyanin family [61], targeted by *osa-miR528-5p* showed negative expression patterns in H21 by qPCR analysis. The suppression of *osa-miR528-5p* using the STTM approach in Huaduo1 resulted in a lower seed set and a decreased number of grains. Therefore, we hypothesized that the abundance of *osa-miR528-5p* was a key regulator for high pollen fertility in H21 and involved in its fertility regulation process. Taken together, we speculated that the accumulation of *osa-miR408-3p* and *osa-miR528-5p* targeted the phytocyanin genes affecting pollen development in neo-tetraploid rice, thus resulting in the high seed set.

Copper is an essential element for plants, especially in photosynthesis, as it is required for plastocyanin function in electron transfer reactions at thylakoid membranes [62]. Copper deficiency reduces lignin accumulation in anthers and reduces anther dehiscence in *Arabidopsis thaliana* [63]. *LOC_Os07g46990* (copper/zinc superoxide dismutase) was one potential target of *osa-miR398a* in the rice. The previous study showed that *osa-miR398a* could enhance rice’s panicle length, grain number, and grain size [54]. Smaller panicles and few grains number were found in transgenic STTM398 (suppression of *osa-miR398a*) and *mOs07g46990* (Modified *LOC_Os07g46990* contained five mismatches in the *miR398* target site and was expressed in transgenic STTM398 rice) lines than wild type. In addition, transgenic OX-miR398a (overexpression of *osa-miR398a*) lines showed bigger panicles and an increased number of grains per panicle compared with the wild type. In the present study, we identified a high expression level of *osa-miR398b* and a decreased expression level of *LOC_Os07g46990* in H21 compared to its parent. This finding suggests that these molecular changes may be attributed to the high pollen fertility and high yield heterosis observed in neo-tetraploid rice.

*OsABCG15* (*LOC_Os06g40550*) was required for rice anther development and pollen fertility [48]. Here, *OsABCG15* showed differential expression patterns in H21 compared to its two parents. *osa-miR5504_R-3* was targeted *OsABCG15* by degradome analysis. These results suggested that the *osa-miR5504_R-3-OsABCG15* regulation pathway might involve in the H21 pollen fertility development. The *osa-miR396d* displayed a significant down-regulation pattern in H21 compared to T44 in the present study. Chandran et al. reported that blocking the expression of *OsamiR396* leads to increasing rice grain yield [51]. In addition, *OsGRF7* (*LOC_Os12g29980*), targeted by *OsamiR396*, could modulate rice plant architecture. Recent studies have found that overexpression of *OsGRF7* caused a semidwarf and compact plant architecture with increased culm wall thickness and narrowed leaf angles in rice [64]. These results demonstrated that the high yield-related traits and the better plant architecture could be related to the role of the *miR396*-*OsGRF7* regulatory module in H21. Furthermore, *osa-MIR5489-p3* significantly down-regulated in H21 compared to T45 in this study. Its target, *Du1* (*LOC_Os10g35550*), has been reported to regulate the starch biosynthesis pathway in rice [52]. *Du1* was expressed mainly in rice panicles. The dull endosperm was found in *du1* grains with a significantly decreasing amylose content compared to the wild-type. This was an interesting result because H21 also had dull endosperm when it was fully; implying that *osa-MIR5489-p3* might target *Du1* to regulate the starch biosynthesis pathway in H21.

Moreover, we selected three non-additive miRNAs (NAM_HP_) of H21 for transgenic studies in this study, including *osa-MIR397b-p3*, *osa-miR5492*, and *osa-MIR5495-p5*. The transgenic analysis result of *osa-MIR397b-p3* was similar to the previous study that *OsmiR397* could play an essential function in rice yield [65]. Meanwhile, low seed set of the *osa-miR5492* and *osa-MIR5495-p5* supression transgene plants were identified in the present study, which implied that *osa-miR5492* and *osa-MIR5495-p5* might play a vital function during pollen development in rice. The function and the regulation network of *osa-miR5492* and *osa-MIR5495-p5* during pollen development in neo-tetraploid rice should be further analyzed.

## Conclusions

**Fig. 8:**
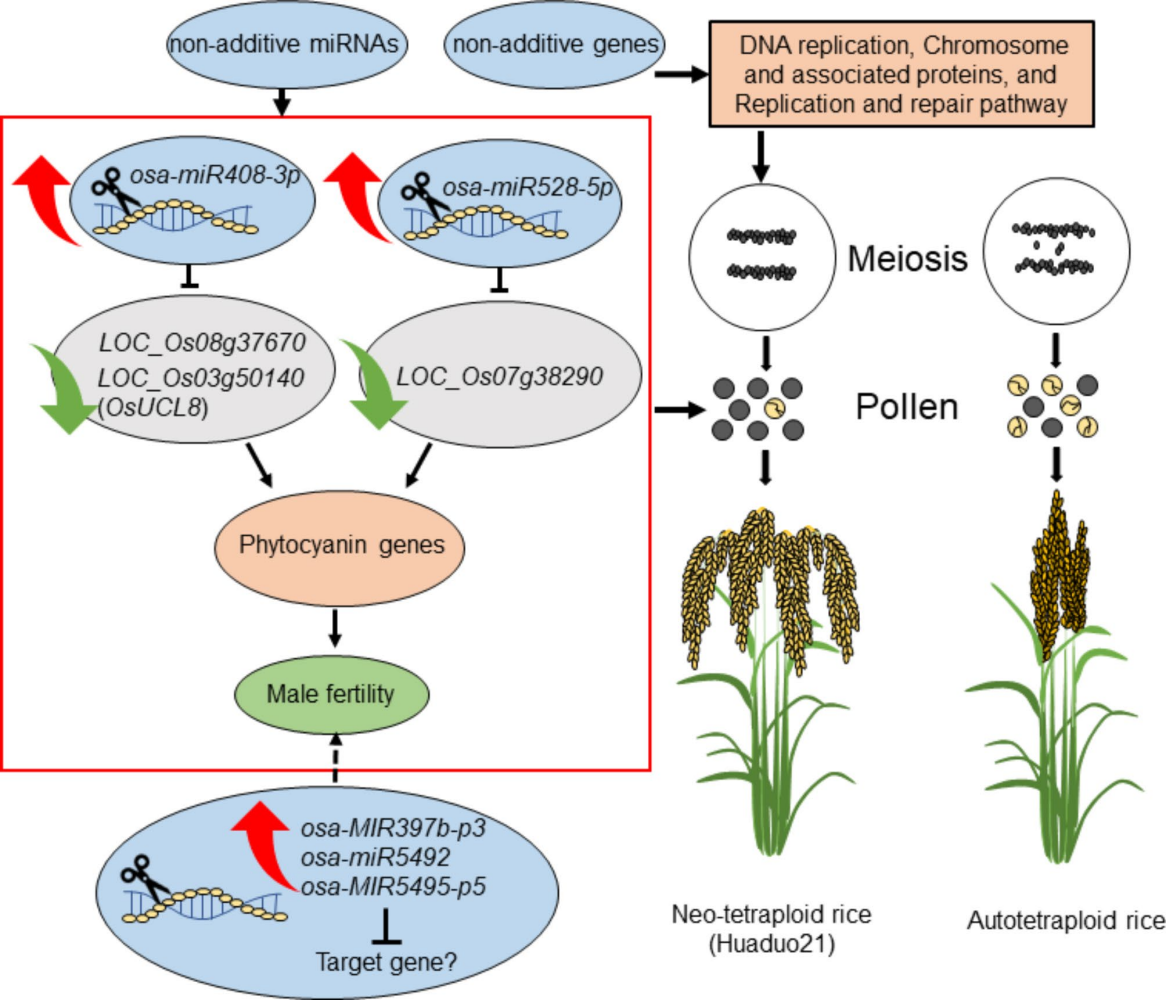
A hypothesis model of miRNA and target gene regulation of fertility in neo-tetraploid rice Huaduo21 (H21). The red arrow indicates the upregulated miRNA or target genes, and the green arrow indicates the downregulated miRNA or target genes. The black line means it cause an effect on the pollen fertility of H21. The dashed line indicated that miRNAs may play an important role in pollen fertility. The red box indicated the miRNA and target gene regulation of pollen fertility

In this study, the cytological observation of the Huaduo21 (H21) and its parents exhibited few chromosome abnormalities and lower abnormalities during the double fertilization and embryogenesis. Furthermore, high expression of fertility-related miRNAs might be responsible for the high fertility in H21, including *osa-miR408-3p*, *osa-miR528-5p*, and *osa-miR398b* (Fig. 8). This finding may provide the molecular basis of the miRNAs associated with fertility in rice, especially to better understand the potential regulatory mechanisms of high seed setting of neo-tetraploid rice.

### Methods

#### Rice material

Neo-tetraploid rice, Huaduo21 (H21) that was bred in our group, and autotetraploid rice, 96025-4x (T44), Jackson-4x (T45), 02428-4x, were used in this study. All the materials were self-crossed for more than 30 generations. They were planted at the experimental farm of South China Agricultural University under field conditions. The embryo after double fertilization of H21, 02428-4x, and 02428-2x, were collected for fertilization and embryo development analysis. During the pollen mother cell meiosis stage of H21, T44, and T45, the anthers were used for high throughput sequencing analysis. The anther sampling procedure followed the description provided by Wu et al. [12]. To determine the meiotic stage, a 1% acetocarmine staining method was performed. Additionally, the length of the anthers was measured for each material: T44 (5.5–6.5 mm), T45 (3.5–4.5 mm), and H21 (4.5–5.5 mm). For RNA-seq sample collection, the plants (tillers with inflorescences) were transferred from the field to the laboratory. Following the sampling standard, anthers (~ 600) from each material were collected and rapidly frozen in liquid nitrogen. The collected anthers were placed in Trizol Reagent (Invitrogen) and stored at -80 ℃ until RNA isolation.

### Cytological observation

Anthers and ovaries were collected and fixed in Formalin-Aceto-Alcohol (50% ethanol: formaldehyde: acetic acid = 90:5:5 v/v) for at least 48 h. The samples were stored in 70% ethanol at 4 °C after washing with 95% ethanol twice. To investigate the variation and measure the size of the anther and ovary of Taichung65-4x and Taichung65-2x, a whole mount eosin B confocal laser scanning microscopy (WE-CLSM) was used according to Li et al., with minor modifications [13]. The dissected anthers and ovaries were hydrated in 50%, 30% ethanol and distilled water for 30 min each. After an eosin B staining procedure for 30 min, the samples were dehydrated in a series of ethanol solutions (10%, 30%, 50%, 70%, 90%, and 100% ethanol) for 30 min. Finally, the dehydrated samples were transferred into a mixture of ethanol and methyl salicylate (1:1) for 1 h, and then stored in pure methyl salicylate and observed under WE-CLSM. The embryo sac samples after fertilization were examined using a Leica SPE laser scanning confocal microscope (Leica Microsystems, Heidelberg, Germany), following the protocol described by Zeng et al. [66]. The excitation wavelength was 543 nm, and the emitted light was detected between 550 and 630 nm. Initially, a quick scan of the entire sample was performed to identify the potential position of the embryo sac. This was achieved by using the “Live” button in the “Acquire” menu of the LEICA CONFOCAL Software (Leica Application Suite Advanced Fluorescence, version 1.8.2, build 1465, Leica Microsystems). Subsequently, a scanning range of 6–8 μm was selected using the “Begin” and “End” buttons in the “Acquire” menu. Images of different focal planes of the embryo sac were captured, and a composite image, consisting of four to six images from different focal planes, was generated to visualize the structure of the embryo sac. To investigate the variations in double-fertilization and embryo development of 02428-2x, 02428-4x, and H21, WE-CLSM was used again and followed the above procedure. The meiosis chromosome behavior observation was performed according to Wu et al. [12].

### Small RNA library construction and sequencing

Total RNA was extracted from the anthers by using a Trizol reagent (Invitrogen, Carlsbad, CA, USA). Illumina’s TruSeq small RNA sample preparation Kits (San Diego, CA, USA) were used to construct small RNA libraries. Then the small RNA sequencing (36 bp) was performed on an Illumina Hiseq2500 at the LC-BIO (LC-Bio Technology CO., Ltd., Hangzhou, China) to the manufacturer’s protocol. Small RNA sequencing analysis was performed using ACGT101-miR-v4.2 (LC Sciences, Houston, Texas, USA) as previously described [17, 67–69]. First, the reads with common RNA families (snRNA, tRNA, snoRNA, rRNA), low complexity, repeats, junk, and adapter dimers were removed. Subsequently, the unique sequences (18–25 nt in length) were mapped to rice precursors in miRBase 21.0 by BLAST search to identify known miRNAs and novel 3p- and 5p- derived miRNAs. Length variation at both 3’ and 5’ ends and one mismatch inside of the sequence were allowed in the alignment. The unique sequences mapping to rice mature miRNAs in hairpin arms were identified as known miRNAs. The unique sequences mapping to the other arm of known rice precursor hairpin opposite to the annotated mature miRNA-containing arm were considered to be novel 5p- or 3p derived miRNA candidates. The remaining sequences were mapped to other plant species precursors in miRBase 21.0 by BLAST search, and the mapped pre-miRNAs were further BLASTed against the rice genomes (http://rice.plantbiology.msu.edu/pub/data/Eukaryotic_Projects/o_sativa/annotation_dbs/pseudomolecules/version_7.0/) to determine their genomic locations. The above two we defined as known miRNAs. The unmapped sequences were BLASTed against the rice genomes, and the hairpin RNA structures containing sequences were predicated from the flank 120 nt sequences using RNAfold software (http://rna.tbi.univie.ac.at/cgi-bin/RNAfold.cgi). The criteria for secondary structure prediction were [70]: (1) number of nucleotides in one bulge in stem (≤ 12), (2) number of base pairs in the stem region of the predicted hairpin (≥ 16), (3) cutoff of free energy (kCal/mol ≤ -15), (4) length of hairpin (up and down stems + terminal loop ≥ 50), (5) length of hairpin loop (≤ 200), (6) number of nucleotides in one bulge in mature region (≤ 4), (7) number of biased errors in one bulge in mature region (≤ 2), (8) number of biased bulges in mature region (≤ 2), (9) number of errors in mature region (≤ 4), (10) number of base pairs in the mature region of the predicted hairpin (≥ 12), (11) percent of mature in stem (≥ 80). Differentially expressed miRNAs based on normalized deep-sequencing counts was analyzed by selectively using Student *t*-test based on the experiments design. miRNAs with *P*-value < 0.05 and |log2 (fold change ratio)| > 1 were considered as differentially expressed miRNAs.

### Expression patterns of differentially expressed miRNAs (DEM)/differentially expressed genes (DEG)

The expression patterns of DEM/DEG were defined according to Chen et al. [22]. The average expression level value of both parental lines (T45 and T44) is MPV (mid-parental value). If the expression level of miRNAs/genes in H21 was significantly (|log_2_ (Fold Change ratio)| > 1 and *q-value* (*p*-values corrected by Benjamini-Hochberg method) *<* 0.05) different from MPV, these miRNAs/genes were defined as non-additive miRNAs (NAM)/non-additive genes (NAG), if there was a non-significant difference between H21 and MPV, these miRNAs/genes were defined as additive miRNAs/genes. Classification of DEM/DEG was performed according to the expression of H21 relative to T45 and T44. “>” and “<” represents statistically higher or lower, and “=” represents statistically similar. If H21 > T45 > T44, or H21 > T44 > T45, then expression patterns of these miRNAs/genes were considered as higher than both parents (HP); if H21 = T45 > T44, or H21 = T45 < T44, then expression patterns of these miRNAs/genes were considered as close to T45 (CT45); if, H21 = T44 > T45 or H21 = T44 < T45, then expression patterns of these miRNAs/genes were considered as close to T44 (CT44); if T45 > H21 > T44, orT44 > H21 > T45, then expression patterns of these miRNAs/genes were considered as between two parents (BP); if H21 < T45 = T44, or H21 < T45 < T44, or H21 < T44 < T45, these miRNAs/genes were considered as lower than both parents (LP). The Heatmap diagram was drawn by TBtools [71].

### Transcriptome library construction and sequencing

Total RNA was extracted from the anthers by using a Trizol reagent (Invitrogen, CA, USA) following the manufacturer’s procedure. The quantity and purity of total RNA were analyzed by Bioanalyzer 2100 and RNA 6000 Nano LabChip Kit (Agilent, CA, USA) with RIN number > 7.0. Approximately 10 ug of total RNA representing a specific adipose type was subjected to isolate Poly (A) mRNA with poly-T oligo attached magnetic beads (Invitrogen, Massachusetts, USA). Following purification, the mRNA is fragmented into small pieces using divalent cations under elevated temperature (Magnesium RNA Fragmentation Module (NEB, Massachusetts, USA)). Then the cleaved RNA fragments were reverse-transcribed to create the final cDNA library following the protocol for the mRNA Seq sample preparation kit (Illumina, San Diego, USA). The average insert size for the paired-end libraries was 300 bp (± 50 bp). The RNA-seq was performed on the Illumina HiSeq 4000 sequencing platform at LC-BIO (LC-Bio Technology CO., Ltd., Hangzhou, China) [22].

Reads obtained from the sequencing machines include raw reads containing adapters or low quality bases which will affect the following analysis. Thus, to get high quality clean reads, reads were further filtered by Cutadapt (https://cutadapt.readthedocs.io/en/stable/, version: cutadapt-1.9). The parameters were as follows: (1) removing reads containing adapters, (2) removing reads containing polyA and polyG, (3) removing reads containing more than 5% of unknown nucleotides (N), (4) removing low quality reads containing more than 20% of low quality (Q-value ≤ 20) bases. Then sequence quality was verified using FastQC (http://www.bioinformatics.babraham.ac.uk/projects/fastqc/, version: FastQC-0.10.1), including the Q20, Q30 and GC-content of the clean data.

Clean reads were aligned to the *Oryza sativa* reference genome using HISAT2 (https://daehwankimlab.github.io/hisat2/, version: hisat2-2.0.4) package [72], which initially remove a portion of the reads based on quality information accompanying each read and then maps the reads to the reference genome. HISAT2 allows multiple alignments per read (up to 20 by default) and a maximum of two mismatch when mapping the reads to the reference. HISAT2 build a database of potential splice junctions and confirms these by comparing the previously unmapped reads against the database of putative junctions.

The mapped reads from each sample were assembled using StringTie (http://ccb.jhu.edu/software/stringtie/, version: stringtie-1.3.0) with default parameters [73]. Then, all transcriptomes from all samples were merged to reconstruct a comprehensive transcriptome using gffcompare software (http://ccb.jhu.edu/software/stringtie/gffcompare.shtml, version: gffcompare-0.9.8). After the generation of the transcriptome, the StringTie [73] and ballgown [74] (http://www.bioconductor.org/packages/release/bioc/html/ballgown.html) were used to estimate the expression levels of all transcripts and perform expression abundance for mRNAs by calculating FPKM (fragment per kilobase of transcript per million mapped reads) value. FPKM = [total_exon_fragments / mapped_reads(millions) × exon_length (kB). The differentially expressed genes were selected with |log_2_ (Fold Change ratio)| > 1 and with statistical significance (*p*-value < 0.05 and *q*-value < 0.05) by R package-ballgown. GO enrichment analysis was conducted using TBtools [71]. The KEGG database was used to determine the metabolic pathways associated with differentially expressed genes [29, 75]. Heatmap, GO (selected with *p*-value < 0.05 and *q*-value < 0.05), and KEGG (selected with *p*-value < 0.05 and *q*-value < 0.05) diagrams were drawn by TBtools [71].

### Degradome library construction and sequencing

The anthers total RNA was balanced mix with H21, T44, and T45, and approximately 20 ug of total RNA (RIN number > 7.0) was used to prepare the Degradome library. The method was followed described by Ma *et al*. [76] with some modifications [77]: (1) Approximately 150 ng of poly (A) + RNA was used as input RNA and annealed with Biotinylated Random Primers. (2) Strap avidin capture of RNA fragments through Biotinylated Random Primers. (3) 5’ adaptor ligation to only those RNAs containing 5’-monophosphates. (4) Reverse transcription and PCR. (5) Libraries were sequenced using the 5’ adapter only, resulting in the sequencing of the first 36 nucleotides of the inserts that represented the 5’ ends of the original RNAs. And then, we performed the single-end sequencing (36 bp) on an Illumina Hiseq2500 at LC-BIO (LC-Bio Technology CO., Ltd., Hangzhou, China) following the vendor’s recommended protocol.

Raw sequencing reads were processed using the ACGT101-DEG-v3.1 (LC Sciences, Houston, Texas, USA) to remove low-quality reads, reads with adaptor and primer contamination, and reads that can be annotated as non-coding RNA families. The extracted sequencing reads were then used to identify potentially cleaved targets by the CleaveLand pipeline [43] and the ACGT101-DEG-v3.1 (LC Sciences, Houston, Texas, USA). The degradome reads were mapped to the transcripts from transcriptome sequencing in this study. Only the perfect matching alignment(s) for the given read would be kept for degradation analysis. A 26-nt long ‘query’ mRNA subsequence was generated by extracting 13-nt long sequences upstream and downstream of the location of the 5’-end of the matching degradome sequence [43]. All resulting reads of ‘query’ mRNA were reverse complemented and aligned to the miRNA identified in this study. Alignments where the degradome sequence position coincident with the tenth or eleventh nucleotide of miRNA were retained, and were scored (score ≤ 4) according to a previously described scheme developed for plant miRNA/target pairings [78]. The target was selected and categorized as 0, 1, 2, 3, or 4, as described by Sun *et al*. [40]. Category 0 was defined as having over one raw read at the position, abundance at the position that was equal to the maximum on the transcript, and only one maximum on the transcript. Category 1 was defined as having over one raw read at the position, abundance at the position that was equal to the maximum on the transcript, and more than one maximum position on the transcript. Category 2 was defined as having over one raw read at the position and abundance at the position that was less than the maximum but higher than the median for the transcript. Category 3 was defined as having over one raw read at the position and abundance at the position that was equal to or less than the median for the transcript. The remaining sequences, which included only one raw read at the position, were defined as category 4. In addition, to easily analyze the miRNA targets and RNA degradation patterns, *t*-plots were built according to the distribution of abundances along these transcripts [79]. GO (selected with *p*-value < 0.05 and *q*-value < 0.05) and KEGG (selected with *p*-value < 0.05 and *q*-value < 0.05) diagrams were drawn by TBtools [71].

### Quantitative real-time qRT-PCR analysis

A total of 30 differentially expressed miRNAs (DEM) were selected for validation of miRNA-seq data by qRT-PCR. Total RNA was taken from sequenced samples, and the first-strand cDNA was synthesized using the Mir-X™ miRNA First-Strand Synthesis kits (Takara, Otsu, Shiga, Japan) according to the manufacturer’s instructions [80]. Mir-X miRNA qRT-PCR SYBR Kits use a single-step, single-tube reaction to produce first-strand cDNA, which is then specifically and quantitatively amplified using a miRNA-specific primer. The miRNA-specific 5’ primers of the DEM were designed following the manufacturer [80]. The mRQ 3’ Primer was provided from the kit itself. A total of 8 differentially expressed genes (DEG) were selected to validate the relationship between miRNAs and targets by qRT-PCR. The DEG primers were designed based on the cleavage site of miRNAs by using Primer Premier 5.0 software. The first-strand cDNA was synthesized using the PrimeScript™ RT reagent kit (Takara, Otsu, Shiga, Japan) according to the manufacturer’s instructions.

Real-time PCR was performed using SsoAdvanced Universal SYBR Green Supermix (Bio-RAD, Hercules, CA, USA) to amplify the PCR products. Real-time PCR conditions were conducted following the manufacturer’s instructions, then performed on the Lightcycler480 system (Roche). *U6* and *ubiquitin* (*LOC_Os03g13170*) were used as internal control genes for miRNAs and gene validation. All qRT-PCR reactions were performed in three biological replicates and three technical replicates, and the results are presented as the mean ± standard deviations. The 2^–*ΔΔCT*^ method was employed to calculate the relative expression level [81]. All the primers are listed in Supplementary Information Table S11.

### Plasmid construction and transgenic analysis

Six miRNAs were used for rice transgene analysis, including *osa-MIR397b-p3*, *osa-miR5492*, *osa-MIR5495-p5*, *osa-miR528-5p*, *osa-MIR818d-p3_2ss13GT17GT*, and *PC-3p-29913_234*. To construct the miRNA repressive-expressed vector, Short Tandem Target Mimic (STTM) approach was applied and followed the description by Yan et al. [82]. The STTM sequence of *osa-MIR397b-p3*, *osa-miR5492*, *osa-MIR5495-p5*, *osa-miR528-5p*, *osa-MIR818d-p3_2ss13GT17GT*, and *PC-3p-29913_234* were synthesized by Tsingke Biotechnology Co., Ltd. (Beijing, China). *osa-miR528-5p-STTM*, *osa-MIR818d-p3_2ss13GT17GT-STTM*, and *PC-3p-29913_234-STTM* were digested by *KpnI* and *HindIII* and fused into the POX vector [83] (Supplementary Information Fig. S10). These constructs were transferred to agrobacterium *EHA105* and then were transformed into rice callus (Neo-tetraploid rice Huaduo1). *osa-MIR397b-p3-STTM*, *osa-miR5492-STTM*, *osa-MIR5495-p5-STTM* were digested by *HindIII* and *BamHI* and fused into the POX vector. These constructs were transferred to agrobacterium *EHA105* and then were transformed into rice callus (*Nipponbare*) (Supplementary Information Table S11). At least three positive transgene lines of each material were used for trait observation.

### Electronic supplementary material

Below is the link to the electronic supplementary material.

Supplementary Material 1

## Data Availability

Transcriptome, small RNA, and Degradome sequencing data are available from the NCBI under the accession number PRJNA955405 (https://www.ncbi.nlm.nih.gov/bioproject/PRJNA955405). All data supporting the conclusions described here are provided in tables, figures, and Supplementary Material.
