# Supplementary material for: Cytological observation and RNA-seq analysis reveal novel miRNAs high expression associated with the pollen fertility of neo-tetraploid rice
Source: BMC Plant Biol. 2023 Sep 18;23:434. doi: 10.1186/s12870-023-04453-y (PMC10506311; doi:10.1186/s12870-023-04453-y)
Supplement: Supplementary file 1 — Supplementary Material 1 [file 12870_2023_4453_MOESM1_ESM.pdf]

Supplementary Material

Fig. S1. The phenotype of Huaduo21 (H21) and its parents.
Fig. S2. Defects of the embryo sac, fertilization, and embryo and endosperm development in autotetraploid rice.
Fig. S3. Fertilization rate abnormalities between neo-tetraploid rice and autotetraploid rice.
Fig. S4. Validation of the DEM (differentially expressed miRNAs) between neo-tetraploid rice and autotetraploid rice.
Fig. S5. Validation of differentially expressed miRNAs and their corresponding target genes.
Fig. S6. Heatmap of non-additive genes between Huaduo21 (H21) and its parents (T44 and T45).
Fig. S7. Gene Ontology (GO) and Kyoto Encyclopedia of Genes and Genomes (KEGG) enrichment analysis of the miRNA's targets by degradome sequencing.
Fig. S8. The phenotype of transgenic plants of fertility-related miRNAs in T2 generation (*japonica* variety Nipponbare background).
Fig. S9. The giant and dull endosperm was found in Huaduo21 among the neo-tetraploid rice.
Fig S10. Original images of agarose electrophoresis of the recombinant plasmid.
Table S1. Genetic variation in agronomic traits of neo-tetraploid rice Huaduo21 and its parents.
Table S2. Heterosis analysis of hybrids generated by the crossing of H21 and autotetraploid rice lines.
Table S3. The differentially expressed miRNAs (DEM) uniquely belong to H21 compared to T44 and T45.
Table S4. The non-additive miRNAs in this study.
Table S5. Overview of transcriptome quality reads in this study.
Table S6. The differentially expressed genes (DEG) uniquely belong to H21 compared to T44 and T45.
Table S7. The non-additive genes in this study.
Table S8. Overview of raw data from degradome sequencing.
Table S9. The results of the targets were identified with degradome analysis in anthers of H21, T44, and T45.
Table S10. The candidate miRNA–target pairs are associated with fertility in the present study.
Table S11. The primers used in this study.

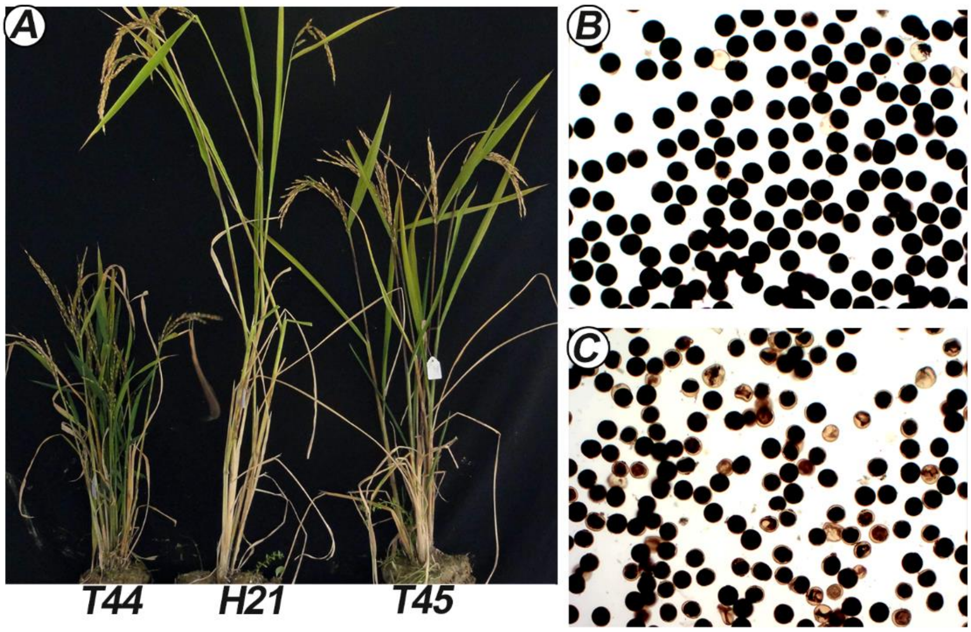

Fig. S1. The phenotype of Huaduo21 (H21) and its parents. (A) Phenotype of Huaduo21 and its parents (T44, H21, T45). (B) Mature pollen of Huaduo21. (C) Mature pollen of T44. ‘T44’ and ‘T45’ indicated the autotetraploid rice 96025-4x and Jackson-4x, respectively. ‘H21’ indicated the neo-tetraploid rice Huaduo21.

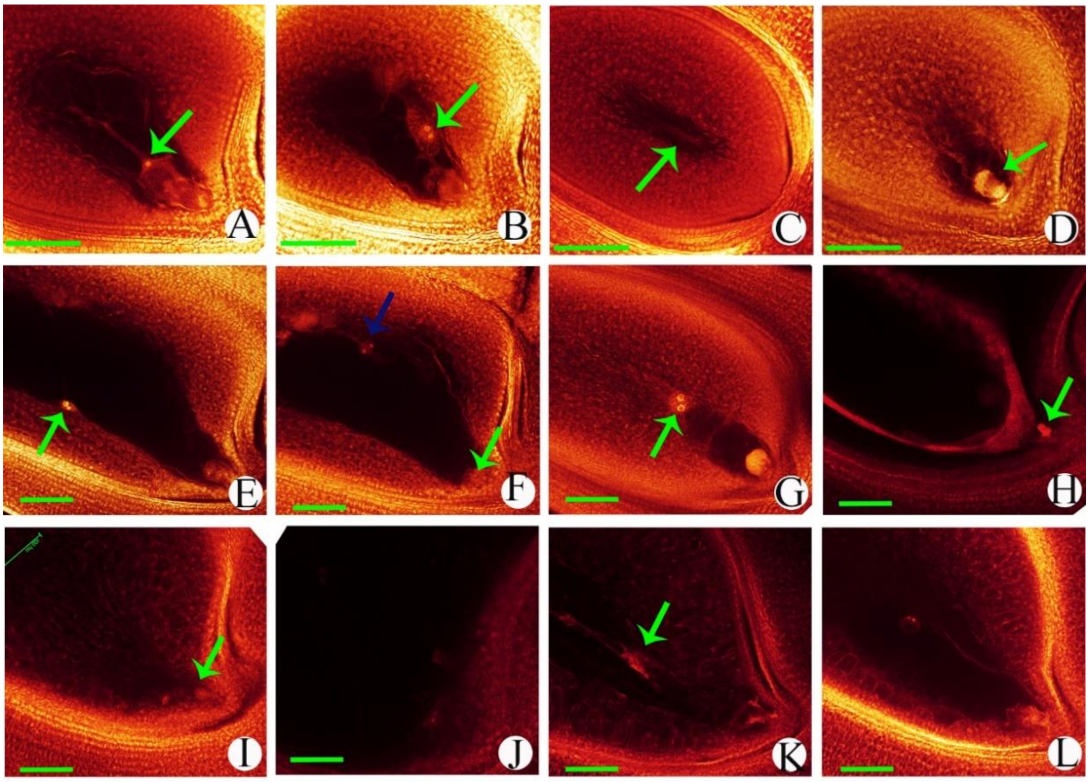

Fig. S2. Defects of the embryo sac, fertilization, and embryo and endosperm development in autotetraploid rice. (A-D) Abnormal embryo sac leads to unsuccessful fertilization at 5 min and 1 hour after flowering. (A, B) Abnormal polar nuclei in number (A) and position (B) were identified in the embryo sac 5min after flowering. The green arrow indicates the polar nuclei. (C) degeneration of embryo sac at 5min after flowering (green arrow). (D) degeneration of egg cells and polar nuclei at 1 hour after flowering (green arrow). (E) the zygote underwent cell division, while the polar nuclei were not fertilized (green arrow), and no free nuclei were observed 1 day after flowering. (F) unfertilization of the polar nuclei (blue arrow) and egg nucleus (green arrow) were identified, and no free nuclei were observed 1 day after flowering. (G) the globular embryo was found at the micropyle pole; however, polar nuclei (green arrow) were unfertilized 3 days after flowering. (H) embryo degeneration at 3 days after flowering (green arrow). (I) ovary enlarged without double-fertilization at 3 days after flowering (green arrow). (J) ovary enlarged without double-fertilization at 5 days after flowering. (K) endosperm and embryo were arrested at 5 days after flowering (green arrow). (L) unfertilization of the polar nuclei and egg nucleus at 5 days after flowering. Bar = 100 μm.

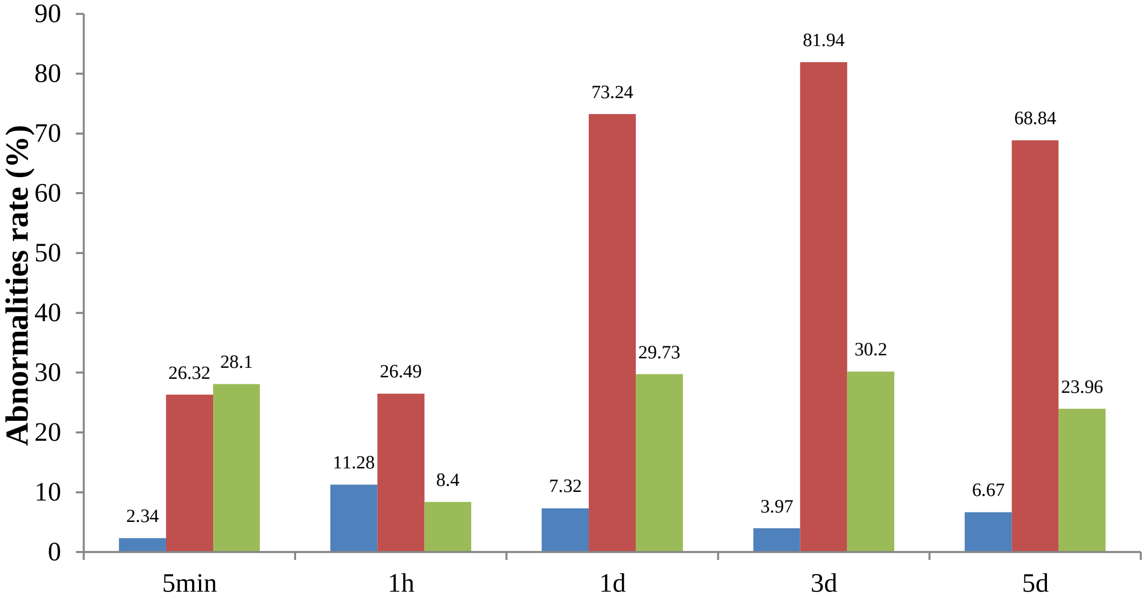

Fig. S3. Fertilization rate abnormalities between neo-tetraploid rice and autotetraploid rice. Blue, red, and green columns indicate diploid rice (02428-2x), autotetraploid rice (02428-4x), and neo-tetraploid rice (Huaduo21), respectively. The sample size of each stage was more than 100.

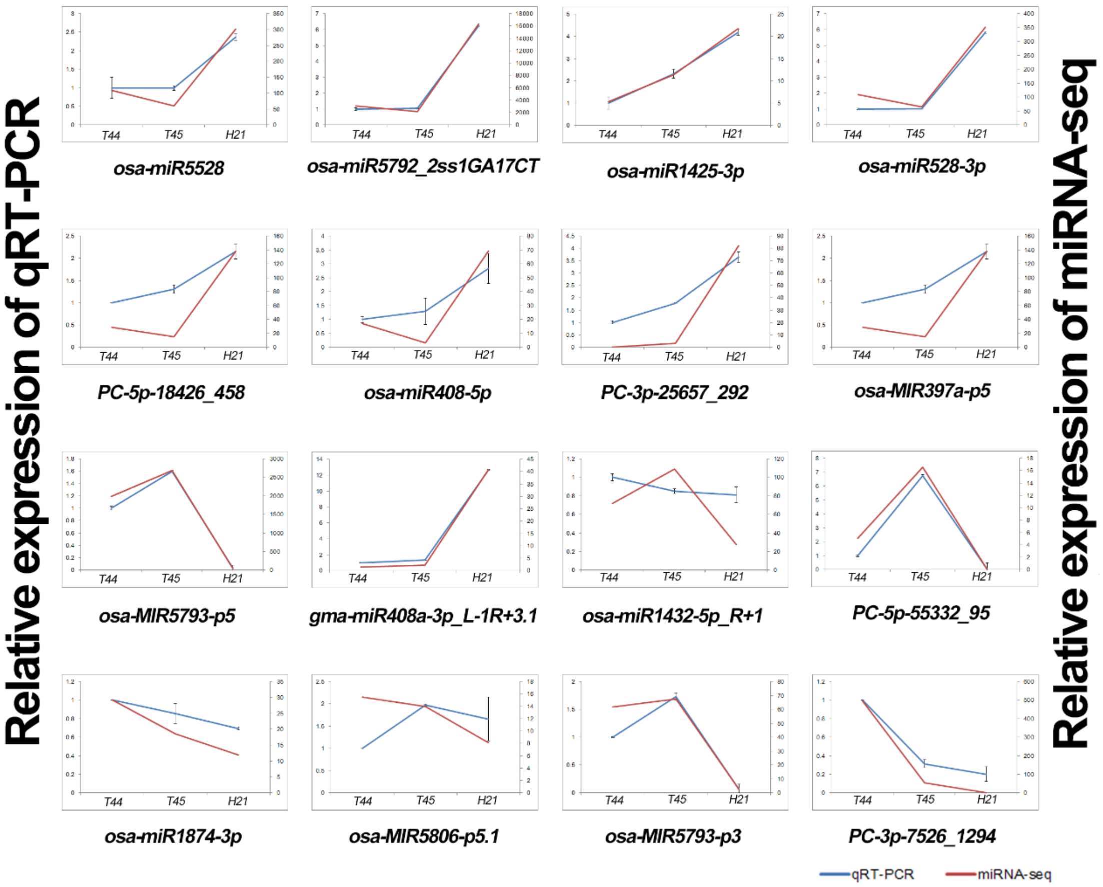

Fig. S4. Validation of the DEM (differentially expressed miRNAs) between neo-tetraploid rice and autotetraploid rice. *U6* snRNA was used as an internal reference for the qRT-PCR. The blue and red lines represent the relative expression levels of qRT-PCR and miRNA-sequences date, respectively. qRT-PCR was performed in three biological replicates and three technical replicates. Error bars represent the standard deviation (SD).

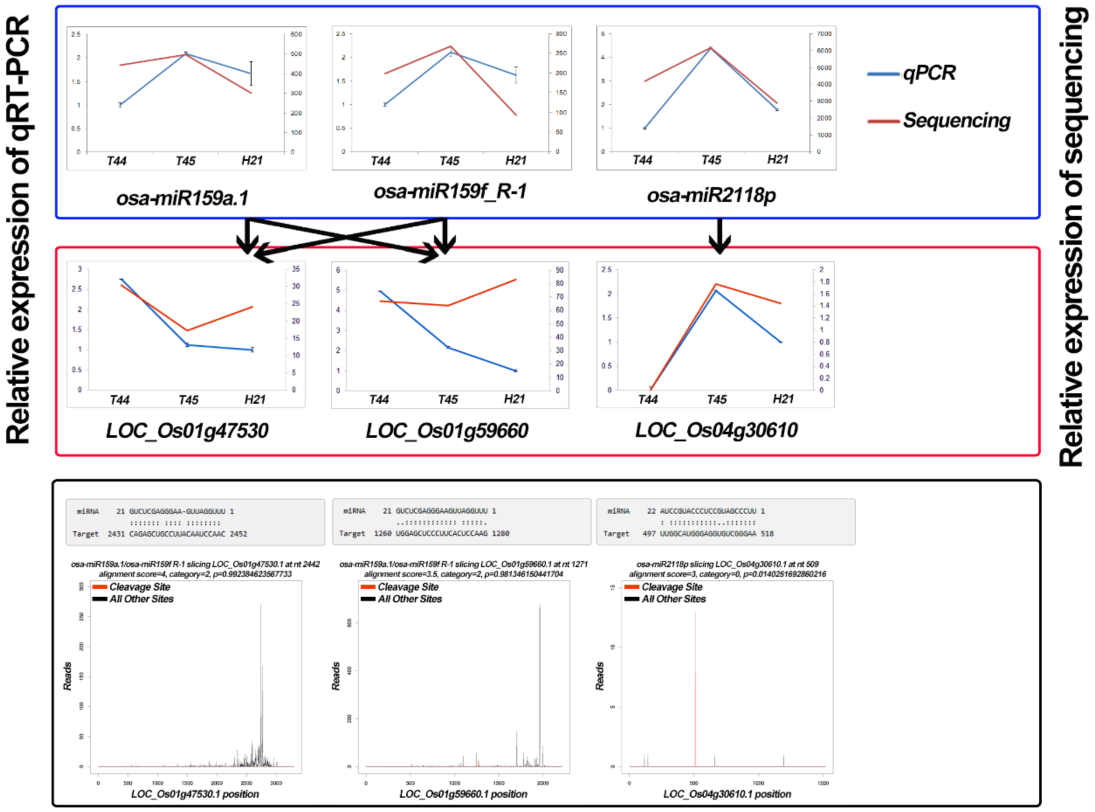

Fig. S5. Validation of differentially expressed miRNAs and their corresponding target genes. The blue and red box contents represent the qRT-PCR results of miRNAs and target genes, respectively. The blue and red lines represent the relative expression levels of qRT-PCR and sequencing data (miRNA/RNA), respectively. qRT-PCR was performed in three biological replicates and three technical replicates. Error bars represent the standard deviation (SD). The black arrow indicates the miRNA-target pairs. The content within the black box represents the t-plot and cleavage site between miRNAs and their targets. The alignment between representative miRNAs and their targets is shown below the corresponding *t*-plot. Red lines indicate the signature produced by miRNA-directed cleavage in the *t*-plot.

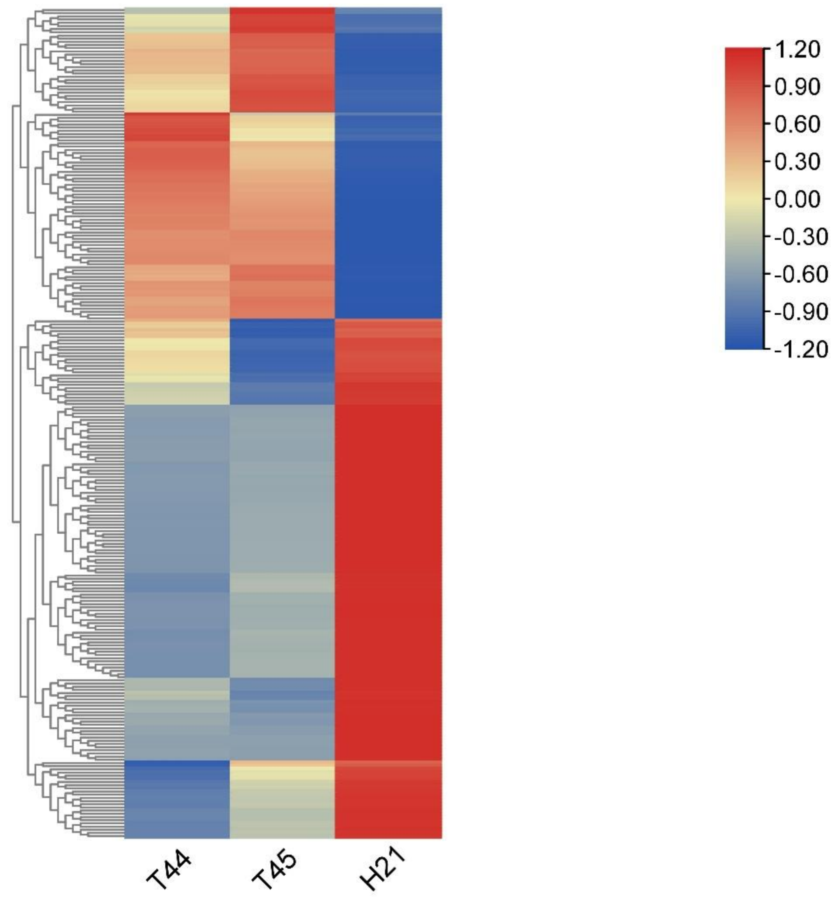

Fig. S6. Heatmap of non-additive genes between Huaduo21 (H21) and its parents (T44 and T45). The scale bar indicates the relative expression levels of genes (log_2).

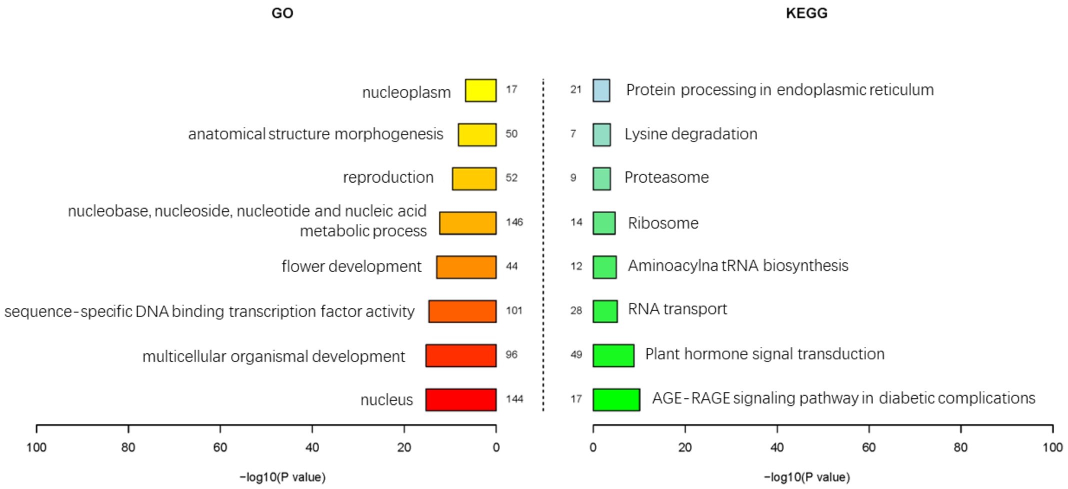

Fig. S7. Gene Ontology (GO) and Kyoto Encyclopedia of Genes and Genomes (KEGG) enrichment analysis of the miRNA's targets by degradome sequencing. The X-axis indicates the -log10 (P-value) of GO terms, and the Y-axis indicates the numbers of enrichment genes corresponding to GO terms and pathways, respectively. *p*-value < 0.05 and *q-value* < 0.05.

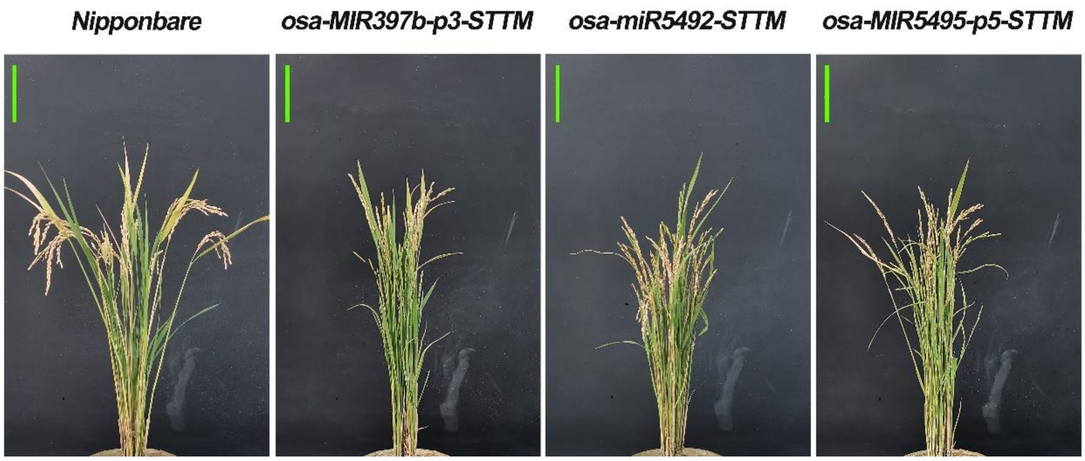

Fig. S8. The phenotype of transgenic plants of fertility-related miRNAs in T2 generation (*japonica* variety Nipponbare background). Bar: 10cm.

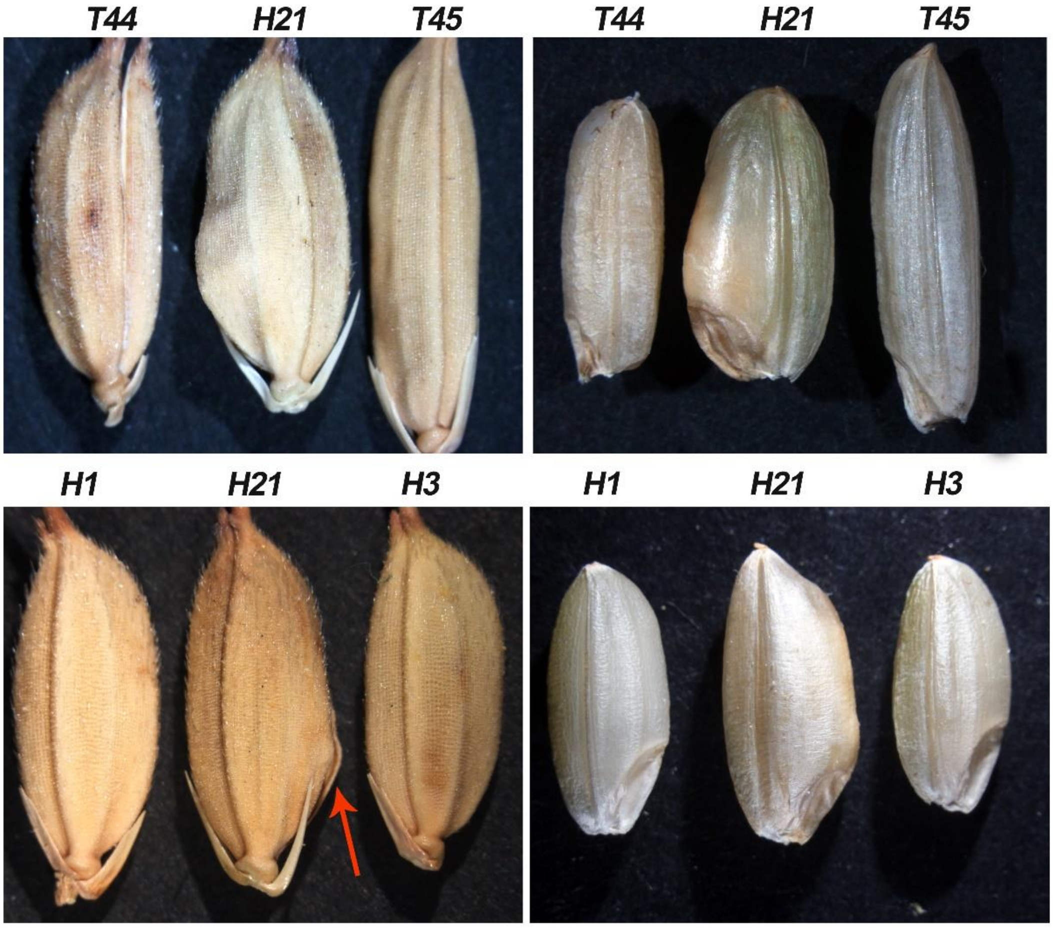

Fig. S9. The giant and dull endosperm was found in Huaduo21 among the neo-tetraploid rice. T44 and T45 indicate the autotetraploid rice 96025-4x and Jackson-4x, respectively. H1, H21 and H3 indicate the neo-tetraploid rice Huaduo1, Huaduo21 and Huaduo3 respectively. Red arrow: the glumes were dehiscence due to the puffed endosperm in Huaduo21.

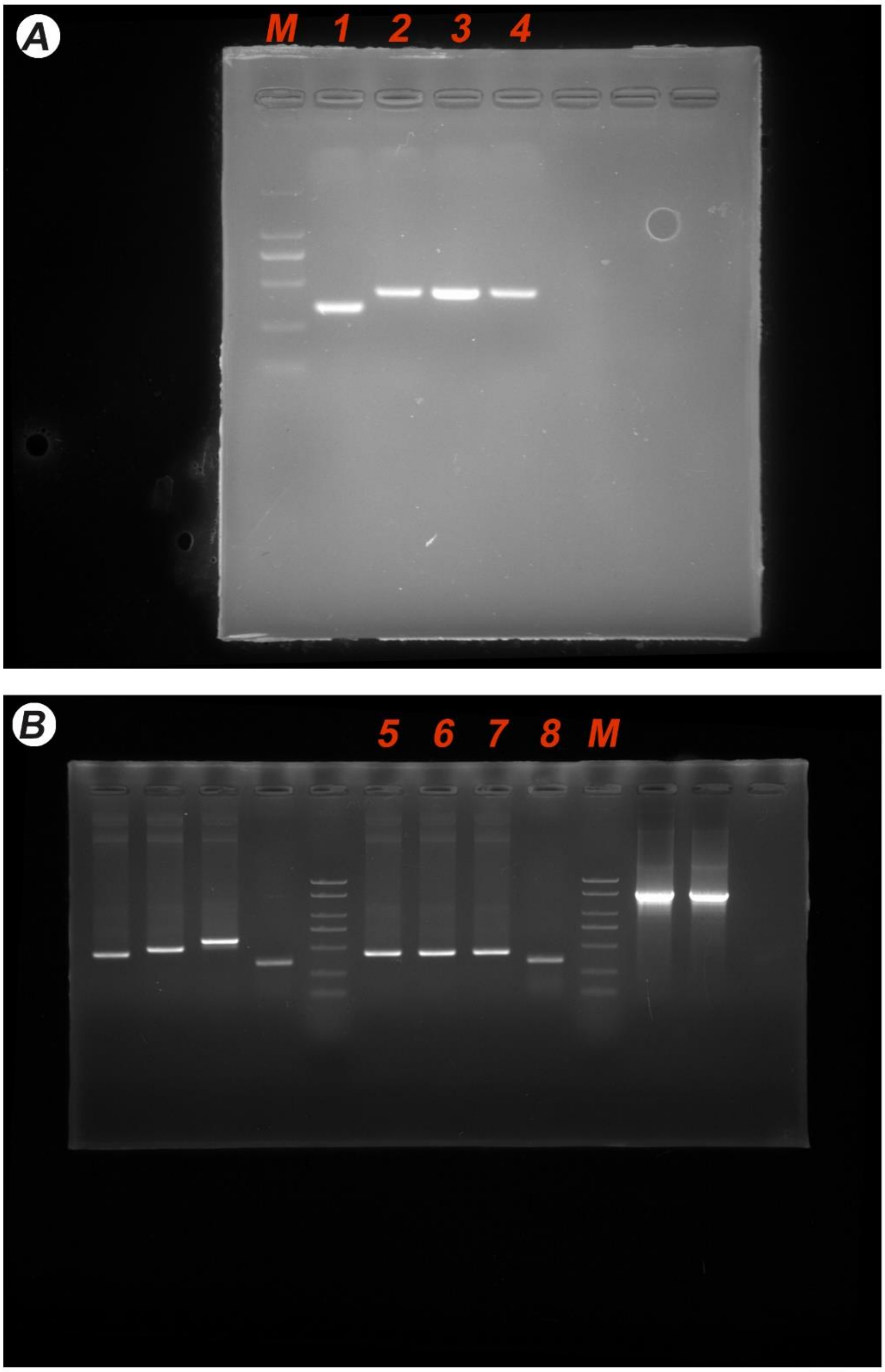

Fig S10. Original images of agarose electrophoresis of the recombinant plasmid. M indicated the D2000 marker. Line 1 and 8 indicated the POX vector. Line 2-4 in Panel A indicated *osa-miR528-5p-STTM*, *osa-MIR818d-p3_2ss13GT17GT-STTM*, and *PC-3p-29913_234-STTM*, respectively. Line 5-7 in Panel B indicated *osa-MIR397b-p3-STTM*, *osa-miR5492-STTM*, *osa-MIR5495-p5-STTM*, respectively. The remaining bands were not related to this study. The samples in Panel A and Panel B originate from the same experiment, respectively, and the gels were processed in parallel.

Table S1. Genetic variation in agronomic traits of neo-tetraploid rice Huaduo21 and its parents.

**Seasons**	Early season in 2015			Late season in 2015		
**Material**	T44	T45	H21	T44	T45	H21
**Plant height/cm**	96.16±4.27c	117.58±2.87b	144.53±3.45a	78.05±3.69c	93.83±2.43b	122.44±8.53a
**Effective panicle number**	5.84±1.54a	4.53±1.22b	3.95±0.85b	5.59±1.99a	3.50±1.20b	4.78±1.30a
**Panicle length/cm**	28.97±2.27b	31.70±2.10a	27.83±2.84b	24.61±1.95ab	25.95±2.80a	22.94±1.11b
**The number of filled grains per plant**	185.37±68.45b	191.53±65.95b	387.47±80.41a	86.24±49.56c	223.28±95.99b	334.67±84.84a
**The number of grains per plant**	461.74±135.21b	587.68±172.48a	551.16±109.27a	303.64±110.91a	400.89±168.79a	401.83±97.62a
**Seed set ratio/%**	40.26±8.00b	31.82±3.96c	70.36±5.82a	26.64±8.96c	56.04±8.23b	83.33±4.34a
**Grain weight per plant/g**	5.95±2.33b	5.73±1.97b	13.72±3.00a	2.81±1.58c	6.64±2.93b	12.76±2.53a
**1000-grain weight/g**	31.95±1.95c	29.99±2.29b	35.32±1.31a	31.66±1.17b	29.55±1.25c	38.76±3.52a

Note: The data were analyzed by the SSR method (Duncan) with P-value = 0.05.

Table S2. Heterosis analysis of hybrids generated by the crossing of H21 and autotetraploid rice lines.

**Index**	**Material**	**Plant height/%**	**panicle nu**	**Panicle length/%**	**The number of filled grains per plant/%**	**The number of grains per plant/%**	**Seed set ratio/%**	**Grain weight per plant/%**	**1000-grain weight/%**
Mid-parent heterosis	T410×H21	11.29	95.84	14.68	175.55	117.16	27.78	141.23	0.57
	T431×H21	11.39	52.09	26.04	166.42	74.72	96.45	166.15	1.57
	T45×H21	3.01	8.57	-3.17	53.78	2.77	48.58	52.89	2.88
	Mean	8.56	52.17	12.52	131.92	64.88	57.61	120.09	1.67
Heterobeltiosis (compared with better parent)	T410×H21	9.84	79.58	9.67	138.92	109.88	14.48	141.21	0.08
	T431×H21	-4	41.3	18.22	40.5	28.41	8.86	40.1	-0.28
	T45×H21	-6.59	1.63	-9.09	14.9	-1.39	7.89	8.38	-4.88
	Mean	-0.25	40.84	6.27	64.77	45.63	10.41	63.23	-1.69

Table S3. The differentially expressed miRNAs (DEM) uniquely belong to H21 compared to T44 and T45.

miRNA id	Note
PC-5p-118418_26	DEM uniquely in H21 compared to T44
osa-miR1874-3p	DEM uniquely in H21 compared to T44
osa-miR1846e_L-1R+4	DEM uniquely in H21 compared to T44
PC-3p-115181_28	DEM uniquely in H21 compared to T44
PC-3p-107648_32	DEM uniquely in H21 compared to T44
osa-miR171d-5p	DEM uniquely in H21 compared to T44
osa-miR2118d	DEM uniquely in H21 compared to T44
osa-MIR2863a-p5	DEM uniquely in H21 compared to T44
osa-miR531a_R-2	DEM uniquely in H21 compared to T44
osa-MIR2118j-p5	DEM uniquely in H21 compared to T44
osa-MIR2118m-p5	DEM uniquely in H21 compared to T44
osa-miR5082_L-1	DEM uniquely in H21 compared to T44
PC-3p-19443_427	DEM uniquely in H21 compared to T44
PC-3p-123182_24	DEM uniquely in H21 compared to T44
osa-miR166e-3p	DEM uniquely in H21 compared to T44
PC-3p-100599_36	DEM uniquely in H21 compared to T44
osa-miR160d-3p_R+1	DEM uniquely in H21 compared to T44
PC-3p-91990_42	DEM uniquely in H21 compared to T44
osa-MIR5542-p5_2ss2AG21TC	DEM uniquely in H21 compared to T44
osa-MIR171b-p5	DEM uniquely in H21 compared to T44
osa-MIR2118f-p5_1ss7AT	DEM uniquely in H21 compared to T44
osa-MIR2863a-p3	DEM uniquely in H21 compared to T44
PC-5p-109489_31	DEM uniquely in H21 compared to T44
PC-5p-88465_45	DEM uniquely in H21 compared to T44
osa-miR159f_R-1	DEM uniquely in H21 compared to T45
PC-5p-73559_61	DEM uniquely in H21 compared to T45
osa-miR1432-5p_R+1	DEM uniquely in H21 compared to T45
bdi-MIR169f-p3	DEM uniquely in H21 compared to T45
osa-miR1849	DEM uniquely in H21 compared to T45
PC-5p-105828_33	DEM uniquely in H21 compared to T45
osa-miR171c-5p	DEM uniquely in H21 compared to T45
osa-miR396e-3p	DEM uniquely in H21 compared to T45
PC-3p-97312_38	DEM uniquely in H21 compared to T45
PC-5p-87356_46	DEM uniquely in H21 compared to T45
osa-MIR5796-p5	DEM uniquely in H21 compared to T45
PC-3p-84778_48	DEM uniquely in H21 compared to T45
PC-5p-29828_234	DEM uniquely in H21 compared to T45
osa-miR159a.1	DEM uniquely in H21 compared to T45
osa-miR1857-3p	DEM uniquely in H21 compared to T45
osa-miR5496	DEM uniquely in H21 compared to T45
osa-miR393b-3p	DEM uniquely in H21 compared to T45
osa-miR1859	DEM uniquely in H21 compared to T45
PC-5p-116714_27	DEM uniquely in H21 compared to T45
osa-MIR5509-p3	DEM uniquely in H21 compared to T45
osa-MIR169g-p3	DEM uniquely in H21 compared to T45
osa-MIR169j-p3	DEM uniquely in H21 compared to T45
osa-MIR169m-p3	DEM uniquely in H21 compared to T45
osa-MIR169h-p3	DEM uniquely in H21 compared to T45
osa-MIR169l-p3	DEM uniquely in H21 compared to T45
osa-miR5822	DEM uniquely in H21 compared to T45
gma-miR167e	DEM uniquely in H21 compared to T45
osa-MIR2118b-p5	DEM uniquely in H21 compared to T45
PC-3p-42098_142	DEM uniquely in H21 compared to T45
bdi-miR167a_R+1	DEM uniquely in H21 compared to T45
osa-miR1862d	DEM uniquely in H21 compared to T45
osa-MIR2118l-p5	DEM uniquely in H21 compared to T45

osa-miR156f-3p	DEM uniquely in H21 compared to T45
zma-miR164a-5p_L+1	DEM uniquely in H21 compared to T45
PC-3p-50344_110	DEM uniquely in H21 compared to T45
PC-5p-114783_28	DEM uniquely in H21 compared to T45
PC-5p-67609_70	DEM uniquely in H21 compared to T45
osa-MIR5806-p5	DEM uniquely in H21 compared to T45
PC-3p-73051_62	DEM uniquely in H21 compared to T45
osa-MIR1868-p5	DEM uniquely in H21 compared to T45
osa-miR160a-3p	DEM uniquely in H21 compared to T45
osa-miR3980a-5p	DEM uniquely in H21 compared to T45
osa-miR5144-5p	DEM uniquely in H21 compared to T45
osa-miR2118b	DEM uniquely in H21 compared to T45
osa-MIR1861d-p3_1ss16AG	DEM uniquely in H21 compared to T45
PC-5p-60832_83	DEM uniquely in H21 compared to T45
osa-MIR397b-p5	DEM common in H21 compared to T44 and T45
osa-MIR5793-p3	DEM common in H21 compared to T44 and T45
PC-5p-112724_29	DEM common in H21 compared to T44 and T45
PC-5p-21700_368	DEM common in H21 compared to T44 and T45
osa-miR5144-3p_L-1R+1	DEM common in H21 compared to T44 and T45
osa-MIR1851-p5_2ss16CG17CT	DEM common in H21 compared to T44 and T45
gma-miR408a-3p_L-1R+3	DEM common in H21 compared to T44 and T45
mdm-miR408a_L-1R+3	DEM common in H21 compared to T44 and T45
osa-MIR5495-p3	DEM common in H21 compared to T44 and T45
osa-miR166a-5p	DEM common in H21 compared to T44 and T45
PC-3p-110736_30	DEM common in H21 compared to T44 and T45
osa-MIR397a-p5	DEM common in H21 compared to T44 and T45
PC-3p-40862_149	DEM common in H21 compared to T44 and T45
PC-3p-62520_79	DEM common in H21 compared to T44 and T45
PC-3p-25657_292	DEM common in H21 compared to T44 and T45
osa-miR5792_2ss1GA17CT	DEM common in H21 compared to T44 and T45
osa-miR5492	DEM common in H21 compared to T44 and T45
PC-3p-67976_69	DEM common in H21 compared to T44 and T45
osa-MIR5083-p3_1ss20GA	DEM common in H21 compared to T44 and T45
osa-miR398b	DEM common in H21 compared to T44 and T45
PC-5p-18426_458	DEM common in H21 compared to T44 and T45
PC-5p-25585_293	DEM common in H21 compared to T44 and T45
osa-miR169r-3p	DEM common in H21 compared to T44 and T45
osa-miR166l-5p	DEM common in H21 compared to T44 and T45
PC-5p-91931_42	DEM common in H21 compared to T44 and T45
PC-5p-63036_78	DEM common in H21 compared to T44 and T45
osa-miR1425-3p	DEM common in H21 compared to T44 and T45
osa-MIR1879-p5	DEM common in H21 compared to T44 and T45
PC-3p-110012_30	DEM common in H21 compared to T44 and T45
PC-5p-4750_1996	DEM common in H21 compared to T44 and T45
osa-MIR1851-p3	DEM common in H21 compared to T44 and T45
PC-3p-76343_57	DEM common in H21 compared to T44 and T45

Table S4. The non-additive miRNAs in this study.

ID	Squences	Classification
osa-MIR2122-p3_2ss18TC21TC	GCTGAGGTGTCTAAGTTCAGCGCC	The expression level of H21 statistically close to T45
PC-5p-118418_26	ACCTAGTACTGGATGTGACACATC	The expression level of H21 statistically close to T45
PC-5p-87616_45	AGAAATGGACGTCCGAGATTCGTC	The expression level of H21 statistically close to T45
osa-MIR1846d-p3	CGGTGACCCCGGTCTCCTCGC	The expression level of H21 statistically close to T45
osa-miR156c-3p	GCTCACTTCTCTCTCTGTCAGC	The expression level of H21 statistically close to T45
osa-miR1874-3p	TATGGATGGAGGTGTAACCCGATG	The expression level of H21 statistically close to T45
PC-3p-115181_28	ATACCTCGTTAGATTCGTCTCGCA	The expression level of H21 statistically close to T45
PC-3p-107648_32	AAGACGGACGGTCAAATATTGGAC	The expression level of H21 statistically close to T45
PC-5p-108927_31	AGCACCGTACCTTTGTTACTGACA	The expression level of H21 statistically close to T45
osa-MIR2863a-p5	TACCCATTGTCCCATTCTAGT	The expression level of H21 statistically close to T45
osa-miR531a_R-2	CTCGCCGGGGCTGCGTGCCGCC	The expression level of H21 statistically close to T45
PC-5p-40558_150	TACCCAAGAGGTCATGGGTTC	The expression level of H21 statistically close to T45
osa-miR395b_L-1	TGAAGTGTTTGGGGGAACTC	The expression level of H21 statistically close to T45
osa-miR5082_L-1	GCGATGATGGCCGCGCGGGTTCA	The expression level of H21 statistically close to T45
PC-3p-123182_24	AGATTTGTAGTACTGGGATACGTC	The expression level of H21 statistically close to T45
PC-3p-100599_36	CTTGAACCCATGACCTCTTGG	The expression level of H21 statistically close to T45
osa-miR160d-3p_R+1	GCGTGCGAGGAGCCAAGCATGA	The expression level of H21 statistically close to T45
osa-MIR5542-p5_2ss2AG21TC	TGGTGTAAAATTTAGTATCTC	The expression level of H21 statistically close to T45
osa-miR1846a-3p	TGACCCCGTTCTCCTCGCCGG	The expression level of H21 statistically close to T45
PC-5p-109489_31	ATCCTCTGACTTAACTGCACATGC	The expression level of H21 statistically close to T45
PC-5p-88465_45	AAAGTCCAATCCTAGCTACGAACC	The expression level of H21 statistically close to T45
PC-5p-149433_16	ATAAGCGTCGCCTGCGAAGATCGA	The expression level of H21 statistically close to T45
osa-MIR5522-p5_1ss1TC	CAAGAACAATAGGAATGGGAG	The expression level of H21 statistically close to T45
bdi-miR395f-5p_2ss12AG18TC	GTTCCCTTCAAGCACTTCACG	The expression level of H21 statistically close to T45
osa-MIR395b-p5_1ss6TC	GTTCCCTTCAAGCACTTTACG	The expression level of H21 statistically close to T45
osa-MIR395d-p5	GTTCCCTTCAAGCACTTCACG	The expression level of H21 statistically close to T45
osa-MIR395p-p5	GTTCCCTTCAAGCACTTCACG	The expression level of H21 statistically close to T45
osa-MIR395q-p5	GTTCCCTTCAAGCACTTCACA	The expression level of H21 statistically close to T45
osa-MIR395s-p5	GTTCCCTTCAAGCACTTCACG	The expression level of H21 statistically close to T45
osa-miR399a	TGCCAAAGGAGAATTGCCCTG	The expression level of H21 statistically close to T45
osa-MIR5148a-p3_2ss12TC21AG	AGGGGTAGAAACGTCATATCGTGT	The expression level of H21 statistically close to T45
osa-MIR5148b-p3_2ss12TC21AG	AGGGGTAGAAACGTCATATCGTGT	The expression level of H21 statistically close to T45
osa-MIR5148c-p3_2ss12TC21AG	AGGGGTAGAAACGTCATATCGTGT	The expression level of H21 statistically close to T45
PC-3p-139016_19	AGATTCATTGTACTAGGAGGGGTC	The expression level of H21 statistically close to T45
PC-3p-39232_158	AGATGGCTGATACTCGAACTC	The expression level of H21 statistically close to T45
PC-3p-79602_54	GCGTGCGGTTGTCTTAAGCGGACC	The expression level of H21 statistically close to T45
PC-5p-114735_28	AATGGCTTGTCTTGTTTTGTGTGC	The expression level of H21 statistically close to T45
PC-5p-123398_24	TCGGCCGAAATTTTTGAATTT	The expression level of H21 statistically close to T45
PC-5p-90636_43	GTTCGAGCATCAGCCATCTAC	The expression level of H21 statistically close to T45
osa-miR162a	TCGATAAACCTCTGCATCCAG	The expression level of H21 statistically close to T44
PC-3p-2416_3415	TTCAGTTTCCTCCAACATCTTA	The expression level of H21 statistically close to T44
zma-miR2275b-5p_2ss12CG20CT	AGGATTAGAGGGAACTGAATC	The expression level of H21 statistically close to T44
osa-miR3979-3p	CTTCGGGGGAGGAGAGAAGC	The expression level of H21 statistically close to T44
osa-miR159f_R-1	CTTGGATTGAAGGGAGCTCT	The expression level of H21 statistically close to T44
osa-MIR169i-p3	GGCAGTCTCCTTGGCTAGT	The expression level of H21 statistically close to T44
osa-MIR169k-p3	GGCAGTCTCCTTGGCTAGT	The expression level of H21 statistically close to T44
PC-5p-73559_61	TTTGTTACTAACCGGGACTAA	The expression level of H21 statistically close to T44
osa-MIR5529-p5	AACTAATTACATACCGGGGTA	The expression level of H21 statistically close to T44
osa-miR1432-5p_R+1	ATCAGGAGAGATGACACCGACA	The expression level of H21 statistically close to T44
osa-MIR5506-p3	TGACTGAACCCTAGGAGTGCT	The expression level of H21 statistically close to T44
PC-5p-34486_190	TAAGACGCCCGCATGCGAAAA	The expression level of H21 statistically close to T44
osa-miR156j-3p	GCTCGCTCCTCTTTCTGTCAGC	The expression level of H21 statistically close to T44
osa-MIR5521-p5	TGTTCTGCTTCCGTGCCTCTC	The expression level of H21 statistically close to T44
PC-5p-105828_33	CGTCCGAGATTCGTCCACGTC	The expression level of H21 statistically close to T44
osa-miR167d-3p	GATCATGCTGTGCAGTTTCATC	The expression level of H21 statistically close to T44
bdi-miR2275b	TTCAGTTTCTTCTAATATCTCA	The expression level of H21 statistically close to T44
osa-miR1432-3p	CAGGTGTCATCTCCCCTGAAC	The expression level of H21 statistically close to T44
PC-5p-29814_235	AGAGTTGGATGAAAACAAACT	The expression level of H21 statistically close to T44
PC-3p-44180_133	AGATCTATTTTCGCATGCGGC	The expression level of H21 statistically close to T44
osa-MIR2275a-p5	AGGTTTGGATGGGACCAAATC	The expression level of H21 statistically close to T44
osa-MIR2275b-p5	AGGTTTGGATGGGACCAAATC	The expression level of H21 statistically close to T44
osa-MIR156f-p3_2ss9AC19AG	AGAGAGAGCGATGAAATGGTG	The expression level of H21 statistically close to T44
PC-5p-87356_46	CAAGTCGTTTCTGATCGTTG	The expression level of H21 statistically close to T44
osa-MIR818d-p3_2ss13GT17GT	TATTATGGGACGTAGGTAGTA	The expression level of H21 statistically close to T44
PC-3p-41245_147	TCTGTGACGGGCCACAACTTG	The expression level of H21 statistically close to T44
PC-3p-84778_48	ATAAGTGGGTTTGCGGCTTAC	The expression level of H21 statistically close to T44
PC-5p-29828_234	TCTCGAGATACCGGTACCTCA	The expression level of H21 statistically close to T44
osa-MIR2118a-p5	GGACTGGGAACATATGAGAAAG	The expression level of H21 statistically close to T44
PC-3p-12301_759	TGAACAATTGGCATCTATGAT	The expression level of H21 statistically close to T44
PC-3p-21667_369	GTTGCACGGGTTTGTATGTTGCAG	The expression level of H21 statistically close to T44
osa-miR1423-3p_L+1R+3_1ss9AG	GAGCGCCCGAGCGGTAGTTGTCTCC	The expression level of H21 statistically close to T44
osa-MIR169i-p5	TGGTGATAAGGGTGTAGCTCTGG	The expression level of H21 statistically close to T44
PC-5p-116714_27	ATGTGGCGCTGAACTTAGACACCT	The expression level of H21 statistically close to T44
PC-5p-47982_118	CGGTACTAAATCGTTTATGAT	The expression level of H21 statistically close to T44
stu-MIR8005c-p5_1ss12AG	TAGGGTTTAGGGTTTAGGGTT	The expression level of H21 statistically close to T44
osa-miR1871	ATGGCTCTGATATCATGTTGGTTT	The expression level of H21 statistically close to T44
PC-5p-38615_162	ATGACATAAGACGTCGCCTGG	The expression level of H21 statistically close to T44
osa-miR1860-5p_R+3	AGAAAACCAGCTTCCAGATCTACA	The expression level of H21 statistically close to T44
osa-miR1862d	ACTAGGTTTGTTTATTTTGGGACG	The expression level of H21 statistically close to T44
PC-3p-49814_112	ACTAACCGGGACTAAAGTTGC	The expression level of H21 statistically close to T44
zma-miR164a-5p_L+1	CTGGAGAAGCAGGGCACGTGCA	The expression level of H21 statistically close to T44
PC-3p-50344_110	CGGCTGCCTTATGTGGACCGC	The expression level of H21 statistically close to T44
PC-5p-114783_28	CTAAAAATAGTTTTTAGTCCC	The expression level of H21 statistically close to T44
PC-5p-130015_22	ACATCCTAGTACTATGAATTTGGA	The expression level of H21 statistically close to T44
osa-MIR1868-p5	GCGTGCTCACGGAAAACGAGGGAG	The expression level of H21 statistically close to T44

PC-5p-63175_78	CGCATGCGAAAATAGATCTTC	The expression level of H21 statistically close to T44
PC-5p-60832_83	TGTAAATTTGGTATCTCTTGG	The expression level of H21 statistically close to T44
PC-3p-93349_41	TTTTGATCTATAAGTAGGTTC	The expression level of H21 statistically close to T44
osa-MIR5494-p3	TAATCCCATGCACGAGAACTG	The expression level of H21 statistically close to T44
PC-3p-144573_17	ATCGAGAATGTCACGGGGAATGTC	The expression level of H21 statistically close to T44
PC-3p-65267_74	AGGGACTTATACTTTTGTGGGAGG	The expression level of H21 statistically close to T44
PC-5p-70024_66	ATTTCATAGTATAACGAACCT	The expression level of H21 statistically close to T44
PC-5p-79082_54	TTGGCGCCGAACTTAGACACC	The expression level of H21 statistically close to T44
PC-3p-37346_169	TAACCTGTCAGAGGTGATGTT	The expression level of H21 statistically close to T44
PC-3p-131613_21	ACTAAAGGGGGTTATGAATCGGGA	The expression level of H21 statistically close to T44
PC-3p-71234_64	TTTGCTGGCGGCTAGCTTAAG	The expression level of H21 statistically close to T44
PC-3p-47378_120	TCTGTGACGAGCGCCAACTTT	The expression level of H21 statistically close to T44
PC-3p-30267_229	TTGCGGGACGGAGGGAGTACC	The expression level of H21 statistically close to T44
PC-3p-37383_169	TTTTCTTCAAACTTCAAAGTT	The expression level of H21 statistically close to T44
PC-3p-63442_78	TCGTAGTAGTAGAATGTGTCA	The expression level of H21 statistically close to T44
PC-5p-42507_140	TTATCCAACCGGGACTAAAGA	The expression level of H21 statistically close to T44
osa-MIR5494-p5	CCACGTCACTTTCTTTTTGCC	The expression level of H21 statistically close to T44
PC-3p-128049_23	ATCCAGATTCATACCTAGAGT	The expression level of H21 statistically close to T44
PC-3p-134526_20	TTTGTGATAGATCGTGCAACG	The expression level of H21 statistically close to T44
PC-3p-104001_34	TACTATAGGATTGGAATCCTC	The expression level of H21 statistically close to T44
PC-5p-152721_16	CGAGCCGGGAACCAATTTACT	The expression level of H21 statistically close to T44
osa-MIR159f-p5	AGCTCCCTTCGATCCAATCC	The expression level of H21 statistically close to T44
PC-3p-128988_22	ACTTATACTTTTGTGGGAGGGACT	The expression level of H21 statistically close to T44
PC-3p-27887_258	TTCGTAGCTAGGGTTGGACTT	The expression level of H21 statistically close to T44
osa-MIR817-p5_1ss5TA	TCAAACAGGCCTCAAGTTGGT	The expression level of H21 statistically close to T44
PC-3p-70247_66	TCGAGATATTTTTTGCTGGAC	The expression level of H21 statistically close to T44
gma-miR408a-3p_L-1R+3	TGCACTGCCTCTTCCCTGGCTTT	The expression level of H21 significantly higher than both parents
mdm-miR408a_L-1R+3	TGCACTGCCTCTTCCCTGGCTTT	The expression level of H21 significantly higher than both parents
osa-miR1425-3p	CAGCAAGAACTGGATCTTAAT	The expression level of H21 significantly higher than both parents
osa-MIR156e-p3	GCTCACTGCTCTTTCTGTCATC	The expression level of H21 significantly higher than both parents
osa-miR169r-3p	TGGCAAGTCTCCTCGGCTACC	The expression level of H21 significantly higher than both parents
osa-MIR1851-p3	ATGGCCGCCGGCGAGCTCTTC	The expression level of H21 significantly higher than both parents
osa-MIR1851-p5_2ss16CG17CT	CGGCCATGGCTAGCTGTCTCC	The expression level of H21 significantly higher than both parents
osa-MIR397a-p5	TTGAGTGCAGCGTTGATGAAC	The expression level of H21 significantly higher than both parents
osa-MIR397b-p3	TTCACCAGCACTGCACCCAATC	The expression level of H21 significantly higher than both parents
osa-MIR397b-p5	TTGAGTGCAGCGTTGATGAACC	The expression level of H21 significantly higher than both parents
osa-miR398b	TGTGTTCTCAGGTCGCCCCTG	The expression level of H21 significantly higher than both parents
osa-miR408-3p	CTGCACTGCCTCTTCCCTGGC	The expression level of H21 significantly higher than both parents
osa-miR408-5p	CAGGGATGAGGCAGAGCATGG	The expression level of H21 significantly higher than both parents
osa-miR528-3p	CCTGTGCTTGCCTCTTCCATT	The expression level of H21 significantly higher than both parents
osa-miR528-5p	TGGAAGGGGCATGCAGAGGAG	The expression level of H21 significantly higher than both parents
osa-miR5492	AGAAGGAGAATAGATATGGTT	The expression level of H21 significantly higher than both parents
osa-MIR5495-p3	CCATGGAGATCATTGTGACCT	The expression level of H21 significantly higher than both parents
osa-MIR5495-p5	TCTTACAGCCTTATAGCACAT	The expression level of H21 significantly higher than both parents
osa-miR5528	AAGACGGTTTTAGATGTTGCC	The expression level of H21 significantly higher than both parents
osa-MIR5528-p5_1ss4CA	TACAAAGGTCTGGTACTGCCC	The expression level of H21 significantly higher than both parents
osa-miR5792_2ss1GA17CT	AATGACAGCGGTGGTTTGGACATC	The expression level of H21 significantly higher than both parents
PC-3p-110012_30	TTACTCCCTGTTGGTAGTACC	The expression level of H21 significantly higher than both parents
PC-3p-110736_30	GGACTAAAGATAACGATGTTT	The expression level of H21 significantly higher than both parents
PC-3p-21233_379	TCTTAGATGGACCGCATGCGA	The expression level of H21 significantly higher than both parents
PC-3p-25552_293	TAGCTCGGCTCGGCTCATTTC	The expression level of H21 significantly higher than both parents
PC-3p-25657_292	TTCTCAAGGACCGTAGAATTA	The expression level of H21 significantly higher than both parents
PC-3p-67976_69	TTGGCTGCATCCCGTTCTCCTC	The expression level of H21 significantly higher than both parents
PC-3p-76343_57	TTTACTCCCGGTTGGTACTAC	The expression level of H21 significantly higher than both parents
PC-5p-112724_29	TTTGACCGTGTGTTGACCGTT	The expression level of H21 significantly higher than both parents
PC-5p-18426_458	TTTGACCGTGTGTTGACCGTTAAC	The expression level of H21 significantly higher than both parents
PC-5p-28716_248	AAGATCGATCTTCGCATGCGG	The expression level of H21 significantly higher than both parents
PC-5p-56309_93	TAACCGGGACTAAAGATTTTT	The expression level of H21 significantly higher than both parents
PC-5p-63036_78	TTGAGTAGATAGCATGAGGTA	The expression level of H21 significantly higher than both parents
PC-5p-91931_42	TGTGTTGACCGTTAACTCTCAAGT	The expression level of H21 significantly higher than both parents
osa-MIR169c-p3	GGCAAGTCTGTCCTTGGCTAC	The expression level of H21 significantly lower than both parents
osa-miR169e	TAGCCAAGGATGACTTGCCGG	The expression level of H21 significantly lower than both parents
osa-miR171e-5p	TGTTGGCTCGGCTCACTCAGA	The expression level of H21 significantly lower than both parents
osa-MIR1879-p5	CAACCCATCCCACCTCGTCCC	The expression level of H21 significantly lower than both parents
osa-miR2118o_1ss1CT	TTCCTGATGCCTCCCAAGCCTA	The expression level of H21 significantly lower than both parents
osa-miR5514	TCCCAGAGCTTTGGCCGTCGC	The expression level of H21 significantly lower than both parents
osa-MIR5793-p3	CTTTGCGTGTACCTTCTCTTG	The expression level of H21 significantly lower than both parents
osa-MIR5793-p5	CATCGCGGGACGGAAATCTTC	The expression level of H21 significantly lower than both parents
PC-3p-62520_79	TTACGTTGTAACTATATTGTAACT	The expression level of H21 significantly lower than both parents
PC-3p-7526_1294	TTGGGGAACGCGCCGATCGTC	The expression level of H21 significantly lower than both parents
PC-5p-21700_368	ATCATTTCTGATCGTTGGGTC	The expression level of H21 significantly lower than both parents
PC-5p-41957_143	CGATCAACGCGTTCCCCAATT	The expression level of H21 significantly lower than both parents
PC-5p-55332_95	AGAGTTGTAGGAAACTGAACT	The expression level of H21 significantly lower than both parents
peu-MIR2916-p3_1ss15TC	TGCCGACCAGGGATCGGCGGA	The expression level of H21 significantly lower than both parents
osa-MIR5817-p5_1ss21AG	GAAAGAAAAAGGTATACGAGGGGA	The expression level of H21 significantly lower than both parents
PC-5p-112416_29	GTCCGAGATTCGTCCACGTCACCA	The expression level of H21 significantly lower than both parents
PC-5p-120225_25	ATAGCAACCTAGAACCGGATGGGA	The expression level of H21 significantly lower than both parents
PC-5p-95888_39	CTGTTTCACCTAGGACGAGGA	The expression level of H21 significantly lower than both parents
osa-MIR2118i-p5	TAAGGGCAATCGGAATGGGAA	The expression level of H21 was between T44 and T45
osa-MIR5798-p3	CGAAAGTTGCTAGGCACCCTG	The expression level of H21 was between T44 and T45
osa-MIR5798-p5	TTTGGCAAGCTTGAGCGTTGT	The expression level of H21 was between T44 and T45

Table S5. Overview of transcriptome quality reads in this study.

Sample	Raw Data	Valid Data	Valid Ratio(reads)	Q20%	Q30%	GC content%	Mapped reads
T44_1	38725666	38314806	98.94	99.54	97.20	51.50	36388748(94.97%)
T44_2	48213060	47306468	98.12	99.27	96.83	52.50	44826286(94.76%)
T44_3	49168580	48545090	98.73	99.50	97.71	51.50	45775482(94.29%)
T45_1	42926752	42419942	98.82	99.53	97.17	51.50	40026205(94.36%)
T45_2	45058012	44560802	98.90	99.49	96.90	51.50	41972516(94.19%)
T45_3	53856466	52671860	97.80	99.19	96.29	51.50	49074669(93.17%)
H21_1	55121288	53989736	97.95	99.10	96.80	52.50	51237769(94.90%)
H21_2	54334784	53268300	98.04	99.20	96.76	52.50	50582286(94.96%)
H21_3	40272088	39531058	98.16	98.87	96.32	52.50	37335898(94.45%)

Table S6. The differentially expressed genes (DEG) uniquely belong to H21 compared to T44 and T45.

Gene id	Gene name	Gene annotation	Classification
LOC_Os02g49790	LOC_Os02g49790	aluminum-activated malate transporter, putative, expressed	DEG uniquely in H21 compared to T44
LOC_Os05g43390	LOC_Os05g43390	signal recognition particle 54 kDa protein, putative, expressed	DEG uniquely in H21 compared to T44
MSTRG.1005	LOC_Os01g16240	OsCam1-3 - Calmodulin, expressed	DEG uniquely in H21 compared to T44
MSTRG.10118	LOC_Os02g07410	glycine cleavage system H protein, putative, expressed	DEG uniquely in H21 compared to T44
MSTRG.1020	LOC_Os01g16490		DEG uniquely in H21 compared to T44
MSTRG.10394	LOC_Os02g12380	histone deacetylase, putative, expressed	DEG uniquely in H21 compared to T44
MSTRG.10425			DEG uniquely in H21 compared to T44
MSTRG.10438	LOC_Os02g12840		DEG uniquely in H21 compared to T44
MSTRG.10532	LOC_Os02g14520		DEG uniquely in H21 compared to T44
MSTRG.10541	LOC_Os02g14820		DEG uniquely in H21 compared to T44
MSTRG.10635	LOC_Os02g17340		DEG uniquely in H21 compared to T44
MSTRG.10653			DEG uniquely in H21 compared to T44
MSTRG.10669	LOC_Os02g18150	expressed protein	DEG uniquely in H21 compared to T44
MSTRG.1075			DEG uniquely in H21 compared to T44
MSTRG.1076			DEG uniquely in H21 compared to T44
MSTRG.10774	LOC_Os02g20250		DEG uniquely in H21 compared to T44
MSTRG.10791	LOC_Os02g20500		DEG uniquely in H21 compared to T44
MSTRG.10820	LOC_Os02g21220	expressed protein	DEG uniquely in H21 compared to T44
MSTRG.10826	LOC_Os02g21260	OsFBL8 - F-box domain and LRR containing protein, expressed	DEG uniquely in H21 compared to T44
MSTRG.10870			DEG uniquely in H21 compared to T44
MSTRG.10897	LOC_Os02g23826	expressed protein	DEG uniquely in H21 compared to T44
MSTRG.11013	LOC_Os02g26800		DEG uniquely in H21 compared to T44
MSTRG.11015	LOC_Os02g26850	rRNA-processing protein FCF, putative, expressed	DEG uniquely in H21 compared to T44
MSTRG.11027	LOC_Os02g27000	ATP-binding region, ATPase-like domain containing protein, expressed	DEG uniquely in H21 compared to T44
MSTRG.11038	LOC_Os02g27360	aspartic proteinase-like protein 2 precursor, putative, expressed	DEG uniquely in H21 compared to T44
MSTRG.11079	LOC_Os02g28220	transferase family protein, putative, expressed	DEG uniquely in H21 compared to T44
MSTRG.11157	LOC_Os02g30080	cytochrome P450, putative, expressed	DEG uniquely in H21 compared to T44
MSTRG.11368	LOC_Os02g34470		DEG uniquely in H21 compared to T44
MSTRG.11682	LOC_Os02g39360	B-box zinc finger family protein, putative, expressed	DEG uniquely in H21 compared to T44
MSTRG.12074	LOC_Os02g46030	MYB family transcription factor, putative, expressed	DEG uniquely in H21 compared to T44
MSTRG.12133	LOC_Os02g46850;LOC_Os02g46860	oligopeptide transporter, putative, expressed;oligopeptide transporter 4, putative, expressed	DEG uniquely in H21 compared to T44
MSTRG.12509	LOC_Os02g52410	expressed protein	DEG uniquely in H21 compared to T44
MSTRG.12564	LOC_Os02g53180	1-aminocyclopropane-1-carboxylate oxidase protein, putative, expressed	DEG uniquely in H21 compared to T44
MSTRG.12606	LOC_Os02g53835	expressed protein	DEG uniquely in H21 compared to T44
MSTRG.12701	LOC_Os02g55280		DEG uniquely in H21 compared to T44
MSTRG.12752	LOC_Os02g56014	40S ribosomal protein S30, putative, expressed	DEG uniquely in H21 compared to T44
MSTRG.12855	LOC_Os02g57250	OsIAA10 - Auxin-responsive Aux/IAA gene family member, expressed	DEG uniquely in H21 compared to T44
MSTRG.12902	LOC_Os02g57760	O-methyltransferase, putative, expressed	DEG uniquely in H21 compared to T44
MSTRG.12983	LOC_Os02g58740		DEG uniquely in H21 compared to T44
MSTRG.13114	LOC_Os03g02670	transporter family protein, putative, expressed	DEG uniquely in H21 compared to T44
MSTRG.1325	LOC_Os01g24210	retrotransposon protein, putative, Ty1-copia subclass, expressed	DEG uniquely in H21 compared to T44
MSTRG.1349	LOC_Os01g25230	expressed protein	DEG uniquely in H21 compared to T44
MSTRG.13508	LOC_Os03g08350	ubiquitin carboxyl-terminal hydrolase, family 1, putative, expressed	DEG uniquely in H21 compared to T44
MSTRG.13530	LOC_Os03g08600	glycosyl transferase, putative, expressed	DEG uniquely in H21 compared to T44
MSTRG.13662	LOC_Os03g10478	glycosyl hydrolase family 10 protein, putative, expressed	DEG uniquely in H21 compared to T44
MSTRG.13781			DEG uniquely in H21 compared to T44
MSTRG.13904	LOC_Os03g13690	expressed protein	DEG uniquely in H21 compared to T44
MSTRG.13987	LOC_Os03g14790	piwi domain containing protein, putative, expressed	DEG uniquely in H21 compared to T44
MSTRG.13991	LOC_Os03g14920		DEG uniquely in H21 compared to T44
MSTRG.13993	LOC_Os03g14980		DEG uniquely in H21 compared to T44
MSTRG.14012	LOC_Os03g15260	expressed protein	DEG uniquely in H21 compared to T44
MSTRG.14026	LOC_Os03g15460	expressed protein	DEG uniquely in H21 compared to T44
MSTRG.14114			DEG uniquely in H21 compared to T44
MSTRG.14132	LOC_Os03g16920	DnaK family protein, putative, expressed	DEG uniquely in H21 compared to T44
MSTRG.14145	LOC_Os03g17110	transposon protein, putative, CACTA, En/Spm sub-class, expressed	DEG uniquely in H21 compared to T44
MSTRG.14279	LOC_Os03g18910	COBRA-like protein 7 precursor, putative, expressed	DEG uniquely in H21 compared to T44
MSTRG.14319	LOC_Os03g19452	SMP-30/Gluconolaconase/LRE domain protein, putative, expressed	DEG uniquely in H21 compared to T44
MSTRG.14357	LOC_Os03g19920	expressed protein	DEG uniquely in H21 compared to T44
MSTRG.14363			DEG uniquely in H21 compared to T44
MSTRG.14418	LOC_Os03g21000	thioredoxin, putative, expressed	DEG uniquely in H21 compared to T44
MSTRG.14461	LOC_Os03g21700	DNA-binding protein, putative, expressed	DEG uniquely in H21 compared to T44
MSTRG.14530	LOC_Os03g22670	triacylglycerol Lipase, putative, expressed	DEG uniquely in H21 compared to T44
MSTRG.14653	LOC_Os03g25420		DEG uniquely in H21 compared to T44
MSTRG.1472			DEG uniquely in H21 compared to T44
MSTRG.14781	LOC_Os03g28250		DEG uniquely in H21 compared to T44

MSTRG.14826	LOC_Os03g29864	retrotransposon protein, putative, unclassified, expressed	DEG uniquely in H21 compared to T44
MSTRG.1487	LOC_Os01g29804	expressed protein	DEG uniquely in H21 compared to T44
MSTRG.15114	LOC_Os03g39655	expressed protein	DEG uniquely in H21 compared to T44
MSTRG.15115	LOC_Os03g39660	expressed protein	DEG uniquely in H21 compared to T44
MSTRG.15199	LOC_Os03g41438		DEG uniquely in H21 compared to T44
MSTRG.15214	LOC_Os03g42070	cyclin, putative, expressed	DEG uniquely in H21 compared to T44
MSTRG.15340	LOC_Os03g44890	anthranilate phosphoribosyltransferase, putative, expressed	DEG uniquely in H21 compared to T44
MSTRG.1539	LOC_Os01g32630		DEG uniquely in H21 compared to T44
MSTRG.15400	LOC_Os03g46140		DEG uniquely in H21 compared to T44
MSTRG.15505	LOC_Os03g47896	ribosome inactivating protein, putative, expressed	DEG uniquely in H21 compared to T44
MSTRG.15553			DEG uniquely in H21 compared to T44
MSTRG.1591	LOC_Os01g33684	disease resistance RPP13-like protein 1, putative, expressed	DEG uniquely in H21 compared to T44
MSTRG.16020			DEG uniquely in H21 compared to T44
MSTRG.16023	LOC_Os03g56200		DEG uniquely in H21 compared to T44
MSTRG.16372	LOC_Os03g60830		DEG uniquely in H21 compared to T44
MSTRG.16856	LOC_Os04g09560	tesmin/TSO1-like CXC domain containing protein, expressed	DEG uniquely in H21 compared to T44
MSTRG.16895	LOC_Os04g11210		DEG uniquely in H21 compared to T44
MSTRG.16927	LOC_Os04g12580		DEG uniquely in H21 compared to T44
MSTRG.16978	LOC_Os04g14150	dehydration response related protein, putative, expressed	DEG uniquely in H21 compared to T44
MSTRG.16996	LOC_Os04g14710		DEG uniquely in H21 compared to T44
MSTRG.17171			DEG uniquely in H21 compared to T44
MSTRG.17191	LOC_Os04g20330;LOC_Os04g20360;LOC_Os04g20400	UDP-glucoronosyl/UDP-glucosyl transferase, putative, expressed;cytokinin-O-glucosyltransferase 1, putative, expressed	DEG uniquely in H21 compared to T44
MSTRG.17613	LOC_Os04g32190	expressed protein	DEG uniquely in H21 compared to T44
MSTRG.17650	LOC_Os04g32770;LOC_Os04g32760	retrotransposon protein, putative, unclassified, expressed	DEG uniquely in H21 compared to T44
MSTRG.17830	LOC_Os04g35280	neutral/alkaline invertase, putative, expressed	DEG uniquely in H21 compared to T44
MSTRG.17847	LOC_Os04g35540	amino acid permease family protein, putative, expressed	DEG uniquely in H21 compared to T44
MSTRG.1789			DEG uniquely in H21 compared to T44
MSTRG.17900	LOC_Os04g36750	hsp20/alpha crystallin family protein, putative, expressed	DEG uniquely in H21 compared to T44
MSTRG.17906	LOC_Os04g36800	3-oxoacyl-synthase, putative, expressed	DEG uniquely in H21 compared to T44
MSTRG.18216	LOC_Os04g41570	ethylene-responsive protein related, putative, expressed	DEG uniquely in H21 compared to T44
MSTRG.18433	LOC_Os04g45070	remorin, putative, expressed	DEG uniquely in H21 compared to T44
MSTRG.18637	LOC_Os04g48370	RNA polymerase IV subunit, putative, expressed	DEG uniquely in H21 compared to T44
MSTRG.18963	LOC_Os04g53350	expressed protein	DEG uniquely in H21 compared to T44
MSTRG.190	LOC_Os01g04380	hsp20/alpha crystallin family protein, putative, expressed	DEG uniquely in H21 compared to T44
MSTRG.19031	LOC_Os04g54240	wound induced protein, putative, expressed	DEG uniquely in H21 compared to T44
MSTRG.19054	LOC_Os04g54610		DEG uniquely in H21 compared to T44
MSTRG.1910	LOC_Os01g41260	OsFBD2 - F-box and FBD domain containing protein, expressed	DEG uniquely in H21 compared to T44
MSTRG.19189	LOC_Os04g56680	OsSAUR22 - Auxin-responsive SAUR gene family member, expressed	DEG uniquely in H21 compared to T44
MSTRG.19538	LOC_Os05g02760	expressed protein	DEG uniquely in H21 compared to T44
MSTRG.19597	LOC_Os05g03640	flavonol synthase/flavanone 3-hydroxylase, putative, expressed	DEG uniquely in H21 compared to T44
MSTRG.19642	LOC_Os05g04660	thioesterase family protein, putative, expressed	DEG uniquely in H21 compared to T44
MSTRG.19849	LOC_Os05g07740	receptor-like protein kinase 2 precursor, putative, expressed	DEG uniquely in H21 compared to T44
MSTRG.1990	LOC_Os01g42400	STE_MEK_ste7_MAP2K.3 - STE kinases include homologs to sterile 7, sterile 11 and sterile 20 from yeast, expressed	DEG uniquely in H21 compared to T44
MSTRG.20095	LOC_Os05g14070	expressed protein	DEG uniquely in H21 compared to T44
MSTRG.20178			DEG uniquely in H21 compared to T44
MSTRG.20528	LOC_Os05g28290	ranBP1 domain containing protein, expressed	DEG uniquely in H21 compared to T44
MSTRG.21184	LOC_Os05g40540;LOC_Os05g40550	expressed protein	DEG uniquely in H21 compared to T44
MSTRG.21210	LOC_Os05g41090;LOC_Os05g41100	CAMK_CAMK_like_CG18020d.1 - CAMK includes calcium/calmodulin depedent protein kinases, expressed;protein kri1, putative, expressed	DEG uniquely in H21 compared to T44
MSTRG.21382	LOC_Os05g43920	auxin response factor 14, putative, expressed	DEG uniquely in H21 compared to T44
MSTRG.21531	LOC_Os05g46350	IQ calmodulin-binding motif domain containing protein, expressed	DEG uniquely in H21 compared to T44
MSTRG.21617	LOC_Os05g47960	expressed protein	DEG uniquely in H21 compared to T44
MSTRG.21720	LOC_Os05g49290	3-ketoacyl-CoA synthase, putative, expressed	DEG uniquely in H21 compared to T44
MSTRG.21766	LOC_Os05g50080	zinc finger C-x8-C-x5-C-x3-H type family protein, expressed	DEG uniquely in H21 compared to T44
MSTRG.22325	LOC_Os06g07430	OsFBX189 - F-box domain containing protein, expressed	DEG uniquely in H21 compared to T44
MSTRG.22491	LOC_Os06g09960	expressed protein	DEG uniquely in H21 compared to T44
MSTRG.22494	LOC_Os06g10020	expressed protein	DEG uniquely in H21 compared to T44
MSTRG.22496	LOC_Os06g10100		DEG uniquely in H21 compared to T44
MSTRG.22512	LOC_Os06g10355	expressed protein	DEG uniquely in H21 compared to T44
MSTRG.22592	LOC_Os06g11812	wound/stress protein, putative, expressed	DEG uniquely in H21 compared to T44

MSTRG.22671	LOC_Os06g13030	OsLIM - LIM domain protein, putative actin-binding protein and transcription factor, expressed	DEG uniquely in H21 compared to T44
MSTRG.22689			DEG uniquely in H21 compared to T44
MSTRG.22755	LOC_Os06g14620	ribonucleoside-diphosphate reductase small chain, putative, expressed	DEG uniquely in H21 compared to T44
MSTRG.22766			DEG uniquely in H21 compared to T44
MSTRG.22836			DEG uniquely in H21 compared to T44
MSTRG.22858	LOC_Os06g17130	expressed protein	DEG uniquely in H21 compared to T44
MSTRG.22880			DEG uniquely in H21 compared to T44
MSTRG.22881			DEG uniquely in H21 compared to T44
MSTRG.22886	LOC_Os06g17950	NBS-LRR disease resistance protein, putative, expressed	DEG uniquely in H21 compared to T44
MSTRG.23016	LOC_Os06g21750	expressed protein	DEG uniquely in H21 compared to T44
MSTRG.23019	LOC_Os06g21730	retrotransposon protein, putative, Ty3-gypsy subclass, expressed	DEG uniquely in H21 compared to T44
MSTRG.233	LOC_Os01g03340;LOC_Os01g03360	BBTI4 - Bowman-Birk type bran trypsin inhibitor precursor, expressed;BBTI5 - Bowman-Birk type bran trypsin inhibitor precursor, expressed	DEG uniquely in H21 compared to T44
MSTRG.23433	LOC_Os06g34810		DEG uniquely in H21 compared to T44
MSTRG.23502	LOC_Os06g36650		DEG uniquely in H21 compared to T44
MSTRG.23503	LOC_Os06g36660;LOC_Os06g36650	retrotransposon protein, putative, unclassified	DEG uniquely in H21 compared to T44
MSTRG.23528	LOC_Os06g37010	metal cation transporter, putative, expressed	DEG uniquely in H21 compared to T44
MSTRG.2389	LOC_Os01g49370	expressed protein	DEG uniquely in H21 compared to T44
MSTRG.24049	LOC_Os06g45020		DEG uniquely in H21 compared to T44
MSTRG.2417	LOC_Os01g49830	B3 DNA binding domain containing protein, expressed	DEG uniquely in H21 compared to T44
MSTRG.24177	LOC_Os06g46900	phosphosulfolactate synthase-related protein, putative, expressed	DEG uniquely in H21 compared to T44
MSTRG.24454	LOC_Os06g50706	expressed protein	DEG uniquely in H21 compared to T44
MSTRG.24456	LOC_Os06g50742	expressed protein	DEG uniquely in H21 compared to T44
MSTRG.2447	LOC_Os01g50290		DEG uniquely in H21 compared to T44
MSTRG.24601	LOC_Os07g03030		DEG uniquely in H21 compared to T44
MSTRG.24655	LOC_Os07g04270		DEG uniquely in H21 compared to T44
MSTRG.24662	LOC_Os07g04370		DEG uniquely in H21 compared to T44
MSTRG.24664	LOC_Os07g04480;LOC_Os07g04490	transposon protein, putative, unclassified, expressed;expressed protein	DEG uniquely in H21 compared to T44
MSTRG.24853			DEG uniquely in H21 compared to T44
MSTRG.24924	LOC_Os07g08540		DEG uniquely in H21 compared to T44
MSTRG.25002	LOC_Os07g09870	OsFBA2 - F-box and FBA domain containing protein, expressed	DEG uniquely in H21 compared to T44
MSTRG.25004			DEG uniquely in H21 compared to T44
MSTRG.25539	LOC_Os07g27330	no apical meristem protein, expressed	DEG uniquely in H21 compared to T44
MSTRG.25674	LOC_Os07g30800		DEG uniquely in H21 compared to T44
MSTRG.25690	LOC_Os07g31200		DEG uniquely in H21 compared to T44
MSTRG.25719	LOC_Os07g31690		DEG uniquely in H21 compared to T44
MSTRG.25826	LOC_Os07g33690	NBS-LRR type disease resistance protein Hom-F, putative, expressed	DEG uniquely in H21 compared to T44
MSTRG.25835	LOC_Os07g33790	glutamate receptor 3.4 precursor, putative, expressed	DEG uniquely in H21 compared to T44
MSTRG.25918	LOC_Os07g35920;LOC_Os07g35940	expressed protein	DEG uniquely in H21 compared to T44
MSTRG.25944	LOC_Os07g36300		DEG uniquely in H21 compared to T44
MSTRG.25954	LOC_Os07g36430		DEG uniquely in H21 compared to T44
MSTRG.26074	LOC_Os07g38290	plastocyanin-like domain containing protein, putative, expressed	DEG uniquely in H21 compared to T44
MSTRG.26313	LOC_Os07g42260	peptidase, T1 family, putative, expressed	DEG uniquely in H21 compared to T44
MSTRG.26427	LOC_Os07g44140	cytochrome P450 72A1, putative, expressed	DEG uniquely in H21 compared to T44
MSTRG.26476	LOC_Os07g45010		DEG uniquely in H21 compared to T44
MSTRG.26523	LOC_Os07g45550	expressed protein	DEG uniquely in H21 compared to T44
MSTRG.26861	LOC_Os08g02020	transferase family protein, putative, expressed	DEG uniquely in H21 compared to T44
MSTRG.26919	LOC_Os08g02860		DEG uniquely in H21 compared to T44
MSTRG.26988	LOC_Os08g04140	X8 domain containing protein, expressed	DEG uniquely in H21 compared to T44
MSTRG.27096	LOC_Os08g06100	O-methyltransferase, putative, expressed	DEG uniquely in H21 compared to T44
MSTRG.27228	LOC_Os08g08680		DEG uniquely in H21 compared to T44
MSTRG.27297	LOC_Os08g10320	SHR5-receptor-like kinase, putative, expressed	DEG uniquely in H21 compared to T44
MSTRG.27350			DEG uniquely in H21 compared to T44
MSTRG.27397			DEG uniquely in H21 compared to T44
MSTRG.27478	LOC_Os08g13460		DEG uniquely in H21 compared to T44
MSTRG.27489	LOC_Os08g13699	expressed protein	DEG uniquely in H21 compared to T44
MSTRG.2780	LOC_Os01g55190	expressed protein	DEG uniquely in H21 compared to T44
MSTRG.2794	LOC_Os01g55370	expressed protein	DEG uniquely in H21 compared to T44
MSTRG.28499	LOC_Os08g38400	oligopeptide transporter, putative, expressed	DEG uniquely in H21 compared to T44
MSTRG.28516	LOC_Os08g38800	retrotransposon protein, putative, unclassified, expressed	DEG uniquely in H21 compared to T44
MSTRG.28658	LOC_Os08g40910	expressed protein	DEG uniquely in H21 compared to T44
MSTRG.28722	LOC_Os08g41860		DEG uniquely in H21 compared to T44
MSTRG.28799	LOC_Os08g42960	ankyrin repeat-containing protein, putative, expressed	DEG uniquely in H21 compared to T44
MSTRG.28958			DEG uniquely in H21 compared to T44
MSTRG.29008	LOC_Os09g01660		DEG uniquely in H21 compared to T44
MSTRG.2917	LOC_Os01g57050	expressed protein	DEG uniquely in H21 compared to T44
MSTRG.29206	LOC_Os09g08370		DEG uniquely in H21 compared to T44
MSTRG.29533	LOC_Os09g19710	hypersensitive-induced response protein, putative, expressed	DEG uniquely in H21 compared to T44

MSTRG.29600	LOC_Os09g21120	armadillo/beta-catenin repeat family protein, putative, expressed	DEG uniquely in H21 compared to T44
MSTRG.29643			DEG uniquely in H21 compared to T44
MSTRG.29699	LOC_Os09g23620	MYB family transcription factor, putative, expressed	DEG uniquely in H21 compared to T44
MSTRG.29887	LOC_Os09g27550	expressed protein	DEG uniquely in H21 compared to T44
MSTRG.29915	LOC_Os09g27950	galactosyltransferase, putative, expressed	DEG uniquely in H21 compared to T44
MSTRG.30158	LOC_Os09g32470	membrane protein, putative, expressed	DEG uniquely in H21 compared to T44
MSTRG.30511	LOC_Os09g38490	expressed protein	DEG uniquely in H21 compared to T44
MSTRG.31	LOC_Os01g01410	TKL_IRAK_C-LEC.1 - TKL_IRAK_C-LEC has homology to C-type lectin receptors, expressed	DEG uniquely in H21 compared to T44
MSTRG.3133	LOC_Os01g59950		DEG uniquely in H21 compared to T44
MSTRG.331	LOC_Os01g05380	expressed protein	DEG uniquely in H21 compared to T44
MSTRG.3360	LOC_Os01g63854	transmembrane amino acid transporter protein, putative, expressed	DEG uniquely in H21 compared to T44
MSTRG.3364	LOC_Os01g63930	cytochrome P450, putative, expressed	DEG uniquely in H21 compared to T44
MSTRG.3421	LOC_Os01g64790	AP2 domain containing protein, expressed	DEG uniquely in H21 compared to T44
MSTRG.3691	LOC_Os01g68680	tetratricopeptide repeat domain containing protein, expressed	DEG uniquely in H21 compared to T44
MSTRG.3775	LOC_Os01g70250	heat shock protein DnaJ, putative, expressed	DEG uniquely in H21 compared to T44
MSTRG.3920	LOC_Os01g72520	phosphoesterase family protein, putative, expressed	DEG uniquely in H21 compared to T44
MSTRG.3949			DEG uniquely in H21 compared to T44
MSTRG.4235	LOC_Os10g05970	POEI12 - Pollen Ole e I allergen and extensin family protein precursor, expressed	DEG uniquely in H21 compared to T44
MSTRG.4254	LOC_Os10g06720	aldose 1-epimerase, putative, expressed	DEG uniquely in H21 compared to T44
MSTRG.4296			DEG uniquely in H21 compared to T44
MSTRG.4399	LOC_Os10g12130	retrotransposon protein, putative, Ty3-gypsy subclass, expressed	DEG uniquely in H21 compared to T44
MSTRG.452	LOC_Os01g07560	receptor-like protein kinase 2 precursor, putative, expressed	DEG uniquely in H21 compared to T44
MSTRG.464	LOC_Os01g07660	expressed protein	DEG uniquely in H21 compared to T44
MSTRG.4740	LOC_Os10g25990	retrotransposon protein, putative, unclassified, expressed	DEG uniquely in H21 compared to T44
MSTRG.475	LOC_Os01g07850	glyoxalase family protein, putative, expressed	DEG uniquely in H21 compared to T44
MSTRG.4938	LOC_Os10g30910	transmembrane protein-related, putative, expressed	DEG uniquely in H21 compared to T44
MSTRG.5080	LOC_Os10g33990	DUF584 domain containing protein, putative, expressed	DEG uniquely in H21 compared to T44
MSTRG.5105	LOC_Os10g34480	cytochrome P450, putative, expressed	DEG uniquely in H21 compared to T44
MSTRG.5134	LOC_Os10g34970	expressed protein	DEG uniquely in H21 compared to T44
MSTRG.5196	LOC_Os10g35604		DEG uniquely in H21 compared to T44
MSTRG.5246	LOC_Os10g36340	expressed protein	DEG uniquely in H21 compared to T44
MSTRG.5331	LOC_Os10g38060	phospholipase D, putative, expressed	DEG uniquely in H21 compared to T44
MSTRG.577	LOC_Os01g09410		DEG uniquely in H21 compared to T44
MSTRG.5801			DEG uniquely in H21 compared to T44
MSTRG.6078			DEG uniquely in H21 compared to T44
MSTRG.6101	LOC_Os11g07830	dirigent, putative, expressed	DEG uniquely in H21 compared to T44
MSTRG.6121	LOC_Os11g08100	eukaryotic aspartyl protease domain containing protein, expressed	DEG uniquely in H21 compared to T44
MSTRG.6263	LOC_Os11g10920	carboxyl-terminal proteinase, putative, expressed	DEG uniquely in H21 compared to T44
MSTRG.6494	LOC_Os11g17014		DEG uniquely in H21 compared to T44
MSTRG.6496	LOC_Os11g17070	expressed protein	DEG uniquely in H21 compared to T44
MSTRG.6591	LOC_Os11g22150	retrotransposon protein, putative, unclassified, expressed	DEG uniquely in H21 compared to T44
MSTRG.6702			DEG uniquely in H21 compared to T44
MSTRG.6777	LOC_Os11g30560	dehydrogenase/reductase, putative, expressed	DEG uniquely in H21 compared to T44
MSTRG.6848	LOC_Os11g32650	chalcone synthase, putative, expressed	DEG uniquely in H21 compared to T44
MSTRG.6863	LOC_Os11g33030	retrotransposon protein, putative, unclassified, expressed	DEG uniquely in H21 compared to T44
MSTRG.6979	LOC_Os11g36030	protein binding protein, putative, expressed	DEG uniquely in H21 compared to T44
MSTRG.7046	LOC_Os11g37390	OsFBDUF54 - F-box and DUF domain containing protein, expressed	DEG uniquely in H21 compared to T44
MSTRG.707			DEG uniquely in H21 compared to T44
MSTRG.7216			DEG uniquely in H21 compared to T44
MSTRG.7261			DEG uniquely in H21 compared to T44
MSTRG.7356	LOC_Os11g42480		DEG uniquely in H21 compared to T44
MSTRG.744	LOC_Os01g12120	expressed protein	DEG uniquely in H21 compared to T44
MSTRG.7533	LOC_Os11g47330	ATP-grasp domain containing protein, expressed	DEG uniquely in H21 compared to T44
MSTRG.7572	LOC_Os11g47840	OsRhmbd18 - Putative Rhomboid homologue, expressed	DEG uniquely in H21 compared to T44
MSTRG.7695	LOC_Os12g02589	hydrolase, alpha/beta fold family protein, putative, expressed	DEG uniquely in H21 compared to T44
MSTRG.7709	LOC_Os12g02790		DEG uniquely in H21 compared to T44
MSTRG.7747	LOC_Os12g03390	OsFBX435 - F-box domain containing protein, expressed	DEG uniquely in H21 compared to T44
MSTRG.782	LOC_Os01g12560	3-methyl-2-oxobutanoate hydroxymethyltransferase, putative, expressed	DEG uniquely in H21 compared to T44
MSTRG.7826	LOC_Os12g04520	FAD-binding and arabino-lactone oxidase domains containing protein, putative, expressed	DEG uniquely in H21 compared to T44
MSTRG.793	LOC_Os01g12810	retrotransposon protein, putative, unclassified, expressed	DEG uniquely in H21 compared to T44
MSTRG.7939	LOC_Os12g06550	expressed protein	DEG uniquely in H21 compared to T44
MSTRG.7987	LOC_Os12g07410		DEG uniquely in H21 compared to T44
MSTRG.8536	LOC_Os12g22839	expressed protein	DEG uniquely in H21 compared to T44

MSTRG.8601	LOC_Os12g24240	expressed protein	DEG uniquely in H21 compared to T44
MSTRG.8682	LOC_Os12g26530	expressed protein	DEG uniquely in H21 compared to T44
MSTRG.8701	LOC_Os12g27335	expressed protein	DEG uniquely in H21 compared to T44
MSTRG.8741	LOC_Os12g29560;LOC_Os12g29570	DHHC zinc finger domain containing protein, expressed;expressed protein	DEG uniquely in H21 compared to T44
MSTRG.8756	LOC_Os12g29960	yippee zinc-binding protein, putative, expressed	DEG uniquely in H21 compared to T44
MSTRG.8831			DEG uniquely in H21 compared to T44
MSTRG.9064	LOC_Os12g36060	expressed protein	DEG uniquely in H21 compared to T44
MSTRG.9208	LOC_Os12g38910	expressed protein	DEG uniquely in H21 compared to T44
MSTRG.9218	LOC_Os12g39110	zinc finger, C3HC4 type domain containing protein, expressed	DEG uniquely in H21 compared to T44
MSTRG.9535	LOC_Os12g43580	expressed protein	DEG uniquely in H21 compared to T44
MSTRG.9658	LOC_Os02g02020	pentatricopeptide, putative, expressed	DEG uniquely in H21 compared to T44
MSTRG.9850	LOC_Os02g04380	expressed protein	DEG uniquely in H21 compared to T44
MSTRG.9865	LOC_Os02g04540	retrotransposon protein, putative, unclassified, expressed	DEG uniquely in H21 compared to T44
MSTRG.9927	LOC_Os02g04960	expressed protein	DEG uniquely in H21 compared to T44
MSTRG.9972			DEG uniquely in H21 compared to T44
LOC_Os08g30340	LOC_Os08g30340	PAS2, putative, expressed	DEG uniquely in H21 compared to T45
LOC_Os11g02650	LOC_Os11g02650	fringe-related protein, putative, expressed	DEG uniquely in H21 compared to T45
LOC_Os11g02660	LOC_Os11g02660	hydrolase, alpha/beta fold family domain containing protein, expressed	DEG uniquely in H21 compared to T45
MSTRG.10001	LOC_Os02g05980		DEG uniquely in H21 compared to T45
MSTRG.10034	LOC_Os02g06300	GTP-binding protein lepA, putative, expressed	DEG uniquely in H21 compared to T45
MSTRG.10048	LOC_Os02g06500	DSHCT domain containing protein, expressed	DEG uniquely in H21 compared to T45
MSTRG.10072	LOC_Os02g06830	expressed protein	DEG uniquely in H21 compared to T45
MSTRG.10100	LOC_Os02g07180	expressed protein	DEG uniquely in H21 compared to T45
MSTRG.10110	LOC_Os02g07310	argonaute, putative, expressed	DEG uniquely in H21 compared to T45
MSTRG.1012	LOC_Os01g16340	expressed protein	DEG uniquely in H21 compared to T45
MSTRG.10125	LOC_Os02g07630		DEG uniquely in H21 compared to T45
MSTRG.10131	LOC_Os02g07730	haloacid dehalogenase-like hydrolase domain-containing protein 3, putative, expressed	DEG uniquely in H21 compared to T45
MSTRG.10153	LOC_Os02g07980		DEG uniquely in H21 compared to T45
MSTRG.10158	LOC_Os02g08080	expressed protein	DEG uniquely in H21 compared to T45
MSTRG.10180	LOC_Os02g08330	gp176, putative, expressed	DEG uniquely in H21 compared to T45
MSTRG.10205	LOC_Os02g09130	palmitoyltransferase TIP1, putative, expressed	DEG uniquely in H21 compared to T45
MSTRG.10277	LOC_Os02g10310	fumarylacetoacetase, putative, expressed	DEG uniquely in H21 compared to T45
MSTRG.10283	LOC_Os02g10390	chlorophyll A-B binding protein, putative, expressed	DEG uniquely in H21 compared to T45
MSTRG.10433			DEG uniquely in H21 compared to T45
MSTRG.10443	LOC_Os02g12900	cysteine synthase, putative, expressed	DEG uniquely in H21 compared to T45
MSTRG.10469	LOC_Os02g13150	pollen-specific protein SF21, putative, expressed	DEG uniquely in H21 compared to T45
MSTRG.10488	LOC_Os02g13580	kinesin motor domain containing protein, expressed	DEG uniquely in H21 compared to T45
MSTRG.10489	LOC_Os02g13630	expressed protein	DEG uniquely in H21 compared to T45
MSTRG.10554	LOC_Os02g15160		DEG uniquely in H21 compared to T45
MSTRG.10562			DEG uniquely in H21 compared to T45
MSTRG.10591	LOC_Os02g15950		DEG uniquely in H21 compared to T45
MSTRG.10626	LOC_Os02g17240	ATROPGEF7/ROPGEF7, putative, expressed	DEG uniquely in H21 compared to T45
MSTRG.10702	LOC_Os02g18830	ribosome biogenesis protein NEP1, putative, expressed	DEG uniquely in H21 compared to T45
MSTRG.10709	LOC_Os02g18880	calcineurin B, putative, expressed	DEG uniquely in H21 compared to T45
MSTRG.1071	LOC_Os01g17279	exonuclease, putative, expressed	DEG uniquely in H21 compared to T45
MSTRG.10756	LOC_Os02g19820	nodulin MtN3 family protein, putative, expressed	DEG uniquely in H21 compared to T45
MSTRG.10764	LOC_Os02g19924	aminotransferase, classes I and II, domain containing protein, expressed	DEG uniquely in H21 compared to T45
MSTRG.10766	LOC_Os02g19990	reticulon domain containing protein, putative, expressed	DEG uniquely in H21 compared to T45
MSTRG.1077			DEG uniquely in H21 compared to T45
MSTRG.10805	LOC_Os02g20970		DEG uniquely in H21 compared to T45
MSTRG.10823	LOC_Os02g21240	OsFBX48 - F-box domain containing protein, expressed	DEG uniquely in H21 compared to T45
MSTRG.10959			DEG uniquely in H21 compared to T45
MSTRG.111	LOC_Os01g02860	expressed protein	DEG uniquely in H21 compared to T45
MSTRG.1116	LOC_Os01g18670	MDR-like ABC transporter, putative, expressed	DEG uniquely in H21 compared to T45
MSTRG.11228	LOC_Os02g31830	expressed protein	DEG uniquely in H21 compared to T45
MSTRG.11229	LOC_Os02g31890		DEG uniquely in H21 compared to T45
MSTRG.11239	LOC_Os02g32160	copine, putative, expressed	DEG uniquely in H21 compared to T45
MSTRG.11260	LOC_Os02g32504	heparan-alpha-glucosaminide N-acetyltransferase, putative, expressed	DEG uniquely in H21 compared to T45
MSTRG.1132	LOC_Os01g19270	expressed protein	DEG uniquely in H21 compared to T45
MSTRG.11366	LOC_Os02g34460	40S ribosomal protein S10, putative, expressed	DEG uniquely in H21 compared to T45
MSTRG.11373	LOC_Os02g34540	tetratricopeptide repeat containing protein, putative, expressed	DEG uniquely in H21 compared to T45
MSTRG.11382	LOC_Os02g34650	expressed protein	DEG uniquely in H21 compared to T45
MSTRG.11395	LOC_Os02g34860	Regulator of chromosome condensation domain containing protein, expressed	DEG uniquely in H21 compared to T45
MSTRG.11408	LOC_Os02g35010	STE_MEKK_ste11_MAP3K.9 - STE kinases include homologs to sterile 7, sterile 11 and sterile 20 from yeast, expressed	DEG uniquely in H21 compared to T45
MSTRG.11411	LOC_Os02g35060	RNA methyltransferase protein, putative, expressed	DEG uniquely in H21 compared to T45
MSTRG.11417	LOC_Os02g35144	zinc finger, C3HC4 type domain containing protein, expressed	DEG uniquely in H21 compared to T45
MSTRG.11492	LOC_Os02g36600	aldose 1-epimerase, putative, expressed	DEG uniquely in H21 compared to T45

MSTRG.11509	LOC_Os02g36940	uncharacterized Cys-rich domain containing protein, putative, expressed	DEG uniquely in H21 compared to T45
MSTRG.11589	LOC_Os02g38140		DEG uniquely in H21 compared to T45
MSTRG.11602	LOC_Os02g38290	cytochrome P450, putative, expressed	DEG uniquely in H21 compared to T45
MSTRG.11664	LOC_Os02g39100	fasciated ear2, putative, expressed	DEG uniquely in H21 compared to T45
MSTRG.11680	LOC_Os02g39350	eukaryotic translation initiation factor 2A, putative, expressed	DEG uniquely in H21 compared to T45
MSTRG.11685	LOC_Os02g39390;LOC_Os02g39400	expressed protein;isochorismatase family protein, putative, expressed	DEG uniquely in H21 compared to T45
MSTRG.1169	LOC_Os01g19940	expressed protein	DEG uniquely in H21 compared to T45
MSTRG.11696	LOC_Os02g39470	cyclin, N-terminal domain containing protein, expressed	DEG uniquely in H21 compared to T45
MSTRG.11755	LOC_Os02g40530	MYB family transcription factor, putative, expressed	DEG uniquely in H21 compared to T45
MSTRG.1178	LOC_Os01g20730	expressed protein	DEG uniquely in H21 compared to T45
MSTRG.11797	LOC_Os02g41650;LOC_Os02g41680	phenylalanine ammonia-lyase, putative, expressed	DEG uniquely in H21 compared to T45
MSTRG.11798	LOC_Os02g41650		DEG uniquely in H21 compared to T45
MSTRG.11807	LOC_Os02g41820	expressed protein	DEG uniquely in H21 compared to T45
MSTRG.1181			DEG uniquely in H21 compared to T45
MSTRG.11862	LOC_Os02g42690	zinc finger, C3HC4 type domain containing protein, expressed	DEG uniquely in H21 compared to T45
MSTRG.11899	LOC_Os02g43340	hydrolase, alpha/beta fold family domain containing protein, expressed	DEG uniquely in H21 compared to T45
MSTRG.11971	LOC_Os02g44370;LOC_Os02g44360	scarecrow transcription factor family protein, putative, expressed;scarecrow, putative, expressed	DEG uniquely in H21 compared to T45
MSTRG.12000			DEG uniquely in H21 compared to T45
MSTRG.12017	LOC_Os02g45054	ZOS2-15 - C2H2 zinc finger protein, expressed	DEG uniquely in H21 compared to T45
MSTRG.12041	LOC_Os02g45530	HOTHEAD precursor, putative, expressed	DEG uniquely in H21 compared to T45
MSTRG.12054	LOC_Os02g45710	zinc finger, C3HC4 type domain containing protein, expressed	DEG uniquely in H21 compared to T45
MSTRG.12055	LOC_Os02g45690	uncharacterized mscS family protein, putative, expressed	DEG uniquely in H21 compared to T45
MSTRG.12115	LOC_Os02g46680		DEG uniquely in H21 compared to T45
MSTRG.12116	LOC_Os02g46650	ubiquitin carboxyl-terminal hydrolase domain containing protein, expressed	DEG uniquely in H21 compared to T45
MSTRG.12185	LOC_Os02g47580	expressed protein	DEG uniquely in H21 compared to T45
MSTRG.12218	LOC_Os02g48110	DnaK family protein, putative, expressed	DEG uniquely in H21 compared to T45
MSTRG.12230	LOC_Os02g48330	3-hydroxy-3-methylglutaryl-coenzyme A reductase, putative, expressed	DEG uniquely in H21 compared to T45
MSTRG.12270	LOC_Os02g48880	chloride transporter, chloride channel family, putative, expressed	DEG uniquely in H21 compared to T45
MSTRG.12298	LOC_Os02g49230	CCT/B-box zinc finger protein, putative, expressed	DEG uniquely in H21 compared to T45
MSTRG.1234	LOC_Os01g21940		DEG uniquely in H21 compared to T45
MSTRG.12390	LOC_Os02g50600	glycosyl transferase 8 domain containing protein, putative, expressed	DEG uniquely in H21 compared to T45
MSTRG.12399	LOC_Os02g50700	expressed protein	DEG uniquely in H21 compared to T45
MSTRG.12524	LOC_Os02g52490;LOC_Os02g52510	expressed protein;SNF2 family N-terminal domain containing protein, expressed	DEG uniquely in H21 compared to T45
MSTRG.12551	LOC_Os02g53070	HYS1, putative, expressed	DEG uniquely in H21 compared to T45
MSTRG.12565	LOC_Os02g53200	glucan endo-1,3-beta-glucosidase precursor, putative, expressed	DEG uniquely in H21 compared to T45
MSTRG.12622	LOC_Os02g54110	expressed protein	DEG uniquely in H21 compared to T45
MSTRG.12633	LOC_Os02g54280;LOC_Os02g54290	retrotransposon protein, putative, unclassified, expressed;BRUSHY 1, putative, expressed	DEG uniquely in H21 compared to T45
MSTRG.12665			DEG uniquely in H21 compared to T45
MSTRG.12708	LOC_Os02g55330	OsPOP6 - Putative Prolyl Oligopeptidase homologue, expressed	DEG uniquely in H21 compared to T45
MSTRG.12716	LOC_Os02g55410	MCM5 - Putative minichromosome maintenance MCM complex subunit 5, expressed	DEG uniquely in H21 compared to T45
MSTRG.12741	LOC_Os02g55920	expressed protein	DEG uniquely in H21 compared to T45
MSTRG.12817	LOC_Os02g56820		DEG uniquely in H21 compared to T45
MSTRG.12839	LOC_Os02g57080	serine/threonine-protein kinase, putative, expressed	DEG uniquely in H21 compared to T45
MSTRG.12842	LOC_Os02g57060	OsCttP2 - Putative C-terminal processing peptidase homologue, expressed	DEG uniquely in H21 compared to T45
MSTRG.12884	LOC_Os02g57530	ethylene receptor, putative, expressed	DEG uniquely in H21 compared to T45
MSTRG.1303	LOC_Os01g23870	expressed protein	DEG uniquely in H21 compared to T45
MSTRG.13037	LOC_Os03g01590	PAIR1, putative, expressed	DEG uniquely in H21 compared to T45
MSTRG.13053	LOC_Os03g01880	possible lysine decarboxylase domain containing protein, expressed	DEG uniquely in H21 compared to T45
MSTRG.13088	LOC_Os03g02320	STE_PAK_Ste20_STLK.3 - STE kinases include homologs to sterile 7, sterile 11 and sterile 20 from yeast, expressed	DEG uniquely in H21 compared to T45
MSTRG.13117	LOC_Os03g02690	dihydrodipicolinate reductase, putative, expressed	DEG uniquely in H21 compared to T45
MSTRG.13149	LOC_Os03g03070;LOC_Os03g03100	transcription factor, putative, expressed;OsMADS50 - MADS-box family gene with MIKCc type-box, expressed	DEG uniquely in H21 compared to T45
MSTRG.13171	LOC_Os03g03450	anthranilate phosphoribosyltransferase, chloroplast precursor, putative, expressed	DEG uniquely in H21 compared to T45
MSTRG.13173	LOC_Os03g03470	expressed protein	DEG uniquely in H21 compared to T45
MSTRG.13193	LOC_Os03g03724	expressed protein	DEG uniquely in H21 compared to T45
MSTRG.13223	LOC_Os03g04140		DEG uniquely in H21 compared to T45

MSTRG.13239	LOC_Os03g04400	heat shock protein DnaJ, putative, expressed	DEG uniquely in H21 compared to T45
MSTRG.13270	LOC_Os03g04890	zinc finger family protein, putative, expressed	DEG uniquely in H21 compared to T45
MSTRG.13288	LOC_Os03g05200	DENN domain containing protein, expressed	DEG uniquely in H21 compared to T45
MSTRG.13306	LOC_Os03g05390	Citrate transporter protein, putative, expressed	DEG uniquely in H21 compared to T45
MSTRG.13317	LOC_Os03g05550		DEG uniquely in H21 compared to T45
MSTRG.13354	LOC_Os03g06190	3-5 exonuclease family protein, putative, expressed	DEG uniquely in H21 compared to T45
MSTRG.1340	LOC_Os01g24880	zinc finger, C3HC4 type domain containing protein, expressed	DEG uniquely in H21 compared to T45
MSTRG.13404	LOC_Os03g06900	DNA topoisomerase 3 protein, putative, expressed	DEG uniquely in H21 compared to T45
MSTRG.13427	LOC_Os03g07200	expressed protein	DEG uniquely in H21 compared to T45
MSTRG.13436	LOC_Os03g07300	ribulose-phosphate 3-epimerase, chloroplast precursor, putative, expressed	DEG uniquely in H21 compared to T45
MSTRG.13458	LOC_Os03g07720	expressed protein	DEG uniquely in H21 compared to T45
MSTRG.13460			DEG uniquely in H21 compared to T45
MSTRG.13462	LOC_Os03g07790	zinc finger, C3HC4 type domain containing protein, expressed	DEG uniquely in H21 compared to T45
MSTRG.13495	LOC_Os03g08070	copper-transporting ATPase PAA1, putative, expressed	DEG uniquely in H21 compared to T45
MSTRG.1350	LOC_Os01g25240	expressed protein	DEG uniquely in H21 compared to T45
MSTRG.13504	LOC_Os03g08320	ZIM domain containing protein, putative, expressed	DEG uniquely in H21 compared to T45
MSTRG.13506	LOC_Os03g08290	expressed protein	DEG uniquely in H21 compared to T45
MSTRG.13521			DEG uniquely in H21 compared to T45
MSTRG.13566	LOC_Os03g08999	dehydrogenase, putative, expressed	DEG uniquely in H21 compared to T45
MSTRG.13637	LOC_Os03g10210	homeobox domain containing protein, expressed	DEG uniquely in H21 compared to T45
MSTRG.13642	LOC_Os03g10250	expressed protein	DEG uniquely in H21 compared to T45
MSTRG.13648	LOC_Os03g10310		DEG uniquely in H21 compared to T45
MSTRG.13654	LOC_Os03g10370	ADP-ribosylation factor, putative, expressed	DEG uniquely in H21 compared to T45
MSTRG.13681	LOC_Os03g10750	CUE domain containing protein, expressed	DEG uniquely in H21 compared to T45
MSTRG.13698	LOC_Os03g10920	syntaxin 6, N-terminal domain containing protein, expressed	DEG uniquely in H21 compared to T45
MSTRG.13721	LOC_Os03g11140	pleckstrin homology domain-containing protein-related taxo, putative, expressed	DEG uniquely in H21 compared to T45
MSTRG.13740	LOC_Os03g11400	targeting protein-related, putative, expressed	DEG uniquely in H21 compared to T45
MSTRG.13760	LOC_Os03g11600	YABBY domain containing protein, putative, expressed	DEG uniquely in H21 compared to T45
MSTRG.13767	LOC_Os03g11670	pentatricopeptide repeat domain containing protein, putative, expressed	DEG uniquely in H21 compared to T45
MSTRG.13789	LOC_Os03g11960	copper/zinc superoxide dismutase, putative, expressed	DEG uniquely in H21 compared to T45
MSTRG.1379	LOC_Os01g25920	expressed protein	DEG uniquely in H21 compared to T45
MSTRG.13826			DEG uniquely in H21 compared to T45
MSTRG.13840	LOC_Os03g12610	DNA polymerase I, putative, expressed	DEG uniquely in H21 compared to T45
MSTRG.13886	LOC_Os03g13370	tetratricopeptide repeat domain containing protein, expressed	DEG uniquely in H21 compared to T45
MSTRG.13891	LOC_Os03g13520	endonuclease/exonuclease/phosphatase family domain containing protein, expressed	DEG uniquely in H21 compared to T45
MSTRG.13897	LOC_Os03g13550	oxidoreductase, putative, expressed	DEG uniquely in H21 compared to T45
MSTRG.13925	LOC_Os03g13984		DEG uniquely in H21 compared to T45
MSTRG.13940	LOC_Os03g14170	3-ketoacyl-CoA synthase, putative, expressed	DEG uniquely in H21 compared to T45
MSTRG.13941	LOC_Os03g14120	dihydrodipicolinate reductase, putative, expressed	DEG uniquely in H21 compared to T45
MSTRG.13982			DEG uniquely in H21 compared to T45
MSTRG.13988	LOC_Os03g14800	serine palmitoyltransferase 1, putative, expressed	DEG uniquely in H21 compared to T45
MSTRG.14000	LOC_Os03g15020	beta-galactosidase precursor, putative, expressed	DEG uniquely in H21 compared to T45
MSTRG.14039	LOC_Os03g15600	expressed protein	DEG uniquely in H21 compared to T45
MSTRG.14047	LOC_Os03g15740	uncharacterized TPR repeat-containing protein, putative, expressed	DEG uniquely in H21 compared to T45
MSTRG.1406			DEG uniquely in H21 compared to T45
MSTRG.14104	LOC_Os03g16500	aspartic proteinase nepenthesin precursor, putative, expressed	DEG uniquely in H21 compared to T45
MSTRG.14157	LOC_Os03g17250	ankyrin repeat-containing protein, putative, expressed	DEG uniquely in H21 compared to T45
MSTRG.1417	LOC_Os01g27560		DEG uniquely in H21 compared to T45
MSTRG.14183	LOC_Os03g17660	expressed protein	DEG uniquely in H21 compared to T45
MSTRG.14206	LOC_Os03g17960	expressed protein	DEG uniquely in H21 compared to T45
MSTRG.14219	LOC_Os03g18160	mitochondrial carrier protein, putative, expressed	DEG uniquely in H21 compared to T45
MSTRG.14220	LOC_Os03g18170	STE_MEKK_ste11_MAP3K.13 - STE kinases include homologs to sterile 7, sterile 11 and sterile 20 from yeast, expressed	DEG uniquely in H21 compared to T45
MSTRG.14305	LOC_Os03g19300	peptide chain release factor protein, putative, expressed	DEG uniquely in H21 compared to T45
MSTRG.14377	LOC_Os03g20300	glucose-6-phosphate 1-dehydrogenase, chloroplast precursor, putative, expressed	DEG uniquely in H21 compared to T45
MSTRG.14400	LOC_Os03g20680	late embryogenesis abundant protein 1, putative, expressed	DEG uniquely in H21 compared to T45
MSTRG.14402	LOC_Os03g20700	magnesium-chelatase, putative, expressed	DEG uniquely in H21 compared to T45
MSTRG.14413	LOC_Os03g20870	zinc finger, C3HC4 type domain containing protein, expressed	DEG uniquely in H21 compared to T45
MSTRG.1443	LOC_Os01g28680	WD domain, G-beta repeat domain containing protein, expressed	DEG uniquely in H21 compared to T45

MSTRG.14441	LOC_Os03g21400;LOC_Os03g21419	cytochrome P450, putative, expressed;retrotransposon protein, putative, Ty1-copia subclass, expressed	DEG uniquely in H21 compared to T45
MSTRG.14480	LOC_Os03g21950	fumarate hydratase, mitochondrial precursor, putative, expressed	DEG uniquely in H21 compared to T45
MSTRG.14523	LOC_Os03g22560	MYB family transcription factor, putative, expressed	DEG uniquely in H21 compared to T45
MSTRG.14525	LOC_Os03g22580	CW-type Zinc Finger, putative, expressed	DEG uniquely in H21 compared to T45
MSTRG.14593	LOC_Os03g24130	CXXC1 - Cysteine-rich protein with CXXC and CXXXC motifs precursor, putative, expressed	DEG uniquely in H21 compared to T45
MSTRG.14612	LOC_Os03g24390		DEG uniquely in H21 compared to T45
MSTRG.14712	LOC_Os03g26460	CS domain containing protein, putative, expressed	DEG uniquely in H21 compared to T45
MSTRG.14713	LOC_Os03g26480	expressed protein	DEG uniquely in H21 compared to T45
MSTRG.14738	LOC_Os03g27230	phospho-2-dehydro-3-deoxyheptonate aldolase, chloroplast precursor, putative, expressed	DEG uniquely in H21 compared to T45
MSTRG.14786			DEG uniquely in H21 compared to T45
MSTRG.1479	LOC_Os01g29680;LOC_Os01g29690	retrotransposon protein, putative, Ty1-copia subclass, expressed	DEG uniquely in H21 compared to T45
MSTRG.14801	LOC_Os03g29190	PDI, putative, expressed	DEG uniquely in H21 compared to T45
MSTRG.14813	LOC_Os03g29584	expressed protein	DEG uniquely in H21 compared to T45
MSTRG.14896	LOC_Os03g31679	annexin A7, putative, expressed	DEG uniquely in H21 compared to T45
MSTRG.14996	LOC_Os03g37010	glycosyltransferase, putative, expressed	DEG uniquely in H21 compared to T45
MSTRG.15001	LOC_Os03g37140	expressed protein	DEG uniquely in H21 compared to T45
MSTRG.15004	LOC_Os03g37240	retrotransposon protein, putative, unclassified, expressed	DEG uniquely in H21 compared to T45
MSTRG.15037	LOC_Os03g38210	MYB family transcription factor, putative, expressed	DEG uniquely in H21 compared to T45
MSTRG.15051	LOC_Os03g38540	folic acid binding protein, putative, expressed	DEG uniquely in H21 compared to T45
MSTRG.15094	LOC_Os03g39610	chlorophyll A-B binding protein, putative, expressed	DEG uniquely in H21 compared to T45
MSTRG.15117	LOC_Os03g39820	expressed protein	DEG uniquely in H21 compared to T45
MSTRG.15132	LOC_Os03g40060		DEG uniquely in H21 compared to T45
MSTRG.15134	LOC_Os03g40070	transposon protein, putative, unclassified, expressed	DEG uniquely in H21 compared to T45
MSTRG.15178	LOC_Os03g40920	expressed protein	DEG uniquely in H21 compared to T45
MSTRG.15183	LOC_Os03g41080	seed maturation protein PM23, putative, expressed	DEG uniquely in H21 compared to T45
MSTRG.15243	LOC_Os03g42760	zinc finger protein-related, putative, expressed	DEG uniquely in H21 compared to T45
MSTRG.15274	LOC_Os03g43590	LSTK-1-like kinase, putative, expressed	DEG uniquely in H21 compared to T45
MSTRG.15281	LOC_Os03g43810		DEG uniquely in H21 compared to T45
MSTRG.15329	LOC_Os03g44670	expressed protein	DEG uniquely in H21 compared to T45
MSTRG.15346			DEG uniquely in H21 compared to T45
MSTRG.15347			DEG uniquely in H21 compared to T45
MSTRG.15465	LOC_Os03g47310	transposon protein, putative, CACTA, En/Spm sub-class, expressed	DEG uniquely in H21 compared to T45
MSTRG.15484	LOC_Os03g47650	ankyrin, putative, expressed	DEG uniquely in H21 compared to T45
MSTRG.15512	LOC_Os03g47949;LOC_Os03g47960	HECT-domain domain containing protein, expressed;expressed protein	DEG uniquely in H21 compared to T45
MSTRG.15536	LOC_Os03g48180	peptide transporter PTR2, putative, expressed	DEG uniquely in H21 compared to T45
MSTRG.1555	LOC_Os01g32830	expressed protein	DEG uniquely in H21 compared to T45
MSTRG.15569	LOC_Os03g48810	nucleobase-ascorbate transporter, putative, expressed	DEG uniquely in H21 compared to T45
MSTRG.15649	LOC_Os03g50190	expressed protein	DEG uniquely in H21 compared to T45
MSTRG.15653	LOC_Os03g50220;LOC_Os03g50230	homologous-pairing protein meu13, putative, expressed;protein kinase, putative, expressed	DEG uniquely in H21 compared to T45
MSTRG.15711	LOC_Os03g51180		DEG uniquely in H21 compared to T45
MSTRG.15751	LOC_Os03g51650	membrane protein, putative, expressed	DEG uniquely in H21 compared to T45
MSTRG.1577	LOC_Os01g33204	retrotransposon protein, putative, unclassified, expressed	DEG uniquely in H21 compared to T45
MSTRG.15779	LOC_Os03g52160	regulatory protein, putative, expressed	DEG uniquely in H21 compared to T45
MSTRG.15787	LOC_Os03g52310		DEG uniquely in H21 compared to T45
MSTRG.15792	LOC_Os03g52380	PIII5 - Proteinase inhibitor II family protein precursor, expressed	DEG uniquely in H21 compared to T45
MSTRG.15876	LOC_Os03g53660	Myosin head domain containing protein, expressed	DEG uniquely in H21 compared to T45
MSTRG.15905	LOC_Os03g54100	potassium channel protein, putative, expressed	DEG uniquely in H21 compared to T45
MSTRG.15940	LOC_Os03g55090	alpha-glucan phosphorylast isozyme, putative, expressed	DEG uniquely in H21 compared to T45
MSTRG.15964	LOC_Os03g55380	oxidoreductase, putative, expressed	DEG uniquely in H21 compared to T45
MSTRG.15981	LOC_Os03g55590	MYB family transcription factor, putative, expressed	DEG uniquely in H21 compared to T45
MSTRG.15983	LOC_Os03g55660;LOC_Os03g55670	nucleoporin, putative, expressed;expressed protein	DEG uniquely in H21 compared to T45
MSTRG.16059	LOC_Os03g56660	calmodulin binding protein, putative, expressed	DEG uniquely in H21 compared to T45
MSTRG.16070	LOC_Os03g56740	expressed protein	DEG uniquely in H21 compared to T45
MSTRG.16115	LOC_Os03g57220	hydroxyacid oxidase 1, putative, expressed	DEG uniquely in H21 compared to T45
MSTRG.16143	LOC_Os03g57660	peptidase, putative, expressed	DEG uniquely in H21 compared to T45
MSTRG.16180	LOC_Os03g58190	expressed protein	DEG uniquely in H21 compared to T45
MSTRG.16184	LOC_Os03g58250	bZIP transcription factor domain containing protein, expressed	DEG uniquely in H21 compared to T45
MSTRG.16195	LOC_Os03g58390	zinc finger, C3HC4 type domain containing protein, expressed	DEG uniquely in H21 compared to T45
MSTRG.16213	LOC_Os03g58650	expressed protein	DEG uniquely in H21 compared to T45
MSTRG.16224	LOC_Os03g58790	ATPase, putative, expressed	DEG uniquely in H21 compared to T45
MSTRG.16237	LOC_Os03g58910	expressed protein	DEG uniquely in H21 compared to T45

MSTRG.16265	LOC_Os03g59320	expressed protein	DEG uniquely in H21 compared to T45
MSTRG.16278	LOC_Os03g59580	hydrolase, NUDIX family, domain containing protein, expressed	DEG uniquely in H21 compared to T45
MSTRG.16284	LOC_Os03g59600	mitochondrial Rho GTPase 1, putative, expressed	DEG uniquely in H21 compared to T45
MSTRG.16288	LOC_Os03g59650	BRCA1 C Terminus domain containing protein, expressed	DEG uniquely in H21 compared to T45
MSTRG.16335	LOC_Os03g60370;LOC_Os03g60380	histidine acid phosphatase, putative, expressed;cinnamoyl CoA reductase, putative, expressed	DEG uniquely in H21 compared to T45
MSTRG.16389	LOC_Os03g61030	transcription termination factor nusG family protein, expressed	DEG uniquely in H21 compared to T45
MSTRG.16428	LOC_Os03g61560	expressed protein	DEG uniquely in H21 compared to T45
MSTRG.16455	LOC_Os03g61920	electron transfer flavoprotein subunit alpha, mitochondrial precursor, putative, expressed	DEG uniquely in H21 compared to T45
MSTRG.16480	LOC_Os03g62340	protein kinase family protein, putative, expressed	DEG uniquely in H21 compared to T45
MSTRG.16603	LOC_Os03g64260	AP2 domain containing protein, expressed	DEG uniquely in H21 compared to T45
MSTRG.1677	LOC_Os01g36080	protein phosphatase 2C containing protein, expressed	DEG uniquely in H21 compared to T45
MSTRG.16777	LOC_Os04g05630	expressed protein	DEG uniquely in H21 compared to T45
MSTRG.16855	LOC_Os04g09550	expressed protein	DEG uniquely in H21 compared to T45
MSTRG.1689	LOC_Os01g36390	MCM4 - Putative minichromosome maintenance MCM complex subunit 4, expressed	DEG uniquely in H21 compared to T45
MSTRG.16907	LOC_Os04g11820	white-brown complex homolog protein, putative, expressed	DEG uniquely in H21 compared to T45
MSTRG.16919	LOC_Os04g12460	Leucine Rich Repeat family protein, expressed	DEG uniquely in H21 compared to T45
MSTRG.16936	LOC_Os04g12950;LOC_Os04g12920	expressed protein;indole-3-acetate beta-glucosyltransferase, putative, expressed	DEG uniquely in H21 compared to T45
MSTRG.16949	LOC_Os04g12820	expressed protein	DEG uniquely in H21 compared to T45
MSTRG.16960			DEG uniquely in H21 compared to T45
MSTRG.16976	LOC_Os04g14140	expressed protein	DEG uniquely in H21 compared to T45
MSTRG.16994	LOC_Os04g14690;LOC_Os04g14710	flavin-containing monooxygenase family protein, putative, expressed	DEG uniquely in H21 compared to T45
MSTRG.17022	LOC_Os04g15660	receptor kinase, putative, expressed	DEG uniquely in H21 compared to T45
MSTRG.17183	LOC_Os04g20180	expressed protein	DEG uniquely in H21 compared to T45
MSTRG.17186	LOC_Os04g20210	expressed protein	DEG uniquely in H21 compared to T45
MSTRG.17261			DEG uniquely in H21 compared to T45
MSTRG.17300	LOC_Os04g24110	anthocyanin 3-O-beta-glucosyltransferase, putative, expressed	DEG uniquely in H21 compared to T45
MSTRG.17370	LOC_Os04g26330	expressed protein	DEG uniquely in H21 compared to T45
MSTRG.17381	LOC_Os04g26920	oxidoreductase, aldo/keto reductase family protein, putative, expressed	DEG uniquely in H21 compared to T45
MSTRG.17405			DEG uniquely in H21 compared to T45
MSTRG.1751	LOC_Os01g37800	ras-related protein, putative, expressed	DEG uniquely in H21 compared to T45
MSTRG.17525	LOC_Os04g30620;LOC_Os04g30720	retrotransposon protein, putative, Ty3-gypsy subclass, expressed;kinesin motor domain containing protein, putative, expressed	DEG uniquely in H21 compared to T45
MSTRG.17548	LOC_Os04g30930		DEG uniquely in H21 compared to T45
MSTRG.17561	LOC_Os04g31140	expressed protein	DEG uniquely in H21 compared to T45
MSTRG.17606	LOC_Os04g32090	expressed protein	DEG uniquely in H21 compared to T45
MSTRG.17629	LOC_Os04g32404		DEG uniquely in H21 compared to T45
MSTRG.17639	LOC_Os04g32600	expressed protein	DEG uniquely in H21 compared to T45
MSTRG.17669	LOC_Os04g33150	desiccation-related protein PCC13-62 precursor, putative, expressed	DEG uniquely in H21 compared to T45
MSTRG.17678	LOC_Os04g33260	expressed protein	DEG uniquely in H21 compared to T45
MSTRG.17733	LOC_Os04g33950	E2F family transcription factor protein, putative, expressed	DEG uniquely in H21 compared to T45
MSTRG.17751	LOC_Os04g34270		DEG uniquely in H21 compared to T45
MSTRG.17814	LOC_Os04g35060	nicotinate phosphoribosyltransferase family domain containing protein, expressed	DEG uniquely in H21 compared to T45
MSTRG.17818	LOC_Os04g35114	receptor-like kinase, putative, expressed	DEG uniquely in H21 compared to T45
MSTRG.17845	LOC_Os04g35520	OsAPx7 - Stromal Ascorbate Peroxidase encoding gene 5,8, expressed	DEG uniquely in H21 compared to T45
MSTRG.1787	LOC_Os01g38660	expressed protein	DEG uniquely in H21 compared to T45
MSTRG.17872	LOC_Os04g35880;LOC_Os04g35864	DDT domain-containing protein, putative, expressed;retrotransposon protein, putative, unclassified, expressed	DEG uniquely in H21 compared to T45
MSTRG.17973	LOC_Os04g38270	expressed protein	DEG uniquely in H21 compared to T45
MSTRG.17999	LOC_Os04g38640	OsDegp5 - Putative Deg protease homologue, expressed	DEG uniquely in H21 compared to T45
MSTRG.18010	LOC_Os04g38780		DEG uniquely in H21 compared to T45
MSTRG.18040	LOC_Os04g39060	CRS1/YhbY domain containing protein, expressed	DEG uniquely in H21 compared to T45
MSTRG.18054	LOC_Os04g39250		DEG uniquely in H21 compared to T45
MSTRG.18058	LOC_Os04g39270	indole-3-glycerol phosphate synthase, chloroplast precursor, putative, expressed	DEG uniquely in H21 compared to T45
MSTRG.18068	LOC_Os04g39520	ZOS4-08 - C2H2 zinc finger protein, expressed	DEG uniquely in H21 compared to T45
MSTRG.18070	LOC_Os04g39489	amino acid transporter, putative, expressed	DEG uniquely in H21 compared to T45
MSTRG.18108	LOC_Os04g40090	zinc finger, ZZ type family protein, expressed	DEG uniquely in H21 compared to T45
MSTRG.18197	LOC_Os04g41260	amine oxidase, flavin-containing, domain containing protein, expressed	DEG uniquely in H21 compared to T45
MSTRG.182	LOC_Os01g04280	calmodulin binding protein, putative, expressed	DEG uniquely in H21 compared to T45
MSTRG.18200	LOC_Os04g41310	STRUBBELIG-RECEPTOR FAMILY 8 precursor, putative, expressed	DEG uniquely in H21 compared to T45
MSTRG.18288	LOC_Os04g42600	polyadenylate-binding protein, putative, expressed	DEG uniquely in H21 compared to T45

MSTRG.18373	LOC_Os04g44220	MIF4G domain containing protein, putative, expressed	DEG uniquely in H21 compared to T45
MSTRG.18387	LOC_Os04g44430		DEG uniquely in H21 compared to T45
MSTRG.18409	LOC_Os04g44780	beta-expansin precursor, putative, expressed	DEG uniquely in H21 compared to T45
MSTRG.18416	LOC_Os04g44910	receptor like protein kinase, putative, expressed	DEG uniquely in H21 compared to T45
MSTRG.18423	LOC_Os04g44950	short-chain dehydrogenase/reductase, putative, expressed	DEG uniquely in H21 compared to T45
MSTRG.18431	LOC_Os04g45010	OsPLIM2b - LIM domain protein, putative actin-binding protein and transcription factor, expressed	DEG uniquely in H21 compared to T45
MSTRG.18462	LOC_Os04g45665	hypothetical protein	DEG uniquely in H21 compared to T45
MSTRG.18507	LOC_Os04g46220	ethylene-responsive transcription factor, putative, expressed	DEG uniquely in H21 compared to T45
MSTRG.18652			DEG uniquely in H21 compared to T45
MSTRG.18666	LOC_Os04g48800;LOC_Os04g48790	rhoGAP domain containing protein, expressed;GDSL-like lipase/acylhydrolase, putative, expressed	DEG uniquely in H21 compared to T45
MSTRG.18672	LOC_Os04g48950	fringe-related protein, putative, expressed	DEG uniquely in H21 compared to T45
MSTRG.18681	LOC_Os04g49150	OsMADS17 - MADS-box family gene with MIKCc type-box, expressed	DEG uniquely in H21 compared to T45
MSTRG.18705	LOC_Os04g49510	CAMK_CAMK_like.27 - CAMK includes calcium/calmodulin depedent protein kinases, expressed	DEG uniquely in H21 compared to T45
MSTRG.1874			DEG uniquely in H21 compared to T45
MSTRG.18818	LOC_Os04g51770	expressed protein	DEG uniquely in H21 compared to T45
MSTRG.18821	LOC_Os04g51792	PAP fibrillin family domain containing protein, expressed	DEG uniquely in H21 compared to T45
MSTRG.18822	LOC_Os04g51700	DNA ligase I, ATP-dependent family protein, expressed	DEG uniquely in H21 compared to T45
MSTRG.18878	LOC_Os04g52370	UTP--glucose-1-phosphate uridylyltransferase, putative, expressed	DEG uniquely in H21 compared to T45
MSTRG.1888	LOC_Os01g40820	peptidase family M41 containing protein, expressed	DEG uniquely in H21 compared to T45
MSTRG.18917	LOC_Os04g52780	leucine-rich repeat receptor protein kinase EXS precursor, putative, expressed	DEG uniquely in H21 compared to T45
MSTRG.18926	LOC_Os04g52880		DEG uniquely in H21 compared to T45
MSTRG.1895	LOC_Os01g40980	helicase, putative, expressed	DEG uniquely in H21 compared to T45
MSTRG.18964	LOC_Os04g53360	expressed protein	DEG uniquely in H21 compared to T45
MSTRG.18974	LOC_Os04g53490		DEG uniquely in H21 compared to T45
MSTRG.19013	LOC_Os04g53970		DEG uniquely in H21 compared to T45
MSTRG.19028	LOC_Os04g54110		DEG uniquely in H21 compared to T45
MSTRG.19029	LOC_Os04g54190	cysteine-rich receptor-like protein kinase 8 precursor, putative, expressed	DEG uniquely in H21 compared to T45
MSTRG.19063	LOC_Os04g54810	beta-D-xylosidase, putative, expressed	DEG uniquely in H21 compared to T45
MSTRG.19086	LOC_Os04g55200	coatomer subunit epsilon, putative, expressed	DEG uniquely in H21 compared to T45
MSTRG.19125	LOC_Os04g55650	oryzain alpha chain precursor, putative, expressed	DEG uniquely in H21 compared to T45
MSTRG.19135	LOC_Os04g55730	alpha-N-acetylglucosaminidase, putative, expressed	DEG uniquely in H21 compared to T45
MSTRG.19141	LOC_Os04g55920	zinc-finger protein, putative, expressed	DEG uniquely in H21 compared to T45
MSTRG.1916	LOC_Os01g41420	transmembrane amino acid transporter protein, putative, expressed	DEG uniquely in H21 compared to T45
MSTRG.19166	LOC_Os04g56320	AAA-type ATPase family protein, putative, expressed	DEG uniquely in H21 compared to T45
MSTRG.19203	LOC_Os04g56840	expressed protein	DEG uniquely in H21 compared to T45
MSTRG.19205	LOC_Os04g56850	auxin response factor, putative, expressed	DEG uniquely in H21 compared to T45
MSTRG.19216	LOC_Os04g57140	kinesin motor domain containing protein, putative, expressed	DEG uniquely in H21 compared to T45
MSTRG.19226	LOC_Os04g57290	OsFBX153 - F-box domain containing protein, expressed	DEG uniquely in H21 compared to T45
MSTRG.19229	LOC_Os04g57300	phosphatidylinositol 3- and 4-kinase family protein, putative, expressed	DEG uniquely in H21 compared to T45
MSTRG.19251	LOC_Os04g57560	amine oxidase, flavin-containing, domain containing protein, expressed	DEG uniquely in H21 compared to T45
MSTRG.19257	LOC_Os04g57630	phytosulfokine receptor precursor, putative, expressed	DEG uniquely in H21 compared to T45
MSTRG.19259	LOC_Os04g57610	auxin response factor, putative, expressed	DEG uniquely in H21 compared to T45
MSTRG.19309	LOC_Os04g58250	protein kinase, putative, expressed	DEG uniquely in H21 compared to T45
MSTRG.1931	LOC_Os01g41630	serine/threonine protein phosphatase 2A 55 kDa regulatory subunit B, putative, expressed	DEG uniquely in H21 compared to T45
MSTRG.19311	LOC_Os04g58320	zinc finger, RING-type, putative, expressed	DEG uniquely in H21 compared to T45
MSTRG.19336	LOC_Os04g58640	cleavage and polyadenylation specificity factor subunit 5, putative, expressed	DEG uniquely in H21 compared to T45
MSTRG.19386	LOC_Os04g59450	CAMK_CAMK_like.28 - CAMK includes calcium/calmodulin depedent protein kinases, expressed	DEG uniquely in H21 compared to T45
MSTRG.19391	LOC_Os04g59520	anthranilate phosphoribosyltransferase, putative, expressed	DEG uniquely in H21 compared to T45
MSTRG.19407			DEG uniquely in H21 compared to T45
MSTRG.1942	LOC_Os01g41780		DEG uniquely in H21 compared to T45
MSTRG.19444	LOC_Os05g01444	polygalacturonase inhibitor precursor, putative, expressed	DEG uniquely in H21 compared to T45
MSTRG.19481	LOC_Os05g01910	pumilio-family RNA binding protein, putative, expressed	DEG uniquely in H21 compared to T45
MSTRG.19484	LOC_Os05g01994	rab5-interacting protein like, putative, expressed	DEG uniquely in H21 compared to T45

MSTRG.19503	LOC_Os05g02300	Core histone H2A/H2B/H3/H4 domain containing protein, putative, expressed	DEG uniquely in H21 compared to T45
MSTRG.19547	LOC_Os05g02890	white-brown complex homolog protein 16, putative, expressed	DEG uniquely in H21 compared to T45
MSTRG.19549	LOC_Os05g02880	splicing factor, arginine/serine-rich 7, putative, expressed	DEG uniquely in H21 compared to T45
MSTRG.19558	LOC_Os05g03030	50S ribosomal protein L10, chloroplast precursor, putative, expressed	DEG uniquely in H21 compared to T45
MSTRG.1956	LOC_Os01g41930		DEG uniquely in H21 compared to T45
MSTRG.19618	LOC_Os05g04170	AMP-binding enzyme, putative, expressed	DEG uniquely in H21 compared to T45
MSTRG.19646	LOC_Os05g04700	OsRCI2-6 - Hydrophobic protein LTI6B, expressed	DEG uniquely in H21 compared to T45
MSTRG.19688	LOC_Os05g05470	T-complex protein, putative, expressed	DEG uniquely in H21 compared to T45
MSTRG.19698	LOC_Os05g05580	expressed protein	DEG uniquely in H21 compared to T45
MSTRG.19703	LOC_Os05g05660	PWWP domain containing protein, expressed	DEG uniquely in H21 compared to T45
MSTRG.19769	LOC_Os05g06450	tubulin/FtsZ domain containing protein, putative, expressed	DEG uniquely in H21 compared to T45
MSTRG.19811	LOC_Os05g07010	myb-like DNA-binding domain containing protein, expressed	DEG uniquely in H21 compared to T45
MSTRG.19852	LOC_Os05g07790	DNA binding protein, putative, expressed	DEG uniquely in H21 compared to T45
MSTRG.19921	LOC_Os05g08970	SSRP1-like FACT complex subunit, putative, expressed	DEG uniquely in H21 compared to T45
MSTRG.19986	LOC_Os05g10810	O-acyltransferase, putative, expressed	DEG uniquely in H21 compared to T45
MSTRG.19991	LOC_Os05g10930		DEG uniquely in H21 compared to T45
MSTRG.20054	LOC_Os05g12330	uncharacterized protein KIAA1310, putative, expressed	DEG uniquely in H21 compared to T45
MSTRG.20089	LOC_Os05g13940	retrotransposon protein, putative, unclassified, expressed	DEG uniquely in H21 compared to T45
MSTRG.20100	LOC_Os05g14170	expressed protein	DEG uniquely in H21 compared to T45
MSTRG.20121			DEG uniquely in H21 compared to T45
MSTRG.20165	LOC_Os05g15770	glycosyl hydrolase, putative, expressed	DEG uniquely in H21 compared to T45
MSTRG.20194			DEG uniquely in H21 compared to T45
MSTRG.20260			DEG uniquely in H21 compared to T45
MSTRG.2034	LOC_Os01g43010	transposon protein, putative, unclassified, expressed	DEG uniquely in H21 compared to T45
MSTRG.20376	LOC_Os05g25040	expressed protein	DEG uniquely in H21 compared to T45
MSTRG.20397	LOC_Os05g25640	cytochrome P450, putative, expressed	DEG uniquely in H21 compared to T45
MSTRG.20523			DEG uniquely in H21 compared to T45
MSTRG.20572	LOC_Os05g29050	phospholipase D p1, putative, expressed	DEG uniquely in H21 compared to T45
MSTRG.20627	LOC_Os05g30695		DEG uniquely in H21 compared to T45
MSTRG.20634			DEG uniquely in H21 compared to T45
MSTRG.20637	LOC_Os05g30790	CW-type Zinc Finger, putative, expressed	DEG uniquely in H21 compared to T45
MSTRG.20722	LOC_Os05g32370	ATP-dependent RNA helicase, putative, expressed	DEG uniquely in H21 compared to T45
MSTRG.20741	LOC_Os05g32660	leucine-rich repeat family protein, putative, expressed	DEG uniquely in H21 compared to T45
MSTRG.20749			DEG uniquely in H21 compared to T45
MSTRG.20754	LOC_Os05g32980		DEG uniquely in H21 compared to T45
MSTRG.20779	LOC_Os05g33260	1-5-phosphoribosyl-5-5-phosphoribosylaminomethylideneaminoimidazole-4-carboxamide isomerase, chloroplast precursor, putative, expressed	DEG uniquely in H21 compared to T45
MSTRG.20784	LOC_Os05g33310;LOC_Os05g33320	oxidoreductase, 2OG-Fe oxygenase family protein, putative, expressed;expressed protein	DEG uniquely in H21 compared to T45
MSTRG.20807	LOC_Os05g33610	expressed protein	DEG uniquely in H21 compared to T45
MSTRG.20837			DEG uniquely in H21 compared to T45
MSTRG.20900	LOC_Os05g35200	glycosyl transferase, putative, expressed	DEG uniquely in H21 compared to T45
MSTRG.20903	LOC_Os05g35340	expressed protein	DEG uniquely in H21 compared to T45
MSTRG.20905	LOC_Os05g35266;LOC_Os05g35274	galactosyltransferase, putative, expressed;RNA recognition motif containing protein, putative, expressed	DEG uniquely in H21 compared to T45
MSTRG.20908	LOC_Os05g35400	DnaK family protein, putative, expressed	DEG uniquely in H21 compared to T45
MSTRG.2091	LOC_Os01g43844	cytochrome P450 72A1, putative, expressed	DEG uniquely in H21 compared to T45
MSTRG.21005	LOC_Os05g37390	G10 protein, putative, expressed	DEG uniquely in H21 compared to T45
MSTRG.21082	LOC_Os05g38860	expressed protein	DEG uniquely in H21 compared to T45
MSTRG.21109	LOC_Os05g39230	low photochemical bleaching 1 protein, putative, expressed	DEG uniquely in H21 compared to T45
MSTRG.21144	LOC_Os05g39870	CAMK_KIN1/SNF1/Nim1_like.24 - CAMK includes calcium/calmodulin depedent protein kinases, expressed	DEG uniquely in H21 compared to T45
MSTRG.21206	LOC_Os05g41030	LTPL67 - Protease inhibitor/seed storage/LTP family protein precursor, expressed	DEG uniquely in H21 compared to T45
MSTRG.21225	LOC_Os05g41290	disease resistance RPP13-like protein 1, putative, expressed	DEG uniquely in H21 compared to T45
MSTRG.2125	LOC_Os01g44330	laccase precursor protein, putative, expressed	DEG uniquely in H21 compared to T45
MSTRG.21254	LOC_Os05g41830		DEG uniquely in H21 compared to T45
MSTRG.21264	LOC_Os05g41950	protein kinase, putative, expressed	DEG uniquely in H21 compared to T45
MSTRG.21273	LOC_Os05g42110	allyl alcohol dehydrogenase, putative, expressed	DEG uniquely in H21 compared to T45
MSTRG.21276	LOC_Os05g42150	OsGH3.4 - Probable indole-3-acetic acid-amido synthetase, expressed	DEG uniquely in H21 compared to T45
MSTRG.21324			DEG uniquely in H21 compared to T45
MSTRG.21370	LOC_Os05g43810		DEG uniquely in H21 compared to T45
MSTRG.21386	LOC_Os05g43970	heat- and acid-stable phosphoprotein, putative, expressed	DEG uniquely in H21 compared to T45
MSTRG.21432	LOC_Os05g44922	6-phosphofructokinase, putative, expressed	DEG uniquely in H21 compared to T45
MSTRG.21449	LOC_Os05g45210	respiratory burst oxidase, putative, expressed	DEG uniquely in H21 compared to T45

MSTRG.21505	LOC_Os05g46140		DEG uniquely in H21 compared to T45
MSTRG.21585	LOC_Os05g47490	MDR-like ABC transporter, putative, expressed	DEG uniquely in H21 compared to T45
MSTRG.2162	LOC_Os01g45460	serine esterase, putative, expressed	DEG uniquely in H21 compared to T45
MSTRG.21620	LOC_Os05g47970	microtubule associated protein, putative, expressed	DEG uniquely in H21 compared to T45
MSTRG.21663	LOC_Os05g48590	OsIAA19 - Auxin-responsive Aux/IAA gene family member, expressed	DEG uniquely in H21 compared to T45
MSTRG.21690	LOC_Os05g48880	expressed protein	DEG uniquely in H21 compared to T45
MSTRG.21705	LOC_Os05g49150	eukaryotic translation initiation factor 3 subunit D, putative, expressed	DEG uniquely in H21 compared to T45
MSTRG.21706	LOC_Os05g49130	16S rRNA processing protein RimM containing protein, expressed	DEG uniquely in H21 compared to T45
MSTRG.21725	LOC_Os05g49380	OsDegp9 - Putative Deg protease homologue, expressed	DEG uniquely in H21 compared to T45
MSTRG.21748	LOC_Os05g49760	dehydrogenase, putative, expressed	DEG uniquely in H21 compared to T45
MSTRG.21756	LOC_Os05g49880	lactate/malate dehydrogenase, putative, expressed	DEG uniquely in H21 compared to T45
MSTRG.2176	LOC_Os01g45730	zinc finger C-x8-C-x5-C-x3-H type family protein, expressed	DEG uniquely in H21 compared to T45
MSTRG.21764	LOC_Os05g50020	expressed protein	DEG uniquely in H21 compared to T45
MSTRG.21805	LOC_Os05g50510	CAS1 domain-containing protein 1 precursor, putative, expressed	DEG uniquely in H21 compared to T45
MSTRG.21821	LOC_Os05g50750	AAA family ATPase, putative, expressed	DEG uniquely in H21 compared to T45
MSTRG.21852	LOC_Os05g51220	aspartic proteinase, putative, expressed	DEG uniquely in H21 compared to T45
MSTRG.21868	LOC_Os05g51590	N-rich protein, putative, expressed	DEG uniquely in H21 compared to T45
MSTRG.21879	LOC_Os05g51690	CCT motif family protein, expressed	DEG uniquely in H21 compared to T45
MSTRG.2189	LOC_Os01g45990	potassium channel AKT1, putative, expressed	DEG uniquely in H21 compared to T45
MSTRG.21926	LOC_Os06g01490	monocopper oxidase, putative, expressed	DEG uniquely in H21 compared to T45
MSTRG.21940	LOC_Os06g01710	expressed protein	DEG uniquely in H21 compared to T45
MSTRG.22019	LOC_Os06g03200	expressed protein	DEG uniquely in H21 compared to T45
MSTRG.22034	LOC_Os06g03740	folic acid binding protein, putative, expressed	DEG uniquely in H21 compared to T45
MSTRG.22046	LOC_Os06g03860	uncharacterized membrane protein, putative, expressed	DEG uniquely in H21 compared to T45
MSTRG.22081	LOC_Os06g04200	starch synthase, putative, expressed	DEG uniquely in H21 compared to T45
MSTRG.22095	LOC_Os06g04360	expressed protein	DEG uniquely in H21 compared to T45
MSTRG.22098	LOC_Os06g04390	expressed protein	DEG uniquely in H21 compared to T45
MSTRG.2212	LOC_Os01g46470		DEG uniquely in H21 compared to T45
MSTRG.22180	LOC_Os06g05390	expressed protein	DEG uniquely in H21 compared to T45
MSTRG.22230	LOC_Os06g06080	serine esterase family protein, putative, expressed	DEG uniquely in H21 compared to T45
MSTRG.22263	LOC_Os06g06480	Core histone H2A/H2B/H3/H4 domain containing protein, putative, expressed	DEG uniquely in H21 compared to T45
MSTRG.22318	LOC_Os06g07230	tyrosine protein kinase domain containing protein, putative, expressed	DEG uniquely in H21 compared to T45
MSTRG.2238	LOC_Os01g47040	C2 domain containing protein, putative, expressed	DEG uniquely in H21 compared to T45
MSTRG.22402	LOC_Os06g08660	pentatricopeptide, putative, expressed	DEG uniquely in H21 compared to T45
MSTRG.22521	LOC_Os06g10520	pantothenate kinase, putative, expressed	DEG uniquely in H21 compared to T45
MSTRG.22527			DEG uniquely in H21 compared to T45
MSTRG.22530	LOC_Os06g10660	lysM domain-containing GPI-anchored protein 1 precursor, putative, expressed	DEG uniquely in H21 compared to T45
MSTRG.22544	LOC_Os06g11050	cyclin-dependent kinase inhibitor, putative, expressed	DEG uniquely in H21 compared to T45
MSTRG.22560	LOC_Os06g11320	peptidyl-prolyl cis-trans isomerase CYP40, putative, expressed	DEG uniquely in H21 compared to T45
MSTRG.2257	LOC_Os01g47410	aspartic proteinase oryzasin-1 precursor, putative, expressed	DEG uniquely in H21 compared to T45
MSTRG.22622	LOC_Os06g12320	transmembrane amino acid transporter protein, putative, expressed	DEG uniquely in H21 compared to T45
MSTRG.22642	LOC_Os06g12590	protein kinase, putative, expressed	DEG uniquely in H21 compared to T45
MSTRG.22645	LOC_Os06g12630	glutathione S-transferase, N-terminal domain containing protein, expressed	DEG uniquely in H21 compared to T45
MSTRG.22665	LOC_Os06g12950	expressed protein	DEG uniquely in H21 compared to T45
MSTRG.22666	LOC_Os06g12950		DEG uniquely in H21 compared to T45
MSTRG.22686	LOC_Os06g13210	peptide transporter PTR2, putative, expressed	DEG uniquely in H21 compared to T45
MSTRG.22722	LOC_Os06g14030	potassium channel SKOR, putative, expressed	DEG uniquely in H21 compared to T45
MSTRG.22775	LOC_Os06g15360	RAD23 DNA repair protein, putative, expressed	DEG uniquely in H21 compared to T45
MSTRG.22821	LOC_Os06g16420	amino acid transporter, putative, expressed	DEG uniquely in H21 compared to T45
MSTRG.22859	LOC_Os06g17280	PHD-finger family protein, expressed	DEG uniquely in H21 compared to T45
MSTRG.22861	LOC_Os06g17290	phosphatidylinositol 3- and 4-kinase family protein, putative, expressed	DEG uniquely in H21 compared to T45
MSTRG.22883	LOC_Os06g18000	protein kinase domain containing protein, expressed	DEG uniquely in H21 compared to T45
MSTRG.2301			DEG uniquely in H21 compared to T45
MSTRG.23044	LOC_Os06g22380	expressed protein	DEG uniquely in H21 compared to T45
MSTRG.23103	LOC_Os06g24130	expressed protein	DEG uniquely in H21 compared to T45
MSTRG.23125			DEG uniquely in H21 compared to T45
MSTRG.23127	LOC_Os06g25294	60S ribosomal protein L18a-1, putative, expressed	DEG uniquely in H21 compared to T45
MSTRG.23145	LOC_Os06g27590		DEG uniquely in H21 compared to T45
MSTRG.23241			DEG uniquely in H21 compared to T45
MSTRG.23292	LOC_Os06g29710	OsFBD12 - F-box and FBD domain containing protein, expressed	DEG uniquely in H21 compared to T45
MSTRG.2339	LOC_Os01g48680	two pore calcium channel protein 1, putative, expressed	DEG uniquely in H21 compared to T45

MSTRG.23402	LOC_Os06g33570	cyclic nucleotide-gated ion channel 1, putative, expressed	DEG uniquely in H21 compared to T45
MSTRG.23471	LOC_Os06g36070	expressed protein	DEG uniquely in H21 compared to T45
MSTRG.23491	LOC_Os06g36450	ferroportin1 domain containing protein, expressed	DEG uniquely in H21 compared to T45
MSTRG.23497	LOC_Os06g36590	transporter, monovalent cation:proton antiporter-2 family, putative, expressed	DEG uniquely in H21 compared to T45
MSTRG.23562	LOC_Os06g37640	expressed protein	DEG uniquely in H21 compared to T45
MSTRG.23637	LOC_Os06g39260	solute carrier family 35 member B1, putative, expressed	DEG uniquely in H21 compared to T45
MSTRG.23644	LOC_Os06g39520	myristoyl-acyl carrier protein thioesterase, chloroplast precursor, putative, expressed	DEG uniquely in H21 compared to T45
MSTRG.23647	LOC_Os06g39640	PINHEAD, putative, expressed	DEG uniquely in H21 compared to T45
MSTRG.23669	LOC_Os06g40020	DEAD-box ATP-dependent RNA helicase 52A, putative, expressed	DEG uniquely in H21 compared to T45
MSTRG.23676	LOC_Os06g40110	RNA polymerase Rpc34 subunit family protein, expressed	DEG uniquely in H21 compared to T45
MSTRG.23688	LOC_Os06g40210	expressed protein	DEG uniquely in H21 compared to T45
MSTRG.23710	LOC_Os06g40570	GRAM and C2 domains containing protein, putative, expressed	DEG uniquely in H21 compared to T45
MSTRG.23731	LOC_Os06g40880	polygalacturonase, putative, expressed	DEG uniquely in H21 compared to T45
MSTRG.23732	LOC_Os06g40840	microtubule associated protein, putative, expressed	DEG uniquely in H21 compared to T45
MSTRG.238	LOC_Os01g03390	BBTI7 - Bowman-Birk type bran trypsin inhibitor precursor, expressed	DEG uniquely in H21 compared to T45
MSTRG.2385	LOC_Os01g49300		DEG uniquely in H21 compared to T45
MSTRG.2387	LOC_Os01g49320	glycosyl hydrolase, putative, expressed	DEG uniquely in H21 compared to T45
MSTRG.23990	LOC_Os06g44010	WRKY28, expressed	DEG uniquely in H21 compared to T45
MSTRG.24022	LOC_Os06g44450	CCT/B-box zinc finger protein, putative, expressed	DEG uniquely in H21 compared to T45
MSTRG.24071	LOC_Os06g45330	endonuclease V, putative, expressed	DEG uniquely in H21 compared to T45
MSTRG.24093	LOC_Os06g45670	glycine cleavage system H protein, putative, expressed	DEG uniquely in H21 compared to T45
MSTRG.24098	LOC_Os06g45770	BTB/POZ domain containing protein, putative, expressed	DEG uniquely in H21 compared to T45
MSTRG.2412	LOC_Os01g49770	zinc finger, C3HC4 type domain containing protein, expressed	DEG uniquely in H21 compared to T45
MSTRG.24178	LOC_Os06g46930	ribosomal protein L24, putative, expressed	DEG uniquely in H21 compared to T45
MSTRG.24206	LOC_Os06g47420	transposon protein, putative, unclassified, expressed	DEG uniquely in H21 compared to T45
MSTRG.24220	LOC_Os06g47550	cadmium/zinc-transporting ATPase, putative, expressed	DEG uniquely in H21 compared to T45
MSTRG.24310	LOC_Os06g48960	AIG2-like family domain containing protein, expressed	DEG uniquely in H21 compared to T45
MSTRG.24327	LOC_Os06g49150	exostosin, putative, expressed	DEG uniquely in H21 compared to T45
MSTRG.24394	LOC_Os06g50140	endoglucanase, putative, expressed	DEG uniquely in H21 compared to T45
MSTRG.24444	LOC_Os06g50499	expressed protein	DEG uniquely in H21 compared to T45
MSTRG.2446	LOC_Os01g50259	expressed protein	DEG uniquely in H21 compared to T45
MSTRG.2452	LOC_Os01g50310		DEG uniquely in H21 compared to T45
MSTRG.24543	LOC_Os07g01904	expressed protein	DEG uniquely in H21 compared to T45
MSTRG.24557	LOC_Os07g02100;LOC_Os07g02120	flavin-containing monooxygenase family protein, putative, expressed	DEG uniquely in H21 compared to T45
MSTRG.24593	LOC_Os07g02930	OsFBX210 - F-box domain containing protein, expressed	DEG uniquely in H21 compared to T45
MSTRG.24622	LOC_Os07g03240		DEG uniquely in H21 compared to T45
MSTRG.24649	LOC_Os07g04200	bacterial transferase hexapeptide domain containing protein, expressed	DEG uniquely in H21 compared to T45
MSTRG.24656	LOC_Os07g04270		DEG uniquely in H21 compared to T45
MSTRG.2467	LOC_Os01g50470	regulator of chromosome condensation, putative, expressed	DEG uniquely in H21 compared to T45
MSTRG.24677			DEG uniquely in H21 compared to T45
MSTRG.2477			DEG uniquely in H21 compared to T45
MSTRG.24786	LOC_Os07g06730		DEG uniquely in H21 compared to T45
MSTRG.24788			DEG uniquely in H21 compared to T45
MSTRG.24796	LOC_Os07g06890	FAD-linked oxidoreductase protein, putative, expressed	DEG uniquely in H21 compared to T45
MSTRG.24814			DEG uniquely in H21 compared to T45
MSTRG.24831	LOC_Os07g07350	zinc finger A20 and AN1 domain-containing stress-associated protein, putative, expressed	DEG uniquely in H21 compared to T45
MSTRG.24836	LOC_Os07g07420	gibberellin 20 oxidase 1-B, putative, expressed	DEG uniquely in H21 compared to T45
MSTRG.2494	LOC_Os01g50940	helix-loop-helix DNA-binding domain containing protein, expressed	DEG uniquely in H21 compared to T45
MSTRG.24944			DEG uniquely in H21 compared to T45
MSTRG.24949	LOC_Os07g08950	FAD-linked oxidoreductase protein, putative, expressed	DEG uniquely in H21 compared to T45
MSTRG.25013	LOC_Os07g10120	expressed protein	DEG uniquely in H21 compared to T45
MSTRG.25021	LOC_Os07g10300	expressed protein	DEG uniquely in H21 compared to T45
MSTRG.25095	LOC_Os07g11300	expressed protein	DEG uniquely in H21 compared to T45
MSTRG.25148	LOC_Os07g12530	ABC1 family domain containing protein, putative, expressed	DEG uniquely in H21 compared to T45
MSTRG.25182	LOC_Os07g13490	expressed protein	DEG uniquely in H21 compared to T45
MSTRG.25192	LOC_Os07g13634	cytokinin-N-glucosyltransferase 1, putative, expressed	DEG uniquely in H21 compared to T45
MSTRG.25238	LOC_Os07g15270	RNA-binding region RNP-1, putative, expressed	DEG uniquely in H21 compared to T45
MSTRG.2529	LOC_Os01g51430	green ripe-like, putative, expressed	DEG uniquely in H21 compared to T45
MSTRG.253	LOC_Os01g03570	transcription factor X1, putative, expressed	DEG uniquely in H21 compared to T45

MSTRG.2536	LOC_Os01g51620		DEG uniquely in H21 compared to T45
MSTRG.25375	LOC_Os07g19530	peptidase, putative, expressed	DEG uniquely in H21 compared to T45
MSTRG.25387	LOC_Os07g20600	retrotransposon, putative, centromere-specific	DEG uniquely in H21 compared to T45
MSTRG.25395	LOC_Os07g22400	POLA3 - Putative DNA polymerase alpha complex subunit, expressed	DEG uniquely in H21 compared to T45
MSTRG.25464			DEG uniquely in H21 compared to T45
MSTRG.25468	LOC_Os07g24660		DEG uniquely in H21 compared to T45
MSTRG.2561	LOC_Os01g52140	expressed protein	DEG uniquely in H21 compared to T45
MSTRG.25611	LOC_Os07g29060		DEG uniquely in H21 compared to T45
MSTRG.25630	LOC_Os07g29480	transposon protein, putative, CACTA, En/Spm sub-class, expressed	DEG uniquely in H21 compared to T45
MSTRG.25633	LOC_Os07g29470		DEG uniquely in H21 compared to T45
MSTRG.25667	LOC_Os07g30300	small G protein family protein, putative, expressed	DEG uniquely in H21 compared to T45
MSTRG.25691	LOC_Os07g31194	expressed protein	DEG uniquely in H21 compared to T45
MSTRG.25718	LOC_Os07g31670	expressed protein	DEG uniquely in H21 compared to T45
MSTRG.25751	LOC_Os07g32480	mitotic checkpoint serine/threonine-protein kinase BUB1, putative, expressed	DEG uniquely in H21 compared to T45
MSTRG.2579	LOC_Os01g52450	hexokinase, putative, expressed	DEG uniquely in H21 compared to T45
MSTRG.25824	LOC_Os07g33670	patatin-like phospholipase family protein, expressed	DEG uniquely in H21 compared to T45
MSTRG.2584	LOC_Os01g52500	NADP-dependent malic enzyme, putative, expressed	DEG uniquely in H21 compared to T45
MSTRG.25849	LOC_Os07g33860		DEG uniquely in H21 compared to T45
MSTRG.2586	LOC_Os01g52550	ABC transporter, ATP-binding protein, putative, expressed	DEG uniquely in H21 compared to T45
MSTRG.25863	LOC_Os07g34598	tyrosyl-DNA phosphodiesterase 1, putative, expressed	DEG uniquely in H21 compared to T45
MSTRG.25865	LOC_Os07g34640	pyruvate Pi dikinase regulatory protein, putative, expressed	DEG uniquely in H21 compared to T45
MSTRG.25866	LOC_Os07g34650	expressed protein	DEG uniquely in H21 compared to T45
MSTRG.25868	LOC_Os07g34680		DEG uniquely in H21 compared to T45
MSTRG.2589	LOC_Os01g52610;LOC_Os01g52630	membrane associated DUF588 domain containing protein, putative, expressed;regulator of chromosome condensation, putative, expressed	DEG uniquely in H21 compared to T45
MSTRG.25896	LOC_Os07g35290	TKL_IRAK_DUF26-lc.10 - DUF26 kinases have homology to DUF26 containing loci, expressed	DEG uniquely in H21 compared to T45
MSTRG.25905	LOC_Os07g35580;LOC_Os07g35600	TKL_IRAK_DUF26-lc.20 - DUF26 kinases have homology to DUF26 containing loci, expressed;expressed protein	DEG uniquely in H21 compared to T45
MSTRG.25938			DEG uniquely in H21 compared to T45
MSTRG.2599	LOC_Os01g52690		DEG uniquely in H21 compared to T45
MSTRG.26001	LOC_Os07g37150	expressed protein	DEG uniquely in H21 compared to T45
MSTRG.26057	LOC_Os07g38070	protein kinase, putative, expressed	DEG uniquely in H21 compared to T45
MSTRG.26063	LOC_Os07g38130	polygalacturonase inhibitor 1 precursor, putative, expressed	DEG uniquely in H21 compared to T45
MSTRG.26073	LOC_Os07g38260	insulin-degrading enzyme, putative, expressed	DEG uniquely in H21 compared to T45
MSTRG.26161	LOC_Os07g39520	STE_PAK_Ste20_Slob_Wnk.5 - STE kinases include homologs to sterile 7, sterile 11 and sterile 20 from yeast, expressed	DEG uniquely in H21 compared to T45
MSTRG.26237	LOC_Os07g40730	SNF2 domain-containing protein, putative, expressed	DEG uniquely in H21 compared to T45
MSTRG.26398	LOC_Os07g43540	ORC6 - Putative origin recognition complex subunit 6, expressed	DEG uniquely in H21 compared to T45
MSTRG.26417	LOC_Os07g43950;LOC_Os07g43960	RNA recognition motif containing protein, putative, expressed;expressed protein	DEG uniquely in H21 compared to T45
MSTRG.26444	LOC_Os07g44400	POK1, putative, expressed	DEG uniquely in H21 compared to T45
MSTRG.2657	LOC_Os01g53560	phosphoesterase, putative, expressed	DEG uniquely in H21 compared to T45
MSTRG.26585	LOC_Os07g46560	seven in absentia protein family domain containing protein, expressed	DEG uniquely in H21 compared to T45
MSTRG.26623	LOC_Os07g46990	copper/zinc superoxide dismutase, putative, expressed	DEG uniquely in H21 compared to T45
MSTRG.26626	LOC_Os07g47040		DEG uniquely in H21 compared to T45
MSTRG.26663	LOC_Os07g47530	AAA-type ATPase family protein, putative, expressed	DEG uniquely in H21 compared to T45
MSTRG.26672	LOC_Os07g47680	transposon protein, putative, Pong sub-class, expressed	DEG uniquely in H21 compared to T45
MSTRG.26700	LOC_Os07g48180		DEG uniquely in H21 compared to T45
MSTRG.26723	LOC_Os07g48550	no apical meristem protein, putative, expressed	DEG uniquely in H21 compared to T45
MSTRG.2677	LOC_Os01g53810	peptidase, putative, expressed	DEG uniquely in H21 compared to T45
MSTRG.26783	LOC_Os07g49270	AMP deaminase, putative, expressed	DEG uniquely in H21 compared to T45
MSTRG.26784	LOC_Os07g49280	PMR5, putative, expressed	DEG uniquely in H21 compared to T45
MSTRG.26796	LOC_Os07g49470	protein kinase APK1B, chloroplast precursor, putative, expressed	DEG uniquely in H21 compared to T45
MSTRG.26826	LOC_Os08g01390	phosphatidylinositol-4-phosphate 5-Kinase, putative, expressed	DEG uniquely in H21 compared to T45
MSTRG.26831	LOC_Os08g01590	expressed protein	DEG uniquely in H21 compared to T45
MSTRG.26859	LOC_Os08g01920	SGR2, putative, expressed	DEG uniquely in H21 compared to T45
MSTRG.27000	LOC_Os08g04340	plastocyanin-like domain containing protein, putative, expressed	DEG uniquely in H21 compared to T45
MSTRG.27012	LOC_Os08g04630	external NADH-ubiquinone oxidoreductase 1, mitochondrial precursor, putative, expressed	DEG uniquely in H21 compared to T45
MSTRG.2703	LOC_Os01g54190	expressed protein	DEG uniquely in H21 compared to T45
MSTRG.2707	LOC_Os01g54300	OsMan02 - Endo-Beta-Mannanase, expressed	DEG uniquely in H21 compared to T45
MSTRG.27071	LOC_Os08g05830	transaldolase, putative, expressed	DEG uniquely in H21 compared to T45

MSTRG.27124	LOC_Os08g06480	lissencephaly type-1-like homology motif, putative, expressed	DEG uniquely in H21 compared to T45
MSTRG.27137	LOC_Os08g06620;LOC_Os08g06630		DEG uniquely in H21 compared to T45
MSTRG.27159	LOC_Os08g06890	expressed protein	DEG uniquely in H21 compared to T45
MSTRG.27177	LOC_Os08g07550	hAT dimerisation domain-containing protein, putative, expressed	DEG uniquely in H21 compared to T45
MSTRG.27222	LOC_Os08g08500	reductase, putative, expressed	DEG uniquely in H21 compared to T45
MSTRG.27267	LOC_Os08g09610	expressed protein	DEG uniquely in H21 compared to T45
MSTRG.27368	LOC_Os08g11760	expressed protein	DEG uniquely in H21 compared to T45
MSTRG.2737	LOC_Os01g54700	retrotransposon protein, putative, unclassified, expressed	DEG uniquely in H21 compared to T45
MSTRG.27485			DEG uniquely in H21 compared to T45
MSTRG.27486	LOC_Os08g13630	expressed protein	DEG uniquely in H21 compared to T45
MSTRG.27507	LOC_Os08g13905	expressed protein	DEG uniquely in H21 compared to T45
MSTRG.27528	LOC_Os08g14570	NADPH reductase, putative, expressed	DEG uniquely in H21 compared to T45
MSTRG.27537	LOC_Os08g14990		DEG uniquely in H21 compared to T45
MSTRG.27546	LOC_Os08g15149		DEG uniquely in H21 compared to T45
MSTRG.27547	LOC_Os08g15170	ATP synthase epsilon chain, mitochondrial, putative, expressed	DEG uniquely in H21 compared to T45
MSTRG.27605	LOC_Os08g16720	retrotransposon protein, putative, unclassified, expressed	DEG uniquely in H21 compared to T45
MSTRG.27615	LOC_Os08g16830	retrotransposon protein, putative, SINE subclass, expressed	DEG uniquely in H21 compared to T45
MSTRG.27634	LOC_Os08g17370	transmembrane 9 superfamily member, putative, expressed	DEG uniquely in H21 compared to T45
MSTRG.2764	LOC_Os01g55000	expressed protein	DEG uniquely in H21 compared to T45
MSTRG.27670	LOC_Os08g19300	retrotransposon protein, putative, unclassified, expressed	DEG uniquely in H21 compared to T45
MSTRG.27696			DEG uniquely in H21 compared to T45
MSTRG.27729	LOC_Os08g20670	retrotransposon protein, putative, unclassified, expressed	DEG uniquely in H21 compared to T45
MSTRG.2775	LOC_Os01g55094	expressed protein	DEG uniquely in H21 compared to T45
MSTRG.27755			DEG uniquely in H21 compared to T45
MSTRG.27757	LOC_Os08g22330	retrotransposon protein, putative, unclassified, expressed	DEG uniquely in H21 compared to T45
MSTRG.27867	LOC_Os08g25140	ethanol tolerance protein GEKO1, putative, expressed	DEG uniquely in H21 compared to T45
MSTRG.2789	LOC_Os01g55350		DEG uniquely in H21 compared to T45
MSTRG.27933	LOC_Os08g27810	OsWAK115 - OsWAK receptor-like protein OsWAK-RLP, expressed	DEG uniquely in H21 compared to T45
MSTRG.27937	LOC_Os08g27840	phosphoenolpyruvate carboxylase, putative, expressed	DEG uniquely in H21 compared to T45
MSTRG.28002	LOC_Os08g29230		DEG uniquely in H21 compared to T45
MSTRG.28004			DEG uniquely in H21 compared to T45
MSTRG.28018	LOC_Os08g29580	expressed protein	DEG uniquely in H21 compared to T45
MSTRG.28034	LOC_Os08g29809	resistance protein LR10, putative, expressed	DEG uniquely in H21 compared to T45
MSTRG.28079	LOC_Os08g30719	expressed protein	DEG uniquely in H21 compared to T45
MSTRG.28086	LOC_Os08g30830		DEG uniquely in H21 compared to T45
MSTRG.28095	LOC_Os08g31060	phospholipase D alpha 1, putative, expressed	DEG uniquely in H21 compared to T45
MSTRG.28112	LOC_Os08g31350		DEG uniquely in H21 compared to T45
MSTRG.28134	LOC_Os08g31780		DEG uniquely in H21 compared to T45
MSTRG.28135	LOC_Os08g31769	expressed protein	DEG uniquely in H21 compared to T45
MSTRG.28137	LOC_Os08g31790	expressed protein	DEG uniquely in H21 compared to T45
MSTRG.28146	LOC_Os08g31930	BRCA1 C Terminus domain containing protein, expressed	DEG uniquely in H21 compared to T45
MSTRG.28178	LOC_Os08g32540		DEG uniquely in H21 compared to T45
MSTRG.28256	LOC_Os08g33740	CSLA11 - cellulose synthase-like family A, expressed	DEG uniquely in H21 compared to T45
MSTRG.28275	LOC_Os08g34060	DUF1336 domain containing protein, expressed	DEG uniquely in H21 compared to T45
MSTRG.28315	LOC_Os08g34720	D-3-phosphoglycerate dehydrogenase, chloroplast precursor, putative, expressed	DEG uniquely in H21 compared to T45
MSTRG.2837	LOC_Os01g56020	expressed protein	DEG uniquely in H21 compared to T45
MSTRG.28403	LOC_Os08g36590	antifreeze glycoprotein, putative, expressed	DEG uniquely in H21 compared to T45
MSTRG.28510			DEG uniquely in H21 compared to T45
MSTRG.28534	LOC_Os08g39040;LOC_Os08g39030	expressed protein	DEG uniquely in H21 compared to T45
MSTRG.28554	LOC_Os08g39320	expressed protein	DEG uniquely in H21 compared to T45
MSTRG.28575	LOC_Os08g39830	ethylene-insensitive 3, putative, expressed	DEG uniquely in H21 compared to T45
MSTRG.28606	LOC_Os08g40060	expressed protein	DEG uniquely in H21 compared to T45
MSTRG.28667	LOC_Os08g41110	chaperone protein dnaJ 10, putative, expressed	DEG uniquely in H21 compared to T45
MSTRG.28700	LOC_Os08g41580	ubiquitin carboxyl-terminal hydrolase, putative, expressed	DEG uniquely in H21 compared to T45
MSTRG.28702	LOC_Os08g41620	ubiquitin carboxyl-terminal hydrolase family protein, expressed	DEG uniquely in H21 compared to T45
MSTRG.28761	LOC_Os08g42400	no apical meristem protein, putative, expressed	DEG uniquely in H21 compared to T45
MSTRG.28778			DEG uniquely in H21 compared to T45
MSTRG.28793	LOC_Os08g42910	peptidase, M24 family protein, putative, expressed	DEG uniquely in H21 compared to T45
MSTRG.28797	LOC_Os08g42940		DEG uniquely in H21 compared to T45
MSTRG.28815	LOC_Os08g43190	dehydrogenase, putative, expressed	DEG uniquely in H21 compared to T45
MSTRG.28824	LOC_Os08g43310	PE-PGRS FAMILY PROTEIN, putative, expressed	DEG uniquely in H21 compared to T45
MSTRG.28947	LOC_Os08g44930	SNARE domain containing protein, putative, expressed	DEG uniquely in H21 compared to T45
MSTRG.28968	LOC_Os08g45170	carboxyl-terminal peptidase, putative, expressed	DEG uniquely in H21 compared to T45

MSTRG.29120	LOC_Os09g04720	SWIB/MDM2 domain containing protein, expressed	DEG uniquely in H21 compared to T45
MSTRG.29124	LOC_Os09g04790	PAP fibrillin family domain containing protein, expressed	DEG uniquely in H21 compared to T45
MSTRG.29153			DEG uniquely in H21 compared to T45
MSTRG.292	LOC_Os01g04720	leucine-rich repeat protein-related, putative, expressed	DEG uniquely in H21 compared to T45
MSTRG.29242	LOC_Os09g09470	WD domain, G-beta repeat domain containing protein, expressed	DEG uniquely in H21 compared to T45
MSTRG.29250	LOC_Os09g09980	glucan endo-1,3-beta-glucosidase precursor, putative, expressed	DEG uniquely in H21 compared to T45
MSTRG.29393			DEG uniquely in H21 compared to T45
MSTRG.29418	LOC_Os09g15480		DEG uniquely in H21 compared to T45
MSTRG.29428	LOC_Os09g15700	receptor-like protein kinase 5 precursor, putative, expressed	DEG uniquely in H21 compared to T45
MSTRG.29433	LOC_Os09g15780	expressed protein	DEG uniquely in H21 compared to T45
MSTRG.29439	LOC_Os09g15850	Leucine Rich Repeat family protein, expressed	DEG uniquely in H21 compared to T45
MSTRG.2945	LOC_Os01g57450		DEG uniquely in H21 compared to T45
MSTRG.29477	LOC_Os09g17146	expressed protein	DEG uniquely in H21 compared to T45
MSTRG.29478	LOC_Os09g17152	OsFBX319 - F-box domain containing protein, expressed	DEG uniquely in H21 compared to T45
MSTRG.29488	LOC_Os09g17670		DEG uniquely in H21 compared to T45
MSTRG.29516	LOC_Os09g19170	expressed protein	DEG uniquely in H21 compared to T45
MSTRG.29541	LOC_Os09g19800;LOC_Os09g19820	aminopeptidase, putative, expressed	DEG uniquely in H21 compared to T45
MSTRG.29547	LOC_Os09g19910	expressed protein	DEG uniquely in H21 compared to T45
MSTRG.2957	LOC_Os01g57570	NADPH-dependent FMN reductase domain containing protein, expressed	DEG uniquely in H21 compared to T45
MSTRG.29580	LOC_Os09g20590	CD2-binding protein-related, putative, expressed	DEG uniquely in H21 compared to T45
MSTRG.29599	LOC_Os09g21110	leucyl-tRNA synthetase, cytoplasmic, putative, expressed	DEG uniquely in H21 compared to T45
MSTRG.29621	LOC_Os09g21510	STE_MEKK_ste11_MAP3K.2 - STE kinases include homologs to sterile 7, sterile 11 and sterile 20 from yeast, expressed	DEG uniquely in H21 compared to T45
MSTRG.29671	LOC_Os09g22540	transcription factor jumonji, putative, expressed	DEG uniquely in H21 compared to T45
MSTRG.29744	LOC_Os09g24924	transporter family protein, putative, expressed	DEG uniquely in H21 compared to T45
MSTRG.29783	LOC_Os09g25620;LOC_Os09g25625	CPuORF8 - conserved peptide uORF-containing transcript, expressed;S-adenosyl-l-methionine decarboxylase leader peptide, putative, expressed	DEG uniquely in H21 compared to T45
MSTRG.29784	LOC_Os09g25600;LOC_Os09g25590	tsi1-interacting protein TSIP1, putative, expressed;AP2 domain containing protein, expressed	DEG uniquely in H21 compared to T45
MSTRG.29789	LOC_Os09g25740	expressed protein	DEG uniquely in H21 compared to T45
MSTRG.29815	LOC_Os09g26000		DEG uniquely in H21 compared to T45
MSTRG.29824	LOC_Os09g26340	Core histone H2A/H2B/H3/H4 domain containing protein, putative, expressed	DEG uniquely in H21 compared to T45
MSTRG.29834	LOC_Os09g26560	expressed protein	DEG uniquely in H21 compared to T45
MSTRG.29889	LOC_Os09g27590	expressed protein	DEG uniquely in H21 compared to T45
MSTRG.29910	LOC_Os09g27890	lysM domain-containing GPI-anchored protein precursor, putative, expressed	DEG uniquely in H21 compared to T45
MSTRG.29932			DEG uniquely in H21 compared to T45
MSTRG.29961	LOC_Os09g28690	gibberellin receptor GID1L2, putative, expressed	DEG uniquely in H21 compared to T45
MSTRG.29963	LOC_Os09g28730	gibberellin receptor GID1L2, putative, expressed	DEG uniquely in H21 compared to T45
MSTRG.29990	LOC_Os09g29360	helix-loop-helix DNA-binding protein, putative, expressed	DEG uniquely in H21 compared to T45
MSTRG.30004	LOC_Os09g29490		DEG uniquely in H21 compared to T45
MSTRG.30102	LOC_Os09g31350	expressed protein	DEG uniquely in H21 compared to T45
MSTRG.30130	LOC_Os09g32100	expressed protein	DEG uniquely in H21 compared to T45
MSTRG.30139	LOC_Os09g32220	pob, putative, expressed	DEG uniquely in H21 compared to T45
MSTRG.3023	LOC_Os01g58380	3-hydroxybutyryl-CoA dehydrogenase, putative, expressed	DEG uniquely in H21 compared to T45
MSTRG.30268	LOC_Os09g34150	NBS-LRR disease resistance protein, putative, expressed	DEG uniquely in H21 compared to T45
MSTRG.30289	LOC_Os09g34920	glycosyl hydrolase family 29, putative, expressed	DEG uniquely in H21 compared to T45
MSTRG.30298	LOC_Os09g35000	flap endonuclease, putative, expressed	DEG uniquely in H21 compared to T45
MSTRG.30308	LOC_Os09g35680	OsFBX339 - F-box domain containing protein, expressed	DEG uniquely in H21 compared to T45
MSTRG.30319	LOC_Os09g35800	UDP-glucose 4-epimerase, putative, expressed	DEG uniquely in H21 compared to T45
MSTRG.30343	LOC_Os09g36130	expressed protein	DEG uniquely in H21 compared to T45
MSTRG.30359	LOC_Os09g36360		DEG uniquely in H21 compared to T45
MSTRG.30384			DEG uniquely in H21 compared to T45
MSTRG.3047	LOC_Os01g58740	glycerol-3-phosphate dehydrogenase, putative, expressed	DEG uniquely in H21 compared to T45
MSTRG.30519	LOC_Os09g38550	protein phosphatase 2C, putative, expressed	DEG uniquely in H21 compared to T45
MSTRG.3057	LOC_Os01g59020	cytochrome P450, putative, expressed	DEG uniquely in H21 compared to T45
MSTRG.3065	LOC_Os01g59120	cyclin, putative, expressed	DEG uniquely in H21 compared to T45
MSTRG.3101	LOC_Os01g59610	KAZ1 - Kazal-type serine protease inhibitor precursor, expressed	DEG uniquely in H21 compared to T45
MSTRG.3148	LOC_Os01g60170	DUF567 domain containing protein, putative, expressed	DEG uniquely in H21 compared to T45
MSTRG.3154	LOC_Os01g60260	KH domain containing protein, putative, expressed	DEG uniquely in H21 compared to T45

MSTRG.3195	LOC_Os01g61080	WRKY24, expressed	DEG uniquely in H21 compared to T45
MSTRG.3244	LOC_Os01g61920	Core histone H2A/H2B/H3/H4 domain containing protein, putative, expressed	DEG uniquely in H21 compared to T45
MSTRG.3282	LOC_Os01g62440	ribonuclease protein, putative, expressed	DEG uniquely in H21 compared to T45
MSTRG.3412	LOC_Os01g64660	fructose-1,6-bisphosphatase, putative, expressed	DEG uniquely in H21 compared to T45
MSTRG.3434	LOC_Os01g64970	CAMK_CAMK_like.11 - CAMK includes calcium/calmodulin depedent protein kinases, expressed	DEG uniquely in H21 compared to T45
MSTRG.3524	LOC_Os01g66190	expressed protein	DEG uniquely in H21 compared to T45
MSTRG.3538	LOC_Os01g66330	ATP-dependent Clp protease ATP-binding subunit clpX, putative, expressed	DEG uniquely in H21 compared to T45
MSTRG.3552	LOC_Os01g66600	rhodanese-like, putative, expressed	DEG uniquely in H21 compared to T45
MSTRG.357	LOC_Os01g05630;LOC_Os01g05800;LOC_Os01g06010	Core histone H2A/H2B/H3/H4 domain containing protein, putative, expressed	DEG uniquely in H21 compared to T45
MSTRG.3573	LOC_Os01g66970	zinc finger, C3HC4 type domain containing protein, expressed	DEG uniquely in H21 compared to T45
MSTRG.3603	LOC_Os01g67360	methyltransferase, putative, expressed	DEG uniquely in H21 compared to T45
MSTRG.3612	LOC_Os01g67420		DEG uniquely in H21 compared to T45
MSTRG.3633	LOC_Os01g67810	transposon protein, putative, unclassified, expressed	DEG uniquely in H21 compared to T45
MSTRG.3634	LOC_Os01g67850	zinc finger, RING-type, putative, expressed	DEG uniquely in H21 compared to T45
MSTRG.3653			DEG uniquely in H21 compared to T45
MSTRG.3661	LOC_Os01g68240		DEG uniquely in H21 compared to T45
MSTRG.3707	LOC_Os01g68930	expressed protein	DEG uniquely in H21 compared to T45
MSTRG.3719	LOC_Os01g69100	expressed protein	DEG uniquely in H21 compared to T45
MSTRG.3733	LOC_Os01g69830	OsSPL2 - SBP-box gene family member, expressed	DEG uniquely in H21 compared to T45
MSTRG.3738	LOC_Os01g69890		DEG uniquely in H21 compared to T45
MSTRG.3799	LOC_Os01g70550	heparan-alpha-glucosaminide N-acetyltransferase, putative, expressed	DEG uniquely in H21 compared to T45
MSTRG.381	LOC_Os01g05960		DEG uniquely in H21 compared to T45
MSTRG.3838	LOC_Os01g71200	RNA recognition motif containing protein, putative, expressed	DEG uniquely in H21 compared to T45
MSTRG.3863	LOC_Os01g71620	expressed protein	DEG uniquely in H21 compared to T45
MSTRG.3882	LOC_Os01g72009	expressed protein	DEG uniquely in H21 compared to T45
MSTRG.3907	LOC_Os01g72380	expressed protein	DEG uniquely in H21 compared to T45
MSTRG.3946	LOC_Os01g72980	tRNA uridine 5-carboxymethylaminomethyl modification enzyme gidA, putative, expressed	DEG uniquely in H21 compared to T45
MSTRG.3952	LOC_Os01g73040	CBS domain-containing protein, putative, expressed	DEG uniquely in H21 compared to T45
MSTRG.3988	LOC_Os01g73580	glycosyl hydrolases, putative, expressed	DEG uniquely in H21 compared to T45
MSTRG.4033			DEG uniquely in H21 compared to T45
MSTRG.4073	LOC_Os10g01380	CW7, putative, expressed	DEG uniquely in H21 compared to T45
MSTRG.4081	LOC_Os10g01610		DEG uniquely in H21 compared to T45
MSTRG.4112	LOC_Os10g02880	O-methyltransferase, putative, expressed	DEG uniquely in H21 compared to T45
MSTRG.4143	LOC_Os10g03780	OsFBX351 - F-box domain containing protein, expressed	DEG uniquely in H21 compared to T45
MSTRG.4155	LOC_Os10g04050	transposon protein, putative, CACTA, En/Spm sub-class, expressed	DEG uniquely in H21 compared to T45
MSTRG.4189	LOC_Os10g05020	cytochrome P450, putative, expressed	DEG uniquely in H21 compared to T45
MSTRG.4214	LOC_Os10g05580	expressed protein	DEG uniquely in H21 compared to T45
MSTRG.4287			DEG uniquely in H21 compared to T45
MSTRG.4307	LOC_Os10g08540	cytochrome P450, putative, expressed	DEG uniquely in H21 compared to T45
MSTRG.4327	LOC_Os10g09200	expressed protein	DEG uniquely in H21 compared to T45
MSTRG.438	LOC_Os01g07330	expressed protein	DEG uniquely in H21 compared to T45
MSTRG.4398			DEG uniquely in H21 compared to T45
MSTRG.4443			DEG uniquely in H21 compared to T45
MSTRG.4469	LOC_Os10g17100		DEG uniquely in H21 compared to T45
MSTRG.4503			DEG uniquely in H21 compared to T45
MSTRG.4599	LOC_Os10g21810	histidine kinase, putative, expressed	DEG uniquely in H21 compared to T45
MSTRG.4608	LOC_Os10g22050		DEG uniquely in H21 compared to T45
MSTRG.4616	LOC_Os10g22394	retrotransposon protein, putative, unclassified, expressed	DEG uniquely in H21 compared to T45
MSTRG.4623	LOC_Os10g22520	cellulase, putative, expressed	DEG uniquely in H21 compared to T45
MSTRG.469	LOC_Os01g07730	phosphate/phosphoenolpyruvate translocator-related protein, putative, expressed	DEG uniquely in H21 compared to T45
MSTRG.470	LOC_Os01g07740	DEAD-box ATP-dependent RNA helicase 14, putative, expressed	DEG uniquely in H21 compared to T45
MSTRG.4702	LOC_Os10g25010	OsCML8 - Calmodulin-related calcium sensor protein, expressed	DEG uniquely in H21 compared to T45
MSTRG.4713	LOC_Os10g25130	aminotransferase, classes I and II, domain containing protein, expressed	DEG uniquely in H21 compared to T45
MSTRG.4721	LOC_Os10g25290	ZIM domain containing protein, putative, expressed	DEG uniquely in H21 compared to T45
MSTRG.4744	LOC_Os10g26280	ORC3 - Putative origin recognition complex subunit 3, expressed	DEG uniquely in H21 compared to T45
MSTRG.4781	LOC_Os10g27040	retrotransposon protein, putative, unclassified, expressed	DEG uniquely in H21 compared to T45
MSTRG.4805	LOC_Os10g27390	no apical meristem protein, putative, expressed	DEG uniquely in H21 compared to T45
MSTRG.4858	LOC_Os10g29159		DEG uniquely in H21 compared to T45
MSTRG.4883	LOC_Os10g29660;LOC_Os10g29670	TATA-binding protein, putative, expressed;expressed protein	DEG uniquely in H21 compared to T45
MSTRG.4905	LOC_Os10g30280	OsFBX386 - F-box domain containing protein, expressed	DEG uniquely in H21 compared to T45

MSTRG.4923	LOC_Os10g30670	transposon protein, putative, CACTA, En/Spm sub-class, expressed	DEG uniquely in H21 compared to T45
MSTRG.4947			DEG uniquely in H21 compared to T45
MSTRG.502	LOC_Os01g08190	transcriptional corepressor LEUNIG, putative, expressed	DEG uniquely in H21 compared to T45
MSTRG.5023	LOC_Os10g33170	POT domain containing peptide transporter, putative, expressed	DEG uniquely in H21 compared to T45
MSTRG.5024	LOC_Os10g33204		DEG uniquely in H21 compared to T45
MSTRG.5033	LOC_Os10g33310	cyclin-dependent kinase inhibitor, putative, expressed	DEG uniquely in H21 compared to T45
MSTRG.507	LOC_Os01g08290	transmembrane protein, putative, expressed	DEG uniquely in H21 compared to T45
MSTRG.5077	LOC_Os10g33930	DUF1336 domain containing protein, expressed	DEG uniquely in H21 compared to T45
MSTRG.5132	LOC_Os10g34820	CDT1B - Putative DNA replication initiation protein, expressed	DEG uniquely in H21 compared to T45
MSTRG.5137	LOC_Os10g35010	ATTIC110/TIC110, putative, expressed	DEG uniquely in H21 compared to T45
MSTRG.5145	LOC_Os10g35070	alpha-galactosidase precursor, putative, expressed	DEG uniquely in H21 compared to T45
MSTRG.521	LOC_Os01g08440	UDP-glucoronosyl and UDP-glucosyl transferase domain containing protein, expressed	DEG uniquely in H21 compared to T45
MSTRG.5213	LOC_Os10g35770	E2F-related protein, putative, expressed	DEG uniquely in H21 compared to T45
MSTRG.5226	LOC_Os10g35960		DEG uniquely in H21 compared to T45
MSTRG.5227			DEG uniquely in H21 compared to T45
MSTRG.5248	LOC_Os10g36370	heat shock protein DnaJ, putative, expressed	DEG uniquely in H21 compared to T45
MSTRG.5260	LOC_Os10g36650	actin, putative, expressed	DEG uniquely in H21 compared to T45
MSTRG.5262	LOC_Os10g36703	CPuORF40 - conserved peptide uORF-containing transcript, expressed	DEG uniquely in H21 compared to T45
MSTRG.5275	LOC_Os10g36880	kinesin motor domain containing protein, putative, expressed	DEG uniquely in H21 compared to T45
MSTRG.5289	LOC_Os10g37260	fringe-related protein, putative, expressed	DEG uniquely in H21 compared to T45
MSTRG.5299	LOC_Os10g37500	AAA-type ATPase family protein, putative, expressed	DEG uniquely in H21 compared to T45
MSTRG.5370	LOC_Os10g38489		DEG uniquely in H21 compared to T45
MSTRG.5391	LOC_Os10g39120	ubiquitin-conjugating enzyme, putative, expressed	DEG uniquely in H21 compared to T45
MSTRG.5393	LOC_Os10g39140	flavonol synthase/flavanone 3-hydroxylase, putative, expressed	DEG uniquely in H21 compared to T45
MSTRG.5398	LOC_Os10g39410	Core histone H2A/H2B/H3/H4 domain containing protein, putative, expressed	DEG uniquely in H21 compared to T45
MSTRG.5458	LOC_Os10g40130	Mur ligase family protein, putative, expressed	DEG uniquely in H21 compared to T45
MSTRG.5496			DEG uniquely in H21 compared to T45
MSTRG.5520	LOC_Os10g41110	autophagy-related protein 3, putative, expressed	DEG uniquely in H21 compared to T45
MSTRG.5533	LOC_Os10g41260	MYB family transcription factor, putative, expressed	DEG uniquely in H21 compared to T45
MSTRG.558	LOC_Os01g08970	SSRP1-like FACT complex subunit, putative, expressed	DEG uniquely in H21 compared to T45
MSTRG.5592	LOC_Os10g42066	expressed protein	DEG uniquely in H21 compared to T45
MSTRG.5595	LOC_Os10g42110	protein kinase family protein, putative, expressed	DEG uniquely in H21 compared to T45
MSTRG.5599	LOC_Os10g42160		DEG uniquely in H21 compared to T45
MSTRG.562	LOC_Os01g09010	transferase family protein, putative, expressed	DEG uniquely in H21 compared to T45
MSTRG.5668	LOC_Os11g01010	autophagy-related protein 8D, putative, expressed	DEG uniquely in H21 compared to T45
MSTRG.5677	LOC_Os11g01200	senescence-induced receptor-like serine/threonine-protein kinase precursor, putative, expressed	DEG uniquely in H21 compared to T45
MSTRG.5680	LOC_Os11g01220	expressed protein	DEG uniquely in H21 compared to T45
MSTRG.5690	LOC_Os11g01330	expressed protein	DEG uniquely in H21 compared to T45
MSTRG.5729	LOC_Os11g01990		DEG uniquely in H21 compared to T45
MSTRG.5785	LOC_Os11g02720		DEG uniquely in H21 compared to T45
MSTRG.5796	LOC_Os11g02820	CRP10 - Cysteine-rich family protein precursor, expressed	DEG uniquely in H21 compared to T45
MSTRG.5843	LOC_Os11g03470	bifunctional aspartokinase/homoserine dehydrogenase, putative, expressed	DEG uniquely in H21 compared to T45
MSTRG.5878	LOC_Os11g04104	major facilitator superfamily antiporter, putative, expressed	DEG uniquely in H21 compared to T45
MSTRG.5935			DEG uniquely in H21 compared to T45
MSTRG.6031			DEG uniquely in H21 compared to T45
MSTRG.6042	LOC_Os11g06650	transcription elongation factor protein, putative, expressed	DEG uniquely in H21 compared to T45
MSTRG.6043	LOC_Os11g06670	expressed protein	DEG uniquely in H21 compared to T45
MSTRG.6064	LOC_Os11g07040	CAMK_CAMK_like.43 - CAMK includes calcium/calmodulin depedent protein kinases, expressed	DEG uniquely in H21 compared to T45
MSTRG.6086	LOC_Os11g07450	zinc finger, C3HC4 type domain containing protein, expressed	DEG uniquely in H21 compared to T45
MSTRG.6115	LOC_Os11g08020	ankyrin repeat family protein, putative, expressed	DEG uniquely in H21 compared to T45
MSTRG.6129	LOC_Os11g08380	1-aminocyclopropane-1-carboxylate oxidase, putative, expressed	DEG uniquely in H21 compared to T45
MSTRG.6165	LOC_Os11g09370;LOC_Os11g09375	histone deacetylase 19, putative, expressed;expressed protein	DEG uniquely in H21 compared to T45
MSTRG.6315	LOC_Os11g12420	serpin domain containing protein, putative, expressed	DEG uniquely in H21 compared to T45
MSTRG.6492	LOC_Os11g16590	ATP-dependent Clp protease ATP-binding subunit clpA homolog CD4B,chloroplast precursor, putative, expressed	DEG uniquely in H21 compared to T45

MSTRG.6531	LOC_Os11g19140	methyltransferase domain containing protein, expressed	DEG uniquely in H21 compared to T45
MSTRG.6541			DEG uniquely in H21 compared to T45
MSTRG.6561			DEG uniquely in H21 compared to T45
MSTRG.6574	LOC_Os11g20710		DEG uniquely in H21 compared to T45
MSTRG.6630	LOC_Os11g24450;LOC_Os11g25030	mitochondrial carrier protein, putative, expressed;mitochondrial 2-oxoglutarate/malate translocator, putative, expressed	DEG uniquely in H21 compared to T45
MSTRG.6676	LOC_Os11g26594	expressed protein	DEG uniquely in H21 compared to T45
MSTRG.6706	LOC_Os11g27795	expressed protein	DEG uniquely in H21 compared to T45
MSTRG.6741	LOC_Os11g29370	haloacid dehalogenase-like hydrolase family protein, putative, expressed	DEG uniquely in H21 compared to T45
MSTRG.6791	LOC_Os11g31190	nodulin MtN3 family protein, putative, expressed	DEG uniquely in H21 compared to T45
MSTRG.6821	LOC_Os11g31705;LOC_Os11g31700	expressed protein	DEG uniquely in H21 compared to T45
MSTRG.684	LOC_Os01g11070	Alg9-like mannosyltransferase protein, putative, expressed	DEG uniquely in H21 compared to T45
MSTRG.687	LOC_Os01g11150	gibberellin 2-beta-dioxygenase, putative, expressed	DEG uniquely in H21 compared to T45
MSTRG.6877	LOC_Os11g33190	OsFBX422 - F-box domain containing protein, expressed	DEG uniquely in H21 compared to T45
MSTRG.6971			DEG uniquely in H21 compared to T45
MSTRG.6989			DEG uniquely in H21 compared to T45
MSTRG.6991	LOC_Os11g36390	RFC1 - Putative clamp loader of PCNA, replication factor C subunit 1, expressed	DEG uniquely in H21 compared to T45
MSTRG.7003	LOC_Os11g36670	expressed protein	DEG uniquely in H21 compared to T45
MSTRG.7011	LOC_Os11g36840	expressed protein	DEG uniquely in H21 compared to T45
MSTRG.7024	LOC_Os11g37080	h/ACA ribonucleoprotein complex subunit 1-like protein 1, putative, expressed	DEG uniquely in H21 compared to T45
MSTRG.7034			DEG uniquely in H21 compared to T45
MSTRG.7069	LOC_Os11g37740	stripe rust resistance protein Yr10, putative, expressed	DEG uniquely in H21 compared to T45
MSTRG.7080	LOC_Os11g37970	WIP5 - Wound-induced protein precursor, expressed	DEG uniquely in H21 compared to T45
MSTRG.7113			DEG uniquely in H21 compared to T45
MSTRG.7198	LOC_Os11g39568	expressed protein	DEG uniquely in H21 compared to T45
MSTRG.7206	LOC_Os11g39630		DEG uniquely in H21 compared to T45
MSTRG.7215	LOC_Os11g39810		DEG uniquely in H21 compared to T45
MSTRG.7226	LOC_Os11g40110	RWP-RK, putative, expressed	DEG uniquely in H21 compared to T45
MSTRG.7229	LOC_Os11g40160	expressed protein	DEG uniquely in H21 compared to T45
MSTRG.7253	LOC_Os11g40500	OsSigP7 - Putative Type I Signal Peptidase homologue; employs a putative Ser/Lys catalytic dyad, expressed	DEG uniquely in H21 compared to T45
MSTRG.726	LOC_Os01g11850		DEG uniquely in H21 compared to T45
MSTRG.7265			DEG uniquely in H21 compared to T45
MSTRG.7268	LOC_Os11g40760;LOC_Os11g40770	expressed protein	DEG uniquely in H21 compared to T45
MSTRG.73	LOC_Os01g02050	phosphoenolpyruvate carboxylase, putative, expressed	DEG uniquely in H21 compared to T45
MSTRG.7360	LOC_Os11g42570		DEG uniquely in H21 compared to T45
MSTRG.7386	LOC_Os11g42800	kinesin motor domain containing protein, putative, expressed	DEG uniquely in H21 compared to T45
MSTRG.7397	LOC_Os11g43320		DEG uniquely in H21 compared to T45
MSTRG.7408	LOC_Os11g43590	kelch repeat protein, putative, expressed	DEG uniquely in H21 compared to T45
MSTRG.7445	LOC_Os11g44420		DEG uniquely in H21 compared to T45
MSTRG.746	LOC_Os01g12160	OsGH3.3 - Probable indole-3-acetic acid-amido synthetase, expressed	DEG uniquely in H21 compared to T45
MSTRG.7473	LOC_Os11g45050	NBS-LRR disease resistance protein, putative, expressed	DEG uniquely in H21 compared to T45
MSTRG.756	LOC_Os01g12220	uncharacterized protein family UPF0016 domain containing protein, expressed	DEG uniquely in H21 compared to T45
MSTRG.7570	LOC_Os11g47809	metallothionein, putative, expressed	DEG uniquely in H21 compared to T45
MSTRG.7581	LOC_Os11g48020	fatty acid hydroxylase, putative, expressed	DEG uniquely in H21 compared to T45
MSTRG.7583	LOC_Os11g47970	AAA-type ATPase family protein, putative, expressed	DEG uniquely in H21 compared to T45
MSTRG.7594	LOC_Os11g48060	laccase-22 precursor, putative, expressed	DEG uniquely in H21 compared to T45
MSTRG.7631	LOC_Os12g01530		DEG uniquely in H21 compared to T45
MSTRG.7632	LOC_Os12g01560	PMR5, putative, expressed	DEG uniquely in H21 compared to T45
MSTRG.7683	LOC_Os12g02390;LOC_Os12g02385	outer membrane protein, OMP85 family, putative, expressed;vacuolar-sorting receptor precursor, putative, expressed	DEG uniquely in H21 compared to T45
MSTRG.7685	LOC_Os12g02440	WRKY95, expressed	DEG uniquely in H21 compared to T45
MSTRG.7691	LOC_Os12g02540	BTBN23 - Bric-a-Brac, Tramtrack, Broad Complex BTB domain with non-phototropic hypocotyl 3 NPH3 and coiled-coil domains, expressed	DEG uniquely in H21 compared to T45
MSTRG.7783	LOC_Os12g03870	major facilitator superfamily antiporter, putative, expressed	DEG uniquely in H21 compared to T45
MSTRG.7804	LOC_Os12g04200		DEG uniquely in H21 compared to T45
MSTRG.7851	LOC_Os12g05050	stem-specific protein TSJT1, putative, expressed	DEG uniquely in H21 compared to T45
MSTRG.7899			DEG uniquely in H21 compared to T45
MSTRG.7920	LOC_Os12g06220	harpin-induced protein 1 domain containing protein, expressed	DEG uniquely in H21 compared to T45
MSTRG.7936	LOC_Os12g06510	calcineurin B, putative, expressed	DEG uniquely in H21 compared to T45
MSTRG.795	LOC_Os01g12840	expressed protein	DEG uniquely in H21 compared to T45

MSTRG.8026	LOC_Os12g07840	dehydration response related protein, putative, expressed	DEG uniquely in H21 compared to T45
MSTRG.8027	LOC_Os12g07874;LOC_Os12g07880	WD-40 repeat family protein, putative, expressed;dynamin family protein, putative, expressed	DEG uniquely in H21 compared to T45
MSTRG.8067	LOC_Os12g08730	thioredoxin, putative, expressed	DEG uniquely in H21 compared to T45
MSTRG.8071	LOC_Os12g08760	carboxyvinyl-carboxyphosphonate phosphorylmutase, putative, expressed	DEG uniquely in H21 compared to T45
MSTRG.8074	LOC_Os12g08810	VTC2, putative, expressed	DEG uniquely in H21 compared to T45
MSTRG.8108	LOC_Os12g09660	expressed protein	DEG uniquely in H21 compared to T45
MSTRG.8210	LOC_Os12g12580	NADP-dependent oxidoreductase, putative, expressed	DEG uniquely in H21 compared to T45
MSTRG.8231			DEG uniquely in H21 compared to T45
MSTRG.825	LOC_Os01g13260	cyclin-A1, putative, expressed	DEG uniquely in H21 compared to T45
MSTRG.8309	LOC_Os12g15314	staphylococcal nuclease homologue, putative, expressed	DEG uniquely in H21 compared to T45
MSTRG.8359	LOC_Os12g17120	retrotransposon protein, putative, Ty1-copia subclass, expressed	DEG uniquely in H21 compared to T45
MSTRG.8380	LOC_Os12g17600	ribulose bisphosphate carboxylase small chain, chloroplast precursor, putative, expressed	DEG uniquely in H21 compared to T45
MSTRG.8384			DEG uniquely in H21 compared to T45
MSTRG.849	LOC_Os01g13550	beta-amylase, putative, expressed	DEG uniquely in H21 compared to T45
MSTRG.8503	LOC_Os12g21789	expressed protein	DEG uniquely in H21 compared to T45
MSTRG.8509	LOC_Os12g21880		DEG uniquely in H21 compared to T45
MSTRG.8566	LOC_Os12g23450	expressed protein	DEG uniquely in H21 compared to T45
MSTRG.859	LOC_Os01g13730	WD domain, G-beta repeat domain containing protein, expressed	DEG uniquely in H21 compared to T45
MSTRG.8605	LOC_Os12g24500	expressed protein	DEG uniquely in H21 compared to T45
MSTRG.8608	LOC_Os12g24550	expressed protein	DEG uniquely in H21 compared to T45
MSTRG.8643	LOC_Os12g25720	expressed protein	DEG uniquely in H21 compared to T45
MSTRG.8719	LOC_Os12g29220	nodulin MtN3 family protein, putative, expressed	DEG uniquely in H21 compared to T45
MSTRG.8759	LOC_Os12g29990;LOC_Os12g30000	O-sialoglycoprotein endopeptidase, putative, expressed;expressed protein	DEG uniquely in H21 compared to T45
MSTRG.882	LOC_Os01g14090	kinesin motor domain containing protein, putative, expressed	DEG uniquely in H21 compared to T45
MSTRG.8824	LOC_Os12g31810	cyclin, putative, expressed	DEG uniquely in H21 compared to T45
MSTRG.884	LOC_Os01g14170		DEG uniquely in H21 compared to T45
MSTRG.889	LOC_Os01g14310		DEG uniquely in H21 compared to T45
MSTRG.8903	LOC_Os12g33230	OsFBL60 - F-box domain and LRR containing protein, expressed	DEG uniquely in H21 compared to T45
MSTRG.8956	LOC_Os12g34510	Core histone H2A/H2B/H3/H4 domain containing protein, putative, expressed	DEG uniquely in H21 compared to T45
MSTRG.8972	LOC_Os12g34840	rhoGAP domain containing protein, expressed	DEG uniquely in H21 compared to T45
MSTRG.8984	LOC_Os12g34920	csAtPR5, putative, expressed	DEG uniquely in H21 compared to T45
MSTRG.9008	LOC_Os12g35480	expressed protein	DEG uniquely in H21 compared to T45
MSTRG.9049			DEG uniquely in H21 compared to T45
MSTRG.9171	LOC_Os12g38150	osmotin, putative, expressed	DEG uniquely in H21 compared to T45
MSTRG.9197	LOC_Os12g38730	expressed protein	DEG uniquely in H21 compared to T45
MSTRG.9210	LOC_Os12g38920	heparanase-like protein precursor, putative, expressed	DEG uniquely in H21 compared to T45
MSTRG.9232	LOC_Os12g39320	DUF221 domain containing protein, expressed	DEG uniquely in H21 compared to T45
MSTRG.9275	LOC_Os12g39650	tubulin, putative, expressed	DEG uniquely in H21 compared to T45
MSTRG.9288	LOC_Os12g39920	expressed protein	DEG uniquely in H21 compared to T45
MSTRG.9337	LOC_Os12g40490	LIM domain-containing protein, putative, expressed	DEG uniquely in H21 compared to T45
MSTRG.9339	LOC_Os12g40510	calcineurin B, putative, expressed	DEG uniquely in H21 compared to T45
MSTRG.9352	LOC_Os12g40860	Leucine Rich Repeat family protein, expressed	DEG uniquely in H21 compared to T45
MSTRG.944			DEG uniquely in H21 compared to T45
MSTRG.9498	LOC_Os12g42840	expressed protein	DEG uniquely in H21 compared to T45
MSTRG.9555	LOC_Os12g43890	GNS1/SUR4 membrane family protein, putative, expressed	DEG uniquely in H21 compared to T45
MSTRG.9568	LOC_Os12g44150	plasma membrane ATPase, putative, expressed	DEG uniquely in H21 compared to T45
MSTRG.9582	LOC_Os12g44370	expressed protein	DEG uniquely in H21 compared to T45
MSTRG.9605	LOC_Os02g01280	T-complex protein, putative, expressed	DEG uniquely in H21 compared to T45
MSTRG.9693	LOC_Os02g02500	remorin family protein, putative, expressed	DEG uniquely in H21 compared to T45
MSTRG.9768	LOC_Os02g03330	expressed protein	DEG uniquely in H21 compared to T45
MSTRG.9888	LOC_Os02g04750	cycloartenol synthase, putative, expressed	DEG uniquely in H21 compared to T45
MSTRG.9893	LOC_Os02g04840	GHMP kinases ATP-binding protein, putative, expressed	DEG uniquely in H21 compared to T45
MSTRG.9898	LOC_Os02g05470	CCT motif family protein, expressed	DEG uniquely in H21 compared to T45
MSTRG.9910	LOC_Os02g05610	ZOS2-03 - C2H2 zinc finger protein, expressed	DEG uniquely in H21 compared to T45
MSTRG.9928	LOC_Os02g04970;LOC_Os02g05020	expressed protein	DEG uniquely in H21 compared to T45
MSTRG.9966	LOC_Os02g05300		DEG uniquely in H21 compared to T45
MSTRG.10007	LOC_Os02g06010	integral membrane protein, putative, expressed	DEG common in H21 compared to T44 and T45
MSTRG.10018	LOC_Os02g06170	expressed protein	DEG common in H21 compared to T44 and T45
MSTRG.10109	LOC_Os02g07260		DEG common in H21 compared to T44 and T45
MSTRG.10267	LOC_Os02g10170	expressed protein	DEG common in H21 compared to T44 and T45
MSTRG.10381	LOC_Os02g12350	histone deacetylase, putative, expressed	DEG common in H21 compared to T44 and T45
MSTRG.1054			DEG common in H21 compared to T44 and T45
MSTRG.10864			DEG common in H21 compared to T44 and T45
MSTRG.1098	LOC_Os01g18230		DEG common in H21 compared to T44 and T45
MSTRG.11074	LOC_Os02g28030	expressed protein	DEG common in H21 compared to T44 and T45
MSTRG.11077	LOC_Os02g28074		DEG common in H21 compared to T44 and T45
MSTRG.11098			DEG common in H21 compared to T44 and T45
MSTRG.11168	LOC_Os02g30114	expressed protein	DEG common in H21 compared to T44 and T45

MSTRG.1119	LOC_Os01g18800	CAMK_KIN1/SNF1/Nim1_like.9 - CAMK includes calcium/calmodulin depedent protein kinases, expressed	DEG common in H21 compared to T44 and T45
MSTRG.11211	LOC_Os02g30974	expressed protein	DEG common in H21 compared to T44 and T45
MSTRG.11274			DEG common in H21 compared to T44 and T45
MSTRG.1134	LOC_Os01g19320		DEG common in H21 compared to T44 and T45
MSTRG.11353	LOC_Os02g34120		DEG common in H21 compared to T44 and T45
MSTRG.11355	LOC_Os02g34190	expressed protein	DEG common in H21 compared to T44 and T45
MSTRG.11356			DEG common in H21 compared to T44 and T45
MSTRG.11388	LOC_Os02g34740	retrotransposon protein, putative, unclassified, expressed	DEG common in H21 compared to T44 and T45
MSTRG.11468	LOC_Os02g35980		DEG common in H21 compared to T44 and T45
MSTRG.12315	LOC_Os02g49510		DEG common in H21 compared to T44 and T45
MSTRG.12384	LOC_Os02g50470	expressed protein	DEG common in H21 compared to T44 and T45
MSTRG.12715	LOC_Os02g55400	ATPase 8, plasma membrane-type, putative, expressed	DEG common in H21 compared to T44 and T45
MSTRG.12736			DEG common in H21 compared to T44 and T45
MSTRG.12915	LOC_Os02g57860;LOC_Os02g57960	OsFBX71 - F-box domain containing protein, expressed;Leucine Rich Repeat family protein, expressed	DEG common in H21 compared to T44 and T45
MSTRG.12918			DEG common in H21 compared to T44 and T45
MSTRG.12921	LOC_Os02g57900	expressed protein	DEG common in H21 compared to T44 and T45
MSTRG.12922	LOC_Os02g57910	OsFBX73 - F-box domain containing protein, expressed	DEG common in H21 compared to T44 and T45
MSTRG.12924	LOC_Os02g57924	expressed protein	DEG common in H21 compared to T44 and T45
MSTRG.12926	LOC_Os02g57940	OsFBX74 - F-box domain containing protein, expressed	DEG common in H21 compared to T44 and T45
MSTRG.13119	LOC_Os03g02710	hydroxymethylglutaryl-CoA synthase, putative, expressed	DEG common in H21 compared to T44 and T45
MSTRG.13743	LOC_Os03g11420	Os3bglu6 - beta-glucosidase/beta-fucosidase/beta-galactosidase, expressed	DEG common in H21 compared to T44 and T45
MSTRG.13776			DEG common in H21 compared to T44 and T45
MSTRG.13779	LOC_Os03g11810		DEG common in H21 compared to T44 and T45
MSTRG.13873	LOC_Os03g13200		DEG common in H21 compared to T44 and T45
MSTRG.13927	LOC_Os03g13976	expressed protein	DEG common in H21 compared to T44 and T45
MSTRG.14165	LOC_Os03g17410	expressed protein	DEG common in H21 compared to T44 and T45
MSTRG.1436			DEG common in H21 compared to T44 and T45
MSTRG.1440			DEG common in H21 compared to T44 and T45
MSTRG.14706	LOC_Os03g26229	40S ribosomal protein S9-1, putative, expressed	DEG common in H21 compared to T44 and T45
MSTRG.14709	LOC_Os03g26350;LOC_Os03g26360	transposon protein, putative, CACTA, En/Spm sub-class, expressed;expressed protein	DEG common in H21 compared to T44 and T45
MSTRG.15092			DEG common in H21 compared to T44 and T45
MSTRG.15128	LOC_Os03g39920	retrotransposon protein, putative, unclassified, expressed	DEG common in H21 compared to T44 and T45
MSTRG.15177			DEG common in H21 compared to T44 and T45
MSTRG.15186	LOC_Os03g41120	expressed protein	DEG common in H21 compared to T44 and T45
MSTRG.15189	LOC_Os03g41200	retrotransposon protein, putative, unclassified, expressed	DEG common in H21 compared to T44 and T45
MSTRG.15195			DEG common in H21 compared to T44 and T45
MSTRG.15642	LOC_Os03g50110	transcription regulator, putative, expressed	DEG common in H21 compared to T44 and T45
MSTRG.1589	LOC_Os01g33540	transposon protein, putative, unclassified, expressed	DEG common in H21 compared to T44 and T45
MSTRG.16024	LOC_Os03g56220		DEG common in H21 compared to T44 and T45
MSTRG.16301	LOC_Os03g59840	expressed protein	DEG common in H21 compared to T44 and T45
MSTRG.16302	LOC_Os03g59880		DEG common in H21 compared to T44 and T45
MSTRG.16569	LOC_Os03g63670	expressed protein	DEG common in H21 compared to T44 and T45
MSTRG.16727	LOC_Os04g06520		DEG common in H21 compared to T44 and T45
MSTRG.16734			DEG common in H21 compared to T44 and T45
MSTRG.16801			DEG common in H21 compared to T44 and T45
MSTRG.16823			DEG common in H21 compared to T44 and T45
MSTRG.16834			DEG common in H21 compared to T44 and T45
MSTRG.16879	LOC_Os04g10410		DEG common in H21 compared to T44 and T45
MSTRG.16921			DEG common in H21 compared to T44 and T45
MSTRG.16965			DEG common in H21 compared to T44 and T45
MSTRG.16969			DEG common in H21 compared to T44 and T45
MSTRG.16971	LOC_Os04g14210		DEG common in H21 compared to T44 and T45
MSTRG.16982			DEG common in H21 compared to T44 and T45
MSTRG.16983	LOC_Os04g14190;LOC_Os04g14200	expressed protein	DEG common in H21 compared to T44 and T45
MSTRG.16985	LOC_Os04g14200		DEG common in H21 compared to T44 and T45
MSTRG.17002	LOC_Os04g14850	retrotransposon protein, putative, Ty3-gypsy subclass, expressed	DEG common in H21 compared to T44 and T45
MSTRG.17023	LOC_Os04g15670;LOC_Os04g15680	expressed protein	DEG common in H21 compared to T44 and T45
MSTRG.17026	LOC_Os04g15550	expressed protein	DEG common in H21 compared to T44 and T45
MSTRG.17049			DEG common in H21 compared to T44 and T45
MSTRG.17154	LOC_Os04g18530		DEG common in H21 compared to T44 and T45
MSTRG.17232			DEG common in H21 compared to T44 and T45
MSTRG.17313	LOC_Os04g24294	OsWAK35d - OsWAK short gene, expressed	DEG common in H21 compared to T44 and T45
MSTRG.17314	LOC_Os04g24300	OsWAK35a - OsWAK short gene, expressed	DEG common in H21 compared to T44 and T45
MSTRG.17324			DEG common in H21 compared to T44 and T45
MSTRG.17351			DEG common in H21 compared to T44 and T45
MSTRG.17396	LOC_Os04g27800	expressed protein	DEG common in H21 compared to T44 and T45
MSTRG.17436			DEG common in H21 compared to T44 and T45
MSTRG.17487	LOC_Os04g30180	F-box/LRR-repeat protein 14, putative, expressed	DEG common in H21 compared to T44 and T45

MSTRG.17538	LOC_Os04g30800	ER lumen protein retaining receptor, putative, expressed	DEG common in H21 compared to T44 and T45
MSTRG.17625	LOC_Os04g32340	RNA-binding motif protein, putative, expressed	DEG common in H21 compared to T44 and T45
MSTRG.17861	LOC_Os04g35630	expressed protein	DEG common in H21 compared to T44 and T45
MSTRG.17985	LOC_Os04g38430	nodulin, putative, expressed	DEG common in H21 compared to T44 and T45
MSTRG.18251			DEG common in H21 compared to T44 and T45
MSTRG.1847			DEG common in H21 compared to T44 and T45
MSTRG.192	LOC_Os01g04409;LOC_Os01g04440	OsWAK1 - OsWAK receptor-like cytoplasmic kinase OsWAK-RLCK, expressed;expressed protein	DEG common in H21 compared to T44 and T45
MSTRG.19250	LOC_Os04g57550	amine oxidase, flavin-containing, domain containing protein, expressed	DEG common in H21 compared to T44 and T45
MSTRG.19431	LOC_Os05g01240	AML1, putative, expressed	DEG common in H21 compared to T44 and T45
MSTRG.19540	LOC_Os05g02780	glycine-rich protein A3, putative, expressed	DEG common in H21 compared to T44 and T45
MSTRG.19787	LOC_Os05g06710	GDSL-like lipase/acylhydrolase, putative, expressed	DEG common in H21 compared to T44 and T45
MSTRG.19800			DEG common in H21 compared to T44 and T45
MSTRG.19968	LOC_Os05g10560	expressed protein	DEG common in H21 compared to T44 and T45
MSTRG.2005	LOC_Os01g42650	cytochrome c oxidase subunit 5B, mitochondrial precursor, putative, expressed	DEG common in H21 compared to T44 and T45
MSTRG.2043	LOC_Os01g43100	protein phosphatase 2C, putative, expressed	DEG common in H21 compared to T44 and T45
MSTRG.2108	LOC_Os01g44100		DEG common in H21 compared to T44 and T45
MSTRG.21943	LOC_Os06g01770	expressed protein	DEG common in H21 compared to T44 and T45
MSTRG.22639	LOC_Os06g12470	retrotransposon protein, putative, Ty3-gypsy subclass, expressed	DEG common in H21 compared to T44 and T45
MSTRG.22688	LOC_Os06g13230	expressed protein	DEG common in H21 compared to T44 and T45
MSTRG.22857	LOC_Os06g17130		DEG common in H21 compared to T44 and T45
MSTRG.23024			DEG common in H21 compared to T44 and T45
MSTRG.23037	LOC_Os06g22394	expressed protein	DEG common in H21 compared to T44 and T45
MSTRG.23435	LOC_Os06g34830	amino acid permease family protein, putative, expressed	DEG common in H21 compared to T44 and T45
MSTRG.23443	LOC_Os06g35160	CAMK_KIN1/SNF1/Nim1_like.26 - CAMK includes calcium/calmodulin depedent protein kinases, expressed	DEG common in H21 compared to T44 and T45
MSTRG.23448	LOC_Os06g35490	peroxidase precursor, putative, expressed	DEG common in H21 compared to T44 and T45
MSTRG.23691	LOC_Os06g40240	retrotransposon protein, putative, unclassified, expressed	DEG common in H21 compared to T44 and T45
MSTRG.2377	LOC_Os01g49219	expressed protein	DEG common in H21 compared to T44 and T45
MSTRG.23991	LOC_Os06g44034	expressed protein	DEG common in H21 compared to T44 and T45
MSTRG.24074	LOC_Os06g45360	peptidase, M24 family protein, putative, expressed	DEG common in H21 compared to T44 and T45
MSTRG.24171	LOC_Os06g46860		DEG common in H21 compared to T44 and T45
MSTRG.24186	LOC_Os06g47140	expressed protein	DEG common in H21 compared to T44 and T45
MSTRG.24189	LOC_Os06g47150	auxin response factor 18, putative, expressed	DEG common in H21 compared to T44 and T45
MSTRG.24223	LOC_Os06g47600	thaumatin family domain containing protein, expressed	DEG common in H21 compared to T44 and T45
MSTRG.24447	LOC_Os06g50600;LOC_Os06g50679;LOC_Os06g50724	bZIP transcription factor domain containing protein, expressed;expressed protein;START domain containing protein, expressed	DEG common in H21 compared to T44 and T45
MSTRG.24453	LOC_Os06g50670	expressed protein	DEG common in H21 compared to T44 and T45
MSTRG.24504	LOC_Os07g01070	peptide transporter, putative, expressed	DEG common in H21 compared to T44 and T45
MSTRG.24549			DEG common in H21 compared to T44 and T45
MSTRG.24568	LOC_Os07g02330	protein phosphatase 2C, putative, expressed	DEG common in H21 compared to T44 and T45
MSTRG.24600	LOC_Os07g03030	expressed protein	DEG common in H21 compared to T44 and T45
MSTRG.24602	LOC_Os07g03040	expressed protein	DEG common in H21 compared to T44 and T45
MSTRG.24627			DEG common in H21 compared to T44 and T45
MSTRG.24630	LOC_Os07g03760	26S proteasome non-ATPase regulatory subunit 14, putative, expressed	DEG common in H21 compared to T44 and T45
MSTRG.24657	LOC_Os07g04310	expressed protein	DEG common in H21 compared to T44 and T45
MSTRG.24658	LOC_Os07g04330	expressed protein	DEG common in H21 compared to T44 and T45
MSTRG.24678	LOC_Os07g04730	expressed protein	DEG common in H21 compared to T44 and T45
MSTRG.24878			DEG common in H21 compared to T44 and T45
MSTRG.24979	LOC_Os07g09540		DEG common in H21 compared to T44 and T45
MSTRG.25001	LOC_Os07g09814	OsFBX221 - F-box domain containing protein, expressed	DEG common in H21 compared to T44 and T45
MSTRG.25005	LOC_Os07g09914	expressed protein	DEG common in H21 compared to T44 and T45
MSTRG.25006	LOC_Os07g09950	OsFBX222 - F-box domain containing protein, expressed	DEG common in H21 compared to T44 and T45
MSTRG.2560	LOC_Os01g52110	RING finger and CHY zinc finger domain-containing protein 1, putative, expressed	DEG common in H21 compared to T44 and T45
MSTRG.25664	LOC_Os07g30240	mutS family domain IV containing protein, expressed	DEG common in H21 compared to T44 and T45
MSTRG.25733			DEG common in H21 compared to T44 and T45
MSTRG.25810	LOC_Os07g33420	hydroxylase, putative, expressed	DEG common in H21 compared to T44 and T45
MSTRG.25872	LOC_Os07g34840		DEG common in H21 compared to T44 and T45
MSTRG.25873	LOC_Os07g34964		DEG common in H21 compared to T44 and T45
MSTRG.25912	LOC_Os07g35880	beta-amylase, putative, expressed	DEG common in H21 compared to T44 and T45
MSTRG.25917	LOC_Os07g35985	expressed protein	DEG common in H21 compared to T44 and T45
MSTRG.25950	LOC_Os07g36340		DEG common in H21 compared to T44 and T45
MSTRG.25957			DEG common in H21 compared to T44 and T45
MSTRG.25967			DEG common in H21 compared to T44 and T45
MSTRG.26134	LOC_Os07g39060		DEG common in H21 compared to T44 and T45
MSTRG.26466	LOC_Os07g44770	expressed protein	DEG common in H21 compared to T44 and T45
MSTRG.26720	LOC_Os07g48490	stress responsive protein, putative, expressed	DEG common in H21 compared to T44 and T45
MSTRG.26726	LOC_Os07g48520		DEG common in H21 compared to T44 and T45
MSTRG.27136	LOC_Os08g06659	expressed protein	DEG common in H21 compared to T44 and T45
MSTRG.27180	LOC_Os08g07900		DEG common in H21 compared to T44 and T45

MSTRG.27470			DEG common in H21 compared to T44 and T45
MSTRG.27500			DEG common in H21 compared to T44 and T45
MSTRG.27565	LOC_Os08g15450	nodulin, putative, expressed	DEG common in H21 compared to T44 and T45
MSTRG.27609	LOC_Os08g16740	expressed protein	DEG common in H21 compared to T44 and T45
MSTRG.27888	LOC_Os08g25710		DEG common in H21 compared to T44 and T45
MSTRG.28038	LOC_Os08g29854	RGH1A, putative, expressed	DEG common in H21 compared to T44 and T45
MSTRG.28045	LOC_Os08g30020		DEG common in H21 compared to T44 and T45
MSTRG.28100	LOC_Os08g31130	integral membrane protein DUF6 containing protein, expressed	DEG common in H21 compared to T44 and T45
MSTRG.28402	LOC_Os08g36570	F-box protein, putative, expressed	DEG common in H21 compared to T44 and T45
MSTRG.28524	LOC_Os08g38900		DEG common in H21 compared to T44 and T45
MSTRG.28556	LOC_Os08g39330	skin secretory protein xP2 precursor, putative, expressed	DEG common in H21 compared to T44 and T45
MSTRG.28668	LOC_Os08g41190		DEG common in H21 compared to T44 and T45
MSTRG.28691	LOC_Os08g41489		DEG common in H21 compared to T44 and T45
MSTRG.28742	LOC_Os08g42210	expressed protein	DEG common in H21 compared to T44 and T45
MSTRG.29372	LOC_Os09g14520	integral membrane protein, putative, expressed	DEG common in H21 compared to T44 and T45
MSTRG.29877	LOC_Os09g27350		DEG common in H21 compared to T44 and T45
MSTRG.3245	LOC_Os01g61940	white-brown complex homolog protein, putative, expressed	DEG common in H21 compared to T44 and T45
MSTRG.3641			DEG common in H21 compared to T44 and T45
MSTRG.4133	LOC_Os10g03620	OsFBX344 - F-box domain containing protein, expressed	DEG common in H21 compared to T44 and T45
MSTRG.4329	LOC_Os10g09320		DEG common in H21 compared to T44 and T45
MSTRG.4397	LOC_Os10g11870	transposon protein, putative, CACTA, En/Spm sub-class, expressed	DEG common in H21 compared to T44 and T45
MSTRG.4627	LOC_Os10g22570	cellulase, putative, expressed	DEG common in H21 compared to T44 and T45
MSTRG.4642	LOC_Os10g22830		DEG common in H21 compared to T44 and T45
MSTRG.4703			DEG common in H21 compared to T44 and T45
MSTRG.4879	LOC_Os10g29650	retrotransposon protein, putative, unclassified, expressed	DEG common in H21 compared to T44 and T45
MSTRG.4884	LOC_Os10g29700	expressed protein	DEG common in H21 compared to T44 and T45
MSTRG.4939	LOC_Os10g30970	expressed protein	DEG common in H21 compared to T44 and T45
MSTRG.4950			DEG common in H21 compared to T44 and T45
MSTRG.5117	LOC_Os10g34680		DEG common in H21 compared to T44 and T45
MSTRG.5153	LOC_Os10g35180	white-brown complex homolog protein 11, putative, expressed	DEG common in H21 compared to T44 and T45
MSTRG.5376	LOC_Os10g38890	expressed protein	DEG common in H21 compared to T44 and T45
MSTRG.5392	LOC_Os10g39130	OsMADS56 - MADS-box family gene with MIKCc type-box, expressed	DEG common in H21 compared to T44 and T45
MSTRG.5406	LOC_Os10g39460;LOC_Os10g39470	expressed protein	DEG common in H21 compared to T44 and T45
MSTRG.5497	LOC_Os10g40770		DEG common in H21 compared to T44 and T45
MSTRG.5672	LOC_Os11g01154	dehydrogenase, putative, expressed	DEG common in H21 compared to T44 and T45
MSTRG.582	LOC_Os01g09384	ankyrin repeat domain containing protein, putative, expressed	DEG common in H21 compared to T44 and T45
MSTRG.584	LOC_Os01g09410	expressed protein	DEG common in H21 compared to T44 and T45
MSTRG.6198	LOC_Os11g09864	wali7, putative, expressed	DEG common in H21 compared to T44 and T45
MSTRG.633	LOC_Os01g10130;LOC_Os01g10140	RNA-dependent RNA polymerase, putative, expressed	DEG common in H21 compared to T44 and T45
MSTRG.6425			DEG common in H21 compared to T44 and T45
MSTRG.6447			DEG common in H21 compared to T44 and T45
MSTRG.6952			DEG common in H21 compared to T44 and T45
MSTRG.7039	LOC_Os11g37480		DEG common in H21 compared to T44 and T45
MSTRG.7124	LOC_Os11g38690		DEG common in H21 compared to T44 and T45
MSTRG.7143	LOC_Os11g38810	mannose-6-phosphate isomerase, putative, expressed	DEG common in H21 compared to T44 and T45
MSTRG.7298	LOC_Os11g41380	transposon protein, putative, unclassified, expressed	DEG common in H21 compared to T44 and T45
MSTRG.7527	LOC_Os11g47120	DEFL48 - Defensin and Defensin-like DEFL family, expressed	DEG common in H21 compared to T44 and T45
MSTRG.7609	LOC_Os12g01260	protein transport protein Sec61 subunit beta, putative, expressed	DEG common in H21 compared to T44 and T45
MSTRG.7616	LOC_Os12g01290		DEG common in H21 compared to T44 and T45
MSTRG.7670			DEG common in H21 compared to T44 and T45
MSTRG.7730	LOC_Os12g03080	rp3 protein, putative, expressed	DEG common in H21 compared to T44 and T45
MSTRG.7847	LOC_Os12g05030		DEG common in H21 compared to T44 and T45
MSTRG.7961	LOC_Os12g06900		DEG common in H21 compared to T44 and T45
MSTRG.8248			DEG common in H21 compared to T44 and T45
MSTRG.8310	LOC_Os12g15340	retrotransposon protein, putative, LINE subclass, expressed	DEG common in H21 compared to T44 and T45
MSTRG.8371			DEG common in H21 compared to T44 and T45
MSTRG.8474	LOC_Os12g20324	cyclin-A1, putative, expressed	DEG common in H21 compared to T44 and T45
MSTRG.8477	LOC_Os12g20390	expressed protein	DEG common in H21 compared to T44 and T45
MSTRG.8483	LOC_Os12g20410	matrix attachment region binding protein, putative, expressed	DEG common in H21 compared to T44 and T45
MSTRG.8518	LOC_Os12g22020		DEG common in H21 compared to T44 and T45
MSTRG.8683	LOC_Os12g26540		DEG common in H21 compared to T44 and T45
MSTRG.8699	LOC_Os12g27102	glycerophosphoryl diester phosphodiesterase family protein, putative, expressed	DEG common in H21 compared to T44 and T45
MSTRG.8720	LOC_Os12g29280		DEG common in H21 compared to T44 and T45
MSTRG.8773	LOC_Os12g30320	expressed protein	DEG common in H21 compared to T44 and T45
MSTRG.8799			DEG common in H21 compared to T44 and T45
MSTRG.8803			DEG common in H21 compared to T44 and T45
MSTRG.8941			DEG common in H21 compared to T44 and T45
MSTRG.9365	LOC_Os12g41140	expressed protein	DEG common in H21 compared to T44 and T45
MSTRG.9659	LOC_Os02g02070		DEG common in H21 compared to T44 and T45

MSTRG.9882	LOC_Os02g04710	cycloartenol synthase, putative, expressed	DEG common in H21 compared to T44 and T45
MSTRG.9894	LOC_Os02g04924;LOC_Os02g04915	expressed protein	DEG common in H21 compared to T44 and T45
MSTRG.9976	LOC_Os02g05420	expressed protein	DEG common in H21 compared to T44 and T45
MSTRG.9978	LOC_Os02g05810	expressed protein	DEG common in H21 compared to T44 and T45
MSTRG.9979	LOC_Os02g05790	expressed protein	DEG common in H21 compared to T44 and T45
MSTRG.9994	LOC_Os02g05940		DEG common in H21 compared to T44 and T45

Table S7. The non-additive genes in this study.

Gene id	Gene name	Gene annotation	Classification
MSTRG.7609	LOC_Os12g01260	protein transport protein Sec61 subunit beta, putative, expressed	The expression level of H21 significantly higher than both parents
MSTRG.8310	LOC_Os12g15340	retrotransposon protein, putative, LINE subclass, expressed	The expression level of H21 significantly higher than both parents
MSTRG.17049			The expression level of H21 significantly higher than both parents
MSTRG.12924	LOC_Os02g57924	expressed protein	The expression level of H21 significantly higher than both parents
MSTRG.5406	LOC_Os10g39460; LOC_Os10g39470	expressed protein	The expression level of H21 significantly higher than both parents
MSTRG.22688	LOC_Os06g13230	expressed protein	The expression level of H21 significantly higher than both parents
MSTRG.23448	LOC_Os06g35490	peroxidase precursor, putative, expressed	The expression level of H21 significantly higher than both parents
MSTRG.4879	LOC_Os10g29650	retrotransposon protein, putative, unclassified, expressed	The expression level of H21 significantly higher than both parents
MSTRG.7616	LOC_Os12g01290		The expression level of H21 significantly higher than both parents
MSTRG.16834			The expression level of H21 significantly higher than both parents
MSTRG.22857	LOC_Os06g17130		The expression level of H21 significantly higher than both parents
MSTRG.11355	LOC_Os02g34190	expressed protein	The expression level of H21 significantly higher than both parents
MSTRG.4950			The expression level of H21 significantly higher than both parents
MSTRG.10018	LOC_Os02g06170	expressed protein	The expression level of H21 significantly higher than both parents
MSTRG.25872	LOC_Os07g34840		The expression level of H21 significantly higher than both parents
MSTRG.23691	LOC_Os06g40240	retrotransposon protein, putative, unclassified, expressed	The expression level of H21 significantly higher than both parents
MSTRG.17351			The expression level of H21 significantly higher than both parents
MSTRG.16301	LOC_Os03g59840	expressed protein	The expression level of H21 significantly higher than both parents
MSTRG.1440			The expression level of H21 significantly higher than both parents
MSTRG.9979	LOC_Os02g05790	expressed protein	The expression level of H21 significantly higher than both parents
MSTRG.27500			The expression level of H21 significantly higher than both parents
MSTRG.10267	LOC_Os02g10170	expressed protein	The expression level of H21 significantly higher than both parents
MSTRG.15189	LOC_Os03g41200	retrotransposon protein, putative, unclassified, expressed	The expression level of H21 significantly higher than both parents
MSTRG.24957	LOC_Os07g09110	OsFBX219 - F-box domain containing protein, expressed	The expression level of H21 significantly higher than both parents
MSTRG.1847			The expression level of H21 significantly higher than both parents
MSTRG.11098			The expression level of H21 significantly higher than both parents
MSTRG.24630	LOC_Os07g03760	26S proteasome non-ATPase regulatory subunit 14, putative, expressed	The expression level of H21 significantly higher than both parents
MSTRG.28524	LOC_Os08g38900		The expression level of H21 significantly higher than both parents
MSTRG.2108	LOC_Os01g44100		The expression level of H21 significantly higher than both parents
MSTRG.17396	LOC_Os04g27800	expressed protein	The expression level of H21 significantly higher than both parents
MSTRG.7847	LOC_Os12g05030		The expression level of H21 significantly higher than both parents
MSTRG.23435	LOC_Os06g34830	amino acid permease family protein, putative, expressed	The expression level of H21 significantly higher than both parents
MSTRG.25005	LOC_Os07g09914	expressed protein	The expression level of H21 significantly higher than both parents
MSTRG.28691	LOC_Os08g41489		The expression level of H21 significantly higher than both parents
MSTRG.1054			The expression level of H21 significantly higher than both parents
MSTRG.1650	LOC_Os01g35110		The expression level of H21 significantly higher than both parents
MSTRG.10864			The expression level of H21 significantly higher than both parents
MSTRG.15128	LOC_Os03g39920	retrotransposon protein, putative, unclassified, expressed	The expression level of H21 significantly higher than both parents
MSTRG.584	LOC_Os01g09410	expressed protein	The expression level of H21 significantly higher than both parents
MSTRG.13779	LOC_Os03g11810		The expression level of H21 significantly higher than both parents
MSTRG.24979	LOC_Os07g09540		The expression level of H21 significantly higher than both parents
MSTRG.13776			The expression level of H21 significantly higher than both parents
MSTRG.12926	LOC_Os02g57940	OsFBX74 - F-box domain containing protein, expressed	The expression level of H21 significantly higher than both parents
MSTRG.7670			The expression level of H21 significantly higher than both parents
MSTRG.582	LOC_Os01g09384	ankyrin repeat domain containing protein, putative, expressed	The expression level of H21 significantly higher than both parents
MSTRG.12922	LOC_Os02g57910	OsFBX73 - F-box domain containing protein, expressed	The expression level of H21 significantly higher than both parents
MSTRG.1134	LOC_Os01g19320		The expression level of H21 significantly higher than both parents
MSTRG.9978	LOC_Os02g05810	expressed protein	The expression level of H21 significantly higher than both parents
MSTRG.17769	LOC_Os04g34530	integral membrane protein DUF6 containing protein, expressed	The expression level of H21 significantly higher than both parents
MSTRG.16965			The expression level of H21 significantly higher than both parents
MSTRG.8371			The expression level of H21 significantly higher than both parents
MSTRG.17232			The expression level of H21 significantly higher than both parents
MSTRG.14706	LOC_Os03g26229	40S ribosomal protein S9-1, putative, expressed	The expression level of H21 significantly higher than both parents
MSTRG.7730	LOC_Os12g03080	rp3 protein, putative, expressed	The expression level of H21 significantly higher than both parents
MSTRG.29007	LOC_Os09g01670; LOC_Os09g01660	peptidyl-prolyl cis-trans isomerase, FKBP-type, putative, expressed	The expression level of H21 significantly higher than both parents
MSTRG.17313	LOC_Os04g24294	OsWAK35d - OsWAK short gene, expressed	The expression level of H21 significantly higher than both parents
MSTRG.7205	LOC_Os11g39630	ZOS11-08 - C2H2 zinc finger protein, expressed	The expression level of H21 significantly higher than both parents
MSTRG.26491	LOC_Os07g45210	expressed protein	The expression level of H21 significantly higher than both parents
MSTRG.4884	LOC_Os10g29700	expressed protein	The expression level of H21 significantly higher than both parents
MSTRG.24600	LOC_Os07g03030	expressed protein	The expression level of H21 significantly higher than both parents
MSTRG.16024	LOC_Os03g56220		The expression level of H21 significantly higher than both parents
MSTRG.24627			The expression level of H21 significantly higher than both parents
MSTRG.17154	LOC_Os04g18530		The expression level of H21 significantly higher than both parents
MSTRG.24549			The expression level of H21 significantly higher than both parents
MSTRG.4703			The expression level of H21 significantly higher than both parents
MSTRG.26726	LOC_Os07g48520		The expression level of H21 significantly higher than both parents

MSTRG.25733			The expression level of H21 significantly higher than both parents
MSTRG.16971	LOC_Os04g14210		The expression level of H21 significantly higher than both parents
MSTRG.24186	LOC_Os06g47140	expressed protein	The expression level of H21 significantly higher than both parents
MSTRG.24171	LOC_Os06g46860		The expression level of H21 significantly higher than both parents
MSTRG.8474	LOC_Os12g20324	cyclin-A1, putative, expressed	The expression level of H21 significantly higher than both parents
MSTRG.15177			The expression level of H21 significantly higher than both parents
MSTRG.8773	LOC_Os12g30320	expressed protein	The expression level of H21 significantly higher than both parents
MSTRG.12921	LOC_Os02g57900	expressed protein	The expression level of H21 significantly higher than both parents
MSTRG.11274			The expression level of H21 significantly higher than both parents
MSTRG.16734			The expression level of H21 significantly higher than both parents
MSTRG.25001	LOC_Os07g09814	OsFBX221 - F-box domain containing protein, expressed	The expression level of H21 significantly higher than both parents
MSTRG.11468	LOC_Os02g35980		The expression level of H21 significantly higher than both parents
MSTRG.1098	LOC_Os01g18230		The expression level of H21 significantly higher than both parents
MSTRG.11388	LOC_Os02g34740	retrotransposon protein, putative, unclassified, expressed	The expression level of H21 significantly higher than both parents
MSTRG.23037	LOC_Os06g22394	expressed protein	The expression level of H21 significantly higher than both parents
MSTRG.12918			The expression level of H21 significantly higher than both parents
MSTRG.27180	LOC_Os08g07900		The expression level of H21 significantly higher than both parents
MSTRG.3641			The expression level of H21 significantly higher than both parents
MSTRG.11074	LOC_Os02g28030	expressed protein	The expression level of H21 significantly higher than both parents
MSTRG.25006	LOC_Os07g09950	OsFBX222 - F-box domain containing protein, expressed	The expression level of H21 significantly higher than both parents
MSTRG.10109	LOC_Os02g07260		The expression level of H21 significantly higher than both parents
MSTRG.16985	LOC_Os04g14200		The expression level of H21 significantly higher than both parents
MSTRG.11283	LOC_Os02g32814	heavy metal-associated domain containing protein, expressed	The expression level of H21 significantly higher than both parents
MSTRG.17314	LOC_Os04g24300	OsWAK35a - OsWAK short gene, expressed	The expression level of H21 significantly higher than both parents
MSTRG.8803			The expression level of H21 significantly higher than both parents
MSTRG.18251			The expression level of H21 significantly higher than both parents
MSTRG.3245	LOC_Os01g61940	white-brown complex homolog protein, putative, expressed	The expression level of H21 significantly higher than both parents
MSTRG.26134	LOC_Os07g39060		The expression level of H21 significantly higher than both parents
MSTRG.15712	LOC_Os03g51180	expressed protein	The expression level of H21 significantly higher than both parents
MSTRG.24658	LOC_Os07g04330	expressed protein	The expression level of H21 significantly higher than both parents
MSTRG.8248			The expression level of H21 significantly higher than both parents
MSTRG.24504	LOC_Os07g01070	peptide transporter, putative, expressed	The expression level of H21 significantly higher than both parents
MSTRG.11356			The expression level of H21 significantly higher than both parents
MSTRG.16727	LOC_Os04g06520		The expression level of H21 significantly higher than both parents
MSTRG.22769			The expression level of H21 significantly higher than both parents
MSTRG.2451	LOC_Os01g50310	VIP1 protein, putative, expressed	The expression level of H21 significantly higher than both parents
MSTRG.27470			The expression level of H21 significantly higher than both parents
MSTRG.25917	LOC_Os07g35985	expressed protein	The expression level of H21 significantly higher than both parents
MSTRG.28668	LOC_Os08g41190		The expression level of H21 significantly higher than both parents
MSTRG.24074	LOC_Os06g45360	peptidase, M24 family protein, putative, expressed	The expression level of H21 significantly higher than both parents
MSTRG.9894	LOC_Os02g04924; LOC_Os02g04915	expressed protein	The expression level of H21 significantly higher than both parents
MSTRG.17249	LOC_Os04g22270	expressed protein	The expression level of H21 significantly higher than both parents
MSTRG.17560	LOC_Os04g31090	expressed protein	The expression level of H21 significantly higher than both parents
MSTRG.9976	LOC_Os02g05420	expressed protein	The expression level of H21 significantly higher than both parents
MSTRG.9212	LOC_Os12g38950	zinc finger, C2H2 type family protein, expressed	The expression level of H21 significantly higher than both parents
MSTRG.23024			The expression level of H21 significantly higher than both parents
MSTRG.24878			The expression level of H21 significantly higher than both parents
MSTRG.17002	LOC_Os04g14850	retrotransposon protein, putative, Ty3-gypsy subclass, expressed	The expression level of H21 significantly higher than both parents
MSTRG.25967			The expression level of H21 significantly higher than both parents
MSTRG.12736			The expression level of H21 significantly higher than both parents
MSTRG.16983	LOC_Os04g14190; LOC_Os04g14200	expressed protein	The expression level of H21 significantly higher than both parents
MSTRG.1279	LOC_Os01g22780	GDSL-like lipase/acylhydrolase, putative, expressed	The expression level of H21 significantly higher than both parents
MSTRG.16879	LOC_Os04g10410		The expression level of H21 significantly higher than both parents
MSTRG.18254	LOC_Os04g42130	integral membrane transporter family protein, putative, expressed	The expression level of H21 significantly higher than both parents
MSTRG.6447			The expression level of H21 significantly higher than both parents
MSTRG.13927	LOC_Os03g13976	expressed protein	The expression level of H21 significantly higher than both parents
MSTRG.22697	LOC_Os06g13580	expressed protein	The expression level of H21 significantly higher than both parents
MSTRG.4329	LOC_Os10g09320		The expression level of H21 significantly higher than both parents
MSTRG.19800			The expression level of H21 significantly higher than both parents
MSTRG.7221	LOC_Os11g40030	cyclin-dependent kinase inhibitor, putative, expressed	The expression level of H21 significantly higher than both parents
MSTRG.12384	LOC_Os02g50470	expressed protein	The expression level of H21 significantly higher than both parents
MSTRG.17023	LOC_Os04g15670; LOC_Os04g15680	expressed protein	The expression level of H21 significantly higher than both parents
MSTRG.17436			The expression level of H21 significantly higher than both parents
MSTRG.4885			The expression level of H21 significantly higher than both parents
MSTRG.15090			The expression level of H21 significantly higher than both parents
MSTRG.16977	LOC_Os04g14140		The expression level of H21 significantly higher than both parents
MSTRG.16982			The expression level of H21 significantly higher than both parents
MSTRG.17687	LOC_Os04g33340		The expression level of H21 significantly higher than both parents
MSTRG.17985	LOC_Os04g38430	nodulin, putative, expressed	The expression level of H21 significantly higher than both parents
MSTRG.12315	LOC_Os02g49510		The expression level of H21 significantly higher than both parents
MSTRG.1436			The expression level of H21 significantly higher than both parents
MSTRG.7961	LOC_Os12g06900		The expression level of H21 significantly higher than both parents

MSTRG.633	LOC_Os01g10130; LOC_Os01g10140	RNA-dependent RNA polymerase, putative, expressed	The expression level of H21 significantly higher than both parents
MSTRG.10381	LOC_Os02g12350	histone deacetylase, putative, expressed	The expression level of H21 significantly higher than both parents
MSTRG.25957			The expression level of H21 significantly higher than both parents
MSTRG.5672	LOC_Os11g01154	dehydrogenase, putative, expressed	The expression level of H21 significantly higher than both parents
MSTRG.7298	LOC_Os11g41380	transposon protein, putative, unclassified, expressed	The expression level of H21 significantly higher than both parents
MSTRG.5376	LOC_Os10g38890	expressed protein	The expression level of H21 significantly higher than both parents
MSTRG.1439	LOC_Os01g28600	exo70 exocyst complex subunit family protein, putative, expressed	The expression level of H21 significantly higher than both parents
MSTRG.10174	LOC_Os02g08230	WAX2, putative, expressed	The expression level of H21 significantly higher than both parents
MSTRG.25950	LOC_Os07g36340		The expression level of H21 significantly higher than both parents
MSTRG.6198	LOC_Os11g09864	wali7, putative, expressed	The expression level of H21 significantly higher than both parents
MSTRG.13873	LOC_Os03g13200		The expression level of H21 significantly higher than both parents
MSTRG.27136	LOC_Os08g06659	expressed protein	The expression level of H21 significantly higher than both parents
MSTRG.6952			The expression level of H21 significantly higher than both parents
MSTRG.5497	LOC_Os10g40770		The expression level of H21 significantly higher than both parents
MSTRG.4861	LOC_Os10g29390	expressed protein	The expression level of H21 significantly higher than both parents
MSTRG.16801			The expression level of H21 significantly higher than both parents
MSTRG.27609	LOC_Os08g16740	expressed protein	The expression level of H21 significantly higher than both parents
MSTRG.24678	LOC_Os07g04730	expressed protein	The expression level of H21 significantly higher than both parents
MSTRG.12715	LOC_Os02g55400	ATPase 8, plasma membrane-type, putative, expressed	The expression level of H21 significantly higher than both parents
MSTRG.15186	LOC_Os03g41120	expressed protein	The expression level of H21 significantly higher than both parents
MSTRG.23443	LOC_Os06g35160	CAMK_KIN1/SNF1/Nim1_like.26 - CAMK includes calcium/calmodulin depedent protein kinases, expressed	The expression level of H21 significantly higher than both parents
MSTRG.11211	LOC_Os02g30974	expressed protein	The expression level of H21 significantly higher than both parents
MSTRG.10543	LOC_Os02g14800		The expression level of H21 significantly higher than both parents
MSTRG.4133	LOC_Os10g03620	OsFBX344 - F-box domain containing protein, expressed	The expression level of H21 significantly higher than both parents
MSTRG.27888	LOC_Os08g25710	CSLD3 - cellulose synthase-like family D, expressed	The expression level of H21 significantly higher than both parents
MSTRG.5392	LOC_Os10g39130	OsMADS56 - MADS-box family gene with MIKCc type-box, expressed	The expression level of H21 significantly lower than both parents
MSTRG.22639	LOC_Os06g12470	retrotransposon protein, putative, Ty3-gypsy subclass, expressed	The expression level of H21 significantly lower than both parents
MSTRG.14165	LOC_Os03g17410	expressed protein	The expression level of H21 significantly lower than both parents
MSTRG.8477	LOC_Os12g20390	expressed protein	The expression level of H21 significantly lower than both parents
MSTRG.9393	LOC_Os12g41480		The expression level of H21 significantly lower than both parents
MSTRG.13979	LOC_Os03g14700	C2 domain containing protein, expressed	The expression level of H21 significantly lower than both parents
MSTRG.8483	LOC_Os12g20410	matrix attachment region binding protein, putative, expressed	The expression level of H21 significantly lower than both parents
MSTRG.24657	LOC_Os07g04310	expressed protein	The expression level of H21 significantly lower than both parents
MSTRG.15642	LOC_Os03g50110	transcription regulator, putative, expressed	The expression level of H21 significantly lower than both parents
MSTRG.17026	LOC_Os04g15550	expressed protein	The expression level of H21 significantly lower than both parents
MSTRG.16208	LOC_Os03g58600	PAZ domain containing protein, putative, expressed	The expression level of H21 significantly lower than both parents
MSTRG.17324			The expression level of H21 significantly lower than both parents
MSTRG.24568	LOC_Os07g02330	protein phosphatase 2C, putative, expressed	The expression level of H21 significantly lower than both parents
MSTRG.17137			The expression level of H21 significantly lower than both parents
MSTRG.8535	LOC_Os12g22810	transposon protein, putative, CACTA, En/Spm sub-class, expressed	The expression level of H21 significantly lower than both parents
MSTRG.5117	LOC_Os10g34680	vacuolar protein sorting-associated protein 52, putative, expressed	The expression level of H21 significantly lower than both parents
MSTRG.24223	LOC_Os06g47600	thaumatin family domain containing protein, expressed	The expression level of H21 significantly lower than both parents
MSTRG.8699	LOC_Os12g27102	glycerophosphoryl diester phosphodiesterase family protein, putative, expressed	The expression level of H21 significantly lower than both parents
MSTRG.14709	LOC_Os03g26350; LOC_Os03g26360	transposon protein, putative, CACTA, En/Spm sub-class, expressed;expressed protein	The expression level of H21 significantly lower than both parents
MSTRG.12915	LOC_Os02g57860; LOC_Os02g57960	OsFBX71 - F-box domain containing protein, expressed;Leucine Rich Repeat family protein, expressed	The expression level of H21 significantly lower than both parents
MSTRG.17033	LOC_Os04g15920	dehydrogenase, putative, expressed	The expression level of H21 significantly lower than both parents
MSTRG.4671			The expression level of H21 significantly lower than both parents
MSTRG.16302	LOC_Os03g59880	expressed protein	The expression level of H21 significantly lower than both parents
MSTRG.26466	LOC_Os07g44770	expressed protein	The expression level of H21 significantly lower than both parents
MSTRG.16823			The expression level of H21 significantly lower than both parents
MSTRG.22691	LOC_Os06g13280	O-methyltransferase, putative, expressed	The expression level of H21 significantly lower than both parents
MSTRG.8683	LOC_Os12g26540		The expression level of H21 significantly lower than both parents
MSTRG.148	LOC_Os01g02920	glycosyltransferase protein, putative, expressed	The expression level of H21 significantly lower than both parents
MSTRG.2377	LOC_Os01g49219	expressed protein	The expression level of H21 significantly lower than both parents
MSTRG.4642	LOC_Os10g22830		The expression level of H21 significantly lower than both parents
MSTRG.4397	LOC_Os10g11870	transposon protein, putative, CACTA, En/Spm sub-class, expressed	The expression level of H21 significantly lower than both parents
MSTRG.8518	LOC_Os12g22020		The expression level of H21 significantly lower than both parents
MSTRG.13666	LOC_Os03g10540	OsFBX78 - F-box domain containing protein, expressed	The expression level of H21 significantly lower than both parents

MSTRG.28742	LOC_Os08g42210	expressed protein	The expression level of H21 significantly lower than both parents
MSTRG.11077	LOC_Os02g28074		The expression level of H21 significantly lower than both parents
MSTRG.25873	LOC_Os07g34964		The expression level of H21 significantly lower than both parents
MSTRG.9659	LOC_Os02g02070		The expression level of H21 significantly lower than both parents
MSTRG.17487	LOC_Os04g30180	F-box/LRR-repeat protein 14, putative, expressed	The expression level of H21 significantly lower than both parents
MSTRG.28045	LOC_Os08g30020		The expression level of H21 significantly lower than both parents
MSTRG.24447	LOC_Os06g50600; LOC_Os06g50679; LOC_Os06g50724; LOC_Os06g50715	bZIP transcription factor domain containing protein, expressed;expressed protein;START domain containing protein, expressed	The expression level of H21 significantly lower than both parents
MSTRG.4674	LOC_Os10g24050	ribosome inactivating protein, putative, expressed	The expression level of H21 significantly lower than both parents
MSTRG.11353	LOC_Os02g34120		The expression level of H21 significantly lower than both parents
MSTRG.7039	LOC_Os11g37480		The expression level of H21 significantly lower than both parents
MSTRG.25890	LOC_Os07g35180	hypothetical protein	The expression level of H21 significantly lower than both parents
MSTRG.12550	LOC_Os02g53020	transposon protein, putative, CACTA, En/Spm sub-class, expressed	The expression level of H21 significantly lower than both parents
MSTRG.11168	LOC_Os02g30114	expressed protein	The expression level of H21 significantly lower than both parents
MSTRG.25810	LOC_Os07g33420	hydroxylase, putative, expressed	The expression level of H21 significantly lower than both parents
MSTRG.23991	LOC_Os06g44034	expressed protein	The expression level of H21 significantly lower than both parents
MSTRG.28038	LOC_Os08g29854	RGH1A, putative, expressed	The expression level of H21 significantly lower than both parents
MSTRG.16969			The expression level of H21 significantly lower than both parents
MSTRG.5153	LOC_Os10g35180	white-brown complex homolog protein 11, putative, expressed	The expression level of H21 significantly lower than both parents
MSTRG.7032	LOC_Os11g37260	SEY1, putative, expressed	The expression level of H21 significantly lower than both parents
MSTRG.3319	LOC_Os01g63050	expressed protein	The expression level of H21 significantly lower than both parents
MSTRG.25664	LOC_Os07g30240	mutS family domain IV containing protein, expressed	The expression level of H21 significantly lower than both parents
MSTRG.8941			The expression level of H21 significantly lower than both parents
MSTRG.29877	LOC_Os09g27350		The expression level of H21 significantly lower than both parents
MSTRG.16569	LOC_Os03g63670	expressed protein	The expression level of H21 significantly lower than both parents
MSTRG.28556	LOC_Os08g39330	skin secretory protein xP2 precursor, putative, expressed	The expression level of H21 significantly lower than both parents
MSTRG.21816	LOC_Os05g50710	late embryogenesis abundant protein, putative, expressed	The expression level of H21 significantly lower than both parents
MSTRG.28100	LOC_Os08g31130	integral membrane protein DUF6 containing protein, expressed	The expression level of H21 significantly lower than both parents
MSTRG.4939	LOC_Os10g30970	expressed protein	The expression level of H21 significantly lower than both parents
MSTRG.15195			The expression level of H21 significantly lower than both parents
MSTRG.19968	LOC_Os05g10560	expressed protein	The expression level of H21 significantly lower than both parents
MSTRG.21602	LOC_Os05g47670	zinc finger, C3HC4 type domain containing protein, expressed	The expression level of H21 significantly lower than both parents
MSTRG.11084			The expression level of H21 significantly lower than both parents
MSTRG.13743	LOC_Os03g11420	Os3bglu6 - beta-glucosidase/beta-fucosidase/beta-galactosidase, expressed	The expression level of H21 significantly lower than both parents
MSTRG.25912	LOC_Os07g35880	beta-amylase, putative, expressed	The expression level of H21 significantly lower than both parents
MSTRG.24453	LOC_Os06g50670	expressed protein	The expression level of H21 significantly lower than both parents
MSTRG.9994	LOC_Os02g05940		The expression level of H21 significantly lower than both parents
MSTRG.17538	LOC_Os04g30800	ER lumen protein retaining receptor, putative, expressed	The expression level of H21 significantly lower than both parents
MSTRG.19540	LOC_Os05g02780	glycine-rich protein A3, putative, expressed	The expression level of H21 significantly lower than both parents
MSTRG.2005	LOC_Os01g42650	cytochrome c oxidase subunit 5B, mitochondrial precursor, putative, expressed	The expression level of H21 significantly lower than both parents
MSTRG.19431	LOC_Os05g01240	AML1, putative, expressed	The expression level of H21 significantly lower than both parents
MSTRG.27565	LOC_Os08g15450	nodulin, putative, expressed	The expression level of H21 significantly lower than both parents
MSTRG.21943	LOC_Os06g01770	expressed protein	The expression level of H21 significantly lower than both parents
MSTRG.10007	LOC_Os02g06010	integral membrane protein, putative, expressed	The expression level of H21 significantly lower than both parents
MSTRG.17625	LOC_Os04g32340	RNA-binding motif protein, putative, expressed	The expression level of H21 significantly lower than both parents
MSTRG.9365	LOC_Os12g41140	expressed protein	The expression level of H21 significantly lower than both parents
MSTRG.19250	LOC_Os04g57550	amine oxidase, flavin-containing, domain containing protein, expressed	The expression level of H21 significantly lower than both parents
MSTRG.21124	LOC_Os05g39520	methyltransferase, putative, expressed	The expression level of H21 significantly lower than both parents
MSTRG.2043	LOC_Os01g43100	protein phosphatase 2C, putative, expressed	The expression level of H21 significantly lower than both parents
MSTRG.26720	LOC_Os07g48490	stress responsive protein, putative, expressed	The expression level of H21 significantly lower than both parents
MSTRG.8720	LOC_Os12g29280		The expression level of H21 significantly lower than both parents
MSTRG.7143	LOC_Os11g38810	mannose-6-phosphate isomerase, putative, expressed	The expression level of H21 significantly lower than both parents
MSTRG.192	LOC_Os01g04409; LOC_Os01g04440	OsWAK1 - OsWAK receptor-like cytoplasmic kinase OsWAK-RLCK, expressed;expressed protein	The expression level of H21 significantly lower than both parents
MSTRG.24602	LOC_Os07g03040	expressed protein	The expression level of H21 significantly lower than both parents
MSTRG.2560	LOC_Os01g52110	RING finger and CHY zinc finger domain-containing protein 1, putative, expressed	The expression level of H21 significantly lower than both parents
MSTRG.19787	LOC_Os05g06710	GDSL-like lipase/acylhydrolase, putative, expressed	The expression level of H21 significantly lower than both parents
MSTRG.24189	LOC_Os06g47150	auxin response factor 18, putative, expressed	The expression level of H21 significantly lower than both parents

MSTRG.7527	LOC_Os11g47120	DEFL48 - Defensin and Defensin-like DEFL family, expressed	The expression level of H21 significantly lower than both parents
MSTRG.17861	LOC_Os04g35630	expressed protein	The expression level of H21 significantly lower than both parents
MSTRG.29372	LOC_Os09g14520	integral membrane protein, putative, expressed	The expression level of H21 significantly lower than both parents
MSTRG.9882	LOC_Os02g04710	cycloartenol synthase, putative, expressed	The expression level of H21 significantly lower than both parents
MSTRG.4627	LOC_Os10g22570	cellulase, putative, expressed	The expression level of H21 significantly lower than both parents
MSTRG.28402	LOC_Os08g36570	F-box protein, putative, expressed	The expression level of H21 significantly lower than both parents
MSTRG.13119	LOC_Os03g02710	hydroxymethylglutaryl-CoA synthase, putative, expressed	The expression level of H21 significantly lower than both parents
MSTRG.1589	LOC_Os01g33540	transposon protein, putative, unclassified, expressed	The expression level of H21 significantly lower than both parents
MSTRG.8799			The expression level of H21 significantly lower than both parents
MSTRG.9390			The expression level of H21 statistically close to T44
MSTRG.23842	LOC_Os06g42280	transposon protein, putative, CACTA, En/Spm sub-class, expressed	The expression level of H21 statistically close to T44
MSTRG.15354	LOC_Os03g45150		The expression level of H21 statistically close to T44
MSTRG.14717	LOC_Os03g26650	heavy metal-associated domain containing protein, expressed	The expression level of H21 statistically close to T44
MSTRG.20098	LOC_Os05g14150	polygalacturonase precursor, putative, expressed	The expression level of H21 statistically close to T44
MSTRG.6257	LOC_Os11g10880	expressed protein	The expression level of H21 statistically close to T44
MSTRG.26882			The expression level of H21 statistically close to T44
MSTRG.8080	LOC_Os12g08960; LOC_Os12g08940		The expression level of H21 statistically close to T44
MSTRG.10349	LOC_Os02g11670	glucosyltransferase, putative, expressed	The expression level of H21 statistically close to T44
MSTRG.19959			The expression level of H21 statistically close to T44
MSTRG.25059			The expression level of H21 statistically close to T44
MSTRG.9116	LOC_Os12g37130	OsFBD17 - F-box and FBD domain containing protein	The expression level of H21 statistically close to T44
MSTRG.11971	LOC_Os02g44370; LOC_Os02g44360	scarecrow transcription factor family protein, putative, expressed;scarecrow, putative, expressed	The expression level of H21 statistically close to T44
MSTRG.6284	LOC_Os11g11630		The expression level of H21 statistically close to T44
MSTRG.20713			The expression level of H21 statistically close to T44
MSTRG.23859			The expression level of H21 statistically close to T44
MSTRG.2246	LOC_Os01g47180	3-5 exonuclease domain-containing protein, putative, expressed	The expression level of H21 statistically close to T44
MSTRG.9005	LOC_Os12g35430	transposon protein, putative, unclassified, expressed	The expression level of H21 statistically close to T44
MSTRG.22240	LOC_Os06g06250	GDSL-like lipase/acylhydrolase, putative, expressed	The expression level of H21 statistically close to T44
MSTRG.9117			The expression level of H21 statistically close to T44
MSTRG.9793	LOC_Os02g03640	expressed protein	The expression level of H21 statistically close to T44
MSTRG.15386	LOC_Os03g45920		The expression level of H21 statistically close to T44
MSTRG.2892			The expression level of H21 statistically close to T44
MSTRG.24735			The expression level of H21 statistically close to T44
MSTRG.25056	LOC_Os07g10740		The expression level of H21 statistically close to T44
MSTRG.17516			The expression level of H21 statistically close to T44
MSTRG.23955	LOC_Os06g43020	expressed protein	The expression level of H21 statistically close to T44
MSTRG.20099	LOC_Os05g14160	expressed protein	The expression level of H21 statistically close to T44
MSTRG.8084	LOC_Os12g08960	hAT dimerisation domain-containing protein, putative, expressed	The expression level of H21 statistically close to T44
MSTRG.9359			The expression level of H21 statistically close to T44
MSTRG.23987	LOC_Os06g43960		The expression level of H21 statistically close to T44
MSTRG.25090	LOC_Os07g11150	expressed protein	The expression level of H21 statistically close to T44
MSTRG.6298	LOC_Os11g11694	retrotransposon protein, putative, unclassified, expressed	The expression level of H21 statistically close to T44
MSTRG.23860	LOC_Os06g42460		The expression level of H21 statistically close to T44
MSTRG.23952			The expression level of H21 statistically close to T44
MSTRG.9113			The expression level of H21 statistically close to T44
MSTRG.30383	LOC_Os09g36700	ribonuclease T2 family domain containing protein, expressed	The expression level of H21 statistically close to T44
MSTRG.4643			The expression level of H21 statistically close to T44
MSTRG.9154	LOC_Os12g37800		The expression level of H21 statistically close to T44
MSTRG.23954			The expression level of H21 statistically close to T44
MSTRG.11917	LOC_Os02g43620		The expression level of H21 statistically close to T44
MSTRG.153	LOC_Os01g03840	ZOS1-02 - C2H2 zinc finger protein, expressed	The expression level of H21 statistically close to T44
MSTRG.5436	LOC_Os10g39840	glycosyl hydrolases family 16, putative, expressed	The expression level of H21 statistically close to T44
MSTRG.29824	LOC_Os09g26340	Core histone H2A/H2B/H3/H4 domain containing protein, putative, expressed	The expression level of H21 statistically close to T44
MSTRG.9369	LOC_Os12g41170		The expression level of H21 statistically close to T44
MSTRG.20380	LOC_Os05g25080	transposon protein, putative, unclassified, expressed	The expression level of H21 statistically close to T44
MSTRG.23853	LOC_Os06g42440		The expression level of H21 statistically close to T44
MSTRG.7720	LOC_Os12g03040	no apical meristem protein, putative, expressed	The expression level of H21 statistically close to T44
MSTRG.20032	LOC_Os05g11910	GDSL-like lipase/acylhydrolase, putative, expressed	The expression level of H21 statistically close to T44
MSTRG.23901			The expression level of H21 statistically close to T44
MSTRG.15351			The expression level of H21 statistically close to T44
MSTRG.167	LOC_Os01g04050	BBTI12 - Bowman-Birk type bran trypsin inhibitor precursor, expressed	The expression level of H21 statistically close to T44

MSTRG.19752			The expression level of H21 statistically close to T44
MSTRG.8675	LOC_Os12g26290	alpha-DOX2, putative, expressed	The expression level of H21 statistically close to T44
MSTRG.25058			The expression level of H21 statistically close to T44
MSTRG.23564	LOC_Os06g37680; LOC_Os06g37690	expressed protein	The expression level of H21 statistically close to T44
MSTRG.19543			The expression level of H21 statistically close to T44
MSTRG.23845			The expression level of H21 statistically close to T44
MSTRG.232	LOC_Os01g03330	BBTI3 - Bowman-Birk type bran trypsin inhibitor precursor, expressed	The expression level of H21 statistically close to T44
MSTRG.20803	LOC_Os05g33560		The expression level of H21 statistically close to T44
MSTRG.3167			The expression level of H21 statistically close to T44
MSTRG.23840			The expression level of H21 statistically close to T44
MSTRG.6380			The expression level of H21 statistically close to T44
MSTRG.7150			The expression level of H21 statistically close to T44
MSTRG.19257	LOC_Os04g57630	phytosulfokine receptor precursor, putative, expressed	The expression level of H21 statistically close to T44
MSTRG.16039	LOC_Os03g56360	transposon protein, putative, unclassified, expressed	The expression level of H21 statistically close to T44
MSTRG.23851	LOC_Os06g42380		The expression level of H21 statistically close to T44
MSTRG.10064	LOC_Os02g06754	expressed protein	The expression level of H21 statistically close to T44
MSTRG.1875			The expression level of H21 statistically close to T44
MSTRG.29476	LOC_Os09g17120	ankyrin repeat domain containing protein, expressed	The expression level of H21 statistically close to T44
MSTRG.11743	LOC_Os02g40320; LOC_Os02g40280; LOC_Os02g40330; LOC_Os02g40340	piwi domain containing protein, putative, expressed;retrotransposon protein, putative, Ty3-gypsy subclass, expressed;expressed protein	The expression level of H21 statistically close to T44
MSTRG.10673			The expression level of H21 statistically close to T44
MSTRG.5715	LOC_Os11g01690	expressed protein	The expression level of H21 statistically close to T44
MSTRG.23846			The expression level of H21 statistically close to T44
MSTRG.23266	LOC_Os06g29250		The expression level of H21 statistically close to T44
MSTRG.5300			The expression level of H21 statistically close to T44
MSTRG.10455	LOC_Os02g12970		The expression level of H21 statistically close to T44
MSTRG.23368	LOC_Os06g32600	THION15 - Plant thionin family protein precursor, expressed	The expression level of H21 statistically close to T44
MSTRG.23217	LOC_Os06g27860	poor homologous synapsis 1 protein, putative, expressed	The expression level of H21 statistically close to T44
MSTRG.4636	LOC_Os10g22780		The expression level of H21 statistically close to T44
MSTRG.22052	LOC_Os06g03960		The expression level of H21 statistically close to T44
MSTRG.10065	LOC_Os02g06760	expressed protein	The expression level of H21 statistically close to T44
MSTRG.7148	LOC_Os11g38840		The expression level of H21 statistically close to T44
MSTRG.7353			The expression level of H21 statistically close to T44
MSTRG.18807	LOC_Os04g51440	villin protein, putative, expressed	The expression level of H21 statistically close to T44
MSTRG.23855			The expression level of H21 statistically close to T44
MSTRG.1410	LOC_Os01g27260	glutathione S-transferase, putative, expressed	The expression level of H21 statistically close to T44
MSTRG.10459			The expression level of H21 statistically close to T44
MSTRG.29523	LOC_Os09g19350	expressed protein	The expression level of H21 statistically close to T44
MSTRG.7492			The expression level of H21 statistically close to T44
MSTRG.14920	LOC_Os03g32330	expressed protein	The expression level of H21 statistically close to T44
MSTRG.26723	LOC_Os07g48550	no apical meristem protein, putative, expressed	The expression level of H21 statistically close to T44
MSTRG.5598	LOC_Os10g42150	transposon protein, putative, unclassified, expressed	The expression level of H21 statistically close to T44
MSTRG.22677	LOC_Os06g13110		The expression level of H21 statistically close to T44
MSTRG.22064			The expression level of H21 statistically close to T44
MSTRG.18602			The expression level of H21 statistically close to T44
MSTRG.27330	LOC_Os08g10760	expressed protein	The expression level of H21 statistically close to T44
MSTRG.11373	LOC_Os02g34540	tetratricopeptide repeat containing protein, putative, expressed	The expression level of H21 statistically close to T44
MSTRG.6247	LOC_Os11g10600		The expression level of H21 statistically close to T44
MSTRG.10067	LOC_Os02g06779	expressed protein	The expression level of H21 statistically close to T44
MSTRG.17383	LOC_Os04g27090		The expression level of H21 statistically close to T44
MSTRG.16715	LOC_Os04g05060		The expression level of H21 statistically close to T44
MSTRG.25176	LOC_Os07g13210; LOC_Os07g13230	retrotransposon protein, putative, unclassified, expressed;eukaryotic initiation factor 5A hypusine, DNA-binding OB fold family protein, expressed	The expression level of H21 statistically close to T44
MSTRG.2655	LOC_Os01g53560		The expression level of H21 statistically close to T44
MSTRG.4107			The expression level of H21 statistically close to T44
MSTRG.15825	LOC_Os03g52860	lipoxygenase, putative, expressed	The expression level of H21 statistically close to T44
MSTRG.4670	LOC_Os10g24000		The expression level of H21 statistically close to T44
MSTRG.24567			The expression level of H21 statistically close to T44
MSTRG.4449	LOC_Os10g14180	expressed protein	The expression level of H21 statistically close to T44
MSTRG.29524	LOC_Os09g19380		The expression level of H21 statistically close to T44
MSTRG.24160			The expression level of H21 statistically close to T44
MSTRG.25088	LOC_Os07g11160	transposon protein, putative, CACTA, En/Spm sub-class, expressed	The expression level of H21 statistically close to T44
MSTRG.10458			The expression level of H21 statistically close to T44
MSTRG.13642	LOC_Os03g10250	expressed protein	The expression level of H21 statistically close to T44
MSTRG.4616	LOC_Os10g22394	retrotransposon protein, putative, unclassified, expressed	The expression level of H21 statistically close to T44
MSTRG.8369	LOC_Os12g17350	retrotransposon protein, putative, unclassified, expressed	The expression level of H21 statistically close to T44
MSTRG.1478			The expression level of H21 statistically close to T44
MSTRG.29833	LOC_Os09g26554	expressed protein	The expression level of H21 statistically close to T44

MSTRG.23287	LOC_Os06g29640		The expression level of H21 statistically close to T44
MSTRG.23358	LOC_Os06g31280	THION1 - Plant thionin family protein precursor, putative, expressed	The expression level of H21 statistically close to T44
MSTRG.7804	LOC_Os12g04200		The expression level of H21 statistically close to T44
MSTRG.23862			The expression level of H21 statistically close to T44
MSTRG.7078	LOC_Os11g37950	WIP3 - Wound-induced protein precursor, expressed	The expression level of H21 statistically close to T44
MSTRG.28763			The expression level of H21 statistically close to T44
MSTRG.23843	LOC_Os06g42310	beta-galactosidase precursor, putative, expressed	The expression level of H21 statistically close to T44
MSTRG.23824	LOC_Os06g42050		The expression level of H21 statistically close to T44
MSTRG.6786			The expression level of H21 statistically close to T44
MSTRG.21072	LOC_Os05g38740	Core histone H2A/H2B/H3/H4 domain containing protein, putative, expressed	The expression level of H21 statistically close to T44
MSTRG.7347			The expression level of H21 statistically close to T44
MSTRG.23612	LOC_Os06g38790	ZmEBE protein, putative, expressed	The expression level of H21 statistically close to T44
MSTRG.8707	LOC_Os12g27780	transposon protein, putative, CACTA, En/Spm sub-class, expressed	The expression level of H21 statistically close to T44
MSTRG.18025	LOC_Os04g38920	DUF593 domain containing protein, expressed	The expression level of H21 statistically close to T44
MSTRG.7348			The expression level of H21 statistically close to T44
MSTRG.9389	LOC_Os12g41450		The expression level of H21 statistically close to T44
MSTRG.1314	LOC_Os01g24120		The expression level of H21 statistically close to T44
MSTRG.6794	LOC_Os11g31340	no apical meristem protein, putative, expressed	The expression level of H21 statistically close to T44
MSTRG.19203	LOC_Os04g56840	expressed protein	The expression level of H21 statistically close to T44
MSTRG.7490	LOC_Os11g45740	MYB family transcription factor, putative, expressed	The expression level of H21 statistically close to T44
MSTRG.25092			The expression level of H21 statistically close to T44
MSTRG.2238	LOC_Os01g47040	C2 domain containing protein, putative, expressed	The expression level of H21 statistically close to T44
MSTRG.1435	LOC_Os01g28300	OsFBX7 - F-box domain containing protein, expressed	The expression level of H21 statistically close to T44
MSTRG.18272	LOC_Os04g42350	heavy metal-associated domain containing protein, expressed	The expression level of H21 statistically close to T44
MSTRG.15253			The expression level of H21 statistically close to T44
MSTRG.7535	LOC_Os11g47370	expressed protein	The expression level of H21 statistically close to T44
MSTRG.9019	LOC_Os12g35610	respiratory burst oxidase, putative, expressed	The expression level of H21 statistically close to T44
MSTRG.27116	LOC_Os08g06370		The expression level of H21 statistically close to T44
MSTRG.9510	LOC_Os12g42990		The expression level of H21 statistically close to T44
MSTRG.11052	LOC_Os02g27540		The expression level of H21 statistically close to T44
MSTRG.17513			The expression level of H21 statistically close to T44
MSTRG.14515			The expression level of H21 statistically close to T44
MSTRG.2958	LOC_Os01g57540	protein kinase, putative, expressed	The expression level of H21 statistically close to T44
MSTRG.7409			The expression level of H21 statistically close to T44
MSTRG.27758			The expression level of H21 statistically close to T44
MSTRG.23453	LOC_Os06g35580		The expression level of H21 statistically close to T44
MSTRG.9580	LOC_Os02g01010		The expression level of H21 statistically close to T44
MSTRG.4708	LOC_Os10g25090		The expression level of H21 statistically close to T44
MSTRG.24017	LOC_Os06g44330	expressed protein	The expression level of H21 statistically close to T44
MSTRG.7313	LOC_Os11g41730	transposon protein, putative, CACTA, En/Spm sub-class, expressed	The expression level of H21 statistically close to T44
MSTRG.29831	LOC_Os09g26530	expressed protein	The expression level of H21 statistically close to T44
MSTRG.14962	LOC_Os03g35750		The expression level of H21 statistically close to T44
MSTRG.24752	LOC_Os07g05840; LOC_Os07g05850	expressed protein;transposon protein, putative, CACTA, En/Spm sub-class, expressed	The expression level of H21 statistically close to T44
MSTRG.15058	LOC_Os03g38720	proteasome maturation factor UMP1 family protein, expressed	The expression level of H21 statistically close to T44
MSTRG.23841	LOC_Os06g42270	expressed protein	The expression level of H21 statistically close to T44
MSTRG.20434	LOC_Os05g27650	transposon protein, putative, CACTA, En/Spm sub-class, expressed	The expression level of H21 statistically close to T44
MSTRG.7481			The expression level of H21 statistically close to T44
MSTRG.4639	LOC_Os10g22820	expressed protein	The expression level of H21 statistically close to T44
MSTRG.4436	LOC_Os10g13830	pleiotropic drug resistance protein 13, putative, expressed	The expression level of H21 statistically close to T44
MSTRG.26881			The expression level of H21 statistically close to T44
MSTRG.24648			The expression level of H21 statistically close to T44
MSTRG.3917	LOC_Os01g72490	LRP1, putative, expressed	The expression level of H21 statistically close to T44
MSTRG.2119	LOC_Os01g44220	glucose-1-phosphate adenylyltransferase large subunit, chloroplast precursor, putative, expressed	The expression level of H21 statistically close to T44
MSTRG.6258	LOC_Os11g10890	expressed protein	The expression level of H21 statistically close to T44
MSTRG.13637	LOC_Os03g10210	homeobox domain containing protein, expressed	The expression level of H21 statistically close to T44
MSTRG.12336	LOC_Os02g49700		The expression level of H21 statistically close to T44
MSTRG.8404	LOC_Os12g18390		The expression level of H21 statistically close to T44
MSTRG.25062			The expression level of H21 statistically close to T44
MSTRG.1742	LOC_Os01g37580	retrotransposon protein, putative, unclassified, expressed	The expression level of H21 statistically close to T44
MSTRG.23858	LOC_Os06g42450		The expression level of H21 statistically close to T44
MSTRG.6289	LOC_Os11g11550	NBS-LRR disease resistance protein, putative, expressed	The expression level of H21 statistically close to T44
MSTRG.5108	LOC_Os10g34430	Dicer, putative, expressed	The expression level of H21 statistically close to T44

MSTRG.8287	LOC_Os12g14440	Jacalin-like lectin domain containing protein, putative, expressed	The expression level of H21 statistically close to T44
MSTRG.19969			The expression level of H21 statistically close to T44
MSTRG.24877			The expression level of H21 statistically close to T44
MSTRG.5873	LOC_Os11g03970	CAMK_KIN1/SNF1/Nim1_like.5 - CAMK includes calcium/calmodulin depedent protein kinases, expressed	The expression level of H21 statistically close to T44
MSTRG.28065	LOC_Os08g30510	expressed protein	The expression level of H21 statistically close to T44
MSTRG.9084			The expression level of H21 statistically close to T44
MSTRG.4920	LOC_Os10g30620	expressed protein	The expression level of H21 statistically close to T44
MSTRG.27958	LOC_Os08g28460	NBS-LRR disease resistance protein, putative, expressed	The expression level of H21 statistically close to T44
MSTRG.4782			The expression level of H21 statistically close to T44
MSTRG.3298	LOC_Os01g62750	expressed protein	The expression level of H21 statistically close to T44
MSTRG.27600			The expression level of H21 statistically close to T44
MSTRG.2606			The expression level of H21 statistically close to T44
MSTRG.2731			The expression level of H21 statistically close to T44
MSTRG.12662	LOC_Os02g54790	expressed protein	The expression level of H21 statistically close to T44
MSTRG.9114	LOC_Os12g37120		The expression level of H21 statistically close to T44
MSTRG.24163			The expression level of H21 statistically close to T44
MSTRG.4430	LOC_Os10g13700	phosphoenolpyruvate carboxykinase, putative, expressed	The expression level of H21 statistically close to T44
MSTRG.6977	LOC_Os11g35970	retrotransposon protein, putative, unclassified, expressed	The expression level of H21 statistically close to T44
MSTRG.6032			The expression level of H21 statistically close to T44
MSTRG.23956	LOC_Os06g43020		The expression level of H21 statistically close to T44
MSTRG.30380	LOC_Os09g36680	ribonuclease T2 family domain containing protein, expressed	The expression level of H21 statistically close to T44
MSTRG.23550			The expression level of H21 statistically close to T44
MSTRG.29375	LOC_Os09g14590	proteasome maturation factor UMP1 family protein, expressed	The expression level of H21 statistically close to T44
MSTRG.18736	LOC_Os04g49920	sensitivity to red light reduced protein 1, putative, expressed	The expression level of H21 statistically close to T44
MSTRG.4077	LOC_Os10g01530	helix-loop-helix DNA-binding domain containing protein, expressed	The expression level of H21 statistically close to T44
MSTRG.7300	LOC_Os11g41530		The expression level of H21 statistically close to T44
MSTRG.27602			The expression level of H21 statistically close to T44
MSTRG.23856	LOC_Os06g42440	expressed protein	The expression level of H21 statistically close to T44
MSTRG.14735	LOC_Os03g27120	ICE-like protease p20 domain containing protein, putative, expressed	The expression level of H21 statistically close to T44
MSTRG.10066	LOC_Os02g06770	expressed protein	The expression level of H21 statistically close to T44
MSTRG.7926	LOC_Os12g06270		The expression level of H21 statistically close to T44
MSTRG.4076			The expression level of H21 statistically close to T44
MSTRG.7480			The expression level of H21 statistically close to T44
MSTRG.27943	LOC_Os08g28010		The expression level of H21 statistically close to T44
MSTRG.29149	LOC_Os09g06920	transposon protein, putative, CACTA, En/Spm sub-class, expressed	The expression level of H21 statistically close to T44
MSTRG.23282	LOC_Os06g29630	transposon protein, putative, unclassified, expressed	The expression level of H21 statistically close to T44
MSTRG.7268	LOC_Os11g40760; LOC_Os11g40770	expressed protein	The expression level of H21 statistically close to T44
MSTRG.18351	LOC_Os04g43650	L-allo-threonine aldolase, putative, expressed	The expression level of H21 statistically close to T44
MSTRG.9310	LOC_Os12g40160		The expression level of H21 statistically close to T44
MSTRG.9244			The expression level of H21 statistically close to T44
MSTRG.14631			The expression level of H21 statistically close to T44
MSTRG.25173			The expression level of H21 statistically close to T44
MSTRG.22062			The expression level of H21 statistically close to T44
MSTRG.23087			The expression level of H21 statistically close to T44
MSTRG.29620	LOC_Os09g21490	expressed protein	The expression level of H21 statistically close to T44
MSTRG.6293	LOC_Os11g11580		The expression level of H21 statistically close to T44
MSTRG.2320			The expression level of H21 statistically close to T44
MSTRG.19751	LOC_Os05g06240	LRR receptor kinase, putative, expressed	The expression level of H21 statistically close to T44
MSTRG.11437	LOC_Os02g35540	expressed protein	The expression level of H21 statistically close to T44
MSTRG.28055			The expression level of H21 statistically close to T44
MSTRG.10450			The expression level of H21 statistically close to T44
MSTRG.24740	LOC_Os07g05640	transporter family protein, putative, expressed	The expression level of H21 statistically close to T44
MSTRG.17514			The expression level of H21 statistically close to T44
MSTRG.7278	LOC_Os11g40860	retrotransposon protein, putative, unclassified, expressed	The expression level of H21 statistically close to T44
MSTRG.20268	LOC_Os05g20660	hypothetical protein	The expression level of H21 statistically close to T44
MSTRG.22882			The expression level of H21 statistically close to T44
MSTRG.19476	LOC_Os05g01850		The expression level of H21 statistically close to T44
MSTRG.6299			The expression level of H21 statistically close to T44
MSTRG.1733	LOC_Os01g37460	zinc finger family protein, putative, expressed	The expression level of H21 statistically close to T44
MSTRG.9132	LOC_Os12g37519	retrotransposon protein, putative, unclassified, expressed	The expression level of H21 statistically close to T44
MSTRG.29952			The expression level of H21 statistically close to T44
MSTRG.6371	LOC_Os11g12790		The expression level of H21 statistically close to T44
MSTRG.10954			The expression level of H21 statistically close to T44
MSTRG.2764	LOC_Os01g55000	expressed protein	The expression level of H21 statistically close to T44
MSTRG.21028	LOC_Os05g38040	expressed protein	The expression level of H21 statistically close to T44
MSTRG.6264	LOC_Os11g10940	expressed protein	The expression level of H21 statistically close to T44
MSTRG.23343			The expression level of H21 statistically close to T44

MSTRG.1972	LOC_Os01g42110	nodulin MtN3 family protein, putative, expressed	The expression level of H21 statistically close to T44
MSTRG.24066	LOC_Os06g45260		The expression level of H21 statistically close to T44
MSTRG.7146			The expression level of H21 statistically close to T44
MSTRG.25104			The expression level of H21 statistically close to T44
MSTRG.1787	LOC_Os01g38660	expressed protein	The expression level of H21 statistically close to T44
MSTRG.8652	LOC_Os12g25880		The expression level of H21 statistically close to T44
MSTRG.22244	LOC_Os06g06290	GDSL-like lipase/acylhydrolase, putative, expressed	The expression level of H21 statistically close to T44
MSTRG.13137	LOC_Os03g02874	retrotransposon protein, putative, Ty3-gypsy subclass, expressed	The expression level of H21 statistically close to T44
MSTRG.5888			The expression level of H21 statistically close to T44
MSTRG.18222	LOC_Os04g41690		The expression level of H21 statistically close to T44
MSTRG.5262	LOC_Os10g36703	CPuORF40 - conserved peptide uORF-containing transcript, expressed	The expression level of H21 statistically close to T44
MSTRG.23899	LOC_Os06g42480	hypothetical protein	The expression level of H21 statistically close to T44
MSTRG.20116			The expression level of H21 statistically close to T44
MSTRG.24739			The expression level of H21 statistically close to T44
MSTRG.2964	LOC_Os01g57599		The expression level of H21 statistically close to T44
MSTRG.18588	LOC_Os04g47580		The expression level of H21 statistically close to T44
MSTRG.6220			The expression level of H21 statistically close to T44
MSTRG.10348			The expression level of H21 statistically close to T44
MSTRG.23690	LOC_Os06g40230	expressed protein	The expression level of H21 statistically close to T44
MSTRG.6006	LOC_Os11g06070	expressed protein	The expression level of H21 statistically close to T44
MSTRG.8782	LOC_Os12g30490	ribosomal protein L4, putative, expressed	The expression level of H21 statistically close to T44
MSTRG.6456	LOC_Os11g15280	TNP1, putative, expressed	The expression level of H21 statistically close to T44
MSTRG.16686	LOC_Os04g02920	leucine-rich repeat family protein, putative, expressed	The expression level of H21 statistically close to T44
MSTRG.11746			The expression level of H21 statistically close to T44
MSTRG.28051	LOC_Os08g30120		The expression level of H21 statistically close to T44
MSTRG.7145			The expression level of H21 statistically close to T44
MSTRG.2594	LOC_Os01g52690		The expression level of H21 statistically close to T44
MSTRG.4551	LOC_Os10g20470	MATE efflux family protein, putative, expressed	The expression level of H21 statistically close to T44
MSTRG.27286			The expression level of H21 statistically close to T44
MSTRG.23286			The expression level of H21 statistically close to T44
MSTRG.7539	LOC_Os11g47453	expressed protein	The expression level of H21 statistically close to T44
MSTRG.16936	LOC_Os04g12950; LOC_Os04g12920	expressed protein;indole-3-acetate beta-glucosyltransferase, putative, expressed	The expression level of H21 statistically close to T44
MSTRG.29755			The expression level of H21 statistically close to T44
MSTRG.29742			The expression level of H21 statistically close to T44
MSTRG.25040			The expression level of H21 statistically close to T44
MSTRG.7570	LOC_Os11g47809	metallothionein, putative, expressed	The expression level of H21 statistically close to T44
MSTRG.22851	LOC_Os06g17070; LOC_Os06g17050	expressed protein;retrotransposon protein, putative, Ty1-copia subclass, expressed	The expression level of H21 statistically close to T44
MSTRG.17803			The expression level of H21 statistically close to T44
MSTRG.17475			The expression level of H21 statistically close to T44
MSTRG.10916			The expression level of H21 statistically close to T44
MSTRG.2494	LOC_Os01g50940	helix-loop-helix DNA-binding domain containing protein, expressed	The expression level of H21 statistically close to T44
MSTRG.9302			The expression level of H21 statistically close to T44
MSTRG.23378	LOC_Os06g32860	zinc finger C-x8-C-x5-C-x3-H type family protein, expressed	The expression level of H21 statistically close to T44
MSTRG.25700	LOC_Os07g31320		The expression level of H21 statistically close to T44
MSTRG.15037	LOC_Os03g38210	MYB family transcription factor, putative, expressed	The expression level of H21 statistically close to T44
MSTRG.6793			The expression level of H21 statistically close to T44
MSTRG.8855	LOC_Os12g32310	expressed protein	The expression level of H21 statistically close to T44
MSTRG.23347	LOC_Os06g30920	expressed protein	The expression level of H21 statistically close to T44
MSTRG.6165	LOC_Os11g09370; LOC_Os11g09375	histone deacetylase 19, putative, expressed;expressed protein	The expression level of H21 statistically close to T44
MSTRG.8120			The expression level of H21 statistically close to T44
MSTRG.23105	LOC_Os06g24240		The expression level of H21 statistically close to T44
MSTRG.26771			The expression level of H21 statistically close to T44
MSTRG.23844			The expression level of H21 statistically close to T44
MSTRG.13933	LOC_Os03g14080		The expression level of H21 statistically close to T44
MSTRG.21436			The expression level of H21 statistically close to T44
MSTRG.21574	LOC_Os05g46845	expressed protein	The expression level of H21 statistically close to T44
MSTRG.8017	LOC_Os12g07710	expressed protein	The expression level of H21 statistically close to T44
MSTRG.6244			The expression level of H21 statistically close to T44
MSTRG.17173	LOC_Os04g19470		The expression level of H21 statistically close to T44
MSTRG.7499	LOC_Os11g45809	expressed protein	The expression level of H21 statistically close to T44
MSTRG.17643			The expression level of H21 statistically close to T44
MSTRG.19368	LOC_Os04g59190	peroxidase precursor, putative, expressed	The expression level of H21 statistically close to T44
MSTRG.4169	LOC_Os10g04490	expressed protein	The expression level of H21 statistically close to T44
MSTRG.5936	LOC_Os11g04954	DNA repair protein Rad51, putative, expressed	The expression level of H21 statistically close to T44
MSTRG.4338	LOC_Os10g09850	EF hand family protein, putative, expressed	The expression level of H21 statistically close to T44
MSTRG.19162	LOC_Os04g56300		The expression level of H21 statistically close to T44
MSTRG.8832			The expression level of H21 statistically close to T44
MSTRG.9356			The expression level of H21 statistically close to T44
MSTRG.1984			The expression level of H21 statistically close to T44

MSTRG.6617	LOC_Os11g24060	permease domain containing protein, putative, expressed	The expression level of H21 statistically close to T44
MSTRG.20022			The expression level of H21 statistically close to T44
MSTRG.24876			The expression level of H21 statistically close to T44
MSTRG.6163	LOC_Os11g09395	retrotransposon protein, putative, unclassified, expressed	The expression level of H21 statistically close to T44
MSTRG.23454	LOC_Os06g35580	retrotransposon protein, putative, unclassified, expressed	The expression level of H21 statistically close to T44
MSTRG.18773	LOC_Os04g51070	helix-loop-helix DNA-binding domain containing protein, expressed	The expression level of H21 statistically close to T44
MSTRG.14471			The expression level of H21 statistically close to T44
MSTRG.27313			The expression level of H21 statistically close to T44
MSTRG.19630	LOC_Os05g04500	peroxidase precursor, putative, expressed	The expression level of H21 statistically close to T44
MSTRG.19749			The expression level of H21 statistically close to T44
MSTRG.1743			The expression level of H21 statistically close to T44
MSTRG.19595			The expression level of H21 statistically close to T44
MSTRG.28034	LOC_Os08g29809	resistance protein LR10, putative, expressed	The expression level of H21 statistically close to T44
MSTRG.9708			The expression level of H21 statistically close to T44
MSTRG.7479			The expression level of H21 statistically close to T44
MSTRG.23847			The expression level of H21 statistically close to T44
MSTRG.4403			The expression level of H21 statistically close to T44
MSTRG.2725			The expression level of H21 statistically close to T44
MSTRG.27592	LOC_Os08g16460	NBS-LRR disease resistance protein, putative, expressed	The expression level of H21 statistically close to T44
MSTRG.18482	LOC_Os04g45890		The expression level of H21 statistically close to T44
MSTRG.10327	LOC_Os02g10990	FAD binding domain of DNA photolyase domain containing protein, expressed	The expression level of H21 statistically close to T44
MSTRG.27312			The expression level of H21 statistically close to T44
MSTRG.20110			The expression level of H21 statistically close to T44
MSTRG.2676			The expression level of H21 statistically close to T44
MSTRG.15410	LOC_Os03g46290		The expression level of H21 statistically close to T44
MSTRG.9652	LOC_Os02g01850		The expression level of H21 statistically close to T44
MSTRG.4284	LOC_Os10g07602		The expression level of H21 statistically close to T44
MSTRG.7531	LOC_Os11g47330		The expression level of H21 statistically close to T44
MSTRG.9250			The expression level of H21 statistically close to T44
MSTRG.12331	LOC_Os02g49640	expressed protein	The expression level of H21 statistically close to T44
MSTRG.5844	LOC_Os11g03484	MATE efflux family protein, putative, expressed	The expression level of H21 statistically close to T44
MSTRG.738	LOC_Os01g11990;LOC_Os01g11980	SFT2, putative, expressed;expressed protein	The expression level of H21 statistically close to T44
MSTRG.3017	LOC_Os01g58330		The expression level of H21 statistically close to T44
MSTRG.19861	LOC_Os05g07940		The expression level of H21 statistically close to T44
MSTRG.20404			The expression level of H21 statistically close to T44
MSTRG.22100	LOC_Os06g04399		The expression level of H21 statistically close to T44
MSTRG.24656	LOC_Os07g04270		The expression level of H21 statistically close to T44
MSTRG.17152			The expression level of H21 statistically close to T44
MSTRG.2590			The expression level of H21 statistically close to T44
MSTRG.19740	LOC_Os05g06190	expressed protein	The expression level of H21 statistically close to T44
MSTRG.20667			The expression level of H21 statistically close to T44
MSTRG.3121	LOC_Os01g59819		The expression level of H21 statistically close to T44
MSTRG.7277			The expression level of H21 statistically close to T44
MSTRG.9045			The expression level of H21 statistically close to T44
MSTRG.27539			The expression level of H21 statistically close to T44
MSTRG.21273	LOC_Os05g42110	allyl alcohol dehydrogenase, putative, expressed	The expression level of H21 statistically close to T44
MSTRG.7915			The expression level of H21 statistically close to T44
MSTRG.2786			The expression level of H21 statistically close to T44
MSTRG.16373	LOC_Os03g60840	BBTI13 - Bowman-Birk type bran trypsin inhibitor precursor, expressed	The expression level of H21 statistically close to T44
MSTRG.19869	LOC_Os05g08060		The expression level of H21 statistically close to T44
MSTRG.9702	LOC_Os02g02560	UTP--glucose-1-phosphate uridylyltransferase, putative, expressed	The expression level of H21 statistically close to T44
MSTRG.12151	LOC_Os02g47130	expressed protein	The expression level of H21 statistically close to T44
MSTRG.23596	LOC_Os06g38510	pectate lyase precursor, putative, expressed	The expression level of H21 statistically close to T44
MSTRG.27610			The expression level of H21 statistically close to T44
MSTRG.6460	LOC_Os11g15640		The expression level of H21 statistically close to T44
MSTRG.16542	LOC_Os03g63350	expressed protein	The expression level of H21 statistically close to T44
MSTRG.4249			The expression level of H21 statistically close to T44
MSTRG.14959	LOC_Os03g35560		The expression level of H21 statistically close to T44
MSTRG.14111	LOC_Os03g16670	haloacid dehalogenase-like hydrolase family protein, putative, expressed	The expression level of H21 statistically close to T44
MSTRG.3625	LOC_Os01g67710	hypothetical protein	The expression level of H21 statistically close to T44
MSTRG.9308			The expression level of H21 statistically close to T44
MSTRG.29038			The expression level of H21 statistically close to T44
MSTRG.25558			The expression level of H21 statistically close to T44
MSTRG.7514	LOC_Os11g46070	MLA10, putative, expressed	The expression level of H21 statistically close to T44
MSTRG.9661			The expression level of H21 statistically close to T44
MSTRG.27486	LOC_Os08g13630	expressed protein	The expression level of H21 statistically close to T44
MSTRG.6271	LOC_Os11g11050		The expression level of H21 statistically close to T44
MSTRG.6757	LOC_Os11g29750	plasma membrane ATPase, putative, expressed	The expression level of H21 statistically close to T44
MSTRG.3077			The expression level of H21 statistically close to T44

MSTRG.6792	LOC_Os11g31330	no apical meristem protein, putative, expressed	The expression level of H21 statistically close to T44
MSTRG.9530	LOC_Os12g43530	no apical meristem protein, putative, expressed	The expression level of H21 statistically close to T44
MSTRG.7080	LOC_Os11g37970	WIP5 - Wound-induced protein precursor, expressed	The expression level of H21 statistically close to T44
MSTRG.2204	LOC_Os01g46260	expressed protein	The expression level of H21 statistically close to T44
MSTRG.29224	LOC_Os09g08720	cinnamoyl CoA reductase, putative, expressed	The expression level of H21 statistically close to T44
MSTRG.6248	LOC_Os11g10610; LOC_Os11g10620	NBS-LRR disease resistance protein, putative, expressed	The expression level of H21 statistically close to T44
MSTRG.20004			The expression level of H21 statistically close to T44
MSTRG.4530	LOC_Os10g19919	retrotransposon protein, putative, Ty3-gypsy subclass, expressed	The expression level of H21 statistically close to T44
MSTRG.6308	LOC_Os11g11920	resistance protein, putative, expressed	The expression level of H21 statistically close to T44
MSTRG.25057			The expression level of H21 statistically close to T44
MSTRG.24159			The expression level of H21 statistically close to T44
MSTRG.21755	LOC_Os05g49860	Core histone H2A/H2B/H3/H4 domain containing protein, putative, expressed	The expression level of H21 statistically close to T44
MSTRG.6158	LOC_Os11g09310		The expression level of H21 statistically close to T44
MSTRG.4641	LOC_Os10g22820		The expression level of H21 statistically close to T44
MSTRG.17662			The expression level of H21 statistically close to T44
MSTRG.5428	LOC_Os10g39770	zinc finger, C3HC4 type, domain containing protein, expressed	The expression level of H21 statistically close to T44
MSTRG.19741			The expression level of H21 statistically close to T44
MSTRG.26320	LOC_Os07g42380	RNA recognition motif containing protein, putative, expressed	The expression level of H21 statistically close to T44
MSTRG.11732			The expression level of H21 statistically close to T44
MSTRG.3805			The expression level of H21 statistically close to T44
MSTRG.12923			The expression level of H21 statistically close to T44
MSTRG.5735	LOC_Os11g02020	retrotransposon protein, putative, Ty3-gypsy subclass, expressed	The expression level of H21 statistically close to T44
MSTRG.7503	LOC_Os11g45880	transposon protein, putative, CACTA, En/Spm sub-class, expressed	The expression level of H21 statistically close to T44
MSTRG.22084			The expression level of H21 statistically close to T44
MSTRG.13037	LOC_Os03g01590	PAIR1, putative, expressed	The expression level of H21 statistically close to T44
MSTRG.11871	LOC_Os02g42850	MYB family transcription factor, putative, expressed	The expression level of H21 statistically close to T44
MSTRG.20714	LOC_Os05g32310		The expression level of H21 statistically close to T44
MSTRG.25091			The expression level of H21 statistically close to T44
MSTRG.21315	LOC_Os05g43070	expressed protein	The expression level of H21 statistically close to T44
MSTRG.4946	LOC_Os10g31040	Citrate transporter protein, putative, expressed	The expression level of H21 statistically close to T44
MSTRG.8362	LOC_Os12g17140	mla1, putative, expressed	The expression level of H21 statistically close to T44
MSTRG.20763	LOC_Os05g33090	expressed protein	The expression level of H21 statistically close to T44
MSTRG.23937	LOC_Os06g42860	triacylglycerol lipase precursor, putative, expressed	The expression level of H21 statistically close to T44
MSTRG.5599	LOC_Os10g42160	transposon protein, putative, unclassified, expressed	The expression level of H21 statistically close to T44
MSTRG.29338	LOC_Os09g12840	plant protein of unknown function domain containing protein, expressed	The expression level of H21 statistically close to T44
MSTRG.23832	LOC_Os06g42120	sulfotransferase domain containing protein, expressed	The expression level of H21 statistically close to T44
MSTRG.8010			The expression level of H21 statistically close to T44
MSTRG.8370	LOC_Os12g17350		The expression level of H21 statistically close to T44
MSTRG.26268	LOC_Os07g41240		The expression level of H21 statistically close to T44
MSTRG.19367	LOC_Os04g59150	peroxidase precursor, putative, expressed	The expression level of H21 statistically close to T44
MSTRG.22949	LOC_Os06g20240	latency associated nuclear antigen, putative, expressed	The expression level of H21 statistically close to T44
MSTRG.25267	LOC_Os07g16260		The expression level of H21 statistically close to T44
MSTRG.11431	LOC_Os02g35320		The expression level of H21 statistically close to T44
MSTRG.17186	LOC_Os04g20210	expressed protein	The expression level of H21 statistically close to T44
MSTRG.20939	LOC_Os05g35910; LOC_Os05g35930	expressed protein	The expression level of H21 statistically close to T44
MSTRG.8663			The expression level of H21 statistically close to T44
MSTRG.27459	LOC_Os08g13130	expressed protein	The expression level of H21 statistically close to T44
MSTRG.4270	LOC_Os10g07160	retrotransposon, putative, centromere-specific, expressed	The expression level of H21 statistically close to T44
MSTRG.23366	LOC_Os06g32550	THION14 - Plant thionin family protein precursor, putative, expressed	The expression level of H21 statistically close to T44
MSTRG.27310	LOC_Os08g10530		The expression level of H21 statistically close to T44
MSTRG.26470	LOC_Os07g44850	gibberellin receptor GID1L2, putative, expressed	The expression level of H21 statistically close to T44
MSTRG.10452			The expression level of H21 statistically close to T44
MSTRG.22454			The expression level of H21 statistically close to T44
MSTRG.12351	LOC_Os02g49860	AWPM-19-like membrane family protein, putative, expressed	The expression level of H21 statistically close to T44
MSTRG.7501	LOC_Os11g45850	WRKY61, expressed	The expression level of H21 statistically close to T44
MSTRG.29169	LOC_Os09g07440	retrotransposon protein, putative, unclassified, expressed	The expression level of H21 statistically close to T44
MSTRG.25137			The expression level of H21 statistically close to T44
MSTRG.2933	LOC_Os01g57280	rp1, putative, expressed	The expression level of H21 statistically close to T44
MSTRG.7866	LOC_Os12g05290	retrotransposon protein, putative, unclassified, expressed	The expression level of H21 statistically close to T44
MSTRG.15469			The expression level of H21 statistically close to T44

MSTRG.6558	LOC_Os11g20239	expressed protein	The expression level of H21 statistically close to T44
MSTRG.10407	LOC_Os02g12510		The expression level of H21 statistically close to T44
MSTRG.27564			The expression level of H21 statistically close to T44
MSTRG.20353			The expression level of H21 statistically close to T44
MSTRG.25723			The expression level of H21 statistically close to T44
MSTRG.29293	LOC_Os09g11260	hypothetical protein	The expression level of H21 statistically close to T44
MSTRG.15277	LOC_Os03g43750	retrotransposon, putative, centromere-specific, expressed	The expression level of H21 statistically close to T44
MSTRG.4152	LOC_Os10g03960		The expression level of H21 statistically close to T44
MSTRG.23288	LOC_Os06g29650	CDP-diacylglycerol--inositol 3-phosphatidyltransferase 1, putative, expressed	The expression level of H21 statistically close to T44
MSTRG.7276	LOC_Os11g40870		The expression level of H21 statistically close to T44
MSTRG.9031	LOC_Os12g35820		The expression level of H21 statistically close to T44
MSTRG.5597	LOC_Os10g42150		The expression level of H21 statistically close to T44
MSTRG.22845			The expression level of H21 statistically close to T44
MSTRG.3410	LOC_Os01g64640	histone H3, putative, expressed	The expression level of H21 statistically close to T44
MSTRG.27025	LOC_Os08g04980	retrotransposon protein, putative, unclassified, expressed	The expression level of H21 statistically close to T44
MSTRG.16524	LOC_Os03g63010	plastid terminal oxidase, putative, expressed	The expression level of H21 statistically close to T44
MSTRG.11049	LOC_Os02g27500	Leucine Rich Repeat family protein, expressed	The expression level of H21 statistically close to T44
MSTRG.25879	LOC_Os07g35140	receptor-like serine-threonine protein kinase, putative, expressed	The expression level of H21 statistically close to T44
MSTRG.25242			The expression level of H21 statistically close to T44
MSTRG.9025			The expression level of H21 statistically close to T44
MSTRG.29795	LOC_Os09g25880	expressed protein	The expression level of H21 statistically close to T44
MSTRG.24736			The expression level of H21 statistically close to T44
MSTRG.15888	LOC_Os03g53860	periplasmic beta-glucosidase precursor, putative, expressed	The expression level of H21 statistically close to T44
MSTRG.18318			The expression level of H21 statistically close to T44
MSTRG.16420	LOC_Os03g61360	hydrolase, alpha/beta fold family domain containing protein, expressed	The expression level of H21 statistically close to T44
MSTRG.22898	LOC_Os06g18770		The expression level of H21 statistically close to T44
MSTRG.21829			The expression level of H21 statistically close to T44
MSTRG.25123			The expression level of H21 statistically close to T44
MSTRG.16910	LOC_Os04g11840	expressed protein	The expression level of H21 statistically close to T44
MSTRG.8081	LOC_Os12g08940	retrotransposon protein, putative, Ty3-gypsy subclass, expressed	The expression level of H21 statistically close to T44
MSTRG.29438			The expression level of H21 statistically close to T44
MSTRG.9171	LOC_Os12g38150	osmotin, putative, expressed	The expression level of H21 statistically close to T44
MSTRG.15431			The expression level of H21 statistically close to T44
MSTRG.20709	LOC_Os05g32280	retrotransposon protein, putative, Ty3-gypsy subclass, expressed	The expression level of H21 statistically close to T44
MSTRG.29799			The expression level of H21 statistically close to T44
MSTRG.1409	LOC_Os01g27230	12-oxophytodienoate reductase, putative, expressed	The expression level of H21 statistically close to T44
MSTRG.2796			The expression level of H21 statistically close to T44
MSTRG.21251	LOC_Os05g41750	RecF/RecN/SMC N terminal domain containing protein, expressed	The expression level of H21 statistically close to T44
MSTRG.7594	LOC_Os11g48060	laccase-22 precursor, putative, expressed	The expression level of H21 statistically close to T44
MSTRG.27234	LOC_Os08g08970	Cupin domain containing protein, expressed	The expression level of H21 statistically close to T44
MSTRG.15606	LOC_Os03g49560		The expression level of H21 statistically close to T44
MSTRG.27591	LOC_Os08g16450	NBS-LRR disease resistance protein, putative, expressed	The expression level of H21 statistically close to T44
MSTRG.9627	LOC_Os02g01590	glycosyl hydrolases, putative, expressed	The expression level of H21 statistically close to T44
MSTRG.16472	LOC_Os03g62160	expressed protein	The expression level of H21 statistically close to T44
MSTRG.9072	LOC_Os12g36240	inhibitor I family protein, putative, expressed	The expression level of H21 statistically close to T44
MSTRG.4448	LOC_Os10g14170	expressed protein	The expression level of H21 statistically close to T44
MSTRG.3497	LOC_Os01g65850	CHD3-type chromatin-remodeling factor PICKLE, putative, expressed	The expression level of H21 statistically close to T44
MSTRG.3631	LOC_Os01g67740	chromosome segregation protein, putative, expressed	The expression level of H21 statistically close to T44
MSTRG.23553	LOC_Os06g37560	beta-galactosidase precursor, putative, expressed	The expression level of H21 statistically close to T44
MSTRG.7500	LOC_Os11g45820	expressed protein	The expression level of H21 statistically close to T44
MSTRG.17454	LOC_Os04g29270		The expression level of H21 statistically close to T44
MSTRG.26133	LOC_Os07g39030		The expression level of H21 statistically close to T44
MSTRG.14659	LOC_Os03g25550	myb-like DNA-binding domain containing protein, putative, expressed	The expression level of H21 statistically close to T44
MSTRG.7908	LOC_Os12g06080	expressed protein	The expression level of H21 statistically close to T44
MSTRG.17300	LOC_Os04g24110	anthocyanin 3-O-beta-glucosyltransferase, putative, expressed	The expression level of H21 statistically close to T44
MSTRG.29803	LOC_Os09g25950	regulator protein, putative, expressed	The expression level of H21 statistically close to T44
MSTRG.2489	LOC_Os01g50910	late embryogenesis abundant protein, group 3, putative, expressed	The expression level of H21 statistically close to T44
MSTRG.22855			The expression level of H21 statistically close to T44
MSTRG.581	LOC_Os01g09370		The expression level of H21 statistically close to T44
MSTRG.29835	LOC_Os09g26550	protease Do-like 14, putative, expressed	The expression level of H21 statistically close to T44

MSTRG.9710	LOC_Os02g02660	retrotransposon protein, putative, unclassified, expressed	The expression level of H21 statistically close to T44
MSTRG.6310	LOC_Os11g11890	protein kinase domain containing protein, expressed	The expression level of H21 statistically close to T44
MSTRG.19627	LOC_Os05g04380	peroxidase precursor, putative, expressed	The expression level of H21 statistically close to T44
MSTRG.26031	LOC_Os07g37680	expressed protein	The expression level of H21 statistically close to T44
MSTRG.19504	LOC_Os05g02240	ATCHX20, putative, expressed	The expression level of H21 statistically close to T44
MSTRG.8071	LOC_Os12g08760	carboxyvinyl-carboxyphosphonate phosphorylmutase, putative, expressed	The expression level of H21 statistically close to T44
MSTRG.5998	LOC_Os11g05940	expressed protein	The expression level of H21 statistically close to T44
MSTRG.7473	LOC_Os11g45050	NBS-LRR disease resistance protein, putative, expressed	The expression level of H21 statistically close to T44
MSTRG.5803			The expression level of H21 statistically close to T44
MSTRG.16384			The expression level of H21 statistically close to T44
MSTRG.9254	LOC_Os12g39510		The expression level of H21 statistically close to T44
MSTRG.29899	LOC_Os09g27744	expressed protein	The expression level of H21 statistically close to T44
MSTRG.29268	LOC_Os09g10650	phosphatidylinositol-4-phosphate 5-Kinase family protein, putative, expressed	The expression level of H21 statistically close to T44
MSTRG.11829	LOC_Os02g42220	transposon protein, putative, unclassified, expressed	The expression level of H21 statistically close to T44
MSTRG.26689	LOC_Os07g48090		The expression level of H21 statistically close to T44
MSTRG.23731	LOC_Os06g40880	polygalacturonase, putative, expressed	The expression level of H21 statistically close to T44
MSTRG.22614	LOC_Os06g12170	expressed protein	The expression level of H21 statistically close to T44
MSTRG.7583	LOC_Os11g47970	AAA-type ATPase family protein, putative, expressed	The expression level of H21 statistically close to T44
MSTRG.28103	LOC_Os08g30860		The expression level of H21 statistically close to T44
MSTRG.22386	LOC_Os06g08380		The expression level of H21 statistically close to T44
MSTRG.24155			The expression level of H21 statistically close to T44
MSTRG.28453	LOC_Os08g37670	plastocyanin-like domain containing protein, putative, expressed	The expression level of H21 statistically close to T44
MSTRG.13218	LOC_Os03g04070	no apical meristem protein, putative, expressed	The expression level of H21 statistically close to T44
MSTRG.3376	LOC_Os01g64110	glycosyl hydrolase, putative, expressed	The expression level of H21 statistically close to T44
MSTRG.17491	LOC_Os04g30230	F-box/LRR-repeat protein 14, putative, expressed	The expression level of H21 statistically close to T44
MSTRG.6124	LOC_Os11g08180; LOC_Os11g08190	aspartic proteinase Asp1 precursor, putative, expressed	The expression level of H21 statistically close to T44
MSTRG.6851			The expression level of H21 statistically close to T44
MSTRG.23285	LOC_Os06g29690	dynein light chain type 1 domain containing protein, expressed	The expression level of H21 statistically close to T44
MSTRG.4608	LOC_Os10g22050		The expression level of H21 statistically close to T44
MSTRG.13504	LOC_Os03g08320	ZIM domain containing protein, putative, expressed	The expression level of H21 statistically close to T44
MSTRG.29955	LOC_Os09g28580	expressed protein	The expression level of H21 statistically close to T44
MSTRG.8897	LOC_Os12g33160	RGH1A, putative, expressed	The expression level of H21 statistically close to T44
MSTRG.1283	LOC_Os01g22954	serine carboxypeptidase, putative, expressed	The expression level of H21 statistically close to T44
MSTRG.18240	LOC_Os04g41980	ATOZI1, putative, expressed	The expression level of H21 statistically close to T44
MSTRG.19561	LOC_Os05g03110	PWWP domain containing protein, expressed	The expression level of H21 statistically close to T44
MSTRG.6624			The expression level of H21 statistically close to T44
MSTRG.28404	LOC_Os08g36630	bifunctional monodehydroascorbate reductase and carbonic anhydrasenectarin-3 precursor, putative, expressed	The expression level of H21 statistically close to T44
MSTRG.12658	LOC_Os02g54730	transmembrane amino acid transporter protein, putative, expressed	The expression level of H21 statistically close to T44
MSTRG.216	LOC_Os01g03170	seven in absentia protein family protein, expressed	The expression level of H21 statistically close to T44
MSTRG.17928	LOC_Os04g37530		The expression level of H21 statistically close to T44
MSTRG.30123	LOC_Os09g31502	dehydrogenase, putative, expressed	The expression level of H21 statistically close to T44
MSTRG.4529	LOC_Os10g19910	expressed protein	The expression level of H21 statistically close to T44
MSTRG.3290	LOC_Os01g62610	peptidyl-prolyl cis-trans isomerase, FKBP-type, putative, expressed	The expression level of H21 statistically close to T44
MSTRG.19488	LOC_Os05g02030	OB-fold nucleic acid binding domain containing protein, putative, expressed	The expression level of H21 statistically close to T44
MSTRG.4632	LOC_Os10g22710	expressed protein	The expression level of H21 statistically close to T44
MSTRG.11240	LOC_Os02g32180	expressed protein	The expression level of H21 statistically close to T44
MSTRG.24565	LOC_Os07g02260	expressed protein	The expression level of H21 statistically close to T44
MSTRG.23762	LOC_Os06g41160		The expression level of H21 statistically close to T44
MSTRG.24098	LOC_Os06g45770	BTB/POZ domain containing protein, putative, expressed	The expression level of H21 statistically close to T44
MSTRG.1874			The expression level of H21 statistically close to T44
MSTRG.7147			The expression level of H21 statistically close to T44
MSTRG.22594	LOC_Os06g11840	trehalose phosphatase, putative, expressed	The expression level of H21 statistically close to T44
MSTRG.15320			The expression level of H21 statistically close to T44
MSTRG.11627	LOC_Os02g38890		The expression level of H21 statistically close to T44
MSTRG.16583	LOC_Os03g63870	expressed protein	The expression level of H21 statistically close to T44
MSTRG.28716	LOC_Os08g41800	regulatory protein, putative, expressed	The expression level of H21 statistically close to T44
MSTRG.9408	LOC_Os12g41680	No apical meristem protein, putative, expressed	The expression level of H21 statistically close to T44
MSTRG.11619	LOC_Os02g38790	expressed protein	The expression level of H21 statistically close to T44
MSTRG.17826	LOC_Os04g35200	expressed protein	The expression level of H21 statistically close to T44

MSTRG.11228	LOC_Os02g31830	expressed protein	The expression level of H21 statistically close to T44
MSTRG.29801	LOC_Os09g25934	expressed protein	The expression level of H21 statistically close to T44
MSTRG.27177	LOC_Os08g07550	hAT dimerisation domain-containing protein, putative, expressed	The expression level of H21 statistically close to T44
MSTRG.29428	LOC_Os09g15700	receptor-like protein kinase 5 precursor, putative, expressed	The expression level of H21 statistically close to T44
MSTRG.3613	LOC_Os01g67480	helix-loop-helix DNA-binding domain containing protein, expressed	The expression level of H21 statistically close to T44
MSTRG.20057	LOC_Os05g12481	expressed protein	The expression level of H21 statistically close to T44
MSTRG.17426	LOC_Os04g28260	Kinesin motor domain domain containing protein, expressed	The expression level of H21 statistically close to T44
MSTRG.5333	LOC_Os10g38110	cytochrome P450, putative, expressed	The expression level of H21 statistically close to T44
MSTRG.19545	LOC_Os05g02790	KIP1, putative, expressed	The expression level of H21 statistically close to T44
MSTRG.27881	LOC_Os08g25430	cysteine-rich receptor-like protein kinase 35 precursor, putative, expressed	The expression level of H21 statistically close to T44
MSTRG.5935			The expression level of H21 statistically close to T44
MSTRG.27166	LOC_Os08g07080	terpene synthase, putative, expressed	The expression level of H21 statistically close to T44
MSTRG.8719	LOC_Os12g29220	nodulin MtN3 family protein, putative, expressed	The expression level of H21 statistically close to T44
MSTRG.24679			The expression level of H21 statistically close to T44
MSTRG.14224	LOC_Os03g18190	importin subunit beta, putative, expressed	The expression level of H21 statistically close to T44
MSTRG.24108			The expression level of H21 statistically close to T44
MSTRG.25439	LOC_Os07g23660	retrotransposon protein, putative, unclassified, expressed	The expression level of H21 statistically close to T44
MSTRG.28797	LOC_Os08g42940		The expression level of H21 statistically close to T44
MSTRG.10887	LOC_Os02g22700;<br>LOC_Os02g22760	retrotransposon protein, putative, LINE subclass, expressed	The expression level of H21 statistically close to T44
MSTRG.7226	LOC_Os11g40110	RWP-RK, putative, expressed	The expression level of H21 statistically close to T44
MSTRG.28149	LOC_Os08g31980	trehalose-6-phosphate synthase, putative, expressed	The expression level of H21 statistically close to T44
MSTRG.11562	LOC_Os02g37850	expressed protein	The expression level of H21 statistically close to T44
MSTRG.2948	LOC_Os01g57480	serine/threonine-protein kinase receptor precursor, putative, expressed	The expression level of H21 statistically close to T44
MSTRG.7330			The expression level of H21 statistically close to T44
MSTRG.16265	LOC_Os03g59320	expressed protein	The expression level of H21 statistically close to T44
MSTRG.3157	LOC_Os01g60309	retrotransposon protein, putative, Ty1-copia subclass, expressed	The expression level of H21 statistically close to T44
MSTRG.8269	LOC_Os12g13950	POLA2 - Putative DNA polymerase alpha complex subunit, expressed	The expression level of H21 statistically close to T44
MSTRG.3431	LOC_Os01g64900	HEAT, putative, expressed	The expression level of H21 statistically close to T44
MSTRG.2021			The expression level of H21 statistically close to T44
MSTRG.5033	LOC_Os10g33310	cyclin-dependent kinase inhibitor, putative, expressed	The expression level of H21 statistically close to T44
MSTRG.18892	LOC_Os04g52540	retrotransposon protein, putative, unclassified, expressed	The expression level of H21 statistically close to T44
MSTRG.24307	LOC_Os06g48920	expressed protein	The expression level of H21 statistically close to T44
MSTRG.28494	LOC_Os08g38300	Core histone H2A/H2B/H3/H4 domain containing protein, putative, expressed	The expression level of H21 statistically close to T44
MSTRG.11859	LOC_Os02g42650	expansin precursor, putative, expressed	The expression level of H21 statistically close to T44
MSTRG.5846	LOC_Os11g03550	expressed protein	The expression level of H21 statistically close to T44
MSTRG.22967	LOC_Os06g20470	expressed protein	The expression level of H21 statistically close to T44
MSTRG.24234	LOC_Os06g47800	disease resistance protein RGA3, putative, expressed	The expression level of H21 statistically close to T44
MSTRG.5075	LOC_Os10g33920	transporter-related, putative, expressed	The expression level of H21 statistically close to T44
MSTRG.7034			The expression level of H21 statistically close to T44
MSTRG.22412	LOC_Os06g08790	ORC1 - Putative origin recognition complex subunit 1, expressed	The expression level of H21 statistically close to T44
MSTRG.7550	LOC_Os11g47570	glycosyl hydrolase, putative, expressed	The expression level of H21 statistically close to T44
MSTRG.379	LOC_Os01g05900;<br>LOC_Os01g05890;<br>LOC_Os01g05970	OsFBX2 - F-box domain containing protein, expressed;Core histone H2A/H2B/H3/H4 domain containing protein, putative, expressed;OsFBO1 - F-box and other domain containing protein, expressed	The expression level of H21 statistically close to T44
MSTRG.6134			The expression level of H21 statistically close to T44
MSTRG.19823			The expression level of H21 statistically close to T44
MSTRG.19503	LOC_Os05g02300	Core histone H2A/H2B/H3/H4 domain containing protein, putative, expressed	The expression level of H21 statistically close to T44
MSTRG.4112	LOC_Os10g02880	O-methyltransferase, putative, expressed	The expression level of H21 statistically close to T44
MSTRG.5370	LOC_Os10g38489		The expression level of H21 statistically close to T44
MSTRG.29063			The expression level of H21 statistically close to T44
MSTRG.28050	LOC_Os08g30070	expressed protein	The expression level of H21 statistically close to T44
MSTRG.7265			The expression level of H21 statistically close to T44
MSTRG.14813	LOC_Os03g29584	expressed protein	The expression level of H21 statistically close to T44
MSTRG.13551	LOC_Os03g08810		The expression level of H21 statistically close to T44
MSTRG.6878	LOC_Os11g33230		The expression level of H21 statistically close to T44
MSTRG.9841	LOC_Os02g04230	CGMC_MAPKCMGC_2.7 - CGMC includes CDA, MAPK, GSK3, and CLKC kinases, expressed	The expression level of H21 statistically close to T44
MSTRG.8794	LOC_Os12g30640	ZmEBE-2 protein, putative, expressed	The expression level of H21 statistically close to T44
MSTRG.29153			The expression level of H21 statistically close to T44
MSTRG.25811	LOC_Os07g33450	retrotransposon protein, putative, unclassified, expressed	The expression level of H21 statistically close to T44

MSTRG.11169	LOC_Os02g30170	hypothetical protein	The expression level of H21 statistically close to T44
LOC_Os12g16200	LOC_Os12g16200	glutathione synthetase, chloroplast precursor, putative, expressed	The expression level of H21 statistically close to T44
MSTRG.25806	LOC_Os07g33370	IQ calmodulin-binding motif family protein, expressed	The expression level of H21 statistically close to T44
MSTRG.5026	LOC_Os10g33210	peptide transporter PTR3-A, putative, expressed	The expression level of H21 statistically close to T44
MSTRG.18255	LOC_Os04g42134	enhancer of rudimentary protein, putative, expressed	The expression level of H21 statistically close to T44
MSTRG.3804	LOC_Os01g70660	potassium transporter, putative, expressed	The expression level of H21 statistically close to T44
MSTRG.15966	LOC_Os03g55410	peroxidase precursor, putative, expressed	The expression level of H21 statistically close to T44
MSTRG.29061	LOC_Os09g03190	expressed protein	The expression level of H21 statistically close to T44
MSTRG.17022	LOC_Os04g15660	receptor kinase, putative, expressed	The expression level of H21 statistically close to T44
MSTRG.12480	LOC_Os02g51930	cytokinin-O-glucosyltransferase 2, putative, expressed	The expression level of H21 statistically close to T44
MSTRG.23305			The expression level of H21 statistically close to T44
MSTRG.218	LOC_Os01g03190	expressed protein	The expression level of H21 statistically close to T44
MSTRG.13837	LOC_Os03g12570	expressed protein	The expression level of H21 statistically close to T44
MSTRG.27291	LOC_Os08g10080	no apical meristem protein, putative, expressed	The expression level of H21 statistically close to T44
MSTRG.14473	LOC_Os03g21820	expansin precursor, putative, expressed	The expression level of H21 statistically close to T44
MSTRG.18200	LOC_Os04g41310	STRUBBELIG-RECEPTOR FAMILY 8 precursor, putative, expressed	The expression level of H21 statistically close to T44
MSTRG.18722	LOC_Os04g49780		The expression level of H21 statistically close to T44
MSTRG.18675	LOC_Os04g49000	zinc finger, C3HC4 type domain containing protein, expressed	The expression level of H21 statistically close to T44
MSTRG.24622	LOC_Os07g03240		The expression level of H21 statistically close to T44
MSTRG.15359	LOC_Os03g45250	2-aminoethanethiol dioxygenase, putative, expressed	The expression level of H21 statistically close to T44
MSTRG.24305	LOC_Os06g48870	methyl-binding domain protein MBD, putative, expressed	The expression level of H21 statistically close to T44
MSTRG.25718	LOC_Os07g31670	expressed protein	The expression level of H21 statistically close to T44
MSTRG.12085			The expression level of H21 statistically close to T44
MSTRG.865	LOC_Os01g13860; LOC_Os01g13810; LOC_Os01g13870	expressed protein	The expression level of H21 statistically close to T44
MSTRG.26772	LOC_Os07g49140	expressed protein	The expression level of H21 statistically close to T44
MSTRG.14047	LOC_Os03g15740	uncharacterized TPR repeat-containing protein, putative, expressed	The expression level of H21 statistically close to T44
MSTRG.5877	LOC_Os11g04070	60S acidic ribosomal protein P0, putative, expressed	The expression level of H21 statistically close to T44
MSTRG.7229	LOC_Os11g40160	expressed protein	The expression level of H21 statistically close to T44
MSTRG.26560			The expression level of H21 statistically close to T44
MSTRG.8317	LOC_Os12g15450	expressed protein	The expression level of H21 statistically close to T44
MSTRG.7899			The expression level of H21 statistically close to T44
MSTRG.11947			The expression level of H21 statistically close to T44
MSTRG.24618	LOC_Os07g03220		The expression level of H21 statistically close to T44
MSTRG.9112			The expression level of H21 statistically close to T44
MSTRG.27920			The expression level of H21 statistically close to T44
MSTRG.29997			The expression level of H21 statistically close to T44
MSTRG.169	LOC_Os01g04090	expressed protein	The expression level of H21 statistically close to T44
MSTRG.12062	LOC_Os02g45830	expressed protein	The expression level of H21 statistically close to T44
MSTRG.8899	LOC_Os12g33194	expressed protein	The expression level of H21 statistically close to T44
MSTRG.5805	LOC_Os11g02850	expressed protein	The expression level of H21 statistically close to T44
MSTRG.16847	LOC_Os04g09390	HEV3 - Hevein family protein precursor, expressed	The expression level of H21 statistically close to T44
LOC_Os08g30340	LOC_Os08g30340	PAS2, putative, expressed	The expression level of H21 statistically close to T44
MSTRG.5496			The expression level of H21 statistically close to T44
MSTRG.1916	LOC_Os01g41420	transmembrane amino acid transporter protein, putative, expressed	The expression level of H21 statistically close to T44
MSTRG.19754			The expression level of H21 statistically close to T44
MSTRG.19156	LOC_Os04g56210		The expression level of H21 statistically close to T44
MSTRG.29305			The expression level of H21 statistically close to T44
MSTRG.4752	LOC_Os10g26560	expressed protein	The expression level of H21 statistically close to T44
MSTRG.18462	LOC_Os04g45665	hypothetical protein	The expression level of H21 statistically close to T44
MSTRG.24023	LOC_Os06g44410	histidine kinase, putative, expressed	The expression level of H21 statistically close to T44
MSTRG.11474	LOC_Os02g36140	terpene synthase, putative, expressed	The expression level of H21 statistically close to T44
MSTRG.11964	LOC_Os02g44250		The expression level of H21 statistically close to T44
MSTRG.9623	LOC_Os02g01540	ribosomal protein, putative, expressed	The expression level of H21 statistically close to T44
MSTRG.19371	LOC_Os04g59260	peroxidase precursor, putative, expressed	The expression level of H21 statistically close to T44
MSTRG.25968	LOC_Os07g36630	CSLF8 - cellulose synthase-like family F; beta1,3;1,4 glucan synthase, expressed	The expression level of H21 statistically close to T44
MSTRG.4214	LOC_Os10g05580	expressed protein	The expression level of H21 statistically close to T44
MSTRG.21796	LOC_Os05g50410	Rad21 / Rec8 like protein, putative, expressed	The expression level of H21 statistically close to T44
MSTRG.17342	LOC_Os04g25420		The expression level of H21 statistically close to T44
MSTRG.29238	LOC_Os09g09220	protein kinase domain containing protein, expressed	The expression level of H21 statistically close to T44
MSTRG.15705	LOC_Os03g51080	glutamate decarboxylase, putative, expressed	The expression level of H21 statistically close to T44
MSTRG.22544	LOC_Os06g11050	cyclin-dependent kinase inhibitor, putative, expressed	The expression level of H21 statistically close to T44

MSTRG.959	LOC_Os01g15520	expressed protein	The expression level of H21 statistically close to T44
MSTRG.2826	LOC_Os01g55830	glutathione S-transferase, putative, expressed	The expression level of H21 statistically close to T44
MSTRG.20119	LOC_Os05g14590	MCM6 - Putative minichromosome maintenance MCM complex subunit 6, expressed	The expression level of H21 statistically close to T44
MSTRG.16981	LOC_Os04g14170	retrotransposon protein, putative, unclassified, expressed	The expression level of H21 statistically close to T44
MSTRG.985	LOC_Os01g15910	UTP--glucose-1-phosphate uridylyltransferase, putative, expressed	The expression level of H21 statistically close to T44
MSTRG.4811	LOC_Os10g28000	glutathione reductase, putative, expressed	The expression level of H21 statistically close to T44
MSTRG.3666	LOC_Os01g68330	antigen peptide transporter-like 1, chloroplast precursor, putative, expressed	The expression level of H21 statistically close to T44
MSTRG.25271	LOC_Os07g16320		The expression level of H21 statistically close to T44
MSTRG.18108	LOC_Os04g40090	zinc finger, ZZ type family protein, expressed	The expression level of H21 statistically close to T44
MSTRG.16396	LOC_Os03g61070		The expression level of H21 statistically close to T44
MSTRG.9454	LOC_Os12g42200	ATCHX, putative, expressed	The expression level of H21 statistically close to T44
MSTRG.307			The expression level of H21 statistically close to T44
MSTRG.22003	LOC_Os06g02590	DUF1336 domain containing protein, expressed	The expression level of H21 statistically close to T44
MSTRG.18333	LOC_Os04g43270	WAX2, putative, expressed	The expression level of H21 statistically close to T44
MSTRG.16976	LOC_Os04g14140	expressed protein	The expression level of H21 statistically close to T44
MSTRG.19373	LOC_Os04g59300	strictosidine synthase, putative, expressed	The expression level of H21 statistically close to T44
MSTRG.16615	LOC_Os03g64415	kinesin motor domain containing protein, putative, expressed	The expression level of H21 statistically close to T44
MSTRG.16519			The expression level of H21 statistically close to T44
MSTRG.29848	LOC_Os09g26780	zinc-finger protein, putative, expressed	The expression level of H21 statistically close to T44
MSTRG.6843			The expression level of H21 statistically close to T44
MSTRG.25183	LOC_Os07g13520	expressed protein	The expression level of H21 statistically close to T44
MSTRG.7742	LOC_Os12g03260	MATE efflux family protein, putative, expressed	The expression level of H21 statistically close to T44
MSTRG.21142	LOC_Os05g39850	MCM3 - Putative minichromosome maintenance MCM complex subunit 3, expressed	The expression level of H21 statistically close to T44
MSTRG.12629			The expression level of H21 statistically close to T44
MSTRG.7534			The expression level of H21 statistically close to T44
MSTRG.4832	LOC_Os10g28350	1,2-dihydroxy-3-keto-5-methylthiopentene dioxygenase protein, putative, expressed	The expression level of H21 statistically close to T44
MSTRG.26688	LOC_Os07g48090	CAMK_KIN1/SNF1/Nim1_like.30 - CAMK includes calcium/calmodulin depedent protein kinases, expressed	The expression level of H21 statistically close to T44
MSTRG.7310			The expression level of H21 statistically close to T44
MSTRG.10941	LOC_Os02g24720	LTPL21 - Protease inhibitor/seed storage/LTP family protein precursor, expressed	The expression level of H21 statistically close to T44
MSTRG.9883	LOC_Os02g04720	expressed protein	The expression level of H21 statistically close to T44
MSTRG.18884			The expression level of H21 statistically close to T44
MSTRG.16115	LOC_Os03g57220	hydroxyacid oxidase 1, putative, expressed	The expression level of H21 statistically close to T44
MSTRG.10100	LOC_Os02g07180	expressed protein	The expression level of H21 statistically close to T44
MSTRG.8331	LOC_Os12g16280	expressed protein	The expression level of H21 statistically close to T44
MSTRG.17743	LOC_Os04g34180	expressed protein	The expression level of H21 statistically close to T44
MSTRG.2704			The expression level of H21 statistically close to T44
MSTRG.29757	LOC_Os09g25070	WRKY62, expressed	The expression level of H21 statistically close to T44
MSTRG.28702	LOC_Os08g41620	ubiquitin carboxyl-terminal hydrolase family protein, expressed	The expression level of H21 statistically close to T44
MSTRG.24699	LOC_Os07g05110	PLA IIIA/PLP7, putative, expressed	The expression level of H21 statistically close to T44
MSTRG.21785	LOC_Os05g50260	polygalacturonase, putative, expressed	The expression level of H21 statistically close to T44
MSTRG.22054	LOC_Os06g03980	expressed protein	The expression level of H21 statistically close to T44
MSTRG.13559	LOC_Os03g08900	MATE efflux family protein, putative, expressed	The expression level of H21 statistically close to T44
MSTRG.8824	LOC_Os12g31810	cyclin, putative, expressed	The expression level of H21 statistically close to T44
MSTRG.11948			The expression level of H21 statistically close to T44
MSTRG.5729	LOC_Os11g01990		The expression level of H21 statistically close to T44
MSTRG.2468	LOC_Os01g50610	SAM dependent carboxyl methyltransferase, putative, expressed	The expression level of H21 statistically close to T44
MSTRG.29773	LOC_Os09g25380	kinesin motor protein-related, putative, expressed	The expression level of H21 statistically close to T44
MSTRG.7097	LOC_Os11g38200	OsFBDUF60 - F-box and DUF domain containing protein, expressed	The expression level of H21 statistically close to T44
MSTRG.8667	LOC_Os12g26060	bifunctional dihydrofolate reductase-thymidylate synthase, putative, expressed	The expression level of H21 statistically close to T44
MSTRG.13439	LOC_Os03g07350	CSLA4 - cellulose synthase-like family A; mannan synthase, expressed	The expression level of H21 statistically close to T44
MSTRG.25896	LOC_Os07g35290	TKL_IRAK_DUF26-lc.10 - DUF26 kinases have homology to DUF26 containing loci, expressed	The expression level of H21 statistically close to T44
MSTRG.6157	LOC_Os11g09270; LOC_Os11g09260	expressed protein;ankyrin repeat family protein, putative, expressed	The expression level of H21 statistically close to T44
MSTRG.23444	LOC_Os06g35165	expressed protein	The expression level of H21 statistically close to T44

MSTRG.2836	LOC_Os01g56010	serpin domain containing protein, putative, expressed	The expression level of H21 statistically close to T44
MSTRG.14400	LOC_Os03g20680	late embryogenesis abundant protein 1, putative, expressed	The expression level of H21 statistically close to T44
MSTRG.1084	LOC_Os01g17402	cyclin, putative, expressed	The expression level of H21 statistically close to T44
MSTRG.30268	LOC_Os09g34150	NBS-LRR disease resistance protein, putative, expressed	The expression level of H21 statistically close to T44
MSTRG.2737	LOC_Os01g54700	retrotransposon protein, putative, unclassified, expressed	The expression level of H21 statistically close to T44
MSTRG.12944	LOC_Os02g58220	RPA2A - Putative single-stranded DNA binding complex subunit 2, expressed	The expression level of H21 statistically close to T44
MSTRG.24299	LOC_Os06g48720	cadmium/zinc-transporting ATPase 4, putative, expressed	The expression level of H21 statistically close to T44
MSTRG.2027	LOC_Os01g42850	ThiF family domain containing protein, putative, expressed	The expression level of H21 statistically close to T44
MSTRG.2982	LOC_Os01g57890	Homeobox domain containing protein, expressed	The expression level of H21 statistically close to T44
MSTRG.13427	LOC_Os03g07200	expressed protein	The expression level of H21 statistically close to T44
MSTRG.19915	LOC_Os05g08910		The expression level of H21 statistically close to T44
MSTRG.7845	LOC_Os12g04980	DNA repair protein Rad51, putative, expressed	The expression level of H21 statistically close to T44
MSTRG.4267			The expression level of H21 statistically close to T44
MSTRG.16274			The expression level of H21 statistically close to T44
MSTRG.11243	LOC_Os02g32190;<br>LOC_Os02g32200	expressed protein;thioesterase family protein, putative, expressed	The expression level of H21 statistically close to T44
MSTRG.2677	LOC_Os01g53810	peptidase, putative, expressed	The expression level of H21 statistically close to T44
MSTRG.10678	LOC_Os02g18410	salt stress root protein RS1, putative, expressed	The expression level of H21 statistically close to T44
MSTRG.2452	LOC_Os01g50310		The expression level of H21 statistically close to T44
MSTRG.15949	LOC_Os03g55240	cytochrome P450, putative, expressed	The expression level of H21 statistically close to T44
MSTRG.23669	LOC_Os06g40020	DEAD-box ATP-dependent RNA helicase 52A, putative, expressed	The expression level of H21 statistically close to T44
MSTRG.354	LOC_Os01g05610	Core histone H2A/H2B/H3/H4 domain containing protein, putative, expressed	The expression level of H21 statistically close to T44
MSTRG.14234	LOC_Os03g18350	importin subunit beta, putative, expressed	The expression level of H21 statistically close to T44
MSTRG.6628	LOC_Os11g24374	OsSCP55 - Putative Serine Carboxypeptidase homologue, expressed	The expression level of H21 statistically close to T44
MSTRG.25496	LOC_Os07g25740	expressed protein	The expression level of H21 statistically close to T44
MSTRG.8079			The expression level of H21 statistically close to T44
MSTRG.2385	LOC_Os01g49300		The expression level of H21 statistically close to T44
MSTRG.20136	LOC_Os05g14980	expressed protein	The expression level of H21 statistically close to T44
MSTRG.18507	LOC_Os04g46220	ethylene-responsive transcription factor, putative, expressed	The expression level of H21 statistically close to T44
MSTRG.13070	LOC_Os03g02030	folylpolyglutamate synthase, mitochondrial precursor, putative, expressed	The expression level of H21 statistically close to T44
MSTRG.1991	LOC_Os01g42380	pleiotropic drug resistance protein, putative, expressed	The expression level of H21 statistically close to T44
MSTRG.2837	LOC_Os01g56020	expressed protein	The expression level of H21 statistically close to T44
MSTRG.26955	LOC_Os08g03770	expressed protein	The expression level of H21 statistically close to T44
MSTRG.5341	LOC_Os10g38160;<br>LOC_Os10g38470;<br>LOC_Os10g38540;<br>LOC_Os10g38580	glutathione S-transferase, putative, expressed	The expression level of H21 statistically close to T44
MSTRG.30011	LOC_Os09g29660	white-brown complex homolog protein 11, putative, expressed	The expression level of H21 statistically close to T44
MSTRG.6324	LOC_Os11g12000	NBS-LRR disease resistance protein, putative, expressed	The expression level of H21 statistically close to T44
MSTRG.21578	LOC_Os05g46920	expressed protein	The expression level of H21 statistically close to T44
MSTRG.2072	LOC_Os01g43480	AAA-type ATPase family protein, putative, expressed	The expression level of H21 statistically close to T44
MSTRG.5942	LOC_Os11g05010	heavy-metal-associated domain-containing protein, putative, expressed	The expression level of H21 statistically close to T44
MSTRG.4623	LOC_Os10g22520	cellulase, putative, expressed	The expression level of H21 statistically close to T44
MSTRG.18652			The expression level of H21 statistically close to T44
MSTRG.3232	LOC_Os01g61760	myosin heavy chain-related, putative, expressed	The expression level of H21 statistically close to T44
MSTRG.12801	LOC_Os02g56600	no apical meristem protein, putative, expressed	The expression level of H21 statistically close to T44
MSTRG.30070	LOC_Os09g30446	transporter, monovalent cation:proton antiporter-2 family, putative, expressed	The expression level of H21 statistically close to T44
MSTRG.6145	LOC_Os11g09020	amino acid transporter, putative, expressed	The expression level of H21 statistically close to T44
MSTRG.19776	LOC_Os05g06530		The expression level of H21 statistically close to T44
MSTRG.5194			The expression level of H21 statistically close to T44
MSTRG.20903	LOC_Os05g35340	expressed protein	The expression level of H21 statistically close to T44
MSTRG.14219	LOC_Os03g18160	mitochondrial carrier protein, putative, expressed	The expression level of H21 statistically close to T44
MSTRG.16299	LOC_Os03g59850	expressed protein	The expression level of H21 statistically close to T44
MSTRG.3500	LOC_Os01g65890		The expression level of H21 statistically close to T44
MSTRG.5226	LOC_Os10g35960		The expression level of H21 statistically close to T44
MSTRG.22176	LOC_Os06g05420	expressed protein	The expression level of H21 statistically close to T44
MSTRG.7839	LOC_Os12g04860	DTW domain containing protein, putative, expressed	The expression level of H21 statistically close to T44

MSTRG.27139	LOC_Os08g06690	expressed protein	The expression level of H21 statistically close to T44
MSTRG.7925	LOC_Os12g06270	retrotransposon protein, putative, unclassified, expressed	The expression level of H21 statistically close to T44
MSTRG.24790	LOC_Os07g06830	gibberellin receptor GID1L2, putative, expressed	The expression level of H21 statistically close to T44
MSTRG.1832	LOC_Os01g39710	expressed protein	The expression level of H21 statistically close to T44
MSTRG.15350	LOC_Os03g45120	ribosome inactivating protein, putative, expressed	The expression level of H21 statistically close to T44
MSTRG.19444	LOC_Os05g01444	polygalacturonase inhibitor precursor, putative, expressed	The expression level of H21 statistically close to T44
MSTRG.26049	LOC_Os07g37920	no apical meristem protein, putative, expressed	The expression level of H21 statistically close to T44
MSTRG.29603	LOC_Os09g21230	AMP-binding enzyme, putative, expressed	The expression level of H21 statistically close to T44
MSTRG.11813	LOC_Os02g41954	gibberellin 2-beta-dioxygenase 7, putative, expressed	The expression level of H21 statistically close to T44
MSTRG.13401			The expression level of H21 statistically close to T44
MSTRG.2754	LOC_Os01g54890		The expression level of H21 statistically close to T44
MSTRG.4808	LOC_Os10g27450	tetrapyrrole methylase family protein, putative, expressed	The expression level of H21 statistically close to T44
MSTRG.16198	LOC_Os03g58400; LOC_Os03g58420	C-5 cytosine-specific DNA methylase, putative, expressed;WRKY6, expressed	The expression level of H21 statistically close to T44
MSTRG.15751	LOC_Os03g51650	membrane protein, putative, expressed	The expression level of H21 statistically close to T44
MSTRG.2714	LOC_Os01g54420	dynamin family protein, putative, expressed	The expression level of H21 statistically close to T44
MSTRG.14561	LOC_Os03g23050	expressed protein	The expression level of H21 statistically close to T44
MSTRG.15635	LOC_Os03g50030	phospholipase A2, putative, expressed	The expression level of H21 statistically close to T44
MSTRG.238	LOC_Os01g03390	BBTI7 - Bowman-Birk type bran trypsin inhibitor precursor, expressed	The expression level of H21 statistically close to T44
MSTRG.2170	LOC_Os01g45640	tat pathway signal sequence family protein, expressed	The expression level of H21 statistically close to T44
MSTRG.3382	LOC_Os01g64230	long cell-linked locus protein, putative, expressed	The expression level of H21 statistically close to T44
MSTRG.19544	LOC_Os05g02810; LOC_Os05g02790	protein binding protein, putative, expressed	The expression level of H21 statistically close to T44
MSTRG.20573	LOC_Os05g29170	expressed protein	The expression level of H21 statistically close to T44
MSTRG.9193	LOC_Os12g38670; LOC_Os12g38660; LOC_Os12g38720	transposon protein, putative, CACTA, En/Spm sub-class, expressed;expressed protein	The expression level of H21 statistically close to T44
MSTRG.17669	LOC_Os04g33150	desiccation-related protein PCC13-62 precursor, putative, expressed	The expression level of H21 statistically close to T44
MSTRG.13925	LOC_Os03g13984		The expression level of H21 statistically close to T44
MSTRG.7386	LOC_Os11g42800	kinesin motor domain containing protein, putative, expressed	The expression level of H21 statistically close to T44
MSTRG.24194	LOC_Os06g47250	DUF292 domain containing protein, expressed	The expression level of H21 statistically close to T44
MSTRG.21312	LOC_Os05g43050	expressed protein	The expression level of H21 statistically close to T44
MSTRG.28922	LOC_Os08g44430; LOC_Os08g44440; LOC_Os08g44470	vesicle-associated membrane protein 727, putative, expressed;multiple myeloma tumor-associated protein 2, putative, expressed;expressed protein	The expression level of H21 statistically close to T44
MSTRG.24609	LOC_Os07g03120	expressed protein	The expression level of H21 statistically close to T44
MSTRG.21198	LOC_Os05g40920	expressed protein	The expression level of H21 statistically close to T44
MSTRG.868	LOC_Os01g13830	expressed protein	The expression level of H21 statistically close to T44
MSTRG.825	LOC_Os01g13260	cyclin-A1, putative, expressed	The expression level of H21 statistically close to T44
MSTRG.11490	LOC_Os02g36570	ABC1 family domain containing protein, putative, expressed	The expression level of H21 statistically close to T44
MSTRG.14738	LOC_Os03g27230	phospho-2-dehydro-3-deoxyheptonate aldolase, chloroplast precursor, putative, expressed	The expression level of H21 statistically close to T44
MSTRG.13982			The expression level of H21 statistically close to T44
MSTRG.7848	LOC_Os12g04990	acyl-CoA synthetase protein, putative, expressed	The expression level of H21 statistically close to T44
MSTRG.16404	LOC_Os03g61150	expressed protein	The expression level of H21 statistically close to T44
MSTRG.5616			The expression level of H21 statistically close to T44
MSTRG.16935	LOC_Os04g13220	ABC transporter family protein, putative, expressed	The expression level of H21 statistically close to T44
MSTRG.20397	LOC_Os05g25640	cytochrome P450, putative, expressed	The expression level of H21 statistically close to T44
MSTRG.15779	LOC_Os03g52160	regulatory protein, putative, expressed	The expression level of H21 statistically close to T44
MSTRG.6973	LOC_Os11g35710	cycloartenol synthase, putative, expressed	The expression level of H21 statistically close to T44
MSTRG.28834	LOC_Os08g43450	MYB-like protein 1, putative, expressed	The expression level of H21 statistically close to T44
MSTRG.16333			The expression level of H21 statistically close to T44
MSTRG.29525			The expression level of H21 statistically close to T44
MSTRG.27159	LOC_Os08g06890	expressed protein	The expression level of H21 statistically close to T44
MSTRG.13640			The expression level of H21 statistically close to T44
MSTRG.7360	LOC_Os11g42570		The expression level of H21 statistically close to T44
MSTRG.259	LOC_Os01g03630	multicopper oxidase domain containing protein, expressed	The expression level of H21 statistically close to T44
MSTRG.5619			The expression level of H21 statistically close to T44
MSTRG.6461			The expression level of H21 statistically close to T44
MSTRG.2415			The expression level of H21 statistically close to T44
MSTRG.9119	LOC_Os12g37260	lipoxygenase 2.1, chloroplast precursor, putative, expressed	The expression level of H21 statistically close to T44

MSTRG.2857	LOC_Os01g56270	transposon protein, putative, CACTA, En/Spm sub-class, expressed	The expression level of H21 statistically close to T44
MSTRG.2600	LOC_Os01g52740	expressed protein	The expression level of H21 statistically close to T44
MSTRG.25172	LOC_Os07g13190		The expression level of H21 statistically close to T44
MSTRG.26947	LOC_Os08g03630	acyl-activating enzyme 14, putative, expressed	The expression level of H21 statistically close to T44
MSTRG.6742	LOC_Os11g29380	MCM2 - Putative minichromosome maintenance MCM complex subunit 2, expressed	The expression level of H21 statistically close to T44
MSTRG.16478	LOC_Os03g62230	ZOS3-24 - C2H2 zinc finger protein, expressed	The expression level of H21 statistically close to T44
MSTRG.21039	LOC_Os05g38219	expressed protein	The expression level of H21 statistically close to T44
MSTRG.22800	LOC_Os06g15990	aldehyde dehydrogenase, putative, expressed	The expression level of H21 statistically close to T44
MSTRG.13772	LOC_Os03g11760	polygalacturonase, putative, expressed	The expression level of H21 statistically close to T44
MSTRG.27146	LOC_Os08g06790	expressed protein	The expression level of H21 statistically close to T44
MSTRG.15545	LOC_Os03g48310	plasma membrane ATPase, putative, expressed	The expression level of H21 statistically close to T44
MSTRG.28968	LOC_Os08g45170	carboxyl-terminal peptidase, putative, expressed	The expression level of H21 statistically close to T44
MSTRG.16603	LOC_Os03g64260	AP2 domain containing protein, expressed	The expression level of H21 statistically close to T44
MSTRG.13053	LOC_Os03g01880	possible lysine decarboxylase domain containing protein, expressed	The expression level of H21 statistically close to T44
MSTRG.1425	LOC_Os01g27750	bifunctional 3-dehydroquinate dehydratase/shikimate dehydrogenase,chloroplast precursor, putative, expressed	The expression level of H21 statistically close to T44
MSTRG.21402	LOC_Os05g44210	trehalose-6-phosphate synthase, putative, expressed	The expression level of H21 statistically close to T44
MSTRG.19063	LOC_Os04g54810	beta-D-xylosidase, putative, expressed	The expression level of H21 statistically close to T44
MSTRG.5393	LOC_Os10g39140	flavonol synthase/flavanone 3-hydroxylase, putative, expressed	The expression level of H21 statistically close to T44
MSTRG.23489			The expression level of H21 statistically close to T44
MSTRG.9210	LOC_Os12g38920	heparanase-like protein precursor, putative, expressed	The expression level of H21 statistically close to T44
MSTRG.18569	LOC_Os04g47310	expressed protein	The expression level of H21 statistically close to T44
MSTRG.27535	LOC_Os08g14860	cytochrome b-c1 complex subunit 7, putative, expressed	The expression level of H21 statistically close to T44
MSTRG.1555	LOC_Os01g32830	expressed protein	The expression level of H21 statistically close to T44
MSTRG.843	LOC_Os01g13520	auxin response factor 1, putative, expressed	The expression level of H21 statistically close to T44
MSTRG.17848			The expression level of H21 statistically close to T44
MSTRG.20089	LOC_Os05g13940	retrotransposon protein, putative, unclassified, expressed	The expression level of H21 statistically close to T44
MSTRG.25145	LOC_Os07g12490	KH domain containing protein, putative, expressed	The expression level of H21 statistically close to T44
MSTRG.12478	LOC_Os02g51890	RNA recognition motif containing protein, putative, expressed	The expression level of H21 statistically close to T44
MSTRG.11876	LOC_Os02g42910	expressed protein	The expression level of H21 statistically close to T44
MSTRG.11245	LOC_Os02g32280	expressed protein	The expression level of H21 statistically close to T44
MSTRG.10209	LOC_Os02g09150	inorganic H+ pyrophosphatase, putative, expressed	The expression level of H21 statistically close to T44
MSTRG.13827	LOC_Os03g12414	cyclin, putative, expressed	The expression level of H21 statistically close to T44
MSTRG.8380	LOC_Os12g17600	ribulose bisphosphate carboxylase small chain, chloroplast precursor, putative, expressed	The expression level of H21 statistically close to T44
MSTRG.7014	LOC_Os11g36930		The expression level of H21 statistically close to T44
MSTRG.20909	LOC_Os05g35410	potassium channel AKT2/3, putative, expressed	The expression level of H21 statistically close to T44
MSTRG.17840	LOC_Os04g35420	helicase conserved C-terminal domain containing protein, expressed	The expression level of H21 statistically close to T44
MSTRG.15010	LOC_Os03g37490	MATE efflux family protein, putative, expressed	The expression level of H21 statistically close to T44
MSTRG.16919	LOC_Os04g12460	Leucine Rich Repeat family protein, expressed	The expression level of H21 statistically close to T44
MSTRG.9873	LOC_Os02g04630	sodium/calcium exchanger protein, putative, expressed	The expression level of H21 statistically close to T44
MSTRG.726	LOC_Os01g11850	expressed protein	The expression level of H21 statistically close to T44
MSTRG.28776	LOC_Os08g42600	retinoblastoma-related protein-like, putative, expressed	The expression level of H21 statistically close to T44
MSTRG.2003	LOC_Os01g42540		The expression level of H21 statistically close to T44
MSTRG.6672	LOC_Os11g26570	dehydrin, putative, expressed	The expression level of H21 statistically close to T44
MSTRG.26785	LOC_Os07g49290	PHD finger family protein, putative, expressed	The expression level of H21 statistically close to T44
MSTRG.30450	LOC_Os09g37540	uncharacterized protein PA4923, putative, expressed	The expression level of H21 statistically close to T44
MSTRG.12716	LOC_Os02g55410	MCM5 - Putative minichromosome maintenance MCM complex subunit 5, expressed	The expression level of H21 statistically close to T44
MSTRG.1196	LOC_Os01g21120	AP2 domain containing protein, expressed	The expression level of H21 statistically close to T44
MSTRG.24916	LOC_Os07g08500	C-5 cytosine-specific DNA methylase, putative, expressed	The expression level of H21 statistically close to T44

MSTRG.24211	LOC_Os06g47470	TKL_IRAK_DUF26-lh.2 - DUF26 kinases have homology to DUF26 containing loci, expressed	The expression level of H21 statistically close to T44
MSTRG.21483	LOC_Os05g45860	glucan endo-1,3-beta-glucosidase precursor, putative, expressed	The expression level of H21 statistically close to T44
MSTRG.10180	LOC_Os02g08330	gp176, putative, expressed	The expression level of H21 statistically close to T44
MSTRG.12300	LOC_Os02g49260	transporter-related, putative, expressed	The expression level of H21 statistically close to T44
MSTRG.4327	LOC_Os10g09200	expressed protein	The expression level of H21 statistically close to T44
MSTRG.19628	LOC_Os05g04470	peroxidase precursor, putative, expressed	The expression level of H21 statistically close to T44
MSTRG.29649	LOC_Os09g22320	retrotransposon protein, putative, Ty3-gypsy subclass, expressed	The expression level of H21 statistically close to T44
MSTRG.8613	LOC_Os12g25090	expressed protein	The expression level of H21 statistically close to T44
MSTRG.27859	LOC_Os08g24930	expressed protein	The expression level of H21 statistically close to T44
MSTRG.25868	LOC_Os07g34680		The expression level of H21 statistically close to T44
MSTRG.16415	LOC_Os03g61280	OsMan05 - Endo-Beta-Mannanase, expressed	The expression level of H21 statistically close to T44
MSTRG.1434	LOC_Os01g28290	expressed protein	The expression level of H21 statistically close to T44
MSTRG.11654	LOC_Os02g38700		The expression level of H21 statistically close to T44
MSTRG.9209	LOC_Os12g38940	ZOS12-06 - C2H2 zinc finger protein, expressed	The expression level of H21 statistically close to T44
MSTRG.17839	LOC_Os04g35430	PHD-finger domain containing protein, expressed	The expression level of H21 statistically close to T44
MSTRG.28762	LOC_Os08g42410	transketolase, putative, expressed	The expression level of H21 statistically close to T44
MSTRG.28821	LOC_Os08g43290	LTPL44 - Protease inhibitor/seed storage/LTP family protein precursor, expressed	The expression level of H21 statistically close to T44
MSTRG.21505	LOC_Os05g46140		The expression level of H21 statistically close to T44
MSTRG.6872	LOC_Os11g33120	respiratory burst oxidase, putative, expressed	The expression level of H21 statistically close to T44
MSTRG.28052			The expression level of H21 statistically close to T44
MSTRG.28398	LOC_Os08g36490	kinetochore protein, putative, expressed	The expression level of H21 statistically close to T44
MSTRG.23298	LOC_Os06g29890	retrotransposon protein, putative, unclassified, expressed	The expression level of H21 statistically close to T44
MSTRG.28193	LOC_Os08g32710		The expression level of H21 statistically close to T44
MSTRG.9741	LOC_Os02g02970	expressed protein	The expression level of H21 statistically close to T44
MSTRG.27327	LOC_Os08g10730	expressed protein	The expression level of H21 statistically close to T44
MSTRG.30012	LOC_Os09g29710	beta-expansin precursor, putative, expressed	The expression level of H21 statistically close to T44
MSTRG.10756	LOC_Os02g19820	nodulin MtN3 family protein, putative, expressed	The expression level of H21 statistically close to T44
MSTRG.6531	LOC_Os11g19140	methyltransferase domain containing protein, expressed	The expression level of H21 statistically close to T44
MSTRG.3412	LOC_Os01g64660	fructose-1,6-bisphosphatase, putative, expressed	The expression level of H21 statistically close to T44
MSTRG.9888	LOC_Os02g04750	cycloartenol synthase, putative, expressed	The expression level of H21 statistically close to T44
MSTRG.11544	LOC_Os02g37480	glycine-rich cell wall structural protein precursor, putative, expressed	The expression level of H21 statistically close to T44
MSTRG.20376	LOC_Os05g25040	expressed protein	The expression level of H21 statistically close to T44
MSTRG.6513	LOC_Os11g17970	POT family protein, expressed	The expression level of H21 statistically close to T44
MSTRG.62	LOC_Os01g01920	HD domain containing protein 2, putative, expressed	The expression level of H21 statistically close to T44
MSTRG.16779	LOC_Os04g05650	expressed protein	The expression level of H21 statistically close to T44
MSTRG.25740	LOC_Os07g32380	protein phosphatase 2C, putative, expressed	The expression level of H21 statistically close to T44
MSTRG.23473	LOC_Os06g36090	ABC-2 type transporter, putative, expressed	The expression level of H21 statistically close to T44
MSTRG.6706	LOC_Os11g27795	expressed protein	The expression level of H21 statistically close to T44
MSTRG.6086	LOC_Os11g07450	zinc finger, C3HC4 type domain containing protein, expressed	The expression level of H21 statistically close to T44
MSTRG.30547	LOC_Os09g38990	subtilase, putative, expressed	The expression level of H21 statistically close to T44
MSTRG.21764	LOC_Os05g50020	expressed protein	The expression level of H21 statistically close to T44
MSTRG.12255	LOC_Os02g48670	slowmo homolog, putative, expressed	The expression level of H21 statistically close to T44
MSTRG.23696	LOC_Os06g40415	expressed protein	The expression level of H21 statistically close to T44
MSTRG.11119	LOC_Os02g29300	expressed protein	The expression level of H21 statistically close to T44
MSTRG.27022			The expression level of H21 statistically close to T44
MSTRG.845			The expression level of H21 statistically close to T44
MSTRG.14201	LOC_Os03g17890		The expression level of H21 statistically close to T44
MSTRG.1302	LOC_Os01g23770	OsMADS94 - MADS-box family gene with M-beta type-box, expressed	The expression level of H21 statistically close to T44
MSTRG.19298	LOC_Os04g58070	aspartic proteinase nepenthesin precursor, putative, expressed	The expression level of H21 statistically close to T44
MSTRG.19153	LOC_Os04g56160	plasma membrane ATPase, putative, expressed	The expression level of H21 statistically close to T44
MSTRG.12192	LOC_Os02g47650	universal stress protein domain containing protein, putative, expressed	The expression level of H21 statistically close to T44
MSTRG.21162	LOC_Os05g40230; LOC_Os05g40240	vacuolar ATP synthase subunit E, putative, expressed;expressed protein	The expression level of H21 statistically close to T44
MSTRG.16423	LOC_Os03g61500		The expression level of H21 statistically close to T44
MSTRG.2382	LOC_Os01g49290	WD repeat-containing protein, putative, expressed	The expression level of H21 statistically close to T44
MSTRG.27541	LOC_Os08g14880; LOC_Os08g14890	transposon protein, putative, unclassified, expressed;expressed protein	The expression level of H21 statistically close to T44

MSTRG.20722	LOC_Os05g32370	ATP-dependent RNA helicase, putative, expressed	The expression level of H21 statistically close to T44
MSTRG.27270	LOC_Os08g09700	OsFBX270 - F-box domain containing protein, expressed	The expression level of H21 statistically close to T44
MSTRG.23543	LOC_Os06g37320		The expression level of H21 statistically close to T44
MSTRG.16467	LOC_Os03g62080	mRNA-decapping enzyme, putative, expressed	The expression level of H21 statistically close to T44
MSTRG.26181	LOC_Os07g39850	expressed protein	The expression level of H21 statistically close to T44
MSTRG.3952	LOC_Os01g73040	CBS domain-containing protein, putative, expressed	The expression level of H21 statistically close to T44
MSTRG.21229	LOC_Os05g41390	cyclin, putative, expressed	The expression level of H21 statistically close to T44
MSTRG.28203	LOC_Os08g32920	dynamin-2B, putative, expressed	The expression level of H21 statistically close to T44
MSTRG.22352	LOC_Os06g07896	ribosomal protein S15 containing protein, expressed	The expression level of H21 statistically close to T44
MSTRG.6541			The expression level of H21 statistically close to T44
MSTRG.11797	LOC_Os02g41650; LOC_Os02g41680	phenylalanine ammonia-lyase, putative, expressed	The expression level of H21 statistically close to T44
MSTRG.6368	LOC_Os11g12740	peptide transporter PTR2, putative, expressed	The expression level of H21 statistically close to T44
MSTRG.6991	LOC_Os11g36390	RFC1 - Putative clamp loader of PCNA, replication factor C subunit 1, expressed	The expression level of H21 statistically close to T44
MSTRG.16693	LOC_Os04g03884	transposon protein, putative, CACTA, En/Spm sub-class, expressed	The expression level of H21 statistically close to T44
MSTRG.27517	LOC_Os08g14230	villin, putative, expressed	The expression level of H21 statistically close to T44
MSTRG.26699	LOC_Os07g48170	nucleotidyltransferase, putative, expressed	The expression level of H21 statistically close to T44
MSTRG.22451	LOC_Os06g09350	expressed protein	The expression level of H21 statistically close to T44
MSTRG.11798	LOC_Os02g41650		The expression level of H21 statistically close to T44
MSTRG.1077			The expression level of H21 statistically close to T44
MSTRG.9179	LOC_Os12g38430	SR repressor protein, putative, expressed	The expression level of H21 statistically close to T44
MSTRG.21084	LOC_Os05g38850	MCM8 - Putative minichromosome maintenance MCM family subunit 8, expressed	The expression level of H21 statistically close to T44
MSTRG.13193	LOC_Os03g03724	expressed protein	The expression level of H21 statistically close to T44
MSTRG.3467	LOC_Os01g65460	beta-galactosidase precursor, putative, expressed	The expression level of H21 statistically close to T44
MSTRG.3195	LOC_Os01g61080	WRKY24, expressed	The expression level of H21 statistically close to T44
MSTRG.2891	LOC_Os01g56710		The expression level of H21 statistically close to T44
MSTRG.16250	LOC_Os03g59090	retrotransposon protein, putative, unclassified, expressed	The expression level of H21 statistically close to T44
MSTRG.27076	LOC_Os08g05870	oxysterol-binding protein-related protein 6, putative, expressed	The expression level of H21 statistically close to T44
MSTRG.16918			The expression level of H21 statistically close to T44
MSTRG.1276	LOC_Os01g22630	ECT protein, putative, expressed	The expression level of H21 statistically close to T44
MSTRG.13940	LOC_Os03g14170	3-ketoacyl-CoA synthase, putative, expressed	The expression level of H21 statistically close to T44
MSTRG.27263	LOC_Os08g09450	OsFBL41 - F-box domain and LRR containing protein, expressed	The expression level of H21 statistically close to T44
MSTRG.18157	LOC_Os04g40870	alveolar soft part sarcoma chromosome region, candidate 1, putative, expressed	The expression level of H21 statistically close to T44
MSTRG.6206	LOC_Os11g09979	expressed protein	The expression level of H21 statistically close to T44
MSTRG.22722	LOC_Os06g14030	potassium channel SKOR, putative, expressed	The expression level of H21 statistically close to T44
MSTRG.7459	LOC_Os11g44780		The expression level of H21 statistically close to T44
MSTRG.29148	LOC_Os09g06680	WD domain, G-beta repeat domain containing protein, expressed	The expression level of H21 statistically close to T44
MSTRG.5077	LOC_Os10g33930	DUF1336 domain containing protein, expressed	The expression level of H21 statistically close to T44
MSTRG.10283	LOC_Os02g10390	chlorophyll A-B binding protein, putative, expressed	The expression level of H21 statistically close to T44
MSTRG.1379	LOC_Os01g25920	expressed protein	The expression level of H21 statistically close to T44
MSTRG.5459	LOC_Os10g40200		The expression level of H21 statistically close to T44
MSTRG.18054	LOC_Os04g39250		The expression level of H21 statistically close to T44
MSTRG.13938	LOC_Os03g14140	POEI16 - Pollen Ole e I allergen and extensin family protein precursor, expressed	The expression level of H21 statistically close to T44
MSTRG.26617	LOC_Os07g46940	sex determination protein tasselseed-2, putative, expressed	The expression level of H21 statistically close to T44
MSTRG.10340	LOC_Os02g11070	3-ketoacyl-CoA synthase, putative, expressed	The expression level of H21 statistically close to T44
MSTRG.24593	LOC_Os07g02930	OsFBX210 - F-box domain containing protein, expressed	The expression level of H21 statistically close to T44
MSTRG.2212	LOC_Os01g46470		The expression level of H21 statistically close to T44
MSTRG.10	LOC_Os01g01170	expressed protein	The expression level of H21 statistically close to T44
MSTRG.82	LOC_Os01g02160	hydroxyproline-rich glycoprotein family protein, putative, expressed	The expression level of H21 statistically close to T44
MSTRG.27266	LOC_Os08g09520	transposon protein, putative, unclassified, expressed	The expression level of H21 statistically close to T44
MSTRG.8057	LOC_Os12g08260	dehydrogenase E1 component domain containing protein, expressed	The expression level of H21 statistically close to T44
MSTRG.10001	LOC_Os02g05980		The expression level of H21 statistically close to T44
MSTRG.23152	LOC_Os06g25500	expressed protein	The expression level of H21 statistically close to T44
MSTRG.28169	LOC_Os08g32390		The expression level of H21 statistically close to T44

MSTRG.16885	LOC_Os04g10650	CDT1A - Putative DNA replication initiation protein, expressed	The expression level of H21 statistically close to T44
MSTRG.13740	LOC_Os03g11400	targeting protein-related, putative, expressed	The expression level of H21 statistically close to T44
MSTRG.19646	LOC_Os05g04700	OsRCI2-6 - Hydrophobic protein LTI6B, expressed	The expression level of H21 statistically close to T44
MSTRG.5227			The expression level of H21 statistically close to T44
MSTRG.10098	LOC_Os02g07160	glyoxalase family protein, putative, expressed	The expression level of H21 statistically close to T44
MSTRG.30347	LOC_Os09g36200	senescence-inducible chloroplast stay-green protein 1, putative, expressed	The expression level of H21 statistically close to T44
MSTRG.11696	LOC_Os02g39470	cyclin, N-terminal domain containing protein, expressed	The expression level of H21 statistically close to T44
MSTRG.13465	LOC_Os03g07810	expressed protein	The expression level of H21 statistically close to T44
MSTRG.24574	LOC_Os07g02470	expressed protein	The expression level of H21 statistically close to T44
MSTRG.23438	LOC_Os06g34950	expressed protein	The expression level of H21 statistically close to T44
MSTRG.24688	LOC_Os07g04980; LOC_Os07g04990	expressed protein;oxidoreductase, aldo/keto reductase family protein, putative, expressed	The expression level of H21 statistically close to T44
MSTRG.28210	LOC_Os08g33100	core histone H2A/H2B/H3/H4, putative, expressed	The expression level of H21 statistically close to T44
MSTRG.302	LOC_Os01g04950	peptide transporter PTR2, putative, expressed	The expression level of H21 statistically close to T44
MSTRG.10072	LOC_Os02g06830	expressed protein	The expression level of H21 statistically close to T44
MSTRG.9452	LOC_Os12g42160	kinesin motor domain containing protein, putative, expressed	The expression level of H21 statistically close to T44
MSTRG.26841	LOC_Os08g01680	WD domain, G-beta repeat domain containing protein, expressed	The expression level of H21 statistically close to T44
MSTRG.15544	LOC_Os03g48300	histidine acid phosphatase, putative, expressed	The expression level of H21 statistically close to T44
MSTRG.15178	LOC_Os03g40920	expressed protein	The expression level of H21 statistically close to T44
MSTRG.24430	LOC_Os06g50910	Phosphatidylinositol kinase and FAT containing domain protein, putative, expressed	The expression level of H21 statistically close to T44
MSTRG.21106	LOC_Os05g39240	ammonium transporter protein, putative, expressed	The expression level of H21 statistically close to T44
MSTRG.2536	LOC_Os01g51620	KRR1 small subunit processome component, putative, expressed	The expression level of H21 statistically close to T44
MSTRG.23100	LOC_Os06g23870	acyl-CoA dehydrogenase domain protein, putative, expressed	The expression level of H21 statistically close to T44
MSTRG.11201	LOC_Os02g30800	POLE1 - Putative DNA polymerase epsilon catalytic subunit, expressed	The expression level of H21 statistically close to T44
MSTRG.22046	LOC_Os06g03860	uncharacterized membrane protein, putative, expressed	The expression level of H21 statistically close to T44
MSTRG.12635	LOC_Os02g54310	expressed protein	The expression level of H21 statistically close to T44
MSTRG.4685	LOC_Os10g24094; LOC_Os10g24100	expressed protein	The expression level of H21 statistically close to T44
MSTRG.13377	LOC_Os03g06460	type I inositol-1,4,5-trisphosphate 5-phosphatase, putative, expressed	The expression level of H21 statistically close to T44
MSTRG.26744	LOC_Os07g48820	transcription factor, putative, expressed	The expression level of H21 statistically close to T44
MSTRG.12116	LOC_Os02g46650	ubiquitin carboxyl-terminal hydrolase domain containing protein, expressed	The expression level of H21 statistically close to T44
MSTRG.29378	LOC_Os09g14660		The expression level of H21 statistically close to T44
MSTRG.11095	LOC_Os02g28850	Kinesin motor domain domain containing protein, expressed	The expression level of H21 statistically close to T44
MSTRG.27570	LOC_Os08g15590; LOC_Os08g15600	Leucine rich repeat N-terminal domain containing protein, putative, expressed;stomatin-like protein 2, putative, expressed	The expression level of H21 statistically close to T44
MSTRG.20525	LOC_Os05g28180	AMP deaminase, putative, expressed	The expression level of H21 statistically close to T44
MSTRG.15536	LOC_Os03g48180	peptide transporter PTR2, putative, expressed	The expression level of H21 statistically close to T44
MSTRG.20905	LOC_Os05g35266; LOC_Os05g35274	galactosyltransferase, putative, expressed;RNA recognition motif containing protein, putative, expressed	The expression level of H21 statistically close to T44
MSTRG.15097	LOC_Os03g39230	OTU-like cysteine protease family protein, putative, expressed	The expression level of H21 statistically close to T44
MSTRG.24734	LOC_Os07g05620	CAMK_KIN1/SNF1/Nim1_like.28 - CAMK includes calcium/calmodulin depedent protein kinases, expressed	The expression level of H21 statistically close to T44
MSTRG.14413	LOC_Os03g20870	zinc finger, C3HC4 type domain containing protein, expressed	The expression level of H21 statistically close to T44
MSTRG.1102	LOC_Os01g18240		The expression level of H21 statistically close to T44
MSTRG.7391	LOC_Os11g42880	expressed protein	The expression level of H21 statistically close to T44
MSTRG.16920	LOC_Os04g12480	pumilio-family RNA binding repeat containing protein, expressed	The expression level of H21 statistically close to T44
MSTRG.13566	LOC_Os03g08999	dehydrogenase, putative, expressed	The expression level of H21 statistically close to T44
MSTRG.12041	LOC_Os02g45530	HOTHEAD precursor, putative, expressed	The expression level of H21 statistically close to T44
MSTRG.27273	LOC_Os08g09715; LOC_Os08g09720; LOC_Os08g09730	F-box domain containing protein, expressed;OsFBX272 - F-box domain containing protein, expressed;OsFBX273 - F-box domain containing protein, expressed	The expression level of H21 statistically close to T44
MSTRG.15001	LOC_Os03g37140	expressed protein	The expression level of H21 statistically close to T44

MSTRG.19268	LOC_Os04g57730	uracil-DNA glycosylase, putative, expressed	The expression level of H21 statistically close to T44
MSTRG.14907	LOC_Os03g31944	expressed protein	The expression level of H21 statistically close to T44
MSTRG.22404	LOC_Os06g08560	multidrug resistance-associated protein 11, putative, expressed	The expression level of H21 statistically close to T44
MSTRG.30163	LOC_Os09g32510	BHLH transcription factor, putative, expressed	The expression level of H21 statistically close to T44
MSTRG.18373	LOC_Os04g44220	MIF4G domain containing protein, putative, expressed	The expression level of H21 statistically close to T44
MSTRG.3560	LOC_Os01g66700	beta-hexosaminidase precursor, putative, expressed	The expression level of H21 statistically close to T44
MSTRG.26909	LOC_Os08g02670	ATP binding protein, putative, expressed	The expression level of H21 statistically close to T44
MSTRG.13891	LOC_Os03g13520	endonuclease/exonuclease/phosphatase family domain containing protein, expressed	The expression level of H21 statistically close to T44
MSTRG.18325	LOC_Os04g43200	caleosin related protein, putative, expressed	The expression level of H21 statistically close to T44
MSTRG.20165	LOC_Os05g15770	glycosyl hydrolase, putative, expressed	The expression level of H21 statistically close to T44
MSTRG.19125	LOC_Os04g55650	oryzain alpha chain precursor, putative, expressed	The expression level of H21 statistically close to T44
MSTRG.12585	LOC_Os02g53450		The expression level of H21 statistically close to T44
MSTRG.12839	LOC_Os02g57080	serine/threonine-protein kinase, putative, expressed	The expression level of H21 statistically close to T44
MSTRG.29374	LOC_Os09g14550	RNA recognition motif containing protein, putative, expressed	The expression level of H21 statistically close to T44
MSTRG.14612	LOC_Os03g24390		The expression level of H21 statistically close to T44
MSTRG.13130	LOC_Os03g02780	Core histone H2A/H2B/H3/H4 domain containing protein, putative, expressed	The expression level of H21 statistically close to T44
MSTRG.7215	LOC_Os11g39810		The expression level of H21 statistically close to T44
MSTRG.24702	LOC_Os07g05145	expressed protein	The expression level of H21 statistically close to T44
MSTRG.7783	LOC_Os12g03870	major facilitator superfamily antiporter, putative, expressed	The expression level of H21 statistically close to T44
MSTRG.30298	LOC_Os09g35000	flap endonuclease, putative, expressed	The expression level of H21 statistically close to T44
MSTRG.1234	LOC_Os01g21940		The expression level of H21 statistically close to T44
MSTRG.9766	LOC_Os02g03320	plant neutral invertase domain containing protein, expressed	The expression level of H21 statistically close to T44
MSTRG.13535	LOC_Os03g08570	amine oxidase, flavin-containing, domain containing protein, expressed	The expression level of H21 statistically close to T44
MSTRG.6888			The expression level of H21 statistically close to T44
MSTRG.9213	LOC_Os12g38960	ZOS12-07 - C2H2 zinc finger protein, expressed	The expression level of H21 statistically close to T44
MSTRG.8309	LOC_Os12g15314	staphylococcal nuclease homologue, putative, expressed	The expression level of H21 statistically close to T44
MSTRG.22861	LOC_Os06g17290	phosphatidylinositol 3- and 4-kinase family protein, putative, expressed	The expression level of H21 statistically close to T44
MSTRG.8507			The expression level of H21 statistically close to T44
MSTRG.19980	LOC_Os05g10670	zinc finger CCCH type family protein, putative, expressed	The expression level of H21 statistically close to T44
MSTRG.15020	LOC_Os03g37864	expressed protein	The expression level of H21 statistically close to T44
MSTRG.3821	LOC_Os01g70840		The expression level of H21 statistically close to T44
MSTRG.4150	LOC_Os10g03910	OsFBX354 - F-box domain containing protein, expressed	The expression level of H21 statistically close to T44
MSTRG.6065	LOC_Os11g07050	FHA domain containing protein, putative, expressed	The expression level of H21 statistically close to T44
MSTRG.21774	LOC_Os05g50120	CGMC_MAPKCMGC_2.3 - CGMC includes CDA, MAPK, GSK3, and CLKC kinases, expressed	The expression level of H21 statistically close to T44
MSTRG.2059	LOC_Os01g43350	protein kinase family protein, putative, expressed	The expression level of H21 statistically close to T44
MSTRG.14713	LOC_Os03g26480	expressed protein	The expression level of H21 statistically close to T44
MSTRG.20637	LOC_Os05g30790	CW-type Zinc Finger, putative, expressed	The expression level of H21 statistically close to T44
MSTRG.14786			The expression level of H21 statistically close to T44
MSTRG.25011	LOC_Os07g10090	expressed protein	The expression level of H21 statistically close to T44
MSTRG.18964	LOC_Os04g53360	expressed protein	The expression level of H21 statistically close to T44
MSTRG.10488	LOC_Os02g13580	kinesin motor domain containing protein, expressed	The expression level of H21 statistically close to T44
MSTRG.21395	LOC_Os05g44120	expressed protein	The expression level of H21 statistically close to T44
MSTRG.29671	LOC_Os09g22540	transcription factor jumonji, putative, expressed	The expression level of H21 statistically close to T44
MSTRG.3595	LOC_Os01g67260	expressed protein	The expression level of H21 statistically close to T44
MSTRG.4370	LOC_Os10g10580		The expression level of H21 statistically close to T44
MSTRG.889	LOC_Os01g14310		The expression level of H21 statistically close to T44
MSTRG.19549	LOC_Os05g02880	splicing factor, arginine/serine-rich 7, putative, expressed	The expression level of H21 statistically close to T44
MSTRG.19524	LOC_Os05g02560	expressed protein	The expression level of H21 statistically close to T44
MSTRG.18656	LOC_Os04g48750		The expression level of H21 statistically close to T44
MSTRG.15983	LOC_Os03g55660; LOC_Os03g55670	nucleoporin, putative, expressed;expressed protein	The expression level of H21 statistically close to T44
MSTRG.2521	LOC_Os01g51360	lipase, putative, expressed	The expression level of H21 statistically close to T44
MSTRG.18867	LOC_Os04g52280	dehydrogenase, putative, expressed	The expression level of H21 statistically close to T44
MSTRG.11463	LOC_Os02g35940	gibberellin receptor GID1L2, putative, expressed	The expression level of H21 statistically close to T44
MSTRG.1388	LOC_Os01g26120	membrane associated DUF588 domain containing protein, putative, expressed	The expression level of H21 statistically close to T44

MSTRG.438	LOC_Os01g07330	expressed protein	The expression level of H21 statistically close to T44
MSTRG.15792	LOC_Os03g52380	PIII5 - Proteinase inhibitor II family protein precursor, expressed	The expression level of H21 statistically close to T44
MSTRG.8074	LOC_Os12g08810	VTC2, putative, expressed	The expression level of H21 statistically close to T44
MSTRG.4155	LOC_Os10g04050	transposon protein, putative, CACTA, En/Spm sub-class, expressed	The expression level of H21 statistically close to T44
MSTRG.6115	LOC_Os11g08020	ankyrin repeat family protein, putative, expressed	The expression level of H21 statistically close to T44
MSTRG.16135	LOC_Os03g57545	expressed protein	The expression level of H21 statistically close to T44
MSTRG.19074	LOC_Os04g54940		The expression level of H21 statistically close to T44
MSTRG.1202	LOC_Os01g21160	2-oxo acid dehydrogenases acyltransferase domain containing protein, expressed	The expression level of H21 statistically close to T44
MSTRG.24501	LOC_Os06g51530;<br>LOC_Os06g51524	expressed protein;ribosomal protein L29, putative, expressed	The expression level of H21 statistically close to T44
MSTRG.18431	LOC_Os04g45010	OsPLIM2b - LIM domain protein, putative actin-binding protein and transcription factor, expressed	The expression level of H21 statistically close to T44
MSTRG.4932	LOC_Os10g30820	expressed protein	The expression level of H21 statistically close to T44
MSTRG.25227			The expression level of H21 statistically close to T44
MSTRG.6619	LOC_Os11g24070	LTPL10 - Protease inhibitor/seed storage/LTP family protein precursor, expressed	The expression level of H21 statistically close to T44
MSTRG.11260	LOC_Os02g32504	heparan-alpha-glucosaminide N-acetyltransferase, putative, expressed	The expression level of H21 statistically close to T44
MSTRG.22497			The expression level of H21 statistically close to T44
MSTRG.8006	LOC_Os12g07540	growth regulator related protein, putative, expressed	The expression level of H21 statistically close to T44
MSTRG.4165	LOC_Os10g04130	expressed protein	The expression level of H21 statistically close to T44
MSTRG.21005	LOC_Os05g37390	G10 protein, putative, expressed	The expression level of H21 statistically close to T44
MSTRG.2387	LOC_Os01g49320	glycosyl hydrolase, putative, expressed	The expression level of H21 statistically close to T44
MSTRG.24650	LOC_Os07g04220	wound and phytochrome signaling involved receptor like kinase, putative, expressed	The expression level of H21 statistically close to T44
MSTRG.18549	LOC_Os04g46940	copper-transporting ATPase 3, putative, expressed	The expression level of H21 statistically close to T44
MSTRG.18999	LOC_Os04g53800	leucoanthocyanidin reductase, putative, expressed	The expression level of H21 statistically close to T44
MSTRG.29789	LOC_Os09g25740	expressed protein	The expression level of H21 statistically close to T44
MSTRG.27137	LOC_Os08g06620;<br>LOC_Os08g06630	DNA polymerase delta subunit 4 family, putative, expressed	The expression level of H21 statistically close to T44
MSTRG.12524	LOC_Os02g52490;<br>LOC_Os02g52510	expressed protein;SNF2 family N-terminal domain containing protein, expressed	The expression level of H21 statistically close to T44
MSTRG.7838			The expression level of H21 statistically close to T44
MSTRG.11956	LOC_Os02g44102;<br>LOC_Os02g44104	remorin C-terminal domain containing protein, putative, expressed;F-box family protein, putative, expressed	The expression level of H21 statistically close to T44
MSTRG.15211	LOC_Os03g42040	HEAT repeat family protein, putative, expressed	The expression level of H21 statistically close to T44
MSTRG.2374	LOC_Os01g49200	microtubule associated protein, putative, expressed	The expression level of H21 statistically close to T44
MSTRG.7837	LOC_Os12g04790	expressed protein	The expression level of H21 statistically close to T44
MSTRG.19822			The expression level of H21 statistically close to T44
MSTRG.7631	LOC_Os12g01530		The expression level of H21 statistically close to T44
MSTRG.22230	LOC_Os06g06080	serine esterase family protein, putative, expressed	The expression level of H21 statistically close to T44
MSTRG.30032	LOC_Os09g30070	expressed protein	The expression level of H21 statistically close to T44
MSTRG.3774	LOC_Os01g70240	expressed protein	The expression level of H21 statistically close to T44
MSTRG.22552	LOC_Os06g11210	12-oxophytodienoate reductase, putative, expressed	The expression level of H21 statistically close to T44
MSTRG.7495	LOC_Os11g45750	WRKY125, expressed	The expression level of H21 statistically close to T44
MSTRG.26391	LOC_Os07g43410	expressed protein	The expression level of H21 statistically close to T44
MSTRG.11427	LOC_Os02g35230	expressed protein	The expression level of H21 statistically close to T44
MSTRG.4744	LOC_Os10g26280	ORC3 - Putative origin recognition complex subunit 3, expressed	The expression level of H21 statistically close to T44
MSTRG.21615	LOC_Os05g47870	expressed protein	The expression level of H21 statistically close to T44
MSTRG.381	LOC_Os01g05960		The expression level of H21 statistically close to T44
MSTRG.5913			The expression level of H21 statistically close to T44
MSTRG.7253	LOC_Os11g40500	OsSigP7 - Putative Type I Signal Peptidase homologue; employs a putative Ser/Lys catalytic dyad, expressed	The expression level of H21 statistically close to T44
MSTRG.6648	LOC_Os11g25260	nucleoside-triphosphatase, putative, expressed	The expression level of H21 statistically close to T44
MSTRG.23628	LOC_Os06g38960	expressed protein	The expression level of H21 statistically close to T44
MSTRG.28783	LOC_Os08g42720	solute carrier family 35 member F1, putative, expressed	The expression level of H21 statistically close to T44
MSTRG.3799	LOC_Os01g70550	heparan-alpha-glucosaminide N-acetyltransferase, putative, expressed	The expression level of H21 statistically close to T44
MSTRG.26729	LOC_Os07g48630	ethylene-insensitive 3, putative, expressed	The expression level of H21 statistically close to T44
MSTRG.9815	LOC_Os02g03870	periplasmic beta-glucosidase precursor, putative, expressed	The expression level of H21 statistically close to T44
MSTRG.22763	LOC_Os06g14780	expressed protein	The expression level of H21 statistically close to T44

MSTRG.22571	LOC_Os06g11520	LMBR1 integral membrane protein, putative, expressed	The expression level of H21 statistically close to T44
MSTRG.9966	LOC_Os02g05300		The expression level of H21 statistically close to T44
MSTRG.21585	LOC_Os05g47490	MDR-like ABC transporter, putative, expressed	The expression level of H21 statistically close to T44
MSTRG.8972	LOC_Os12g34840	rhoGAP domain containing protein, expressed	The expression level of H21 statistically close to T44
MSTRG.14148	LOC_Os03g17164	kinesin-related protein, putative, expressed	The expression level of H21 statistically close to T44
MSTRG.18674	LOC_Os04g48970	transposon protein, putative, unclassified, expressed	The expression level of H21 statistically close to T44
MSTRG.28111	LOC_Os08g31350		The expression level of H21 statistically close to T44
MSTRG.28145	LOC_Os08g31910	expressed protein	The expression level of H21 statistically close to T44
MSTRG.23480	LOC_Os06g36180	amino acid transporter, putative, expressed	The expression level of H21 statistically close to T44
MSTRG.6133	LOC_Os11g08330	POLD1 - Putative DNA polymerase delta catalytic subunit, expressed	The expression level of H21 statistically close to T44
MSTRG.30519	LOC_Os09g38550	protein phosphatase 2C, putative, expressed	The expression level of H21 statistically close to T44
MSTRG.7683	LOC_Os12g02390;<br>LOC_Os12g02385	outer membrane protein, OMP85 family, putative, expressed;vacuolar-sorting receptor precursor, putative, expressed	The expression level of H21 statistically close to T44
MSTRG.3719	LOC_Os01g69100	expressed protein	The expression level of H21 statistically close to T44
MSTRG.13406	LOC_Os03g06920	DRD1, putative, expressed	The expression level of H21 statistically close to T44
MSTRG.1343	LOC_Os01g24940	poly synthetase 2-A, putative, expressed	The expression level of H21 statistically close to T44
MSTRG.14480	LOC_Os03g21950	fumarate hydratase, mitochondrial precursor, putative, expressed	The expression level of H21 statistically close to T44
MSTRG.22686	LOC_Os06g13210	peptide transporter PTR2, putative, expressed	The expression level of H21 statistically close to T44
MSTRG.29478	LOC_Os09g17152	OsFBX319 - F-box domain containing protein, expressed	The expression level of H21 statistically close to T44
MSTRG.9568	LOC_Os12g44150	plasma membrane ATPase, putative, expressed	The expression level of H21 statistically close to T44
MSTRG.22622	LOC_Os06g12320	transmembrane amino acid transporter protein, putative, expressed	The expression level of H21 statistically close to T44
MSTRG.3865	LOC_Os01g71630	expressed protein	The expression level of H21 statistically close to T44
MSTRG.17183	LOC_Os04g20180	expressed protein	The expression level of H21 statistically close to T44
MSTRG.25858	LOC_Os07g34550	retrotransposon protein, putative, unclassified, expressed	The expression level of H21 statistically close to T44
MSTRG.3988	LOC_Os01g73580	glycosyl hydrolases, putative, expressed	The expression level of H21 statistically close to T44
MSTRG.7856			The expression level of H21 statistically close to T44
MSTRG.11625	LOC_Os02g38840	glucose-6-phosphate 1-dehydrogenase, cytoplasmic isoform, putative, expressed	The expression level of H21 statistically close to T44
MSTRG.4721	LOC_Os10g25290	ZIM domain containing protein, putative, expressed	The expression level of H21 statistically close to T44
MSTRG.20867	LOC_Os05g34580		The expression level of H21 statistically close to T44
MSTRG.19215	LOC_Os04g57130		The expression level of H21 statistically close to T44
MSTRG.25192	LOC_Os07g13634	cytokinin-N-glucosyltransferase 1, putative, expressed	The expression level of H21 statistically close to T44
MSTRG.21291	LOC_Os05g42350	ferredoxin--nitrite reductase, putative, expressed	The expression level of H21 statistically close to T44
MSTRG.13175	LOC_Os03g03520	expressed protein	The expression level of H21 statistically close to T44
MSTRG.4457	LOC_Os10g15240	retrotransposon protein, putative, Ty3-gypsy subclass, expressed	The expression level of H21 statistically close to T44
MSTRG.21076	LOC_Os05g38800	PWWP domain containing protein, expressed	The expression level of H21 statistically close to T44
MSTRG.5647	LOC_Os10g42820	early-responsive to dehydration protein-related, putative, expressed	The expression level of H21 statistically close to T44
MSTRG.29832	LOC_Os09g26540	expressed protein	The expression level of H21 statistically close to T44
MSTRG.537	LOC_Os01g08650	expressed protein	The expression level of H21 statistically close to T44
MSTRG.11888	LOC_Os02g43080		The expression level of H21 statistically close to T44
MSTRG.23741	LOC_Os06g41050	expressed protein	The expression level of H21 statistically close to T44
MSTRG.14441	LOC_Os03g21400;<br>LOC_Os03g21419	cytochrome P450, putative, expressed;retrotransposon protein, putative, Ty1-copia subclass, expressed	The expression level of H21 statistically close to T44
MSTRG.21109	LOC_Os05g39230	low photochemical bleaching 1 protein, putative, expressed	The expression level of H21 statistically close to T44
MSTRG.6492	LOC_Os11g16590	ATP-dependent Clp protease ATP-binding subunit clpA homolog CD4B,chloroplast precursor, putative, expressed	The expression level of H21 statistically close to T44
MSTRG.29931	LOC_Os09g28180	D-mannose binding lectin family protein, expressed	The expression level of H21 statistically close to T44
MSTRG.8067	LOC_Os12g08730	thioredoxin, putative, expressed	The expression level of H21 statistically close to T44
MSTRG.12390	LOC_Os02g50600	glycosyl transferase 8 domain containing protein, putative, expressed	The expression level of H21 statistically close to T44
MSTRG.18004			The expression level of H21 statistically close to T44
MSTRG.885	LOC_Os01g14180	expressed protein	The expression level of H21 statistically close to T44
MSTRG.4469	LOC_Os10g17100		The expression level of H21 statistically close to T44
MSTRG.24327	LOC_Os06g49150	exostosin, putative, expressed	The expression level of H21 statistically close to T44
MSTRG.9540	LOC_Os12g43640	receptor-like protein kinase HAIKU2 precursor, putative, expressed	The expression level of H21 statistically close to T44

MSTRG.26626	LOC_Os07g47040		The expression level of H21 statistically close to T44
MSTRG.21180	LOC_Os05g40700	transmembrane protein 56, putative, expressed	The expression level of H21 statistically close to T44
MSTRG.937	LOC_Os01g15210	expressed protein	The expression level of H21 statistically close to T44
MSTRG.28575	LOC_Os08g39830	ethylene-insensitive 3, putative, expressed	The expression level of H21 statistically close to T44
MSTRG.15950	LOC_Os03g55200	ORC5 - Putative origin recognition complex subunit 5, expressed	The expression level of H21 statistically close to T44
MSTRG.28412	LOC_Os08g36910	alpha-amylase precursor, putative, expressed	The expression level of H21 statistically close to T44
MSTRG.15028	LOC_Os03g38010	nuf2 family protein, expressed	The expression level of H21 statistically close to T44
MSTRG.26237	LOC_Os07g40730	SNF2 domain-containing protein, putative, expressed	The expression level of H21 statistically close to T44
MSTRG.11165	LOC_Os02g30060	3-oxoacyl-reductase, chloroplast precursor, putative, expressed	The expression level of H21 statistically close to T44
MSTRG.10344	LOC_Os02g11640; LOC_Os02g11660; LOC_Os02g11700	UDP-glucoronosyl and UDP-glucosyl transferase, putative, expressed;cytokinin-O-glucosyltransferase 3, putative, expressed	The expression level of H21 statistically close to T44
MSTRG.4702	LOC_Os10g25010	OsCML8 - Calmodulin-related calcium sensor protein, expressed	The expression level of H21 statistically close to T44
MSTRG.4479	LOC_Os10g17489	UDP-glucoronosyl and UDP-glucosyl transferase domain containing protein, expressed	The expression level of H21 statistically close to T44
MSTRG.8900	LOC_Os12g33180	pnn protein, putative, expressed	The expression level of H21 statistically close to T44
MSTRG.2125	LOC_Os01g44330	laccase precursor protein, putative, expressed	The expression level of H21 statistically close to T44
MSTRG.4905	LOC_Os10g30280	OsFBX386 - F-box domain containing protein, expressed	The expression level of H21 statistically close to T44
MSTRG.27173	LOC_Os08g07390	mla1, putative, expressed	The expression level of H21 statistically close to T44
MSTRG.12633	LOC_Os02g54280; LOC_Os02g54290	retrotransposon protein, putative, unclassified, expressed;BRUSHY 1, putative, expressed	The expression level of H21 statistically close to T44
MSTRG.24713			The expression level of H21 statistically close to T44
MSTRG.10101	LOC_Os02g07190	expressed protein	The expression level of H21 statistically close to T44
MSTRG.11528	LOC_Os02g37090	hydrolase, alpha/beta fold family domain containing protein, expressed	The expression level of H21 statistically close to T44
MSTRG.28761	LOC_Os08g42400	no apical meristem protein, putative, expressed	The expression level of H21 statistically close to T44
MSTRG.8415	LOC_Os12g18729	expressed protein	The expression level of H21 statistically close to T44
MSTRG.24784	LOC_Os07g06680	fasciclin domain containing protein, expressed	The expression level of H21 statistically close to T44
MSTRG.21563	LOC_Os05g46720	phosphatidylinositol transfer, putative, expressed	The expression level of H21 statistically close to T44
MSTRG.20830	LOC_Os05g34010	peptide transporter PTR2, putative, expressed	The expression level of H21 statistically close to T44
MSTRG.11805	LOC_Os02g41840	DUF584 domain containing protein, putative, expressed	The expression level of H21 statistically close to T44
MSTRG.27537	LOC_Os08g14990		The expression level of H21 statistically close to T44
MSTRG.24649	LOC_Os07g04200	bacterial transferase hexapeptide domain containing protein, expressed	The expression level of H21 statistically close to T44
MSTRG.11409	LOC_Os02g35020	glycosyl transferase, putative, expressed	The expression level of H21 statistically close to T44
MSTRG.20040	LOC_Os05g11980	timeless protein, expressed	The expression level of H21 statistically close to T44
MSTRG.14668	LOC_Os03g25660	expressed protein	The expression level of H21 statistically close to T44
MSTRG.20657	LOC_Os05g31110	hexokinase, putative, expressed	The expression level of H21 statistically close to T44
MSTRG.5668	LOC_Os11g01010	autophagy-related protein 8D, putative, expressed	The expression level of H21 statistically close to T44
MSTRG.13789	LOC_Os03g11960	copper/zinc superoxide dismutase, putative, expressed	The expression level of H21 statistically close to T44
MSTRG.20727	LOC_Os05g32460		The expression level of H21 statistically close to T44
MSTRG.27605	LOC_Os08g16720	retrotransposon protein, putative, unclassified, expressed	The expression level of H21 statistically close to T44
MSTRG.22747	LOC_Os06g14460	chromosome condensation protein like, putative, expressed	The expression level of H21 statistically close to T44
MSTRG.25606	LOC_Os07g28930	expressed protein	The expression level of H21 statistically close to T44
MSTRG.11273	LOC_Os02g32660	1,4-alpha-glucan-branching enzyme, chloroplast precursor, putative, expressed	The expression level of H21 statistically close to T44
MSTRG.6651	LOC_Os11g25454	cytokinin-N-glucosyltransferase 1, putative, expressed	The expression level of H21 statistically close to T44
MSTRG.23532			The expression level of H21 statistically close to T44
MSTRG.8985	LOC_Os12g34900	expressed protein	The expression level of H21 statistically close to T44
MSTRG.9928	LOC_Os02g04970; LOC_Os02g05020	expressed protein	The expression level of H21 statistically close to T44
MSTRG.15274	LOC_Os03g43590	LSTK-1-like kinase, putative, expressed	The expression level of H21 statistically close to T44
MSTRG.7305	LOC_Os11g41540	disease resistance RPP8-like protein 3, putative, expressed	The expression level of H21 statistically close to T44
MSTRG.23280			The expression level of H21 statistically close to T44
MSTRG.5589	LOC_Os10g41970	methyltransferase, putative, expressed	The expression level of H21 statistically close to T44
MSTRG.15278	LOC_Os03g43720	transporter family protein, putative, expressed	The expression level of H21 statistically close to T44
MSTRG.2653	LOC_Os01g53460	anthocyanidin 5,3-O-glucosyltransferase, putative, expressed	The expression level of H21 statistically close to T44

MSTRG.29439	LOC_Os09g15850	Leucine Rich Repeat family protein, expressed	The expression level of H21 statistically close to T44
MSTRG.27447	LOC_Os08g12800	glucan endo-1,3-beta-glucosidase precursor, putative, expressed	The expression level of H21 statistically close to T44
MSTRG.1104	LOC_Os01g18280		The expression level of H21 statistically close to T44
MSTRG.20194			The expression level of H21 statistically close to T44
MSTRG.27222	LOC_Os08g08500	reductase, putative, expressed	The expression level of H21 statistically close to T44
MSTRG.27516	LOC_Os08g14320	zinc finger, C3HC4 type domain containing protein, expressed	The expression level of H21 statistically close to T44
MSTRG.21165			The expression level of H21 statistically close to T44
MSTRG.19311	LOC_Os04g58320	zinc finger, RING-type, putative, expressed	The expression level of H21 statistically close to T44
MSTRG.11382	LOC_Os02g34650	expressed protein	The expression level of H21 statistically close to T44
MSTRG.21144	LOC_Os05g39870	CAMK_KIN1/SNF1/Nim1_like.24 - CAMK includes calcium/calmodulin depedent protein kinases, expressed	The expression level of H21 statistically close to T44
MSTRG.20900	LOC_Os05g35200	glycosyl transferase, putative, expressed	The expression level of H21 statistically close to T44
MSTRG.17546			The expression level of H21 statistically close to T44
MSTRG.27925	LOC_Os08g27580	expressed protein	The expression level of H21 statistically close to T44
MSTRG.11918	LOC_Os02g43620	transporter, major facilitator family, putative, expressed	The expression level of H21 statistically close to T44
MSTRG.19168	LOC_Os04g56470	amino acid transporter, putative, expressed	The expression level of H21 statistically close to T44
MSTRG.11392	LOC_Os02g34810	OsAPx8 - Thylakoid-bound Ascorbate Peroxidase encoding gene 5,8, expressed	The expression level of H21 statistically close to T44
MSTRG.4925	LOC_Os10g30690	MYB family transcription factor, putative, expressed	The expression level of H21 statistically close to T44
MSTRG.24989	LOC_Os07g09690	galactosyltransferase family protein, putative, expressed	The expression level of H21 statistically close to T44
MSTRG.19981			The expression level of H21 statistically close to T44
MSTRG.12002	LOC_Os02g44880	expressed protein	The expression level of H21 statistically close to T44
MSTRG.21529	LOC_Os05g46290	T-complex protein, putative, expressed	The expression level of H21 statistically close to T44
MSTRG.23145	LOC_Os06g27590		The expression level of H21 statistically close to T44
MSTRG.6031			The expression level of H21 statistically close to T44
MSTRG.27531	LOC_Os08g14640	syntaxin 6, N-terminal domain containing protein, expressed	The expression level of H21 statistically close to T44
MSTRG.5796	LOC_Os11g02820	CRP10 - Cysteine-rich family protein precursor, expressed	The expression level of H21 statistically close to T44
MSTRG.22883	LOC_Os06g18000	protein kinase domain containing protein, expressed	The expression level of H21 statistically close to T44
MSTRG.8956	LOC_Os12g34510	Core histone H2A/H2B/H3/H4 domain containing protein, putative, expressed	The expression level of H21 statistically close to T44
MSTRG.14297	LOC_Os03g19190	TOPBP1C - Similar to DNA replication protein TOPBP1 from, expressed	The expression level of H21 statistically close to T44
MSTRG.2874	LOC_Os01g56510	periplasmic beta-glucosidase precursor, putative, expressed	The expression level of H21 statistically close to T44
MSTRG.28112	LOC_Os08g31350		The expression level of H21 statistically close to T44
MSTRG.404	LOC_Os01g06560	transcription factor HBP-1b, putative, expressed	The expression level of H21 statistically close to T44
MSTRG.26092	LOC_Os07g38440	uncharacterized 50.6 kDa protein in the 5region of gyrA and gyrB, putative, expressed	The expression level of H21 statistically close to T44
MSTRG.5804	LOC_Os11g02840	protein kinase, putative, expressed	The expression level of H21 statistically close to T44
MSTRG.3891	LOC_Os01g72190	expressed protein	The expression level of H21 statistically close to T44
MSTRG.16855	LOC_Os04g09550	expressed protein	The expression level of H21 statistically close to T44
MSTRG.10300	LOC_Os02g10630	GRAM and C2 domains containing protein, putative, expressed	The expression level of H21 statistically close to T44
MSTRG.25332	LOC_Os07g17210	FAS1, putative, expressed	The expression level of H21 statistically close to T44
MSTRG.29318	LOC_Os09g12310	ubiquitin-conjugating enzyme domain containing protein, expressed	The expression level of H21 statistically close to T44
MSTRG.429	LOC_Os01g07260	expressed protein	The expression level of H21 statistically close to T44
MSTRG.18070	LOC_Os04g39489	amino acid transporter, putative, expressed	The expression level of H21 statistically close to T44
MSTRG.18312	LOC_Os04g42980	zinc finger family protein, putative, expressed	The expression level of H21 statistically close to T44
MSTRG.16538	LOC_Os03g63330	aspartokinase, chloroplast precursor, putative, expressed	The expression level of H21 statistically close to T44
MSTRG.8231			The expression level of H21 statistically close to T44
MSTRG.29744	LOC_Os09g24924	transporter family protein, putative, expressed	The expression level of H21 statistically close to T44
MSTRG.7102	LOC_Os11g38340; LOC_Os11g38360	expressed protein;retrotransposon protein, putative, unclassified, expressed	The expression level of H21 statistically close to T44
MSTRG.4922	LOC_Os10g30640	translin, putative, expressed	The expression level of H21 statistically close to T44
MSTRG.8384			The expression level of H21 statistically close to T44
MSTRG.3057	LOC_Os01g59020	cytochrome P450, putative, expressed	The expression level of H21 statistically close to T44
MSTRG.27624	LOC_Os08g17080	PPR repeat domain containing protein, putative, expressed	The expression level of H21 statistically close to T44
MSTRG.20710	LOC_Os05g32270	AP2 domain containing protein, expressed	The expression level of H21 statistically close to T44
MSTRG.23579			The expression level of H21 statistically close to T44
MSTRG.25865	LOC_Os07g34640	pyruvate Pi dikinase regulatory protein, putative, expressed	The expression level of H21 statistically close to T44

MSTRG.14019	LOC_Os03g15370	ubiquitin fusion protein, putative, expressed	The expression level of H21 statistically close to T44
MSTRG.12416	LOC_Os02g50910	expressed protein	The expression level of H21 statistically close to T44
MSTRG.20198	LOC_Os05g16824	SHR5-receptor-like kinase, putative, expressed	The expression level of H21 statistically close to T44
MSTRG.19598			The expression level of H21 statistically close to T44
MSTRG.4143	LOC_Os10g03780	OsFBX351 - F-box domain containing protein, expressed	The expression level of H21 statistically close to T44
MSTRG.15434	LOC_Os03g46560		The expression level of H21 statistically close to T44
MSTRG.27650	LOC_Os08g18079	expressed protein	The expression level of H21 statistically close to T44
MSTRG.16021			The expression level of H21 statistically close to T44
MSTRG.21852	LOC_Os05g51220	aspartic proteinase, putative, expressed	The expression level of H21 statistically close to T44
MSTRG.18083	LOC_Os04g39670	expressed protein	The expression level of H21 statistically close to T44
MSTRG.22666	LOC_Os06g12950		The expression level of H21 statistically close to T44
MSTRG.29471	LOC_Os09g17049	expressed protein	The expression level of H21 statistically close to T44
MSTRG.12759	LOC_Os02g56100	ribonucleoside-diphosphate reductase large subunit, putative, expressed	The expression level of H21 statistically close to T44
MSTRG.28403	LOC_Os08g36590	antifreeze glycoprotein, putative, expressed	The expression level of H21 statistically close to T44
MSTRG.7685	LOC_Os12g02440	WRKY95, expressed	The expression level of H21 statistically close to T44
MSTRG.27774	LOC_Os08g22960;<br>LOC_Os08g23090	retrotransposon protein, putative, Ty3-gypsy subclass, expressed;retrotransposon protein, putative, unclassified, expressed	The expression level of H21 statistically close to T44
MSTRG.3772	LOC_Os01g70220	histone-lysine N-methyltransferase, putative, expressed	The expression level of H21 statistically close to T44
MSTRG.14523	LOC_Os03g22560	MYB family transcription factor, putative, expressed	The expression level of H21 statistically close to T44
MSTRG.9935	LOC_Os02g05040	cyclin-related protein, putative, expressed	The expression level of H21 statistically close to T44
MSTRG.20261	LOC_Os05g20550		The expression level of H21 statistically close to T44
MSTRG.3733	LOC_Os01g69830	OsSPL2 - SBP-box gene family member, expressed	The expression level of H21 statistically close to T44
MSTRG.4807	LOC_Os10g27470	KH domain containing protein, putative, expressed	The expression level of H21 statistically close to T44
MSTRG.27329	LOC_Os08g10740	transposon protein, putative, CACTA, En/Spm sub-class, expressed	The expression level of H21 statistically close to T44
MSTRG.12119	LOC_Os02g46680		The expression level of H21 statistically close to T44
MSTRG.2034	LOC_Os01g43010	transposon protein, putative, unclassified, expressed	The expression level of H21 statistically close to T44
MSTRG.18661	LOC_Os04g48850	aminotransferase, classes I and II, domain containing protein, expressed	The expression level of H21 statistically close to T44
MSTRG.551	LOC_Os01g08890	expressed protein	The expression level of H21 statistically close to T44
MSTRG.19221	LOC_Os04g57200	heavy metal transport/detoxification protein, putative, expressed	The expression level of H21 statistically close to T44
MSTRG.28178	LOC_Os08g32540	cyclin, putative, expressed	The expression level of H21 statistically close to T44
MSTRG.26796	LOC_Os07g49470	protein kinase APK1B, chloroplast precursor, putative, expressed	The expression level of H21 statistically close to T44
MSTRG.7113			The expression level of H21 statistically close to T44
MSTRG.17872	LOC_Os04g35880;<br>LOC_Os04g35864	DDT domain-containing protein, putative, expressed;retrotransposon protein, putative, unclassified, expressed	The expression level of H21 statistically close to T44
MSTRG.28373	LOC_Os08g35860		The expression level of H21 statistically close to T44
MSTRG.22019	LOC_Os06g03200	expressed protein	The expression level of H21 statistically close to T44
MSTRG.1389	LOC_Os01g26130	expressed protein	The expression level of H21 statistically close to T44
MSTRG.24022	LOC_Os06g44450	CCT/B-box zinc finger protein, putative, expressed	The expression level of H21 statistically close to T44
MSTRG.1516	LOC_Os01g31980	MATE efflux family protein, putative, expressed	The expression level of H21 statistically close to T44
MSTRG.30394	LOC_Os09g36800	3-dehydroquinate synthase, putative, expressed	The expression level of H21 statistically close to T44
MSTRG.29433	LOC_Os09g15780	expressed protein	The expression level of H21 statistically close to T44
MSTRG.3707	LOC_Os01g68930	expressed protein	The expression level of H21 statistically close to T44
MSTRG.8566	LOC_Os12g23450	expressed protein	The expression level of H21 statistically close to T44
MSTRG.3552	LOC_Os01g66600	rhodanese-like, putative, expressed	The expression level of H21 statistically close to T44
MSTRG.16309	LOC_Os03g60030	transposon protein, putative, CACTA, En/Spm sub-class, expressed	The expression level of H21 statistically close to T44
MSTRG.10486	LOC_Os02g13600	expressed protein	The expression level of H21 statistically close to T44
MSTRG.2760	LOC_Os01g54980	expressed protein	The expression level of H21 statistically close to T44
MSTRG.2302	LOC_Os01g48110	hypothetical protein	The expression level of H21 statistically close to T44
MSTRG.16831	LOC_Os04g08350	cysteine synthase, chloroplast/chromoplast precursor, putative, expressed	The expression level of H21 statistically close to T44
MSTRG.27728	LOC_Os08g20680	expressed protein	The expression level of H21 statistically close to T44
MSTRG.19205	LOC_Os04g56850	auxin response factor, putative, expressed	The expression level of H21 statistically close to T44
MSTRG.2729	LOC_Os01g54600	WRKY13, expressed	The expression level of H21 statistically close to T44
MSTRG.13224	LOC_Os03g04140	serine acetyltransferase protein, putative, expressed	The expression level of H21 statistically close to T44
MSTRG.27079	LOC_Os08g05910	peptide transporter PTR2, putative, expressed	The expression level of H21 statistically close to T44
MSTRG.3596	LOC_Os01g67280	expressed protein	The expression level of H21 statistically close to T44
MSTRG.27000	LOC_Os08g04340	plastocyanin-like domain containing protein, putative, expressed	The expression level of H21 statistically close to T44

MSTRG.15355	LOC_Os03g45170	amino acid permease, putative, expressed	The expression level of H21 statistically close to T44
MSTRG.29275	LOC_Os09g10750	rhodanese-like, putative, expressed	The expression level of H21 statistically close to T44
MSTRG.23491	LOC_Os06g36450	ferroportin1 domain containing protein, expressed	The expression level of H21 statistically close to T44
MSTRG.7530	LOC_Os11g47320	protein transporter, putative, expressed	The expression level of H21 statistically close to T44
MSTRG.22266	LOC_Os06g06580	expressed protein	The expression level of H21 statistically close to T44
MSTRG.20931	LOC_Os05g35650	peptide transporter PTR2, putative, expressed	The expression level of H21 statistically close to T44
MSTRG.1580	LOC_Os01g33450	expressed protein	The expression level of H21 statistically close to T44
MSTRG.6989			The expression level of H21 statistically close to T44
MSTRG.29013	LOC_Os09g01960	MYB family transcription factor, putative, expressed	The expression level of H21 statistically close to T44
MSTRG.22925			The expression level of H21 statistically close to T44
MSTRG.7003	LOC_Os11g36670	expressed protein	The expression level of H21 statistically close to T44
MSTRG.12708	LOC_Os02g55330	OsPOP6 - Putative Prolyl Oligopeptidase homologue, expressed	The expression level of H21 statistically close to T44
MSTRG.5132	LOC_Os10g34820	CDT1B - Putative DNA replication initiation protein, expressed	The expression level of H21 statistically close to T44
MSTRG.17261			The expression level of H21 statistically close to T44
MSTRG.16907	LOC_Os04g11820	white-brown complex homolog protein, putative, expressed	The expression level of H21 statistically close to T44
MSTRG.17660	LOC_Os04g32960	TUDOR protein with multiple SNc domains, putative, expressed	The expression level of H21 statistically close to T44
MSTRG.21432	LOC_Os05g44922	6-phosphofructokinase, putative, expressed	The expression level of H21 statistically close to T44
MSTRG.3427	LOC_Os01g64870	expressed protein	The expression level of H21 statistically close to T44
MSTRG.26918	LOC_Os08g02850	zinc finger family protein, putative, expressed	The expression level of H21 statistically close to T44
MSTRG.2477			The expression level of H21 statistically close to T44
MSTRG.21088	LOC_Os05g38984	expressed protein	The expression level of H21 statistically close to T44
MSTRG.5023	LOC_Os10g33170	POT domain containing peptide transporter, putative, expressed	The expression level of H21 statistically close to T44
MSTRG.13468	LOC_Os03g07820	exostosin family protein, putative, expressed	The expression level of H21 statistically close to T44
MSTRG.5024	LOC_Os10g33204		The expression level of H21 statistically close to T44
MSTRG.19073	LOC_Os04g54940	ATEB1A-like microtubule associated protein, putative, expressed	The expression level of H21 statistically close to T44
MSTRG.953	LOC_Os01g15480	EMB3013, putative, expressed	The expression level of H21 statistically close to T44
MSTRG.884	LOC_Os01g14170		The expression level of H21 statistically close to T44
MSTRG.21718	LOC_Os05g49260	transporter family protein, putative, expressed	The expression level of H21 statistically close to T44
MSTRG.8605	LOC_Os12g24500	expressed protein	The expression level of H21 statistically close to T44
MSTRG.6947	LOC_Os11g35090	kinesin motor domain containing protein, putative, expressed	The expression level of H21 statistically close to T44
MSTRG.12842	LOC_Os02g57060	OsCttP2 - Putative C-terminal processing peptidase homologue, expressed	The expression level of H21 statistically close to T44
MSTRG.1169	LOC_Os01g19940	expressed protein	The expression level of H21 statistically close to T44
MSTRG.7918	LOC_Os12g06180	HVA22, putative, expressed	The expression level of H21 statistically close to T44
MSTRG.13436	LOC_Os03g07300	ribulose-phosphate 3-epimerase, chloroplast precursor, putative, expressed	The expression level of H21 statistically close to T44
MSTRG.4383	LOC_Os10g11140	phosphoglucomutase, putative, expressed	The expression level of H21 statistically close to T44
MSTRG.530	LOC_Os01g08550	aminoacyl-tRNA synthetase, putative, expressed	The expression level of H21 statistically close to T44
MSTRG.13458	LOC_Os03g07720	expressed protein	The expression level of H21 statistically close to T44
MSTRG.22665	LOC_Os06g12950	expressed protein	The expression level of H21 statistically close to T44
MSTRG.21019	LOC_Os05g37884	50S ribosomal protein L33, putative, expressed	The expression level of H21 statistically close to T44
MSTRG.25360	LOC_Os07g19060	transport protein particle component, Bet3, domain containing protein, expressed	The expression level of H21 statistically close to T44
MSTRG.25854	LOC_Os07g33954	transporter family protein, putative, expressed	The expression level of H21 statistically close to T44
MSTRG.4822	LOC_Os10g28200	NAD dependent epimerase/dehydratase family protein, putative, expressed	The expression level of H21 statistically close to T44
MSTRG.19940	LOC_Os05g09520	IQ calmodulin-binding motif family protein, expressed	The expression level of H21 statistically close to T44
MSTRG.13570	LOC_Os03g09070		The expression level of H21 statistically close to T44
MSTRG.10782	LOC_Os02g20330	expressed protein	The expression level of H21 statistically close to T44
MSTRG.12723	LOC_Os02g55530	neurochondrin family protein, putative, expressed	The expression level of H21 statistically close to T44
MSTRG.21281	LOC_Os05g42210	serine/threonine-protein kinase receptor precursor, putative, expressed	The expression level of H21 statistically close to T44
MSTRG.30343	LOC_Os09g36130	expressed protein	The expression level of H21 statistically close to T44
MSTRG.1471	LOC_Os01g29507	retrotransposon, putative, centromere-specific, expressed	The expression level of H21 statistically close to T44
MSTRG.13149	LOC_Os03g03070; LOC_Os03g03100	transcription factor, putative, expressed;OsMADS50 - MADS-box family gene with MIKCc type-box, expressed	The expression level of H21 statistically close to T44
MSTRG.14792			The expression level of H21 statistically close to T44
MSTRG.20385	LOC_Os05g25210	expressed protein	The expression level of H21 statistically close to T44

MSTRG.14286	LOC_Os03g18980	kinesin motor domain containing protein, expressed	The expression level of H21 statistically close to T44
MSTRG.19943	LOC_Os05g09530	expressed protein	The expression level of H21 statistically close to T44
MSTRG.28254	LOC_Os08g33710	ribonuclease T2 family domain containing protein, expressed	The expression level of H21 statistically close to T44
MSTRG.5639	LOC_Os10g42730	expressed protein	The expression level of H21 statistically close to T44
MSTRG.3844	LOC_Os01g71256	expressed protein	The expression level of H21 statistically close to T44
MSTRG.1137	LOC_Os01g19370	retrotransposon protein, putative, unclassified, expressed	The expression level of H21 statistically close to T44
MSTRG.26801	LOC_Os07g49540	expressed protein	The expression level of H21 statistically close to T44
MSTRG.23516	LOC_Os06g36890	glycosyl transferase, group 1 domain containing protein, expressed	The expression level of H21 statistically close to T44
MSTRG.18273	LOC_Os04g42360	expressed protein	The expression level of H21 statistically close to T44
MSTRG.3626	LOC_Os01g67590	oligopeptidase, putative, expressed	The expression level of H21 statistically close to T44
MSTRG.8108	LOC_Os12g09660	expressed protein	The expression level of H21 statistically close to T44
MSTRG.14206	LOC_Os03g17960	expressed protein	The expression level of H21 statistically close to T44
MSTRG.23710	LOC_Os06g40570	GRAM and C2 domains containing protein, putative, expressed	The expression level of H21 statistically close to T44
MSTRG.6448	LOC_Os11g14900	thiol protease SEN102 precursor, putative, expressed	The expression level of H21 statistically close to T44
MSTRG.30308	LOC_Os09g35680	OsFBX339 - F-box domain containing protein, expressed	The expression level of H21 statistically close to T44
MSTRG.6278	LOC_Os11g11240	expressed protein	The expression level of H21 statistically close to T44
MSTRG.10440	LOC_Os02g12870	expressed protein	The expression level of H21 statistically close to T44
MSTRG.25395	LOC_Os07g22400	POLA3 - Putative DNA polymerase alpha complex subunit, expressed	The expression level of H21 statistically close to T44
MSTRG.7869	LOC_Os12g05310	retrotransposon protein, putative, unclassified, expressed	The expression level of H21 statistically close to T44
MSTRG.20630	LOC_Os05g30720		The expression level of H21 statistically close to T44
MSTRG.22645	LOC_Os06g12630	glutathione S-transferase, N-terminal domain containing protein, expressed	The expression level of H21 statistically close to T44
MSTRG.9201	LOC_Os12g38790	targeting protein for Xklp2 containing protein, expressed	The expression level of H21 statistically close to T44
MSTRG.28180	LOC_Os08g32570	expressed protein	The expression level of H21 statistically close to T44
MSTRG.19991	LOC_Os05g10930		The expression level of H21 statistically close to T44
MSTRG.20097			The expression level of H21 statistically close to T44
MSTRG.1382	LOC_Os01g26070		The expression level of H21 statistically close to T44
MSTRG.6678	LOC_Os11g26780	dehydrin, putative, expressed	The expression level of H21 statistically close to T44
MSTRG.4740	LOC_Os10g25990	retrotransposon protein, putative, unclassified, expressed	The expression level of H21 statistically close to T45
MSTRG.4629	LOC_Os10g22630	expressed protein	The expression level of H21 statistically close to T45
MSTRG.26552	LOC_Os07g46210	LTPL2 - Protease inhibitor/seed storage/LTP family protein precursor, expressed	The expression level of H21 statistically close to T45
MSTRG.5748	LOC_Os11g02165	LTPL57 - Protease inhibitor/seed storage/LTP family protein precursor, expressed	The expression level of H21 statistically close to T45
MSTRG.13422	LOC_Os03g07140	male sterility protein, putative, expressed	The expression level of H21 statistically close to T45
MSTRG.10793			The expression level of H21 statistically close to T45
MSTRG.2389	LOC_Os01g49370	expressed protein	The expression level of H21 statistically close to T45
MSTRG.5332	LOC_Os10g38050	HOTHEAD precursor, putative, expressed	The expression level of H21 statistically close to T45
MSTRG.7664	LOC_Os12g02105	LTPL58 - Protease inhibitor/seed storage/LTP family protein precursor, expressed	The expression level of H21 statistically close to T45
MSTRG.9656	LOC_Os02g01980	GDSL-like lipase/acylhydrolase, putative, expressed	The expression level of H21 statistically close to T45
MSTRG.18489	LOC_Os04g45960	OsSub42 - Putative Subtilisin homologue, expressed	The expression level of H21 statistically close to T45
MSTRG.18873	LOC_Os04g52320	QRT3, putative, expressed	The expression level of H21 statistically close to T45
MSTRG.15504	LOC_Os03g47890	transposon protein, putative, unclassified, expressed	The expression level of H21 statistically close to T45
MSTRG.22623	LOC_Os06g12330	amino acid transporter, putative, expressed	The expression level of H21 statistically close to T45
MSTRG.8563	LOC_Os12g23780	expressed protein	The expression level of H21 statistically close to T45
MSTRG.24559	LOC_Os07g02110	expressed protein	The expression level of H21 statistically close to T45
MSTRG.24655	LOC_Os07g04270		The expression level of H21 statistically close to T45
MSTRG.17386			The expression level of H21 statistically close to T45
MSTRG.16729	LOC_Os04g06734		The expression level of H21 statistically close to T45
MSTRG.13015	LOC_Os03g01360; LOC_Os03g01350	expressed protein	The expression level of H21 statistically close to T45
MSTRG.9927	LOC_Os02g04960	expressed protein	The expression level of H21 statistically close to T45
MSTRG.3346	LOC_Os01g63540	cytochrome P450, putative, expressed	The expression level of H21 statistically close to T45
MSTRG.30129	LOC_Os09g32020; LOC_Os09g32050; LOC_Os09g32030; LOC_Os09g32040	expressed protein;no apical meristem protein, putative, expressed;chaperone protein dnaJ 10, putative, expressed	The expression level of H21 statistically close to T45
MSTRG.6484	LOC_Os11g16550	uncharacterized protein ycf53, putative, expressed	The expression level of H21 statistically close to T45
MSTRG.8558	LOC_Os12g23754	expressed protein	The expression level of H21 statistically close to T45
MSTRG.15174	LOC_Os03g40830	OsSub30 - Putative Subtilisin homologue, expressed	The expression level of H21 statistically close to T45
MSTRG.13116	LOC_Os03g02700		The expression level of H21 statistically close to T45
MSTRG.24380	LOC_Os06g49930	OsFBX207 - F-box domain containing protein, expressed	The expression level of H21 statistically close to T45

MSTRG.26518	LOC_Os07g45530	retrotransposon protein, putative, unclassified, expressed	The expression level of H21 statistically close to T45
MSTRG.25954	LOC_Os07g36430		The expression level of H21 statistically close to T45
MSTRG.30133	LOC_Os09g32140	expressed protein	The expression level of H21 statistically close to T45
MSTRG.9064	LOC_Os12g36060	expressed protein	The expression level of H21 statistically close to T45
MSTRG.14200	LOC_Os03g17870		The expression level of H21 statistically close to T45
MSTRG.27917	LOC_Os08g27220		The expression level of H21 statistically close to T45
MSTRG.27117	LOC_Os08g06415	expressed protein	The expression level of H21 statistically close to T45
MSTRG.5105	LOC_Os10g34480	cytochrome P450, putative, expressed	The expression level of H21 statistically close to T45
MSTRG.5766	LOC_Os11g02440	chalcone--flavonone isomerase, putative, expressed	The expression level of H21 statistically close to T45
MSTRG.1260	LOC_Os01g22352	peroxidase precursor, putative, expressed	The expression level of H21 statistically close to T45
MSTRG.8700			The expression level of H21 statistically close to T45
MSTRG.26919	LOC_Os08g02860		The expression level of H21 statistically close to T45
MSTRG.14725	LOC_Os03g26930; LOC_Os03g26920	OsSCP12 - Putative Serine Carboxypeptidase homologue, expressed;OsSCP13 - Putative Serine Carboxypeptidase homologue, expressed	The expression level of H21 statistically close to T45
MSTRG.23706	LOC_Os06g40550	ABC-2 type transporter domain containing protein, expressed	The expression level of H21 statistically close to T45
MSTRG.16848	LOC_Os04g09350	expressed protein	The expression level of H21 statistically close to T45
MSTRG.26287	LOC_Os07g41650	pectinesterase, putative, expressed	The expression level of H21 statistically close to T45
MSTRG.11368	LOC_Os02g34470		The expression level of H21 statistically close to T45
MSTRG.6534			The expression level of H21 statistically close to T45
MSTRG.20105	LOC_Os05g14260	peroxidase precursor, putative, expressed	The expression level of H21 statistically close to T45
MSTRG.18629	LOC_Os04g48210	cytochrome P450, putative, expressed	The expression level of H21 statistically close to T45
MSTRG.17650	LOC_Os04g32770; LOC_Os04g32760	retrotransposon protein, putative, unclassified, expressed	The expression level of H21 statistically close to T45
MSTRG.6931	LOC_Os11g34824	expressed protein	The expression level of H21 statistically close to T45
MSTRG.26405			The expression level of H21 statistically close to T45
MSTRG.14369	LOC_Os03g20140	transposon protein, putative, CACTA, En/Spm sub-class, expressed	The expression level of H21 statistically close to T45
MSTRG.7238	LOC_Os11g40249	expressed protein	The expression level of H21 statistically close to T45
MSTRG.10294	LOC_Os02g10520	OsSub12 - Putative Subtilisin homologue, expressed	The expression level of H21 statistically close to T45
MSTRG.7680	LOC_Os12g02370	chalcone--flavonone isomerase, putative, expressed	The expression level of H21 statistically close to T45
MSTRG.7709	LOC_Os12g02790		The expression level of H21 statistically close to T45
MSTRG.24776	LOC_Os07g06530		The expression level of H21 statistically close to T45
MSTRG.8003	LOC_Os12g07500	csAtPR5, putative, expressed	The expression level of H21 statistically close to T45
MSTRG.14747	LOC_Os03g27340	expressed protein	The expression level of H21 statistically close to T45
MSTRG.13584			The expression level of H21 statistically close to T45
MSTRG.5244	LOC_Os10g36260	expressed protein	The expression level of H21 statistically close to T45
MSTRG.13904	LOC_Os03g13690	expressed protein	The expression level of H21 statistically close to T45
MSTRG.16870	LOC_Os04g10214	expressed protein	The expression level of H21 statistically close to T45
MSTRG.14707	LOC_Os03g26370	zinc finger, C3HC4 type domain containing protein, expressed	The expression level of H21 statistically close to T45
MSTRG.26507	LOC_Os07g45439	expressed protein	The expression level of H21 statistically close to T45
MSTRG.8428			The expression level of H21 statistically close to T45
MSTRG.15077	LOC_Os03g39100	no apical meristem protein, expressed	The expression level of H21 statistically close to T45
MSTRG.16845			The expression level of H21 statistically close to T45
MSTRG.14884			The expression level of H21 statistically close to T45
MSTRG.5241	LOC_Os10g36270	disease resistance RPP13-like protein 1, putative, expressed	The expression level of H21 statistically close to T45
MSTRG.8265			The expression level of H21 statistically close to T45
MSTRG.22830			The expression level of H21 statistically close to T45
MSTRG.30564	LOC_Os09g39290	retrotransposon protein, putative, unclassified, expressed	The expression level of H21 statistically close to T45
MSTRG.14299	LOC_Os03g19220	expressed protein	The expression level of H21 statistically close to T45
MSTRG.24375	LOC_Os06g49920	expressed protein	The expression level of H21 statistically close to T45
MSTRG.1595	LOC_Os01g33762	expressed protein	The expression level of H21 statistically close to T45
MSTRG.23041			The expression level of H21 statistically close to T45
MSTRG.29639	LOC_Os09g22000	hydrolase, HAD superfamily, Cof family, putative, expressed	The expression level of H21 statistically close to T45
MSTRG.7070	LOC_Os11g37759	stripe rust resistance protein Yr10, putative, expressed	The expression level of H21 statistically close to T45
MSTRG.6263	LOC_Os11g10920	carboxyl-terminal proteinase, putative, expressed	The expression level of H21 statistically close to T45
MSTRG.8634	LOC_Os12g25494	transposon protein, putative, unclassified, expressed	The expression level of H21 statistically close to T45
MSTRG.23016	LOC_Os06g21750	expressed protein	The expression level of H21 statistically close to T45
MSTRG.8795			The expression level of H21 statistically close to T45
MSTRG.17847	LOC_Os04g35540	amino acid permease family protein, putative, expressed	The expression level of H21 statistically close to T45
MSTRG.24435	LOC_Os06g50950	GDSL-like lipase/acylhydrolase, putative, expressed	The expression level of H21 statistically close to T45
MSTRG.26454	LOC_Os07g44670	retrotransposon protein, putative, unclassified, expressed	The expression level of H21 statistically close to T45
MSTRG.6101	LOC_Os11g07830	dirigent, putative, expressed	The expression level of H21 statistically close to T45
MSTRG.24521	LOC_Os07g01410	peroxidase precursor, putative, expressed	The expression level of H21 statistically close to T45
MSTRG.22774			The expression level of H21 statistically close to T45
MSTRG.20291	LOC_Os05g22930		The expression level of H21 statistically close to T45

MSTRG.8267	LOC_Os12g13930;<br>LOC_Os12g13940	3-oxoacyl-reductase, chloroplast precursor, putative, expressed;DNA-binding storekeeper protein-related, putative, expressed	The expression level of H21 statistically close to T45
MSTRG.1230			The expression level of H21 statistically close to T45
MSTRG.11156	LOC_Os02g30070	expressed protein	The expression level of H21 statistically close to T45
MSTRG.28707	LOC_Os08g41690	expressed protein	The expression level of H21 statistically close to T45
MSTRG.16904	LOC_Os04g11570		The expression level of H21 statistically close to T45
MSTRG.4626			The expression level of H21 statistically close to T45
MSTRG.15468	LOC_Os03g47470	STE_PAK_Ste20_STLK.4 - STE kinases include homologs to sterile 7, sterile 11 and sterile 20 from yeast, expressed	The expression level of H21 statistically close to T45
MSTRG.6848	LOC_Os11g32650	chalcone synthase, putative, expressed	The expression level of H21 statistically close to T45
MSTRG.26952	LOC_Os08g03676;<br>LOC_Os08g03682	pentatricopeptide, putative, expressed;cytochrome P450, putative, expressed	The expression level of H21 statistically close to T45
MSTRG.29628	LOC_Os09g21689		The expression level of H21 statistically close to T45
MSTRG.707			The expression level of H21 statistically close to T45
MSTRG.21757	LOC_Os05g49900	3-ketoacyl-CoA synthase, putative, expressed	The expression level of H21 statistically close to T45
MSTRG.26245	LOC_Os07g40850	retrotransposon protein, putative, unclassified, expressed	The expression level of H21 statistically close to T45
MSTRG.29634	LOC_Os09g21880;<br>LOC_Os09g21900	hypothetical protein;expressed protein	The expression level of H21 statistically close to T45
MSTRG.5630	LOC_Os10g42620	dihydroflavonol-4-reductase, putative, expressed	The expression level of H21 statistically close to T45
MSTRG.23502	LOC_Os06g36650		The expression level of H21 statistically close to T45
MSTRG.24400	LOC_Os06g50230	expressed protein	The expression level of H21 statistically close to T45
MSTRG.14363			The expression level of H21 statistically close to T45
MSTRG.10721	LOC_Os02g19200		The expression level of H21 statistically close to T45
MSTRG.1325	LOC_Os01g24210	retrotransposon protein, putative, Ty1-copia subclass, expressed	The expression level of H21 statistically close to T45
MSTRG.24601	LOC_Os07g03030		The expression level of H21 statistically close to T45
MSTRG.1855	LOC_Os01g40170	translation initiation factor, putative, expressed	The expression level of H21 statistically close to T45
MSTRG.22351	LOC_Os06g07887	exostosin family protein, putative, expressed	The expression level of H21 statistically close to T45
MSTRG.25719	LOC_Os07g31690		The expression level of H21 statistically close to T45
MSTRG.8702	LOC_Os12g27370	expressed protein	The expression level of H21 statistically close to T45
MSTRG.22947	LOC_Os06g20200	gibberellin receptor GID1L2, putative, expressed	The expression level of H21 statistically close to T45
MSTRG.15400	LOC_Os03g46140		The expression level of H21 statistically close to T45
MSTRG.8002			The expression level of H21 statistically close to T45
MSTRG.1005	LOC_Os01g16240	OsCam1-3 - Calmodulin, expressed	The expression level of H21 statistically close to T45
MSTRG.12752	LOC_Os02g56014	40S ribosomal protein S30, putative, expressed	The expression level of H21 statistically close to T45
MSTRG.14043	LOC_Os03g15710	strictosidine synthase, putative, expressed	The expression level of H21 statistically close to T45
MSTRG.363	LOC_Os01g05730		The expression level of H21 statistically close to T45
MSTRG.22831	LOC_Os06g16630		The expression level of H21 statistically close to T45
MSTRG.233	LOC_Os01g03340;<br>LOC_Os01g03360	BBTI4 - Bowman-Birk type bran trypsin inhibitor precursor, expressed;BBTI5 - Bowman-Birk type bran trypsin inhibitor precursor, expressed	The expression level of H21 statistically close to T45
MSTRG.22755	LOC_Os06g14620	ribonucleoside-diphosphate reductase small chain, putative, expressed	The expression level of H21 statistically close to T45
MSTRG.11188	LOC_Os02g30530	transposon protein, putative, unclassified, expressed	The expression level of H21 statistically close to T45
MSTRG.27706	LOC_Os08g20200	male sterility protein, putative, expressed	The expression level of H21 statistically close to T45
MSTRG.8320	LOC_Os12g15505	expressed protein	The expression level of H21 statistically close to T45
MSTRG.28325	LOC_Os08g34860	OsFBX293 - F-box domain containing protein, expressed	The expression level of H21 statistically close to T45
MSTRG.18963	LOC_Os04g53350	expressed protein	The expression level of H21 statistically close to T45
LOC_Os08g20290	LOC_Os08g20290	transposon protein, putative, unclassified, expressed	The expression level of H21 statistically close to T45
MSTRG.28958			The expression level of H21 statistically close to T45
MSTRG.16704	LOC_Os04g04070	expressed protein	The expression level of H21 statistically close to T45
MSTRG.2380	LOC_Os01g49270	XPA-binding protein 2, putative, expressed	The expression level of H21 statistically close to T45
MSTRG.27223	LOC_Os08g08570	expressed protein	The expression level of H21 statistically close to T45
MSTRG.17306	LOC_Os04g24200		The expression level of H21 statistically close to T45
MSTRG.24049	LOC_Os06g45020		The expression level of H21 statistically close to T45
MSTRG.6929	LOC_Os11g34810	expressed protein	The expression level of H21 statistically close to T45
MSTRG.25418	LOC_Os07g23190		The expression level of H21 statistically close to T45
MSTRG.22512	LOC_Os06g10355	expressed protein	The expression level of H21 statistically close to T45
MSTRG.7245	LOC_Os11g40410		The expression level of H21 statistically close to T45
MSTRG.26576	LOC_Os07g46470		The expression level of H21 statistically close to T45
MSTRG.5246	LOC_Os10g36340	expressed protein	The expression level of H21 statistically close to T45
MSTRG.20095	LOC_Os05g14070	expressed protein	The expression level of H21 statistically close to T45
MSTRG.11079	LOC_Os02g28220	transferase family protein, putative, expressed	The expression level of H21 statistically close to T45
MSTRG.26330	LOC_Os07g42490	sucrose synthase, putative, expressed	The expression level of H21 statistically close to T45
MSTRG.27476			The expression level of H21 statistically close to T45

MSTRG.16076	LOC_Os03g56890		The expression level of H21 statistically close to T45
MSTRG.1146	LOC_Os01g19460	retrotransposon protein, putative, unclassified, expressed	The expression level of H21 statistically close to T45
MSTRG.8788			The expression level of H21 statistically close to T45
MSTRG.22496	LOC_Os06g10100		The expression level of H21 statistically close to T45
MSTRG.25996	LOC_Os07g37080		The expression level of H21 statistically close to T45
MSTRG.7987	LOC_Os12g07410		The expression level of H21 statistically close to T45
MSTRG.24857	LOC_Os07g07790	LTPL75 - Protease inhibitor/seed storage/LTP family protein precursor, expressed	The expression level of H21 statistically close to T45
MSTRG.17811	LOC_Os04g35010	helix-loop-helix DNA-binding domain containing protein, expressed	The expression level of H21 statistically close to T45
MSTRG.2814	LOC_Os01g55600	peptide transporter PTR2, putative, expressed	The expression level of H21 statistically close to T45
MSTRG.13991	LOC_Os03g14920		The expression level of H21 statistically close to T45
MSTRG.15173	LOC_Os03g40800		The expression level of H21 statistically close to T45
MSTRG.10724	LOC_Os02g19380	microfibrillar-associated protein 1, putative, expressed	The expression level of H21 statistically close to T45
MSTRG.13295	LOC_Os03g05270	RING finger and CHY zinc finger domain-containing protein 1, putative, expressed	The expression level of H21 statistically close to T45
MSTRG.2838	LOC_Os01g56040	zinc finger A20 and AN1 domain-containing stress-associated protein, putative, expressed	The expression level of H21 statistically close to T45
MSTRG.2745	LOC_Os01g54770		The expression level of H21 statistically close to T45
MSTRG.5983	LOC_Os11g05614	no apical meristem protein, putative, expressed	The expression level of H21 statistically close to T45
MSTRG.7063	LOC_Os11g37660		The expression level of H21 statistically close to T45
MSTRG.8251			The expression level of H21 statistically close to T45
MSTRG.25819	LOC_Os07g33640	expressed protein	The expression level of H21 statistically close to T45
MSTRG.24924	LOC_Os07g08540		The expression level of H21 statistically close to T45
MSTRG.30508	LOC_Os09g38470	expressed protein	The expression level of H21 statistically close to T45
MSTRG.21210	LOC_Os05g41090; LOC_Os05g41100	CAMK_CAMK_like_CG18020d.1 - CAMK includes calcium/calmodulin depedent protein kinases, expressed;protein kri1, putative, expressed	The expression level of H21 statistically close to T45
MSTRG.452	LOC_Os01g07560	receptor-like protein kinase 2 precursor, putative, expressed	The expression level of H21 statistically close to T45
MSTRG.8676			The expression level of H21 statistically close to T45
MSTRG.28502	LOC_Os08g38470		The expression level of H21 statistically close to T45
MSTRG.12606	LOC_Os02g53835	expressed protein	The expression level of H21 statistically close to T45
MSTRG.6260	LOC_Os11g10910	chloroplast nucleoid DNA-binding protein, putative, expressed	The expression level of H21 statistically close to T45
MSTRG.3949			The expression level of H21 statistically close to T45
MSTRG.26645	LOC_Os07g47310		The expression level of H21 statistically close to T45
MSTRG.6930	LOC_Os11g34820	THUMP domain-containing protein, putative, expressed	The expression level of H21 statistically close to T45
MSTRG.14012	LOC_Os03g15260	expressed protein	The expression level of H21 statistically close to T45
MSTRG.17834			The expression level of H21 statistically close to T45
MSTRG.28722	LOC_Os08g41860		The expression level of H21 statistically close to T45
MSTRG.16978	LOC_Os04g14150	dehydration response related protein, putative, expressed	The expression level of H21 statistically close to T45
MSTRG.21833	LOC_Os05g50920	transmembrane amino acid transporter protein, putative, expressed	The expression level of H21 statistically close to T45
MSTRG.15682	LOC_Os03g50760	EF hand family protein, putative, expressed	The expression level of H21 statistically close to T45
MSTRG.23726	LOC_Os06g40770		The expression level of H21 statistically close to T45
MSTRG.7046	LOC_Os11g37390	OsFBDUF54 - F-box and DUF domain containing protein, expressed	The expression level of H21 statistically close to T45
MSTRG.13114	LOC_Os03g02670	transporter family protein, putative, expressed	The expression level of H21 statistically close to T45
MSTRG.18154	LOC_Os04g40800	OsFBL18 - F-box domain and LRR containing protein, expressed	The expression level of H21 statistically close to T45
MSTRG.14461	LOC_Os03g21700	DNA-binding protein, putative, expressed	The expression level of H21 statistically close to T45
MSTRG.27224	LOC_Os08g08586	expressed protein	The expression level of H21 statistically close to T45
MSTRG.1594	LOC_Os01g33760	expressed protein	The expression level of H21 statistically close to T45
MSTRG.14145	LOC_Os03g17110	transposon protein, putative, CACTA, En/Spm sub-class, expressed	The expression level of H21 statistically close to T45
MSTRG.27489	LOC_Os08g13699	expressed protein	The expression level of H21 statistically close to T45
MSTRG.26463	LOC_Os07g44760		The expression level of H21 statistically close to T45
MSTRG.25004			The expression level of H21 statistically close to T45
MSTRG.5134	LOC_Os10g34970	expressed protein	The expression level of H21 statistically close to T45
MSTRG.4559	LOC_Os10g20990		The expression level of H21 statistically close to T45
MSTRG.25832	LOC_Os07g33730	NB-ARC domain containing protein, expressed	The expression level of H21 statistically close to T45
MSTRG.2211			The expression level of H21 statistically close to T45
MSTRG.25826	LOC_Os07g33690	NBS-LRR type disease resistance protein Hom-F, putative, expressed	The expression level of H21 statistically close to T45
MSTRG.27627	LOC_Os08g17130		The expression level of H21 statistically close to T45
MSTRG.18738	LOC_Os04g49930	sensitivity to red light reduced protein 1, putative, expressed	The expression level of H21 statistically close to T45
MSTRG.7053			The expression level of H21 statistically close to T45
MSTRG.10438	LOC_Os02g12840		The expression level of H21 statistically close to T45
MSTRG.22889	LOC_Os06g17980		The expression level of H21 statistically close to T45

MSTRG.10620	LOC_Os02g16770		The expression level of H21 statistically close to T45
MSTRG.13781			The expression level of H21 statistically close to T45
MSTRG.27350			The expression level of H21 statistically close to T45
MSTRG.364	LOC_Os01g05730	retrotransposon protein, putative, unclassified, expressed	The expression level of H21 statistically close to T45
MSTRG.13508	LOC_Os03g08350	ubiquitin carboxyl-terminal hydrolase, family 1, putative, expressed	The expression level of H21 statistically close to T45
MSTRG.18103	LOC_Os04g40040	copper methylamine oxidase precursor, putative, expressed	The expression level of H21 statistically close to T45
MSTRG.16109	LOC_Os03g57200	glutathione S-transferase, putative, expressed	The expression level of H21 statistically close to T45
MSTRG.4238	LOC_Os10g06000	POEI15 - Pollen Ole e I allergen and extensin family protein precursor, expressed	The expression level of H21 statistically close to T45
MSTRG.2447	LOC_Os01g50290		The expression level of H21 statistically close to T45
MSTRG.1075			The expression level of H21 statistically close to T45
MSTRG.22592	LOC_Os06g11812	wound/stress protein, putative, expressed	The expression level of H21 statistically close to T45
MSTRG.10774	LOC_Os02g20250		The expression level of H21 statistically close to T45
MSTRG.23019	LOC_Os06g21730	retrotransposon protein, putative, Ty3-gypsy subclass, expressed	The expression level of H21 statistically close to T45
MSTRG.12855	LOC_Os02g57250	OsIAA10 - Auxin-responsive Aux/IAA gene family member, expressed	The expression level of H21 statistically close to T45
MSTRG.23566	LOC_Os06g37700; LOC_Os06g37720; LOC_Os06g37710	expressed protein;hypothetical protein	The expression level of H21 statistically close to T45
MSTRG.2917	LOC_Os01g57050	expressed protein	The expression level of H21 statistically close to T45
MSTRG.3803	LOC_Os01g70600	DUF567 domain containing protein, putative, expressed	The expression level of H21 statistically close to T45
MSTRG.4046	LOC_Os01g74500; LOC_Os01g74510	expressed protein;KIP1, putative, expressed	The expression level of H21 statistically close to T45
MSTRG.4819	LOC_Os10g28130		The expression level of H21 statistically close to T45
MSTRG.11691	LOC_Os02g39450		The expression level of H21 statistically close to T45
MSTRG.25781	LOC_Os07g33010		The expression level of H21 statistically close to T45
MSTRG.653	LOC_Os01g10700		The expression level of H21 statistically close to T45
MSTRG.21720	LOC_Os05g49290	3-ketoacyl-CoA synthase, putative, expressed	The expression level of H21 statistically close to T45
MSTRG.9559	LOC_Os12g44010	purple acid phosphatase precursor, putative, expressed	The expression level of H21 statistically close to T45
MSTRG.28043			The expression level of H21 statistically close to T45
MSTRG.8242			The expression level of H21 statistically close to T45
MSTRG.2056	LOC_Os01g43320	transmembrane amino acid transporter protein, putative, expressed	The expression level of H21 statistically close to T45
MSTRG.21184	LOC_Os05g40540; LOC_Os05g40550	expressed protein	The expression level of H21 statistically close to T45
MSTRG.26631	LOC_Os07g47120	beta-amylase, putative, expressed	The expression level of H21 statistically close to T45
MSTRG.27482			The expression level of H21 statistically close to T45
MSTRG.11009	LOC_Os02g26790	expressed protein	The expression level of H21 statistically close to T45
MSTRG.16996	LOC_Os04g14710		The expression level of H21 statistically close to T45
MSTRG.22795	LOC_Os06g15760	eukaryotic aspartyl protease domain containing protein, expressed	The expression level of H21 statistically close to T45
MSTRG.26244	LOC_Os07g40860	retrotransposon protein, putative, unclassified, expressed	The expression level of H21 statistically close to T45
MSTRG.15236	LOC_Os03g42500	expressed protein	The expression level of H21 statistically close to T45
MSTRG.28979	LOC_Os09g01310	expressed protein	The expression level of H21 statistically close to T45
MSTRG.7185	LOC_Os11g39430	expressed protein	The expression level of H21 statistically close to T45
MSTRG.7695	LOC_Os12g02589	hydrolase, alpha/beta fold family protein, putative, expressed	The expression level of H21 statistically close to T45
MSTRG.7572	LOC_Os11g47840	OsRhmbd18 - Putative Rhomboid homologue, expressed	The expression level of H21 statistically close to T45
MSTRG.8831			The expression level of H21 statistically close to T45
MSTRG.17613	LOC_Os04g32190	expressed protein	The expression level of H21 statistically close to T45
MSTRG.16832			The expression level of H21 statistically close to T45
MSTRG.24856	LOC_Os07g07780		The expression level of H21 statistically close to T45
MSTRG.25918	LOC_Os07g35920; LOC_Os07g35940	expressed protein	The expression level of H21 statistically close to T45
MSTRG.331	LOC_Os01g05380	expressed protein	The expression level of H21 statistically close to T45
MSTRG.3920	LOC_Os01g72520	phosphoesterase family protein, putative, expressed	The expression level of H21 statistically close to T45
MSTRG.14578	LOC_Os03g23940		The expression level of H21 statistically close to T45
MSTRG.21245	LOC_Os05g41610	glycosyl hydrolases family 17, putative, expressed	The expression level of H21 statistically close to T45
MSTRG.8701	LOC_Os12g27335	expressed protein	The expression level of H21 statistically close to T45
MSTRG.654	LOC_Os01g10700	D-mannose binding lectin family protein, expressed	The expression level of H21 statistically close to T45
MSTRG.11013	LOC_Os02g26800		The expression level of H21 statistically close to T45
MSTRG.2533	LOC_Os01g51550	peroxidase family protein, expressed	The expression level of H21 statistically close to T45
MSTRG.13993	LOC_Os03g14980		The expression level of H21 statistically close to T45
MSTRG.26141	LOC_Os07g39210; LOC_Os07g39190	expressed protein	The expression level of H21 statistically close to T45
MSTRG.16412	LOC_Os03g61290	ATCHX, putative, expressed	The expression level of H21 statistically close to T45
MSTRG.2019	LOC_Os01g42810	leaf senescence related protein, putative, expressed	The expression level of H21 statistically close to T45
MSTRG.6928			The expression level of H21 statistically close to T45
MSTRG.4374	LOC_Os10g11012	expressed protein	The expression level of H21 statistically close to T45
MSTRG.24525	LOC_Os07g01600	dirigent, putative, expressed	The expression level of H21 statistically close to T45

MSTRG.7216			The expression level of H21 statistically close to T45
MSTRG.6986	LOC_Os11g36190	receptor kinase, putative, expressed	The expression level of H21 statistically close to T45
MSTRG.21371	LOC_Os05g43800	expressed protein	The expression level of H21 statistically close to T45
MSTRG.24952	LOC_Os07g09010	nodulin, putative, expressed	The expression level of H21 statistically close to T45
MSTRG.4938	LOC_Os10g30910	transmembrane protein-related, putative, expressed	The expression level of H21 statistically close to T45
MSTRG.4325	LOC_Os10g08940		The expression level of H21 statistically close to T45
MSTRG.4260	LOC_Os10g06770		The expression level of H21 statistically close to T45
MSTRG.14781	LOC_Os03g28250		The expression level of H21 statistically close to T45
MSTRG.22355			The expression level of H21 statistically close to T45
MSTRG.6496	LOC_Os11g17070	expressed protein	The expression level of H21 statistically close to T45
MSTRG.25785	LOC_Os07g33030; LOC_Os07g33040; LOC_Os07g33090	expressed protein	The expression level of H21 statistically close to T45
MSTRG.27225	LOC_Os08g08592	expressed protein	The expression level of H21 statistically close to T45
MSTRG.15505	LOC_Os03g47896	ribosome inactivating protein, putative, expressed	The expression level of H21 statistically close to T45
MSTRG.27096	LOC_Os08g06100	O-methyltransferase, putative, expressed	The expression level of H21 statistically close to T45
MSTRG.12669	LOC_Os02g54890	UDP-glucuronate 4-epimerase, putative, expressed	The expression level of H21 statistically close to T45
MSTRG.14530	LOC_Os03g22670	triacylglycerol Lipase, putative, expressed	The expression level of H21 statistically close to T45
MSTRG.16066	LOC_Os03g56780	expressed protein	The expression level of H21 statistically close to T45
MSTRG.6925	LOC_Os11g34770	maf, putative, expressed	The expression level of H21 statistically close to T45
MSTRG.13987	LOC_Os03g14790	piwi domain containing protein, putative, expressed	The expression level of H21 statistically close to T45
MSTRG.14114			The expression level of H21 statistically close to T45
MSTRG.23503	LOC_Os06g36660; LOC_Os06g36650	retrotransposon protein, putative, unclassified	The expression level of H21 statistically close to T45
MSTRG.30511	LOC_Os09g38490	expressed protein	The expression level of H21 statistically close to T45
MSTRG.26611			The expression level of H21 statistically close to T45
MSTRG.8225			The expression level of H21 statistically close to T45
MSTRG.28301	LOC_Os08g34490	retrotransposon protein, putative, Ty1-copia subclass, expressed	The expression level of H21 statistically close to T45
MSTRG.26074	LOC_Os07g38290	plastocyanin-like domain containing protein, putative, expressed	The expression level of H21 statistically close to T45
MSTRG.27397			The expression level of H21 statistically close to T45
MSTRG.26427	LOC_Os07g44140	cytochrome P450 72A1, putative, expressed	The expression level of H21 statistically close to T45
MSTRG.4399	LOC_Os10g12130	retrotransposon protein, putative, Ty3-gypsy subclass, expressed	The expression level of H21 statistically close to T45
MSTRG.26476	LOC_Os07g45010		The expression level of H21 statistically close to T45
MSTRG.20031	LOC_Os05g11960	expressed protein	The expression level of H21 statistically close to T45
MSTRG.4236	LOC_Os10g05980	POEI13 - Pollen Ole e I allergen and extensin family protein precursor, expressed	The expression level of H21 statistically close to T45
MSTRG.16064	LOC_Os03g56720		The expression level of H21 statistically close to T45
MSTRG.25835	LOC_Os07g33790	glutamate receptor 3.4 precursor, putative, expressed	The expression level of H21 statistically close to T45
MSTRG.27473	LOC_Os08g13430	expressed protein	The expression level of H21 statistically close to T45
MSTRG.22689			The expression level of H21 statistically close to T45
MSTRG.28516	LOC_Os08g38800	retrotransposon protein, putative, unclassified, expressed	The expression level of H21 statistically close to T45
MSTRG.16895	LOC_Os04g11210		The expression level of H21 statistically close to T45
MSTRG.15363	LOC_Os03g45280	dehydrin, putative, expressed	The expression level of H21 statistically close to T45
MSTRG.14026	LOC_Os03g15460	expressed protein	The expression level of H21 statistically close to T45
MSTRG.24662	LOC_Os07g04370	expressed protein	The expression level of H21 statistically close to T45
MSTRG.26606	LOC_Os07g46780		The expression level of H21 statistically close to T45
MSTRG.5479	LOC_Os10g40450	expressed protein	The expression level of H21 statistically close to T45
LOC_Os05g43390	LOC_Os05g43390	signal recognition particle 54 kDa protein, putative, expressed	The expression level of H21 statistically close to T45
MSTRG.24242	LOC_Os06g47880		The expression level of H21 statistically close to T45
MSTRG.14826	LOC_Os03g29864	retrotransposon protein, putative, unclassified, expressed	The expression level of H21 statistically close to T45
MSTRG.27491			The expression level of H21 statistically close to T45
MSTRG.5331	LOC_Os10g38060	phospholipase D, putative, expressed	The expression level of H21 statistically close to T45
MSTRG.1539	LOC_Os01g32630		The expression level of H21 statistically close to T45
MSTRG.12133	LOC_Os02g46850; LOC_Os02g46860	oligopeptide transporter, putative, expressed;oligopeptide transporter 4, putative, expressed	The expression level of H21 statistically close to T45
MSTRG.782	LOC_Os01g12560	3-methyl-2-oxobutanoate hydroxymethyltransferase, putative, expressed	The expression level of H21 statistically close to T45
MSTRG.10820	LOC_Os02g21220	expressed protein	The expression level of H21 statistically close to T45
MSTRG.5740	LOC_Os11g02070	BTB8 - Bric-a-Brac, Tramtrack, Broad Complex BTB domain, expressed	The expression level of H21 statistically close to T45
MSTRG.4254	LOC_Os10g06720	aldose 1-epimerase, putative, expressed	The expression level of H21 statistically close to T45
MSTRG.29008	LOC_Os09g01660		The expression level of H21 statistically close to T45
MSTRG.14319	LOC_Os03g19452	SMP-30/Gluconolaconase/LRE domain protein, putative, expressed	The expression level of H21 statistically close to T45
MSTRG.10021	LOC_Os02g06180		The expression level of H21 statistically close to T45
MSTRG.26117	LOC_Os07g38900		The expression level of H21 statistically close to T45
MSTRG.25944	LOC_Os07g36300		The expression level of H21 statistically close to T45
MSTRG.15595	LOC_Os03g49360	expressed protein	The expression level of H21 statistically close to T45
MSTRG.576	LOC_Os01g09340	expressed protein	The expression level of H21 statistically close to T45

MSTRG.9850	LOC_Os02g04380	expressed protein	The expression level of H21 statistically close to T45
MSTRG.21617	LOC_Os05g47960	expressed protein	The expression level of H21 statistically close to T45
MSTRG.28302	LOC_Os08g34490		The expression level of H21 statistically close to T45
MSTRG.29600	LOC_Os09g21120	armadillo/beta-catenin repeat family protein, putative, expressed	The expression level of H21 statistically close to T45
MSTRG.10902			The expression level of H21 statistically close to T45
MSTRG.28818	LOC_Os08g43220		The expression level of H21 statistically close to T45
MSTRG.15737	LOC_Os03g51490	expressed protein	The expression level of H21 statistically close to T45
MSTRG.27829	LOC_Os08g23950		The expression level of H21 statistically close to T45
MSTRG.11136	LOC_Os02g29510		The expression level of H21 statistically close to T45
MSTRG.20178			The expression level of H21 statistically close to T45
MSTRG.14078	LOC_Os03g16090	LIM domain-containing protein, putative, expressed	The expression level of H21 statistically close to T45
MSTRG.11671	LOC_Os02g39260	cyclin, putative, expressed	The expression level of H21 statistically close to T45
MSTRG.10791	LOC_Os02g20500		The expression level of H21 statistically close to T45
MSTRG.29699	LOC_Os09g23620	MYB family transcription factor, putative, expressed	The expression level of H21 statistically close to T45
MSTRG.22881			The expression level of H21 statistically close to T45
MSTRG.8565	LOC_Os12g23470; LOC_Os12g23540		The expression level of H21 statistically close to T45
MSTRG.14132	LOC_Os03g16920	DnaK family protein, putative, expressed	The expression level of H21 statistically close to T45
MSTRG.29915	LOC_Os09g27950	galactosyltransferase, putative, expressed	The expression level of H21 statistically close to T45
MSTRG.24454	LOC_Os06g50706	expressed protein	The expression level of H21 statistically close to T45
MSTRG.11157	LOC_Os02g30080	cytochrome P450, putative, expressed	The expression level of H21 statistically close to T45
MSTRG.8669			The expression level of H21 statistically close to T45
MSTRG.9208	LOC_Os12g38910	expressed protein	The expression level of H21 statistically close to T45
MSTRG.11015	LOC_Os02g26850	rRNA-processing protein FCF, putative, expressed	The expression level of H21 statistically close to T45
MSTRG.1591	LOC_Os01g33684	disease resistance RPP13-like protein 1, putative, expressed	The expression level of H21 statistically close to T45
MSTRG.4232	LOC_Os10g05930	POEI10 - Pollen Ole e I allergen and extensin family protein precursor, expressed	The expression level of H21 statistically close to T45
MSTRG.4235	LOC_Os10g05970	POEI12 - Pollen Ole e I allergen and extensin family protein precursor, expressed	The expression level of H21 statistically close to T45
MSTRG.24855	LOC_Os07g07780	expressed protein	The expression level of H21 statistically close to T45
MSTRG.17519	LOC_Os04g30620		The expression level of H21 statistically close to T45
MSTRG.18637	LOC_Os04g48370	RNA polymerase IV subunit, putative, expressed	The expression level of H21 statistically close to T45
MSTRG.10635	LOC_Os02g17340		The expression level of H21 statistically close to T45
MSTRG.24456	LOC_Os06g50742	expressed protein	The expression level of H21 statistically close to T45
MSTRG.5971	LOC_Os11g05390	transporter, major facilitator family, putative, expressed	The expression level of H21 statistically close to T45
MSTRG.22948			The expression level of H21 statistically close to T45
MSTRG.12701	LOC_Os02g55280		The expression level of H21 statistically close to T45
MSTRG.1487	LOC_Os01g29804	expressed protein	The expression level of H21 statistically close to T45
MSTRG.17523	LOC_Os04g30750	expressed protein	The expression level of H21 statistically close to T45
MSTRG.3114	LOC_Os01g59720	expressed protein	The expression level of H21 statistically close to T45
MSTRG.25690	LOC_Os07g31200		The expression level of H21 statistically close to T45
MSTRG.7121	LOC_Os11g38630	expressed protein	The expression level of H21 statistically close to T45
MSTRG.8755			The expression level of H21 statistically close to T45
MSTRG.11682	LOC_Os02g39360	B-box zinc finger family protein, putative, expressed	The expression level of H21 statistically close to T45
MSTRG.15503	LOC_Os03g47820		The expression level of H21 statistically close to T45
MSTRG.25002	LOC_Os07g09870	OsFBA2 - F-box and FBA domain containing protein, expressed	The expression level of H21 statistically close to T45
MSTRG.19977	LOC_Os05g10650		The expression level of H21 statistically close to T45
MSTRG.10612	LOC_Os02g16620	reticulon domain containing protein, putative, expressed	The expression level of H21 statistically close to T45
MSTRG.23431	LOC_Os06g34790	DUF538 domain containing protein, putative, expressed	The expression level of H21 statistically close to T45
MSTRG.5515	LOC_Os10g41070	glycerol-3-phosphate acyltransferase 1, putative, expressed	The expression level of H21 statistically close to T45
MSTRG.18415	LOC_Os04g44870	C2 domain containing protein, putative, expressed	The expression level of H21 statistically close to T45
MSTRG.8473	LOC_Os12g20310	sarcoma antigen NY-SAR-91, putative, expressed	The expression level of H21 statistically close to T45
MSTRG.11179	LOC_Os02g30280		The expression level of H21 statistically close to T45
MSTRG.27490	LOC_Os08g13710	expressed protein	The expression level of H21 statistically close to T45
MSTRG.10670	LOC_Os02g18180	ATP-binding cassette sub-family E member 1, putative, expressed	The expression level of H21 statistically close to T45
MSTRG.793	LOC_Os01g12810	retrotransposon protein, putative, unclassified, expressed	The expression level of H21 statistically close to T45
MSTRG.13662	LOC_Os03g10478	glycosyl hydrolase family 10 protein, putative, expressed	The expression level of H21 statistically close to T45
MSTRG.24853			The expression level of H21 statistically close to T45
MSTRG.24551	LOC_Os07g01990	expressed protein	The expression level of H21 statistically close to T45
MSTRG.10532	LOC_Os02g14520		The expression level of H21 statistically close to T45
MSTRG.26988	LOC_Os08g04140	X8 domain containing protein, expressed	The expression level of H21 statistically close to T45
MSTRG.19961	LOC_Os05g10420; LOC_Os05g10440; LOC_Os05g10480	expressed protein	The expression level of H21 statistically close to T45

MSTRG.7747	LOC_Os12g03390	OsFBX435 - F-box domain containing protein, expressed	The expression level of H21 statistically close to T45
MSTRG.28658	LOC_Os08g40910	expressed protein	The expression level of H21 statistically close to T45
MSTRG.22760			The expression level of H21 statistically close to T45
MSTRG.17590	LOC_Os04g31880		The expression level of H21 statistically close to T45
MSTRG.28499	LOC_Os08g38400	oligopeptide transporter, putative, expressed	The expression level of H21 statistically close to T45
MSTRG.25999	LOC_Os07g37110	nucleoside transporter, putative, expressed	The expression level of H21 statistically close to T45
MSTRG.12983	LOC_Os02g58740		The expression level of H21 statistically close to T45
MSTRG.15214	LOC_Os03g42070	cyclin, putative, expressed	The expression level of H21 statistically close to T45
MSTRG.17191	LOC_Os04g20330; LOC_Os04g20360; LOC_Os04g20400	UDP-glucoronosyl/UDP-glucosyl transferase, putative, expressed;cytokinin-O-glucosyltransferase 1, putative, expressed	The expression level of H21 statistically close to T45
MSTRG.3360	LOC_Os01g63854	transmembrane amino acid transporter protein, putative, expressed	The expression level of H21 statistically close to T45
MSTRG.17766	LOC_Os04g34460	PAP fibrillin family domain containing protein, expressed	The expression level of H21 statistically close to T45
MSTRG.10669	LOC_Os02g18150	expressed protein	The expression level of H21 statistically close to T45
MSTRG.4296			The expression level of H21 statistically close to T45
MSTRG.15115	LOC_Os03g39660	expressed protein	The expression level of H21 statistically close to T45
MSTRG.10715	LOC_Os02g19070		The expression level of H21 statistically close to T45
MSTRG.29653	LOC_Os09g22410	pyruvate kinase-like, putative, expressed	The expression level of H21 statistically close to T45
MSTRG.28723	LOC_Os08g41880	nucleotide pyrophosphatase/phosphodiesterase, putative, expressed	The expression level of H21 statistically close to T45
MSTRG.24177	LOC_Os06g46900	phosphosulfolactate synthase-related protein, putative, expressed	The expression level of H21 statistically close to T45
MSTRG.18537	LOC_Os04g46690		The expression level of H21 statistically close to T45
MSTRG.10394	LOC_Os02g12380	histone deacetylase, putative, expressed	The expression level of H21 statistically close to T45
MSTRG.10741			The expression level of H21 statistically close to T45
MSTRG.20528	LOC_Os05g28290	ranBP1 domain containing protein, expressed	The expression level of H21 statistically close to T45
MSTRG.17576	LOC_Os04g31524	expressed protein	The expression level of H21 statistically close to T45
MSTRG.26523	LOC_Os07g45550	expressed protein	The expression level of H21 statistically close to T45
MSTRG.14279	LOC_Os03g18910	COBRA-like protein 7 precursor, putative, expressed	The expression level of H21 statistically close to T45
MSTRG.22671	LOC_Os06g13030	OsLIM - LIM domain protein, putative actin-binding protein and transcription factor, expressed	The expression level of H21 statistically close to T45
MSTRG.27228	LOC_Os08g08680		The expression level of H21 statistically close to T45
MSTRG.11027	LOC_Os02g27000	ATP-binding region, ATPase-like domain containing protein, expressed	The expression level of H21 statistically close to T45
MSTRG.6863	LOC_Os11g33030	retrotransposon protein, putative, unclassified, expressed	The expression level of H21 statistically close to T45
MSTRG.23433	LOC_Os06g34810		The expression level of H21 statistically close to T45
MSTRG.22610	LOC_Os06g12129	expressed protein	The expression level of H21 statistically close to T45
MSTRG.23866	LOC_Os06g43520	cytochrome P450 71D7, putative, expressed	The expression level of H21 statistically close to T45
MSTRG.10903	LOC_Os02g23930	expressed protein	The expression level of H21 statistically close to T45
LOC_Os02g49790	LOC_Os02g49790	aluminum-activated malate transporter, putative, expressed	The expression level of H21 statistically close to T45
MSTRG.19031	LOC_Os04g54240	wound induced protein, putative, expressed	The expression level of H21 statistically close to T45
MSTRG.21947	LOC_Os06g01850	ferredoxin--NADP reductase, chloroplast precursor, putative, expressed	The expression level of H21 statistically close to T45
MSTRG.26105	LOC_Os07g38630		The expression level of H21 statistically close to T45
MSTRG.24775	LOC_Os07g06500	OsFBL34 - F-box domain and LRR containing protein, expressed	The expression level of H21 statistically close to T45
MSTRG.7261			The expression level of H21 statistically close to T45
MSTRG.464	LOC_Os01g07660	expressed protein	The expression level of H21 statistically close to T45
MSTRG.15114	LOC_Os03g39655	expressed protein	The expression level of H21 statistically close to T45
MSTRG.10425			The expression level of H21 statistically close to T45
MSTRG.29533	LOC_Os09g19710	hypersensitive-induced response protein, putative, expressed	The expression level of H21 statistically close to T45
MSTRG.28743	LOC_Os08g42189	expressed protein	The expression level of H21 statistically close to T45
MSTRG.6702			The expression level of H21 statistically close to T45
MSTRG.3309	LOC_Os01g62870	oxidoreductase, aldo/keto reductase family protein, putative, expressed	The expression level of H21 statistically close to T45
MSTRG.14357	LOC_Os03g19920	expressed protein	The expression level of H21 statistically close to T45
MSTRG.22193	LOC_Os06g05610	OsFBDUF32 - F-box and DUF domain containing protein, expressed	The expression level of H21 statistically close to T45
MSTRG.17906	LOC_Os04g36800	3-oxoacyl-synthase, putative, expressed	The expression level of H21 statistically close to T45
MSTRG.26108			The expression level of H21 statistically close to T45
MSTRG.11151	LOC_Os02g29620	expressed protein	The expression level of H21 statistically close to T45
MSTRG.4237	LOC_Os10g05990	POEI14 - Pollen Ole e I allergen and extensin family protein precursor, expressed	The expression level of H21 statistically close to T45
MSTRG.25674	LOC_Os07g30800		The expression level of H21 statistically close to T45
MSTRG.1106	LOC_Os01g18320	protoporphyrinogen oxidase, chloroplast precursor, putative, expressed	The expression level of H21 statistically close to T45

MSTRG.12272	LOC_Os02g48950	ubiquitin-conjugating enzyme, putative, expressed	The expression level of H21 statistically close to T45
MSTRG.11038	LOC_Os02g27360	aspartic proteinase-like protein 2 precursor, putative, expressed	The expression level of H21 statistically close to T45
MSTRG.10341			The expression level of H21 was between T44 and T45
MSTRG.22060			The expression level of H21 was between T44 and T45
MSTRG.13502	LOC_Os03g08310	ZIM domain containing protein, putative, expressed	The expression level of H21 was between T44 and T45
MSTRG.11601	LOC_Os02g38260	glycosyl hydrolase family 5 protein, putative, expressed	The expression level of H21 was between T44 and T45
MSTRG.18451	LOC_Os04g45370	OsSAUR19 - Auxin-responsive SAUR gene family member, expressed	The expression level of H21 was between T44 and T45
MSTRG.22726			The expression level of H21 was between T44 and T45
MSTRG.21654	LOC_Os05g48422	60S ribosomal protein-related, putative, expressed	The expression level of H21 was between T44 and T45
MSTRG.16407	LOC_Os03g61240	expressed protein	The expression level of H21 was between T44 and T45
MSTRG.20431	LOC_Os05g26840	permease domain containing protein, putative, expressed	The expression level of H21 was between T44 and T45
MSTRG.19718	LOC_Os05g05920		The expression level of H21 was between T44 and T45
MSTRG.24187	LOC_Os06g47120	expressed protein	The expression level of H21 was between T44 and T45
MSTRG.7220	LOC_Os11g39940	transposon protein, putative, unclassified, expressed	The expression level of H21 was between T44 and T45
MSTRG.14583			The expression level of H21 was between T44 and T45
MSTRG.16736	LOC_Os04g06850	expressed protein	The expression level of H21 was between T44 and T45
MSTRG.7733	LOC_Os12g03130	CDC45A - Putative DNA replication initiation protein, expressed	The expression level of H21 was between T44 and T45
MSTRG.7349			The expression level of H21 was between T44 and T45
MSTRG.20744	LOC_Os05g32710	Alpha amylase, catalytic domain containing protein, expressed	The expression level of H21 was between T44 and T45
MSTRG.19970	LOC_Os05g10570	expressed protein	The expression level of H21 was between T44 and T45
MSTRG.15357	LOC_Os03g45220	expressed protein	The expression level of H21 was between T44 and T45
MSTRG.4223	LOC_Os10g05720	LTPL37 - Protease inhibitor/seed storage/LTP family protein precursor, expressed	The expression level of H21 was between T44 and T45
MSTRG.677	LOC_Os01g10890	CAMK_KIN1/SNF1/Nim1_like.8 - CAMK includes calcium/calmodulin depedent protein kinases, expressed	The expression level of H21 was between T44 and T45
MSTRG.9926	LOC_Os02g04950	splicing factor 3B subunit 1, putative, expressed	The expression level of H21 was between T44 and T45
MSTRG.23848			The expression level of H21 was between T44 and T45
MSTRG.26923	LOC_Os08g02990;<br>LOC_Os08g02996	retrotransposon protein, putative, unclassified, expressed;receptor-like kinase, putative, expressed	The expression level of H21 was between T44 and T45
MSTRG.10456	LOC_Os02g12960	expressed protein	The expression level of H21 was between T44 and T45
MSTRG.8611	LOC_Os12g24659		The expression level of H21 was between T44 and T45
MSTRG.14977	LOC_Os03g36550	transposon protein, putative, unclassified, expressed	The expression level of H21 was between T44 and T45
MSTRG.23449	LOC_Os06g35520	peroxidase precursor, putative, expressed	The expression level of H21 was between T44 and T45
MSTRG.22050	LOC_Os06g03930	cytochrome P450 86A1, putative, expressed	The expression level of H21 was between T44 and T45
MSTRG.28113	LOC_Os08g31360	glycine-rich protein, putative, expressed	The expression level of H21 was between T44 and T45
MSTRG.26618	LOC_Os07g46920	sex determination protein tasselseed-2, putative, expressed	The expression level of H21 was between T44 and T45
MSTRG.6302	LOC_Os11g11810	NBS-LRR disease resistance protein, putative, expressed	The expression level of H21 was between T44 and T45
MSTRG.1974	LOC_Os01g42140	expressed protein	The expression level of H21 was between T44 and T45
MSTRG.11799	LOC_Os02g41670	phenylalanine ammonia-lyase, putative, expressed	The expression level of H21 was between T44 and T45
MSTRG.19833	LOC_Os05g07300	serine/threonine-protein kinase receptor precursor, putative, expressed	The expression level of H21 was between T44 and T45
MSTRG.20966	LOC_Os05g36350	pentatricopeptide, putative, expressed	The expression level of H21 was between T44 and T45
MSTRG.19892	LOC_Os05g08540	gibberellin 3-beta-dioxygenase 2-2, putative, expressed	The expression level of H21 was between T44 and T45
MSTRG.3074	LOC_Os01g59265		The expression level of H21 was between T44 and T45
MSTRG.3762	LOC_Os01g70130		The expression level of H21 was between T44 and T45
MSTRG.17745	LOC_Os04g34170		The expression level of H21 was between T44 and T45
MSTRG.10068	LOC_Os02g06790	expressed protein	The expression level of H21 was between T44 and T45
MSTRG.21177	LOC_Os05g40620	expressed protein	The expression level of H21 was between T44 and T45
MSTRG.13817			The expression level of H21 was between T44 and T45
MSTRG.19400	LOC_Os04g59630		The expression level of H21 was between T44 and T45
MSTRG.24266	LOC_Os06g48200	glycosyl hydrolases family 16, putative, expressed	The expression level of H21 was between T44 and T45
MSTRG.18015	LOC_Os04g38840	LTPL81 - Protease inhibitor/seed storage/LTP family protein precursor, expressed	The expression level of H21 was between T44 and T45
MSTRG.22245			The expression level of H21 was between T44 and T45
MSTRG.20904			The expression level of H21 was between T44 and T45
MSTRG.25022	LOC_Os07g10310	expressed protein	The expression level of H21 was between T44 and T45
MSTRG.18116	LOC_Os04g40190	circumsporozoite protein, putative, expressed	The expression level of H21 was between T44 and T45
MSTRG.17447	LOC_Os04g29080		The expression level of H21 was between T44 and T45
MSTRG.7517	LOC_Os11g46300	expressed protein	The expression level of H21 was between T44 and T45
MSTRG.17389	LOC_Os04g27670	terpene synthase family, metal binding domain containing protein, expressed	The expression level of H21 was between T44 and T45

MSTRG.23442	LOC_Os06g35140	MYB family transcription factor, putative, expressed	The expression level of H21 was between T44 and T45
MSTRG.27142	LOC_Os08g06700	expressed protein	The expression level of H21 was between T44 and T45
MSTRG.9438			The expression level of H21 was between T44 and T45
MSTRG.4769			The expression level of H21 was between T44 and T45
MSTRG.17282	LOC_Os04g23700		The expression level of H21 was between T44 and T45
MSTRG.25920	LOC_Os07g36010	transposon protein, putative, unclassified, expressed	The expression level of H21 was between T44 and T45
MSTRG.9671	LOC_Os02g02260		The expression level of H21 was between T44 and T45
MSTRG.25127	LOC_Os07g12304	expressed protein	The expression level of H21 was between T44 and T45
MSTRG.17352	LOC_Os04g25740	expressed protein	The expression level of H21 was between T44 and T45
MSTRG.17873	LOC_Os04g35870	retrotransposon protein, putative, unclassified, expressed	The expression level of H21 was between T44 and T45
MSTRG.2607	LOC_Os01g52810	expressed protein	The expression level of H21 was between T44 and T45
LOC_Os01g57270	LOC_Os01g57270	disease resistance RPP13-like protein 1, putative, expressed	The expression level of H21 was between T44 and T45
MSTRG.15089	LOC_Os03g39510	expressed protein	The expression level of H21 was between T44 and T45
MSTRG.7466	LOC_Os11g44960	NBS-LRR disease resistance protein, putative, expressed	The expression level of H21 was between T44 and T45
MSTRG.21044	LOC_Os05g38350	glycerol-3-phosphate acyltransferase 8, putative, expressed	The expression level of H21 was between T44 and T45
MSTRG.23367	LOC_Os06g32500	retrotransposon protein, putative, Ty3-gypsy subclass, expressed	The expression level of H21 was between T44 and T45
MSTRG.5149	LOC_Os10g35160	expressed protein	The expression level of H21 was between T44 and T45
MSTRG.5992	LOC_Os11g05840	retrotransposon protein, putative, Ty1-copia subclass, expressed	The expression level of H21 was between T44 and T45
MSTRG.1955	LOC_Os01g41930	leucine rich repeat protein, putative, expressed	The expression level of H21 was between T44 and T45
MSTRG.4943	LOC_Os10g30944		The expression level of H21 was between T44 and T45
MSTRG.6927	LOC_Os11g34790	expressed protein	The expression level of H21 was between T44 and T45
MSTRG.4706			The expression level of H21 was between T44 and T45
MSTRG.9070			The expression level of H21 was between T44 and T45
MSTRG.24162	LOC_Os06g46650	hypothetical protein	The expression level of H21 was between T44 and T45
MSTRG.3940			The expression level of H21 was between T44 and T45
MSTRG.19645	LOC_Os05g04680	expressed protein	The expression level of H21 was between T44 and T45
MSTRG.25668	LOC_Os07g30590	LTPL55 - Protease inhibitor/seed storage/LTP family protein precursor, putative, expressed	The expression level of H21 was between T44 and T45
MSTRG.905	LOC_Os01g14670	Cupin domain containing protein, expressed	The expression level of H21 was between T44 and T45
MSTRG.14584			The expression level of H21 was between T44 and T45
MSTRG.4061	LOC_Os01g74670		The expression level of H21 was between T44 and T45
MSTRG.24547	LOC_Os07g01960	hypothetical protein	The expression level of H21 was between T44 and T45
MSTRG.4192	LOC_Os10g05180	26S proteasome regulatory subunit S5A, putative, expressed	The expression level of H21 was between T44 and T45
MSTRG.17871	LOC_Os04g35860	T-complex protein 11, putative, expressed	The expression level of H21 was between T44 and T45
MSTRG.29942	LOC_Os09g28370	retrotransposon protein, putative, unclassified, expressed	The expression level of H21 was between T44 and T45
MSTRG.20084	LOC_Os05g13910; LOC_Os05g13940		The expression level of H21 was between T44 and T45
MSTRG.3356	LOC_Os01g63840		The expression level of H21 was between T44 and T45
MSTRG.28745			The expression level of H21 was between T44 and T45
MSTRG.5838	LOC_Os11g03420		The expression level of H21 was between T44 and T45
MSTRG.14348			The expression level of H21 was between T44 and T45
MSTRG.23703	LOC_Os06g40530	transposon protein, putative, CACTA, En/Spm sub-class, expressed	The expression level of H21 was between T44 and T45
MSTRG.6316	LOC_Os11g12490	transposon protein, putative, unclassified, expressed	The expression level of H21 was between T44 and T45
MSTRG.8590	LOC_Os12g24020	rhodanese-like domain containing protein, putative, expressed	The expression level of H21 was between T44 and T45
MSTRG.264	LOC_Os01g03670	dihydroflavonol-4-reductase, putative, expressed	The expression level of H21 was between T44 and T45
MSTRG.5833	LOC_Os11g03300	NAC domain transcription factor, putative, expressed	The expression level of H21 was between T44 and T45
MSTRG.22495	LOC_Os06g10100	expressed protein	The expression level of H21 was between T44 and T45
MSTRG.13669	LOC_Os03g10570		The expression level of H21 was between T44 and T45
MSTRG.2522	LOC_Os01g51370	expressed protein	The expression level of H21 was between T44 and T45
MSTRG.24133	LOC_Os06g46434	cytochrome c biogenesis protein ccsA, putative, expressed	The expression level of H21 was between T44 and T45
MSTRG.17247			The expression level of H21 was between T44 and T45
MSTRG.26756			The expression level of H21 was between T44 and T45
MSTRG.27194			The expression level of H21 was between T44 and T45
MSTRG.29399	LOC_Os09g15250	retrotransposon protein, putative, unclassified, expressed	The expression level of H21 was between T44 and T45
MSTRG.24670	LOC_Os07g04580	zinc finger C-x8-C-x5-C-x3-H type family protein, expressed	The expression level of H21 was between T44 and T45
MSTRG.24195	LOC_Os06g47260; LOC_Os06g47270	ras-related protein, putative, expressed;zinc finger, C3HC4 type domain containing protein, expressed	The expression level of H21 was between T44 and T45
MSTRG.1899	LOC_Os01g41050	sulfate transporter, putative, expressed	The expression level of H21 was between T44 and T45
MSTRG.598	LOC_Os01g09570		The expression level of H21 was between T44 and T45
MSTRG.11672	LOC_Os02g39220; LOC_Os02g39230; LOC_Os02g39240	transposon protein, putative, unclassified, expressed;cyclin-F1-4, putative, expressed;cyclin, putative, expressed	The expression level of H21 was between T44 and T45

MSTRG.8270	LOC_Os12g14059	expressed protein	The expression level of H21 was between T44 and T45
MSTRG.28629	LOC_Os08g40440; LOC_Os08g40450	dihydroflavonol-4-reductase, putative, expressed;uvrB/uvrC motif family protein, expressed	The expression level of H21 was between T44 and T45
MSTRG.7151	LOC_Os11g38850	TKL_IRAK_DUF26-la.7 - DUF26 kinases have homology to DUF26 containing loci, expressed	The expression level of H21 was between T44 and T45
MSTRG.2476	LOC_Os01g50780		The expression level of H21 was between T44 and T45
MSTRG.20852	LOC_Os05g34390	protein kinase domain containing protein, expressed	The expression level of H21 was between T44 and T45
MSTRG.21445	LOC_Os05g45170	glucosyl transferase, putative, expressed	The expression level of H21 was between T44 and T45
MSTRG.16827	LOC_Os04g08280	retrotransposon protein, putative, Ty3-gypsy subclass, expressed	The expression level of H21 was between T44 and T45
MSTRG.25821			The expression level of H21 was between T44 and T45
MSTRG.407	LOC_Os01g06640	basic helix-loop-helix, putative, expressed	The expression level of H21 was between T44 and T45
MSTRG.24958	LOC_Os07g09120		The expression level of H21 was between T44 and T45
MSTRG.6458	LOC_Os11g15620	OsFBX420 - F-box domain containing protein, expressed	The expression level of H21 was between T44 and T45
MSTRG.16842	LOC_Os04g08800	expressed protein	The expression level of H21 was between T44 and T45
MSTRG.7345			The expression level of H21 was between T44 and T45
MSTRG.4949	LOC_Os10g31360	GRF zinc finger family protein, expressed	The expression level of H21 was between T44 and T45
MSTRG.17474			The expression level of H21 was between T44 and T45
LOC_Os01g22190	LOC_Os01g22190	transposon protein, putative, CACTA, En/Spm sub-class, expressed	The expression level of H21 was between T44 and T45
MSTRG.25657	LOC_Os07g30150		The expression level of H21 was between T44 and T45
MSTRG.231	LOC_Os01g03320	BBTI2 - Bowman-Birk type bran trypsin inhibitor precursor, expressed	The expression level of H21 was between T44 and T45
MSTRG.20711	LOC_Os05g32290	retrotransposon protein, putative, Ty1-copia subclass, expressed	The expression level of H21 was between T44 and T45
MSTRG.6372			The expression level of H21 was between T44 and T45
MSTRG.25997	LOC_Os07g37090	ribosome inactivating protein, putative, expressed	The expression level of H21 was between T44 and T45
MSTRG.10779	LOC_Os02g20290	expressed protein	The expression level of H21 was between T44 and T45
MSTRG.5503	LOC_Os10g40830	metalloendoproteinase 1 precursor, putative, expressed	The expression level of H21 was between T44 and T45
MSTRG.26941	LOC_Os08g03570		The expression level of H21 was between T44 and T45
MSTRG.3761			The expression level of H21 was between T44 and T45
MSTRG.4391			The expression level of H21 was between T44 and T45
MSTRG.8279	LOC_Os12g14150	expressed protein	The expression level of H21 was between T44 and T45
MSTRG.1944	LOC_Os01g41790	expressed protein	The expression level of H21 was between T44 and T45
MSTRG.658	LOC_Os01g10720	retrotransposon protein, putative, unclassified, expressed	The expression level of H21 was between T44 and T45
MSTRG.30226	LOC_Os09g33720	protein transport protein Sec61, putative, expressed	The expression level of H21 was between T44 and T45
MSTRG.1961	LOC_Os01g41960	retrotransposon protein, putative, unclassified, expressed	The expression level of H21 was between T44 and T45
MSTRG.30565	LOC_Os09g39300	retrotransposon protein, putative, unclassified, expressed	The expression level of H21 was between T44 and T45
MSTRG.29563	LOC_Os09g20260	polyamine oxidase precursor, putative, expressed	The expression level of H21 was between T44 and T45
MSTRG.19211	LOC_Os04g57060	retrotransposon protein, putative, unclassified, expressed	The expression level of H21 was between T44 and T45
MSTRG.22891	LOC_Os06g18010		The expression level of H21 was between T44 and T45
MSTRG.6701			The expression level of H21 was between T44 and T45
MSTRG.3879	LOC_Os01g71840	retrotransposon protein, putative, unclassified, expressed	The expression level of H21 was between T44 and T45
MSTRG.15168	LOC_Os03g40720		The expression level of H21 was between T44 and T45
MSTRG.29284	LOC_Os09g10980	ZOS9-02 - C2H2 zinc finger protein, expressed	The expression level of H21 was between T44 and T45
MSTRG.23864	LOC_Os06g43410	cytochrome P450, putative, expressed	The expression level of H21 was between T44 and T45
MSTRG.1963	LOC_Os01g41990	OsCML12 - Calmodulin-related calcium sensor protein, expressed	The expression level of H21 was between T44 and T45
MSTRG.17241	LOC_Os04g21850		The expression level of H21 was between T44 and T45
MSTRG.17433	LOC_Os04g28590	expressed protein	The expression level of H21 was between T44 and T45
MSTRG.3139	LOC_Os01g60020	NAC domain transcription factor, putative, expressed	The expression level of H21 was between T44 and T45
MSTRG.24737			The expression level of H21 was between T44 and T45
MSTRG.9037	LOC_Os12g35920		The expression level of H21 was between T44 and T45
MSTRG.16943	LOC_Os04g12760		The expression level of H21 was between T44 and T45
MSTRG.13336	LOC_Os03g05880	monooxygenase, putative, expressed	The expression level of H21 was between T44 and T45
MSTRG.4600			The expression level of H21 was between T44 and T45
MSTRG.18683	LOC_Os04g49230		The expression level of H21 was between T44 and T45
MSTRG.7536	LOC_Os11g47400	expressed protein	The expression level of H21 was between T44 and T45
MSTRG.7493			The expression level of H21 was between T44 and T45
MSTRG.8970			The expression level of H21 was between T44 and T45
MSTRG.22713	LOC_Os06g13850	OsFBX193 - F-box domain containing protein, expressed	The expression level of H21 was between T44 and T45
MSTRG.13641	LOC_Os03g10240	DUF677 domain containing protein, putative, expressed	The expression level of H21 was between T44 and T45
MSTRG.22160	LOC_Os06g05180		The expression level of H21 was between T44 and T45
LOC_Os07g35480	LOC_Os07g35480	glucan endo-1,3-beta-glucosidase precursor, putative, expressed	The expression level of H21 was between T44 and T45

MSTRG.18047	LOC_Os04g39150	pathogenesis-related Bet v I family protein, putative, expressed	The expression level of H21 was between T44 and T45
MSTRG.7863	LOC_Os12g05210	expressed protein	The expression level of H21 was between T44 and T45
MSTRG.22848			The expression level of H21 was between T44 and T45
MSTRG.4554	LOC_Os10g20530		The expression level of H21 was between T44 and T45
MSTRG.13520			The expression level of H21 was between T44 and T45
LOC_Os11g47160	LOC_Os11g47160	receptor kinase 1, putative, expressed	The expression level of H21 was between T44 and T45
MSTRG.24827	LOC_Os07g07320	glutathione S-transferase, putative, expressed	The expression level of H21 was between T44 and T45
MSTRG.28676			The expression level of H21 was between T44 and T45
MSTRG.29079	LOC_Os09g03680	ankyrin, putative, expressed	The expression level of H21 was between T44 and T45
MSTRG.2058			The expression level of H21 was between T44 and T45
MSTRG.9304			The expression level of H21 was between T44 and T45
LOC_Os12g26020	LOC_Os12g26020	expressed protein	The expression level of H21 was between T44 and T45
MSTRG.22853	LOC_Os06g17040	retrotransposon protein, putative, unclassified, expressed	The expression level of H21 was between T44 and T45
MSTRG.25150	LOC_Os07g12550	expressed protein	The expression level of H21 was between T44 and T45
MSTRG.15471	LOC_Os03g47460		The expression level of H21 was between T44 and T45
MSTRG.3451			The expression level of H21 was between T44 and T45
MSTRG.4246	LOC_Os10g06510	cyclin-dependent kinase G-1, putative, expressed	The expression level of H21 was between T44 and T45
MSTRG.16955	LOC_Os04g12890	retrotransposon protein, putative, unclassified, expressed	The expression level of H21 was between T44 and T45
MSTRG.7520	LOC_Os11g46970	receptor-like protein kinase 5 precursor, putative, expressed	The expression level of H21 was between T44 and T45
MSTRG.25078	LOC_Os07g10970	leucine zipper protein-like, putative, expressed	The expression level of H21 was between T44 and T45
LOC_Os07g48620	LOC_Os07g48620	expressed protein	The expression level of H21 was between T44 and T45
MSTRG.26757	LOC_Os07g48980	nicotianamine synthase, putative, expressed	The expression level of H21 was between T44 and T45
MSTRG.17459	LOC_Os04g29310	retrotransposon protein, putative, unclassified, expressed	The expression level of H21 was between T44 and T45
MSTRG.26770	LOC_Os07g49110		The expression level of H21 was between T44 and T45
MSTRG.9799	LOC_Os02g03710	UP-9A, putative, expressed	The expression level of H21 was between T44 and T45
MSTRG.23020	LOC_Os06g21740	retrotransposon protein, putative, Ty3-gypsy subclass, expressed	The expression level of H21 was between T44 and T45
MSTRG.22573	LOC_Os06g11490	plastocyanin-like domain containing protein, putative, expressed	The expression level of H21 was between T44 and T45
MSTRG.22201	LOC_Os06g05680	retrotransposon protein, putative, unclassified, expressed	The expression level of H21 was between T44 and T45
MSTRG.28307			The expression level of H21 was between T44 and T45
MSTRG.17805	LOC_Os04g34930		The expression level of H21 was between T44 and T45
MSTRG.1426			The expression level of H21 was between T44 and T45
MSTRG.21444	LOC_Os05g45150	anthocyanidin 5,3-O-glucosyltransferase, putative, expressed	The expression level of H21 was between T44 and T45
MSTRG.2877	LOC_Os01g56530	DUF260 domain containing protein, putative, expressed	The expression level of H21 was between T44 and T45
MSTRG.7541			The expression level of H21 was between T44 and T45
MSTRG.11776	LOC_Os02g40940	expressed protein	The expression level of H21 was between T44 and T45
MSTRG.20106			The expression level of H21 was between T44 and T45
MSTRG.26998			The expression level of H21 was between T44 and T45
MSTRG.26333	LOC_Os07g42590	OsFBX258 - F-box domain containing protein, expressed	The expression level of H21 was between T44 and T45
MSTRG.1266	LOC_Os01g22450		The expression level of H21 was between T44 and T45
MSTRG.6764	LOC_Os11g29920	NB-ARC domain containing protein, expressed	The expression level of H21 was between T44 and T45
MSTRG.22835			The expression level of H21 was between T44 and T45
MSTRG.13639			The expression level of H21 was between T44 and T45
MSTRG.28964	LOC_Os08g45130	histone-lysine N-methyltransferase, putative, expressed	The expression level of H21 was between T44 and T45
MSTRG.9502	LOC_Os12g42910	sodium/calcium exchanger protein, putative, expressed	The expression level of H21 was between T44 and T45
MSTRG.9239			The expression level of H21 was between T44 and T45
MSTRG.14143	LOC_Os03g17100	Core histone H2A/H2B/H3/H4 domain containing protein, putative, expressed	The expression level of H21 was between T44 and T45
MSTRG.1968	LOC_Os01g42090	nodulin MtN3 family protein, putative, expressed	The expression level of H21 was between T44 and T45
MSTRG.7139	LOC_Os11g38790	expressed protein	The expression level of H21 was between T44 and T45
MSTRG.25923	LOC_Os07g36040;LOC_Os07g36060	strictosidine synthase 1 precursor, putative, expressed;SMP-30/Gluconolaconase/LRE-like region containing protein, expressed	The expression level of H21 was between T44 and T45
MSTRG.17269	LOC_Os04g23140	expressed protein	The expression level of H21 was between T44 and T45
MSTRG.2216	LOC_Os01g46660	transposon protein, putative, unclassified, expressed	The expression level of H21 was between T44 and T45
MSTRG.24475	LOC_Os06g51290	phytoene synthase, chloroplast precursor, putative, expressed	The expression level of H21 was between T44 and T45
MSTRG.25070	LOC_Os07g10850	retrotransposon protein, putative, unclassified, expressed	The expression level of H21 was between T44 and T45
MSTRG.2559	LOC_Os01g52130	sulfate transporter, putative, expressed	The expression level of H21 was between T44 and T45
MSTRG.12181	LOC_Os02g47510	9-cis-epoxycarotenoid dioxygenase 1, chloroplast precursor, putative, expressed	The expression level of H21 was between T44 and T45
LOC_Os11g16924	LOC_Os11g16924	expressed protein	The expression level of H21 was between T44 and T45
MSTRG.1987			The expression level of H21 was between T44 and T45

MSTRG.3909	LOC_Os01g72410	Leucine Rich Repeat family protein, expressed	The expression level of H21 was between T44 and T45
MSTRG.7218	LOC_Os11g39850	expressed protein	The expression level of H21 was between T44 and T45
MSTRG.23120			The expression level of H21 was between T44 and T45
MSTRG.7988	LOC_Os12g07400		The expression level of H21 was between T44 and T45
MSTRG.25594	LOC_Os07g28644	expressed protein	The expression level of H21 was between T44 and T45
MSTRG.15043	LOC_Os03g38350	expressed protein	The expression level of H21 was between T44 and T45
MSTRG.5281	LOC_Os10g37180	glycine cleavage system H protein, putative, expressed	The expression level of H21 was between T44 and T45
MSTRG.26586	LOC_Os07g46570	glutaredoxin, putative, expressed	The expression level of H21 was between T44 and T45
MSTRG.6385	LOC_Os11g13490	expressed protein	The expression level of H21 was between T44 and T45
MSTRG.26319	LOC_Os07g42370	zinc-finger protein, putative, expressed	The expression level of H21 was between T44 and T45
LOC_Os11g11940	LOC_Os11g11940	MLA10, putative, expressed	The expression level of H21 was between T44 and T45
MSTRG.5624	LOC_Os10g42550	Inositol 1, 3, 4-trisphosphate 5/6-kinase, putative, expressed	The expression level of H21 was between T44 and T45
MSTRG.15473			The expression level of H21 was between T44 and T45
MSTRG.29898	LOC_Os09g27734	expressed protein	The expression level of H21 was between T44 and T45
MSTRG.16256	LOC_Os03g59190	retrotransposon protein, putative, unclassified, expressed	The expression level of H21 was between T44 and T45
MSTRG.4039	LOC_Os01g74340	RNA recognition motif containing protein, putative, expressed	The expression level of H21 was between T44 and T45
MSTRG.1946	LOC_Os01g41834		The expression level of H21 was between T44 and T45
MSTRG.4893	LOC_Os10g29880		The expression level of H21 was between T44 and T45
MSTRG.18292	LOC_Os04g42670	OsFBL19 - F-box domain and LRR containing protein, expressed	The expression level of H21 was between T44 and T45
MSTRG.14498	LOC_Os03g22259	expressed protein	The expression level of H21 was between T44 and T45
MSTRG.28832	LOC_Os08g43410	LRP1, putative, expressed	The expression level of H21 was between T44 and T45
MSTRG.23730			The expression level of H21 was between T44 and T45
MSTRG.975	LOC_Os01g15830	peroxidase precursor, putative, expressed	The expression level of H21 was between T44 and T45
MSTRG.7031	LOC_Os11g37280	LTPL68 - Protease inhibitor/seed storage/LTP family protein precursor, expressed	The expression level of H21 was between T44 and T45
MSTRG.23167	LOC_Os06g25605	expressed protein	The expression level of H21 was between T44 and T45
MSTRG.25915	LOC_Os07g35970; LOC_Os07g35985; LOC_Os07g35990	SMP-30/Gluconolaconase/LRE-like region containing protein, expressed;strictosidine synthase 1 precursor, putative, expressed	The expression level of H21 was between T44 and T45
MSTRG.20023			The expression level of H21 was between T44 and T45
MSTRG.19110	LOC_Os04g55450	transposon protein, putative, unclassified, expressed	The expression level of H21 was between T44 and T45
MSTRG.18879	LOC_Os04g52380	methyl-CpG binding domain containing protein, putative, expressed	The expression level of H21 was between T44 and T45
MSTRG.9077	LOC_Os12g36400; LOC_Os12g36410		The expression level of H21 was between T44 and T45
MSTRG.22733	LOC_Os06g14220	STF-1, putative, expressed	The expression level of H21 was between T44 and T45
MSTRG.2869	LOC_Os01g56420	ctr copper transporter family protein, putative, expressed	The expression level of H21 was between T44 and T45
MSTRG.1229	LOC_Os01g21630	pectinacetylesterase domain containing protein, expressed	The expression level of H21 was between T44 and T45
MSTRG.13629			The expression level of H21 was between T44 and T45
MSTRG.25816	LOC_Os07g33580; LOC_Os07g33610	cytochrome P450, putative, expressed	The expression level of H21 was between T44 and T45
MSTRG.13667	LOC_Os03g10550		The expression level of H21 was between T44 and T45
MSTRG.20407	LOC_Os05g25860	expressed protein	The expression level of H21 was between T44 and T45
MSTRG.14516	LOC_Os03g22480	transposon protein, putative, unclassified, expressed	The expression level of H21 was between T44 and T45
MSTRG.9680	LOC_Os02g02340	glycerol-3-phosphate acyltransferase, putative, expressed	The expression level of H21 was between T44 and T45
MSTRG.11287	LOC_Os02g32900	expressed protein	The expression level of H21 was between T44 and T45
MSTRG.873	LOC_Os01g14030	DUF260 domain containing protein, putative, expressed	The expression level of H21 was between T44 and T45
MSTRG.28527	LOC_Os08g38920	caffeoyl-CoA O-methyltransferase, putative, expressed	The expression level of H21 was between T44 and T45
MSTRG.4193	LOC_Os10g05190	transposon protein, putative, CACTA, En/Spm sub-class, expressed	The expression level of H21 was between T44 and T45
MSTRG.27628	LOC_Os08g17150		The expression level of H21 was between T44 and T45
MSTRG.22513			The expression level of H21 was between T44 and T45
MSTRG.11176			The expression level of H21 was between T44 and T45
MSTRG.8204			The expression level of H21 was between T44 and T45
MSTRG.22628	LOC_Os06g12455	expressed protein	The expression level of H21 was between T44 and T45
MSTRG.15967	LOC_Os03g55420	peroxidase precursor, putative, expressed	The expression level of H21 was between T44 and T45
MSTRG.10014	LOC_Os02g06190		The expression level of H21 was between T44 and T45
MSTRG.11293	LOC_Os02g32980		The expression level of H21 was between T44 and T45
MSTRG.29473	LOC_Os09g17100		The expression level of H21 was between T44 and T45
MSTRG.14608	LOC_Os03g24200	OsFBX85 - F-box domain containing protein, expressed	The expression level of H21 was between T44 and T45
MSTRG.9790	LOC_Os02g03630	transposon protein, putative, CACTA, En/Spm sub-class, expressed	The expression level of H21 was between T44 and T45
MSTRG.25403	LOC_Os07g22680	SKP1-like protein 1B, putative, expressed	The expression level of H21 was between T44 and T45
MSTRG.29891	LOC_Os09g27620	PHD-finger domain containing protein, putative, expressed	The expression level of H21 was between T44 and T45
MSTRG.6216	LOC_Os11g10130	MYB family transcription factor, putative, expressed	The expression level of H21 was between T44 and T45

MSTRG.19723	LOC_Os05g05950	TOC159, putative, expressed	The expression level of H21 was between T44 and T45
MSTRG.20902	LOC_Os05g35290	phenylalanine ammonia-lyase, putative, expressed	The expression level of H21 was between T44 and T45
MSTRG.25731			The expression level of H21 was between T44 and T45
MSTRG.13341			The expression level of H21 was between T44 and T45
MSTRG.22261	LOC_Os06g06470; LOC_Os06g06490	U-box domain containing heat shock protein, putative, expressed	The expression level of H21 was between T44 and T45
MSTRG.11359	LOC_Os02g34410	U-box domain-containing protein, putative, expressed	The expression level of H21 was between T44 and T45
MSTRG.20994	LOC_Os05g37470	transmembrane amino acid transporter protein, putative, expressed	The expression level of H21 was between T44 and T45
MSTRG.16881	LOC_Os04g10434		The expression level of H21 was between T44 and T45
MSTRG.28959	LOC_Os08g45060	protein Kinase-like protein TMKL1 precursor, putative, expressed	The expression level of H21 was between T44 and T45
MSTRG.1352	LOC_Os01g25360	esterase, putative, expressed	The expression level of H21 was between T44 and T45
MSTRG.8624	LOC_Os12g25350	expressed protein	The expression level of H21 was between T44 and T45
MSTRG.25969			The expression level of H21 was between T44 and T45
MSTRG.24377	LOC_Os06g50040	OsSAUR29 - Auxin-responsive SAUR gene family member, expressed	The expression level of H21 was between T44 and T45
MSTRG.27730	LOC_Os08g20730	peroxidase precursor, putative, expressed	The expression level of H21 was between T44 and T45
MSTRG.29558	LOC_Os09g20024	expressed protein	The expression level of H21 was between T44 and T45
MSTRG.15470	LOC_Os03g47460	ribosome inactivating protein, putative, expressed	The expression level of H21 was between T44 and T45
MSTRG.30179	LOC_Os09g32740	ubiquitin carboxyl-terminal hydrolase family protein, expressed	The expression level of H21 was between T44 and T45
MSTRG.15227	LOC_Os03g42320	Sec1 family transport protein, putative, expressed	The expression level of H21 was between T44 and T45
MSTRG.10020	LOC_Os02g06180	phytosulfokine receptor precursor, putative, expressed	The expression level of H21 was between T44 and T45
MSTRG.20296	LOC_Os05g23130	expressed protein	The expression level of H21 was between T44 and T45
MSTRG.14187	LOC_Os03g17690		The expression level of H21 was between T44 and T45
MSTRG.7290	LOC_Os11g41210	disease resistance protein RPM1, putative, expressed	The expression level of H21 was between T44 and T45
MSTRG.24542	LOC_Os07g01910	expressed protein	The expression level of H21 was between T44 and T45
MSTRG.2319	LOC_Os01g48339	expressed protein	The expression level of H21 was between T44 and T45
MSTRG.20457	LOC_Os05g28090	expressed protein	The expression level of H21 was between T44 and T45
MSTRG.2935	LOC_Os01g57310	rp1, putative, expressed	The expression level of H21 was between T44 and T45
MSTRG.27149			The expression level of H21 was between T44 and T45
MSTRG.14761	LOC_Os03g27830		The expression level of H21 was between T44 and T45
MSTRG.17663			The expression level of H21 was between T44 and T45
MSTRG.7714	LOC_Os12g02814	retrotransposon protein, putative, unclassified, expressed	The expression level of H21 was between T44 and T45
MSTRG.9017	LOC_Os12g35590	expressed protein	The expression level of H21 was between T44 and T45
MSTRG.2314	LOC_Os01g48320	transcription factor like protein, putative, expressed	The expression level of H21 was between T44 and T45
MSTRG.18258			The expression level of H21 was between T44 and T45
MSTRG.7463	LOC_Os11g44870	expressed protein	The expression level of H21 was between T44 and T45
MSTRG.15181	LOC_Os03g41060; LOC_Os03g41050	expressed protein	The expression level of H21 was between T44 and T45
MSTRG.24717	LOC_Os07g05365	photosystem II 10 kDa polypeptide, chloroplast precursor, putative, expressed	The expression level of H21 was between T44 and T45
MSTRG.10307			The expression level of H21 was between T44 and T45
MSTRG.13680	LOC_Os03g10740		The expression level of H21 was between T44 and T45
MSTRG.22692	LOC_Os06g13330	expressed protein	The expression level of H21 was between T44 and T45
MSTRG.1086	LOC_Os01g18050	tubulin/FtsZ domain containing protein, putative, expressed	The expression level of H21 was between T44 and T45
MSTRG.12707	LOC_Os02g55270		The expression level of H21 was between T44 and T45
MSTRG.27462	LOC_Os08g13310		The expression level of H21 was between T44 and T45
MSTRG.29309			The expression level of H21 was between T44 and T45
MSTRG.26227	LOC_Os07g40620	saccharopine dehydrogenase, putative, expressed	The expression level of H21 was between T44 and T45
MSTRG.3292	LOC_Os01g62630	aspartic proteinase nepenthesin precursor, putative, expressed	The expression level of H21 was between T44 and T45
MSTRG.27921	LOC_Os08g27490	expressed protein	The expression level of H21 was between T44 and T45
MSTRG.17158			The expression level of H21 was between T44 and T45
MSTRG.2285	LOC_Os01g47760	OsGrx_I1 - glutaredoxin subgroup III, expressed	The expression level of H21 was between T44 and T45
MSTRG.8283	LOC_Os12g14224	expressed protein	The expression level of H21 was between T44 and T45
MSTRG.2442	LOC_Os01g50200	UDP-glucoronosyl and UDP-glucosyl transferase domain containing protein, expressed	The expression level of H21 was between T44 and T45
MSTRG.16730	LOC_Os04g06734	expressed protein	The expression level of H21 was between T44 and T45
MSTRG.24012	LOC_Os06g44280	retrotransposon protein, putative, Ty3-gypsy subclass, expressed	The expression level of H21 was between T44 and T45
MSTRG.662	LOC_Os01g10580	B-box zinc finger family protein, putative, expressed	The expression level of H21 was between T44 and T45
MSTRG.22877	LOC_Os06g17900	disease resistance protein RPM1, putative, expressed	The expression level of H21 was between T44 and T45
MSTRG.28132	LOC_Os08g31760	expressed protein	The expression level of H21 was between T44 and T45
MSTRG.10279	LOC_Os02g10350	MLO domain containing protein, putative, expressed	The expression level of H21 was between T44 and T45
MSTRG.1683	LOC_Os01g36250	transposon protein, putative, unclassified, expressed	The expression level of H21 was between T44 and T45
MSTRG.26511	LOC_Os07g45490		The expression level of H21 was between T44 and T45

MSTRG.14961	LOC_Os03g35788		The expression level of H21 was between T44 and T45
MSTRG.22327	LOC_Os06g07470	expressed protein	The expression level of H21 was between T44 and T45
MSTRG.18020			The expression level of H21 was between T44 and T45
MSTRG.7948	LOC_Os12g06780	expressed protein	The expression level of H21 was between T44 and T45
MSTRG.17262	LOC_Os04g22950		The expression level of H21 was between T44 and T45
MSTRG.24263	LOC_Os06g48180	glycosyl hydrolases family 16, putative, expressed	The expression level of H21 was between T44 and T45
MSTRG.17541	LOC_Os04g30890	MYB family transcription factor, putative, expressed	The expression level of H21 was between T44 and T45
MSTRG.21625	LOC_Os05g48030	anti-silencing protein, ASF1-like domain containing protein, expressed	The expression level of H21 was between T44 and T45
MSTRG.7965	LOC_Os12g06910		The expression level of H21 was between T44 and T45
MSTRG.1558	LOC_Os01g32864	retrotransposon protein, putative, unclassified, expressed	The expression level of H21 was between T44 and T45
MSTRG.29619			The expression level of H21 was between T44 and T45
MSTRG.25162	LOC_Os07g12820	B3 DNA binding domain containing protein	The expression level of H21 was between T44 and T45
MSTRG.5545	LOC_Os10g41390	protein kinase domain containing protein, expressed	The expression level of H21 was between T44 and T45
MSTRG.18335	LOC_Os04g43310	expressed protein	The expression level of H21 was between T44 and T45
MSTRG.18321	LOC_Os04g43110	hypothetical protein	The expression level of H21 was between T44 and T45
MSTRG.7033	LOC_Os11g37300	OsFBDUF53 - F-box and DUF domain containing protein, expressed	The expression level of H21 was between T44 and T45
MSTRG.22770	LOC_Os06g15180	transposon protein, putative, CACTA, En/Spm sub-class	The expression level of H21 was between T44 and T45
MSTRG.2090	LOC_Os01g43774	cytochrome P450 72A1, putative, expressed	The expression level of H21 was between T44 and T45
MSTRG.13783	LOC_Os03g11860	expressed protein	The expression level of H21 was between T44 and T45
MSTRG.28273	LOC_Os08g34120	expressed protein	The expression level of H21 was between T44 and T45
MSTRG.28415	LOC_Os08g37040	gibberellin receptor GID1L2, putative, expressed	The expression level of H21 was between T44 and T45
MSTRG.25774	LOC_Os07g32850	hypothetical protein	The expression level of H21 was between T44 and T45
MSTRG.19270	LOC_Os04g57760	expressed protein	The expression level of H21 was between T44 and T45
MSTRG.10569	LOC_Os02g15560	transposon protein, putative, Mutator sub-class, expressed	The expression level of H21 was between T44 and T45
MSTRG.20967	LOC_Os05g36360	U-box domain containing protein, expressed	The expression level of H21 was between T44 and T45
MSTRG.12625	LOC_Os02g54140	hsp20/alpha crystallin family protein, putative, expressed	The expression level of H21 was between T44 and T45
MSTRG.27886	LOC_Os08g25690	expressed protein	The expression level of H21 was between T44 and T45
MSTRG.20916	LOC_Os05g35444	expressed protein	The expression level of H21 was between T44 and T45
MSTRG.5420	LOC_Os10g39680	CHIT14 - Chitinase family protein precursor, expressed	The expression level of H21 was between T44 and T45
MSTRG.26912	LOC_Os08g02700	fructose-bisphospate aldolase isozyme, putative, expressed	The expression level of H21 was between T44 and T45
MSTRG.15816	LOC_Os03g52680	expressed protein	The expression level of H21 was between T44 and T45
MSTRG.29131	LOC_Os09g04990	cytoskeletal protein, putative, expressed	The expression level of H21 was between T44 and T45
MSTRG.28485	LOC_Os08g38040	transposon protein, putative, unclassified, expressed	The expression level of H21 was between T44 and T45
MSTRG.14641	LOC_Os03g25080	OsFBX86 - F-box domain containing protein, expressed	The expression level of H21 was between T44 and T45
LOC_Os01g17300	LOC_Os01g17300	expressed protein	The expression level of H21 was between T44 and T45
MSTRG.19593	LOC_Os05g03620	TKL_IRAK_CR4L.4 - The CR4L subfamily has homology with Crinkly4, expressed	The expression level of H21 was between T44 and T45
MSTRG.28411	LOC_Os08g36860	cytochrome P450, putative, expressed	The expression level of H21 was between T44 and T45
MSTRG.6466	LOC_Os11g15700		The expression level of H21 was between T44 and T45
MSTRG.2963	LOC_Os01g57599	retrotransposon protein, putative, unclassified, expressed	The expression level of H21 was between T44 and T45
MSTRG.6620			The expression level of H21 was between T44 and T45
MSTRG.2332	LOC_Os01g48570	expressed protein	The expression level of H21 was between T44 and T45
MSTRG.15148			The expression level of H21 was between T44 and T45
MSTRG.12170	LOC_Os02g47370	transcription factor TF2, putative, expressed	The expression level of H21 was between T44 and T45
MSTRG.343	LOC_Os01g05540	expressed protein	The expression level of H21 was between T44 and T45
MSTRG.17949	LOC_Os04g37740	zinc finger, C3HC4 type domain containing protein, expressed	The expression level of H21 was between T44 and T45
MSTRG.12501	LOC_Os02g52300	CPuORF38 - conserved peptide uORF-containing transcript, expressed	The expression level of H21 was between T44 and T45
MSTRG.2931	LOC_Os01g57250	expressed protein	The expression level of H21 was between T44 and T45
MSTRG.17783	LOC_Os04g34700		The expression level of H21 was between T44 and T45
MSTRG.230	LOC_Os01g03310	BBTI1 - Bowman-Birk type bran trypsin inhibitor precursor, expressed	The expression level of H21 was between T44 and T45
MSTRG.19474	LOC_Os05g01810		The expression level of H21 was between T44 and T45
MSTRG.10886			The expression level of H21 was between T44 and T45
MSTRG.10013	LOC_Os02g06120		The expression level of H21 was between T44 and T45
MSTRG.13221	LOC_Os03g04120	AMP-binding enzyme, putative, expressed	The expression level of H21 was between T44 and T45
MSTRG.14888	LOC_Os03g31320	RING zinc finger protein-like, putative, expressed	The expression level of H21 was between T44 and T45
MSTRG.14562			The expression level of H21 was between T44 and T45
MSTRG.14760	LOC_Os03g27830	expressed protein	The expression level of H21 was between T44 and T45
MSTRG.20691	LOC_Os05g31890	aspartyl protease family protein, putative, expressed	The expression level of H21 was between T44 and T45
MSTRG.24885	LOC_Os07g08090		The expression level of H21 was between T44 and T45

MSTRG.24207	LOC_Os06g47430	expressed protein	The expression level of H21 was between T44 and T45
MSTRG.22793	LOC_Os06g15740	expressed protein	The expression level of H21 was between T44 and T45
MSTRG.7239	LOC_Os11g40360	transposon protein, putative, unclassified, expressed	The expression level of H21 was between T44 and T45
MSTRG.3658	LOC_Os01g68300	expressed protein	The expression level of H21 was between T44 and T45
MSTRG.7130	LOC_Os11g38720	retrotransposon protein, putative, Ty3-gypsy subclass	The expression level of H21 was between T44 and T45
MSTRG.11415	LOC_Os02g35090	expressed protein	The expression level of H21 was between T44 and T45
MSTRG.8740	LOC_Os12g29605	expressed protein	The expression level of H21 was between T44 and T45
MSTRG.14595	LOC_Os03g24260		The expression level of H21 was between T44 and T45
MSTRG.24599	LOC_Os07g03025	expressed protein	The expression level of H21 was between T44 and T45
MSTRG.10472	LOC_Os02g13160	F-box protein PP2-A15, putative, expressed	The expression level of H21 was between T44 and T45
MSTRG.29851	LOC_Os09g26820	exo70 exocyst complex subunit, putative, expressed	The expression level of H21 was between T44 and T45
MSTRG.329	LOC_Os01g05310	expressed protein	The expression level of H21 was between T44 and T45
MSTRG.8476	LOC_Os12g20380	expressed protein	The expression level of H21 was between T44 and T45
MSTRG.22476	LOC_Os06g09679		The expression level of H21 was between T44 and T45
MSTRG.28656	LOC_Os08g40890	spermidine synthase-related, putative, expressed	The expression level of H21 was between T44 and T45
MSTRG.9798	LOC_Os02g03700	expressed protein	The expression level of H21 was between T44 and T45
MSTRG.622	LOC_Os01g10030;LOC_Os01g10040	expressed protein;cytochrome P450, putative, expressed	The expression level of H21 was between T44 and T45
LOC_Os06g49380	LOC_Os06g49380	NBS-LRR disease resistance protein, putative, expressed	The expression level of H21 was between T44 and T45
MSTRG.8281	LOC_Os12g14220	expressed protein	The expression level of H21 was between T44 and T45
MSTRG.7274	LOC_Os11g40840	receptor-like protein kinase 2 precursor, putative, expressed	The expression level of H21 was between T44 and T45
MSTRG.25108	LOC_Os07g11770	expressed protein	The expression level of H21 was between T44 and T45
MSTRG.22043	LOC_Os06g03820	expressed protein	The expression level of H21 was between T44 and T45
MSTRG.11614	LOC_Os02g38440	oxidoreductase, short chain dehydrogenase/reductase family, putative, expressed	The expression level of H21 was between T44 and T45
MSTRG.25878	LOC_Os07g35050;LOC_Os07g35060	OsFBX237 - F-box domain containing protein, expressed;OsFBX238 - F-box domain containing protein, expressed	The expression level of H21 was between T44 and T45
MSTRG.7047	LOC_Os11g37400		The expression level of H21 was between T44 and T45
MSTRG.27514	LOC_Os08g14195	expressed protein	The expression level of H21 was between T44 and T45
MSTRG.5505	LOC_Os10g40920		The expression level of H21 was between T44 and T45
MSTRG.30086	LOC_Os09g31080	induced stolen tip protein TUB8, putative, expressed	The expression level of H21 was between T44 and T45
MSTRG.3350	LOC_Os01g63710	CDC6 - Putative DNA replication initiation protein, expressed	The expression level of H21 was between T44 and T45
MSTRG.5582	LOC_Os10g41810		The expression level of H21 was between T44 and T45
MSTRG.18481			The expression level of H21 was between T44 and T45
MSTRG.13846	LOC_Os03g12730	receptor protein kinase CLAVATA1 precursor, putative, expressed	The expression level of H21 was between T44 and T45
MSTRG.6164	LOC_Os11g09360		The expression level of H21 was between T44 and T45
MSTRG.15977	LOC_Os03g55550	helix-loop-helix DNA-binding domain containing protein, expressed	The expression level of H21 was between T44 and T45
MSTRG.26139	LOC_Os07g39090	pentatricopeptide repeat domain containing protein, putative, expressed	The expression level of H21 was between T44 and T45
MSTRG.7590	LOC_Os11g48050		The expression level of H21 was between T44 and T45
MSTRG.7118	LOC_Os11g38590	expressed protein	The expression level of H21 was between T44 and T45
MSTRG.11957	LOC_Os02g44130	ZOS2-14 - C2H2 zinc finger protein, expressed	The expression level of H21 was between T44 and T45
MSTRG.26243	LOC_Os07g40830		The expression level of H21 was between T44 and T45
MSTRG.24407	LOC_Os06g50350	expressed protein	The expression level of H21 was between T44 and T45
MSTRG.5216	LOC_Os10g35820	expressed protein	The expression level of H21 was between T44 and T45
MSTRG.24578	LOC_Os07g02660	expressed protein	The expression level of H21 was between T44 and T45
MSTRG.4024	LOC_Os01g74140	WRKY17, expressed	The expression level of H21 was between T44 and T45
MSTRG.22354	LOC_Os06g07878	peptidase, T1 family, putative, expressed	The expression level of H21 was between T44 and T45
MSTRG.8235	LOC_Os12g13280		The expression level of H21 was between T44 and T45
MSTRG.22272	LOC_Os06g06660	expressed protein	The expression level of H21 was between T44 and T45
LOC_Os12g01090	LOC_Os12g01090	retrotransposon protein, putative, unclassified, expressed	The expression level of H21 was between T44 and T45
MSTRG.23501	LOC_Os06g36650	ABC transporter family protein, putative, expressed	The expression level of H21 was between T44 and T45
MSTRG.18795	LOC_Os04g51350	pentatricopeptide, putative, expressed	The expression level of H21 was between T44 and T45
MSTRG.24625	LOC_Os07g03260	CSLC10 - cellulose synthase-like family C, expressed	The expression level of H21 was between T44 and T45
MSTRG.10002	LOC_Os02g05980	phytosulfokine receptor precursor, putative, expressed	The expression level of H21 was between T44 and T45
MSTRG.24992	LOC_Os07g09710	OsFBX220 - F-box domain containing protein, expressed	The expression level of H21 was between T44 and T45
MSTRG.7117	LOC_Os11g38580	NBS-LRR type disease resistance protein, putative, expressed	The expression level of H21 was between T44 and T45
MSTRG.14960	LOC_Os03g35560	retrotransposon protein, putative, Ty1-copia subclass, expressed	The expression level of H21 was between T44 and T45
MSTRG.20959	LOC_Os05g36240	expressed protein	The expression level of H21 was between T44 and T45
MSTRG.25948	LOC_Os07g36330		The expression level of H21 was between T44 and T45
MSTRG.24598	LOC_Os07g03015	expressed protein	The expression level of H21 was between T44 and T45
MSTRG.26994	LOC_Os08g04240	cysteine-rich repeat secretory protein 55 precursor, putative, expressed	The expression level of H21 was between T44 and T45
MSTRG.29879	LOC_Os09g27360	expressed protein	The expression level of H21 was between T44 and T45

MSTRG.28098	LOC_Os08g31110		The expression level of H21 was between T44 and T45
MSTRG.835	LOC_Os01g13430	importin-alpha re-exporter, putative, expressed	The expression level of H21 was between T44 and T45
LOC_Os11g01090	LOC_Os11g01090	retrotransposon protein, putative, unclassified, expressed	The expression level of H21 was between T44 and T45
MSTRG.7600	LOC_Os12g01160; LOC_Os12g01150	dehydrogenase, putative, expressed	The expression level of H21 was between T44 and T45
MSTRG.7291	LOC_Os11g41230	ATBPM6, putative, expressed	The expression level of H21 was between T44 and T45
MSTRG.28044	LOC_Os08g30020	membrane protein, putative, expressed	The expression level of H21 was between T44 and T45
MSTRG.12414	LOC_Os02g50930	RING-H2 finger protein, putative, expressed	The expression level of H21 was between T44 and T45
MSTRG.10866	LOC_Os02g22100	OsRhmbd6 - Putative Rhomboid homologue, expressed	The expression level of H21 was between T44 and T45
MSTRG.22247	LOC_Os06g06330	expressed protein	The expression level of H21 was between T44 and T45
MSTRG.1635	LOC_Os01g34790	expressed protein	The expression level of H21 was between T44 and T45
MSTRG.12284	LOC_Os02g49060	amino acid transporter, putative, expressed	The expression level of H21 was between T44 and T45
MSTRG.25857	LOC_Os07g34520	isocitrate lyase, putative, expressed	The expression level of H21 was between T44 and T45
MSTRG.13701	LOC_Os03g10950	protein phosphatase 2C, putative, expressed	The expression level of H21 was between T44 and T45
MSTRG.4368	LOC_Os10g10460	retrotransposon protein, putative, Ty3-gypsy subclass, expressed	The expression level of H21 was between T44 and T45
MSTRG.4982	LOC_Os10g32444		The expression level of H21 was between T44 and T45
MSTRG.21905	LOC_Os06g01210	plastocyanin, chloroplast precursor, putative, expressed	The expression level of H21 was between T44 and T45
MSTRG.16141			The expression level of H21 was between T44 and T45
MSTRG.24175	LOC_Os06g46770		The expression level of H21 was between T44 and T45
MSTRG.23259	LOC_Os06g28990	transposon protein, putative, Mutator sub-class, expressed	The expression level of H21 was between T44 and T45
MSTRG.24365	LOC_Os06g49780	expressed protein	The expression level of H21 was between T44 and T45
MSTRG.2936	LOC_Os01g57310		The expression level of H21 was between T44 and T45
MSTRG.27339	LOC_Os08g11190		The expression level of H21 was between T44 and T45
MSTRG.8156	LOC_Os12g11370	verticillium wilt disease resistance protein, putative, expressed	The expression level of H21 was between T44 and T45
MSTRG.6976			The expression level of H21 was between T44 and T45
MSTRG.7243	LOC_Os11g40390	expressed protein	The expression level of H21 was between T44 and T45
MSTRG.18336			The expression level of H21 was between T44 and T45
MSTRG.24936	LOC_Os07g08660	40S ribosomal protein S15, putative, expressed	The expression level of H21 was between T44 and T45
MSTRG.18505	LOC_Os04g46170	ubiquitin carboxyl-terminal hydrolase, family 1, putative, expressed	The expression level of H21 was between T44 and T45
MSTRG.24990			The expression level of H21 was between T44 and T45
LOC_Os11g12810	LOC_Os11g12810	sucrose-phosphate synthase, putative, expressed	The expression level of H21 was between T44 and T45
MSTRG.11539	LOC_Os02g37350		The expression level of H21 was between T44 and T45
MSTRG.5097	LOC_Os10g34360	stilbene synthase, putative, expressed	The expression level of H21 was between T44 and T45
MSTRG.4068	LOC_Os10g01290	PHLOEM 2-LIKE A10, putative, expressed	The expression level of H21 was between T44 and T45
LOC_Os10g41930	LOC_Os10g41930	haloacid dehalogenase-like hydrolase family protein, putative, expressed	The expression level of H21 was between T44 and T45
LOC_Os12g24420	LOC_Os12g24420	cyclin-dependent kinase G-1, putative, expressed	The expression level of H21 was between T44 and T45
MSTRG.4531	LOC_Os10g20080; LOC_Os10g19970	expressed protein	The expression level of H21 was between T44 and T45
MSTRG.24597			The expression level of H21 was between T44 and T45
MSTRG.4198	LOC_Os10g05240	OsFBX369 - F-box domain containing protein, expressed	The expression level of H21 was between T44 and T45
MSTRG.13370	LOC_Os03g06390	expressed protein	The expression level of H21 was between T44 and T45
MSTRG.21389	LOC_Os05g44060	expressed protein	The expression level of H21 was between T44 and T45
LOC_Os01g15990	LOC_Os01g15990	expressed protein	The expression level of H21 was between T44 and T45
MSTRG.30425	LOC_Os09g37300	transporter, monovalent cation:proton antiporter-2 family, putative, expressed	The expression level of H21 was between T44 and T45
MSTRG.9640	LOC_Os02g01750	expressed protein	The expression level of H21 was between T44 and T45
MSTRG.24391	LOC_Os06g50090		The expression level of H21 was between T44 and T45
MSTRG.25777	LOC_Os07g32960; LOC_Os07g32990	expressed protein	The expression level of H21 was between T44 and T45
MSTRG.6311	LOC_Os11g11960		The expression level of H21 was between T44 and T45
LOC_Os05g15220	LOC_Os05g15220	retrotransposon protein, putative, unclassified, expressed	The expression level of H21 was between T44 and T45
MSTRG.7217	LOC_Os11g39820		The expression level of H21 was between T44 and T45
MSTRG.12129	LOC_Os02g46810		The expression level of H21 was between T44 and T45
MSTRG.6988			The expression level of H21 was between T44 and T45
MSTRG.7502	LOC_Os11g45864	transposon protein, putative, CACTA, En/Spm sub-class, expressed	The expression level of H21 was between T44 and T45
MSTRG.8451	LOC_Os12g19470	ribulose bisphosphate carboxylase small chain, chloroplast precursor, putative, expressed	The expression level of H21 was between T44 and T45
MSTRG.8745	LOC_Os12g29670	expressed protein	The expression level of H21 was between T44 and T45
MSTRG.12925	LOC_Os02g57930	retrotransposon protein, putative, Ty3-gypsy subclass, expressed	The expression level of H21 was between T44 and T45
MSTRG.2473	LOC_Os01g50750	zinc finger, C3HC4 type domain containing protein, expressed	The expression level of H21 was between T44 and T45
MSTRG.3263	LOC_Os01g62160	trp repressor/replication initiator, putative, expressed	The expression level of H21 was between T44 and T45
MSTRG.21097			The expression level of H21 was between T44 and T45
MSTRG.24219	LOC_Os06g47544	expressed protein	The expression level of H21 was between T44 and T45

MSTRG.22538	LOC_Os06g10830	retrotransposon protein, putative, unclassified, expressed	The expression level of H21 was between T44 and T45
MSTRG.21078	LOC_Os05g38810		The expression level of H21 was between T44 and T45
MSTRG.26876	LOC_Os08g02210	expressed protein	The expression level of H21 was between T44 and T45
MSTRG.8567	LOC_Os12g23540		The expression level of H21 was between T44 and T45
MSTRG.16738	LOC_Os04g07280	AGAP002737-PA, putative, expressed	The expression level of H21 was between T44 and T45
LOC_Os09g06634	LOC_Os09g06634	transposon protein, putative, Pong sub-class, expressed	The expression level of H21 was between T44 and T45
MSTRG.7233	LOC_Os11g40210	remorin C-terminal domain containing protein, putative, expressed	The expression level of H21 was between T44 and T45
MSTRG.26806	LOC_Os08g01100	HMG1/2, putative, expressed	The expression level of H21 was between T44 and T45
MSTRG.28645	LOC_Os08g40640	retrotransposon protein, putative, Ty1-copia subclass, expressed	The expression level of H21 was between T44 and T45
MSTRG.20342			The expression level of H21 was between T44 and T45
MSTRG.18628	LOC_Os04g48200	cytochrome P450, putative, expressed	The expression level of H21 was between T44 and T45
MSTRG.1912	LOC_Os01g41280	OsFBD4 - F-box and FBD domain containing protein, expressed	The expression level of H21 was between T44 and T45
MSTRG.2956	LOC_Os01g57560	serine/threonine-protein kinase receptor precursor, putative, expressed	The expression level of H21 was between T44 and T45
MSTRG.21235	LOC_Os05g41490	circadian clock coupling factor ZGT, putative, expressed	The expression level of H21 was between T44 and T45
MSTRG.14789	LOC_Os03g28389	expressed protein	The expression level of H21 was between T44 and T45
MSTRG.30397	LOC_Os09g36820	MCM10 - Putative minichromosome maintenance MCM protein 10, expressed	The expression level of H21 was between T44 and T45
MSTRG.17167	LOC_Os04g19114	retrotransposon protein, putative, Ty3-gypsy subclass, expressed	The expression level of H21 was between T44 and T45
MSTRG.15565	LOC_Os03g48610	galactosyltransferase family protein, putative, expressed	The expression level of H21 was between T44 and T45
MSTRG.2975	LOC_Os01g57770	OsPOP4 - Putative Prolyl Oligopeptidase homologue, expressed	The expression level of H21 was between T44 and T45
MSTRG.13428	LOC_Os03g07234	pentatricopeptide, putative, expressed	The expression level of H21 was between T44 and T45
MSTRG.25862	LOC_Os07g34620	expressed protein	The expression level of H21 was between T44 and T45
MSTRG.17765	LOC_Os04g34420	serine/threonine-protein kinase receptor precursor, putative, expressed	The expression level of H21 was between T44 and T45
MSTRG.16349	LOC_Os03g60560	ZOS3-21 - C2H2 zinc finger protein, expressed	The expression level of H21 was between T44 and T45
MSTRG.601	LOC_Os01g09640	Myb transcription factor, putative, expressed	The expression level of H21 was between T44 and T45
MSTRG.11856	LOC_Os02g42585	AP2 domain containing protein, expressed	The expression level of H21 was between T44 and T45
MSTRG.7575	LOC_Os11g47900	SCARECROW, putative, expressed	The expression level of H21 was between T44 and T45
MSTRG.26837	LOC_Os08g01650	Rf1, mitochondrial precursor, putative, expressed	The expression level of H21 was between T44 and T45
MSTRG.25939	LOC_Os07g36250	peptide chain release factor protein, putative, expressed	The expression level of H21 was between T44 and T45
MSTRG.20363	LOC_Os05g24650	DUF567 domain containing protein, putative, expressed	The expression level of H21 was between T44 and T45
MSTRG.30407	LOC_Os09g36930	aquaporin protein, putative, expressed	The expression level of H21 was between T44 and T45
MSTRG.16970	LOC_Os04g14204	expressed protein	The expression level of H21 was between T44 and T45
MSTRG.8679	LOC_Os12g26510	expressed protein	The expression level of H21 was between T44 and T45
MSTRG.12111	LOC_Os02g46620	expressed protein	The expression level of H21 was between T44 and T45
MSTRG.23267	LOC_Os06g29260	retrotransposon protein, putative, unclassified, expressed	The expression level of H21 was between T44 and T45
MSTRG.30310	LOC_Os09g35700	LTPL45 - Protease inhibitor/seed storage/LTP family protein precursor, expressed	The expression level of H21 was between T44 and T45
MSTRG.18484			The expression level of H21 was between T44 and T45
MSTRG.28952	LOC_Os08g45000	inorganic phosphate transporter, putative, expressed	The expression level of H21 was between T44 and T45
MSTRG.7041	LOC_Os11g37440; LOC_Os11g37360	expressed protein	The expression level of H21 was between T44 and T45
MSTRG.21257	LOC_Os05g41870	glycine-rich cell wall protein, putative, expressed	The expression level of H21 was between T44 and T45
MSTRG.4504			The expression level of H21 was between T44 and T45
MSTRG.11547			The expression level of H21 was between T44 and T45
MSTRG.28833	LOC_Os08g43430	CXE carboxylesterase, putative, expressed	The expression level of H21 was between T44 and T45
MSTRG.22447			The expression level of H21 was between T44 and T45
MSTRG.12731	LOC_Os02g55610	serine-rich protein, putative, expressed	The expression level of H21 was between T44 and T45
MSTRG.28603	LOC_Os08g39990	expressed protein	The expression level of H21 was between T44 and T45
MSTRG.10480	LOC_Os02g13370	expressed protein	The expression level of H21 was between T44 and T45
MSTRG.23261	LOC_Os06g29110	MLO domain containing protein, putative, expressed	The expression level of H21 was between T44 and T45
MSTRG.15709	LOC_Os03g51100		The expression level of H21 was between T44 and T45
MSTRG.14560			The expression level of H21 was between T44 and T45
MSTRG.19370	LOC_Os04g59200	peroxidase precursor, putative, expressed	The expression level of H21 was between T44 and T45
LOC_Os04g09580	LOC_Os04g09580	expressed protein	The expression level of H21 was between T44 and T45
MSTRG.12168	LOC_Os02g47330		The expression level of H21 was between T44 and T45
MSTRG.20284	LOC_Os05g22790	expressed protein	The expression level of H21 was between T44 and T45
MSTRG.19392	LOC_Os04g59540	phosphatidylinositol-4-phosphate 5-Kinase, putative, expressed	The expression level of H21 was between T44 and T45
MSTRG.13945			The expression level of H21 was between T44 and T45

MSTRG.5756	LOC_Os11g02350	LTPL25 - Protease inhibitor/seed storage/LTP family protein precursor, expressed	The expression level of H21 was between T44 and T45
MSTRG.3050	LOC_Os01g58850	circadian clock coupling factor-related, putative, expressed	The expression level of H21 was between T44 and T45
MSTRG.28204	LOC_Os08g32960	endonuclease/exonuclease/phosphatase family domain containing protein, expressed	The expression level of H21 was between T44 and T45
MSTRG.23123	LOC_Os06g25010	glycosyl hydrolase, putative, expressed	The expression level of H21 was between T44 and T45
MSTRG.24754	LOC_Os07g05940	9-cis-epoxycarotenoid dioxygenase 1, chloroplast precursor, putative, expressed	The expression level of H21 was between T44 and T45
MSTRG.7646	LOC_Os12g01820		The expression level of H21 was between T44 and T45
MSTRG.1335	LOC_Os01g24710	jacalin-like lectin domain containing protein, expressed	The expression level of H21 was between T44 and T45
MSTRG.6397	LOC_Os11g13750	expressed protein	The expression level of H21 was between T44 and T45
MSTRG.6225	LOC_Os11g10220	retrotransposon protein, putative, Ty1-copia subclass	The expression level of H21 was between T44 and T45
MSTRG.2218	LOC_Os01g46570	CTP synthase, putative, expressed	The expression level of H21 was between T44 and T45
MSTRG.8922	LOC_Os12g34018	ATP synthase protein YMF19, putative, expressed	The expression level of H21 was between T44 and T45
MSTRG.3181	LOC_Os01g60830	expressed protein	The expression level of H21 was between T44 and T45
MSTRG.30510	LOC_Os09g38480	expressed protein	The expression level of H21 was between T44 and T45
MSTRG.7207	LOC_Os11g39680	expressed protein	The expression level of H21 was between T44 and T45
MSTRG.22059	LOC_Os06g04030	histone H3, putative, expressed	The expression level of H21 was between T44 and T45
MSTRG.26129			The expression level of H21 was between T44 and T45
MSTRG.29554	LOC_Os09g20000	heavy metal-associated domain containing protein, expressed	The expression level of H21 was between T44 and T45
MSTRG.24628	LOC_Os07g03810		The expression level of H21 was between T44 and T45
MSTRG.19212	LOC_Os04g57060		The expression level of H21 was between T44 and T45
MSTRG.24576	LOC_Os07g02630	expressed protein	The expression level of H21 was between T44 and T45
MSTRG.19862	LOC_Os05g07950	OsFBX160 - F-box domain containing protein, expressed	The expression level of H21 was between T44 and T45
MSTRG.16634	LOC_Os04g01520	expressed protein	The expression level of H21 was between T44 and T45
MSTRG.23132			The expression level of H21 was between T44 and T45
MSTRG.23053	LOC_Os06g22810	TKL_IRAK_CrRLK1L-1.14 - The CrRLK1L-1 subfamily has homology to the CrRLK1L homolog, expressed	The expression level of H21 was between T44 and T45
MSTRG.13974	LOC_Os03g14630	LTPL106 - Protease inhibitor/seed storage/LTP family protein precursor, expressed	The expression level of H21 was between T44 and T45
MSTRG.15927			The expression level of H21 was between T44 and T45
MSTRG.2098	LOC_Os01g43910	CGMC_MAPKCMGC_2.4 - CGMC includes CDA, MAPK, GSK3, and CLKC kinases, expressed	The expression level of H21 was between T44 and T45
MSTRG.11076	LOC_Os02g28074	XRN 5-3 exonuclease N-terminus family protein, expressed	The expression level of H21 was between T44 and T45
MSTRG.11354	LOC_Os02g34130	transposon protein, putative, CACTA, En/Spm sub-class, expressed	The expression level of H21 was between T44 and T45
MSTRG.11326	LOC_Os02g33705	expressed protein	The expression level of H21 was between T44 and T45
MSTRG.14338	LOC_Os03g19600	retrotransposon protein, putative, unclassified, expressed	The expression level of H21 was between T44 and T45
MSTRG.12289	LOC_Os02g49140	glycosyltransferase, putative, expressed	The expression level of H21 was between T44 and T45
MSTRG.10460	LOC_Os02g13020	expressed protein	The expression level of H21 was between T44 and T45
MSTRG.7088	LOC_Os11g38100	OsFBDUF55 - F-box and DUF domain containing protein, expressed	The expression level of H21 was between T44 and T45
MSTRG.6765	LOC_Os11g29950	retrotransposon protein, putative, unclassified, expressed	The expression level of H21 was between T44 and T45
MSTRG.7094	LOC_Os11g38150	expressed protein	The expression level of H21 was between T44 and T45
MSTRG.6320	LOC_Os11g12590		The expression level of H21 was between T44 and T45
MSTRG.19055	LOC_Os04g54620		The expression level of H21 was between T44 and T45
MSTRG.987	LOC_Os01g16020	FAD binding domain containing protein, expressed	The expression level of H21 was between T44 and T45
MSTRG.18721	LOC_Os04g49748	purine permease, putative, expressed	The expression level of H21 was between T44 and T45
MSTRG.26143	LOC_Os07g39250		The expression level of H21 was between T44 and T45
MSTRG.2771	LOC_Os01g55090		The expression level of H21 was between T44 and T45
MSTRG.29246	LOC_Os09g09540	transposon protein, putative, unclassified, expressed	The expression level of H21 was between T44 and T45
MSTRG.17584	LOC_Os04g31640	expressed protein	The expression level of H21 was between T44 and T45
MSTRG.29702	LOC_Os09g23730	HMG-Y-related protein A, putative, expressed	The expression level of H21 was between T44 and T45
MSTRG.5209	LOC_Os10g35730	pentatricopeptide repeat domain containing protein, putative, expressed	The expression level of H21 was between T44 and T45
MSTRG.6211	LOC_Os11g10080		The expression level of H21 was between T44 and T45
MSTRG.13762	LOC_Os03g11540	RPA1B - Putative single-stranded DNA binding complex subunit 1, expressed	The expression level of H21 was between T44 and T45
MSTRG.15893	LOC_Os03g53920	kinesin motor domain containing protein, putative, expressed	The expression level of H21 was between T44 and T45
MSTRG.5979	LOC_Os11g05530	expressed protein	The expression level of H21 was between T44 and T45
MSTRG.7079	LOC_Os11g37960	WIP4 - Wound-induced protein precursor, expressed	The expression level of H21 was between T44 and T45
MSTRG.22276	LOC_Os06g06710	expressed protein	The expression level of H21 was between T44 and T45
MSTRG.17507	LOC_Os04g30490	MATE efflux family protein, putative, expressed	The expression level of H21 was between T44 and T45
MSTRG.1718	LOC_Os01g37000	carboxyl-terminal peptidase, putative, expressed	The expression level of H21 was between T44 and T45

MSTRG.9264			The expression level of H21 was between T44 and T45
MSTRG.12490	LOC_Os02g52150	heat shock 22 kDa protein, mitochondrial precursor, putative, expressed	The expression level of H21 was between T44 and T45
MSTRG.27582	LOC_Os08g16030	expressed protein	The expression level of H21 was between T44 and T45
MSTRG.17615	LOC_Os04g32210		The expression level of H21 was between T44 and T45
MSTRG.13350	LOC_Os03g06139		The expression level of H21 was between T44 and T45
MSTRG.19390	LOC_Os04g59510		The expression level of H21 was between T44 and T45
MSTRG.22613	LOC_Os06g12160	AAA-type ATPase family protein, putative, expressed	The expression level of H21 was between T44 and T45
MSTRG.6760	LOC_Os11g29870	WRKY72, expressed	The expression level of H21 was between T44 and T45
MSTRG.21234	LOC_Os05g41480		The expression level of H21 was between T44 and T45
MSTRG.2402	LOC_Os01g49614	Protein kinase domain containing protein, expressed	The expression level of H21 was between T44 and T45
MSTRG.14708	LOC_Os03g26380	transposon protein, putative, unclassified, expressed	The expression level of H21 was between T44 and T45
MSTRG.1380	LOC_Os01g26000	expressed protein	The expression level of H21 was between T44 and T45
MSTRG.27479			The expression level of H21 was between T44 and T45
MSTRG.23830	LOC_Os06g42080	retrotransposon protein, putative, unclassified, expressed	The expression level of H21 was between T44 and T45
MSTRG.27236	LOC_Os08g09000	Cupin domain containing protein, expressed	The expression level of H21 was between T44 and T45
MSTRG.1292	LOC_Os01g23580	inorganic H+ pyrophosphatase, putative, expressed	The expression level of H21 was between T44 and T45
MSTRG.20030			The expression level of H21 was between T44 and T45
MSTRG.5119	LOC_Os10g34720	LOI1, putative, expressed	The expression level of H21 was between T44 and T45
MSTRG.23641	LOC_Os06g39460	transposon protein, putative, CACTA, En/Spm sub-class, expressed	The expression level of H21 was between T44 and T45
MSTRG.7623	LOC_Os12g01449	chloroplast unusual positioning protein, putative, expressed	The expression level of H21 was between T44 and T45
MSTRG.4306	LOC_Os10g08460	expressed protein	The expression level of H21 was between T44 and T45
MSTRG.26652	LOC_Os07g47430	expressed protein	The expression level of H21 was between T44 and T45
MSTRG.22275	LOC_Os06g06680	expressed protein	The expression level of H21 was between T44 and T45
MSTRG.4428	LOC_Os10g13800	ufm1-conjugating enzyme 1, putative, expressed	The expression level of H21 was between T44 and T45
MSTRG.26064	LOC_Os07g38130		The expression level of H21 was between T44 and T45
MSTRG.28706	LOC_Os08g41630		The expression level of H21 was between T44 and T45
MSTRG.8810	LOC_Os12g31450	plastid division regulator MinE, putative, expressed	The expression level of H21 was between T44 and T45
MSTRG.20164	LOC_Os05g15680		The expression level of H21 was between T44 and T45
MSTRG.7329	LOC_Os11g41920	expressed protein	The expression level of H21 was between T44 and T45
MSTRG.1239	LOC_Os01g21970	protein kinase, putative, expressed	The expression level of H21 was between T44 and T45
MSTRG.25760	LOC_Os07g32660	monocopper oxidase, putative, expressed	The expression level of H21 was between T44 and T45
MSTRG.11405	LOC_Os02g34970	no apical meristem protein, putative, expressed	The expression level of H21 was between T44 and T45
MSTRG.18089	LOC_Os04g39880	Os4bglu12 - beta-glucosidase, exo-beta-glucanase, expressed	The expression level of H21 was between T44 and T45
MSTRG.7824	LOC_Os12g04500	response regulator receiver domain containing protein, expressed	The expression level of H21 was between T44 and T45
MSTRG.12511	LOC_Os02g52460	zinc knuckle domain containing protein, expressed	The expression level of H21 was between T44 and T45
MSTRG.17021	LOC_Os04g15660		The expression level of H21 was between T44 and T45
MSTRG.19299	LOC_Os04g58120	CRP2 - Cysteine-rich family protein precursor, expressed	The expression level of H21 was between T44 and T45
MSTRG.2360	LOC_Os01g49030	expressed protein	The expression level of H21 was between T44 and T45
MSTRG.28819	LOC_Os08g43240	LTPL97 - Protease inhibitor/seed storage/LTP family protein precursor, expressed	The expression level of H21 was between T44 and T45
MSTRG.11624			The expression level of H21 was between T44 and T45
MSTRG.19157	LOC_Os04g56210	polyprenyl synthetase, putative, expressed	The expression level of H21 was between T44 and T45
MSTRG.25437	LOC_Os07g23570	cytochrome P450 72A1, putative, expressed	The expression level of H21 was between T44 and T45
MSTRG.12381	LOC_Os02g50350	dihydroorotate dihydrogenase protein, putative, expressed	The expression level of H21 was between T44 and T45
MSTRG.5094	LOC_Os10g34290	retrotransposon protein, putative, unclassified, expressed	The expression level of H21 was between T44 and T45
MSTRG.22708	LOC_Os06g13760	glycosyl transferase 8 domain containing protein, putative, expressed	The expression level of H21 was between T44 and T45
MSTRG.15041	LOC_Os03g38330	disease resistance RPP13-like protein 1, putative, expressed	The expression level of H21 was between T44 and T45
MSTRG.20521			The expression level of H21 was between T44 and T45
MSTRG.18805	LOC_Os04g51480	expressed protein	The expression level of H21 was between T44 and T45
MSTRG.9071	LOC_Os12g36210; LOC_Os12g36220	inhibitor I family protein, putative, expressed	The expression level of H21 was between T44 and T45
MSTRG.30478	LOC_Os09g38020; LOC_Os09g38010	Core histone H2A/H2B/H3/H4 domain containing protein, putative, expressed	The expression level of H21 was between T44 and T45
MSTRG.7241	LOC_Os11g40380	expressed protein	The expression level of H21 was between T44 and T45
MSTRG.21036	LOC_Os05g38260		The expression level of H21 was between T44 and T45
MSTRG.28573	LOC_Os08g39810	retrotransposon protein, putative, unclassified, expressed	The expression level of H21 was between T44 and T45
MSTRG.8083			The expression level of H21 was between T44 and T45
MSTRG.13970	LOC_Os03g14570	expressed protein	The expression level of H21 was between T44 and T45
MSTRG.9092	LOC_Os12g36750	expressed protein	The expression level of H21 was between T44 and T45

MSTRG.22599	LOC_Os06g12020	membrane-anchored ubiquitin-fold protein, putative, expressed	The expression level of H21 was between T44 and T45
MSTRG.9550	LOC_Os12g43780	expressed protein	The expression level of H21 was between T44 and T45
MSTRG.26993	LOC_Os08g04210	cysteine-rich repeat secretory protein 55 precursor, putative, expressed	The expression level of H21 was between T44 and T45
MSTRG.17170	LOC_Os04g19260	retrotransposon protein, putative, unclassified, expressed	The expression level of H21 was between T44 and T45
LOC_Os04g10530	LOC_Os04g10530	amidase, putative, expressed	The expression level of H21 was between T44 and T45
MSTRG.3807	LOC_Os01g70770	glutathione S-transferase, putative, expressed	The expression level of H21 was between T44 and T45
MSTRG.24923	LOC_Os07g08520	auxin response factor, putative, expressed	The expression level of H21 was between T44 and T45
MSTRG.29601	LOC_Os09g21210	beta-glucan-binding protein 4, putative, expressed	The expression level of H21 was between T44 and T45
MSTRG.17340	LOC_Os04g25410	pentatricopeptide repeat domain containing protein, putative, expressed	The expression level of H21 was between T44 and T45
MSTRG.5850	LOC_Os11g03520	GDSL-like lipase/acylhydrolase, putative, expressed	The expression level of H21 was between T44 and T45
MSTRG.20150	LOC_Os05g15470		The expression level of H21 was between T44 and T45
MSTRG.12543	LOC_Os02g52934	expressed protein	The expression level of H21 was between T44 and T45
MSTRG.30269	LOC_Os09g34214	UDP-glucoronosyl and UDP-glucosyl transferase domain containing protein, expressed	The expression level of H21 was between T44 and T45
MSTRG.18131	LOC_Os04g40460	cytochrome P450, putative, expressed	The expression level of H21 was between T44 and T45
MSTRG.7512	LOC_Os11g46000	von Willebrand factor type A domain containing protein, putative, expressed	The expression level of H21 was between T44 and T45
MSTRG.7395	LOC_Os11g43060; LOC_Os11g43070	transposon protein, putative, unclassified, expressed;expressed protein	The expression level of H21 was between T44 and T45
MSTRG.26395	LOC_Os07g43480	expressed protein	The expression level of H21 was between T44 and T45
MSTRG.716	LOC_Os01g11650	GDSL-like lipase/acylhydrolase, putative, expressed	The expression level of H21 was between T44 and T45
MSTRG.7234	LOC_Os11g40230	expressed protein	The expression level of H21 was between T44 and T45
MSTRG.14256	LOC_Os03g18620	PPR repeat domain containing protein, putative, expressed	The expression level of H21 was between T44 and T45
MSTRG.17707	LOC_Os04g33600	hydrolase, alpha/beta fold family protein, putative, expressed	The expression level of H21 was between T44 and T45
MSTRG.894	LOC_Os01g14340		The expression level of H21 was between T44 and T45
MSTRG.9384	LOC_Os12g41390	homocysteine S-methyltransferase protein, putative, expressed	The expression level of H21 was between T44 and T45
MSTRG.17489	LOC_Os04g30200	OsFBL14 - F-box domain and LRR containing protein, expressed	The expression level of H21 was between T44 and T45
MSTRG.27461	LOC_Os08g13350	expressed protein	The expression level of H21 was between T44 and T45
MSTRG.20093			The expression level of H21 was between T44 and T45
MSTRG.12562	LOC_Os02g53120	Peptidase family C50, putative, expressed	The expression level of H21 was between T44 and T45
MSTRG.22540	LOC_Os06g10890	sterol carrier protein-2, putative, expressed	The expression level of H21 was between T44 and T45
MSTRG.6360	LOC_Os11g12330	disease resistance protein RPM1, putative, expressed	The expression level of H21 was between T44 and T45
MSTRG.382	LOC_Os01g05960	receptor kinase, putative, expressed	The expression level of H21 was between T44 and T45
MSTRG.17357	LOC_Os04g25900	go35 NBS-LRR, putative, expressed	The expression level of H21 was between T44 and T45
MSTRG.10693	LOC_Os02g18660	expressed protein	The expression level of H21 was between T44 and T45
MSTRG.15154			The expression level of H21 was between T44 and T45
LOC_Os05g02870	LOC_Os05g02870	white-brown complex homolog protein, putative, expressed	The expression level of H21 was between T44 and T45
MSTRG.29767	LOC_Os09g25330	BTBN19 - Bric-a-Brac, Tramtrack, Broad Complex BTB domain with non-phototropic hypocotyl 3 NPH3 domain, expressed	The expression level of H21 was between T44 and T45
MSTRG.340	LOC_Os01g05510	expressed protein	The expression level of H21 was between T44 and T45
MSTRG.14597	LOC_Os03g24300	LTPL1 - Protease inhibitor/seed storage/LTP family protein precursor, expressed	The expression level of H21 was between T44 and T45
MSTRG.2448	LOC_Os01g50280	expressed protein	The expression level of H21 was between T44 and T45
MSTRG.28526	LOC_Os08g38900	caffeoyl-CoA O-methyltransferase, putative, expressed	The expression level of H21 was between T44 and T45
MSTRG.11091	LOC_Os02g28720	spotted leaf 11, putative, expressed	The expression level of H21 was between T44 and T45
MSTRG.27900	LOC_Os08g26840	plant protein of unknown function domain containing protein, expressed	The expression level of H21 was between T44 and T45
LOC_Os03g03790	LOC_Os03g03790	AMP-binding domain containing protein, expressed	The expression level of H21 was between T44 and T45
MSTRG.18709	LOC_Os04g49560	zinc finger family protein, putative, expressed	The expression level of H21 was between T44 and T45
MSTRG.26135	LOC_Os07g39110	AP2/EREBP transcription factor BABY BOOM, putative, expressed	The expression level of H21 was between T44 and T45
MSTRG.12610	LOC_Os02g53900		The expression level of H21 was between T44 and T45
MSTRG.3036	LOC_Os01g58550	methyladenine glycosylase, putative, expressed	The expression level of H21 was between T44 and T45
MSTRG.19489	LOC_Os05g02040	RPA1C - Putative single-stranded DNA binding complex subunit 1, expressed	The expression level of H21 was between T44 and T45
MSTRG.21241	LOC_Os05g41540	bZIP transcription factor domain containing protein, expressed	The expression level of H21 was between T44 and T45
MSTRG.14096	LOC_Os03g16420	retrotransposon protein, putative, unclassified, expressed	The expression level of H21 was between T44 and T45
MSTRG.17328	LOC_Os04g24830	expressed protein	The expression level of H21 was between T44 and T45

MSTRG.16199	LOC_Os03g58410	prenylated rab acceptor, putative, expressed	The expression level of H21 was between T44 and T45
MSTRG.15429	LOC_Os03g46550	RGH1A, putative, expressed	The expression level of H21 was between T44 and T45
MSTRG.11276	LOC_Os02g32690	pleiotropic drug resistance protein 15, putative, expressed	The expression level of H21 was between T44 and T45
MSTRG.5604	LOC_Os10g42210	enoyl-CoA-hydratase, putative, expressed	The expression level of H21 was between T44 and T45
MSTRG.11472	LOC_Os02g36070	cytochrome P450, putative, expressed	The expression level of H21 was between T44 and T45
MSTRG.3507	LOC_Os01g66010	amino acid transporter, putative, expressed	The expression level of H21 was between T44 and T45
MSTRG.10169	LOC_Os02g08140	CAMK_KIN1/SNF1/Nim1_like.14 - CAMK includes calcium/calmodulin depedent protein kinases, expressed	The expression level of H21 was between T44 and T45
MSTRG.27158			The expression level of H21 was between T44 and T45
MSTRG.8798	LOC_Os12g31000	pumilio-family RNA binding repeat domain containing protein, expressed	The expression level of H21 was between T44 and T45
MSTRG.29611	LOC_Os09g21290	expressed protein	The expression level of H21 was between T44 and T45
MSTRG.23679	LOC_Os06g40180	phospholipase D, putative, expressed	The expression level of H21 was between T44 and T45
MSTRG.3796	LOC_Os01g70525		The expression level of H21 was between T44 and T45
MSTRG.8609	LOC_Os12g24650		The expression level of H21 was between T44 and T45
MSTRG.27143	LOC_Os08g06730; LOC_Os08g06720	expressed protein	The expression level of H21 was between T44 and T45
MSTRG.943	LOC_Os01g15290	ribosomal L18p/L5e family protein, putative, expressed	The expression level of H21 was between T44 and T45
MSTRG.27931	LOC_Os08g27720	pirin, putative, expressed	The expression level of H21 was between T44 and T45
MSTRG.4714	LOC_Os10g25170	AP2 domain containing protein, expressed	The expression level of H21 was between T44 and T45
MSTRG.11029	LOC_Os02g27160	retrotransposon protein, putative, unclassified, expressed	The expression level of H21 was between T44 and T45
MSTRG.26158	LOC_Os07g39460	transposon protein, putative, unclassified, expressed	The expression level of H21 was between T44 and T45
MSTRG.10172	LOC_Os02g08220	expressed protein	The expression level of H21 was between T44 and T45
MSTRG.22895	LOC_Os06g18670	anthocyanidin 3-O-glucosyltransferase, putative, expressed	The expression level of H21 was between T44 and T45
MSTRG.23594	LOC_Os06g38480	retrotransposon protein, putative, unclassified, expressed	The expression level of H21 was between T44 and T45
MSTRG.18147	LOC_Os04g40680	expressed protein	The expression level of H21 was between T44 and T45
MSTRG.24742	LOC_Os07g05670	expressed protein	The expression level of H21 was between T44 and T45
LOC_Os11g31715	LOC_Os11g31715	expressed protein	The expression level of H21 was between T44 and T45
MSTRG.11740	LOC_Os02g40080	tic20, putative, expressed	The expression level of H21 was between T44 and T45
MSTRG.23419	LOC_Os06g34440; LOC_Os06g34430	zinc finger protein, putative, expressed;dnaJ domain containing protein, expressed	The expression level of H21 was between T44 and T45
MSTRG.15208	LOC_Os03g41920	expressed protein	The expression level of H21 was between T44 and T45
MSTRG.22273	LOC_Os06g06670	expressed protein	The expression level of H21 was between T44 and T45
MSTRG.21400	LOC_Os05g44200	GDSL-like lipase/acylhydrolase, putative, expressed	The expression level of H21 was between T44 and T45
MSTRG.24434	LOC_Os06g50940	GDSL-like lipase/acylhydrolase, putative, expressed	The expression level of H21 was between T44 and T45
MSTRG.4379	LOC_Os10g11100	retrotransposon protein, putative, unclassified, expressed	The expression level of H21 was between T44 and T45
MSTRG.3798	LOC_Os01g70540	retrotransposon protein, putative, unclassified, expressed	The expression level of H21 was between T44 and T45
MSTRG.20013	LOC_Os05g11560	aquaporin protein, putative, expressed	The expression level of H21 was between T44 and T45
MSTRG.7093	LOC_Os11g38160	transporter family protein, putative, expressed	The expression level of H21 was between T44 and T45
MSTRG.4034	LOC_Os01g74300	metallothionein, putative, expressed	The expression level of H21 was between T44 and T45
MSTRG.10605	LOC_Os02g16480; LOC_Os02g16490	nucleolar transcription factor 1, putative, expressed;expressed protein	The expression level of H21 was between T44 and T45
MSTRG.683	LOC_Os01g11054	phosphoenolpyruvate carboxylase, putative, expressed	The expression level of H21 was between T44 and T45
MSTRG.27191	LOC_Os08g07840		The expression level of H21 was between T44 and T45
MSTRG.3964	LOC_Os01g73200	peroxidase precursor, putative, expressed	The expression level of H21 was between T44 and T45
MSTRG.17322	LOC_Os04g24600	cysteine proteinase 1 precursor, putative, expressed	The expression level of H21 was between T44 and T45
MSTRG.8746	LOC_Os12g29740	expressed protein	The expression level of H21 was between T44 and T45
MSTRG.13001	LOC_Os03g01170	transporter family protein, putative, expressed	The expression level of H21 was between T44 and T45
MSTRG.10326	LOC_Os02g10920	zinc finger family protein, putative, expressed	The expression level of H21 was between T44 and T45
MSTRG.18348	LOC_Os04g43560	no apical meristem protein, putative, expressed	The expression level of H21 was between T44 and T45
MSTRG.6891	LOC_Os11g34080	expressed protein	The expression level of H21 was between T44 and T45
MSTRG.8009	LOC_Os12g07580	dirigent, putative, expressed	The expression level of H21 was between T44 and T45
MSTRG.18129	LOC_Os04g40450	retrotransposon protein, putative, unclassified, expressed	The expression level of H21 was between T44 and T45
MSTRG.2270	LOC_Os01g47580	lipid phosphatase protein, putative, expressed	The expression level of H21 was between T44 and T45
MSTRG.17234	LOC_Os04g21705	PB1 domain containing protein, expressed	The expression level of H21 was between T44 and T45
MSTRG.5923	LOC_Os11g04800	expressed protein	The expression level of H21 was between T44 and T45
MSTRG.2075	LOC_Os01g43530	late embryogenesis abundant protein, putative, expressed	The expression level of H21 was between T44 and T45

MSTRG.14447	LOC_Os03g21540	TKL_IRAK_CrRLK1L-1.8 - The CrRLK1L-1 subfamily has homology to the CrRLK1L homolog, expressed	The expression level of H21 was between T44 and T45
MSTRG.25913	LOC_Os07g35960	expressed protein	The expression level of H21 was between T44 and T45
MSTRG.14935	LOC_Os03g32790	expressed protein	The expression level of H21 was between T44 and T45
MSTRG.161	LOC_Os01g03980	expressed protein	The expression level of H21 was between T44 and T45
MSTRG.12687	LOC_Os02g55090	expressed protein	The expression level of H21 was between T44 and T45
MSTRG.29472	LOC_Os09g17049		The expression level of H21 was between T44 and T45
MSTRG.25041	LOC_Os07g10550	cyclin-related protein, putative, expressed	The expression level of H21 was between T44 and T45
MSTRG.24439	LOC_Os06g51050	CHIT7 - Chitinase family protein precursor, expressed	The expression level of H21 was between T44 and T45
MSTRG.7074	LOC_Os11g37850	stripe rust resistance protein Yr10, putative, expressed	The expression level of H21 was between T44 and T45
MSTRG.22209	LOC_Os06g05790		The expression level of H21 was between T44 and T45
MSTRG.21421	LOC_Os05g44630	IQ calmodulin-binding motif family protein, putative, expressed	The expression level of H21 was between T44 and T45
MSTRG.16507	LOC_Os03g62780	S1 RNA binding domain containing protein, expressed	The expression level of H21 was between T44 and T45
MSTRG.14008	LOC_Os03g15220	DUF292 domain containing protein, expressed	The expression level of H21 was between T44 and T45
MSTRG.9895	LOC_Os02g04924	retrotransposon protein, putative, unclassified, expressed	The expression level of H21 was between T44 and T45
MSTRG.3760	LOC_Os01g70110	No apical meristem protein, putative, expressed	The expression level of H21 was between T44 and T45
MSTRG.29984	LOC_Os09g29270	expressed protein	The expression level of H21 was between T44 and T45
MSTRG.30097	LOC_Os09g31280		The expression level of H21 was between T44 and T45
MSTRG.27163	LOC_Os08g07040		The expression level of H21 was between T44 and T45
MSTRG.18227	LOC_Os04g41820	transcription factor RF2a, putative, expressed	The expression level of H21 was between T44 and T45
MSTRG.17316	LOC_Os04g24414	expressed protein	The expression level of H21 was between T44 and T45
MSTRG.3906	LOC_Os01g72350	amidohydrolase, putative, expressed	The expression level of H21 was between T44 and T45
MSTRG.17124	LOC_Os04g17479	expressed protein	The expression level of H21 was between T44 and T45
MSTRG.25367	LOC_Os07g19444	regulatory protein, putative, expressed	The expression level of H21 was between T44 and T45
MSTRG.4032	LOC_Os01g74280	TGD2, putative, expressed	The expression level of H21 was between T44 and T45
MSTRG.5761	LOC_Os11g02379	LTPL6 - Protease inhibitor/seed storage/LTP family protein precursor, expressed	The expression level of H21 was between T44 and T45
MSTRG.20906	LOC_Os05g35266		The expression level of H21 was between T44 and T45
MSTRG.7694	LOC_Os12g02570	expressed protein	The expression level of H21 was between T44 and T45
MSTRG.21932	LOC_Os06g01620		The expression level of H21 was between T44 and T45
MSTRG.30327			The expression level of H21 was between T44 and T45
MSTRG.13408	LOC_Os03g06930	homeodomain protein, putative, expressed	The expression level of H21 was between T44 and T45
MSTRG.29772	LOC_Os09g25370	deoxyhypusine synthase, putative, expressed	The expression level of H21 was between T44 and T45
MSTRG.21658	LOC_Os05g48500	expressed protein	The expression level of H21 was between T44 and T45
MSTRG.22047	LOC_Os06g03900	esterase precursor, putative, expressed	The expression level of H21 was between T44 and T45
MSTRG.28788	LOC_Os08g42750	CAMK_CAMK_like.37 - CAMK includes calcium/calmodulin depedent protein kinases, expressed	The expression level of H21 was between T44 and T45
MSTRG.16408	LOC_Os03g61220	DEAD-box ATP-dependent RNA helicase 3, putative, expressed	The expression level of H21 was between T44 and T45
MSTRG.28626	LOC_Os08g40420	ternary complex factor MIP1, putative, expressed	The expression level of H21 was between T44 and T45
MSTRG.11261	LOC_Os02g32520	ERD1 protein, chloroplast precursor, putative, expressed	The expression level of H21 was between T44 and T45
MSTRG.11759			The expression level of H21 was between T44 and T45
MSTRG.3132	LOC_Os01g59930	NADH-cytochrome b5 reductase, putative, expressed	The expression level of H21 was between T44 and T45
MSTRG.25943	LOC_Os07g36300	OsFBX243 - F-box domain containing protein, expressed	The expression level of H21 was between T44 and T45
MSTRG.23734	LOC_Os06g40940	glycine dehydrogenase, putative, expressed	The expression level of H21 was between T44 and T45
MSTRG.29434	LOC_Os09g15800;<br>LOC_Os09g15790	ras-related protein, putative, expressed;tetratricopeptide-like helical, putative, expressed	The expression level of H21 was between T44 and T45
MSTRG.25759	LOC_Os07g32650	retrotransposon protein, putative, unclassified, expressed	The expression level of H21 was between T44 and T45
MSTRG.17345	LOC_Os04g25540	ribosomal protein L27, putative, expressed	The expression level of H21 was between T44 and T45
MSTRG.3474	LOC_Os01g65590	galactosyltransferase, putative, expressed	The expression level of H21 was between T44 and T45
MSTRG.22133	LOC_Os06g04870	homeobox associated leucine zipper, putative, expressed	The expression level of H21 was between T44 and T45
MSTRG.18812	LOC_Os04g51500	expressed protein	The expression level of H21 was between T44 and T45
MSTRG.16159	LOC_Os03g57920	AN1-like zinc finger domain containing protein, expressed	The expression level of H21 was between T44 and T45
MSTRG.3426	LOC_Os01g64820	POLA1 - Putative DNA polymerase alpha catalytic subunit, expressed	The expression level of H21 was between T44 and T45
LOC_Os04g35890	LOC_Os04g35890	TKL_IRAK_CR4L.3 - The CR4L subfamily has homology with Crinkly4, expressed	The expression level of H21 was between T44 and T45
MSTRG.26567	LOC_Os07g46350	OsSCP40 - Putative Serine Carboxypeptidase homologue, expressed	The expression level of H21 was between T44 and T45

MSTRG.15118	LOC_Os03g39830	expressed protein	The expression level of H21 was between T44 and T45
MSTRG.29334	LOC_Os09g12780	expressed protein	The expression level of H21 was between T44 and T45
MSTRG.5222	LOC_Os10g35870	cytochrome b5-like Heme/Steroid binding domain containing protein, expressed	The expression level of H21 was between T44 and T45
MSTRG.19477	LOC_Os05g01940	zinc finger, RING-type, putative, expressed	The expression level of H21 was between T44 and T45
MSTRG.7099	LOC_Os11g38250	expressed protein	The expression level of H21 was between T44 and T45
MSTRG.4804	LOC_Os10g27380	expressed protein	The expression level of H21 was between T44 and T45
MSTRG.26686	LOC_Os07g48080	expressed protein	The expression level of H21 was between T44 and T45
MSTRG.23078	LOC_Os06g23360	LTPL70 - Protease inhibitor/seed storage/LTP family protein precursor, expressed	The expression level of H21 was between T44 and T45
MSTRG.28983	LOC_Os09g01000		The expression level of H21 was between T44 and T45
MSTRG.1938	LOC_Os01g41750	expressed protein	The expression level of H21 was between T44 and T45
MSTRG.3553	LOC_Os01g66610	serine/threonine-protein kinase receptor precursor, putative, expressed	The expression level of H21 was between T44 and T45
MSTRG.211	LOC_Os01g03100	protein binding protein, putative, expressed	The expression level of H21 was between T44 and T45
MSTRG.28649	LOC_Os08g40780	hypothetical protein	The expression level of H21 was between T44 and T45
MSTRG.18845	LOC_Os04g52020		The expression level of H21 was between T44 and T45
MSTRG.13197	LOC_Os03g03810	DEF8 - Defensin and Defensin-like DEFL family, expressed	The expression level of H21 was between T44 and T45
MSTRG.18829	LOC_Os04g51800;<br>LOC_Os04g51809	MYB protein, putative, expressed;expressed protein	The expression level of H21 was between T44 and T45
MSTRG.84	LOC_Os01g02190	aquaporin protein, putative, expressed	The expression level of H21 was between T44 and T45
MSTRG.23602	LOC_Os06g38660	expressed protein	The expression level of H21 was between T44 and T45
MSTRG.15167	LOC_Os03g40720	UDP-glucose 6-dehydrogenase, putative, expressed	The expression level of H21 was between T44 and T45
MSTRG.17229	LOC_Os04g21590	PB1 domain containing protein, expressed	The expression level of H21 was between T44 and T45
MSTRG.6111	LOC_Os11g07922;<br>LOC_Os11g07930	short chain dehydrogenase/reductase protein, putative, expressed;oxidoreductase, short chain dehydrogenase/reductase family domain containing family, expressed	The expression level of H21 was between T44 and T45
MSTRG.16552	LOC_Os03g63480	ankyrin repeat domain containing protein, expressed	The expression level of H21 was between T44 and T45
MSTRG.17881	LOC_Os04g36040	peptide transporter PTR2, putative, expressed	The expression level of H21 was between T44 and T45
MSTRG.22237	LOC_Os06g06170	expressed protein	The expression level of H21 was between T44 and T45
MSTRG.26530	LOC_Os07g45780	expressed protein	The expression level of H21 was between T44 and T45
MSTRG.19138	LOC_Os04g55840	expressed protein	The expression level of H21 was between T44 and T45
MSTRG.21518	LOC_Os05g46240	green ripe-like, putative, expressed	The expression level of H21 was between T44 and T45
MSTRG.16883	LOC_Os04g10470	expressed protein	The expression level of H21 was between T44 and T45
LOC_Os08g28890	LOC_Os08g28890	protein kinase family protein, putative, expressed	The expression level of H21 was between T44 and T45
MSTRG.15207			The expression level of H21 was between T44 and T45
MSTRG.29572	LOC_Os09g20430		The expression level of H21 was between T44 and T45
MSTRG.11760	LOC_Os02g40680	mis12 protein, expressed	The expression level of H21 was between T44 and T45
MSTRG.18663			The expression level of H21 was between T44 and T45
MSTRG.1004	LOC_Os01g16220	Sad1 / UNC-like C-terminal domain containing protein, putative, expressed	The expression level of H21 was between T44 and T45
MSTRG.30484	LOC_Os09g37920	helicase, putative, expressed	The expression level of H21 was between T44 and T45
MSTRG.17030	LOC_Os04g15840	expansin precursor, putative, expressed	The expression level of H21 was between T44 and T45
MSTRG.28518	LOC_Os08g38790	expressed protein	The expression level of H21 was between T44 and T45
MSTRG.7902	LOC_Os12g06010	retrotransposon protein, putative, unclassified, expressed	The expression level of H21 was between T44 and T45
MSTRG.29464	LOC_Os09g16920	desaturase/cytochrome b5 protein, putative, expressed	The expression level of H21 was between T44 and T45
MSTRG.17903	LOC_Os04g36740	potassium channel SKOR, putative, expressed	The expression level of H21 was between T44 and T45
MSTRG.24450	LOC_Os06g50630;<br>LOC_Os06g50780	expressed protein	The expression level of H21 was between T44 and T45
MSTRG.21537	LOC_Os05g46370	bHelix-loop-helix transcription factor, putative, expressed	The expression level of H21 was between T44 and T45
MSTRG.6266	LOC_Os11g10990	heat shock protein DnaJ, putative, expressed	The expression level of H21 was between T44 and T45
MSTRG.17039	LOC_Os04g16680	fructose-1,6-bisphosphatase, putative, expressed	The expression level of H21 was between T44 and T45
MSTRG.5298	LOC_Os10g37430	expressed protein	The expression level of H21 was between T44 and T45
MSTRG.10111	LOC_Os02g07330	expressed protein	The expression level of H21 was between T44 and T45
MSTRG.8257	LOC_Os12g13640	glycosyltransferase, putative, expressed	The expression level of H21 was between T44 and T45
MSTRG.23235	LOC_Os06g28530	retrotransposon protein, putative, unclassified, expressed	The expression level of H21 was between T44 and T45
MSTRG.25942	LOC_Os07g36270	retrotransposon protein, putative, Ty1-copia subclass, expressed	The expression level of H21 was between T44 and T45
MSTRG.21918	LOC_Os06g01370	DNA-binding protein-related, putative, expressed	The expression level of H21 was between T44 and T45
MSTRG.16334	LOC_Os03g60419	expressed protein	The expression level of H21 was between T44 and T45
MSTRG.760	LOC_Os01g12320	GDSL-like lipase/acylhydrolase, putative, expressed	The expression level of H21 was between T44 and T45
MSTRG.17931	LOC_Os04g37570	aspartic proteinase nepenthesin precursor, putative, expressed	The expression level of H21 was between T44 and T45
MSTRG.11361			The expression level of H21 was between T44 and T45
MSTRG.860	LOC_Os01g13780	expressed protein	The expression level of H21 was between T44 and T45

MSTRG.4720	LOC_Os10g25260		The expression level of H21 was between T44 and T45
MSTRG.28514	LOC_Os08g38740	glycosyl transferase 8 domain containing protein, putative, expressed	The expression level of H21 was between T44 and T45
MSTRG.6190			The expression level of H21 was between T44 and T45
MSTRG.15756	LOC_Os03g51710	homeobox protein knotted-1, putative, expressed	The expression level of H21 was between T44 and T45
MSTRG.4729			The expression level of H21 was between T44 and T45
MSTRG.27320	LOC_Os08g10630	metal cation transporter, putative, expressed	The expression level of H21 was between T44 and T45
MSTRG.4170	LOC_Os10g04342	stripe rust resistance protein Yr10, putative, expressed	The expression level of H21 was between T44 and T45
MSTRG.9971	LOC_Os02g05400; LOC_Os02g05350; LOC_Os02g05360; LOC_Os02g05430	expressed protein	The expression level of H21 was between T44 and T45
MSTRG.8747	LOC_Os12g29680; LOC_Os12g29690	NBS-LRR disease resistance protein, putative, expressed	The expression level of H21 was between T44 and T45
MSTRG.9595	LOC_Os02g01190		The expression level of H21 was between T44 and T45
MSTRG.18390	LOC_Os04g44590	expressed protein	The expression level of H21 was between T44 and T45
LOC_Os10g02210	LOC_Os10g02210	peptide transporter PTR2, putative, expressed	The expression level of H21 was between T44 and T45
MSTRG.9245	LOC_Os12g39450		The expression level of H21 was between T44 and T45
MSTRG.24014	LOC_Os06g44310	tetraspanin family protein, putative, expressed	The expression level of H21 was between T44 and T45
MSTRG.14444			The expression level of H21 was between T44 and T45
MSTRG.5996	LOC_Os11g05870	harpin-induced protein 1 domain containing protein, expressed	The expression level of H21 was between T44 and T45
MSTRG.14356	LOC_Os03g19900	AP2 domain containing protein, expressed	The expression level of H21 was between T44 and T45
MSTRG.17160			The expression level of H21 was between T44 and T45
MSTRG.5648	LOC_Os10g42830	transporter family protein, putative, expressed	The expression level of H21 was between T44 and T45
MSTRG.13866	LOC_Os03g13040	haemolysin-III, putative, expressed	The expression level of H21 was between T44 and T45
MSTRG.25834	LOC_Os07g33850	ras-related protein, putative, expressed	The expression level of H21 was between T44 and T45
MSTRG.19239	LOC_Os04g57400; LOC_Os04g57410	methylthioribose kinase, putative, expressed	The expression level of H21 was between T44 and T45
MSTRG.6010	LOC_Os11g06130	PHD-finger family protein, expressed	The expression level of H21 was between T44 and T45
MSTRG.18946	LOC_Os04g53120; LOC_Os04g53160	NB-ARC domain containing protein, expressed;NBS-LRR disease resistance protein, putative, expressed	The expression level of H21 was between T44 and T45
MSTRG.22434	LOC_Os06g08990; LOC_Os06g09020	expressed protein	The expression level of H21 was between T44 and T45
MSTRG.27699	LOC_Os08g19950; LOC_Os08g19910	expressed protein;retrotransposon protein, putative, Ty3-gypsy subclass	The expression level of H21 was between T44 and T45
MSTRG.27939	LOC_Os08g27860	EARLY flowering protein, putative, expressed	The expression level of H21 was between T44 and T45
MSTRG.18811	LOC_Os04g51580	leucine rich repeat containing protein, expressed	The expression level of H21 was between T44 and T45
MSTRG.23434	LOC_Os06g34810		The expression level of H21 was between T44 and T45
MSTRG.24812	LOC_Os07g07070	anthranilate phosphoribosyltransferase, putative, expressed	The expression level of H21 was between T44 and T45
MSTRG.20123	LOC_Os05g14750	AGC_PVPK_like_kin82y.12 - ACG kinases include homologs to PKA, PKG and PKC, expressed	The expression level of H21 was between T44 and T45
MSTRG.19748			The expression level of H21 was between T44 and T45
MSTRG.25500	LOC_Os07g25810	retrotransposon protein, putative, unclassified, expressed	The expression level of H21 was between T44 and T45
MSTRG.9206	LOC_Os12g38880	tetratricopeptide-like helical, putative, expressed	The expression level of H21 was between T44 and T45
MSTRG.6066	LOC_Os11g07060	receptor protein kinase CLAVATA1 precursor, putative, expressed	The expression level of H21 was between T44 and T45
LOC_Os05g06480	LOC_Os05g06480	inorganic H+ pyrophosphatase, putative, expressed	The expression level of H21 was between T44 and T45
LOC_Os01g16370	LOC_Os01g16370	NBS-LRR type disease resistance protein, putative, expressed	The expression level of H21 was between T44 and T45
MSTRG.16462	LOC_Os03g61990	glycine-rich RNA-binding protein 7, putative, expressed	The expression level of H21 was between T44 and T45
MSTRG.18099	LOC_Os04g39930; LOC_Os04g39910	receptor-like protein kinase, putative, expressed	The expression level of H21 was between T44 and T45
MSTRG.1791	LOC_Os01g38970	carbamoyl-phosphate synthase large chain, putative, expressed	The expression level of H21 was between T44 and T45
MSTRG.6767	LOC_Os11g29960	retrotransposon protein, putative, unclassified, expressed	The expression level of H21 was between T44 and T45
MSTRG.6658	LOC_Os11g25780	PB1 domain containing protein, expressed	The expression level of H21 was between T44 and T45
MSTRG.13999	LOC_Os03g15010; LOC_Os03g15020		The expression level of H21 was between T44 and T45
MSTRG.1199	LOC_Os01g21250	late embryogenesis abundant protein, putative, expressed	The expression level of H21 was between T44 and T45
MSTRG.28289	LOC_Os08g34300	retrotransposon protein, putative, unclassified, expressed	The expression level of H21 was between T44 and T45
MSTRG.21271	LOC_Os05g42000	peroxidase precursor, putative, expressed	The expression level of H21 was between T44 and T45
MSTRG.23872	LOC_Os06g43600	LTPL129 - Protease inhibitor/seed storage/LTP family protein precursor, expressed	The expression level of H21 was between T44 and T45

MSTRG.29571	LOC_Os09g20390	uncharacterized glycosyl hydrolase Rv2006/MT2062, putative, expressed	The expression level of H21 was between T44 and T45
MSTRG.643	LOC_Os01g10400	expressed protein	The expression level of H21 was between T44 and T45
MSTRG.5254	LOC_Os10g36550	CRP3 - Cysteine-rich family protein precursor, expressed	The expression level of H21 was between T44 and T45
MSTRG.24123	LOC_Os06g46284	glycosyl hydrolase, family 31, putative, expressed	The expression level of H21 was between T44 and T45
MSTRG.2207	LOC_Os01g46340	chloroplast unusual positioning protein, putative, expressed	The expression level of H21 was between T44 and T45
MSTRG.4441	LOC_Os10g14295		The expression level of H21 was between T44 and T45
MSTRG.30545	LOC_Os09g38970	zinc finger family protein, putative, expressed	The expression level of H21 was between T44 and T45
MSTRG.25193	LOC_Os07g13810	cytokinin-N-glucosyltransferase 1, putative, expressed	The expression level of H21 was between T44 and T45
MSTRG.3111	LOC_Os01g59680	NHL25, putative, expressed	The expression level of H21 was between T44 and T45
MSTRG.30409	LOC_Os09g36982	protein of unknown function DUF1279 domain containing protein, expressed	The expression level of H21 was between T44 and T45
MSTRG.9809	LOC_Os02g03840		The expression level of H21 was between T44 and T45
MSTRG.5203	LOC_Os10g35670	zinc finger, RING-type, putative, expressed	The expression level of H21 was between T44 and T45
MSTRG.30151	LOC_Os09g32360	gp176, putative, expressed	The expression level of H21 was between T44 and T45
MSTRG.20173	LOC_Os05g16060		The expression level of H21 was between T44 and T45
MSTRG.26830	LOC_Os08g01514	expressed protein	The expression level of H21 was between T44 and T45
LOC_Os11g44380	LOC_Os11g44380	expressed protein	The expression level of H21 was between T44 and T45
MSTRG.5905	LOC_Os11g04490	astaxanthin synthase KC28, putative, expressed	The expression level of H21 was between T44 and T45
MSTRG.30572	LOC_Os09g39370	expressed protein	The expression level of H21 was between T44 and T45
MSTRG.4243	LOC_Os10g06270	retrotransposon protein, putative, Ty3-gypsy subclass, expressed	The expression level of H21 was between T44 and T45
MSTRG.26495	LOC_Os07g45250	transposon protein, putative, unclassified, expressed	The expression level of H21 was between T44 and T45
MSTRG.30290	LOC_Os09g34930	3-ketoacyl-CoA synthase precursor, putative, expressed	The expression level of H21 was between T44 and T45
MSTRG.4347	LOC_Os10g10149	expressed protein	The expression level of H21 was between T44 and T45
MSTRG.18405	LOC_Os04g44730;<br>LOC_Os04g44740	lanC-like protein 2, putative, expressed;glycosyltransferase sugar-binding region containing DXD motif, putative, expressed	The expression level of H21 was between T44 and T45
MSTRG.12039	LOC_Os02g45450	dehydration-responsive element-binding protein, putative, expressed	The expression level of H21 was between T44 and T45
MSTRG.24552	LOC_Os07g02000	expressed protein	The expression level of H21 was between T44 and T45
MSTRG.17371			The expression level of H21 was between T44 and T45
MSTRG.6411	LOC_Os11g14060	expressed protein	The expression level of H21 was between T44 and T45
MSTRG.23638	LOC_Os06g39370	OsFBK16 - F-box domain and kelch repeat containing protein, expressed	The expression level of H21 was between T44 and T45
MSTRG.5956	LOC_Os11g05190	phytosulfokines precursor, putative, expressed	The expression level of H21 was between T44 and T45
MSTRG.11506	LOC_Os02g36870	YGL010w, putative, expressed	The expression level of H21 was between T44 and T45
MSTRG.16573	LOC_Os03g63750	HSF-type DNA-binding domain containing protein, expressed	The expression level of H21 was between T44 and T45
MSTRG.338	LOC_Os01g05480	expressed protein	The expression level of H21 was between T44 and T45
MSTRG.794	LOC_Os01g12820	harpin-induced protein, putative, expressed	The expression level of H21 was between T44 and T45
MSTRG.28331	LOC_Os08g34879		The expression level of H21 was between T44 and T45
MSTRG.17311	LOC_Os04g24290	wall-associated receptor kinase 3 precursor, putative, expressed	The expression level of H21 was between T44 and T45
MSTRG.2960	LOC_Os01g57550	nodulation protein-related, putative, expressed	The expression level of H21 was between T44 and T45
MSTRG.8852	LOC_Os12g32280	SWIB/MDM2 domain containing protein, expressed	The expression level of H21 was between T44 and T45
MSTRG.248	LOC_Os01g03500	expressed protein	The expression level of H21 was between T44 and T45
MSTRG.26493	LOC_Os07g45194	expressed protein	The expression level of H21 was between T44 and T45
MSTRG.16403	LOC_Os03g61140	expressed protein	The expression level of H21 was between T44 and T45
MSTRG.19564	LOC_Os05g03060	expressed protein	The expression level of H21 was between T44 and T45
MSTRG.17771	LOC_Os04g34590;<br>LOC_Os04g34580	FAD binding domain containing protein, expressed	The expression level of H21 was between T44 and T45
MSTRG.17258	LOC_Os04g22680		The expression level of H21 was between T44 and T45
MSTRG.19763	LOC_Os05g06340	GATA zinc finger domain containing protein, expressed	The expression level of H21 was between T44 and T45
MSTRG.28188	LOC_Os08g32630	FAD dependent oxidoreductase, putative, expressed	The expression level of H21 was between T44 and T45
MSTRG.5159	LOC_Os10g35470	oxidoreductase, putative, expressed	The expression level of H21 was between T44 and T45
MSTRG.9706			The expression level of H21 was between T44 and T45
MSTRG.21448	LOC_Os05g45230		The expression level of H21 was between T44 and T45
MSTRG.17318	LOC_Os04g24530	AMP-binding domain containing protein, expressed	The expression level of H21 was between T44 and T45
LOC_Os04g32620	LOC_Os04g32620	ethylene-responsive transcription factor ERF114, putative, expressed	The expression level of H21 was between T44 and T45
MSTRG.18133	LOC_Os04g40490	glycosyl hydrolase family 5 protein, putative, expressed	The expression level of H21 was between T44 and T45
MSTRG.15862	LOC_Os03g53450	expressed protein	The expression level of H21 was between T44 and T45
MSTRG.4168	LOC_Os10g04280	DNA-directed RNA polymerase II subunit RPB1, putative, expressed	The expression level of H21 was between T44 and T45
MSTRG.26685	LOC_Os07g48020	peroxidase precursor, putative, expressed	The expression level of H21 was between T44 and T45
MSTRG.13814	LOC_Os03g12238		The expression level of H21 was between T44 and T45

LOC_Os06g44970	LOC_Os06g44970	auxin efflux carrier component, putative, expressed	The expression level of H21 was between T44 and T45
MSTRG.18117	LOC_Os04g40200	HNH endonuclease family protein, putative, expressed	The expression level of H21 was between T44 and T45
MSTRG.12880	LOC_Os02g57520	DNA binding protein, putative, expressed	The expression level of H21 was between T44 and T45
MSTRG.24359	LOC_Os06g49710	expressed protein	The expression level of H21 was between T44 and T45
MSTRG.22028	LOC_Os06g03640	BAG domain containing protein, expressed	The expression level of H21 was between T44 and T45
MSTRG.25902	LOC_Os07g35390	TKL_IRAK_DUF26-lc.17 - DUF26 kinases have homology to DUF26 containing loci, expressed	The expression level of H21 was between T44 and T45
MSTRG.4495	LOC_Os10g17790	remorin C-terminal domain containing protein, putative, expressed	The expression level of H21 was between T44 and T45
MSTRG.14028	LOC_Os03g15530	expressed protein	The expression level of H21 was between T44 and T45
MSTRG.26647	LOC_Os07g47350	potassium transporter, putative, expressed	The expression level of H21 was between T44 and T45
MSTRG.22099	LOC_Os06g04399	hypersensitive-induced reaction protein 4, putative, expressed	The expression level of H21 was between T44 and T45
MSTRG.620	LOC_Os01g09990	helix-loop-helix DNA-binding domain containing protein, expressed	The expression level of H21 was between T44 and T45
MSTRG.30166	LOC_Os09g32540	expressed protein	The expression level of H21 was between T44 and T45
MSTRG.1907	LOC_Os01g41220	DUF538 domain containing protein, putative, expressed	The expression level of H21 was between T44 and T45
MSTRG.22470	LOC_Os06g09620	expressed protein	The expression level of H21 was between T44 and T45
MSTRG.8868	LOC_Os12g32480		The expression level of H21 was between T44 and T45
MSTRG.17932	LOC_Os04g37580	cation transport regulator-like protein 1, putative, expressed	The expression level of H21 was between T44 and T45
MSTRG.21181	LOC_Os05g40500; LOC_Os05g40520; LOC_Os05g40590; LOC_Os05g40610	F-box domain containing protein, expressed;expressed protein;OsFBDUF25 - F-box and DUF domain containing protein, expressed	The expression level of H21 was between T44 and T45
MSTRG.7560	LOC_Os11g47670	thaumatin family domain containing protein, expressed	The expression level of H21 was between T44 and T45
MSTRG.14978	LOC_Os03g36630	expressed protein	The expression level of H21 was between T44 and T45
MSTRG.16875	LOC_Os04g10350	1-aminocyclopropane-1-carboxylate oxidase homolog 2, putative, expressed	The expression level of H21 was between T44 and T45
MSTRG.6413	LOC_Os11g14070	expressed protein	The expression level of H21 was between T44 and T45
MSTRG.27543	LOC_Os08g15020	MYB family transcription factor, putative, expressed	The expression level of H21 was between T44 and T45
MSTRG.20703	LOC_Os05g32170		The expression level of H21 was between T44 and T45
MSTRG.7913	LOC_Os12g06140		The expression level of H21 was between T44 and T45
MSTRG.24530	LOC_Os07g01560	transporter family protein, putative, expressed	The expression level of H21 was between T44 and T45
LOC_Os11g08400	LOC_Os11g08400	expressed protein	The expression level of H21 was between T44 and T45
MSTRG.6063	LOC_Os11g07020	fructose-bisphospate aldolase isozyme, putative, expressed	The expression level of H21 was between T44 and T45
MSTRG.5754	LOC_Os11g02300; LOC_Os11g02305	CPuORF36 - conserved peptide uORF-containing transcript, expressed;expressed protein	The expression level of H21 was between T44 and T45
MSTRG.9255	LOC_Os12g39520	OsFBDUF66 - F-box and DUF domain containing protein, expressed	The expression level of H21 was between T44 and T45
MSTRG.24514	LOC_Os07g01230	expressed protein	The expression level of H21 was between T44 and T45
MSTRG.4846	LOC_Os10g28680	DUF581 domain containing protein, expressed	The expression level of H21 was between T44 and T45
MSTRG.17987	LOC_Os04g38500		The expression level of H21 was between T44 and T45
MSTRG.4897	LOC_Os10g30150	universal stress protein domain containing protein, putative, expressed	The expression level of H21 was between T44 and T45
MSTRG.18838	LOC_Os04g51930	retrotransposon protein, putative, LINE subclass, expressed	The expression level of H21 was between T44 and T45
MSTRG.7828	LOC_Os12g04570		The expression level of H21 was between T44 and T45
MSTRG.24910	LOC_Os07g08420	bZIP transcription factor domain containing protein, expressed	The expression level of H21 was between T44 and T45
MSTRG.29969	LOC_Os09g28840	OsSCP43 - Putative Serine Carboxypeptidase homologue, expressed	The expression level of H21 was between T44 and T45
MSTRG.3705	LOC_Os01g68900	zinc finger, C3HC4 type family protein, expressed	The expression level of H21 was between T44 and T45
MSTRG.15337	LOC_Os03g44840	choline transporter-related, putative, expressed	The expression level of H21 was between T44 and T45
MSTRG.25696	LOC_Os07g31270	cupin 2, conserved barrel domain protein, putative, expressed	The expression level of H21 was between T44 and T45
MSTRG.748	LOC_Os01g12190	expressed protein	The expression level of H21 was between T44 and T45
MSTRG.21660	LOC_Os05g48580	expressed protein	The expression level of H21 was between T44 and T45
MSTRG.9967	LOC_Os02g05310	splicing factor 3B subunit 1, putative, expressed	The expression level of H21 was between T44 and T45
MSTRG.5280	LOC_Os10g37160	transposon protein, putative, unclassified, expressed	The expression level of H21 was between T44 and T45
MSTRG.23487	LOC_Os06g36390	expressed protein	The expression level of H21 was between T44 and T45
MSTRG.1977	LOC_Os01g42160		The expression level of H21 was between T44 and T45
MSTRG.30088	LOC_Os09g31130	citrate transporter, putative, expressed	The expression level of H21 was between T44 and T45
MSTRG.12171	LOC_Os02g47400	pectinacetylesterase domain containing protein, expressed	The expression level of H21 was between T44 and T45
MSTRG.8450	LOC_Os12g19381	ribulose bisphosphate carboxylase small chain, chloroplast precursor, putative, expressed	The expression level of H21 was between T44 and T45

MSTRG.24422	LOC_Os06g50520; LOC_Os06g50530	expressed protein	The expression level of H21 was between T44 and T45
MSTRG.17926	LOC_Os04g37520	extracellular ligand-gated ion channel, putative, expressed	The expression level of H21 was between T44 and T45
MSTRG.23874	LOC_Os06g43620	haemolysin-III, putative, expressed	The expression level of H21 was between T44 and T45
MSTRG.43	LOC_Os01g01670	expressed protein	The expression level of H21 was between T44 and T45
MSTRG.22307	LOC_Os06g07100	RING-H2 finger protein, putative, expressed	The expression level of H21 was between T44 and T45
MSTRG.25807	LOC_Os07g33410; LOC_Os07g33390	BCS1 protein, putative, expressed;ATPase, AAA family protein, expressed	The expression level of H21 was between T44 and T45
MSTRG.16621	LOC_Os04g01130	chromatin modification-related protein EAF3, putative, expressed	The expression level of H21 was between T44 and T45
MSTRG.27965			The expression level of H21 was between T44 and T45
MSTRG.20610	LOC_Os05g30310	transposon protein, putative, unclassified, expressed	The expression level of H21 was between T44 and T45
MSTRG.20005	LOC_Os05g11320	metallothionein-like protein 3B, putative, expressed	The expression level of H21 was between T44 and T45
MSTRG.5233	LOC_Os10g36050	hypothetical protein	The expression level of H21 was between T44 and T45
MSTRG.17763	LOC_Os04g34410	serine/threonine-protein kinase receptor precursor, putative, expressed	The expression level of H21 was between T44 and T45
MSTRG.14268	LOC_Os03g18779	expressed protein	The expression level of H21 was between T44 and T45
MSTRG.25007	LOC_Os07g09970	LTPL84 - Protease inhibitor/seed storage/LTP family protein precursor, expressed	The expression level of H21 was between T44 and T45
MSTRG.26011	LOC_Os07g37320	transporter family protein, putative, expressed	The expression level of H21 was between T44 and T45
MSTRG.752	LOC_Os01g12250	expressed protein	The expression level of H21 was between T44 and T45
MSTRG.19989	LOC_Os05g10910; LOC_Os05g10900	plant protein of unknown function domain containing protein, expressed;expressed protein	The expression level of H21 was between T44 and T45
MSTRG.12100	LOC_Os02g46473	expressed protein	The expression level of H21 was between T44 and T45
MSTRG.15587	LOC_Os03g49200	WD domain, G-beta repeat domain containing protein, expressed	The expression level of H21 was between T44 and T45
MSTRG.20567	LOC_Os05g29010	POLA4 - Putative DNA polymerase alpha complex subunit, expressed	The expression level of H21 was between T44 and T45
MSTRG.20978	LOC_Os05g37060	MYB family transcription factor, putative, expressed	The expression level of H21 was between T44 and T45
MSTRG.7677	LOC_Os12g02300	LTPL26 - Protease inhibitor/seed storage/LTP family protein precursor, expressed	The expression level of H21 was between T44 and T45
MSTRG.9592	LOC_Os02g01150	erythronate-4-phosphate dehydrogenase domain containing protein, expressed	The expression level of H21 was between T44 and T45
MSTRG.10843	LOC_Os02g21700	STE_MEKK_ste11_MAP3K.8 - STE kinases include homologs to sterile 7, sterile 11 and sterile 20 from yeast, expressed	The expression level of H21 was between T44 and T45
MSTRG.12392	LOC_Os02g50560	DSHCT domain containing protein, expressed	The expression level of H21 was between T44 and T45
MSTRG.29444	LOC_Os09g16000; LOC_Os09g16010	BURP domain containing protein, expressed	The expression level of H21 was between T44 and T45
MSTRG.21789	LOC_Os05g50310		The expression level of H21 was between T44 and T45
MSTRG.5165	LOC_Os10g35530	hydrolase, alpha/beta fold family domain containing protein, expressed	The expression level of H21 was between T44 and T45
MSTRG.270	LOC_Os01g03730	nuclease PA3, putative, expressed	The expression level of H21 was between T44 and T45
MSTRG.14178			The expression level of H21 was between T44 and T45
MSTRG.15270	LOC_Os03g43540	expressed protein	The expression level of H21 was between T44 and T45
MSTRG.24961	LOC_Os07g09150	expressed protein	The expression level of H21 was between T44 and T45
MSTRG.1975	LOC_Os01g42170		The expression level of H21 was between T44 and T45
MSTRG.12306	LOC_Os02g49410	histone-like transcription factor and archaeal histone, putative, expressed	The expression level of H21 was between T44 and T45
MSTRG.22450	LOC_Os06g09320	expressed protein	The expression level of H21 was between T44 and T45
MSTRG.11687	LOC_Os02g39480	protein phosphatase 2C, putative, expressed	The expression level of H21 was between T44 and T45
MSTRG.10218	LOC_Os02g09410	cytochrome P450, putative, expressed	The expression level of H21 was between T44 and T45
MSTRG.17612	LOC_Os04g32180	expressed protein	The expression level of H21 was between T44 and T45
MSTRG.29840	LOC_Os09g26620	auxin-repressed protein, putative, expressed	The expression level of H21 was between T44 and T45
MSTRG.22654	LOC_Os06g12740	expressed protein	The expression level of H21 was between T44 and T45
MSTRG.22360	LOC_Os06g07923	expressed protein	The expression level of H21 was between T44 and T45
MSTRG.1782	LOC_Os01g38520	expressed protein	The expression level of H21 was between T44 and T45
MSTRG.5410	LOC_Os10g39500	expressed protein	The expression level of H21 was between T44 and T45
MSTRG.24722	LOC_Os07g05440	retrotransposon protein, putative, unclassified, expressed	The expression level of H21 was between T44 and T45
MSTRG.17276	LOC_Os04g23440		The expression level of H21 was between T44 and T45
MSTRG.9318	LOC_Os12g40310	OsFBX458 - F-box domain containing protein, expressed	The expression level of H21 was between T44 and T45
MSTRG.25895	LOC_Os07g35370	TKL_IRAK_DUF26-lc.15 - DUF26 kinases have homology to DUF26 containing loci, expressed	The expression level of H21 was between T44 and T45
MSTRG.25003	LOC_Os07g09890	hexokinase, putative, expressed	The expression level of H21 was between T44 and T45
MSTRG.1284	LOC_Os01g22980	OsSCP3 - Putative Serine Carboxypeptidase homologue, expressed	The expression level of H21 was between T44 and T45
MSTRG.5413	LOC_Os10g39540	protein phosphatase 2C, putative, expressed	The expression level of H21 was between T44 and T45

MSTRG.26260	LOC_Os07g41220	peptidase aspartic family protein, putative, expressed	The expression level of H21 was between T44 and T45
MSTRG.19566	LOC_Os05g03150	expressed protein	The expression level of H21 was between T44 and T45
MSTRG.10822	LOC_Os02g21230	F-box domain containing protein, expressed	The expression level of H21 was between T44 and T45
MSTRG.6233	LOC_Os11g10370	OsFBX418 - F-box domain containing protein, expressed	The expression level of H21 was between T44 and T45
MSTRG.24974	LOC_Os07g09480	ATPase, putative, expressed	The expression level of H21 was between T44 and T45
MSTRG.11097	LOC_Os02g28870	U-box domain-containing protein, putative, expressed	The expression level of H21 was between T44 and T45
MSTRG.20080	LOC_Os05g13810	expressed protein	The expression level of H21 was between T44 and T45
MSTRG.5310	LOC_Os10g37660	trehalase precursor, putative, expressed	The expression level of H21 was between T44 and T45
MSTRG.21849	LOC_Os05g51130	mitochondrial chaperone BCS1, putative, expressed	The expression level of H21 was between T44 and T45
MSTRG.11879	LOC_Os02g42950	YABBY domain containing protein, putative, expressed	The expression level of H21 was between T44 and T45
MSTRG.10757	LOC_Os02g19820		The expression level of H21 was between T44 and T45
MSTRG.26432	LOC_Os07g44180	OsRCI2-10 - Hydrophobic protein LTI6A, expressed	The expression level of H21 was between T44 and T45
MSTRG.10513	LOC_Os02g13970	Complex I intermediate-associated protein 30 domain containing protein, putative, expressed	The expression level of H21 was between T44 and T45
MSTRG.8867	LOC_Os12g32480	expressed protein	The expression level of H21 was between T44 and T45
MSTRG.17440	LOC_Os04g28840		The expression level of H21 was between T44 and T45
MSTRG.6850	LOC_Os11g32490	expressed protein	The expression level of H21 was between T44 and T45
MSTRG.5277	LOC_Os10g37060	T-complex protein, putative, expressed	The expression level of H21 was between T44 and T45
MSTRG.26903	LOC_Os08g02580	expressed protein	The expression level of H21 was between T44 and T45
MSTRG.13858	LOC_Os03g12890	aminotransferase domain containing protein, putative, expressed	The expression level of H21 was between T44 and T45
MSTRG.14454	LOC_Os03g21590	expressed protein	The expression level of H21 was between T44 and T45
MSTRG.11591	LOC_Os02g38190	expressed protein	The expression level of H21 was between T44 and T45
MSTRG.12493	LOC_Os02g52210	zinc finger, C3HC4 type domain containing protein, expressed	The expression level of H21 was between T44 and T45
MSTRG.8990	LOC_Os12g35030		The expression level of H21 was between T44 and T45
LOC_Os03g39710	LOC_Os03g39710	transporter family protein, putative, expressed	The expression level of H21 was between T44 and T45
MSTRG.24183	LOC_Os06g46940	Os6bglu25 - beta-glucosidase homologue, similar to Os3bglu6, expressed	The expression level of H21 was between T44 and T45
LOC_Os02g37690	LOC_Os02g37690	UDP-glucoronosyl and UDP-glucosyl transferase, putative, expressed	The expression level of H21 was between T44 and T45
MSTRG.7975	LOC_Os12g07150	amidase family protein, putative, expressed	The expression level of H21 was between T44 and T45
MSTRG.13854	LOC_Os03g12860	homeobox associated leucine zipper, putative, expressed	The expression level of H21 was between T44 and T45
MSTRG.2910	LOC_Os01g56880	purple acid phosphatase precursor, putative, expressed	The expression level of H21 was between T44 and T45
MSTRG.12788	LOC_Os02g56370	OsWAK20 - OsWAK receptor-like protein kinase, expressed	The expression level of H21 was between T44 and T45
MSTRG.18737	LOC_Os04g49910	retrotransposon protein, putative, unclassified, expressed	The expression level of H21 was between T44 and T45
MSTRG.11041	LOC_Os02g27400	OsFBX49 - F-box domain containing protein, expressed	The expression level of H21 was between T44 and T45
MSTRG.2424	LOC_Os01g49910	expressed protein	The expression level of H21 was between T44 and T45
MSTRG.9487	LOC_Os12g42700	expressed protein	The expression level of H21 was between T44 and T45
MSTRG.24911	LOC_Os07g08400	GTP binding protein, putative, expressed	The expression level of H21 was between T44 and T45
MSTRG.17048	LOC_Os04g16080	transposon protein, putative, CACTA, En/Spm sub-class, expressed	The expression level of H21 was between T44 and T45
MSTRG.2276	LOC_Os01g47670		The expression level of H21 was between T44 and T45
MSTRG.1880	LOC_Os01g40710	high light inducible protein, putative, expressed	The expression level of H21 was between T44 and T45
MSTRG.18237	LOC_Os04g41960	NADP-dependent oxidoreductase, putative, expressed	The expression level of H21 was between T44 and T45
MSTRG.4655	LOC_Os10g22980	leucine rich repeat domain containing protein, putative, expressed	The expression level of H21 was between T44 and T45
MSTRG.22326	LOC_Os06g07420	retrotransposon protein, putative, unclassified, expressed	The expression level of H21 was between T44 and T45
MSTRG.7667	LOC_Os12g02150	expressed protein	The expression level of H21 was between T44 and T45
MSTRG.17072			The expression level of H21 was between T44 and T45
MSTRG.21738	LOC_Os05g49630	expressed protein	The expression level of H21 was between T44 and T45
LOC_Os12g34300	LOC_Os12g34300	OsFBX454 - F-box domain containing protein, expressed	The expression level of H21 was between T44 and T45
MSTRG.12780	LOC_Os02g56260	expressed protein	The expression level of H21 was between T44 and T45
MSTRG.5656	LOC_Os10g42930	expressed protein	The expression level of H21 was between T44 and T45
MSTRG.3543	LOC_Os01g66490	no apical meristem protein, putative, expressed	The expression level of H21 was between T44 and T45
MSTRG.616	LOC_Os01g09940		The expression level of H21 was between T44 and T45
MSTRG.24190	LOC_Os06g47200	LTPL85 - Protease inhibitor/seed storage/LTP family protein precursor, expressed	The expression level of H21 was between T44 and T45
MSTRG.11551	LOC_Os02g37830	protein kinase domain containing protein, expressed	The expression level of H21 was between T44 and T45
MSTRG.982	LOC_Os01g15970	expressed protein	The expression level of H21 was between T44 and T45
MSTRG.14416	LOC_Os03g20949	phospholipid-transporting ATPase, putative, expressed	The expression level of H21 was between T44 and T45

MSTRG.23021	LOC_Os06g21890	basic proline-rich protein precursor, putative, expressed	The expression level of H21 was between T44 and T45
MSTRG.23290	LOC_Os06g29700		The expression level of H21 was between T44 and T45
MSTRG.26753			The expression level of H21 was between T44 and T45
MSTRG.27764	LOC_Os08g22590; LOC_Os08g22600	retrotransposon protein, putative, unclassified, expressed;retrotransposon, putative, centromere-specific	The expression level of H21 was between T44 and T45
MSTRG.12535			The expression level of H21 was between T44 and T45
MSTRG.13867	LOC_Os03g13050	E2F-related protein, putative, expressed	The expression level of H21 was between T44 and T45
MSTRG.29316	LOC_Os09g12290	bifunctional aspartokinase/homoserine dehydrogenase, chloroplast precursor, putative, expressed	The expression level of H21 was between T44 and T45
MSTRG.13613	LOC_Os03g09970	sulfate transporter, putative, expressed	The expression level of H21 was between T44 and T45
MSTRG.6680	LOC_Os11g26790	dehydrin, putative, expressed	The expression level of H21 was between T44 and T45
MSTRG.16088			The expression level of H21 was between T44 and T45
MSTRG.22262	LOC_Os06g06470	U-box domain containing heat shock protein, putative, expressed	The expression level of H21 was between T44 and T45
MSTRG.14199	LOC_Os03g17860	OsPDIL5-1 protein disulfide isomerase PDIL5-1, expressed	The expression level of H21 was between T44 and T45
MSTRG.22923	LOC_Os06g19480	expressed protein	The expression level of H21 was between T44 and T45
MSTRG.17849	LOC_Os04g35550		The expression level of H21 was between T44 and T45
MSTRG.8055	LOC_Os12g08564	retrotransposon protein, putative, unclassified, expressed	The expression level of H21 was between T44 and T45
MSTRG.20554	LOC_Os05g28830	PMR5, putative, expressed	The expression level of H21 was between T44 and T45
MSTRG.3925	LOC_Os01g72650	RNA recognition motif containing protein, putative, expressed	The expression level of H21 was between T44 and T45
MSTRG.15615	LOC_Os03g49630	expressed protein	The expression level of H21 was between T44 and T45
MSTRG.3649	LOC_Os01g68060	copine, putative, expressed	The expression level of H21 was between T44 and T45
MSTRG.786	LOC_Os01g12710	oxidoreductase, short chain dehydrogenase/reductase family domain containing protein, expressed	The expression level of H21 was between T44 and T45
MSTRG.17397	LOC_Os04g27810		The expression level of H21 was between T44 and T45
MSTRG.17744	LOC_Os04g34170	retrotransposon protein, putative, unclassified, expressed	The expression level of H21 was between T44 and T45
MSTRG.21228			The expression level of H21 was between T44 and T45
MSTRG.18365	LOC_Os04g44050		The expression level of H21 was between T44 and T45
MSTRG.516	LOC_Os01g08380	transferase family protein, putative, expressed	The expression level of H21 was between T44 and T45
MSTRG.7579	LOC_Os11g47920		The expression level of H21 was between T44 and T45
MSTRG.1641	LOC_Os01g34870	expressed protein	The expression level of H21 was between T44 and T45
MSTRG.27834	LOC_Os08g24160		The expression level of H21 was between T44 and T45
MSTRG.10682	LOC_Os02g18460	NAM, putative, expressed	The expression level of H21 was between T44 and T45
MSTRG.26869	LOC_Os08g02120	kinase, pfkB family, putative, expressed	The expression level of H21 was between T44 and T45
MSTRG.16485	LOC_Os03g62430	OsWAK28 - OsWAK receptor-like protein kinase, expressed	The expression level of H21 was between T44 and T45
MSTRG.3477	LOC_Os01g65630	expressed protein	The expression level of H21 was between T44 and T45
MSTRG.13934	LOC_Os03g14080	transmembrane amino acid transporter protein, putative, expressed	The expression level of H21 was between T44 and T45
MSTRG.16189	LOC_Os03g58300	indole-3-glycerol phosphate lyase, chloroplast precursor, putative, expressed	The expression level of H21 was between T44 and T45
LOC_Os01g25740	LOC_Os01g25740	powdery mildew resistance protein PM3F, putative, expressed	The expression level of H21 was between T44 and T45
MSTRG.284	LOC_Os01g04670	expressed protein	The expression level of H21 was between T44 and T45
MSTRG.11101	LOC_Os02g28970	expressed protein	The expression level of H21 was between T44 and T45
MSTRG.22591	LOC_Os06g11800	annexin, putative, expressed	The expression level of H21 was between T44 and T45
MSTRG.20393	LOC_Os05g25450	TKL_IRAK_CrRLK1L-1.3 - The CrRLK1L-1 subfamily has homology to the CrRLK1L homolog, expressed	The expression level of H21 was between T44 and T45
MSTRG.24997	LOC_Os07g09760	retrotransposon protein, putative, unclassified, expressed	The expression level of H21 was between T44 and T45
MSTRG.24830	LOC_Os07g07310	polynucleotide phosphorylase, putative, expressed	The expression level of H21 was between T44 and T45
MSTRG.2528	LOC_Os01g51420	calcineurin B, putative, expressed	The expression level of H21 was between T44 and T45
MSTRG.17255			The expression level of H21 was between T44 and T45
MSTRG.18203	LOC_Os04g41340	4-nitrophenylphosphatase, putative, expressed	The expression level of H21 was between T44 and T45
MSTRG.15952	LOC_Os03g55220	bHelix-loop-helix transcription factor, putative, expressed	The expression level of H21 was between T44 and T45
MSTRG.9975	LOC_Os02g05410	splicing factor 3B subunit 1, putative, expressed	The expression level of H21 was between T44 and T45
MSTRG.1254	LOC_Os01g22209	expressed protein	The expression level of H21 was between T44 and T45
MSTRG.4611	LOC_Os10g22164	transposon protein, putative, unclassified, expressed	The expression level of H21 was between T44 and T45
MSTRG.1263			The expression level of H21 was between T44 and T45
MSTRG.2065	LOC_Os01g43400		The expression level of H21 was between T44 and T45
MSTRG.17641	LOC_Os04g32590	transcription factor, putative, expressed	The expression level of H21 was between T44 and T45
MSTRG.18893	LOC_Os04g52550	PAZ domain-containing protein, putative, expressed	The expression level of H21 was between T44 and T45
MSTRG.21178	LOC_Os05g40630	DUF567 domain containing protein, putative, expressed	The expression level of H21 was between T44 and T45
MSTRG.7007	LOC_Os11g36719		The expression level of H21 was between T44 and T45
MSTRG.14205	LOC_Os03g17950	expressed protein	The expression level of H21 was between T44 and T45

MSTRG.6083	LOC_Os11g07430	proteophosphoglycan ppg1, putative, expressed	The expression level of H21 was between T44 and T45
MSTRG.20025	LOC_Os05g11810	gibberellin 2-beta-dioxygenase 1, putative, expressed	The expression level of H21 was between T44 and T45
MSTRG.18239	LOC_Os04g41970	endoglucanase, putative, expressed	The expression level of H21 was between T44 and T45
MSTRG.5282	LOC_Os10g37190	protein kinase domain containing protein, expressed	The expression level of H21 was between T44 and T45
MSTRG.11762	LOC_Os02g40700	enzyme of the cupin superfamily protein, putative, expressed	The expression level of H21 was between T44 and T45
MSTRG.30270	LOC_Os09g34230	UDP-glucoronosyl/UDP-glucosyl transferase, putative, expressed	The expression level of H21 was between T44 and T45
MSTRG.2844	LOC_Os01g56100	BSD domain containing protein, expressed	The expression level of H21 was between T44 and T45
MSTRG.16451	LOC_Os03g61840	expressed protein	The expression level of H21 was between T44 and T45
MSTRG.28890	LOC_Os08g44050	ZOS8-12 - C2H2 zinc finger protein, expressed	The expression level of H21 was between T44 and T45
MSTRG.5854	LOC_Os11g03620; LOC_Os11g03630	expressed protein	The expression level of H21 was between T44 and T45
MSTRG.2437	LOC_Os01g50100	ABC transporter, ATP-binding protein, putative, expressed	The expression level of H21 was between T44 and T45
MSTRG.26284	LOC_Os07g41600	proline-rich protein, putative, expressed	The expression level of H21 was between T44 and T45
MSTRG.6374			The expression level of H21 was between T44 and T45
MSTRG.24235	LOC_Os06g47780	TKL_IRAK_DUF26-lh.3 - DUF26 kinases have homology to DUF26 containing loci, expressed	The expression level of H21 was between T44 and T45
MSTRG.15641	LOC_Os03g50090; LOC_Os03g50080	peptidyl-prolyl cis-trans isomerase, FKBP-type, putative, expressed;transposon protein, putative, CACTA, En/Spm sub-class, expressed	The expression level of H21 was between T44 and T45
MSTRG.13271	LOC_Os03g04930	expressed protein	The expression level of H21 was between T44 and T45
MSTRG.6301	LOC_Os11g11770	disease resistance protein RPM1, putative, expressed	The expression level of H21 was between T44 and T45
MSTRG.22534	LOC_Os06g10750	integral membrane protein DUF6 containing protein, expressed	The expression level of H21 was between T44 and T45
MSTRG.17995	LOC_Os04g38600	glyceraldehyde-3-phosphate dehydrogenase, putative, expressed	The expression level of H21 was between T44 and T45
MSTRG.20112	LOC_Os05g14360	expressed protein	The expression level of H21 was between T44 and T45
MSTRG.27300	LOC_Os08g10350	integral membrane family protein, putative, expressed	The expression level of H21 was between T44 and T45
MSTRG.25836	LOC_Os07g34006	transporter family protein, putative, expressed	The expression level of H21 was between T44 and T45
MSTRG.28509	LOC_Os08g38700	C3-BTB2 - Bric-a-Brac, Tramtrack, Broad Complex BTB domain with C3 subfamily conserved sequence, expressed	The expression level of H21 was between T44 and T45
MSTRG.409	LOC_Os01g06660	thiamine pyrophosphate enzyme, C-terminal TPP binding domain containing protein, expressed	The expression level of H21 was between T44 and T45
MSTRG.22802	LOC_Os06g16140	expressed protein	The expression level of H21 was between T44 and T45
MSTRG.10042	LOC_Os02g06430	DUF292 domain containing protein, expressed	The expression level of H21 was between T44 and T45
MSTRG.26920	LOC_Os08g02860	transposon protein, putative, unclassified, expressed	The expression level of H21 was between T44 and T45
LOC_Os11g36160	LOC_Os11g36160	receptor-like protein kinase 2 precursor, putative, expressed	The expression level of H21 was between T44 and T45
MSTRG.1640	LOC_Os01g34860	expressed protein	The expression level of H21 was between T44 and T45
MSTRG.26565	LOC_Os07g46330	uncharacterized UPF0114 domain containing protein, expressed	The expression level of H21 was between T44 and T45
MSTRG.30612	LOC_Os09g39940	plastocyanin-like domain containing protein, putative, expressed	The expression level of H21 was between T44 and T45
MSTRG.15417	LOC_Os03g46400	UDP-glucoronosyl and UDP-glucosyl transferase domain containing protein, expressed	The expression level of H21 was between T44 and T45
MSTRG.317	LOC_Os01g05090	expressed protein	The expression level of H21 was between T44 and T45
MSTRG.7332			The expression level of H21 was between T44 and T45
MSTRG.2817	LOC_Os01g55700	NLI interacting factor-like phosphatase, putative, expressed	The expression level of H21 was between T44 and T45
MSTRG.18500	LOC_Os04g46079	ELMO/CED-12 family protein, putative, expressed	The expression level of H21 was between T44 and T45
MSTRG.26458			The expression level of H21 was between T44 and T45
MSTRG.11935	LOC_Os02g43820	AP2 domain containing protein, expressed	The expression level of H21 was between T44 and T45
MSTRG.10588	LOC_Os02g15930	expressed protein	The expression level of H21 was between T44 and T45
MSTRG.20548	LOC_Os05g28740	universal stress protein domain containing protein, putative, expressed	The expression level of H21 was between T44 and T45
MSTRG.9736	LOC_Os02g02920	HVA22, putative, expressed	The expression level of H21 was between T44 and T45
MSTRG.13108	LOC_Os03g02570	dihydrodipicolinate reductase, putative, expressed	The expression level of H21 was between T44 and T45
MSTRG.13206	LOC_Os03g03910	catalase domain containing protein, expressed	The expression level of H21 was between T44 and T45
MSTRG.30392	LOC_Os09g36750	L-ascorbate peroxidase 4, putative, expressed	The expression level of H21 was between T44 and T45
MSTRG.21632			The expression level of H21 was between T44 and T45
MSTRG.10056	LOC_Os02g06580	formin, putative, expressed	The expression level of H21 was between T44 and T45

LOC_Os02g13510	LOC_Os02g13510	receptor-like protein kinase 5 precursor, putative, expressed	The expression level of H21 was between T44 and T45
MSTRG.13657	LOC_Os03g10410; LOC_Os03g10430	expressed protein	The expression level of H21 was between T44 and T45
MSTRG.12728	LOC_Os02g55570	shugoshin-1, putative, expressed	The expression level of H21 was between T44 and T45
MSTRG.511	LOC_Os01g08310	serine/threonine protein phosphatase 2A 59 kDa regulatory subunit Bgamma isoform, putative, expressed	The expression level of H21 was between T44 and T45
MSTRG.5553	LOC_Os10g41480	phospho-2-dehydro-3-deoxyheptonate aldolase, chloroplast precursor, putative, expressed	The expression level of H21 was between T44 and T45
MSTRG.28164	LOC_Os08g32340	expressed protein	The expression level of H21 was between T44 and T45
MSTRG.27648	LOC_Os08g18060	expressed protein	The expression level of H21 was between T44 and T45
MSTRG.21246	LOC_Os05g41620	expressed protein	The expression level of H21 was between T44 and T45
MSTRG.6733	LOC_Os11g29190	40S ribosomal protein S5, putative, expressed	The expression level of H21 was between T44 and T45
MSTRG.14064	LOC_Os03g16050	fructose-1,6-bisphosphatase, putative, expressed	The expression level of H21 was between T44 and T45
MSTRG.14873	LOC_Os03g31044	expressed protein	The expression level of H21 was between T44 and T45
MSTRG.16424	LOC_Os03g61530	invertase/pectin methylesterase inhibitor family protein, putative, expressed	The expression level of H21 was between T44 and T45
MSTRG.7566	LOC_Os11g47770	selT/selW/selH selenoprotein domain containing protein, expressed	The expression level of H21 was between T44 and T45
MSTRG.18732	LOC_Os04g49950	OsFBX150 - F-box domain containing protein, expressed	The expression level of H21 was between T44 and T45
MSTRG.16086	LOC_Os03g56974		The expression level of H21 was between T44 and T45
MSTRG.20823	LOC_Os05g33920		The expression level of H21 was between T44 and T45
MSTRG.7553	LOC_Os11g47600	glycosyl hydrolase, putative, expressed	The expression level of H21 was between T44 and T45
MSTRG.25852	LOC_Os07g33910	transporter family protein, putative, expressed	The expression level of H21 was between T44 and T45
MSTRG.11920	LOC_Os02g43660	plastocyanin-like domain containing protein, putative, expressed	The expression level of H21 was between T44 and T45
MSTRG.11568	LOC_Os02g37890	retrotransposon protein, putative, unclassified, expressed	The expression level of H21 was between T44 and T45
MSTRG.20811	LOC_Os05g33680		The expression level of H21 was between T44 and T45
MSTRG.21957	LOC_Os06g02000	adenylate kinase, putative, expressed	The expression level of H21 was between T44 and T45
MSTRG.26500	LOC_Os07g45370	expressed protein	The expression level of H21 was between T44 and T45
MSTRG.27860	LOC_Os08g25010	TBC domain containing protein, expressed	The expression level of H21 was between T44 and T45
MSTRG.5320	LOC_Os10g37860	expressed protein	The expression level of H21 was between T44 and T45
MSTRG.18299	LOC_Os04g42770	expressed protein	The expression level of H21 was between T44 and T45
MSTRG.3443	LOC_Os01g65130	peptide transporter, putative, expressed	The expression level of H21 was between T44 and T45
MSTRG.28549			The expression level of H21 was between T44 and T45
MSTRG.5390	LOC_Os10g39100	uncharacterized Cys-rich domain containing protein, putative, expressed	The expression level of H21 was between T44 and T45
MSTRG.28368	LOC_Os08g35700	Leucine Rich Repeat family protein, expressed	The expression level of H21 was between T44 and T45
MSTRG.12824	LOC_Os02g56880	transcriptional corepressor LEUNIG, putative, expressed	The expression level of H21 was between T44 and T45
MSTRG.2566	LOC_Os01g52230	phosphoethanolamine/phosphocholine phosphatase, putative, expressed	The expression level of H21 was between T44 and T45
MSTRG.28853	LOC_Os08g43680	glutathione S-transferase, C-terminal domain containing protein, expressed	The expression level of H21 was between T44 and T45
MSTRG.458			The expression level of H21 was between T44 and T45
MSTRG.2965	LOC_Os01g57610	OsGH3.1 - Probable indole-3-acetic acid-amido synthetase, expressed	The expression level of H21 was between T44 and T45
MSTRG.7957	LOC_Os12g06820	PE repeat family protein, putative, expressed	The expression level of H21 was between T44 and T45
MSTRG.27538	LOC_Os08g15010; LOC_Os08g15000	pentatricopeptide repeat domain containing protein, putative, expressed	The expression level of H21 was between T44 and T45
MSTRG.15878	LOC_Os03g53710	aldose 1-epimerase, putative, expressed	The expression level of H21 was between T44 and T45
MSTRG.24606	LOC_Os07g03070	tetratricopeptide repeat containing protein, putative, expressed	The expression level of H21 was between T44 and T45
MSTRG.26189	LOC_Os07g39960	ZOS7-07 - C2H2 zinc finger protein, expressed	The expression level of H21 was between T44 and T45
MSTRG.4880	LOC_Os10g29680; LOC_Os10g29690		The expression level of H21 was between T44 and T45
MSTRG.7814	LOC_Os12g04260	astaxanthin synthase KC28, putative, expressed	The expression level of H21 was between T44 and T45
MSTRG.9685	LOC_Os02g02400	catalase isozyme A, putative, expressed	The expression level of H21 was between T44 and T45
MSTRG.23268	LOC_Os06g29260		The expression level of H21 was between T44 and T45
MSTRG.15	LOC_Os01g01295	expressed protein	The expression level of H21 was between T44 and T45
MSTRG.9874	LOC_Os02g04640	Myb-like DNA-binding domain containing protein, putative, expressed	The expression level of H21 was between T44 and T45
MSTRG.8028	LOC_Os12g07940	expressed protein	The expression level of H21 was between T44 and T45
MSTRG.16219	LOC_Os03g58764	OsFBDUF18 - F-box and DUF domain containing protein, expressed	The expression level of H21 was between T44 and T45
MSTRG.11967	LOC_Os02g44320	LTPL113 - Protease inhibitor/seed storage/LTP family protein precursor, expressed	The expression level of H21 was between T44 and T45
MSTRG.28726	LOC_Os08g41910	Sua5/YciO/YrdC/YwlC family protein, putative, expressed	The expression level of H21 was between T44 and T45
MSTRG.19240	LOC_Os04g57410		The expression level of H21 was between T44 and T45
MSTRG.22644	LOC_Os06g12620	expressed protein	The expression level of H21 was between T44 and T45

MSTRG.25167	LOC_Os07g12910	PHD finger protein, putative, expressed	The expression level of H21 was between T44 and T45
MSTRG.26850	LOC_Os08g01830	TKL_IRAK_CR4L.6 - The CR4L subfamily has homology with Crinkly4, expressed	The expression level of H21 was between T44 and T45
MSTRG.27238	LOC_Os08g09170	expressed protein	The expression level of H21 was between T44 and T45
MSTRG.7008	LOC_Os11g36740	DUF593 domain containing protein, expressed	The expression level of H21 was between T44 and T45
MSTRG.21773	LOC_Os05g50090	oxidoreductase, 2OG-FeII oxygenase domain containing protein, putative, expressed	The expression level of H21 was between T44 and T45
MSTRG.15665	LOC_Os03g50420	expressed protein	The expression level of H21 was between T44 and T45
MSTRG.25416	LOC_Os07g23120	expressed protein	The expression level of H21 was between T44 and T45
MSTRG.17421	LOC_Os04g28210	verticillium wilt disease resistance protein, putative, expressed	The expression level of H21 was between T44 and T45
MSTRG.5169	LOC_Os10g35290		The expression level of H21 was between T44 and T45
MSTRG.21417	LOC_Os05g44580	expressed protein	The expression level of H21 was between T44 and T45
MSTRG.30050	LOC_Os09g30270	transposon protein, putative, Mutator sub-class, expressed	The expression level of H21 was between T44 and T45
MSTRG.17236	LOC_Os04g21710	expressed protein	The expression level of H21 was between T44 and T45
MSTRG.18182	LOC_Os04g41090; LOC_Os04g41080		The expression level of H21 was between T44 and T45
MSTRG.8725	LOC_Os12g29330	no apical meristem protein, putative, expressed	The expression level of H21 was between T44 and T45
MSTRG.28148	LOC_Os08g31970	NHL repeat-containing protein, putative, expressed	The expression level of H21 was between T44 and T45
MSTRG.2974	LOC_Os01g57740	expressed protein	The expression level of H21 was between T44 and T45
MSTRG.15005	LOC_Os03g37250	retrotransposon protein, putative, Ty3-gypsy subclass, expressed	The expression level of H21 was between T44 and T45
MSTRG.22658	LOC_Os06g12830	expressed protein	The expression level of H21 was between T44 and T45
MSTRG.16974	LOC_Os04g14110; LOC_Os04g14190	TRAF-type zinc finger family protein, expressed	The expression level of H21 was between T44 and T45
MSTRG.25634	LOC_Os07g29600	zinc finger, C3HC4 type, domain containing protein, expressed	The expression level of H21 was between T44 and T45
MSTRG.4396	LOC_Os10g11980	transferase family protein, putative, expressed	The expression level of H21 was between T44 and T45
MSTRG.28083	LOC_Os08g30780	ABC transporter, ATP-binding protein, putative, expressed	The expression level of H21 was between T44 and T45

Table S8. Overview of raw data from degradome sequencing.

Items	Number	Ratio
Raw Reads	29137433	/
reads < 15nt after removing 3 adaptor	140962	0.48%
Mappable Reads	28996471	99.52%
Unique Raw Reads	6768733	/
Unique reads < 15nt after removing 3 adaptor	39242	0.58%
Unique Mappable Reads	6729491	99.42%
Transcript Mapped Reads	26070347	89.47%
Unique Transcript Mapped Reads	5399372	79.77%
Number of input Transcript	103427	/
Number of Coverd Transcript	81229	78.54%

Table S9. The results of the targets were identified with degradome analysis in anthers of H21, T44, and T45.

ID	SmallRNA	SmallRNA_seq	Transcript	Alignment Score	Alignment Range	Degradome Cleavage Site	Degradome Category	Degradome Read Number Raw	Degradome Read Number Normalized
1	aly-miR157a-5p	TTGACAGAAGATAGAGAGCAC	LOC_Os08g39890.1	2	992-1012	1003	0	4	2
2	aly-miR157a-5p	TTGACAGAAGATAGAGAGCAC	MSTRG.28596.1	2	1089-1109	1100	0	4	2
3	aly-miR157a-5p	TTGACAGAAGATAGAGAGCAC	LOC_Os08g41940.1	2.5	1054-1074	1065	2	2	2
4	aly-miR157a-5p	TTGACAGAAGATAGAGAGCAC	LOC_Os02g04680.2	3	1962-1982	1973	2	9	0.9
5	aly-miR157a-5p	TTGACAGAAGATAGAGAGCAC	MSTRG.9878.8	3	1960-1980	1971	2	9	0.9
6	aly-miR157a-5p	TTGACAGAAGATAGAGAGCAC	LOC_Os02g04680.1	3	1965-1985	1976	2	9	0.9
7	aly-miR157a-5p	TTGACAGAAGATAGAGAGCAC	MSTRG.9878.7	3	1972-1992	1983	2	9	0.9
8	aly-miR157a-5p	TTGACAGAAGATAGAGAGCAC	MSTRG.9878.6	3	2154-2174	2165	2	9	0.9
9	aly-miR157a-5p	TTGACAGAAGATAGAGAGCAC	MSTRG.9878.5	3	2280-2300	2291	2	9	0.9
10	aly-miR157a-5p	TTGACAGAAGATAGAGAGCAC	MSTRG.9878.4	3	2512-2532	2523	2	9	0.9
11	aly-miR157a-5p	TTGACAGAAGATAGAGAGCAC	MSTRG.9878.1	3	2580-2600	2591	2	9	0.9
12	aly-miR157a-5p	TTGACAGAAGATAGAGAGCAC	MSTRG.9878.3	3	2603-2623	2614	2	9	0.9
13	aly-miR157a-5p	TTGACAGAAGATAGAGAGCAC	MSTRG.9878.2	3	3038-3058	3049	2	9	0.9
14	aly-miR157a-5p	TTGACAGAAGATAGAGAGCAC	LOC_Os06g49010.3	2	1439-1459	1450	2	7	0.7
15	aly-miR157a-5p	TTGACAGAAGATAGAGAGCAC	LOC_Os06g49010.2	2	1494-1514	1505	2	7	0.7
16	aly-miR157a-5p	TTGACAGAAGATAGAGAGCAC	LOC_Os06g49010.4	2	1638-1658	1649	2	9	0.9
17	aly-miR157a-5p	TTGACAGAAGATAGAGAGCAC	LOC_Os06g49010.1	2	1686-1706	1697	2	9	0.9
18	aly-miR157a-5p	TTGACAGAAGATAGAGAGCAC	MSTRG.24320.4	2	1729-1749	1740	2	8	0.8
19	aly-miR157a-5p	TTGACAGAAGATAGAGAGCAC	MSTRG.24320.6	2	1830-1850	1841	2	8	0.8
20	aly-miR157a-5p	TTGACAGAAGATAGAGAGCAC	LOC_Os06g49010.6	2	1842-1862	1853	2	8	0.8
21	aly-miR157a-5p	TTGACAGAAGATAGAGAGCAC	MSTRG.24320.18	2	1939-1959	1950	2	6	0.6
22	aly-miR157a-5p	TTGACAGAAGATAGAGAGCAC	MSTRG.24320.21	2	2025-2045	2036	2	7	0.7
23	aly-miR157a-5p	TTGACAGAAGATAGAGAGCAC	LOC_Os06g49010.5	2	2018-2038	2029	2	10	1
24	aly-miR157a-5p	TTGACAGAAGATAGAGAGCAC	MSTRG.24320.23	2	2315-2335	2326	2	9	0.9
25	aly-miR157a-5p	TTGACAGAAGATAGAGAGCAC	MSTRG.24320.16	2	2279-2299	2290	2	6	0.6
26	aly-miR157a-5p	TTGACAGAAGATAGAGAGCAC	MSTRG.24320.14	2	2298-2318	2309	2	7	0.7
27	aly-miR157a-5p	TTGACAGAAGATAGAGAGCAC	MSTRG.24320.19	2	2413-2433	2424	2	9	0.9
28	aly-miR157a-5p	TTGACAGAAGATAGAGAGCAC	MSTRG.24320.5	2	2360-2380	2371	2	9	0.9
29	aly-miR157a-5p	TTGACAGAAGATAGAGAGCAC	MSTRG.24320.20	2	2684-2704	2695	2	9	0.9
30	aly-miR157a-5p	TTGACAGAAGATAGAGAGCAC	MSTRG.24320.24	2	2743-2763	2754	2	8	0.8
31	aly-miR157a-5p	TTGACAGAAGATAGAGAGCAC	MSTRG.24320.17	2	2673-2693	2684	2	9	0.9
32	aly-miR157a-5p	TTGACAGAAGATAGAGAGCAC	MSTRG.24320.22	2	2781-2801	2792	2	6	0.6
33	aly-miR157a-5p	TTGACAGAAGATAGAGAGCAC	MSTRG.24320.1	2	2717-2737	2728	2	8	0.8
34	aly-miR157a-5p	TTGACAGAAGATAGAGAGCAC	MSTRG.24320.15	2	2764-2784	2775	2	9	0.9
35	aly-miR157a-5p	TTGACAGAAGATAGAGAGCAC	MSTRG.24320.2	2	2768-2788	2779	2	8	0.8
36	aly-miR157a-5p	TTGACAGAAGATAGAGAGCAC	MSTRG.24320.3	2	2787-2807	2798	2	7	0.7
37	aly-miR157a-5p	TTGACAGAAGATAGAGAGCAC	MSTRG.24320.13	2	2864-2884	2875	2	7	0.7
38	aly-miR157a-5p	TTGACAGAAGATAGAGAGCAC	LOC_Os06g45310.1	2.5	854-874	865	4	1	1
39	aly-miR159b-3p_R+1	TTTGGATTGAAGGGAGCTCTTT	LOC_Os01g59660.1	4	1259-1280	1271	2	26	6.5
40	aly-miR159b-3p_R+1	TTTGGATTGAAGGGAGCTCTTT	LOC_Os01g59660.4	4	1179-1200	1191	2	26	6.5
41	aly-miR159b-3p_R+1	TTTGGATTGAAGGGAGCTCTTT	LOC_Os01g59660.3	4	1257-1278	1269	2	26	6.5
42	aly-miR159b-3p_R+1	TTTGGATTGAAGGGAGCTCTTT	LOC_Os01g59660.2	4	1347-1368	1359	2	26	6.5
43	ath-miR167a-5p_L+1	ATGAAGCTGCCAGCATGATCTA	LOC_Os09g39420.1	4	2909-2930	2921	4	1	1
44	ath-miR8175_L-2	TCCCCGGCAACGGCGCCA	LOC_Os02g21750.1	4	2361-2378	2369	2	6	3
45	ath-miR8175_L-2	TCCCCGGCAACGGCGCCA	MSTRG.10846.1	4	4189-4206	4197	2	6	3
46	ath-miR8175_L-2	TCCCCGGCAACGGCGCCA	LOC_Os04g51570.1	4	185-202	193	2	2	1
47	ath-miR8175_L-2	TCCCCGGCAACGGCGCCA	MSTRG.18810.1	4	193-210	201	2	2	1
48	ath-miR8175_L-2	TCCCCGGCAACGGCGCCA	LOC_Os05g07890.1	4	431-448	439	2	5	2.5
49	ath-miR8175_L-2	TCCCCGGCAACGGCGCCA	MSTRG.19856.1	4	630-647	638	2	5	2.5
50	ath-miR8175_L-2	TCCCCGGCAACGGCGCCA	MSTRG.5637.9	4	159-177	168	2	4	0.4
51	ath-miR8175_L-2	TCCCCGGCAACGGCGCCA	LOC_Os10g42710.1	4	196-214	205	2	4	0.4
52	ath-miR8175_L-2	TCCCCGGCAACGGCGCCA	LOC_Os10g42710.4	4	196-214	205	2	4	0.4
53	ath-miR8175_L-2	TCCCCGGCAACGGCGCCA	MSTRG.5637.8	4	230-248	239	2	4	0.4
54	ath-miR8175_L-2	TCCCCGGCAACGGCGCCA	LOC_Os10g42710.3	4	196-214	205	2	4	0.4
55	ath-miR8175_L-2	TCCCCGGCAACGGCGCCA	LOC_Os10g42710.5	4	196-214	205	2	4	0.4
56	ath-miR8175_L-2	TCCCCGGCAACGGCGCCA	LOC_Os10g42710.2	4	196-214	205	2	4	0.4

57	ath-miR8175_L-2	TCCCCGGCAACGGCGCCA	LOC_Os10g42710.6	4	196-214	205	2	4	0.4
58	ath-miR8175_L-2	TCCCCGGCAACGGCGCCA	LOC_Os10g42710.7	4	196-214	205	2	4	0.4
59	ath-miR8175_L-2	TCCCCGGCAACGGCGCCA	MSTRG.5637.1	4	154-172	163	2	4	0.4
60	ath-miR8175_L-2	TCCCCGGCAACGGCGCCA	MSTRG.9365.5	4	992-1009	1000	4	1	0.2
61	ath-miR8175_L-2	TCCCCGGCAACGGCGCCA	MSTRG.9365.1	4	1003-1020	1011	4	1	0.2
62	ath-miR8175_L-2	TCCCCGGCAACGGCGCCA	LOC_Os12g41140.2	4	1021-1038	1029	4	1	0.2
63	ath-miR8175_L-2	TCCCCGGCAACGGCGCCA	LOC_Os12g41140.3	4	1315-1332	1323	4	1	0.2
64	ath-miR8175_L-2	TCCCCGGCAACGGCGCCA	LOC_Os12g41140.1	4	1318-1335	1326	4	1	0.2
65	ath-miR8175_L-2	TCCCCGGCAACGGCGCCA	LOC_Os03g47970.2	3	336-353	344	4	1	0.2
66	ath-miR8175_L-2	TCCCCGGCAACGGCGCCA	LOC_Os03g47970.1	3	336-353	344	4	1	0.2
67	ath-miR8175_L-2	TCCCCGGCAACGGCGCCA	MSTRG.15513.4	3	206-223	214	4	1	0.2
68	ath-miR8175_L-2	TCCCCGGCAACGGCGCCA	MSTRG.15513.2	3	352-369	360	4	1	0.2
69	ath-miR8175_L-2	TCCCCGGCAACGGCGCCA	MSTRG.15513.1	3	537-554	545	4	1	0.2
70	ath-miR8175_L-2	TCCCCGGCAACGGCGCCA	LOC_Os11g42000.1	4	69-86	77	2	2	0.5
71	ath-miR8175_L-2	TCCCCGGCAACGGCGCCA	MSTRG.7334.2	4	73-90	81	2	2	0.5
72	ath-miR8175_L-2	TCCCCGGCAACGGCGCCA	MSTRG.7334.4	4	52-69	60	2	2	0.5
73	ath-miR8175_L-2	TCCCCGGCAACGGCGCCA	MSTRG.7334.1	4	81-98	89	2	2	0.5
74	bdi-miR167a_R+1	TGAAGCTGCCAGCATGATCTAT	MSTRG.19259.4	4	2532-2554	2544	2	5	0.714285714
75	bdi-miR167a_R+1	TGAAGCTGCCAGCATGATCTAT	MSTRG.19259.7	4	2616-2638	2628	2	5	0.714285714
76	bdi-miR167a_R+1	TGAAGCTGCCAGCATGATCTAT	MSTRG.19259.3	4	2633-2655	2645	2	5	0.714285714
77	bdi-miR167a_R+1	TGAAGCTGCCAGCATGATCTAT	MSTRG.19259.5	4	2638-2660	2650	2	5	0.714285714
78	bdi-miR167a_R+1	TGAAGCTGCCAGCATGATCTAT	LOC_Os04g57610.3	4	2645-2667	2657	2	5	0.714285714
79	bdi-miR167a_R+1	TGAAGCTGCCAGCATGATCTAT	LOC_Os04g57610.1	4	2657-2679	2669	2	5	0.714285714
80	bdi-miR167a_R+1	TGAAGCTGCCAGCATGATCTAT	MSTRG.19259.6	4	2725-2747	2737	2	5	0.714285714
81	bdi-miR167a_R+1	TGAAGCTGCCAGCATGATCTAT	LOC_Os07g33790.1	4	3419-3439	3430	2	2	0.666666667
82	bdi-miR167a_R+1	TGAAGCTGCCAGCATGATCTAT	MSTRG.25835.3	4	3370-3390	3381	2	2	0.666666667
83	bdi-miR167a_R+1	TGAAGCTGCCAGCATGATCTAT	MSTRG.25835.2	4	3396-3416	3407	2	2	0.666666667
84	bdi-miR167a_R+1	TGAAGCTGCCAGCATGATCTAT	LOC_Os09g39420.1	4	2908-2929	2920	2	8	8
85	bdi-MIR169f-p3	GGCAAGTTTGTCCTTGGCTAC	LOC_Os08g36450.1	3.5	1569-1589	1580	2	3	1.5
86	bdi-MIR169f-p3	GGCAAGTTTGTCCTTGGCTAC	MSTRG.28396.2	3.5	1127-1147	1138	2	3	1.5
87	bdi-MIR169f-p3	GGCAAGTTTGTCCTTGGCTAC	LOC_Os08g08080.2	4	1201-1220	1212	2	6	1.2
88	bdi-MIR169f-p3	GGCAAGTTTGTCCTTGGCTAC	LOC_Os08g08080.1	4	1216-1235	1227	2	6	1.2
89	bdi-MIR169f-p3	GGCAAGTTTGTCCTTGGCTAC	MSTRG.27206.5	4	1296-1315	1307	2	6	1.2
90	bdi-MIR169f-p3	GGCAAGTTTGTCCTTGGCTAC	MSTRG.27206.1	4	1440-1459	1451	2	6	1.2
91	bdi-MIR169f-p3	GGCAAGTTTGTCCTTGGCTAC	MSTRG.27206.2	4	1562-1581	1573	2	6	1.2
92	bdi-miR2118a	TTTCCGATGCCTCCCATTCCTA	MSTRG.11558.1	4	114-135	126	0	15	15
93	bdi-miR2118a	TTTCCGATGCCTCCCATTCCTA	MSTRG.17799.1	3.5	738-759	750	0	36	36
94	bdi-miR2118a	TTTCCGATGCCTCCCATTCCTA	MSTRG.29394.1	4	106-127	118	0	65	65
95	bdi-miR2118a	TTTCCGATGCCTCCCATTCCTA	MSTRG.6412.3	3	904-925	916	4	1	0.5
96	bdi-miR2118a	TTTCCGATGCCTCCCATTCCTA	MSTRG.6412.1	3	1140-1161	1152	4	1	0.5
97	bdi-miR2118a	TTTCCGATGCCTCCCATTCCTA	MSTRG.8957.1	2.5	145-166	157	0	6	6
98	bdi-miR2118a	TTTCCGATGCCTCCCATTCCTA	MSTRG.9247.1	3.5	196-217	208	0	101	101
99	bdi-miR2118a	TTTCCGATGCCTCCCATTCCTA	MSTRG.8777.1	4	106-127	118	4	1	1
100	bdi-miR2118a	TTTCCGATGCCTCCCATTCCTA	MSTRG.8657.1	4	137-158	149	1	16	16
101	bdi-miR2118a	TTTCCGATGCCTCCCATTCCTA	MSTRG.8960.1	4	178-199	190	0	14	14
102	bdi-miR2118a	TTTCCGATGCCTCCCATTCCTA	MSTRG.9266.1	4	286-307	298	0	84	84
103	bdi-miR2118a	TTTCCGATGCCTCCCATTCCTA	LOC_Os03g50150.1	4	1343-1364	1355	4	1	1
104	bdi-miR2118a	TTTCCGATGCCTCCCATTCCTA	MSTRG.23754.1	4	263-284	275	0	33	33
105	bdi-miR2275b	TTCAGTTTCTTCTAATATCTCA	LOC_Os02g52880.2	3.5	856-876	867	4	1	0.5
106	bdi-miR2275b	TTCAGTTTCTTCTAATATCTCA	LOC_Os02g52880.1	3.5	876-896	887	4	1	0.5
107	bdi-miR5054_1ss10TA	TCCCCACGGACGGCGCCA	LOC_Os02g35500.1	3.5	257-273	265	4	1	0.5
108	bdi-miR5054_1ss10TA	TCCCCACGGACGGCGCCA	MSTRG.11434.2	3.5	236-252	244	4	1	0.5
109	bdi-miR5054_1ss10TA	TCCCCACGGACGGCGCCA	LOC_Os02g13630.1	4	141-158	149	4	1	0.2
110	bdi-miR5054_1ss10TA	TCCCCACGGACGGCGCCA	MSTRG.10489.2	4	141-158	149	4	1	0.2
111	bdi-miR5054_1ss10TA	TCCCCACGGACGGCGCCA	MSTRG.10489.4	4	133-150	141	4	1	0.2
112	bdi-miR5054_1ss10TA	TCCCCACGGACGGCGCCA	MSTRG.10489.1	4	141-158	149	4	1	0.2
113	bdi-miR5054_1ss10TA	TCCCCACGGACGGCGCCA	MSTRG.10489.5	4	126-143	134	4	1	0.2
114	bdi-miR5054_1ss10TA	TCCCCACGGACGGCGCCA	LOC_Os07g04530.1	3.5	5110-5127	5118	2	3	3
115	bdi-miR5054_1ss10TA	TCCCCACGGACGGCGCCA	MSTRG.24614.1	4	2861-2878	2869	4	1	1

116	bdi-miR529-3p_L-1R+1	CTGTACCCTCTCTCTTCTTCT	LOC_Os08g44040.1	4	183-203	194	2	2	0.25
117	bdi-miR529-3p_L-1R+1	CTGTACCCTCTCTCTTCTTCT	LOC_Os10g29560.1	4	2692-2711	2703	4	1	0.125
118	bdi-miR529-3p_L-1R+1	CTGTACCCTCTCTCTTCTTCT	MSTRG.4876.5	4	2644-2663	2655	4	1	0.125
119	bdi-miR529-3p_L-1R+1	CTGTACCCTCTCTCTTCTTCT	MSTRG.4876.3	4	2698-2717	2709	4	1	0.125
120	bdi-miR529-3p_L-1R+1	CTGTACCCTCTCTCTTCTTCT	MSTRG.4876.2	4	2658-2677	2669	4	1	0.125
121	bdi-miR529-3p_L-1R+1	CTGTACCCTCTCTCTTCTTCT	MSTRG.4876.4	4	2656-2675	2667	4	1	0.125
122	bdi-miR529-3p_L-1R+1	CTGTACCCTCTCTCTTCTTCT	MSTRG.4876.1	4	2700-2719	2711	4	1	0.125
123	bdi-miR529-3p_L-1R+1	CTGTACCCTCTCTCTTCTTCT	MSTRG.4876.8	4	2280-2299	2291	4	1	0.125
124	bdi-MIR529-p5_1ss7GA	TAAAGGAAGAAGAGAGAGAGT	LOC_Os03g55990.3	4	764-784	775	4	1	0.5
125	cme-MIR171h-p3	TTGAGCCGCGTCAATATCTCTT	LOC_Os02g44360.1	1.5	1347-1368	1359	0	6	6
126	cme-MIR171h-p3	TTGAGCCGCGTCAATATCTCTT	MSTRG.11971.1	2.5	1405-1426	1417	0	8	4
127	cme-MIR171h-p3	TTGAGCCGCGTCAATATCTCTT	LOC_Os02g44370.1	2.5	1522-1543	1534	0	8	4
128	cme-MIR171h-p3	TTGAGCCGCGTCAATATCTCTT	LOC_Os04g46860.1	1.5	1322-1343	1334	0	62	62
129	cme-MIR171h-p3	TTGAGCCGCGTCAATATCTCTT	LOC_Os06g01620.1	2	452-473	464	1	2	1
130	cme-MIR171h-p3	TTGAGCCGCGTCAATATCTCTT	MSTRG.21932.1	2	709-730	721	2	2	1
131	cme-MIR171h-p3	TTGAGCCGCGTCAATATCTCTT	LOC_Os10g40390.1	2.5	164-185	176	0	35	17.5
132	cme-MIR171h-p3	TTGAGCCGCGTCAATATCTCTT	MSTRG.5478.2	2.5	1162-1183	1174	0	35	17.5
133	cme-miR172a	GGAATCTTGATGATGCTGCAG	MSTRG.4023.1	4	518-538	529	2	5	5
134	cme-miR172a	GGAATCTTGATGATGCTGCAG	LOC_Os04g55560.4	2	807-827	818	0	29	5.8
135	cme-miR172a	GGAATCTTGATGATGCTGCAG	LOC_Os04g55560.2	2	1623-1643	1634	0	29	5.8
136	cme-miR172a	GGAATCTTGATGATGCTGCAG	LOC_Os04g55560.3	2	1634-1654	1645	0	29	5.8
137	cme-miR172a	GGAATCTTGATGATGCTGCAG	MSTRG.19117.4	2	1909-1929	1920	0	29	5.8
138	cme-miR172a	GGAATCTTGATGATGCTGCAG	MSTRG.19117.1	2	2085-2105	2096	0	29	5.8
139	cme-miR172a	GGAATCTTGATGATGCTGCAG	LOC_Os05g03040.2	1.5	1552-1572	1563	0	15	5
140	cme-miR172a	GGAATCTTGATGATGCTGCAG	LOC_Os05g03040.1	1.5	1976-1996	1987	0	15	5
141	cme-miR172a	GGAATCTTGATGATGCTGCAG	LOC_Os05g03040.3	1.5	2089-2109	2100	0	15	5
142	cme-miR172a	GGAATCTTGATGATGCTGCAG	LOC_Os05g07070.2	4	1274-1294	1285	2	2	0.2
143	cme-miR172a	GGAATCTTGATGATGCTGCAG	LOC_Os05g07070.1	4	1274-1294	1285	2	2	0.2
144	cme-miR172a	GGAATCTTGATGATGCTGCAG	MSTRG.19816.2	4	1538-1558	1549	4	1	0.1
145	cme-miR172a	GGAATCTTGATGATGCTGCAG	LOC_Os05g07070.4	4	1601-1621	1612	2	2	0.2
146	cme-miR172a	GGAATCTTGATGATGCTGCAG	MSTRG.19816.10	4	1672-1692	1683	4	1	0.1
147	cme-miR172a	GGAATCTTGATGATGCTGCAG	LOC_Os05g07070.5	4	3942-3962	3953	2	2	0.2
148	cme-miR172a	GGAATCTTGATGATGCTGCAG	MSTRG.19816.4	4	3967-3987	3978	2	2	0.2
149	cme-miR172a	GGAATCTTGATGATGCTGCAG	MSTRG.19816.3	4	4014-4034	4025	4	1	0.1
150	cme-miR172a	GGAATCTTGATGATGCTGCAG	MSTRG.19816.1	4	4278-4298	4289	4	1	0.1
151	cme-miR172a	GGAATCTTGATGATGCTGCAG	LOC_Os05g07070.7	4	4362-4382	4373	2	2	0.2
152	cme-miR172a	GGAATCTTGATGATGCTGCAG	LOC_Os05g07070.8	4	5150-5170	5161	2	2	0.2
153	cme-miR172a	GGAATCTTGATGATGCTGCAG	LOC_Os05g07070.3	4	5590-5610	5601	2	2	0.2
154	cme-miR172a	GGAATCTTGATGATGCTGCAG	MSTRG.28588.2	4	2016-2036	2027	4	1	0.5
155	cme-miR172a	GGAATCTTGATGATGCTGCAG	LOC_Os08g39630.1	4	1868-1888	1879	4	1	0.5
156	cme-miR172a	GGAATCTTGATGATGCTGCAG	LOC_Os03g60430.1	1.5	1755-1775	1766	1	3	0.75
157	cme-miR172a	GGAATCTTGATGATGCTGCAG	LOC_Os03g60430.2	1.5	1767-1787	1778	1	3	0.75
158	cme-miR172a	GGAATCTTGATGATGCTGCAG	MSTRG.16339.2	1.5	1933-1953	1944	1	3	0.75
159	cme-miR172a	GGAATCTTGATGATGCTGCAG	MSTRG.16339.1	1.5	2040-2060	2051	1	3	0.75
160	cme-miR172a	GGAATCTTGATGATGCTGCAG	MSTRG.23975.2	1	1427-1447	1438	4	1	0.5
161	cme-miR172a	GGAATCTTGATGATGCTGCAG	LOC_Os06g43220.1	1	1480-1500	1491	4	1	0.5
162	cme-miR172a	GGAATCTTGATGATGCTGCAG	LOC_Os07g13170.2	1.5	1463-1483	1474	2	3	0.75
163	cme-miR172a	GGAATCTTGATGATGCTGCAG	LOC_Os07g13170.1	1.5	1404-1424	1415	2	3	0.75
164	cme-miR172a	GGAATCTTGATGATGCTGCAG	MSTRG.25169.4	1.5	1572-1592	1583	2	3	0.75
165	cme-miR172a	GGAATCTTGATGATGCTGCAG	MSTRG.25169.1	1.5	1565-1585	1576	2	3	0.75
166	gma-miR166m_L+1_1ss21CA	TCGGACCAGGCTTCATTCCCA	LOC_Os03g01890.2	3	1089-1109	1100	0	59	6
167	gma-miR166m_L+1_1ss21CA	TCGGACCAGGCTTCATTCCCA	MSTRG.13058.1	3	763-783	774	0	53	5.4
168	gma-miR166m_L+1_1ss21CA	TCGGACCAGGCTTCATTCCCA	MSTRG.13058.3	3	997-1017	1008	0	57	5.8
169	gma-miR166m_L+1_1ss21CA	TCGGACCAGGCTTCATTCCCA	LOC_Os03g01890.1	3	1089-1109	1100	0	57	5.8
170	gma-miR166m_L+1_1ss21CA	TCGGACCAGGCTTCATTCCCA	MSTRG.13058.2	3	1185-1205	1196	0	63	6.4
171	gma-miR166m_L+1_1ss21CA	TCGGACCAGGCTTCATTCCCA	LOC_Os04g48290.1	4	400-420	411	2	2	2
172	gma-miR166m_L+1_1ss21CA	TCGGACCAGGCTTCATTCCCA	LOC_Os10g33960.3	3	924-944	935	0	65	6.5
173	gma-miR166m_L+1_1ss21CA	TCGGACCAGGCTTCATTCCCA	LOC_Os10g33960.4	3	924-944	935	0	68	6.8
174	gma-miR166m_L+1_1ss21CA	TCGGACCAGGCTTCATTCCCA	LOC_Os10g33960.2	3	696-716	707	0	65	6.5

175	gma-miR166m_L+1_1ss21CA	TCGGACCAGGCTTCATTCCCA	LOC_Os10g33960.1	3	924-944	935	0	62	6.2
176	gma-miR166m_L+1_1ss21CA	TCGGACCAGGCTTCATTCCCA	MSTRG.5082.2	3	1060-1080	1071	0	63	6.3
177	gma-miR166m_L+1_1ss21CA	TCGGACCAGGCTTCATTCCCA	MSTRG.5082.3	3	1127-1147	1138	0	67	6.7
178	gma-miR166m_L+1_1ss21CA	TCGGACCAGGCTTCATTCCCA	MSTRG.9425.2	2	498-518	509	2	6	0.6
179	gma-miR166m_L+1_1ss21CA	TCGGACCAGGCTTCATTCCCA	LOC_Os12g41860.1	2	877-897	888	2	7	0.7
180	gma-miR166m_L+1_1ss21CA	TCGGACCAGGCTTCATTCCCA	MSTRG.15291.7	2	328-348	339	2	7	0.7
181	gma-miR166m_L+1_1ss21CA	TCGGACCAGGCTTCATTCCCA	MSTRG.15291.5	2	360-380	371	2	8	0.8
182	gma-miR166m_L+1_1ss21CA	TCGGACCAGGCTTCATTCCCA	MSTRG.15291.1	2	540-560	551	2	7	0.7
183	gma-miR166m_L+1_1ss21CA	TCGGACCAGGCTTCATTCCCA	MSTRG.15291.8	2	918-938	929	2	6	0.6
184	gma-miR166m_L+1_1ss21CA	TCGGACCAGGCTTCATTCCCA	LOC_Os03g43930.2	2	955-975	966	2	8	0.8
185	gma-miR166m_L+1_1ss21CA	TCGGACCAGGCTTCATTCCCA	LOC_Os03g43930.1	2	955-975	966	2	7	0.7
186	gma-miR166m_L+1_1ss21CA	TCGGACCAGGCTTCATTCCCA	MSTRG.15291.2	2	886-906	897	2	8	0.8
187	gma-miR166m_L+1_1ss21CA	TCGGACCAGGCTTCATTCCCA	MSTRG.15291.9	2	917-937	928	2	8	0.8
188	gma-miR167e	TGAAGCTGCCAGCATGATCTT	LOC_Os09g39420.1	3.5	2909-2929	2920	2	8	8
189	gma-miR167e	TGAAGCTGCCAGCATGATCTT	MSTRG.9434.2	4	3154-3175	3165	2	11	2.75
190	gma-miR167e	TGAAGCTGCCAGCATGATCTT	LOC_Os12g41950.1	4	3322-3343	3333	2	11	2.75
191	gma-miR167e	TGAAGCTGCCAGCATGATCTT	MSTRG.9434.3	4	3387-3408	3398	2	11	2.75
192	gma-miR167e	TGAAGCTGCCAGCATGATCTT	MSTRG.9434.4	4	3595-3616	3606	2	11	2.75
193	gma-miR171a_L+1	TTGAGCCGTGCCAATATCACGA	MSTRG.3519.3	3.5	1158-1179	1170	4	1	0.25
194	gma-miR171a_L+1	TTGAGCCGTGCCAATATCACGA	MSTRG.3519.2	3.5	2164-2185	2176	4	1	0.25
195	gma-miR171a_L+1	TTGAGCCGTGCCAATATCACGA	LOC_Os01g66140.1	3.5	2418-2439	2430	4	1	0.25
196	gma-miR171a_L+1	TTGAGCCGTGCCAATATCACGA	MSTRG.3519.1	3.5	2463-2484	2475	4	1	0.25
197	gma-miR171a_L+1	TTGAGCCGTGCCAATATCACGA	MSTRG.11971.1	3	1405-1426	1417	0	8	4
198	gma-miR171a_L+1	TTGAGCCGTGCCAATATCACGA	LOC_Os02g44370.1	3	1522-1543	1534	0	8	4
199	gma-miR171a_L+1	TTGAGCCGTGCCAATATCACGA	LOC_Os02g44360.1	4	1347-1368	1359	0	6	6
200	gma-miR171a_L+1	TTGAGCCGTGCCAATATCACGA	LOC_Os04g46860.1	4	1322-1343	1334	0	62	62
201	gma-miR171a_L+1	TTGAGCCGTGCCAATATCACGA	LOC_Os06g01620.1	4	452-473	464	1	2	1
202	gma-miR171a_L+1	TTGAGCCGTGCCAATATCACGA	MSTRG.21932.1	4	709-730	721	2	2	1
203	gma-miR171a_L+1	TTGAGCCGTGCCAATATCACGA	LOC_Os10g40390.1	3	164-185	176	0	35	17.5
204	gma-miR171a_L+1	TTGAGCCGTGCCAATATCACGA	MSTRG.5478.2	3	1162-1183	1174	0	35	17.5
205	gma-miR171m	TTGAGCCGCGTCAATATCTCA	LOC_Os02g44360.1	2	1348-1368	1359	0	6	6
206	gma-miR171m	TTGAGCCGCGTCAATATCTCA	MSTRG.11971.1	2	1406-1426	1417	0	8	4
207	gma-miR171m	TTGAGCCGCGTCAATATCTCA	LOC_Os02g44370.1	2	1523-1543	1534	0	8	4
208	gma-miR171m	TTGAGCCGCGTCAATATCTCA	LOC_Os04g46860.1	2	1323-1343	1334	0	62	62
209	gma-miR171m	TTGAGCCGCGTCAATATCTCA	LOC_Os06g01620.1	2	453-473	464	1	2	1
210	gma-miR171m	TTGAGCCGCGTCAATATCTCA	MSTRG.21932.1	2	710-730	721	2	2	1
211	gma-miR171m	TTGAGCCGCGTCAATATCTCA	LOC_Os10g40390.1	2	165-185	176	0	35	17.5
212	gma-miR171m	TTGAGCCGCGTCAATATCTCA	MSTRG.5478.2	2	1163-1183	1174	0	35	17.5
213	gma-miR408a-3p_L-1R+3	TGCACTGCCTCTTCCCTGGCTTT	LOC_Os03g15340.1	3	82-104	95	2	20	20
214	mdm-miR408a_L-1R+3	TGCACTGCCTCTTCCCTGGCTTT	LOC_Os03g15340.1	3	82-104	95	2	20	20
215	mes-miR2275	TTTGGTTTCCTCCAATATCTTA	MSTRG.1636.4	4	1594-1614	1605	2	2	1
216	mes-miR2275	TTTGGTTTCCTCCAATATCTTA	MSTRG.1636.1	4	1617-1637	1628	2	2	1
217	mes-miR2275	TTTGGTTTCCTCCAATATCTTA	MSTRG.20022.1	4	903-924	915	0	704	704
218	mes-miR2275	TTTGGTTTCCTCCAATATCTTA	MSTRG.5300.1	3.5	13-35	25	0	5668	5668
219	mes-miR2275	TTTGGTTTCCTCCAATATCTTA	MSTRG.7148.1	2.5	1669-1690	1681	0	6011	6011
220	mes-miR2275	TTTGGTTTCCTCCAATATCTTA	MSTRG.7150.1	3	3697-3718	3709	0	373	373
221	mes-miR2275	TTTGGTTTCCTCCAATATCTTA	MSTRG.23851.1	3	8-29	20	0	422	422
222	mtr-miR160a_1ss15TG	TGCCTGGCTCCCTGGATGCCA	LOC_Os02g41800.1	2	1484-1504	1495	0	267	89
223	mtr-miR160a_1ss15TG	TGCCTGGCTCCCTGGATGCCA	MSTRG.11804.1	2	1572-1592	1583	0	267	89
224	mtr-miR160a_1ss15TG	TGCCTGGCTCCCTGGATGCCA	LOC_Os02g41800.2	2	1484-1504	1495	0	267	89
225	mtr-miR160a_1ss15TG	TGCCTGGCTCCCTGGATGCCA	LOC_Os04g43910.1	2	1344-1364	1355	2	3	3
226	mtr-miR160a_1ss15TG	TGCCTGGCTCCCTGGATGCCA	LOC_Os04g59430.1	1.5	1334-1354	1345	2	2	2
227	mtr-miR160a_1ss15TG	TGCCTGGCTCCCTGGATGCCA	LOC_Os10g33940.1	2	1635-1655	1646	0	285	142.5
228	mtr-miR160a_1ss15TG	TGCCTGGCTCCCTGGATGCCA	MSTRG.5076.1	2	1903-1923	1914	0	285	142.5
229	mtr-miR160a_1ss15TG	TGCCTGGCTCCCTGGATGCCA	LOC_Os06g47150.3	2	1811-1831	1822	0	336	84
230	mtr-miR160a_1ss15TG	TGCCTGGCTCCCTGGATGCCA	LOC_Os06g47150.2	2	1844-1864	1855	0	336	84
231	mtr-miR160a_1ss15TG	TGCCTGGCTCCCTGGATGCCA	LOC_Os06g47150.1	2	2042-2062	2053	0	336	84
232	mtr-miR160a_1ss15TG	TGCCTGGCTCCCTGGATGCCA	LOC_Os06g47150.4	2	1844-1864	1855	0	336	84
233	mtr-miR167b-5p_R+1	TGAAGCTGCCAGCATGATCTGT	LOC_Os07g33790.1	3.5	3418-3439	3430	2	2	0.666666667

234	mtr-miR167b-5p_R+1	TGAAGCTGCCAGCATGATCTGT	MSTRG.25835.3	3.5	3369-3390	3381	2	2	0.666666667
235	mtr-miR167b-5p_R+1	TGAAGCTGCCAGCATGATCTGT	MSTRG.25835.2	3.5	3395-3416	3407	2	2	0.666666667
236	mtr-miR167b-5p_R+1	TGAAGCTGCCAGCATGATCTGT	LOC_Os09g39420.1	4	2908-2929	2920	2	8	8
237	mtr-miR171a_L+1_1ss10TC	ATGATTGAGCCGTGCCAATATC	LOC_Os04g46860.1	2	1326-1347	1338	4	1	1
238	osa-miR1425-5p	TAGGATTCAATCCTTGCTGCT	LOC_Os02g49730.1	4	615-636	626	4	1	0.5
239	osa-miR1425-5p	TAGGATTCAATCCTTGCTGCT	MSTRG.12341.1	4	1089-1110	1100	4	1	0.5
240	osa-miR1425-5p	TAGGATTCAATCCTTGCTGCT	LOC_Os10g35640.1	2.5	1376-1396	1387	4	1	1
241	osa-miR1425-5p	TAGGATTCAATCCTTGCTGCT	MSTRG.5167.14	3	1183-1203	1194	4	1	0.142857143
242	osa-miR1425-5p	TAGGATTCAATCCTTGCTGCT	MSTRG.5167.15	3	1183-1203	1194	4	1	0.142857143
243	osa-miR1425-5p	TAGGATTCAATCCTTGCTGCT	LOC_Os10g35436.1	3	1308-1328	1319	4	1	0.142857143
244	osa-miR1425-5p	TAGGATTCAATCCTTGCTGCT	MSTRG.5167.10	3	1526-1546	1537	4	1	0.142857143
245	osa-miR1425-5p	TAGGATTCAATCCTTGCTGCT	MSTRG.5167.12	3	1498-1518	1509	4	1	0.142857143
246	osa-miR1425-5p	TAGGATTCAATCCTTGCTGCT	MSTRG.5167.9	3	1526-1546	1537	4	1	0.142857143
247	osa-miR1425-5p	TAGGATTCAATCCTTGCTGCT	MSTRG.5167.16	3	1098-1118	1109	4	1	0.142857143
248	osa-miR1425-5p	TAGGATTCAATCCTTGCTGCT	LOC_Os10g35230.1	3.5	1437-1457	1448	2	4	4
249	osa-miR1425-5p	TAGGATTCAATCCTTGCTGCT	LOC_Os10g35240.1	3.5	1296-1316	1307	2	5	2.5
250	osa-miR1425-5p	TAGGATTCAATCCTTGCTGCT	LOC_Os10g35240.2	3.5	1299-1319	1310	2	5	2.5
251	osa-miR1427	TGCGGAACCGTGCGGTGGCGC	LOC_Os05g35480.1	4	2459-2478	2469	1	13	13
252	osa-MIR1430-p5	TAGCCAAGAATGGCTTGCCTATC	LOC_Os12g42400.2	4	1200-1222	1213	0	59	19.66666667
253	osa-MIR1430-p5	TAGCCAAGAATGGCTTGCCTATC	LOC_Os12g42400.3	4	1245-1267	1258	0	59	19.66666667
254	osa-MIR1430-p5	TAGCCAAGAATGGCTTGCCTATC	LOC_Os12g42400.1	4	1254-1276	1267	0	59	19.66666667
255	osa-miR1432-3p	CAGGTGTCATCTCCCCTGAAC	LOC_Os03g19580.2	4	311-330	322	2	4	1.333333333
256	osa-miR1432-3p	CAGGTGTCATCTCCCCTGAAC	LOC_Os03g19580.1	4	311-330	322	2	4	1.333333333
257	osa-miR1432-3p	CAGGTGTCATCTCCCCTGAAC	MSTRG.14332.2	4	330-349	341	2	4	1.333333333
258	osa-miR1432-5p_R+1	ATCAGGAGAGATGACACCGACA	MSTRG.10159.2	2	450-471	462	4	1	0.25
259	osa-miR1432-5p_R+1	ATCAGGAGAGATGACACCGACA	MSTRG.10159.4	2	522-543	534	4	1	0.25
260	osa-miR1432-5p_R+1	ATCAGGAGAGATGACACCGACA	MSTRG.10159.1	2	695-716	707	4	1	0.25
261	osa-miR1432-5p_R+1	ATCAGGAGAGATGACACCGACA	MSTRG.10159.3	2	522-543	534	4	1	0.25
262	osa-MIR1441-p5_2ss19TC24TC	TCATTCGTGTCCGAAAACCCCTTC	MSTRG.10564.4	1	1530-1553	1544	2	3	0.75
263	osa-MIR1441-p5_2ss19TC24TC	TCATTCGTGTCCGAAAACCCCTTC	MSTRG.10564.1	1	2071-2094	2085	2	3	0.75
264	osa-MIR1441-p5_2ss19TC24TC	TCATTCGTGTCCGAAAACCCCTTC	LOC_Os02g15360.1	1	2331-2354	2345	2	3	0.75
265	osa-MIR1441-p5_2ss19TC24TC	TCATTCGTGTCCGAAAACCCCTTC	MSTRG.13831.1	0	2643-2666	2657	2	3	0.75
266	osa-MIR1442-p3_2ss15AC18TC	GACACATCCTAGTACAACGAATCT	LOC_Os12g39380.1	1	2723-2746	2737	4	1	1
267	osa-miR156a	TGACAGAAGAGAGTGAGCAC	LOC_Os07g32170.2	2	664-683	674	4	1	0.333333333
268	osa-miR156a	TGACAGAAGAGAGTGAGCAC	MSTRG.25734.1	2	660-679	670	4	1	0.333333333
269	osa-miR156a	TGACAGAAGAGAGTGAGCAC	LOC_Os07g32170.1	2	852-871	862	4	1	0.333333333
270	osa-miR156a	TGACAGAAGAGAGTGAGCAC	LOC_Os08g41940.1	1	1054-1073	1064	0	10	10
271	osa-miR156a	TGACAGAAGAGAGTGAGCAC	LOC_Os08g39890.1	1	992-1011	1002	2	3	1.5
272	osa-miR156a	TGACAGAAGAGAGTGAGCAC	MSTRG.28596.1	1	1089-1108	1099	2	3	1.5
273	osa-miR156a	TGACAGAAGAGAGTGAGCAC	LOC_Os09g32944.1	1	1034-1053	1044	4	1	0.5
274	osa-miR156a	TGACAGAAGAGAGTGAGCAC	MSTRG.30197.1	1	1760-1779	1770	4	1	0.5
275	osa-miR156a	TGACAGAAGAGAGTGAGCAC	MSTRG.10135.3	1	837-856	847	4	1	0.333333333
276	osa-miR156a	TGACAGAAGAGAGTGAGCAC	LOC_Os02g07780.1	1	987-1006	997	4	1	0.333333333
277	osa-miR156a	TGACAGAAGAGAGTGAGCAC	LOC_Os02g07780.2	1	2221-2240	2231	4	1	0.333333333
278	osa-miR156a	TGACAGAAGAGAGTGAGCAC	LOC_Os02g04680.2	2	1962-1981	1972	0	14	1.4
279	osa-miR156a	TGACAGAAGAGAGTGAGCAC	MSTRG.9878.8	2	1960-1979	1970	0	14	1.4
280	osa-miR156a	TGACAGAAGAGAGTGAGCAC	LOC_Os02g04680.1	2	1965-1984	1975	0	14	1.4
281	osa-miR156a	TGACAGAAGAGAGTGAGCAC	MSTRG.9878.7	2	1972-1991	1982	0	14	1.4
282	osa-miR156a	TGACAGAAGAGAGTGAGCAC	MSTRG.9878.6	2	2154-2173	2164	0	14	1.4
283	osa-miR156a	TGACAGAAGAGAGTGAGCAC	MSTRG.9878.5	2	2280-2299	2290	0	14	1.4
284	osa-miR156a	TGACAGAAGAGAGTGAGCAC	MSTRG.9878.4	2	2512-2531	2522	0	14	1.4
285	osa-miR156a	TGACAGAAGAGAGTGAGCAC	MSTRG.9878.1	2	2580-2599	2590	0	14	1.4
286	osa-miR156a	TGACAGAAGAGAGTGAGCAC	MSTRG.9878.3	2	2603-2622	2613	0	14	1.4
287	osa-miR156a	TGACAGAAGAGAGTGAGCAC	MSTRG.9878.2	2	3038-3057	3048	0	14	1.4
288	osa-miR156a	TGACAGAAGAGAGTGAGCAC	LOC_Os06g45310.1	1	854-873	864	2	2	2
289	osa-miR156a	TGACAGAAGAGAGTGAGCAC	LOC_Os06g49010.3	1	1439-1458	1449	1	13	1.3
290	osa-miR156a	TGACAGAAGAGAGTGAGCAC	LOC_Os06g49010.2	1	1494-1513	1504	0	13	1.3
291	osa-miR156a	TGACAGAAGAGAGTGAGCAC	LOC_Os06g49010.4	1	1638-1657	1648	0	17	1.7
292	osa-miR156a	TGACAGAAGAGAGTGAGCAC	LOC_Os06g49010.1	1	1686-1705	1696	0	14	1.4

293	osa-miR156a	TGACAGAAGAGAGTGAGCAC	MSTRG.24320.4	1	1729-1748	1739	0	15	1.5
294	osa-miR156a	TGACAGAAGAGAGTGAGCAC	MSTRG.24320.6	1	1830-1849	1840	0	14	1.4
295	osa-miR156a	TGACAGAAGAGAGTGAGCAC	LOC_Os06g49010.6	1	1842-1861	1852	0	14	1.4
296	osa-miR156a	TGACAGAAGAGAGTGAGCAC	MSTRG.24320.18	1	1939-1958	1949	0	14	1.4
297	osa-miR156a	TGACAGAAGAGAGTGAGCAC	MSTRG.24320.21	1	2025-2044	2035	0	14	1.4
298	osa-miR156a	TGACAGAAGAGAGTGAGCAC	LOC_Os06g49010.5	1	2018-2037	2028	0	17	1.7
299	osa-miR156a	TGACAGAAGAGAGTGAGCAC	MSTRG.24320.23	1	2315-2334	2325	0	15	1.5
300	osa-miR156a	TGACAGAAGAGAGTGAGCAC	MSTRG.24320.16	1	2279-2298	2289	0	14	1.4
301	osa-miR156a	TGACAGAAGAGAGTGAGCAC	MSTRG.24320.14	1	2298-2317	2308	0	16	1.6
302	osa-miR156a	TGACAGAAGAGAGTGAGCAC	MSTRG.24320.19	1	2413-2432	2423	0	14	1.4
303	osa-miR156a	TGACAGAAGAGAGTGAGCAC	MSTRG.24320.5	1	2360-2379	2370	0	18	1.8
304	osa-miR156a	TGACAGAAGAGAGTGAGCAC	MSTRG.24320.20	1	2684-2703	2694	0	14	1.4
305	osa-miR156a	TGACAGAAGAGAGTGAGCAC	MSTRG.24320.24	1	2743-2762	2753	0	13	1.3
306	osa-miR156a	TGACAGAAGAGAGTGAGCAC	MSTRG.24320.17	1	2673-2692	2683	0	16	1.6
307	osa-miR156a	TGACAGAAGAGAGTGAGCAC	MSTRG.24320.22	1	2781-2800	2791	1	12	1.2
308	osa-miR156a	TGACAGAAGAGAGTGAGCAC	MSTRG.24320.1	1	2717-2736	2727	0	14	1.4
309	osa-miR156a	TGACAGAAGAGAGTGAGCAC	MSTRG.24320.15	1	2764-2783	2774	0	16	1.6
310	osa-miR156a	TGACAGAAGAGAGTGAGCAC	MSTRG.24320.2	1	2768-2787	2778	0	14	1.4
311	osa-miR156a	TGACAGAAGAGAGTGAGCAC	MSTRG.24320.3	1	2787-2806	2797	0	15	1.5
312	osa-miR156a	TGACAGAAGAGAGTGAGCAC	MSTRG.24320.13	1	2864-2883	2874	0	14	1.4
313	osa-MIR156e-p3	GCTCACTGCTCTTTCTGTCATC	MSTRG.1811.2	3	1595-1615	1607	4	1	0.166666667
314	osa-MIR156e-p3	GCTCACTGCTCTTTCTGTCATC	MSTRG.1811.3	3	1841-1861	1853	4	1	0.166666667
315	osa-MIR156e-p3	GCTCACTGCTCTTTCTGTCATC	MSTRG.1811.5	3	1849-1869	1861	4	1	0.166666667
316	osa-MIR156e-p3	GCTCACTGCTCTTTCTGTCATC	LOC_Os01g39250.1	3	1911-1931	1923	4	1	0.166666667
317	osa-MIR156e-p3	GCTCACTGCTCTTTCTGTCATC	MSTRG.1811.6	3	2033-2053	2045	4	1	0.166666667
318	osa-MIR156e-p3	GCTCACTGCTCTTTCTGTCATC	MSTRG.1811.4	3	2170-2190	2182	4	1	0.166666667
319	osa-miR156j-3p	GCTCGCTCCTCTTTCTGTCAGC	MSTRG.1568.2	4	2983-3003	2994	2	2	1
320	osa-miR156j-3p	GCTCGCTCCTCTTTCTGTCAGC	LOC_Os01g33040.1	4	2928-2948	2939	2	2	1
321	osa-miR156j-3p	GCTCGCTCCTCTTTCTGTCAGC	LOC_Os12g09580.1	3.5	1605-1625	1616	2	8	8
322	osa-miR156l-5p_R-1	CGACAGAAGAGAGTGAGCAT	LOC_Os07g32170.2	2	664-683	674	4	1	0.333333333
323	osa-miR156l-5p_R-1	CGACAGAAGAGAGTGAGCAT	MSTRG.25734.1	2	660-679	670	4	1	0.333333333
324	osa-miR156l-5p_R-1	CGACAGAAGAGAGTGAGCAT	LOC_Os07g32170.1	2	852-871	862	4	1	0.333333333
325	osa-miR156l-5p_R-1	CGACAGAAGAGAGTGAGCAT	LOC_Os08g41940.1	2.5	1054-1073	1064	0	10	10
326	osa-miR156l-5p_R-1	CGACAGAAGAGAGTGAGCAT	LOC_Os08g39890.1	2.5	992-1011	1002	2	3	1.5
327	osa-miR156l-5p_R-1	CGACAGAAGAGAGTGAGCAT	MSTRG.28596.1	2.5	1089-1108	1099	2	3	1.5
328	osa-miR156l-5p_R-1	CGACAGAAGAGAGTGAGCAT	LOC_Os09g32944.1	2.5	1034-1053	1044	4	1	0.5
329	osa-miR156l-5p_R-1	CGACAGAAGAGAGTGAGCAT	MSTRG.30197.1	2.5	1760-1779	1770	4	1	0.5
330	osa-miR156l-5p_R-1	CGACAGAAGAGAGTGAGCAT	LOC_Os02g04680.2	2	1962-1981	1972	0	14	1.4
331	osa-miR156l-5p_R-1	CGACAGAAGAGAGTGAGCAT	MSTRG.9878.8	2	1960-1979	1970	0	14	1.4
332	osa-miR156l-5p_R-1	CGACAGAAGAGAGTGAGCAT	LOC_Os02g04680.1	2	1965-1984	1975	0	14	1.4
333	osa-miR156l-5p_R-1	CGACAGAAGAGAGTGAGCAT	MSTRG.9878.7	2	1972-1991	1982	0	14	1.4
334	osa-miR156l-5p_R-1	CGACAGAAGAGAGTGAGCAT	MSTRG.9878.6	2	2154-2173	2164	0	14	1.4
335	osa-miR156l-5p_R-1	CGACAGAAGAGAGTGAGCAT	MSTRG.9878.5	2	2280-2299	2290	0	14	1.4
336	osa-miR156l-5p_R-1	CGACAGAAGAGAGTGAGCAT	MSTRG.9878.4	2	2512-2531	2522	0	14	1.4
337	osa-miR156l-5p_R-1	CGACAGAAGAGAGTGAGCAT	MSTRG.9878.1	2	2580-2599	2590	0	14	1.4
338	osa-miR156l-5p_R-1	CGACAGAAGAGAGTGAGCAT	MSTRG.9878.3	2	2603-2622	2613	0	14	1.4
339	osa-miR156l-5p_R-1	CGACAGAAGAGAGTGAGCAT	MSTRG.9878.2	2	3038-3057	3048	0	14	1.4
340	osa-miR156l-5p_R-1	CGACAGAAGAGAGTGAGCAT	MSTRG.10135.3	2.5	837-856	847	4	1	0.333333333
341	osa-miR156l-5p_R-1	CGACAGAAGAGAGTGAGCAT	LOC_Os02g07780.1	2.5	987-1006	997	4	1	0.333333333
342	osa-miR156l-5p_R-1	CGACAGAAGAGAGTGAGCAT	LOC_Os02g07780.2	2.5	2221-2240	2231	4	1	0.333333333
343	osa-miR156l-5p_R-1	CGACAGAAGAGAGTGAGCAT	LOC_Os06g45310.1	2.5	854-873	864	2	2	2
344	osa-miR156l-5p_R-1	CGACAGAAGAGAGTGAGCAT	LOC_Os06g49010.3	2.5	1439-1458	1449	1	13	1.3
345	osa-miR156l-5p_R-1	CGACAGAAGAGAGTGAGCAT	LOC_Os06g49010.2	2.5	1494-1513	1504	0	13	1.3
346	osa-miR156l-5p_R-1	CGACAGAAGAGAGTGAGCAT	LOC_Os06g49010.4	2.5	1638-1657	1648	0	17	1.7
347	osa-miR156l-5p_R-1	CGACAGAAGAGAGTGAGCAT	LOC_Os06g49010.1	2.5	1686-1705	1696	0	14	1.4
348	osa-miR156l-5p_R-1	CGACAGAAGAGAGTGAGCAT	MSTRG.24320.4	2.5	1729-1748	1739	0	15	1.5
349	osa-miR156l-5p_R-1	CGACAGAAGAGAGTGAGCAT	MSTRG.24320.6	2.5	1830-1849	1840	0	14	1.4
350	osa-miR156l-5p_R-1	CGACAGAAGAGAGTGAGCAT	LOC_Os06g49010.6	2.5	1842-1861	1852	0	14	1.4
351	osa-miR156l-5p_R-1	CGACAGAAGAGAGTGAGCAT	MSTRG.24320.18	2.5	1939-1958	1949	0	14	1.4

352	osa-miR156l-5p_R-1	CGACAGAAGAGAGTGAGCAT	MSTRG.24320.21	2.5	2025-2044	2035	0	14	1.4
353	osa-miR156l-5p_R-1	CGACAGAAGAGAGTGAGCAT	LOC_Os06g49010.5	2.5	2018-2037	2028	0	17	1.7
354	osa-miR156l-5p_R-1	CGACAGAAGAGAGTGAGCAT	MSTRG.24320.23	2.5	2315-2334	2325	0	15	1.5
355	osa-miR156l-5p_R-1	CGACAGAAGAGAGTGAGCAT	MSTRG.24320.16	2.5	2279-2298	2289	0	14	1.4
356	osa-miR156l-5p_R-1	CGACAGAAGAGAGTGAGCAT	MSTRG.24320.14	2.5	2298-2317	2308	0	16	1.6
357	osa-miR156l-5p_R-1	CGACAGAAGAGAGTGAGCAT	MSTRG.24320.19	2.5	2413-2432	2423	0	14	1.4
358	osa-miR156l-5p_R-1	CGACAGAAGAGAGTGAGCAT	MSTRG.24320.5	2.5	2360-2379	2370	0	18	1.8
359	osa-miR156l-5p_R-1	CGACAGAAGAGAGTGAGCAT	MSTRG.24320.20	2.5	2684-2703	2694	0	14	1.4
360	osa-miR156l-5p_R-1	CGACAGAAGAGAGTGAGCAT	MSTRG.24320.24	2.5	2743-2762	2753	0	13	1.3
361	osa-miR156l-5p_R-1	CGACAGAAGAGAGTGAGCAT	MSTRG.24320.17	2.5	2673-2692	2683	0	16	1.6
362	osa-miR156l-5p_R-1	CGACAGAAGAGAGTGAGCAT	MSTRG.24320.22	2.5	2781-2800	2791	1	12	1.2
363	osa-miR156l-5p_R-1	CGACAGAAGAGAGTGAGCAT	MSTRG.24320.1	2.5	2717-2736	2727	0	14	1.4
364	osa-miR156l-5p_R-1	CGACAGAAGAGAGTGAGCAT	MSTRG.24320.15	2.5	2764-2783	2774	0	16	1.6
365	osa-miR156l-5p_R-1	CGACAGAAGAGAGTGAGCAT	MSTRG.24320.2	2.5	2768-2787	2778	0	14	1.4
366	osa-miR156l-5p_R-1	CGACAGAAGAGAGTGAGCAT	MSTRG.24320.3	2.5	2787-2806	2797	0	15	1.5
367	osa-miR156l-5p_R-1	CGACAGAAGAGAGTGAGCAT	MSTRG.24320.13	2.5	2864-2883	2874	0	14	1.4
368	osa-miR159a.1	TTTGGATTGAAGGGAGCTCTG	MSTRG.2268.8	4	1505-1526	1516	3	2	0.25
369	osa-miR159a.1	TTTGGATTGAAGGGAGCTCTG	LOC_Os01g47530.3	4	1683-1704	1694	2	2	0.25
370	osa-miR159a.1	TTTGGATTGAAGGGAGCTCTG	LOC_Os01g47530.2	4	1797-1818	1808	2	2	0.25
371	osa-miR159a.1	TTTGGATTGAAGGGAGCTCTG	MSTRG.2268.3	4	2489-2510	2500	2	2	0.25
372	osa-miR159a.1	TTTGGATTGAAGGGAGCTCTG	MSTRG.2268.4	4	2590-2611	2601	2	2	0.25
373	osa-miR159a.1	TTTGGATTGAAGGGAGCTCTG	LOC_Os01g47530.1	4	2431-2452	2442	2	2	0.25
374	osa-miR159a.1	TTTGGATTGAAGGGAGCTCTG	MSTRG.2268.5	4	2620-2641	2631	2	2	0.25
375	osa-miR159a.1	TTTGGATTGAAGGGAGCTCTG	LOC_Os01g59660.1	3.5	1260-1280	1271	2	26	6.5
376	osa-miR159a.1	TTTGGATTGAAGGGAGCTCTG	LOC_Os01g59660.4	3.5	1180-1200	1191	2	26	6.5
377	osa-miR159a.1	TTTGGATTGAAGGGAGCTCTG	LOC_Os01g59660.3	3.5	1258-1278	1269	2	26	6.5
378	osa-miR159a.1	TTTGGATTGAAGGGAGCTCTG	LOC_Os01g59660.2	3.5	1348-1368	1359	2	26	6.5
379	osa-miR159a.1	TTTGGATTGAAGGGAGCTCTG	LOC_Os09g36650.1	3.5	699-719	710	4	1	0.5
380	osa-miR159a.1	TTTGGATTGAAGGGAGCTCTG	MSTRG.30377.1	3.5	870-890	881	4	1	0.5
381	osa-miR159a.1	TTTGGATTGAAGGGAGCTCTG	LOC_Os01g11430.1	3.5	1112-1132	1123	2	45	22.5
382	osa-miR159a.1	TTTGGATTGAAGGGAGCTCTG	MSTRG.705.1	3.5	936-956	947	2	45	22.5
383	osa-miR159a.1	TTTGGATTGAAGGGAGCTCTG	LOC_Os06g40330.1	3.5	1418-1438	1429	4	1	1
384	osa-MIR159a-p5	TTGAGCTCCTTTCGGTCCAAA	LOC_Os02g34460.2	4	529-549	540	4	1	0.5
385	osa-MIR159a-p5	TTGAGCTCCTTTCGGTCCAAA	MSTRG.11366.1	4	735-755	746	4	1	0.5
386	osa-MIR159b-p5	GAGCTCCTTTCGTTCCAATGA	LOC_Os02g34460.2	4	527-547	538	2	3	1.5
387	osa-MIR159b-p5	GAGCTCCTTTCGTTCCAATGA	MSTRG.11366.1	4	733-753	744	2	3	1.5
388	osa-MIR159b-p5	GAGCTCCTTTCGTTCCAATGA	MSTRG.6635.2	4	1408-1429	1420	2	2	1
389	osa-MIR159b-p5	GAGCTCCTTTCGTTCCAATGA	LOC_Os11g24560.1	4	1493-1514	1505	2	2	1
390	osa-miR159f_R-1	CTTGGATTGAAGGGAGCTCT	MSTRG.2268.8	4	1506-1526	1516	3	2	0.25
391	osa-miR159f_R-1	CTTGGATTGAAGGGAGCTCT	LOC_Os01g47530.3	4	1684-1704	1694	2	2	0.25
392	osa-miR159f_R-1	CTTGGATTGAAGGGAGCTCT	LOC_Os01g47530.2	4	1798-1818	1808	2	2	0.25
393	osa-miR159f_R-1	CTTGGATTGAAGGGAGCTCT	MSTRG.2268.3	4	2490-2510	2500	2	2	0.25
394	osa-miR159f_R-1	CTTGGATTGAAGGGAGCTCT	LOC_Os01g47530.4	4	2355-2375	2365	2	2	0.25
395	osa-miR159f_R-1	CTTGGATTGAAGGGAGCTCT	MSTRG.2268.4	4	2591-2611	2601	2	2	0.25
396	osa-miR159f_R-1	CTTGGATTGAAGGGAGCTCT	LOC_Os01g47530.1	4	2432-2452	2442	2	2	0.25
397	osa-miR159f_R-1	CTTGGATTGAAGGGAGCTCT	MSTRG.2268.5	4	2621-2641	2631	2	2	0.25
398	osa-miR159f_R-1	CTTGGATTGAAGGGAGCTCT	LOC_Os01g59660.1	2.5	1261-1280	1271	2	26	6.5
399	osa-miR159f_R-1	CTTGGATTGAAGGGAGCTCT	LOC_Os01g59660.4	2.5	1181-1200	1191	2	26	6.5
400	osa-miR159f_R-1	CTTGGATTGAAGGGAGCTCT	LOC_Os01g59660.3	2.5	1259-1278	1269	2	26	6.5
401	osa-miR159f_R-1	CTTGGATTGAAGGGAGCTCT	LOC_Os01g59660.2	2.5	1349-1368	1359	2	26	6.5
402	osa-miR159f_R-1	CTTGGATTGAAGGGAGCTCT	LOC_Os09g36650.1	3.5	700-719	710	4	1	0.5
403	osa-miR159f_R-1	CTTGGATTGAAGGGAGCTCT	MSTRG.30377.1	3.5	871-890	881	4	1	0.5
404	osa-miR159f_R-1	CTTGGATTGAAGGGAGCTCT	LOC_Os06g40330.1	3	1419-1438	1429	4	1	1
405	osa-miR160a-5p	TGCCTGGCTCCCTGTATGCCA	LOC_Os02g41800.1	1	1484-1504	1495	0	267	89
406	osa-miR160a-5p	TGCCTGGCTCCCTGTATGCCA	MSTRG.11804.1	1	1572-1592	1583	0	267	89
407	osa-miR160a-5p	TGCCTGGCTCCCTGTATGCCA	LOC_Os02g41800.2	1	1484-1504	1495	0	267	89
408	osa-miR160a-5p	TGCCTGGCTCCCTGTATGCCA	LOC_Os04g43910.1	1	1344-1364	1355	2	3	3
409	osa-miR160a-5p	TGCCTGGCTCCCTGTATGCCA	LOC_Os04g59430.1	2	1334-1354	1345	2	2	2
410	osa-miR160a-5p	TGCCTGGCTCCCTGTATGCCA	LOC_Os10g33940.1	1	1635-1655	1646	0	285	142.5

411	osa-miR160a-5p	TGCCTGGCTCCCTGTATGCCA	MSTRG.5076.1	1	1903-1923	1914	0	285	142.5
412	osa-miR160a-5p	TGCCTGGCTCCCTGTATGCCA	LOC_Os06g47150.3	1	1811-1831	1822	0	336	84
413	osa-miR160a-5p	TGCCTGGCTCCCTGTATGCCA	LOC_Os06g47150.2	1	1844-1864	1855	0	336	84
414	osa-miR160a-5p	TGCCTGGCTCCCTGTATGCCA	LOC_Os06g47150.1	1	2042-2062	2053	0	336	84
415	osa-miR160a-5p	TGCCTGGCTCCCTGTATGCCA	LOC_Os06g47150.4	1	1844-1864	1855	0	336	84
416	osa-miR160e-5p	TGCCTGGCTCCCTGTATGCCG	LOC_Os02g41800.1	1	1484-1504	1495	0	267	89
417	osa-miR160e-5p	TGCCTGGCTCCCTGTATGCCG	MSTRG.11804.1	1	1572-1592	1583	0	267	89
418	osa-miR160e-5p	TGCCTGGCTCCCTGTATGCCG	LOC_Os02g41800.2	1	1484-1504	1495	0	267	89
419	osa-miR160e-5p	TGCCTGGCTCCCTGTATGCCG	LOC_Os04g43910.1	1	1344-1364	1355	2	3	3
420	osa-miR160e-5p	TGCCTGGCTCCCTGTATGCCG	LOC_Os04g59430.1	2.5	1334-1354	1345	2	2	2
421	osa-miR160e-5p	TGCCTGGCTCCCTGTATGCCG	LOC_Os10g33940.1	1	1635-1655	1646	0	285	142.5
422	osa-miR160e-5p	TGCCTGGCTCCCTGTATGCCG	MSTRG.5076.1	1	1903-1923	1914	0	285	142.5
423	osa-miR160e-5p	TGCCTGGCTCCCTGTATGCCG	LOC_Os06g47150.3	1	1811-1831	1822	0	336	84
424	osa-miR160e-5p	TGCCTGGCTCCCTGTATGCCG	LOC_Os06g47150.2	1	1844-1864	1855	0	336	84
425	osa-miR160e-5p	TGCCTGGCTCCCTGTATGCCG	LOC_Os06g47150.1	1	2042-2062	2053	0	336	84
426	osa-miR160e-5p	TGCCTGGCTCCCTGTATGCCG	LOC_Os06g47150.4	1	1844-1864	1855	0	336	84
427	osa-miR160f-5p_L+1_1ss16AT	CTGCCTGGCTCCCTGTATGCCA	LOC_Os02g41800.1	2	1484-1505	1496	4	1	0.333333333
428	osa-miR160f-5p_L+1_1ss16AT	CTGCCTGGCTCCCTGTATGCCA	MSTRG.11804.1	2	1572-1593	1584	4	1	0.333333333
429	osa-miR160f-5p_L+1_1ss16AT	CTGCCTGGCTCCCTGTATGCCA	LOC_Os02g41800.2	2	1484-1505	1496	4	1	0.333333333
430	osa-miR160f-5p_L+1_1ss16AT	CTGCCTGGCTCCCTGTATGCCA	LOC_Os10g33940.1	2	1635-1656	1647	2	4	2
431	osa-miR160f-5p_L+1_1ss16AT	CTGCCTGGCTCCCTGTATGCCA	MSTRG.5076.1	2	1903-1924	1915	2	4	2
432	osa-miR160f-5p_L+1_1ss16AT	CTGCCTGGCTCCCTGTATGCCA	LOC_Os06g47150.3	2	1811-1832	1823	2	6	1.5
433	osa-miR160f-5p_L+1_1ss16AT	CTGCCTGGCTCCCTGTATGCCA	LOC_Os06g47150.2	2	1844-1865	1856	2	6	1.5
434	osa-miR160f-5p_L+1_1ss16AT	CTGCCTGGCTCCCTGTATGCCA	LOC_Os06g47150.1	2	2042-2063	2054	2	6	1.5
435	osa-miR160f-5p_L+1_1ss16AT	CTGCCTGGCTCCCTGTATGCCA	LOC_Os06g47150.4	2	1844-1865	1856	2	6	1.5
436	osa-miR162a	TCGATAAACCTCTGCATCCAG	LOC_Os03g02970.1	2	2977-2998	2988	2	4	0.666666667
437	osa-miR162a	TCGATAAACCTCTGCATCCAG	MSTRG.13143.5	2	3554-3575	3565	2	4	0.666666667
438	osa-miR162a	TCGATAAACCTCTGCATCCAG	MSTRG.13143.3	2	3540-3561	3551	2	4	0.666666667
439	osa-miR162a	TCGATAAACCTCTGCATCCAG	MSTRG.13143.2	2	3543-3564	3554	2	4	0.666666667
440	osa-miR162a	TCGATAAACCTCTGCATCCAG	MSTRG.13143.6	2	3637-3658	3648	2	4	0.666666667
441	osa-miR162a	TCGATAAACCTCTGCATCCAG	MSTRG.13143.1	2	3723-3744	3734	2	4	0.666666667
442	osa-miR162b	TCGATAAGCCTCTGCATCCAG	LOC_Os03g02970.1	3	2977-2998	2988	2	4	0.666666667
443	osa-miR162b	TCGATAAGCCTCTGCATCCAG	MSTRG.13143.5	3	3554-3575	3565	2	4	0.666666667
444	osa-miR162b	TCGATAAGCCTCTGCATCCAG	MSTRG.13143.3	3	3540-3561	3551	2	4	0.666666667
445	osa-miR162b	TCGATAAGCCTCTGCATCCAG	MSTRG.13143.2	3	3543-3564	3554	2	4	0.666666667
446	osa-miR162b	TCGATAAGCCTCTGCATCCAG	MSTRG.13143.6	3	3637-3658	3648	2	4	0.666666667
447	osa-miR162b	TCGATAAGCCTCTGCATCCAG	MSTRG.13143.1	3	3723-3744	3734	2	4	0.666666667
448	osa-miR164a	TGGAGAAGCAGGGCACGTGCA	MSTRG.18003.2	3	784-804	795	4	1	0.5
449	osa-miR164a	TGGAGAAGCAGGGCACGTGCA	LOC_Os04g38720.1	3	800-820	811	4	1	0.5
450	osa-miR164a	TGGAGAAGCAGGGCACGTGCA	MSTRG.9408.2	2	873-893	884	2	2	1
451	osa-miR164a	TGGAGAAGCAGGGCACGTGCA	LOC_Os12g41680.1	2	911-931	922	2	2	1
452	osa-miR164a	TGGAGAAGCAGGGCACGTGCA	LOC_Os06g23650.1	2	794-814	805	0	3	3
453	osa-miR164a	TGGAGAAGCAGGGCACGTGCA	LOC_Os06g46270.1	2	954-974	965	1	4	4
454	osa-miR164a	TGGAGAAGCAGGGCACGTGCA	LOC_Os12g05260.1	3	202-222	213	2	2	2
455	osa-miR164c	TGGAGAAGCAGGGTACGTGCA	MSTRG.18003.2	3.5	784-804	795	4	1	0.5
456	osa-miR164c	TGGAGAAGCAGGGTACGTGCA	LOC_Os04g38720.1	3.5	800-820	811	4	1	0.5
457	osa-miR164c	TGGAGAAGCAGGGTACGTGCA	MSTRG.9408.2	2.5	873-893	884	2	2	1
458	osa-miR164c	TGGAGAAGCAGGGTACGTGCA	LOC_Os12g41680.1	2.5	911-931	922	2	2	1
459	osa-miR164c	TGGAGAAGCAGGGTACGTGCA	LOC_Os06g23650.1	2.5	794-814	805	0	3	3
460	osa-miR164c	TGGAGAAGCAGGGTACGTGCA	LOC_Os06g46270.1	2.5	954-974	965	1	4	4
461	osa-miR164c	TGGAGAAGCAGGGTACGTGCA	LOC_Os12g05260.1	3.5	202-222	213	2	2	2
462	osa-miR164d_R-1	TGGAGAAGCAGGGCACGTGC	MSTRG.18003.2	2	785-804	795	4	1	0.5
463	osa-miR164d_R-1	TGGAGAAGCAGGGCACGTGC	LOC_Os04g38720.1	2	801-820	811	4	1	0.5
464	osa-miR164d_R-1	TGGAGAAGCAGGGCACGTGC	MSTRG.9408.2	1	874-893	884	2	2	1
465	osa-miR164d_R-1	TGGAGAAGCAGGGCACGTGC	LOC_Os12g41680.1	1	912-931	922	2	2	1
466	osa-miR164d_R-1	TGGAGAAGCAGGGCACGTGC	LOC_Os06g23650.1	1	795-814	805	0	3	3
467	osa-miR164d_R-1	TGGAGAAGCAGGGCACGTGC	LOC_Os06g46270.1	1	955-974	965	1	4	4
468	osa-miR164d_R-1	TGGAGAAGCAGGGCACGTGC	LOC_Os12g05260.1	3	203-222	213	2	2	2
469	osa-miR166a-3p	TCGGACCAGGCTTCATTCCCC	MSTRG.3879.1	4	366-385	376	2	2	0.666666667

470	osa-miR166a-3p	TCGGACCAGGCTTCATTCCCC	LOC_Os03g01890.2	3	1089-1109	1100	0	59	6
471	osa-miR166a-3p	TCGGACCAGGCTTCATTCCCC	MSTRG.13058.1	3	763-783	774	0	53	5.4
472	osa-miR166a-3p	TCGGACCAGGCTTCATTCCCC	MSTRG.13058.3	3	997-1017	1008	0	57	5.8
473	osa-miR166a-3p	TCGGACCAGGCTTCATTCCCC	LOC_Os03g01890.1	3	1089-1109	1100	0	57	5.8
474	osa-miR166a-3p	TCGGACCAGGCTTCATTCCCC	MSTRG.13058.2	3	1185-1205	1196	0	63	6.4
475	osa-miR166a-3p	TCGGACCAGGCTTCATTCCCC	LOC_Os04g48290.1	3	400-420	411	2	2	2
476	osa-miR166a-3p	TCGGACCAGGCTTCATTCCCC	LOC_Os10g33960.3	3	924-944	935	0	65	6.5
477	osa-miR166a-3p	TCGGACCAGGCTTCATTCCCC	LOC_Os10g33960.4	3	924-944	935	0	68	6.8
478	osa-miR166a-3p	TCGGACCAGGCTTCATTCCCC	LOC_Os10g33960.2	3	696-716	707	0	65	6.5
479	osa-miR166a-3p	TCGGACCAGGCTTCATTCCCC	LOC_Os10g33960.1	3	924-944	935	0	62	6.2
480	osa-miR166a-3p	TCGGACCAGGCTTCATTCCCC	MSTRG.5082.2	3	1060-1080	1071	0	63	6.3
481	osa-miR166a-3p	TCGGACCAGGCTTCATTCCCC	MSTRG.5082.3	3	1127-1147	1138	0	67	6.7
482	osa-miR166a-3p	TCGGACCAGGCTTCATTCCCC	MSTRG.9425.2	3	498-518	509	2	6	0.6
483	osa-miR166a-3p	TCGGACCAGGCTTCATTCCCC	LOC_Os12g41860.1	3	877-897	888	2	7	0.7
484	osa-miR166a-3p	TCGGACCAGGCTTCATTCCCC	LOC_Os01g08520.1	3.5	577-598	589	2	2	1
485	osa-miR166a-3p	TCGGACCAGGCTTCATTCCCC	MSTRG.528.1	3.5	1092-1113	1104	2	2	1
486	osa-miR166a-3p	TCGGACCAGGCTTCATTCCCC	MSTRG.15291.7	3	328-348	339	2	7	0.7
487	osa-miR166a-3p	TCGGACCAGGCTTCATTCCCC	MSTRG.15291.5	3	360-380	371	2	8	0.8
488	osa-miR166a-3p	TCGGACCAGGCTTCATTCCCC	MSTRG.15291.1	3	540-560	551	2	7	0.7
489	osa-miR166a-3p	TCGGACCAGGCTTCATTCCCC	MSTRG.15291.8	3	918-938	929	2	6	0.6
490	osa-miR166a-3p	TCGGACCAGGCTTCATTCCCC	LOC_Os03g43930.2	3	955-975	966	2	8	0.8
491	osa-miR166a-3p	TCGGACCAGGCTTCATTCCCC	LOC_Os03g43930.1	3	955-975	966	2	7	0.7
492	osa-miR166a-3p	TCGGACCAGGCTTCATTCCCC	MSTRG.15291.2	3	886-906	897	2	8	0.8
493	osa-miR166a-3p	TCGGACCAGGCTTCATTCCCC	MSTRG.15291.9	3	917-937	928	2	8	0.8
494	osa-miR166a-3p	TCGGACCAGGCTTCATTCCCC	MSTRG.24337.1	4	373-392	383	2	2	0.666666667
495	osa-miR166a-3p	TCGGACCAGGCTTCATTCCCC	MSTRG.7866.1	4	466-485	476	2	2	0.666666667
496	osa-miR166g-3p	TCGGACCAGGCTTCATTCCTC	LOC_Os03g01890.2	3	1089-1109	1100	0	59	6
497	osa-miR166g-3p	TCGGACCAGGCTTCATTCCTC	MSTRG.13058.1	3	763-783	774	0	53	5.4
498	osa-miR166g-3p	TCGGACCAGGCTTCATTCCTC	MSTRG.13058.3	3	997-1017	1008	0	57	5.8
499	osa-miR166g-3p	TCGGACCAGGCTTCATTCCTC	LOC_Os03g01890.1	3	1089-1109	1100	0	57	5.8
500	osa-miR166g-3p	TCGGACCAGGCTTCATTCCTC	MSTRG.13058.2	3	1185-1205	1196	0	63	6.4
501	osa-miR166g-3p	TCGGACCAGGCTTCATTCCTC	LOC_Os04g48290.1	3.5	400-420	411	2	2	2
502	osa-miR166g-3p	TCGGACCAGGCTTCATTCCTC	LOC_Os10g33960.3	3	924-944	935	0	65	6.5
503	osa-miR166g-3p	TCGGACCAGGCTTCATTCCTC	LOC_Os10g33960.4	3	924-944	935	0	68	6.8
504	osa-miR166g-3p	TCGGACCAGGCTTCATTCCTC	LOC_Os10g33960.2	3	696-716	707	0	65	6.5
505	osa-miR166g-3p	TCGGACCAGGCTTCATTCCTC	LOC_Os10g33960.1	3	924-944	935	0	62	6.2
506	osa-miR166g-3p	TCGGACCAGGCTTCATTCCTC	MSTRG.5082.2	3	1060-1080	1071	0	63	6.3
507	osa-miR166g-3p	TCGGACCAGGCTTCATTCCTC	MSTRG.5082.3	3	1127-1147	1138	0	67	6.7
508	osa-miR166g-3p	TCGGACCAGGCTTCATTCCTC	MSTRG.9425.2	3	498-518	509	2	6	0.6
509	osa-miR166g-3p	TCGGACCAGGCTTCATTCCTC	LOC_Os12g41860.1	3	877-897	888	2	7	0.7
510	osa-miR166g-3p	TCGGACCAGGCTTCATTCCTC	LOC_Os01g08520.1	4	577-598	589	2	2	1
511	osa-miR166g-3p	TCGGACCAGGCTTCATTCCTC	MSTRG.528.1	4	1092-1113	1104	2	2	1
512	osa-miR166g-3p	TCGGACCAGGCTTCATTCCTC	MSTRG.15291.7	3	328-348	339	2	7	0.7
513	osa-miR166g-3p	TCGGACCAGGCTTCATTCCTC	MSTRG.15291.5	3	360-380	371	2	8	0.8
514	osa-miR166g-3p	TCGGACCAGGCTTCATTCCTC	MSTRG.15291.1	3	540-560	551	2	7	0.7
515	osa-miR166g-3p	TCGGACCAGGCTTCATTCCTC	MSTRG.15291.8	3	918-938	929	2	6	0.6
516	osa-miR166g-3p	TCGGACCAGGCTTCATTCCTC	LOC_Os03g43930.2	3	955-975	966	2	8	0.8
517	osa-miR166g-3p	TCGGACCAGGCTTCATTCCTC	LOC_Os03g43930.1	3	955-975	966	2	7	0.7
518	osa-miR166g-3p	TCGGACCAGGCTTCATTCCTC	MSTRG.15291.2	3	886-906	897	2	8	0.8
519	osa-miR166g-3p	TCGGACCAGGCTTCATTCCTC	MSTRG.15291.9	3	917-937	928	2	8	0.8
520	osa-miR166k-3p	TCGGACCAGGCTTCAATCCCT	LOC_Os03g01890.2	2.5	1090-1109	1100	0	59	6
521	osa-miR166k-3p	TCGGACCAGGCTTCAATCCCT	MSTRG.13058.1	2.5	764-783	774	0	53	5.4
522	osa-miR166k-3p	TCGGACCAGGCTTCAATCCCT	MSTRG.13058.3	2.5	998-1017	1008	0	57	5.8
523	osa-miR166k-3p	TCGGACCAGGCTTCAATCCCT	LOC_Os03g01890.1	2.5	1090-1109	1100	0	57	5.8
524	osa-miR166k-3p	TCGGACCAGGCTTCAATCCCT	MSTRG.13058.2	2.5	1186-1205	1196	0	63	6.4
525	osa-miR166k-3p	TCGGACCAGGCTTCAATCCCT	LOC_Os10g33960.3	2.5	925-944	935	0	65	6.5
526	osa-miR166k-3p	TCGGACCAGGCTTCAATCCCT	LOC_Os10g33960.4	2.5	925-944	935	0	68	6.8
527	osa-miR166k-3p	TCGGACCAGGCTTCAATCCCT	LOC_Os10g33960.2	2.5	697-716	707	0	65	6.5
528	osa-miR166k-3p	TCGGACCAGGCTTCAATCCCT	LOC_Os10g33960.1	2.5	925-944	935	0	62	6.2

529	osa-miR166k-3p	TCGGACCAGGCTTCAATCCCT	MSTRG.5082.2	2.5	1061-1080	1071	0	63	6.3
530	osa-miR166k-3p	TCGGACCAGGCTTCAATCCCT	MSTRG.5082.3	2.5	1128-1147	1138	0	67	6.7
531	osa-miR166k-3p	TCGGACCAGGCTTCAATCCCT	MSTRG.9425.2	2.5	499-518	509	2	6	0.6
532	osa-miR166k-3p	TCGGACCAGGCTTCAATCCCT	LOC_Os12g41860.1	2.5	878-897	888	2	7	0.7
533	osa-miR166k-3p	TCGGACCAGGCTTCAATCCCT	LOC_Os01g08520.1	3	577-598	589	2	2	1
534	osa-miR166k-3p	TCGGACCAGGCTTCAATCCCT	MSTRG.528.1	3	1092-1113	1104	2	2	1
535	osa-miR166k-3p	TCGGACCAGGCTTCAATCCCT	MSTRG.15291.7	2.5	329-348	339	2	7	0.7
536	osa-miR166k-3p	TCGGACCAGGCTTCAATCCCT	MSTRG.15291.5	2.5	361-380	371	2	8	0.8
537	osa-miR166k-3p	TCGGACCAGGCTTCAATCCCT	MSTRG.15291.1	2.5	541-560	551	2	7	0.7
538	osa-miR166k-3p	TCGGACCAGGCTTCAATCCCT	MSTRG.15291.8	2.5	919-938	929	2	6	0.6
539	osa-miR166k-3p	TCGGACCAGGCTTCAATCCCT	LOC_Os03g43930.2	2.5	956-975	966	2	8	0.8
540	osa-miR166k-3p	TCGGACCAGGCTTCAATCCCT	LOC_Os03g43930.1	2.5	956-975	966	2	7	0.7
541	osa-miR166k-3p	TCGGACCAGGCTTCAATCCCT	MSTRG.15291.2	2.5	887-906	897	2	8	0.8
542	osa-miR166k-3p	TCGGACCAGGCTTCAATCCCT	MSTRG.15291.9	2.5	918-937	928	2	8	0.8
543	osa-miR166k-5p	GGTTTGTTGTCTGGCTCGAGG	MSTRG.4970.7	3.5	2823-2843	2834	4	1	0.1
544	osa-miR166k-5p	GGTTTGTTGTCTGGCTCGAGG	MSTRG.4970.14	3.5	6173-6193	6184	4	1	0.1
545	osa-miR166k-5p	GGTTTGTTGTCTGGCTCGAGG	MSTRG.4970.10	3.5	6544-6564	6555	4	1	0.1
546	osa-miR166k-5p	GGTTTGTTGTCTGGCTCGAGG	MSTRG.4970.11	3.5	6621-6641	6632	4	1	0.1
547	osa-miR166k-5p	GGTTTGTTGTCTGGCTCGAGG	MSTRG.4970.12	3.5	11404-11424	11415	4	1	0.1
548	osa-miR166k-5p	GGTTTGTTGTCTGGCTCGAGG	MSTRG.4970.2	3.5	11311-11331	11322	4	1	0.1
549	osa-miR166k-5p	GGTTTGTTGTCTGGCTCGAGG	MSTRG.4970.16	3.5	13634-13654	13645	4	1	0.1
550	osa-miR166k-5p	GGTTTGTTGTCTGGCTCGAGG	MSTRG.4970.1	3.5	15054-15074	15065	4	1	0.1
551	osa-miR166k-5p	GGTTTGTTGTCTGGCTCGAGG	MSTRG.4970.6	3.5	15106-15126	15117	4	1	0.1
552	osa-miR166k-5p	GGTTTGTTGTCTGGCTCGAGG	MSTRG.4970.3	3.5	15524-15544	15535	4	1	0.1
553	osa-miR166m_R-1	TCGGACCAGGCTTCATTCCC	LOC_Os03g01890.2	2	1090-1109	1100	0	59	6
554	osa-miR166m_R-1	TCGGACCAGGCTTCATTCCC	MSTRG.13058.1	2	764-783	774	0	53	5.4
555	osa-miR166m_R-1	TCGGACCAGGCTTCATTCCC	MSTRG.13058.3	2	998-1017	1008	0	57	5.8
556	osa-miR166m_R-1	TCGGACCAGGCTTCATTCCC	LOC_Os03g01890.1	2	1090-1109	1100	0	57	5.8
557	osa-miR166m_R-1	TCGGACCAGGCTTCATTCCC	MSTRG.13058.2	2	1186-1205	1196	0	63	6.4
558	osa-miR166m_R-1	TCGGACCAGGCTTCATTCCC	LOC_Os04g48290.1	3	401-420	411	2	2	2
559	osa-miR166m_R-1	TCGGACCAGGCTTCATTCCC	LOC_Os10g33960.3	2	925-944	935	0	65	6.5
560	osa-miR166m_R-1	TCGGACCAGGCTTCATTCCC	LOC_Os10g33960.4	2	925-944	935	0	68	6.8
561	osa-miR166m_R-1	TCGGACCAGGCTTCATTCCC	LOC_Os10g33960.2	2	697-716	707	0	65	6.5
562	osa-miR166m_R-1	TCGGACCAGGCTTCATTCCC	LOC_Os10g33960.1	2	925-944	935	0	62	6.2
563	osa-miR166m_R-1	TCGGACCAGGCTTCATTCCC	MSTRG.5082.2	2	1061-1080	1071	0	63	6.3
564	osa-miR166m_R-1	TCGGACCAGGCTTCATTCCC	MSTRG.5082.3	2	1128-1147	1138	0	67	6.7
565	osa-miR166m_R-1	TCGGACCAGGCTTCATTCCC	MSTRG.9425.2	2	499-518	509	2	6	0.6
566	osa-miR166m_R-1	TCGGACCAGGCTTCATTCCC	LOC_Os12g41860.1	2	878-897	888	2	7	0.7
567	osa-miR166m_R-1	TCGGACCAGGCTTCATTCCC	MSTRG.15291.7	2	329-348	339	2	7	0.7
568	osa-miR166m_R-1	TCGGACCAGGCTTCATTCCC	MSTRG.15291.5	2	361-380	371	2	8	0.8
569	osa-miR166m_R-1	TCGGACCAGGCTTCATTCCC	MSTRG.15291.1	2	541-560	551	2	7	0.7
570	osa-miR166m_R-1	TCGGACCAGGCTTCATTCCC	MSTRG.15291.8	2	919-938	929	2	6	0.6
571	osa-miR166m_R-1	TCGGACCAGGCTTCATTCCC	LOC_Os03g43930.2	2	956-975	966	2	8	0.8
572	osa-miR166m_R-1	TCGGACCAGGCTTCATTCCC	LOC_Os03g43930.1	2	956-975	966	2	7	0.7
573	osa-miR166m_R-1	TCGGACCAGGCTTCATTCCC	MSTRG.15291.2	2	887-906	897	2	8	0.8
574	osa-miR166m_R-1	TCGGACCAGGCTTCATTCCC	MSTRG.15291.9	2	918-937	928	2	8	0.8
575	osa-miR167a-5p	TGAAGCTGCCAGCATGATCTA	MSTRG.19259.4	4	2533-2554	2544	2	5	0.714285714
576	osa-miR167a-5p	TGAAGCTGCCAGCATGATCTA	MSTRG.19259.7	4	2617-2638	2628	2	5	0.714285714
577	osa-miR167a-5p	TGAAGCTGCCAGCATGATCTA	MSTRG.19259.3	4	2634-2655	2645	2	5	0.714285714
578	osa-miR167a-5p	TGAAGCTGCCAGCATGATCTA	MSTRG.19259.5	4	2639-2660	2650	2	5	0.714285714
579	osa-miR167a-5p	TGAAGCTGCCAGCATGATCTA	LOC_Os04g57610.3	4	2646-2667	2657	2	5	0.714285714
580	osa-miR167a-5p	TGAAGCTGCCAGCATGATCTA	LOC_Os04g57610.1	4	2658-2679	2669	2	5	0.714285714
581	osa-miR167a-5p	TGAAGCTGCCAGCATGATCTA	MSTRG.19259.6	4	2726-2747	2737	2	5	0.714285714
582	osa-miR167a-5p	TGAAGCTGCCAGCATGATCTA	LOC_Os07g33790.1	3	3420-3439	3430	2	2	0.666666667
583	osa-miR167a-5p	TGAAGCTGCCAGCATGATCTA	MSTRG.25835.3	3	3371-3390	3381	2	2	0.666666667
584	osa-miR167a-5p	TGAAGCTGCCAGCATGATCTA	MSTRG.25835.2	3	3397-3416	3407	2	2	0.666666667
585	osa-miR167a-5p	TGAAGCTGCCAGCATGATCTA	LOC_Os09g39420.1	4	2909-2929	2920	2	8	8
586	osa-miR167d-3p	GATCATGCTGTGCAGTTTCATC	LOC_Os02g12810.3	3.5	1564-1585	1576	2	2	0.333333333
587	osa-miR167d-3p	GATCATGCTGTGCAGTTTCATC	LOC_Os02g12810.4	3.5	1309-1330	1321	2	2	0.333333333

588	osa-miR167d-3p	GATCATGCTGTGCAGTTTCATC	LOC_Os02g12810.1	3.5	1564-1585	1576	2	2	0.333333333
589	osa-miR167d-3p	GATCATGCTGTGCAGTTTCATC	MSTRG.10436.1	3.5	1607-1628	1619	2	2	0.333333333
590	osa-miR167d-3p	GATCATGCTGTGCAGTTTCATC	MSTRG.10436.2	3.5	1287-1308	1299	2	2	0.333333333
591	osa-miR167d-3p	GATCATGCTGTGCAGTTTCATC	LOC_Os02g12810.2	3.5	1809-1830	1821	2	2	0.333333333
592	osa-miR167d-5p	TGAAGCTGCCAGCATGATCTG	LOC_Os07g33790.1	3.5	3420-3439	3430	2	2	0.666666667
593	osa-miR167d-5p	TGAAGCTGCCAGCATGATCTG	MSTRG.25835.3	3.5	3371-3390	3381	2	2	0.666666667
594	osa-miR167d-5p	TGAAGCTGCCAGCATGATCTG	MSTRG.25835.2	3.5	3397-3416	3407	2	2	0.666666667
595	osa-miR167d-5p	TGAAGCTGCCAGCATGATCTG	LOC_Os09g39420.1	4	2909-2929	2920	2	8	8
596	osa-miR167e-3p	AGATCATGTTGCAGCTTCACT	LOC_Os08g12760.3	3.5	1450-1470	1461	2	2	0.5
597	osa-miR167e-3p	AGATCATGTTGCAGCTTCACT	LOC_Os08g12760.4	3.5	1472-1492	1483	2	2	0.5
598	osa-miR167e-3p	AGATCATGTTGCAGCTTCACT	LOC_Os08g12760.2	3.5	1504-1524	1515	2	2	0.5
599	osa-miR167e-3p	AGATCATGTTGCAGCTTCACT	LOC_Os08g12760.1	3.5	1525-1545	1536	2	2	0.5
600	osa-miR169b	CAGCCAAGGATGACTTGCCGG	LOC_Os03g07880.3	3.5	756-776	767	2	15	5
601	osa-miR169b	CAGCCAAGGATGACTTGCCGG	LOC_Os03g07880.1	3.5	844-864	855	2	15	5
602	osa-miR169b	CAGCCAAGGATGACTTGCCGG	LOC_Os03g07880.2	3.5	993-1013	1004	2	15	5
603	osa-miR169b	CAGCCAAGGATGACTTGCCGG	MSTRG.14822.4	3.5	1248-1268	1259	0	11	1.833333333
604	osa-miR169b	CAGCCAAGGATGACTTGCCGG	MSTRG.14822.5	3.5	1255-1275	1266	0	11	1.833333333
605	osa-miR169b	CAGCCAAGGATGACTTGCCGG	LOC_Os03g29760.3	3.5	1264-1284	1275	0	11	1.833333333
606	osa-miR169b	CAGCCAAGGATGACTTGCCGG	LOC_Os03g29760.1	3.5	1284-1304	1295	0	11	1.833333333
607	osa-miR169b	CAGCCAAGGATGACTTGCCGG	LOC_Os03g29760.2	3.5	1364-1384	1375	0	11	1.833333333
608	osa-miR169b	CAGCCAAGGATGACTTGCCGG	MSTRG.14822.1	3.5	2759-2779	2770	2	11	1.833333333
609	osa-miR169b	CAGCCAAGGATGACTTGCCGG	LOC_Os07g41720.1	3.5	1169-1189	1180	2	7	3.5
610	osa-miR169b	CAGCCAAGGATGACTTGCCGG	LOC_Os07g41720.2	3.5	1302-1322	1313	2	7	3.5
611	osa-miR169b	CAGCCAAGGATGACTTGCCGG	LOC_Os12g42400.2	3.5	1202-1222	1213	0	59	19.66666667
612	osa-miR169b	CAGCCAAGGATGACTTGCCGG	LOC_Os12g42400.3	3.5	1247-1267	1258	0	59	19.66666667
613	osa-miR169b	CAGCCAAGGATGACTTGCCGG	LOC_Os12g42400.1	3.5	1256-1276	1267	0	59	19.66666667
614	osa-miR169b	CAGCCAAGGATGACTTGCCGG	LOC_Os03g48970.4	4	1228-1248	1239	2	113	18.83333333
615	osa-miR169b	CAGCCAAGGATGACTTGCCGG	LOC_Os03g48970.1	4	1236-1256	1247	2	113	18.83333333
616	osa-miR169b	CAGCCAAGGATGACTTGCCGG	LOC_Os03g48970.2	4	1765-1785	1776	2	113	18.83333333
617	osa-miR169b	CAGCCAAGGATGACTTGCCGG	MSTRG.15580.1	4	1873-1893	1884	2	113	18.83333333
618	osa-miR169b	CAGCCAAGGATGACTTGCCGG	LOC_Os03g48970.3	4	1935-1955	1946	2	113	18.83333333
619	osa-miR169b	CAGCCAAGGATGACTTGCCGG	MSTRG.15580.2	4	2753-2773	2764	2	113	18.83333333
620	osa-MIR169c-p3	GGCAAGTCTGTCCTTGGCTAC	LOC_Os08g36450.1	3.5	1569-1589	1580	2	3	1.5
621	osa-MIR169c-p3	GGCAAGTCTGTCCTTGGCTAC	MSTRG.28396.2	3.5	1127-1147	1138	2	3	1.5
622	osa-miR169e	TAGCCAAGGATGACTTGCCGG	LOC_Os03g07880.3	3.5	756-776	767	2	15	5
623	osa-miR169e	TAGCCAAGGATGACTTGCCGG	LOC_Os03g07880.1	3.5	844-864	855	2	15	5
624	osa-miR169e	TAGCCAAGGATGACTTGCCGG	LOC_Os03g07880.2	3.5	993-1013	1004	2	15	5
625	osa-miR169e	TAGCCAAGGATGACTTGCCGG	MSTRG.14822.4	3.5	1248-1268	1259	0	11	1.833333333
626	osa-miR169e	TAGCCAAGGATGACTTGCCGG	MSTRG.14822.5	3.5	1255-1275	1266	0	11	1.833333333
627	osa-miR169e	TAGCCAAGGATGACTTGCCGG	LOC_Os03g29760.3	3.5	1264-1284	1275	0	11	1.833333333
628	osa-miR169e	TAGCCAAGGATGACTTGCCGG	LOC_Os03g29760.1	3.5	1284-1304	1295	0	11	1.833333333
629	osa-miR169e	TAGCCAAGGATGACTTGCCGG	LOC_Os03g29760.2	3.5	1364-1384	1375	0	11	1.833333333
630	osa-miR169e	TAGCCAAGGATGACTTGCCGG	MSTRG.14822.1	3.5	2759-2779	2770	2	11	1.833333333
631	osa-miR169e	TAGCCAAGGATGACTTGCCGG	LOC_Os07g41720.1	3.5	1169-1189	1180	2	7	3.5
632	osa-miR169e	TAGCCAAGGATGACTTGCCGG	LOC_Os07g41720.2	3.5	1302-1322	1313	2	7	3.5
633	osa-miR169e	TAGCCAAGGATGACTTGCCGG	LOC_Os12g42400.2	4	1202-1222	1213	0	59	19.66666667
634	osa-miR169e	TAGCCAAGGATGACTTGCCGG	LOC_Os12g42400.3	4	1247-1267	1258	0	59	19.66666667
635	osa-miR169e	TAGCCAAGGATGACTTGCCGG	LOC_Os12g42400.1	4	1256-1276	1267	0	59	19.66666667
636	osa-miR169e	TAGCCAAGGATGACTTGCCGG	LOC_Os03g48970.4	4	1228-1248	1239	2	113	18.83333333
637	osa-miR169e	TAGCCAAGGATGACTTGCCGG	LOC_Os03g48970.1	4	1236-1256	1247	2	113	18.83333333
638	osa-miR169e	TAGCCAAGGATGACTTGCCGG	LOC_Os03g48970.2	4	1765-1785	1776	2	113	18.83333333
639	osa-miR169e	TAGCCAAGGATGACTTGCCGG	MSTRG.15580.1	4	1873-1893	1884	2	113	18.83333333
640	osa-miR169e	TAGCCAAGGATGACTTGCCGG	LOC_Os03g48970.3	4	1935-1955	1946	2	113	18.83333333
641	osa-miR169e	TAGCCAAGGATGACTTGCCGG	MSTRG.15580.2	4	2753-2773	2764	2	113	18.83333333
642	osa-MIR169g-p3	GGCAGTCTCCTTGGCTAGC	LOC_Os01g55020.1	4	141-158	149	0	13	1.444444444
643	osa-MIR169g-p3	GGCAGTCTCCTTGGCTAGC	LOC_Os01g55020.3	4	141-158	149	2	13	1.444444444
644	osa-MIR169g-p3	GGCAGTCTCCTTGGCTAGC	LOC_Os01g55020.5	4	141-158	149	2	13	1.444444444
645	osa-MIR169g-p3	GGCAGTCTCCTTGGCTAGC	LOC_Os01g55020.2	4	141-158	149	2	13	1.444444444
646	osa-MIR169g-p3	GGCAGTCTCCTTGGCTAGC	MSTRG.2766.1	4	193-210	201	2	13	1.444444444

647	osa-MIR169g-p3	GGCAGTCTCCTTGGCTAGC	LOC_Os01g55020.4	4	169-186	177	2	13	1.444444444
648	osa-MIR169g-p3	GGCAGTCTCCTTGGCTAGC	LOC_Os01g55020.6	4	141-158	149	2	13	1.444444444
649	osa-MIR169g-p3	GGCAGTCTCCTTGGCTAGC	MSTRG.2766.3	4	180-197	188	2	13	1.444444444
650	osa-MIR169g-p3	GGCAGTCTCCTTGGCTAGC	MSTRG.2766.2	4	186-203	194	2	13	1.444444444
651	osa-MIR169g-p3	GGCAGTCTCCTTGGCTAGC	MSTRG.3753.1	4	864-882	873	2	4	4
652	osa-MIR169g-p3	GGCAGTCTCCTTGGCTAGC	LOC_Os02g24330.1	3.5	1400-1417	1408	2	2	1
653	osa-MIR169g-p3	GGCAGTCTCCTTGGCTAGC	LOC_Os02g24330.2	3.5	3307-3324	3315	2	2	1
654	osa-MIR169g-p3	GGCAGTCTCCTTGGCTAGC	LOC_Os03g04400.1	4	825-842	833	2	22	22
655	osa-MIR169g-p3	GGCAGTCTCCTTGGCTAGC	LOC_Os03g08010.1	3	961-978	969	2	39	4.05
656	osa-MIR169g-p3	GGCAGTCTCCTTGGCTAGC	LOC_Os03g08020.1	3	961-978	969	2	40	4
657	osa-MIR169g-p3	GGCAGTCTCCTTGGCTAGC	LOC_Os03g08050.1	3	961-978	969	2	40	4
658	osa-MIR169g-p3	GGCAGTCTCCTTGGCTAGC	MSTRG.13491.8	3	1081-1098	1089	2	37	3.7
659	osa-MIR169g-p3	GGCAGTCTCCTTGGCTAGC	MSTRG.13491.13	3	1068-1085	1076	2	34	3.55
660	osa-MIR169g-p3	GGCAGTCTCCTTGGCTAGC	MSTRG.13491.7	3	1081-1098	1089	2	40	4
661	osa-MIR169g-p3	GGCAGTCTCCTTGGCTAGC	MSTRG.13491.10	3	1160-1177	1168	2	43	4.3
662	osa-MIR169g-p3	GGCAGTCTCCTTGGCTAGC	MSTRG.13491.11	3	1160-1177	1168	2	37	3.85
663	osa-MIR169g-p3	GGCAGTCTCCTTGGCTAGC	MSTRG.13491.3	3	1167-1184	1175	2	39	3.9
664	osa-MIR169g-p3	GGCAGTCTCCTTGGCTAGC	MSTRG.13491.6	3	1168-1185	1176	2	41	4.1
665	osa-MIR169g-p3	GGCAGTCTCCTTGGCTAGC	MSTRG.13491.2	3	1167-1184	1175	2	38	3.8
666	osa-MIR169g-p3	GGCAGTCTCCTTGGCTAGC	MSTRG.13491.1	3	1167-1184	1175	2	38	3.95
667	osa-MIR169g-p3	GGCAGTCTCCTTGGCTAGC	MSTRG.13491.4	3	1159-1176	1167	2	38	3.8
668	osa-MIR169g-p3	GGCAGTCTCCTTGGCTAGC	LOC_Os05g28290.1	3.5	760-777	768	2	34	34
669	osa-MIR169g-p3	GGCAGTCTCCTTGGCTAGC	LOC_Os10g34660.1	3.5	701-720	711	4	1	0.5
670	osa-MIR169g-p3	GGCAGTCTCCTTGGCTAGC	MSTRG.5115.1	3.5	725-744	735	4	1	0.5
671	osa-MIR169g-p3	GGCAGTCTCCTTGGCTAGC	LOC_Os03g49140.1	3	208-225	216	4	1	0.333333333
672	osa-MIR169g-p3	GGCAGTCTCCTTGGCTAGC	MSTRG.15582.1	3	1526-1543	1534	4	1	0.333333333
673	osa-MIR169g-p3	GGCAGTCTCCTTGGCTAGC	MSTRG.15582.2	3	1442-1459	1450	4	1	0.333333333
674	osa-MIR169g-p3	GGCAGTCTCCTTGGCTAGC	MSTRG.27813.3	2	905-923	914	2	4	1.333333333
675	osa-MIR169g-p3	GGCAGTCTCCTTGGCTAGC	LOC_Os08g23710.1	2	836-854	845	2	4	1.333333333
676	osa-MIR169g-p3	GGCAGTCTCCTTGGCTAGC	MSTRG.27813.2	2	1088-1106	1097	2	4	1.333333333
677	osa-miR169h	TAGCCAAGGATGACTTGCCTG	LOC_Os03g07880.3	3	755-776	767	2	15	5
678	osa-miR169h	TAGCCAAGGATGACTTGCCTG	LOC_Os03g07880.1	3	843-864	855	2	15	5
679	osa-miR169h	TAGCCAAGGATGACTTGCCTG	LOC_Os03g07880.2	3	992-1013	1004	2	15	5
680	osa-miR169h	TAGCCAAGGATGACTTGCCTG	MSTRG.14822.4	3.5	1247-1268	1259	0	11	1.833333333
681	osa-miR169h	TAGCCAAGGATGACTTGCCTG	MSTRG.14822.5	3.5	1254-1275	1266	0	11	1.833333333
682	osa-miR169h	TAGCCAAGGATGACTTGCCTG	LOC_Os03g29760.3	3.5	1263-1284	1275	0	11	1.833333333
683	osa-miR169h	TAGCCAAGGATGACTTGCCTG	LOC_Os03g29760.1	3.5	1283-1304	1295	0	11	1.833333333
684	osa-miR169h	TAGCCAAGGATGACTTGCCTG	LOC_Os03g29760.2	3.5	1363-1384	1375	0	11	1.833333333
685	osa-miR169h	TAGCCAAGGATGACTTGCCTG	MSTRG.14822.1	3.5	2758-2779	2770	2	11	1.833333333
686	osa-miR169h	TAGCCAAGGATGACTTGCCTG	LOC_Os07g41720.1	4	1169-1189	1180	2	7	3.5
687	osa-miR169h	TAGCCAAGGATGACTTGCCTG	LOC_Os07g41720.2	4	1302-1322	1313	2	7	3.5
688	osa-miR169h	TAGCCAAGGATGACTTGCCTG	LOC_Os12g42400.2	3	1202-1222	1213	0	59	19.66666667
689	osa-miR169h	TAGCCAAGGATGACTTGCCTG	LOC_Os12g42400.3	3	1247-1267	1258	0	59	19.66666667
690	osa-miR169h	TAGCCAAGGATGACTTGCCTG	LOC_Os12g42400.1	3	1256-1276	1267	0	59	19.66666667
691	osa-miR169h	TAGCCAAGGATGACTTGCCTG	LOC_Os03g48970.4	3	1228-1248	1239	2	113	18.83333333
692	osa-miR169h	TAGCCAAGGATGACTTGCCTG	LOC_Os03g48970.1	3	1236-1256	1247	2	113	18.83333333
693	osa-miR169h	TAGCCAAGGATGACTTGCCTG	LOC_Os03g48970.2	3	1765-1785	1776	2	113	18.83333333
694	osa-miR169h	TAGCCAAGGATGACTTGCCTG	MSTRG.15580.1	3	1873-1893	1884	2	113	18.83333333
695	osa-miR169h	TAGCCAAGGATGACTTGCCTG	LOC_Os03g48970.3	3	1935-1955	1946	2	113	18.83333333
696	osa-miR169h	TAGCCAAGGATGACTTGCCTG	MSTRG.15580.2	3	2753-2773	2764	2	113	18.83333333
697	osa-miR169h	TAGCCAAGGATGACTTGCCTG	LOC_Os03g44540.1	3.5	1718-1738	1729	0	11	11
698	osa-MIR169h-p3	GGCAGTCTCCTTGGCTAGC	LOC_Os01g55020.1	4	141-158	149	0	13	1.444444444
699	osa-MIR169h-p3	GGCAGTCTCCTTGGCTAGC	LOC_Os01g55020.3	4	141-158	149	2	13	1.444444444
700	osa-MIR169h-p3	GGCAGTCTCCTTGGCTAGC	LOC_Os01g55020.5	4	141-158	149	2	13	1.444444444
701	osa-MIR169h-p3	GGCAGTCTCCTTGGCTAGC	LOC_Os01g55020.2	4	141-158	149	2	13	1.444444444
702	osa-MIR169h-p3	GGCAGTCTCCTTGGCTAGC	MSTRG.2766.1	4	193-210	201	2	13	1.444444444
703	osa-MIR169h-p3	GGCAGTCTCCTTGGCTAGC	LOC_Os01g55020.4	4	169-186	177	2	13	1.444444444
704	osa-MIR169h-p3	GGCAGTCTCCTTGGCTAGC	LOC_Os01g55020.6	4	141-158	149	2	13	1.444444444
705	osa-MIR169h-p3	GGCAGTCTCCTTGGCTAGC	MSTRG.2766.3	4	180-197	188	2	13	1.444444444

706	osa-MIR169h-p3	GGCAGTCTCCTTGGCTAGC	MSTRG.2766.2	4	186-203	194	2	13	1.444444444
707	osa-MIR169h-p3	GGCAGTCTCCTTGGCTAGC	MSTRG.3753.1	4	864-882	873	2	4	4
708	osa-MIR169h-p3	GGCAGTCTCCTTGGCTAGC	LOC_Os02g24330.1	3.5	1400-1417	1408	2	2	1
709	osa-MIR169h-p3	GGCAGTCTCCTTGGCTAGC	LOC_Os02g24330.2	3.5	3307-3324	3315	2	2	1
710	osa-MIR169h-p3	GGCAGTCTCCTTGGCTAGC	LOC_Os03g04400.1	4	825-842	833	2	22	22
711	osa-MIR169h-p3	GGCAGTCTCCTTGGCTAGC	LOC_Os03g08050.1	3	961-978	969	2	40	4
712	osa-MIR169h-p3	GGCAGTCTCCTTGGCTAGC	LOC_Os03g08010.1	3	961-978	969	2	39	4.05
713	osa-MIR169h-p3	GGCAGTCTCCTTGGCTAGC	LOC_Os03g08020.1	3	961-978	969	2	40	4
714	osa-MIR169h-p3	GGCAGTCTCCTTGGCTAGC	MSTRG.13491.8	3	1081-1098	1089	2	37	3.7
715	osa-MIR169h-p3	GGCAGTCTCCTTGGCTAGC	MSTRG.13491.13	3	1068-1085	1076	2	34	3.55
716	osa-MIR169h-p3	GGCAGTCTCCTTGGCTAGC	MSTRG.13491.7	3	1081-1098	1089	2	40	4
717	osa-MIR169h-p3	GGCAGTCTCCTTGGCTAGC	MSTRG.13491.10	3	1160-1177	1168	2	43	4.3
718	osa-MIR169h-p3	GGCAGTCTCCTTGGCTAGC	MSTRG.13491.11	3	1160-1177	1168	2	37	3.85
719	osa-MIR169h-p3	GGCAGTCTCCTTGGCTAGC	MSTRG.13491.3	3	1167-1184	1175	2	39	3.9
720	osa-MIR169h-p3	GGCAGTCTCCTTGGCTAGC	MSTRG.13491.6	3	1168-1185	1176	2	41	4.1
721	osa-MIR169h-p3	GGCAGTCTCCTTGGCTAGC	MSTRG.13491.2	3	1167-1184	1175	2	38	3.8
722	osa-MIR169h-p3	GGCAGTCTCCTTGGCTAGC	MSTRG.13491.1	3	1167-1184	1175	2	38	3.95
723	osa-MIR169h-p3	GGCAGTCTCCTTGGCTAGC	MSTRG.13491.4	3	1159-1176	1167	2	38	3.8
724	osa-MIR169h-p3	GGCAGTCTCCTTGGCTAGC	LOC_Os05g28290.1	3.5	760-777	768	2	34	34
725	osa-MIR169h-p3	GGCAGTCTCCTTGGCTAGC	LOC_Os10g34660.1	3.5	701-720	711	4	1	0.5
726	osa-MIR169h-p3	GGCAGTCTCCTTGGCTAGC	MSTRG.5115.1	3.5	725-744	735	4	1	0.5
727	osa-MIR169h-p3	GGCAGTCTCCTTGGCTAGC	LOC_Os03g49140.1	3	208-225	216	4	1	0.333333333
728	osa-MIR169h-p3	GGCAGTCTCCTTGGCTAGC	MSTRG.15582.1	3	1526-1543	1534	4	1	0.333333333
729	osa-MIR169h-p3	GGCAGTCTCCTTGGCTAGC	MSTRG.15582.2	3	1442-1459	1450	4	1	0.333333333
730	osa-MIR169h-p3	GGCAGTCTCCTTGGCTAGC	MSTRG.27813.3	2	905-923	914	2	4	1.333333333
731	osa-MIR169h-p3	GGCAGTCTCCTTGGCTAGC	LOC_Os08g23710.1	2	836-854	845	2	4	1.333333333
732	osa-MIR169h-p3	GGCAGTCTCCTTGGCTAGC	MSTRG.27813.2	2	1088-1106	1097	2	4	1.333333333
733	osa-MIR169i-p3	GGCAGTCTCCTTGGCTAGT	MSTRG.3753.1	4	864-882	873	2	4	4
734	osa-MIR169i-p3	GGCAGTCTCCTTGGCTAGT	LOC_Os02g24330.1	4	1400-1417	1408	2	2	1
735	osa-MIR169i-p3	GGCAGTCTCCTTGGCTAGT	LOC_Os02g24330.2	4	3307-3324	3315	2	2	1
736	osa-MIR169i-p3	GGCAGTCTCCTTGGCTAGT	LOC_Os03g08020.1	3	961-978	969	2	40	4
737	osa-MIR169i-p3	GGCAGTCTCCTTGGCTAGT	LOC_Os03g08010.1	3	961-978	969	2	39	4.05
738	osa-MIR169i-p3	GGCAGTCTCCTTGGCTAGT	LOC_Os03g08050.1	3	961-978	969	2	40	4
739	osa-MIR169i-p3	GGCAGTCTCCTTGGCTAGT	MSTRG.13491.8	3	1081-1098	1089	2	37	3.7
740	osa-MIR169i-p3	GGCAGTCTCCTTGGCTAGT	MSTRG.13491.13	3	1068-1085	1076	2	34	3.55
741	osa-MIR169i-p3	GGCAGTCTCCTTGGCTAGT	MSTRG.13491.7	3	1081-1098	1089	2	40	4
742	osa-MIR169i-p3	GGCAGTCTCCTTGGCTAGT	MSTRG.13491.10	3	1160-1177	1168	2	43	4.3
743	osa-MIR169i-p3	GGCAGTCTCCTTGGCTAGT	MSTRG.13491.11	3	1160-1177	1168	2	37	3.85
744	osa-MIR169i-p3	GGCAGTCTCCTTGGCTAGT	MSTRG.13491.3	3	1167-1184	1175	2	39	3.9
745	osa-MIR169i-p3	GGCAGTCTCCTTGGCTAGT	MSTRG.13491.6	3	1168-1185	1176	2	41	4.1
746	osa-MIR169i-p3	GGCAGTCTCCTTGGCTAGT	MSTRG.13491.2	3	1167-1184	1175	2	38	3.8
747	osa-MIR169i-p3	GGCAGTCTCCTTGGCTAGT	MSTRG.13491.1	3	1167-1184	1175	2	38	3.95
748	osa-MIR169i-p3	GGCAGTCTCCTTGGCTAGT	MSTRG.13491.4	3	1159-1176	1167	2	38	3.8
749	osa-MIR169i-p3	GGCAGTCTCCTTGGCTAGT	LOC_Os05g28290.1	4	760-777	768	2	34	34
750	osa-MIR169i-p3	GGCAGTCTCCTTGGCTAGT	LOC_Os07g29630.1	4	639-657	648	2	2	1
751	osa-MIR169i-p3	GGCAGTCTCCTTGGCTAGT	MSTRG.25637.1	4	696-714	705	2	2	1
752	osa-MIR169i-p3	GGCAGTCTCCTTGGCTAGT	LOC_Os10g34660.1	4	701-720	711	4	1	0.5
753	osa-MIR169i-p3	GGCAGTCTCCTTGGCTAGT	MSTRG.5115.1	4	725-744	735	4	1	0.5
754	osa-MIR169i-p3	GGCAGTCTCCTTGGCTAGT	MSTRG.684.3	4	1453-1470	1461	4	1	0.2
755	osa-MIR169i-p3	GGCAGTCTCCTTGGCTAGT	MSTRG.684.1	4	1718-1735	1726	4	1	0.2
756	osa-MIR169i-p3	GGCAGTCTCCTTGGCTAGT	LOC_Os01g11070.1	4	1870-1887	1878	4	1	0.2
757	osa-MIR169i-p3	GGCAGTCTCCTTGGCTAGT	MSTRG.684.4	4	2293-2310	2301	4	1	0.2
758	osa-MIR169i-p3	GGCAGTCTCCTTGGCTAGT	MSTRG.684.5	4	2399-2416	2407	4	1	0.2
759	osa-MIR169i-p3	GGCAGTCTCCTTGGCTAGT	LOC_Os03g49140.1	3.5	208-225	216	4	1	0.333333333
760	osa-MIR169i-p3	GGCAGTCTCCTTGGCTAGT	MSTRG.15582.1	3.5	1526-1543	1534	4	1	0.333333333
761	osa-MIR169i-p3	GGCAGTCTCCTTGGCTAGT	MSTRG.15582.2	3.5	1442-1459	1450	4	1	0.333333333
762	osa-MIR169i-p3	GGCAGTCTCCTTGGCTAGT	MSTRG.27813.3	2.5	905-923	914	2	4	1.333333333
763	osa-MIR169i-p3	GGCAGTCTCCTTGGCTAGT	LOC_Os08g23710.1	2.5	836-854	845	2	4	1.333333333
764	osa-MIR169i-p3	GGCAGTCTCCTTGGCTAGT	MSTRG.27813.2	2.5	1088-1106	1097	2	4	1.333333333

765	osa-MIR169j-p3	GGCAGTCTCCTTGGCTAGC	LOC_Os01g55020.1	4	141-158	149	0	13	1.444444444
766	osa-MIR169j-p3	GGCAGTCTCCTTGGCTAGC	LOC_Os01g55020.3	4	141-158	149	2	13	1.444444444
767	osa-MIR169j-p3	GGCAGTCTCCTTGGCTAGC	LOC_Os01g55020.5	4	141-158	149	2	13	1.444444444
768	osa-MIR169j-p3	GGCAGTCTCCTTGGCTAGC	LOC_Os01g55020.2	4	141-158	149	2	13	1.444444444
769	osa-MIR169j-p3	GGCAGTCTCCTTGGCTAGC	MSTRG.2766.1	4	193-210	201	2	13	1.444444444
770	osa-MIR169j-p3	GGCAGTCTCCTTGGCTAGC	LOC_Os01g55020.4	4	169-186	177	2	13	1.444444444
771	osa-MIR169j-p3	GGCAGTCTCCTTGGCTAGC	LOC_Os01g55020.6	4	141-158	149	2	13	1.444444444
772	osa-MIR169j-p3	GGCAGTCTCCTTGGCTAGC	MSTRG.2766.3	4	180-197	188	2	13	1.444444444
773	osa-MIR169j-p3	GGCAGTCTCCTTGGCTAGC	MSTRG.2766.2	4	186-203	194	2	13	1.444444444
774	osa-MIR169j-p3	GGCAGTCTCCTTGGCTAGC	MSTRG.3753.1	4	864-882	873	2	4	4
775	osa-MIR169j-p3	GGCAGTCTCCTTGGCTAGC	LOC_Os02g24330.1	3.5	1400-1417	1408	2	2	1
776	osa-MIR169j-p3	GGCAGTCTCCTTGGCTAGC	LOC_Os02g24330.2	3.5	3307-3324	3315	2	2	1
777	osa-MIR169j-p3	GGCAGTCTCCTTGGCTAGC	LOC_Os03g04400.1	4	825-842	833	2	22	22
778	osa-MIR169j-p3	GGCAGTCTCCTTGGCTAGC	LOC_Os03g08020.1	3	961-978	969	2	40	4
779	osa-MIR169j-p3	GGCAGTCTCCTTGGCTAGC	LOC_Os03g08010.1	3	961-978	969	2	39	4.05
780	osa-MIR169j-p3	GGCAGTCTCCTTGGCTAGC	LOC_Os03g08050.1	3	961-978	969	2	40	4
781	osa-MIR169j-p3	GGCAGTCTCCTTGGCTAGC	MSTRG.13491.8	3	1081-1098	1089	2	37	3.7
782	osa-MIR169j-p3	GGCAGTCTCCTTGGCTAGC	MSTRG.13491.13	3	1068-1085	1076	2	34	3.55
783	osa-MIR169j-p3	GGCAGTCTCCTTGGCTAGC	MSTRG.13491.7	3	1081-1098	1089	2	40	4
784	osa-MIR169j-p3	GGCAGTCTCCTTGGCTAGC	MSTRG.13491.10	3	1160-1177	1168	2	43	4.3
785	osa-MIR169j-p3	GGCAGTCTCCTTGGCTAGC	MSTRG.13491.11	3	1160-1177	1168	2	37	3.85
786	osa-MIR169j-p3	GGCAGTCTCCTTGGCTAGC	MSTRG.13491.3	3	1167-1184	1175	2	39	3.9
787	osa-MIR169j-p3	GGCAGTCTCCTTGGCTAGC	MSTRG.13491.6	3	1168-1185	1176	2	41	4.1
788	osa-MIR169j-p3	GGCAGTCTCCTTGGCTAGC	MSTRG.13491.1	3	1167-1184	1175	2	38	3.95
789	osa-MIR169j-p3	GGCAGTCTCCTTGGCTAGC	MSTRG.13491.2	3	1167-1184	1175	2	38	3.8
790	osa-MIR169j-p3	GGCAGTCTCCTTGGCTAGC	MSTRG.13491.4	3	1159-1176	1167	2	38	3.8
791	osa-MIR169j-p3	GGCAGTCTCCTTGGCTAGC	LOC_Os05g28290.1	3.5	760-777	768	2	34	34
792	osa-MIR169j-p3	GGCAGTCTCCTTGGCTAGC	LOC_Os10g34660.1	3.5	701-720	711	4	1	0.5
793	osa-MIR169j-p3	GGCAGTCTCCTTGGCTAGC	MSTRG.5115.1	3.5	725-744	735	4	1	0.5
794	osa-MIR169j-p3	GGCAGTCTCCTTGGCTAGC	LOC_Os03g49140.1	3	208-225	216	4	1	0.333333333
795	osa-MIR169j-p3	GGCAGTCTCCTTGGCTAGC	MSTRG.15582.1	3	1526-1543	1534	4	1	0.333333333
796	osa-MIR169j-p3	GGCAGTCTCCTTGGCTAGC	MSTRG.15582.2	3	1442-1459	1450	4	1	0.333333333
797	osa-MIR169j-p3	GGCAGTCTCCTTGGCTAGC	MSTRG.27813.3	2	905-923	914	2	4	1.333333333
798	osa-MIR169j-p3	GGCAGTCTCCTTGGCTAGC	LOC_Os08g23710.1	2	836-854	845	2	4	1.333333333
799	osa-MIR169j-p3	GGCAGTCTCCTTGGCTAGC	MSTRG.27813.2	2	1088-1106	1097	2	4	1.333333333
800	osa-MIR169k-p3	GGCAGTCTCCTTGGCTAGT	MSTRG.3753.1	4	864-882	873	2	4	4
801	osa-MIR169k-p3	GGCAGTCTCCTTGGCTAGT	LOC_Os02g24330.1	4	1400-1417	1408	2	2	1
802	osa-MIR169k-p3	GGCAGTCTCCTTGGCTAGT	LOC_Os02g24330.2	4	3307-3324	3315	2	2	1
803	osa-MIR169k-p3	GGCAGTCTCCTTGGCTAGT	LOC_Os03g08010.1	3	961-978	969	2	39	4.05
804	osa-MIR169k-p3	GGCAGTCTCCTTGGCTAGT	LOC_Os03g08020.1	3	961-978	969	2	40	4
805	osa-MIR169k-p3	GGCAGTCTCCTTGGCTAGT	LOC_Os03g08050.1	3	961-978	969	2	40	4
806	osa-MIR169k-p3	GGCAGTCTCCTTGGCTAGT	MSTRG.13491.8	3	1081-1098	1089	2	37	3.7
807	osa-MIR169k-p3	GGCAGTCTCCTTGGCTAGT	MSTRG.13491.13	3	1068-1085	1076	2	34	3.55
808	osa-MIR169k-p3	GGCAGTCTCCTTGGCTAGT	MSTRG.13491.7	3	1081-1098	1089	2	40	4
809	osa-MIR169k-p3	GGCAGTCTCCTTGGCTAGT	MSTRG.13491.10	3	1160-1177	1168	2	43	4.3
810	osa-MIR169k-p3	GGCAGTCTCCTTGGCTAGT	MSTRG.13491.11	3	1160-1177	1168	2	37	3.85
811	osa-MIR169k-p3	GGCAGTCTCCTTGGCTAGT	MSTRG.13491.3	3	1167-1184	1175	2	39	3.9
812	osa-MIR169k-p3	GGCAGTCTCCTTGGCTAGT	MSTRG.13491.6	3	1168-1185	1176	2	41	4.1
813	osa-MIR169k-p3	GGCAGTCTCCTTGGCTAGT	MSTRG.13491.2	3	1167-1184	1175	2	38	3.8
814	osa-MIR169k-p3	GGCAGTCTCCTTGGCTAGT	MSTRG.13491.1	3	1167-1184	1175	2	38	3.95
815	osa-MIR169k-p3	GGCAGTCTCCTTGGCTAGT	MSTRG.13491.4	3	1159-1176	1167	2	38	3.8
816	osa-MIR169k-p3	GGCAGTCTCCTTGGCTAGT	LOC_Os05g28290.1	4	760-777	768	2	34	34
817	osa-MIR169k-p3	GGCAGTCTCCTTGGCTAGT	LOC_Os07g29630.1	4	639-657	648	2	2	1
818	osa-MIR169k-p3	GGCAGTCTCCTTGGCTAGT	MSTRG.25637.1	4	696-714	705	2	2	1
819	osa-MIR169k-p3	GGCAGTCTCCTTGGCTAGT	LOC_Os10g34660.1	4	701-720	711	4	1	0.5
820	osa-MIR169k-p3	GGCAGTCTCCTTGGCTAGT	MSTRG.5115.1	4	725-744	735	4	1	0.5
821	osa-MIR169k-p3	GGCAGTCTCCTTGGCTAGT	MSTRG.684.3	4	1453-1470	1461	4	1	0.2
822	osa-MIR169k-p3	GGCAGTCTCCTTGGCTAGT	MSTRG.684.1	4	1718-1735	1726	4	1	0.2
823	osa-MIR169k-p3	GGCAGTCTCCTTGGCTAGT	LOC_Os01g11070.1	4	1870-1887	1878	4	1	0.2

824	osa-MIR169k-p3	GGCAGTCTCCTTGGCTAGT	MSTRG.684.4	4	2293-2310	2301	4	1	0.2
825	osa-MIR169k-p3	GGCAGTCTCCTTGGCTAGT	MSTRG.684.5	4	2399-2416	2407	4	1	0.2
826	osa-MIR169k-p3	GGCAGTCTCCTTGGCTAGT	LOC_Os03g49140.1	3.5	208-225	216	4	1	0.333333333
827	osa-MIR169k-p3	GGCAGTCTCCTTGGCTAGT	MSTRG.15582.1	3.5	1526-1543	1534	4	1	0.333333333
828	osa-MIR169k-p3	GGCAGTCTCCTTGGCTAGT	MSTRG.15582.2	3.5	1442-1459	1450	4	1	0.333333333
829	osa-MIR169k-p3	GGCAGTCTCCTTGGCTAGT	MSTRG.27813.3	2.5	905-923	914	2	4	1.333333333
830	osa-MIR169k-p3	GGCAGTCTCCTTGGCTAGT	LOC_Os08g23710.1	2.5	836-854	845	2	4	1.333333333
831	osa-MIR169k-p3	GGCAGTCTCCTTGGCTAGT	MSTRG.27813.2	2.5	1088-1106	1097	2	4	1.333333333
832	osa-MIR169l-p3	GGCAGTCTCCTTGGCTAGC	LOC_Os01g55020.1	4	141-158	149	0	13	1.444444444
833	osa-MIR169l-p3	GGCAGTCTCCTTGGCTAGC	LOC_Os01g55020.3	4	141-158	149	2	13	1.444444444
834	osa-MIR169l-p3	GGCAGTCTCCTTGGCTAGC	LOC_Os01g55020.5	4	141-158	149	2	13	1.444444444
835	osa-MIR169l-p3	GGCAGTCTCCTTGGCTAGC	LOC_Os01g55020.2	4	141-158	149	2	13	1.444444444
836	osa-MIR169l-p3	GGCAGTCTCCTTGGCTAGC	MSTRG.2766.1	4	193-210	201	2	13	1.444444444
837	osa-MIR169l-p3	GGCAGTCTCCTTGGCTAGC	LOC_Os01g55020.4	4	169-186	177	2	13	1.444444444
838	osa-MIR169l-p3	GGCAGTCTCCTTGGCTAGC	LOC_Os01g55020.6	4	141-158	149	2	13	1.444444444
839	osa-MIR169l-p3	GGCAGTCTCCTTGGCTAGC	MSTRG.2766.3	4	180-197	188	2	13	1.444444444
840	osa-MIR169l-p3	GGCAGTCTCCTTGGCTAGC	MSTRG.2766.2	4	186-203	194	2	13	1.444444444
841	osa-MIR169l-p3	GGCAGTCTCCTTGGCTAGC	MSTRG.3753.1	4	864-882	873	2	4	4
842	osa-MIR169l-p3	GGCAGTCTCCTTGGCTAGC	LOC_Os02g24330.1	3.5	1400-1417	1408	2	2	1
843	osa-MIR169l-p3	GGCAGTCTCCTTGGCTAGC	LOC_Os02g24330.2	3.5	3307-3324	3315	2	2	1
844	osa-MIR169l-p3	GGCAGTCTCCTTGGCTAGC	LOC_Os03g04400.1	4	825-842	833	2	22	22
845	osa-MIR169l-p3	GGCAGTCTCCTTGGCTAGC	LOC_Os03g08020.1	3	961-978	969	2	40	4
846	osa-MIR169l-p3	GGCAGTCTCCTTGGCTAGC	LOC_Os03g08050.1	3	961-978	969	2	40	4
847	osa-MIR169l-p3	GGCAGTCTCCTTGGCTAGC	LOC_Os03g08010.1	3	961-978	969	2	39	4.05
848	osa-MIR169l-p3	GGCAGTCTCCTTGGCTAGC	MSTRG.13491.8	3	1081-1098	1089	2	37	3.7
849	osa-MIR169l-p3	GGCAGTCTCCTTGGCTAGC	MSTRG.13491.13	3	1068-1085	1076	2	34	3.55
850	osa-MIR169l-p3	GGCAGTCTCCTTGGCTAGC	MSTRG.13491.7	3	1081-1098	1089	2	40	4
851	osa-MIR169l-p3	GGCAGTCTCCTTGGCTAGC	MSTRG.13491.10	3	1160-1177	1168	2	43	4.3
852	osa-MIR169l-p3	GGCAGTCTCCTTGGCTAGC	MSTRG.13491.11	3	1160-1177	1168	2	37	3.85
853	osa-MIR169l-p3	GGCAGTCTCCTTGGCTAGC	MSTRG.13491.3	3	1167-1184	1175	2	39	3.9
854	osa-MIR169l-p3	GGCAGTCTCCTTGGCTAGC	MSTRG.13491.6	3	1168-1185	1176	2	41	4.1
855	osa-MIR169l-p3	GGCAGTCTCCTTGGCTAGC	MSTRG.13491.1	3	1167-1184	1175	2	38	3.95
856	osa-MIR169l-p3	GGCAGTCTCCTTGGCTAGC	MSTRG.13491.2	3	1167-1184	1175	2	38	3.8
857	osa-MIR169l-p3	GGCAGTCTCCTTGGCTAGC	MSTRG.13491.4	3	1159-1176	1167	2	38	3.8
858	osa-MIR169l-p3	GGCAGTCTCCTTGGCTAGC	LOC_Os05g28290.1	3.5	760-777	768	2	34	34
859	osa-MIR169l-p3	GGCAGTCTCCTTGGCTAGC	LOC_Os10g34660.1	3.5	701-720	711	4	1	0.5
860	osa-MIR169l-p3	GGCAGTCTCCTTGGCTAGC	MSTRG.5115.1	3.5	725-744	735	4	1	0.5
861	osa-MIR169l-p3	GGCAGTCTCCTTGGCTAGC	LOC_Os03g49140.1	3	208-225	216	4	1	0.333333333
862	osa-MIR169l-p3	GGCAGTCTCCTTGGCTAGC	MSTRG.15582.1	3	1526-1543	1534	4	1	0.333333333
863	osa-MIR169l-p3	GGCAGTCTCCTTGGCTAGC	MSTRG.15582.2	3	1442-1459	1450	4	1	0.333333333
864	osa-MIR169l-p3	GGCAGTCTCCTTGGCTAGC	MSTRG.27813.3	2	905-923	914	2	4	1.333333333
865	osa-MIR169l-p3	GGCAGTCTCCTTGGCTAGC	LOC_Os08g23710.1	2	836-854	845	2	4	1.333333333
866	osa-MIR169l-p3	GGCAGTCTCCTTGGCTAGC	MSTRG.27813.2	2	1088-1106	1097	2	4	1.333333333
867	osa-MIR169m-p3	GGCAGTCTCCTTGGCTAGC	LOC_Os01g55020.1	4	141-158	149	0	13	1.444444444
868	osa-MIR169m-p3	GGCAGTCTCCTTGGCTAGC	LOC_Os01g55020.3	4	141-158	149	2	13	1.444444444
869	osa-MIR169m-p3	GGCAGTCTCCTTGGCTAGC	LOC_Os01g55020.5	4	141-158	149	2	13	1.444444444
870	osa-MIR169m-p3	GGCAGTCTCCTTGGCTAGC	LOC_Os01g55020.2	4	141-158	149	2	13	1.444444444
871	osa-MIR169m-p3	GGCAGTCTCCTTGGCTAGC	MSTRG.2766.1	4	193-210	201	2	13	1.444444444
872	osa-MIR169m-p3	GGCAGTCTCCTTGGCTAGC	LOC_Os01g55020.4	4	169-186	177	2	13	1.444444444
873	osa-MIR169m-p3	GGCAGTCTCCTTGGCTAGC	LOC_Os01g55020.6	4	141-158	149	2	13	1.444444444
874	osa-MIR169m-p3	GGCAGTCTCCTTGGCTAGC	MSTRG.2766.3	4	180-197	188	2	13	1.444444444
875	osa-MIR169m-p3	GGCAGTCTCCTTGGCTAGC	MSTRG.2766.2	4	186-203	194	2	13	1.444444444
876	osa-MIR169m-p3	GGCAGTCTCCTTGGCTAGC	MSTRG.3753.1	4	864-882	873	2	4	4
877	osa-MIR169m-p3	GGCAGTCTCCTTGGCTAGC	LOC_Os02g24330.1	3.5	1400-1417	1408	2	2	1
878	osa-MIR169m-p3	GGCAGTCTCCTTGGCTAGC	LOC_Os02g24330.2	3.5	3307-3324	3315	2	2	1
879	osa-MIR169m-p3	GGCAGTCTCCTTGGCTAGC	LOC_Os03g04400.1	4	825-842	833	2	22	22
880	osa-MIR169m-p3	GGCAGTCTCCTTGGCTAGC	LOC_Os03g08020.1	3	961-978	969	2	40	4
881	osa-MIR169m-p3	GGCAGTCTCCTTGGCTAGC	LOC_Os03g08010.1	3	961-978	969	2	39	4.05
882	osa-MIR169m-p3	GGCAGTCTCCTTGGCTAGC	LOC_Os03g08050.1	3	961-978	969	2	40	4

883	osa-MIR169m-p3	GGCAGTCTCCTTGGCTAGC	MSTRG.13491.8	3	1081-1098	1089	2	37	3.7
884	osa-MIR169m-p3	GGCAGTCTCCTTGGCTAGC	MSTRG.13491.13	3	1068-1085	1076	2	34	3.55
885	osa-MIR169m-p3	GGCAGTCTCCTTGGCTAGC	MSTRG.13491.7	3	1081-1098	1089	2	40	4
886	osa-MIR169m-p3	GGCAGTCTCCTTGGCTAGC	MSTRG.13491.10	3	1160-1177	1168	2	43	4.3
887	osa-MIR169m-p3	GGCAGTCTCCTTGGCTAGC	MSTRG.13491.11	3	1160-1177	1168	2	37	3.85
888	osa-MIR169m-p3	GGCAGTCTCCTTGGCTAGC	MSTRG.13491.3	3	1167-1184	1175	2	39	3.9
889	osa-MIR169m-p3	GGCAGTCTCCTTGGCTAGC	MSTRG.13491.6	3	1168-1185	1176	2	41	4.1
890	osa-MIR169m-p3	GGCAGTCTCCTTGGCTAGC	MSTRG.13491.2	3	1167-1184	1175	2	38	3.8
891	osa-MIR169m-p3	GGCAGTCTCCTTGGCTAGC	MSTRG.13491.1	3	1167-1184	1175	2	38	3.95
892	osa-MIR169m-p3	GGCAGTCTCCTTGGCTAGC	MSTRG.13491.4	3	1159-1176	1167	2	38	3.8
893	osa-MIR169m-p3	GGCAGTCTCCTTGGCTAGC	LOC_Os05g28290.1	3.5	760-777	768	2	34	34
894	osa-MIR169m-p3	GGCAGTCTCCTTGGCTAGC	LOC_Os10g34660.1	3.5	701-720	711	4	1	0.5
895	osa-MIR169m-p3	GGCAGTCTCCTTGGCTAGC	MSTRG.5115.1	3.5	725-744	735	4	1	0.5
896	osa-MIR169m-p3	GGCAGTCTCCTTGGCTAGC	LOC_Os03g49140.1	3	208-225	216	4	1	0.333333333
897	osa-MIR169m-p3	GGCAGTCTCCTTGGCTAGC	MSTRG.15582.1	3	1526-1543	1534	4	1	0.333333333
898	osa-MIR169m-p3	GGCAGTCTCCTTGGCTAGC	MSTRG.15582.2	3	1442-1459	1450	4	1	0.333333333
899	osa-MIR169m-p3	GGCAGTCTCCTTGGCTAGC	MSTRG.27813.3	2	905-923	914	2	4	1.333333333
900	osa-MIR169m-p3	GGCAGTCTCCTTGGCTAGC	LOC_Os08g23710.1	2	836-854	845	2	4	1.333333333
901	osa-MIR169m-p3	GGCAGTCTCCTTGGCTAGC	MSTRG.27813.2	2	1088-1106	1097	2	4	1.333333333
902	osa-miR169n	TAGCCAAGAATGACTTGCCTA	LOC_Os05g25180.1	4	1270-1291	1282	2	2	2
903	osa-miR169n	TAGCCAAGAATGACTTGCCTA	LOC_Os12g42400.2	1.5	1202-1222	1213	0	59	19.66666667
904	osa-miR169n	TAGCCAAGAATGACTTGCCTA	LOC_Os12g42400.3	1.5	1247-1267	1258	0	59	19.66666667
905	osa-miR169n	TAGCCAAGAATGACTTGCCTA	LOC_Os12g42400.1	1.5	1256-1276	1267	0	59	19.66666667
906	osa-miR169n	TAGCCAAGAATGACTTGCCTA	LOC_Os03g44540.1	2	1718-1738	1729	0	11	11
907	osa-miR169n	TAGCCAAGAATGACTTGCCTA	LOC_Os03g48970.4	3	1228-1248	1239	2	113	18.83333333
908	osa-miR169n	TAGCCAAGAATGACTTGCCTA	LOC_Os03g48970.1	3	1236-1256	1247	2	113	18.83333333
909	osa-miR169n	TAGCCAAGAATGACTTGCCTA	LOC_Os03g48970.2	3	1765-1785	1776	2	113	18.83333333
910	osa-miR169n	TAGCCAAGAATGACTTGCCTA	MSTRG.15580.1	3	1873-1893	1884	2	113	18.83333333
911	osa-miR169n	TAGCCAAGAATGACTTGCCTA	LOC_Os03g48970.3	3	1935-1955	1946	2	113	18.83333333
912	osa-miR169n	TAGCCAAGAATGACTTGCCTA	MSTRG.15580.2	3	2753-2773	2764	2	113	18.83333333
913	osa-MIR169n-p3	GGCCGGTCTTCTTGGCTAGC	LOC_Os03g04550.1	3.5	1092-1111	1102	2	2	1
914	osa-MIR169n-p3	GGCCGGTCTTCTTGGCTAGC	MSTRG.13255.1	3.5	2834-2853	2844	2	2	1
915	osa-MIR169n-p3	GGCCGGTCTTCTTGGCTAGC	LOC_Os02g54890.1	4	1122-1140	1131	4	1	1
916	osa-MIR169n-p3	GGCCGGTCTTCTTGGCTAGC	LOC_Os05g38640.1	2.5	475-494	485	2	4	4
917	osa-MIR169n-p3	GGCCGGTCTTCTTGGCTAGC	MSTRG.15888.6	4	187-206	197	4	1	0.166666667
918	osa-MIR169n-p3	GGCCGGTCTTCTTGGCTAGC	LOC_Os03g53860.4	4	247-266	257	4	1	0.166666667
919	osa-MIR169n-p3	GGCCGGTCTTCTTGGCTAGC	LOC_Os03g53860.1	4	371-390	381	4	1	0.166666667
920	osa-MIR169n-p3	GGCCGGTCTTCTTGGCTAGC	LOC_Os03g53860.2	4	587-606	597	4	1	0.166666667
921	osa-MIR169n-p3	GGCCGGTCTTCTTGGCTAGC	LOC_Os03g53860.3	4	806-825	816	4	1	0.166666667
922	osa-MIR169n-p3	GGCCGGTCTTCTTGGCTAGC	LOC_Os03g53860.5	4	806-825	816	4	1	0.166666667
923	osa-MIR169o-p3	GGCCGGTCTTCTTGGCTAGC	LOC_Os03g04550.1	3.5	1092-1111	1102	2	2	1
924	osa-MIR169o-p3	GGCCGGTCTTCTTGGCTAGC	MSTRG.13255.1	3.5	2834-2853	2844	2	2	1
925	osa-MIR169o-p3	GGCCGGTCTTCTTGGCTAGC	LOC_Os02g54890.1	4	1122-1140	1131	4	1	1
926	osa-MIR169o-p3	GGCCGGTCTTCTTGGCTAGC	LOC_Os05g38640.1	2.5	475-494	485	2	4	4
927	osa-MIR169o-p3	GGCCGGTCTTCTTGGCTAGC	MSTRG.15888.6	4	187-206	197	4	1	0.166666667
928	osa-MIR169o-p3	GGCCGGTCTTCTTGGCTAGC	LOC_Os03g53860.4	4	247-266	257	4	1	0.166666667
929	osa-MIR169o-p3	GGCCGGTCTTCTTGGCTAGC	LOC_Os03g53860.1	4	371-390	381	4	1	0.166666667
930	osa-MIR169o-p3	GGCCGGTCTTCTTGGCTAGC	LOC_Os03g53860.2	4	587-606	597	4	1	0.166666667
931	osa-MIR169o-p3	GGCCGGTCTTCTTGGCTAGC	LOC_Os03g53860.3	4	806-825	816	4	1	0.166666667
932	osa-MIR169o-p3	GGCCGGTCTTCTTGGCTAGC	LOC_Os03g53860.5	4	806-825	816	4	1	0.166666667
933	osa-miR169r-5p_R+1	TAGCCAAGGATGATTTGCCTGT	LOC_Os03g07880.3	3	754-776	767	2	15	5
934	osa-miR169r-5p_R+1	TAGCCAAGGATGATTTGCCTGT	LOC_Os03g07880.1	3	842-864	855	2	15	5
935	osa-miR169r-5p_R+1	TAGCCAAGGATGATTTGCCTGT	LOC_Os03g07880.2	3	991-1013	1004	2	15	5
936	osa-miR169r-5p_R+1	TAGCCAAGGATGATTTGCCTGT	LOC_Os12g42400.2	3.5	1201-1222	1213	0	59	19.66666667
937	osa-miR169r-5p_R+1	TAGCCAAGGATGATTTGCCTGT	LOC_Os12g42400.3	3.5	1246-1267	1258	0	59	19.66666667
938	osa-miR169r-5p_R+1	TAGCCAAGGATGATTTGCCTGT	LOC_Os12g42400.1	3.5	1255-1276	1267	0	59	19.66666667
939	osa-miR169r-5p_R+1	TAGCCAAGGATGATTTGCCTGT	LOC_Os03g44540.1	3	1717-1738	1729	0	11	11
940	osa-miR169r-5p_R+1	TAGCCAAGGATGATTTGCCTGT	LOC_Os03g48970.4	4	1227-1248	1239	2	113	18.83333333
941	osa-miR169r-5p_R+1	TAGCCAAGGATGATTTGCCTGT	LOC_Os03g48970.1	4	1235-1256	1247	2	113	18.83333333

942	osa-miR169r-5p_R+1	TAGCCAAGGATGATTTGCCTGT	LOC_Os03g48970.2	4	1764-1785	1776	2	113	18.83333333
943	osa-miR169r-5p_R+1	TAGCCAAGGATGATTTGCCTGT	MSTRG.15580.1	4	1872-1893	1884	2	113	18.83333333
944	osa-miR169r-5p_R+1	TAGCCAAGGATGATTTGCCTGT	LOC_Os03g48970.3	4	1934-1955	1946	2	113	18.83333333
945	osa-miR169r-5p_R+1	TAGCCAAGGATGATTTGCCTGT	MSTRG.15580.2	4	2752-2773	2764	2	113	18.83333333
946	osa-miR171a_1ss12CT	TGATTGAGCCGTGCCAATATC	LOC_Os02g44360.1	1	1351-1371	1362	4	1	1
947	osa-miR171a_1ss12CT	TGATTGAGCCGTGCCAATATC	MSTRG.11971.1	1	1409-1429	1420	2	5	2.5
948	osa-miR171a_1ss12CT	TGATTGAGCCGTGCCAATATC	LOC_Os02g44370.1	1	1526-1546	1537	2	5	2.5
949	osa-miR171a_1ss12CT	TGATTGAGCCGTGCCAATATC	LOC_Os04g46860.1	1	1326-1346	1337	2	28	28
950	osa-miR171a_1ss12CT	TGATTGAGCCGTGCCAATATC	LOC_Os06g01620.1	1	456-476	467	4	1	0.5
951	osa-miR171a_1ss12CT	TGATTGAGCCGTGCCAATATC	MSTRG.21932.1	1	713-733	724	4	1	0.5
952	osa-miR171a_1ss12CT	TGATTGAGCCGTGCCAATATC	LOC_Os10g40390.1	2	168-188	179	4	1	0.5
953	osa-miR171a_1ss12CT	TGATTGAGCCGTGCCAATATC	MSTRG.5478.2	2	1166-1186	1177	4	1	0.5
954	osa-miR171b	TGATTGAGCCGTGCCAATATC	LOC_Os02g44360.1	1	1351-1371	1362	4	1	1
955	osa-miR171b	TGATTGAGCCGTGCCAATATC	MSTRG.11971.1	1	1409-1429	1420	2	5	2.5
956	osa-miR171b	TGATTGAGCCGTGCCAATATC	LOC_Os02g44370.1	1	1526-1546	1537	2	5	2.5
957	osa-miR171b	TGATTGAGCCGTGCCAATATC	LOC_Os04g46860.1	1	1326-1346	1337	2	28	28
958	osa-miR171b	TGATTGAGCCGTGCCAATATC	LOC_Os06g01620.1	1	456-476	467	4	1	0.5
959	osa-miR171b	TGATTGAGCCGTGCCAATATC	MSTRG.21932.1	1	713-733	724	4	1	0.5
960	osa-miR171b	TGATTGAGCCGTGCCAATATC	LOC_Os10g40390.1	2	168-188	179	4	1	0.5
961	osa-miR171b	TGATTGAGCCGTGCCAATATC	MSTRG.5478.2	2	1166-1186	1177	4	1	0.5
962	osa-MIR171i-p3	TTGAGCCGCGTCAATATCTCT	LOC_Os02g44360.1	1	1348-1368	1359	0	6	6
963	osa-MIR171i-p3	TTGAGCCGCGTCAATATCTCT	MSTRG.11971.1	2	1406-1426	1417	0	8	4
964	osa-MIR171i-p3	TTGAGCCGCGTCAATATCTCT	LOC_Os02g44370.1	2	1523-1543	1534	0	8	4
965	osa-MIR171i-p3	TTGAGCCGCGTCAATATCTCT	LOC_Os04g46860.1	1	1323-1343	1334	0	62	62
966	osa-MIR171i-p3	TTGAGCCGCGTCAATATCTCT	LOC_Os06g01620.1	1.5	453-473	464	1	2	1
967	osa-MIR171i-p3	TTGAGCCGCGTCAATATCTCT	MSTRG.21932.1	1.5	710-730	721	2	2	1
968	osa-MIR171i-p3	TTGAGCCGCGTCAATATCTCT	LOC_Os10g40390.1	2	165-185	176	0	35	17.5
969	osa-MIR171i-p3	TTGAGCCGCGTCAATATCTCT	MSTRG.5478.2	2	1163-1183	1174	0	35	17.5
970	osa-miR172a	AGAATCTTGATGATGCTGCAT	MSTRG.4023.1	3	518-538	529	2	5	5
971	osa-miR172a	AGAATCTTGATGATGCTGCAT	LOC_Os04g55560.4	4	807-827	818	0	29	5.8
972	osa-miR172a	AGAATCTTGATGATGCTGCAT	LOC_Os04g55560.2	4	1623-1643	1634	0	29	5.8
973	osa-miR172a	AGAATCTTGATGATGCTGCAT	LOC_Os04g55560.3	4	1634-1654	1645	0	29	5.8
974	osa-miR172a	AGAATCTTGATGATGCTGCAT	MSTRG.19117.4	4	1909-1929	1920	0	29	5.8
975	osa-miR172a	AGAATCTTGATGATGCTGCAT	MSTRG.19117.1	4	2085-2105	2096	0	29	5.8
976	osa-miR172a	AGAATCTTGATGATGCTGCAT	LOC_Os05g03040.2	2	1552-1572	1563	0	15	5
977	osa-miR172a	AGAATCTTGATGATGCTGCAT	LOC_Os05g03040.1	2	1976-1996	1987	0	15	5
978	osa-miR172a	AGAATCTTGATGATGCTGCAT	LOC_Os05g03040.3	2	2089-2109	2100	0	15	5
979	osa-miR172a	AGAATCTTGATGATGCTGCAT	LOC_Os09g21770.1	3	686-705	697	2	6	2
980	osa-miR172a	AGAATCTTGATGATGCTGCAT	MSTRG.29630.1	3	798-817	809	2	6	2
981	osa-miR172a	AGAATCTTGATGATGCTGCAT	MSTRG.29630.2	3	843-862	854	2	6	2
982	osa-miR172a	AGAATCTTGATGATGCTGCAT	LOC_Os03g60430.1	2	1755-1775	1766	1	3	0.75
983	osa-miR172a	AGAATCTTGATGATGCTGCAT	LOC_Os03g60430.2	2	1767-1787	1778	1	3	0.75
984	osa-miR172a	AGAATCTTGATGATGCTGCAT	MSTRG.16339.2	2	1933-1953	1944	1	3	0.75
985	osa-miR172a	AGAATCTTGATGATGCTGCAT	MSTRG.16339.1	2	2040-2060	2051	1	3	0.75
986	osa-miR172a	AGAATCTTGATGATGCTGCAT	LOC_Os07g13170.2	2	1463-1483	1474	2	3	0.75
987	osa-miR172a	AGAATCTTGATGATGCTGCAT	LOC_Os07g13170.1	2	1404-1424	1415	2	3	0.75
988	osa-miR172a	AGAATCTTGATGATGCTGCAT	MSTRG.25169.4	2	1572-1592	1583	2	3	0.75
989	osa-miR172a	AGAATCTTGATGATGCTGCAT	MSTRG.25169.1	2	1565-1585	1576	2	3	0.75
990	osa-miR172a	AGAATCTTGATGATGCTGCAT	MSTRG.23975.2	3	1427-1447	1438	4	1	0.5
991	osa-miR172a	AGAATCTTGATGATGCTGCAT	LOC_Os06g43220.1	3	1480-1500	1491	4	1	0.5
992	osa-MIR172a-p5	GTGGCATCATCAAGATTCACA	MSTRG.11635.46	4	923-943	934	4	1	0.1
993	osa-MIR172a-p5	GTGGCATCATCAAGATTCACA	LOC_Os02g38690.1	4	195-215	206	4	1	0.1
994	osa-MIR172a-p5	GTGGCATCATCAAGATTCACA	MSTRG.11635.48	4	915-935	926	4	1	0.1
995	osa-MIR172a-p5	GTGGCATCATCAAGATTCACA	MSTRG.11635.26	4	758-778	769	4	1	0.1
996	osa-MIR172a-p5	GTGGCATCATCAAGATTCACA	MSTRG.11635.47	4	923-943	934	4	1	0.1
997	osa-MIR172a-p5	GTGGCATCATCAAGATTCACA	MSTRG.11635.41	4	936-956	947	4	1	0.1
998	osa-MIR172a-p5	GTGGCATCATCAAGATTCACA	MSTRG.11635.31	4	3671-3691	3682	4	1	0.1
999	osa-MIR172a-p5	GTGGCATCATCAAGATTCACA	MSTRG.11635.35	4	3648-3668	3659	4	1	0.1
1000	osa-MIR172a-p5	GTGGCATCATCAAGATTCACA	MSTRG.11635.34	4	3770-3790	3781	4	1	0.1

1001	osa-MIR172a-p5	GTGGCATCATCAAGATTCACA	MSTRG.11635.49	4	195-215	206	4	1	0.1
1002	osa-MIR172a-p5	GTGGCATCATCAAGATTCACA	LOC_Os02g42320.4	4	944-964	955	2	31	7.75
1003	osa-MIR172a-p5	GTGGCATCATCAAGATTCACA	LOC_Os02g42320.2	4	698-718	709	2	31	7.75
1004	osa-MIR172a-p5	GTGGCATCATCAAGATTCACA	LOC_Os02g42320.3	4	797-817	808	2	31	7.75
1005	osa-MIR172a-p5	GTGGCATCATCAAGATTCACA	LOC_Os02g42320.1	4	807-827	818	2	31	7.75
1006	osa-MIR172a-p5	GTGGCATCATCAAGATTCACA	LOC_Os08g03550.1	4	1177-1197	1188	2	2	1
1007	osa-MIR172a-p5	GTGGCATCATCAAGATTCACA	MSTRG.26939.1	4	1273-1293	1284	2	2	1
1008	osa-miR172b	GGAATCTTGATGATGCTGCAT	MSTRG.4023.1	3	518-538	529	2	5	5
1009	osa-miR172b	GGAATCTTGATGATGCTGCAT	LOC_Os04g55560.4	3	807-827	818	0	29	5.8
1010	osa-miR172b	GGAATCTTGATGATGCTGCAT	LOC_Os04g55560.2	3	1623-1643	1634	0	29	5.8
1011	osa-miR172b	GGAATCTTGATGATGCTGCAT	LOC_Os04g55560.3	3	1634-1654	1645	0	29	5.8
1012	osa-miR172b	GGAATCTTGATGATGCTGCAT	MSTRG.19117.4	3	1909-1929	1920	0	29	5.8
1013	osa-miR172b	GGAATCTTGATGATGCTGCAT	MSTRG.19117.1	3	2085-2105	2096	0	29	5.8
1014	osa-miR172b	GGAATCTTGATGATGCTGCAT	LOC_Os05g03040.2	2.5	1552-1572	1563	0	15	5
1015	osa-miR172b	GGAATCTTGATGATGCTGCAT	LOC_Os05g03040.1	2.5	1976-1996	1987	0	15	5
1016	osa-miR172b	GGAATCTTGATGATGCTGCAT	LOC_Os05g03040.3	2.5	2089-2109	2100	0	15	5
1017	osa-miR172b	GGAATCTTGATGATGCTGCAT	LOC_Os09g21770.1	3.5	686-705	697	2	6	2
1018	osa-miR172b	GGAATCTTGATGATGCTGCAT	MSTRG.29630.1	3.5	798-817	809	2	6	2
1019	osa-miR172b	GGAATCTTGATGATGCTGCAT	MSTRG.29630.2	3.5	843-862	854	2	6	2
1020	osa-miR172b	GGAATCTTGATGATGCTGCAT	LOC_Os03g60430.1	2.5	1755-1775	1766	1	3	0.75
1021	osa-miR172b	GGAATCTTGATGATGCTGCAT	LOC_Os03g60430.2	2.5	1767-1787	1778	1	3	0.75
1022	osa-miR172b	GGAATCTTGATGATGCTGCAT	MSTRG.16339.2	2.5	1933-1953	1944	1	3	0.75
1023	osa-miR172b	GGAATCTTGATGATGCTGCAT	MSTRG.16339.1	2.5	2040-2060	2051	1	3	0.75
1024	osa-miR172b	GGAATCTTGATGATGCTGCAT	MSTRG.23975.2	2	1427-1447	1438	4	1	0.5
1025	osa-miR172b	GGAATCTTGATGATGCTGCAT	LOC_Os06g43220.1	2	1480-1500	1491	4	1	0.5
1026	osa-miR172b	GGAATCTTGATGATGCTGCAT	LOC_Os07g13170.2	2.5	1463-1483	1474	2	3	0.75
1027	osa-miR172b	GGAATCTTGATGATGCTGCAT	LOC_Os07g13170.1	2.5	1404-1424	1415	2	3	0.75
1028	osa-miR172b	GGAATCTTGATGATGCTGCAT	MSTRG.25169.4	2.5	1572-1592	1583	2	3	0.75
1029	osa-miR172b	GGAATCTTGATGATGCTGCAT	MSTRG.25169.1	2.5	1565-1585	1576	2	3	0.75
1030	osa-MIR172b-p5	GTGGCATCATCAAGATTCACA	MSTRG.11635.46	4	923-943	934	4	1	0.1
1031	osa-MIR172b-p5	GTGGCATCATCAAGATTCACA	LOC_Os02g38690.1	4	195-215	206	4	1	0.1
1032	osa-MIR172b-p5	GTGGCATCATCAAGATTCACA	MSTRG.11635.48	4	915-935	926	4	1	0.1
1033	osa-MIR172b-p5	GTGGCATCATCAAGATTCACA	MSTRG.11635.26	4	758-778	769	4	1	0.1
1034	osa-MIR172b-p5	GTGGCATCATCAAGATTCACA	MSTRG.11635.47	4	923-943	934	4	1	0.1
1035	osa-MIR172b-p5	GTGGCATCATCAAGATTCACA	MSTRG.11635.41	4	936-956	947	4	1	0.1
1036	osa-MIR172b-p5	GTGGCATCATCAAGATTCACA	MSTRG.11635.31	4	3671-3691	3682	4	1	0.1
1037	osa-MIR172b-p5	GTGGCATCATCAAGATTCACA	MSTRG.11635.35	4	3648-3668	3659	4	1	0.1
1038	osa-MIR172b-p5	GTGGCATCATCAAGATTCACA	MSTRG.11635.34	4	3770-3790	3781	4	1	0.1
1039	osa-MIR172b-p5	GTGGCATCATCAAGATTCACA	MSTRG.11635.49	4	195-215	206	4	1	0.1
1040	osa-MIR172b-p5	GTGGCATCATCAAGATTCACA	LOC_Os02g42320.4	4	944-964	955	2	31	7.75
1041	osa-MIR172b-p5	GTGGCATCATCAAGATTCACA	LOC_Os02g42320.2	4	698-718	709	2	31	7.75
1042	osa-MIR172b-p5	GTGGCATCATCAAGATTCACA	LOC_Os02g42320.3	4	797-817	808	2	31	7.75
1043	osa-MIR172b-p5	GTGGCATCATCAAGATTCACA	LOC_Os02g42320.1	4	807-827	818	2	31	7.75
1044	osa-MIR172b-p5	GTGGCATCATCAAGATTCACA	LOC_Os08g03550.1	4	1177-1197	1188	2	2	1
1045	osa-MIR172b-p5	GTGGCATCATCAAGATTCACA	MSTRG.26939.1	4	1273-1293	1284	2	2	1
1046	osa-miR172c_1ss1TG	GGAATCTTGATGATGCTGCAC	MSTRG.4023.1	4	518-538	529	2	5	5
1047	osa-miR172c_1ss1TG	GGAATCTTGATGATGCTGCAC	LOC_Os04g55560.4	3	807-827	818	0	29	5.8
1048	osa-miR172c_1ss1TG	GGAATCTTGATGATGCTGCAC	LOC_Os04g55560.2	3	1623-1643	1634	0	29	5.8
1049	osa-miR172c_1ss1TG	GGAATCTTGATGATGCTGCAC	LOC_Os04g55560.3	3	1634-1654	1645	0	29	5.8
1050	osa-miR172c_1ss1TG	GGAATCTTGATGATGCTGCAC	MSTRG.19117.4	3	1909-1929	1920	0	29	5.8
1051	osa-miR172c_1ss1TG	GGAATCTTGATGATGCTGCAC	MSTRG.19117.1	3	2085-2105	2096	0	29	5.8
1052	osa-miR172c_1ss1TG	GGAATCTTGATGATGCTGCAC	LOC_Os05g03040.2	2.5	1552-1572	1563	0	15	5
1053	osa-miR172c_1ss1TG	GGAATCTTGATGATGCTGCAC	LOC_Os05g03040.1	2.5	1976-1996	1987	0	15	5
1054	osa-miR172c_1ss1TG	GGAATCTTGATGATGCTGCAC	LOC_Os05g03040.3	2.5	2089-2109	2100	0	15	5
1055	osa-miR172c_1ss1TG	GGAATCTTGATGATGCTGCAC	LOC_Os03g60430.1	2.5	1755-1775	1766	1	3	0.75
1056	osa-miR172c_1ss1TG	GGAATCTTGATGATGCTGCAC	LOC_Os03g60430.2	2.5	1767-1787	1778	1	3	0.75
1057	osa-miR172c_1ss1TG	GGAATCTTGATGATGCTGCAC	MSTRG.16339.2	2.5	1933-1953	1944	1	3	0.75
1058	osa-miR172c_1ss1TG	GGAATCTTGATGATGCTGCAC	MSTRG.16339.1	2.5	2040-2060	2051	1	3	0.75
1059	osa-miR172c_1ss1TG	GGAATCTTGATGATGCTGCAC	MSTRG.23975.2	2	1427-1447	1438	4	1	0.5

1060	osa-miR172c_1ss1TG	GGAATCTTGATGATGCTGCAC	LOC_Os06g43220.1	2	1480-1500	1491	4	1	0.5
1061	osa-miR172c_1ss1TG	GGAATCTTGATGATGCTGCAC	LOC_Os07g13170.2	2.5	1463-1483	1474	2	3	0.75
1062	osa-miR172c_1ss1TG	GGAATCTTGATGATGCTGCAC	LOC_Os07g13170.1	2.5	1404-1424	1415	2	3	0.75
1063	osa-miR172c_1ss1TG	GGAATCTTGATGATGCTGCAC	MSTRG.25169.4	2.5	1572-1592	1583	2	3	0.75
1064	osa-miR172c_1ss1TG	GGAATCTTGATGATGCTGCAC	MSTRG.25169.1	2.5	1565-1585	1576	2	3	0.75
1065	osa-miR172d-5p_R+1	GCAGCACCATCAAGATTCACA	LOC_Os02g37150.2	3.5	504-523	515	2	31	15.5
1066	osa-miR172d-5p_R+1	GCAGCACCATCAAGATTCACA	LOC_Os02g37150.1	3.5	677-696	688	2	31	15.5
1067	osa-miR1846a-3p	TGACCCCGTTCTCCTCGCCGG	LOC_Os05g15520.1	3.5	295-315	306	4	1	1
1068	osa-MIR1846d-p3	CGGTGACCCCGGTCTCCTCGC	LOC_Os05g15520.1	2	298-318	309	3	2	2
1069	osa-MIR1847-p5	TTTGTGCAGTTTGCAGTTGTG	MSTRG.2849.3	3.5	2533-2552	2543	4	1	0.333333333
1070	osa-MIR1847-p5	TTTGTGCAGTTTGCAGTTGTG	LOC_Os01g56200.2	3.5	1279-1298	1289	4	1	0.333333333
1071	osa-MIR1847-p5	TTTGTGCAGTTTGCAGTTGTG	LOC_Os01g56200.1	3.5	2030-2049	2040	4	1	0.333333333
1072	osa-MIR1852-p3	GAATGCAGGTGTAGTTTGTTC	LOC_Os03g18590.1	4	668-688	679	2	3	3
1073	osa-MIR1852-p3	GAATGCAGGTGTAGTTTGTTC	MSTRG.22922.2	3.5	1792-1812	1803	4	1	0.333333333
1074	osa-MIR1852-p3	GAATGCAGGTGTAGTTTGTTC	LOC_Os06g19444.1	3.5	1873-1893	1884	4	1	0.333333333
1075	osa-MIR1852-p3	GAATGCAGGTGTAGTTTGTTC	LOC_Os06g19444.2	3.5	1870-1890	1881	4	1	0.333333333
1076	osa-MIR1861d-p3_1ss16AG	GGTTCCTGTCCCAAGGCCGAG	MSTRG.15828.3	4	398-418	409	4	1	0.333333333
1077	osa-MIR1861d-p3_1ss16AG	GGTTCCTGTCCCAAGGCCGAG	LOC_Os03g52780.1	4	404-424	415	4	1	0.333333333
1078	osa-MIR1861d-p3_1ss16AG	GGTTCCTGTCCCAAGGCCGAG	MSTRG.15828.1	4	512-532	523	4	1	0.333333333
1079	osa-miR1861h	CGGTCTTGAGGCAGGAACTGAG	LOC_Os05g51790.1	3.5	578-599	590	4	1	0.2
1080	osa-miR1861h	CGGTCTTGAGGCAGGAACTGAG	LOC_Os05g51790.2	3.5	533-554	545	4	1	0.2
1081	osa-miR1861h	CGGTCTTGAGGCAGGAACTGAG	MSTRG.21889.5	3.5	565-586	577	4	1	0.2
1082	osa-miR1861h	CGGTCTTGAGGCAGGAACTGAG	MSTRG.21889.2	3.5	711-732	723	4	1	0.2
1083	osa-miR1861h	CGGTCTTGAGGCAGGAACTGAG	MSTRG.21889.1	3.5	1199-1220	1211	4	1	0.2
1084	osa-MIR1862a-p3	TTGGTTTATTTTGGGACGGAG	MSTRG.4283.1	3.5	1446-1467	1458	4	1	1
1085	osa-MIR1862a-p3	TTGGTTTATTTTGGGACGGAG	MSTRG.3842.2	4	905-926	916	2	4	2
1086	osa-MIR1862a-p3	TTGGTTTATTTTGGGACGGAG	MSTRG.3842.1	4	789-810	800	2	4	2
1087	osa-MIR1862a-p3	TTGGTTTATTTTGGGACGGAG	MSTRG.9944.1	4	1094-1114	1105	4	1	1
1088	osa-MIR1862a-p3	TTGGTTTATTTTGGGACGGAG	LOC_Os06g39760.1	3	1603-1624	1615	4	1	0.5
1089	osa-MIR1862a-p3	TTGGTTTATTTTGGGACGGAG	MSTRG.23658.2	3	1934-1955	1946	4	1	0.5
1090	osa-MIR1862b-p3	TTGGTTTATTTTGGGACGGAG	MSTRG.4283.1	3.5	1446-1467	1458	4	1	1
1091	osa-MIR1862b-p3	TTGGTTTATTTTGGGACGGAG	MSTRG.3842.2	4	905-926	916	2	4	2
1092	osa-MIR1862b-p3	TTGGTTTATTTTGGGACGGAG	MSTRG.3842.1	4	789-810	800	2	4	2
1093	osa-MIR1862b-p3	TTGGTTTATTTTGGGACGGAG	MSTRG.9944.1	4	1094-1114	1105	4	1	1
1094	osa-MIR1862b-p3	TTGGTTTATTTTGGGACGGAG	LOC_Os06g39760.1	3	1603-1624	1615	4	1	0.5
1095	osa-MIR1862b-p3	TTGGTTTATTTTGGGACGGAG	MSTRG.23658.2	3	1934-1955	1946	4	1	0.5
1096	osa-MIR1862c-p3	TTGGTTTATTTTGGGACGGAG	MSTRG.4283.1	3.5	1446-1467	1458	4	1	1
1097	osa-MIR1862c-p3	TTGGTTTATTTTGGGACGGAG	MSTRG.3842.2	4	905-926	916	2	4	2
1098	osa-MIR1862c-p3	TTGGTTTATTTTGGGACGGAG	MSTRG.3842.1	4	789-810	800	2	4	2
1099	osa-MIR1862c-p3	TTGGTTTATTTTGGGACGGAG	MSTRG.9944.1	4	1094-1114	1105	4	1	1
1100	osa-MIR1862c-p3	TTGGTTTATTTTGGGACGGAG	LOC_Os06g39760.1	3	1603-1624	1615	4	1	0.5
1101	osa-MIR1862c-p3	TTGGTTTATTTTGGGACGGAG	MSTRG.23658.2	3	1934-1955	1946	4	1	0.5
1102	osa-miR1862d	ACTAGGTTTGTTTATTTTGGGACG	LOC_Os02g30730.1	3.5	2708-2731	2722	4	1	0.2
1103	osa-miR1862d	ACTAGGTTTGTTTATTTTGGGACG	LOC_Os02g30730.2	3.5	2784-2807	2798	4	1	0.2
1104	osa-miR1862d	ACTAGGTTTGTTTATTTTGGGACG	MSTRG.11196.3	3.5	2921-2944	2935	4	1	0.2
1105	osa-miR1862d	ACTAGGTTTGTTTATTTTGGGACG	MSTRG.11196.1	3.5	2923-2946	2937	4	1	0.2
1106	osa-miR1862d	ACTAGGTTTGTTTATTTTGGGACG	MSTRG.11196.2	3.5	2965-2988	2979	4	1	0.2
1107	osa-miR1862d	ACTAGGTTTGTTTATTTTGGGACG	LOC_Os04g31120.1	4	1892-1915	1906	4	1	0.1
1108	osa-miR1862d	ACTAGGTTTGTTTATTTTGGGACG	LOC_Os04g31120.6	4	1951-1974	1965	4	1	0.1
1109	osa-miR1862d	ACTAGGTTTGTTTATTTTGGGACG	MSTRG.17563.4	4	1788-1811	1802	4	1	0.1
1110	osa-miR1862d	ACTAGGTTTGTTTATTTTGGGACG	LOC_Os04g31120.2	4	2026-2049	2040	4	1	0.1
1111	osa-miR1862d	ACTAGGTTTGTTTATTTTGGGACG	MSTRG.17563.2	4	1868-1891	1882	4	1	0.1
1112	osa-miR1862d	ACTAGGTTTGTTTATTTTGGGACG	MSTRG.17563.1	4	1937-1960	1951	4	1	0.1
1113	osa-miR1862d	ACTAGGTTTGTTTATTTTGGGACG	LOC_Os04g31120.4	4	2384-2407	2398	4	1	0.1
1114	osa-miR1862d	ACTAGGTTTGTTTATTTTGGGACG	MSTRG.17563.3	4	2195-2218	2209	4	1	0.1
1115	osa-miR1862d	ACTAGGTTTGTTTATTTTGGGACG	LOC_Os04g31120.3	4	2461-2484	2475	4	1	0.1
1116	osa-miR1862d	ACTAGGTTTGTTTATTTTGGGACG	LOC_Os04g31120.5	4	3920-3943	3934	4	1	0.1
1117	osa-MIR1862e-p3	TATTTTGGGACGGAGGGAGTA	MSTRG.2373.3	3	4345-4365	4356	4	1	0.333333333
1118	osa-MIR1862e-p3	TATTTTGGGACGGAGGGAGTA	MSTRG.2373.2	3	4479-4499	4490	4	1	0.333333333

1119	osa-MIR1862e-p3	TATTTTGGGACGGAGGGAGTA	MSTRG.2373.1	3	4529-4549	4540	4	1	0.333333333
1120	osa-MIR1862e-p3	TATTTTGGGACGGAGGGAGTA	MSTRG.13526.2	3	1790-1810	1801	4	1	0.5
1121	osa-MIR1862e-p3	TATTTTGGGACGGAGGGAGTA	LOC_Os03g08530.1	3	1871-1891	1882	4	1	0.5
1122	osa-MIR1862e-p3	TATTTTGGGACGGAGGGAGTA	MSTRG.13772.3	4	1620-1640	1631	3	2	0.666666667
1123	osa-MIR1862e-p3	TATTTTGGGACGGAGGGAGTA	MSTRG.19222.8	4	1173-1193	1184	4	1	0.142857143
1124	osa-MIR1862e-p3	TATTTTGGGACGGAGGGAGTA	MSTRG.19222.2	4	1226-1246	1237	4	1	0.142857143
1125	osa-MIR1862e-p3	TATTTTGGGACGGAGGGAGTA	MSTRG.19222.5	4	1501-1521	1512	4	1	0.142857143
1126	osa-MIR1862e-p3	TATTTTGGGACGGAGGGAGTA	MSTRG.19222.4	4	1546-1566	1557	4	1	0.142857143
1127	osa-MIR1862e-p3	TATTTTGGGACGGAGGGAGTA	MSTRG.19222.9	4	2335-2355	2346	4	1	0.142857143
1128	osa-MIR1862e-p3	TATTTTGGGACGGAGGGAGTA	MSTRG.19222.3	4	2698-2718	2709	4	1	0.142857143
1129	osa-MIR1862e-p3	TATTTTGGGACGGAGGGAGTA	MSTRG.19222.1	4	1236-1256	1247	4	1	0.142857143
1130	osa-MIR1862e-p3	TATTTTGGGACGGAGGGAGTA	MSTRG.20874.3	2.5	1173-1193	1184	2	8	2.666666667
1131	osa-MIR1862e-p3	TATTTTGGGACGGAGGGAGTA	MSTRG.20874.4	2.5	1176-1196	1187	2	8	2.666666667
1132	osa-MIR1862e-p3	TATTTTGGGACGGAGGGAGTA	LOC_Os05g34700.1	2.5	1270-1290	1281	2	8	2.666666667
1133	osa-MIR1862e-p3	TATTTTGGGACGGAGGGAGTA	LOC_Os07g28260.1	3	1801-1821	1812	2	4	0.4
1134	osa-MIR1862e-p3	TATTTTGGGACGGAGGGAGTA	LOC_Os07g28260.6	3	1937-1957	1948	2	3	0.3
1135	osa-MIR1862e-p3	TATTTTGGGACGGAGGGAGTA	MSTRG.25585.1	3	1937-1957	1948	2	3	0.3
1136	osa-MIR1862e-p3	TATTTTGGGACGGAGGGAGTA	MSTRG.25585.6	3	1786-1806	1797	2	3	0.3
1137	osa-MIR1862e-p3	TATTTTGGGACGGAGGGAGTA	MSTRG.25585.4	3	1801-1821	1812	2	4	0.4
1138	osa-MIR1862e-p3	TATTTTGGGACGGAGGGAGTA	MSTRG.25585.9	3	1808-1828	1819	2	4	0.4
1139	osa-MIR1862e-p3	TATTTTGGGACGGAGGGAGTA	MSTRG.25585.10	3	1808-1828	1819	2	3	0.3
1140	osa-MIR1862e-p3	TATTTTGGGACGGAGGGAGTA	MSTRG.25585.3	3	2275-2295	2286	2	4	0.4
1141	osa-MIR1862e-p3	TATTTTGGGACGGAGGGAGTA	LOC_Os07g28260.5	3	2055-2075	2066	2	3	0.3
1142	osa-MIR1862e-p3	TATTTTGGGACGGAGGGAGTA	MSTRG.25585.12	3	1801-1821	1812	2	3	0.3
1143	osa-MIR1862e-p3	TATTTTGGGACGGAGGGAGTA	MSTRG.25585.7	3	1751-1771	1762	2	3	0.3
1144	osa-MIR1862e-p3	TATTTTGGGACGGAGGGAGTA	LOC_Os07g28260.4	3	2152-2172	2163	2	3	0.3
1145	osa-MIR1862e-p3	TATTTTGGGACGGAGGGAGTA	MSTRG.28819.1	2	778-798	789	3	2	2
1146	osa-MIR1862e-p3	TATTTTGGGACGGAGGGAGTA	LOC_Os09g20000.1	2	761-781	772	2	3	1
1147	osa-MIR1862e-p3	TATTTTGGGACGGAGGGAGTA	MSTRG.29554.1	2	1227-1247	1238	2	3	1
1148	osa-MIR1862e-p3	TATTTTGGGACGGAGGGAGTA	MSTRG.29554.2	2	1709-1729	1720	2	3	1
1149	osa-MIR1862e-p3	TATTTTGGGACGGAGGGAGTA	LOC_Os09g33690.3	2.5	1532-1552	1543	4	1	0.25
1150	osa-MIR1862e-p3	TATTTTGGGACGGAGGGAGTA	LOC_Os09g33690.2	2.5	1653-1673	1664	4	1	0.25
1151	osa-MIR1862e-p3	TATTTTGGGACGGAGGGAGTA	LOC_Os09g33690.4	2.5	1663-1683	1674	4	1	0.25
1152	osa-MIR1862e-p3	TATTTTGGGACGGAGGGAGTA	LOC_Os09g33690.1	2.5	1699-1719	1710	4	1	0.25
1153	osa-MIR1862e-p3	TATTTTGGGACGGAGGGAGTA	MSTRG.6714.2	3	839-859	850	4	1	0.5
1154	osa-MIR1862e-p3	TATTTTGGGACGGAGGGAGTA	MSTRG.6714.1	3	1823-1843	1834	4	1	0.5
1155	osa-MIR1862e-p3	TATTTTGGGACGGAGGGAGTA	LOC_Os06g50146.1	2.5	1196-1216	1207	4	1	0.5
1156	osa-MIR1862e-p3	TATTTTGGGACGGAGGGAGTA	MSTRG.24397.1	2.5	1207-1227	1218	4	1	0.5
1157	osa-MIR1862g-p3_1ss13TC	AGATTCGTTGTACTAGGATGTGTC	MSTRG.26498.1	2	1113-1136	1127	2	10	1.25
1158	osa-MIR1862g-p3_1ss13TC	AGATTCGTTGTACTAGGATGTGTC	LOC_Os07g45350.2	2	1263-1286	1277	2	10	1.25
1159	osa-MIR1862g-p3_1ss13TC	AGATTCGTTGTACTAGGATGTGTC	LOC_Os07g45350.4	2	1331-1354	1345	2	10	1.25
1160	osa-MIR1862g-p3_1ss13TC	AGATTCGTTGTACTAGGATGTGTC	LOC_Os07g45350.5	2	1354-1377	1368	2	10	1.25
1161	osa-MIR1862g-p3_1ss13TC	AGATTCGTTGTACTAGGATGTGTC	LOC_Os07g45350.1	2	1358-1381	1372	2	10	1.25
1162	osa-MIR1862g-p3_1ss13TC	AGATTCGTTGTACTAGGATGTGTC	MSTRG.26498.8	2	1445-1468	1459	2	10	1.25
1163	osa-MIR1862g-p3_1ss13TC	AGATTCGTTGTACTAGGATGTGTC	MSTRG.26498.2	2	1397-1420	1411	2	10	1.25
1164	osa-MIR1862g-p3_1ss13TC	AGATTCGTTGTACTAGGATGTGTC	LOC_Os07g45350.3	2	1761-1784	1775	2	10	1.25
1165	osa-MIR1862g-p3_1ss13TC	AGATTCGTTGTACTAGGATGTGTC	MSTRG.24976.5	3	1364-1387	1378	2	3	0.428571429
1166	osa-MIR1862g-p3_1ss13TC	AGATTCGTTGTACTAGGATGTGTC	MSTRG.24976.6	3	1403-1426	1417	2	3	0.428571429
1167	osa-MIR1862g-p3_1ss13TC	AGATTCGTTGTACTAGGATGTGTC	MSTRG.24976.1	3	1432-1455	1446	2	3	0.428571429
1168	osa-MIR1862g-p3_1ss13TC	AGATTCGTTGTACTAGGATGTGTC	MSTRG.24976.7	3	2849-2872	2863	2	3	0.428571429
1169	osa-MIR1862g-p3_1ss13TC	AGATTCGTTGTACTAGGATGTGTC	MSTRG.24976.2	3	2860-2883	2874	2	3	0.428571429
1170	osa-MIR1862g-p3_1ss13TC	AGATTCGTTGTACTAGGATGTGTC	LOC_Os07g09460.2	3	541-564	555	2	3	0.428571429
1171	osa-MIR1862g-p3_1ss13TC	AGATTCGTTGTACTAGGATGTGTC	LOC_Os07g09460.1	3	487-510	501	2	3	0.428571429
1172	osa-MIR1862g-p3_1ss13TC	AGATTCGTTGTACTAGGATGTGTC	LOC_Os12g06650.1	3.5	2122-2145	2136	2	2	2
1173	osa-MIR1863a-p5	GTCTAATATGGTATCCGAGCT	MSTRG.1629.8	4	590-610	601	4	1	0.1
1174	osa-MIR1863a-p5	GTCTAATATGGTATCCGAGCT	LOC_Os01g34620.3	4	603-623	614	4	1	0.1
1175	osa-MIR1863a-p5	GTCTAATATGGTATCCGAGCT	LOC_Os01g34620.4	4	603-623	614	4	1	0.1
1176	osa-MIR1863a-p5	GTCTAATATGGTATCCGAGCT	LOC_Os01g34620.9	4	598-618	609	4	1	0.1
1177	osa-MIR1863a-p5	GTCTAATATGGTATCCGAGCT	LOC_Os01g34620.5	4	603-623	614	4	1	0.1

1178	osa-MIR1863a-p5	GTCTAATATGGTATCCGAGCT	LOC_Os01g34620.2	4	575-595	586	4	1	0.1
1179	osa-MIR1863a-p5	GTCTAATATGGTATCCGAGCT	MSTRG.1629.9	4	586-606	597	4	1	0.1
1180	osa-MIR1863a-p5	GTCTAATATGGTATCCGAGCT	LOC_Os01g34620.8	4	1478-1498	1489	4	1	0.1
1181	osa-MIR1863a-p5	GTCTAATATGGTATCCGAGCT	MSTRG.484.2	4	400-420	411	4	1	0.1
1182	osa-MIR1863a-p5	GTCTAATATGGTATCCGAGCT	LOC_Os01g07950.1	4	611-631	622	4	1	0.1
1183	osa-miR1874-3p	TATGGATGGAGGTGTAACCCGATG	LOC_Os02g20950.1	3	446-469	460	2	2	0.4
1184	osa-miR1874-3p	TATGGATGGAGGTGTAACCCGATG	MSTRG.10808.5	3	1012-1035	1026	2	2	0.4
1185	osa-miR1874-3p	TATGGATGGAGGTGTAACCCGATG	MSTRG.10808.1	3	1144-1167	1158	2	2	0.4
1186	osa-miR1874-3p	TATGGATGGAGGTGTAACCCGATG	MSTRG.10808.3	3	1091-1114	1105	2	2	0.4
1187	osa-miR1874-3p	TATGGATGGAGGTGTAACCCGATG	MSTRG.10808.2	3	1128-1151	1142	2	2	0.4
1188	osa-miR2055	TTTCCTTGGGAAGGTGGTTTC	LOC_Os04g54330.3	3.5	785-804	795	4	1	0.5
1189	osa-miR2055	TTTCCTTGGGAAGGTGGTTTC	LOC_Os04g54330.1	3.5	788-807	798	4	1	0.5
1190	osa-miR2055	TTTCCTTGGGAAGGTGGTTTC	MSTRG.5671.3	3.5	474-494	485	4	1	0.1
1191	osa-miR2118a	TTCTCGATGCCTCCCATTCCTA	MSTRG.17799.1	3.5	738-759	750	0	36	36
1192	osa-miR2118a	TTCTCGATGCCTCCCATTCCTA	LOC_Os07g48420.1	4	578-598	589	2	3	0.75
1193	osa-miR2118a	TTCTCGATGCCTCCCATTCCTA	LOC_Os07g48420.3	4	578-598	589	2	3	0.75
1194	osa-miR2118a	TTCTCGATGCCTCCCATTCCTA	LOC_Os07g48420.2	4	578-598	589	2	3	0.75
1195	osa-miR2118a	TTCTCGATGCCTCCCATTCCTA	MSTRG.26717.1	4	743-763	754	2	3	0.75
1196	osa-miR2118a	TTCTCGATGCCTCCCATTCCTA	MSTRG.29394.1	4	106-127	118	0	65	65
1197	osa-miR2118a	TTCTCGATGCCTCCCATTCCTA	MSTRG.6412.3	3	904-924	916	4	1	0.5
1198	osa-miR2118a	TTCTCGATGCCTCCCATTCCTA	MSTRG.6412.1	3	1140-1160	1152	4	1	0.5
1199	osa-miR2118a	TTCTCGATGCCTCCCATTCCTA	MSTRG.9266.1	4	286-307	298	0	84	84
1200	osa-miR2118a	TTCTCGATGCCTCCCATTCCTA	MSTRG.9242.1	4	41-62	53	0	118	118
1201	osa-miR2118a	TTCTCGATGCCTCCCATTCCTA	MSTRG.15550.1	2.5	580-601	592	0	33	33
1202	osa-miR2118b	TTCCCGATGCCTCCCATTCCTA	LOC_Os04g30610.1	4	497-518	509	0	13	13
1203	osa-miR2118b	TTCCCGATGCCTCCCATTCCTA	MSTRG.17799.1	2.5	738-759	750	0	36	36
1204	osa-miR2118b	TTCCCGATGCCTCCCATTCCTA	MSTRG.17802.1	3.5	38-59	50	0	18	18
1205	osa-miR2118b	TTCCCGATGCCTCCCATTCCTA	LOC_Os05g34220.1	4	671-692	683	0	18	9
1206	osa-miR2118b	TTCCCGATGCCTCCCATTCCTA	LOC_Os05g34220.2	4	671-692	683	0	18	9
1207	osa-miR2118b	TTCCCGATGCCTCCCATTCCTA	MSTRG.29394.1	3	106-127	118	0	65	65
1208	osa-miR2118b	TTCCCGATGCCTCCCATTCCTA	MSTRG.9266.1	3	286-307	298	0	84	84
1209	osa-miR2118b	TTCCCGATGCCTCCCATTCCTA	MSTRG.8777.1	4	106-127	118	4	1	1
1210	osa-miR2118b	TTCCCGATGCCTCCCATTCCTA	MSTRG.8657.1	4	137-158	149	1	16	16
1211	osa-miR2118b	TTCCCGATGCCTCCCATTCCTA	MSTRG.23756.1	3.5	142-163	154	1	16	16
1212	osa-miR2118b	TTCCCGATGCCTCCCATTCCTA	MSTRG.23774.1	4	10-31	22	0	29	29
1213	osa-miR2118b	TTCCCGATGCCTCCCATTCCTA	MSTRG.28057.1	3.5	66-87	78	0	7	7
1214	osa-miR2118c	TTCCCGATGCCTCCTATTCCTA	MSTRG.17799.1	3	738-759	750	0	36	36
1215	osa-miR2118c	TTCCCGATGCCTCCTATTCCTA	MSTRG.17802.1	4	38-59	50	0	18	18
1216	osa-miR2118c	TTCCCGATGCCTCCTATTCCTA	LOC_Os05g34220.1	4	671-692	683	0	18	9
1217	osa-miR2118c	TTCCCGATGCCTCCTATTCCTA	LOC_Os05g34220.2	4	671-692	683	0	18	9
1218	osa-miR2118c	TTCCCGATGCCTCCTATTCCTA	MSTRG.29394.1	3.5	106-127	118	0	65	65
1219	osa-miR2118c	TTCCCGATGCCTCCTATTCCTA	MSTRG.9266.1	2	286-307	298	0	84	84
1220	osa-miR2118c	TTCCCGATGCCTCCTATTCCTA	MSTRG.8777.1	3	106-127	118	4	1	1
1221	osa-miR2118c	TTCCCGATGCCTCCTATTCCTA	MSTRG.9258.1	4	43-64	55	1	2	2
1222	osa-miR2118c	TTCCCGATGCCTCCTATTCCTA	MSTRG.8970.1	4	905-926	917	0	8	8
1223	osa-miR2118c	TTCCCGATGCCTCCTATTCCTA	MSTRG.23774.1	3	10-31	22	0	29	29
1224	osa-miR2118c	TTCCCGATGCCTCCTATTCCTA	MSTRG.23766.1	3.5	267-288	279	0	14	14
1225	osa-miR2118c	TTCCCGATGCCTCCTATTCCTA	MSTRG.23756.1	4	142-163	154	1	16	16
1226	osa-miR2118c	TTCCCGATGCCTCCTATTCCTA	MSTRG.28057.1	4	66-87	78	0	7	7
1227	osa-miR2118d	TTCCTGATGCCTCCCATGCCTA	LOC_Os04g30610.1	4	497-518	509	0	13	13
1228	osa-miR2118d	TTCCTGATGCCTCCCATGCCTA	MSTRG.17803.1	3	316-337	328	0	253	253
1229	osa-miR2118d	TTCCTGATGCCTCCCATGCCTA	MSTRG.17799.1	4	738-759	750	0	36	36
1230	osa-miR2118d	TTCCTGATGCCTCCCATGCCTA	MSTRG.29394.1	3	106-127	118	0	65	65
1231	osa-miR2118d	TTCCTGATGCCTCCCATGCCTA	MSTRG.6412.3	4	904-925	916	4	1	0.5
1232	osa-miR2118d	TTCCTGATGCCTCCCATGCCTA	MSTRG.6412.1	4	1140-1161	1152	4	1	0.5
1233	osa-miR2118d	TTCCTGATGCCTCCCATGCCTA	MSTRG.9237.1	3	28-49	40	0	4	4
1234	osa-miR2118d	TTCCTGATGCCTCCCATGCCTA	MSTRG.9266.1	3	286-307	298	0	84	84
1235	osa-miR2118d	TTCCTGATGCCTCCCATGCCTA	MSTRG.9256.1	3.5	114-135	126	0	72	72
1236	osa-miR2118d	TTCCTGATGCCTCCCATGCCTA	MSTRG.9254.1	3.5	401-422	413	2	3	3

1237	osa-miR2118d	TTCCTGATGCCTCCCATGCCTA	MSTRG.9791.1	4	81-102	93	1	4	4
1238	osa-miR2118d	TTCCTGATGCCTCCCATGCCTA	MSTRG.9246.1	4	115-136	127	0	46	46
1239	osa-miR2118d	TTCCTGATGCCTCCCATGCCTA	LOC_Os12g39430.1	4	697-718	709	0	8	8
1240	osa-miR2118d	TTCCTGATGCCTCCCATGCCTA	MSTRG.9308.1	4	49-70	61	0	30	30
1241	osa-miR2118d	TTCCTGATGCCTCCCATGCCTA	MSTRG.23774.1	3	10-31	22	0	29	29
1242	osa-miR2118d	TTCCTGATGCCTCCCATGCCTA	MSTRG.23338.1	4	150-171	162	0	12	12
1243	osa-miR2118d	TTCCTGATGCCTCCCATGCCTA	MSTRG.25143.1	3.5	143-164	155	0	5	5
1244	osa-miR2118d	TTCCTGATGCCTCCCATGCCTA	MSTRG.25110.1	4	712-733	724	0	3	3
1245	osa-miR2118d	TTCCTGATGCCTCCCATGCCTA	MSTRG.7409.1	2.5	746-767	758	2	40	40
1246	osa-miR2118d	TTCCTGATGCCTCCCATGCCTA	MSTRG.7294.1	3	160-181	172	0	16	16
1247	osa-miR2118e	TTCCCAATGCCTCCCATGCCTA	MSTRG.17799.1	3	738-759	750	0	36	36
1248	osa-miR2118e	TTCCCAATGCCTCCCATGCCTA	MSTRG.17791.1	3	1106-1127	1118	0	5	5
1249	osa-miR2118e	TTCCCAATGCCTCCCATGCCTA	MSTRG.17802.1	4	38-59	50	0	18	18
1250	osa-miR2118e	TTCCCAATGCCTCCCATGCCTA	LOC_Os05g34220.1	3.5	671-692	683	0	18	9
1251	osa-miR2118e	TTCCCAATGCCTCCCATGCCTA	LOC_Os05g34220.2	3.5	671-692	683	0	18	9
1252	osa-miR2118e	TTCCCAATGCCTCCCATGCCTA	MSTRG.29394.1	4	106-127	118	0	65	65
1253	osa-miR2118e	TTCCCAATGCCTCCCATGCCTA	MSTRG.9258.1	3	43-64	55	1	2	2
1254	osa-miR2118e	TTCCCAATGCCTCCCATGCCTA	MSTRG.9266.1	4	286-307	298	0	84	84
1255	osa-miR2118e	TTCCCAATGCCTCCCATGCCTA	MSTRG.307.1	4	110-131	122	0	113	113
1256	osa-miR2118e	TTCCCAATGCCTCCCATGCCTA	MSTRG.28057.1	3.5	66-87	78	0	7	7
1257	osa-miR2118f	TTCCTGATGCCTCCCATTCCTA	MSTRG.17799.1	3.5	738-759	750	0	36	36
1258	osa-miR2118f	TTCCTGATGCCTCCCATTCCTA	MSTRG.19743.1	4	175-196	187	0	75	75
1259	osa-miR2118f	TTCCTGATGCCTCCCATTCCTA	MSTRG.29394.1	4	106-127	118	0	65	65
1260	osa-miR2118f	TTCCTGATGCCTCCCATTCCTA	MSTRG.6412.3	3	904-925	916	4	1	0.5
1261	osa-miR2118f	TTCCTGATGCCTCCCATTCCTA	MSTRG.6412.1	3	1140-1161	1152	4	1	0.5
1262	osa-miR2118f	TTCCTGATGCCTCCCATTCCTA	MSTRG.9237.1	4	28-49	40	0	4	4
1263	osa-miR2118f	TTCCTGATGCCTCCCATTCCTA	MSTRG.9266.1	4	286-307	298	0	84	84
1264	osa-miR2118f	TTCCTGATGCCTCCCATTCCTA	LOC_Os12g26270.1	4	86-107	98	1	3	3
1265	osa-miR2118f	TTCCTGATGCCTCCCATTCCTA	MSTRG.23774.1	2	10-31	22	0	29	29
1266	osa-miR2118f	TTCCTGATGCCTCCCATTCCTA	MSTRG.23338.1	3	150-171	162	0	12	12
1267	osa-miR2118f	TTCCTGATGCCTCCCATTCCTA	MSTRG.25143.1	4	143-164	155	0	5	5
1268	osa-miR2118f	TTCCTGATGCCTCCCATTCCTA	MSTRG.7409.1	3.5	746-767	758	2	40	40
1269	osa-miR2118f	TTCCTGATGCCTCCCATTCCTA	MSTRG.8632.1	4	110-131	122	1	2	2
1270	osa-miR2118f	TTCCTGATGCCTCCCATTCCTA	MSTRG.7294.1	4	160-181	172	0	16	16
1271	osa-miR2118g_1ss5TC	TTCCCAATGCCTCCCATTCCTA	MSTRG.3077.1	4	179-200	191	0	931	931
1272	osa-miR2118g_1ss5TC	TTCCCAATGCCTCCCATTCCTA	MSTRG.17799.1	2.5	738-759	750	0	36	36
1273	osa-miR2118g_1ss5TC	TTCCCAATGCCTCCCATTCCTA	MSTRG.17802.1	3.5	38-59	50	0	18	18
1274	osa-miR2118g_1ss5TC	TTCCCAATGCCTCCCATTCCTA	MSTRG.17791.1	4	1106-1127	1118	0	5	5
1275	osa-miR2118g_1ss5TC	TTCCCAATGCCTCCCATTCCTA	LOC_Os05g34220.1	3	671-692	683	0	18	9
1276	osa-miR2118g_1ss5TC	TTCCCAATGCCTCCCATTCCTA	LOC_Os05g34220.2	3	671-692	683	0	18	9
1277	osa-miR2118g_1ss5TC	TTCCCAATGCCTCCCATTCCTA	LOC_Os08g42700.1	3.5	1456-1477	1468	0	13	4.333333333
1278	osa-miR2118g_1ss5TC	TTCCCAATGCCTCCCATTCCTA	MSTRG.28782.2	3.5	2100-2121	2112	0	13	4.333333333
1279	osa-miR2118g_1ss5TC	TTCCCAATGCCTCCCATTCCTA	MSTRG.9258.1	4	43-64	55	1	2	2
1280	osa-miR2118g_1ss5TC	TTCCCAATGCCTCCCATTCCTA	MSTRG.23766.1	3.5	267-288	279	0	14	14
1281	osa-miR2118g_1ss5TC	TTCCCAATGCCTCCCATTCCTA	MSTRG.28057.1	2.5	66-87	78	0	7	7
1282	osa-miR2118h	TTCCTGATGCCTCTCATTCCTA	MSTRG.17848.7	0	1587-1608	1599	0	8	4
1283	osa-miR2118h	TTCCTGATGCCTCTCATTCCTA	MSTRG.17848.8	0	2838-2859	2850	0	8	4
1284	osa-miR2118h	TTCCTGATGCCTCTCATTCCTA	MSTRG.17799.1	4	738-759	750	0	36	36
1285	osa-miR2118h	TTCCTGATGCCTCTCATTCCTA	MSTRG.29456.3	3.5	695-715	707	4	1	0.333333333
1286	osa-miR2118h	TTCCTGATGCCTCTCATTCCTA	MSTRG.29456.1	3.5	531-551	543	4	1	0.333333333
1287	osa-miR2118h	TTCCTGATGCCTCTCATTCCTA	LOC_Os09g16520.1	3.5	594-614	606	4	1	0.333333333
1288	osa-miR2118h	TTCCTGATGCCTCTCATTCCTA	MSTRG.6412.3	3	904-925	916	4	1	0.5
1289	osa-miR2118h	TTCCTGATGCCTCTCATTCCTA	MSTRG.6412.1	3	1140-1161	1152	4	1	0.5
1290	osa-miR2118h	TTCCTGATGCCTCTCATTCCTA	LOC_Os12g26270.1	3	86-107	98	1	3	3
1291	osa-miR2118h	TTCCTGATGCCTCTCATTCCTA	MSTRG.9051.1	4	61-82	73	0	58	58
1292	osa-miR2118h	TTCCTGATGCCTCTCATTCCTA	MSTRG.23774.1	2.5	10-31	22	0	29	29
1293	osa-miR2118h	TTCCTGATGCCTCTCATTCCTA	MSTRG.23338.1	3.5	150-171	162	0	12	12
1294	osa-miR2118h	TTCCTGATGCCTCTCATTCCTA	MSTRG.7409.1	2.5	746-767	758	2	40	40
1295	osa-miR2118h	TTCCTGATGCCTCTCATTCCTA	MSTRG.8632.1	3	110-131	122	1	2	2

1296	osa-miR2118h	TTCCTGATGCCTCTCATTCCTA	MSTRG.7311.1	3.5	46-67	58	1	8	8
1297	osa-miR2118i	TTCCTAGTGCCTCCCATTCCTA	MSTRG.25103.1	3.5	88-109	100	0	34	34
1298	osa-miR2118i	TTCCTAGTGCCTCCCATTCCTA	LOC_Os06g46640.1	3.5	99-120	111	1	3	3
1299	osa-miR2118l	TTCCTAATGCTTCCCATTCCTA	MSTRG.9062.1	4	182-203	194	1	2	2
1300	osa-miR2118o_1ss1CT	TTCCTGATGCCTCCCAAGCCTA	LOC_Os04g25740.1	3.5	1875-1896	1887	0	18	18
1301	osa-miR2118o_1ss1CT	TTCCTGATGCCTCCCAAGCCTA	MSTRG.17848.3	1	833-854	845	4	1	1
1302	osa-miR2118o_1ss1CT	TTCCTGATGCCTCCCAAGCCTA	MSTRG.17803.1	2	316-337	328	0	253	253
1303	osa-miR2118o_1ss1CT	TTCCTGATGCCTCCCAAGCCTA	MSTRG.29394.1	2	106-127	118	0	65	65
1304	osa-miR2118o_1ss1CT	TTCCTGATGCCTCCCAAGCCTA	MSTRG.9254.1	2.5	401-422	413	2	3	3
1305	osa-miR2118o_1ss1CT	TTCCTGATGCCTCCCAAGCCTA	MSTRG.9237.1	3	28-49	40	0	4	4
1306	osa-miR2118o_1ss1CT	TTCCTGATGCCTCCCAAGCCTA	MSTRG.9791.1	3	81-102	93	1	4	4
1307	osa-miR2118o_1ss1CT	TTCCTGATGCCTCCCAAGCCTA	MSTRG.9266.1	3	286-307	298	0	84	84
1308	osa-miR2118o_1ss1CT	TTCCTGATGCCTCCCAAGCCTA	MSTRG.9026.1	4	75-96	87	0	91	91
1309	osa-miR2118o_1ss1CT	TTCCTGATGCCTCCCAAGCCTA	MSTRG.9246.1	4	115-136	127	0	46	46
1310	osa-miR2118o_1ss1CT	TTCCTGATGCCTCCCAAGCCTA	MSTRG.9308.1	4	49-70	61	0	30	30
1311	osa-miR2118o_1ss1CT	TTCCTGATGCCTCCCAAGCCTA	MSTRG.23338.1	4	150-171	162	0	12	12
1312	osa-miR2118o_1ss1CT	TTCCTGATGCCTCCCAAGCCTA	MSTRG.23774.1	4	10-31	22	0	29	29
1313	osa-miR2118o_1ss1CT	TTCCTGATGCCTCCCAAGCCTA	MSTRG.25143.1	2.5	143-164	155	0	5	5
1314	osa-miR2118o_1ss1CT	TTCCTGATGCCTCCCAAGCCTA	MSTRG.25110.1	3	712-733	724	0	3	3
1315	osa-miR2118o_1ss1CT	TTCCTGATGCCTCCCAAGCCTA	MSTRG.7294.1	2	160-181	172	0	16	16
1316	osa-miR2118o_1ss1CT	TTCCTGATGCCTCCCAAGCCTA	MSTRG.7409.1	3.5	746-767	758	2	40	40
1317	osa-miR2118p	TTCCCGATGCCTCCCATGCCTA	LOC_Os01g58510.1	4	509-530	521	2	2	0.666666667
1318	osa-miR2118p	TTCCCGATGCCTCCCATGCCTA	LOC_Os01g57710.1	4	1193-1215	1205	2	4	1.333333333
1319	osa-miR2118p	TTCCCGATGCCTCCCATGCCTA	LOC_Os01g57710.3	4	1249-1271	1261	1	4	1.333333333
1320	osa-miR2118p	TTCCCGATGCCTCCCATGCCTA	LOC_Os01g57710.2	4	1315-1337	1327	1	4	1.333333333
1321	osa-miR2118p	TTCCCGATGCCTCCCATGCCTA	LOC_Os01g71950.1	4	560-581	572	2	2	0.666666667
1322	osa-miR2118p	TTCCCGATGCCTCCCATGCCTA	MSTRG.3877.2	4	1135-1156	1147	2	2	0.666666667
1323	osa-miR2118p	TTCCCGATGCCTCCCATGCCTA	LOC_Os04g30610.1	3	497-518	509	0	13	13
1324	osa-miR2118p	TTCCCGATGCCTCCCATGCCTA	MSTRG.17799.1	3	738-759	750	0	36	36
1325	osa-miR2118p	TTCCCGATGCCTCCCATGCCTA	MSTRG.17791.1	4	1106-1127	1118	0	5	5
1326	osa-miR2118p	TTCCCGATGCCTCCCATGCCTA	MSTRG.17802.1	4	38-59	50	0	18	18
1327	osa-miR2118p	TTCCCGATGCCTCCCATGCCTA	MSTRG.29394.1	2	106-127	118	0	65	65
1328	osa-miR2118p	TTCCCGATGCCTCCCATGCCTA	MSTRG.9266.1	2	286-307	298	0	84	84
1329	osa-miR2118p	TTCCCGATGCCTCCCATGCCTA	MSTRG.9258.1	4	43-64	55	1	2	2
1330	osa-miR2118p	TTCCCGATGCCTCCCATGCCTA	MSTRG.8970.1	4	905-926	917	0	8	8
1331	osa-miR2121a_L+1R-1	AAAAACGGAGCGGTCCATTAGCGC	MSTRG.16616.1	1	3100-3123	3114	4	1	0.5
1332	osa-miR2121a_L+1R-1	AAAAACGGAGCGGTCCATTAGCGC	MSTRG.16616.8	1	3352-3375	3366	4	1	0.5
1333	osa-MIR2122-p3_2ss18TC21TC	GCTGAGGTGTCTAAGTTCAGCGCC	MSTRG.27030.3	3.5	1253-1276	1267	4	1	0.333333333
1334	osa-MIR2122-p3_2ss18TC21TC	GCTGAGGTGTCTAAGTTCAGCGCC	MSTRG.27030.1	3.5	1816-1839	1830	4	1	0.333333333
1335	osa-MIR2122-p3_2ss18TC21TC	GCTGAGGTGTCTAAGTTCAGCGCC	MSTRG.27030.2	3.5	2166-2189	2180	4	1	0.333333333
1336	osa-MIR2122-p5_1ss2CT	GTTGAACTTAGACACCTCAGCGCC	MSTRG.6797.13	1	2006-2029	2020	4	1	0.1
1337	osa-MIR2122-p5_1ss2CT	GTTGAACTTAGACACCTCAGCGCC	MSTRG.6797.12	1	2569-2592	2583	4	1	0.1
1338	osa-MIR2122-p5_1ss2CT	GTTGAACTTAGACACCTCAGCGCC	MSTRG.6797.10	1	2802-2825	2816	4	1	0.1
1339	osa-MIR2122-p5_1ss2CT	GTTGAACTTAGACACCTCAGCGCC	MSTRG.6797.9	1	3389-3412	3403	4	1	0.1
1340	osa-MIR2122-p5_1ss2CT	GTTGAACTTAGACACCTCAGCGCC	MSTRG.6797.5	1	3675-3698	3689	4	1	0.1
1341	osa-MIR2122-p5_1ss2CT	GTTGAACTTAGACACCTCAGCGCC	MSTRG.6797.6	1	3746-3769	3760	4	1	0.1
1342	osa-MIR2122-p5_1ss2CT	GTTGAACTTAGACACCTCAGCGCC	MSTRG.6797.7	1	3770-3793	3784	4	1	0.1
1343	osa-MIR2122-p5_1ss2CT	GTTGAACTTAGACACCTCAGCGCC	MSTRG.6797.4	1	3748-3771	3762	4	1	0.1
1344	osa-MIR2122-p5_1ss2CT	GTTGAACTTAGACACCTCAGCGCC	MSTRG.6797.2	1	3979-4002	3993	4	1	0.1
1345	osa-MIR2122-p5_1ss2CT	GTTGAACTTAGACACCTCAGCGCC	MSTRG.6797.3	1	3833-3856	3847	4	1	0.1
1346	osa-MIR2122-p5_1ss2CT	GTTGAACTTAGACACCTCAGCGCC	MSTRG.27030.3	3.5	1363-1386	1377	2	5	1.666666667
1347	osa-MIR2122-p5_1ss2CT	GTTGAACTTAGACACCTCAGCGCC	MSTRG.27030.1	3.5	1926-1949	1940	2	5	1.666666667
1348	osa-MIR2122-p5_1ss2CT	GTTGAACTTAGACACCTCAGCGCC	MSTRG.27030.2	3.5	2276-2299	2290	2	5	1.666666667
1349	osa-miR2275a	TTTGGTTTCCTCCAATATCTCA	LOC_Os01g67040.2	4	293-313	305	0	5	1.25
1350	osa-miR2275a	TTTGGTTTCCTCCAATATCTCA	MSTRG.3576.1	4	425-445	437	0	5	1.25
1351	osa-miR2275a	TTTGGTTTCCTCCAATATCTCA	LOC_Os01g67040.1	4	425-445	437	0	5	1.25
1352	osa-miR2275a	TTTGGTTTCCTCCAATATCTCA	MSTRG.3576.4	4	352-372	364	0	5	1.25
1353	osa-miR2275a	TTTGGTTTCCTCCAATATCTCA	MSTRG.17506.1	4	204-225	216	0	142	142
1354	osa-miR2275a	TTTGGTTTCCTCCAATATCTCA	MSTRG.20022.1	3.5	903-924	915	0	704	704

1355	osa-miR2275a	TTTGGTTTCCTCCAATATCTCA	MSTRG.5300.1	3	13-35	25	0	5668	5668
1356	osa-miR2275a	TTTGGTTTCCTCCAATATCTCA	MSTRG.4706.2	4	734-755	746	0	10909	5454.5
1357	osa-miR2275a	TTTGGTTTCCTCCAATATCTCA	MSTRG.4706.1	4	683-704	695	0	10909	5454.5
1358	osa-miR2275a	TTTGGTTTCCTCCAATATCTCA	MSTRG.7148.1	2.5	1669-1690	1681	0	6011	6011
1359	osa-miR2275a	TTTGGTTTCCTCCAATATCTCA	MSTRG.7150.1	2.5	3697-3718	3709	0	373	373
1360	osa-miR2275a	TTTGGTTTCCTCCAATATCTCA	MSTRG.9117.1	4	1724-1745	1736	0	220	220
1361	osa-miR2275a	TTTGGTTTCCTCCAATATCTCA	MSTRG.25059.1	4	3665-3686	3677	0	458	458
1362	osa-miR2275a	TTTGGTTTCCTCCAATATCTCA	MSTRG.23851.1	4	8-29	20	0	422	422
1363	osa-miR2275a	TTTGGTTTCCTCCAATATCTCA	MSTRG.7345.2	4	80-101	92	0	199	66.33333333
1364	osa-miR2275a	TTTGGTTTCCTCCAATATCTCA	MSTRG.7345.3	4	80-101	92	0	199	66.33333333
1365	osa-miR2275a	TTTGGTTTCCTCCAATATCTCA	MSTRG.7345.1	4	80-101	92	0	199	66.33333333
1366	osa-miR2275a	TTTGGTTTCCTCCAATATCTCA	MSTRG.7347.2	4	165-186	177	0	117	117
1367	osa-miR2275a	TTTGGTTTCCTCCAATATCTCA	MSTRG.7347.1	4	165-186	177	0	549	549
1368	osa-MIR2275c-p3	TTGTTTTTCTCCAATATCTCA	LOC_Os01g41610.2	4	432-452	443	4	1	0.5
1369	osa-MIR2275c-p3	TTGTTTTTCTCCAATATCTCA	LOC_Os01g41610.1	4	239-259	250	4	1	0.5
1370	osa-MIR2275c-p3	TTGTTTTTCTCCAATATCTCA	MSTRG.10455.1	0.5	1459-1479	1470	2	28	28
1371	osa-MIR2275c-p3	TTGTTTTTCTCCAATATCTCA	MSTRG.10341.1	2	1241-1261	1252	2	2	2
1372	osa-MIR2275c-p3	TTGTTTTTCTCCAATATCTCA	MSTRG.10342.1	2	1920-1940	1931	2	21	10.5
1373	osa-MIR2275c-p3	TTGTTTTTCTCCAATATCTCA	MSTRG.10337.1	3	236-256	247	2	76	76
1374	osa-MIR2275c-p3	TTGTTTTTCTCCAATATCTCA	MSTRG.14324.5	3.5	1846-1866	1857	2	5	1
1375	osa-MIR2275c-p3	TTGTTTTTCTCCAATATCTCA	MSTRG.14324.4	3.5	1811-1831	1822	2	5	1
1376	osa-MIR2275c-p3	TTGTTTTTCTCCAATATCTCA	MSTRG.14324.3	3.5	1841-1861	1852	2	5	1
1377	osa-MIR2275c-p3	TTGTTTTTCTCCAATATCTCA	LOC_Os03g19480.1	3.5	1846-1866	1857	2	5	1
1378	osa-MIR2275c-p3	TTGTTTTTCTCCAATATCTCA	MSTRG.14324.2	3.5	2102-2122	2113	2	5	1
1379	osa-MIR2275c-p3	TTGTTTTTCTCCAATATCTCA	MSTRG.17514.1	1.5	41-61	52	2	162	162
1380	osa-MIR2275c-p3	TTGTTTTTCTCCAATATCTCA	MSTRG.17506.1	4	204-224	215	4	1	1
1381	osa-MIR2275c-p3	TTGTTTTTCTCCAATATCTCA	MSTRG.9115.2	3	3509-3529	3520	2	2	0.666666667
1382	osa-MIR2275c-p3	TTGTTTTTCTCCAATATCTCA	MSTRG.9115.1	3	3570-3590	3581	2	2	0.666666667
1383	osa-MIR2275c-p3	TTGTTTTTCTCCAATATCTCA	MSTRG.9115.3	3	3557-3577	3568	2	2	0.666666667
1384	osa-MIR2275c-p3	TTGTTTTTCTCCAATATCTCA	MSTRG.9359.1	4	3138-3158	3149	2	42	42
1385	osa-MIR2275c-p3	TTGTTTTTCTCCAATATCTCA	MSTRG.22726.1	4	366-386	377	2	34	34
1386	osa-MIR2275c-p3	TTGTTTTTCTCCAATATCTCA	MSTRG.23853.1	2	210-230	221	2	6	6
1387	osa-MIR2275c-p3	TTGTTTTTCTCCAATATCTCA	MSTRG.26882.1	2	122-143	134	2	6	6
1388	osa-MIR2275c-p3	TTGTTTTTCTCCAATATCTCA	MSTRG.7345.2	4	80-100	91	4	1	0.333333333
1389	osa-MIR2275c-p3	TTGTTTTTCTCCAATATCTCA	MSTRG.7345.3	4	80-100	91	4	1	0.333333333
1390	osa-MIR2275c-p3	TTGTTTTTCTCCAATATCTCA	MSTRG.7345.1	4	80-100	91	4	1	0.333333333
1391	osa-MIR2275c-p3	TTGTTTTTCTCCAATATCTCA	MSTRG.7349.1	4	531-551	542	2	7	7
1392	osa-MIR2275c-p3	TTGTTTTTCTCCAATATCTCA	MSTRG.7347.1	4	165-185	176	2	4	4
1393	osa-MIR2275c-p3	TTGTTTTTCTCCAATATCTCA	MSTRG.7464.1	4	4543-4563	4554	2	2	2
1394	osa-miR2275d	CTTGTTTTTCTCCAATATCTCA	LOC_Os01g41610.2	4	432-453	444	3	2	1
1395	osa-miR2275d	CTTGTTTTTCTCCAATATCTCA	LOC_Os01g41610.1	4	239-260	251	3	2	1
1396	osa-miR2275d	CTTGTTTTTCTCCAATATCTCA	MSTRG.10455.1	1.5	1459-1480	1471	0	5360	5360
1397	osa-miR2275d	CTTGTTTTTCTCCAATATCTCA	MSTRG.10341.1	2	1241-1262	1253	2	263	263
1398	osa-miR2275d	CTTGTTTTTCTCCAATATCTCA	MSTRG.10342.1	3	1920-1941	1932	0	3309	1654.5
1399	osa-miR2275d	CTTGTTTTTCTCCAATATCTCA	MSTRG.10337.1	4	236-257	248	0	16266	16266
1400	osa-miR2275d	CTTGTTTTTCTCCAATATCTCA	MSTRG.17514.1	2.5	41-62	53	0	29455	29455
1401	osa-miR2275d	CTTGTTTTTCTCCAATATCTCA	MSTRG.28209.3	3.5	236-257	248	4	1	0.25
1402	osa-miR2275d	CTTGTTTTTCTCCAATATCTCA	MSTRG.28209.4	3.5	236-257	248	4	1	0.25
1403	osa-miR2275d	CTTGTTTTTCTCCAATATCTCA	LOC_Os08g33076.1	3.5	477-498	489	4	1	0.25
1404	osa-miR2275d	CTTGTTTTTCTCCAATATCTCA	MSTRG.28209.1	3.5	598-619	610	4	1	0.25
1405	osa-miR2275d	CTTGTTTTTCTCCAATATCTCA	MSTRG.9115.2	4	3509-3530	3521	0	1443	481
1406	osa-miR2275d	CTTGTTTTTCTCCAATATCTCA	MSTRG.9115.1	4	3570-3591	3582	0	1443	481
1407	osa-miR2275d	CTTGTTTTTCTCCAATATCTCA	MSTRG.9115.3	4	3557-3578	3569	0	1443	481
1408	osa-miR2275d	CTTGTTTTTCTCCAATATCTCA	MSTRG.23853.1	3	210-231	222	0	1540	1540
1409	osa-miR2275d	CTTGTTTTTCTCCAATATCTCA	MSTRG.26882.1	3	122-144	135	0	1828	1828
1410	osa-miR2863b	TTCGTTTATTTGGACTAGAGT	MSTRG.22723.2	4	1290-1310	1301	2	3	1
1411	osa-miR2863b	TTCGTTTATTTGGACTAGAGT	LOC_Os06g14040.1	4	1369-1389	1380	2	3	1
1412	osa-miR2863b	TTCGTTTATTTGGACTAGAGT	MSTRG.22723.3	4	1476-1496	1487	2	3	1
1413	osa-miR2864.1	TTTTGCTGCCCTTGTTTTGCA	MSTRG.19759.4	4	2002-2021	2012	4	1	0.25

1414	osa-miR2864.1	TTTTGCTGCCCTTGTTTTGCA	MSTRG.19759.2	4	1968-1987	1978	4	1	0.25
1415	osa-miR2864.1	TTTTGCTGCCCTTGTTTTGCA	LOC_Os05g06300.1	4	2373-2392	2383	4	1	0.25
1416	osa-miR2864.1	TTTTGCTGCCCTTGTTTTGCA	MSTRG.19759.1	4	2317-2336	2327	4	1	0.25
1417	osa-miR2870	TAATCAGTTTGGGGAGACAAA	MSTRG.22210.3	4	1082-1102	1093	4	1	1
1418	osa-MIR2873a-p5_1ss6GT	TATTTTTGAGAATTTTTG	MSTRG.3424.8	4	1517-1534	1525	2	2	0.222222222
1419	osa-MIR2873a-p5_1ss6GT	TATTTTTGAGAATTTTTG	MSTRG.3424.5	4	1045-1062	1053	2	2	0.222222222
1420	osa-MIR2873a-p5_1ss6GT	TATTTTTGAGAATTTTTG	LOC_Os01g64770.2	4	1127-1144	1135	2	2	0.222222222
1421	osa-MIR2873a-p5_1ss6GT	TATTTTTGAGAATTTTTG	MSTRG.3424.6	4	994-1011	1002	2	2	0.222222222
1422	osa-MIR2873a-p5_1ss6GT	TATTTTTGAGAATTTTTG	LOC_Os01g64770.1	4	1139-1156	1147	2	2	0.222222222
1423	osa-MIR2873a-p5_1ss6GT	TATTTTTGAGAATTTTTG	MSTRG.3424.7	4	1621-1638	1629	2	2	0.222222222
1424	osa-MIR2873a-p5_1ss6GT	TATTTTTGAGAATTTTTG	LOC_Os01g64770.3	4	1310-1327	1318	2	2	0.222222222
1425	osa-MIR2873a-p5_1ss6GT	TATTTTTGAGAATTTTTG	MSTRG.3424.4	4	1175-1192	1183	2	2	0.222222222
1426	osa-MIR2873a-p5_1ss6GT	TATTTTTGAGAATTTTTG	LOC_Os01g64770.5	4	1387-1404	1395	2	2	0.222222222
1427	osa-MIR2873a-p5_1ss6GT	TATTTTTGAGAATTTTTG	LOC_Os01g69120.1	4	1170-1188	1179	4	1	0.25
1428	osa-MIR2873a-p5_1ss6GT	TATTTTTGAGAATTTTTG	LOC_Os01g69120.3	4	1170-1188	1179	4	1	0.25
1429	osa-MIR2873a-p5_1ss6GT	TATTTTTGAGAATTTTTG	MSTRG.3722.1	4	1170-1188	1179	4	1	0.25
1430	osa-MIR2873a-p5_1ss6GT	TATTTTTGAGAATTTTTG	LOC_Os01g69120.2	4	1170-1188	1179	4	1	0.25
1431	osa-MIR2873a-p5_1ss6GT	TATTTTTGAGAATTTTTG	LOC_Os06g05740.1	4	814-831	822	2	2	1
1432	osa-MIR2873a-p5_1ss6GT	TATTTTTGAGAATTTTTG	MSTRG.22208.1	4	858-875	866	2	2	1
1433	osa-MIR2873a-p5_1ss6GT	TATTTTTGAGAATTTTTG	MSTRG.29422.2	3.5	1909-1926	1917	4	1	0.333333333
1434	osa-MIR2873a-p5_1ss6GT	TATTTTTGAGAATTTTTG	MSTRG.29422.4	3.5	2451-2468	2459	4	1	0.333333333
1435	osa-MIR2873a-p5_1ss6GT	TATTTTTGAGAATTTTTG	MSTRG.29422.11	3.5	2590-2607	2598	4	1	0.333333333
1436	osa-MIR2873a-p5_1ss6GT	TATTTTTGAGAATTTTTG	LOC_Os01g13530.1	4	738-755	746	3	2	2
1437	osa-MIR2873a-p5_1ss6GT	TATTTTTGAGAATTTTTG	LOC_Os03g58130.1	3.5	568-585	576	2	6	1.5
1438	osa-MIR2873a-p5_1ss6GT	TATTTTTGAGAATTTTTG	MSTRG.16176.4	3.5	479-496	487	2	6	1.5
1439	osa-MIR2873a-p5_1ss6GT	TATTTTTGAGAATTTTTG	MSTRG.16176.3	3.5	494-511	502	2	6	1.5
1440	osa-MIR2873a-p5_1ss6GT	TATTTTTGAGAATTTTTG	MSTRG.16176.1	3.5	568-585	576	2	6	1.5
1441	osa-MIR2873a-p5_1ss6GT	TATTTTTGAGAATTTTTG	LOC_Os06g40040.1	3	407-424	415	3	2	1
1442	osa-MIR2873a-p5_1ss6GT	TATTTTTGAGAATTTTTG	LOC_Os06g40040.2	3	407-424	415	2	2	1
1443	osa-MIR2873a-p5_1ss6GT	TATTTTTGAGAATTTTTG	MSTRG.23967.3	3	381-398	389	4	1	0.333333333
1444	osa-MIR2873a-p5_1ss6GT	TATTTTTGAGAATTTTTG	LOC_Os06g43130.1	3	639-656	647	4	1	0.333333333
1445	osa-MIR2873a-p5_1ss6GT	TATTTTTGAGAATTTTTG	MSTRG.23967.1	3	1069-1086	1077	4	1	0.333333333
1446	osa-miR2874_L-3	TGAACAGTGTCAAACAGTGTC	MSTRG.29189.1	4	6334-6353	6344	2	2	0.285714286
1447	osa-miR2874_L-3	TGAACAGTGTCAAACAGTGTC	LOC_Os09g07900.1	4	5941-5960	5951	2	2	0.285714286
1448	osa-miR2874_L-3	TGAACAGTGTCAAACAGTGTC	MSTRG.29189.7	4	6168-6187	6178	2	2	0.285714286
1449	osa-miR2874_L-3	TGAACAGTGTCAAACAGTGTC	MSTRG.29189.6	4	6176-6195	6186	2	2	0.285714286
1450	osa-miR2874_L-3	TGAACAGTGTCAAACAGTGTC	MSTRG.29189.3	4	6259-6278	6269	2	2	0.285714286
1451	osa-miR2874_L-3	TGAACAGTGTCAAACAGTGTC	MSTRG.29189.4	4	6250-6269	6260	2	2	0.285714286
1452	osa-miR2874_L-3	TGAACAGTGTCAAACAGTGTC	MSTRG.29189.2	4	6314-6333	6324	2	2	0.285714286
1453	osa-MIR2905-p5_1ss21AG	TGTCACTGACATGTGGGCCCG	LOC_Os04g44850.1	3.5	1468-1488	1479	2	3	3
1454	osa-MIR2905-p5_1ss21AG	TGTCACTGACATGTGGGCCCG	LOC_Os06g44500.1	4	1331-1351	1342	4	1	1
1455	osa-miR319a-3p.2-3p_R+1	TTGGACTGAAGGGTGCTCCCT	LOC_Os01g55100.1	3	1303-1322	1313	0	21	21
1456	osa-miR319a-3p.2-3p_R+1	TTGGACTGAAGGGTGCTCCCT	LOC_Os01g59660.1	4	1259-1279	1270	4	1	0.25
1457	osa-miR319a-3p.2-3p_R+1	TTGGACTGAAGGGTGCTCCCT	LOC_Os01g59660.4	4	1179-1199	1190	4	1	0.25
1458	osa-miR319a-3p.2-3p_R+1	TTGGACTGAAGGGTGCTCCCT	LOC_Os01g59660.3	4	1257-1277	1268	4	1	0.25
1459	osa-miR319a-3p.2-3p_R+1	TTGGACTGAAGGGTGCTCCCT	LOC_Os01g59660.2	4	1347-1367	1358	4	1	0.25
1460	osa-miR319a-3p.2-3p_R+1	TTGGACTGAAGGGTGCTCCCT	LOC_Os01g11550.1	3	1654-1673	1664	0	51	25.5
1461	osa-miR319a-3p.2-3p_R+1	TTGGACTGAAGGGTGCTCCCT	MSTRG.711.1	3	2111-2130	2121	0	51	25.5
1462	osa-miR319a-3p.2-3p_R+1	TTGGACTGAAGGGTGCTCCCT	LOC_Os03g57190.1	1.5	1182-1201	1192	2	3	3
1463	osa-miR319a-3p.2-3p_R+1	TTGGACTGAAGGGTGCTCCCT	LOC_Os07g05720.1	1.5	1330-1349	1340	2	2	2
1464	osa-miR390-5p	AAGCTCAGGAGGGATAGCGCC	LOC_Os02g10100.1	2	2170-2190	2181	1	2	1
1465	osa-miR390-5p	AAGCTCAGGAGGGATAGCGCC	MSTRG.10259.1	2	2379-2399	2390	1	2	1
1466	osa-miR393a	TCCAAAGGGATCGCATTGATC	LOC_Os04g32460.2	1	1823-1842	1833	2	21	7
1467	osa-miR393a	TCCAAAGGGATCGCATTGATC	LOC_Os04g32460.1	1	2225-2244	2235	2	21	7
1468	osa-miR393a	TCCAAAGGGATCGCATTGATC	MSTRG.17631.1	1	2192-2211	2202	2	21	7
1469	osa-miR393a	TCCAAAGGGATCGCATTGATC	LOC_Os05g05800.1	1	1698-1717	1708	0	42	21
1470	osa-miR393a	TCCAAAGGGATCGCATTGATC	MSTRG.19711.2	1	2028-2047	2038	2	42	21
1471	osa-miR394	TTGGCATTCTGTCCACCTCC	LOC_Os01g69940.1	0	1241-1260	1251	0	14	14
1472	osa-MIR394-p3	AGGTGGGCATACTGCCAATGG	MSTRG.12857.9	4	914-933	924	4	1	0.1

1473	osa-MIR394-p3	AGGTGGGCATACTGCCAATGG	MSTRG.12857.8	4	920-939	930	4	1	0.1
1474	osa-MIR394-p3	AGGTGGGCATACTGCCAATGG	MSTRG.12857.11	4	917-936	927	4	1	0.1
1475	osa-MIR394-p3	AGGTGGGCATACTGCCAATGG	LOC_Os02g57270.2	4	947-966	957	4	1	0.1
1476	osa-MIR394-p3	AGGTGGGCATACTGCCAATGG	LOC_Os02g57270.1	4	949-968	959	4	1	0.1
1477	osa-MIR394-p3	AGGTGGGCATACTGCCAATGG	MSTRG.12857.5	4	927-946	937	4	1	0.1
1478	osa-MIR394-p3	AGGTGGGCATACTGCCAATGG	MSTRG.12857.3	4	971-990	981	4	1	0.1
1479	osa-MIR394-p3	AGGTGGGCATACTGCCAATGG	MSTRG.12857.2	4	931-950	941	4	1	0.1
1480	osa-MIR394-p3	AGGTGGGCATACTGCCAATGG	MSTRG.12857.1	4	987-1006	997	4	1	0.1
1481	osa-MIR394-p3	AGGTGGGCATACTGCCAATGG	MSTRG.12857.4	4	911-930	921	4	1	0.1
1482	osa-MIR394-p3	AGGTGGGCATACTGCCAATGG	LOC_Os07g15430.1	3.5	1331-1350	1341	2	2	2
1483	osa-miR395b_L-1	TGAAGTGTTTGGGGGAACTC	LOC_Os07g31370.1	4	606-626	617	3	4	0.4
1484	osa-miR395b_L-1	TGAAGTGTTTGGGGGAACTC	LOC_Os10g35870.1	4	651-671	662	2	22	4.4
1485	osa-miR395b_L-1	TGAAGTGTTTGGGGGAACTC	MSTRG.5222.4	4	590-610	601	2	22	4.4
1486	osa-miR395b_L-1	TGAAGTGTTTGGGGGAACTC	MSTRG.5222.3	4	590-610	601	2	22	4.4
1487	osa-miR395b_L-1	TGAAGTGTTTGGGGGAACTC	MSTRG.5222.2	4	669-689	680	2	22	4.4
1488	osa-miR395b_L-1	TGAAGTGTTTGGGGGAACTC	MSTRG.5222.1	4	669-689	680	2	22	4.4
1489	osa-miR395b_L-1	TGAAGTGTTTGGGGGAACTC	LOC_Os03g53230.1	2	595-614	605	2	2	2
1490	osa-miR396a-3p_R+1	GTTCAATAAAGCTGTGGGAAA	LOC_Os02g13270.1	4	794-814	805	4	1	0.5
1491	osa-miR396a-3p_R+1	GTTCAATAAAGCTGTGGGAAA	MSTRG.10474.1	4	961-981	972	4	1	0.5
1492	osa-miR396a-5p	TTCCACAGCTTTCTTGAACTG	LOC_Os02g45570.1	3	629-650	640	0	120	40
1493	osa-miR396a-5p	TTCCACAGCTTTCTTGAACTG	LOC_Os02g45570.2	3	838-859	849	0	120	40
1494	osa-miR396a-5p	TTCCACAGCTTTCTTGAACTG	MSTRG.12043.2	3	879-900	890	0	120	40
1495	osa-miR396a-5p	TTCCACAGCTTTCTTGAACTG	LOC_Os02g47280.2	3	570-591	581	0	12	6
1496	osa-miR396a-5p	TTCCACAGCTTTCTTGAACTG	LOC_Os02g47280.1	3	667-688	678	0	12	6
1497	osa-miR396a-5p	TTCCACAGCTTTCTTGAACTG	LOC_Os02g53690.1	3	570-591	581	0	50	25
1498	osa-miR396a-5p	TTCCACAGCTTTCTTGAACTG	MSTRG.12592.2	3	670-691	681	0	50	25
1499	osa-miR396a-5p	TTCCACAGCTTTCTTGAACTG	LOC_Os04g51190.3	3	535-556	546	0	6	1
1500	osa-miR396a-5p	TTCCACAGCTTTCTTGAACTG	LOC_Os04g51190.1	3	535-556	546	0	6	1
1501	osa-miR396a-5p	TTCCACAGCTTTCTTGAACTG	LOC_Os04g51190.2	3	535-556	546	0	6	1
1502	osa-miR396a-5p	TTCCACAGCTTTCTTGAACTG	MSTRG.18786.2	3	720-741	731	0	6	1
1503	osa-miR396a-5p	TTCCACAGCTTTCTTGAACTG	MSTRG.18786.4	3	1504-1525	1515	0	6	1
1504	osa-miR396a-5p	TTCCACAGCTTTCTTGAACTG	MSTRG.18786.1	3	1541-1562	1552	0	6	1
1505	osa-miR396a-5p	TTCCACAGCTTTCTTGAACTG	MSTRG.21999.5	3	469-490	480	0	26	5.2
1506	osa-miR396a-5p	TTCCACAGCTTTCTTGAACTG	LOC_Os06g02560.2	3	605-626	616	0	26	5.2
1507	osa-miR396a-5p	TTCCACAGCTTTCTTGAACTG	MSTRG.21999.2	3	509-530	520	0	26	5.2
1508	osa-miR396a-5p	TTCCACAGCTTTCTTGAACTG	LOC_Os06g02560.3	3	625-646	636	0	26	5.2
1509	osa-miR396a-5p	TTCCACAGCTTTCTTGAACTG	LOC_Os06g02560.1	3	605-626	616	0	26	5.2
1510	osa-miR396a-5p	TTCCACAGCTTTCTTGAACTG	LOC_Os11g35030.1	3.5	869-890	880	0	32	16
1511	osa-miR396a-5p	TTCCACAGCTTTCTTGAACTG	LOC_Os11g35030.2	3.5	1556-1577	1567	0	32	16
1512	osa-miR396a-5p	TTCCACAGCTTTCTTGAACTG	LOC_Os12g29980.2	3	732-753	743	2	4	2
1513	osa-miR396a-5p	TTCCACAGCTTTCTTGAACTG	LOC_Os12g29980.1	3	774-795	785	2	4	2
1514	osa-miR396a-5p	TTCCACAGCTTTCTTGAACTG	MSTRG.533.4	4	1997-2017	2008	2	2	0.5
1515	osa-miR396a-5p	TTCCACAGCTTTCTTGAACTG	LOC_Os01g08560.2	4	1991-2011	2002	2	2	0.5
1516	osa-miR396a-5p	TTCCACAGCTTTCTTGAACTG	LOC_Os01g08560.1	4	1995-2015	2006	2	2	0.5
1517	osa-miR396a-5p	TTCCACAGCTTTCTTGAACTG	MSTRG.533.3	4	2421-2441	2432	2	2	0.5
1518	osa-miR396a-5p	TTCCACAGCTTTCTTGAACTG	LOC_Os03g51970.1	3	419-440	430	0	6	3
1519	osa-miR396a-5p	TTCCACAGCTTTCTTGAACTG	LOC_Os03g47140.1	3	947-968	958	0	20	5
1520	osa-miR396a-5p	TTCCACAGCTTTCTTGAACTG	MSTRG.15768.1	3	696-717	707	0	6	3
1521	osa-miR396a-5p	TTCCACAGCTTTCTTGAACTG	MSTRG.15456.4	3	1382-1403	1393	0	20	5
1522	osa-miR396a-5p	TTCCACAGCTTTCTTGAACTG	MSTRG.15456.3	3	1359-1380	1370	0	20	5
1523	osa-miR396a-5p	TTCCACAGCTTTCTTGAACTG	MSTRG.15456.2	3	1386-1407	1397	0	20	5
1524	osa-miR396a-5p	TTCCACAGCTTTCTTGAACTG	LOC_Os06g10310.1	3	423-444	434	0	3	3
1525	osa-miR396c-5p	TTCCACAGCTTTCTTGAACTT	LOC_Os02g45570.1	4	629-650	640	0	120	40
1526	osa-miR396c-5p	TTCCACAGCTTTCTTGAACTT	LOC_Os02g45570.2	4	838-859	849	0	120	40
1527	osa-miR396c-5p	TTCCACAGCTTTCTTGAACTT	MSTRG.12043.2	4	879-900	890	0	120	40
1528	osa-miR396c-5p	TTCCACAGCTTTCTTGAACTT	LOC_Os02g47280.2	4	570-591	581	0	12	6
1529	osa-miR396c-5p	TTCCACAGCTTTCTTGAACTT	LOC_Os02g47280.1	4	667-688	678	0	12	6
1530	osa-miR396c-5p	TTCCACAGCTTTCTTGAACTT	LOC_Os02g53690.1	4	570-591	581	0	50	25
1531	osa-miR396c-5p	TTCCACAGCTTTCTTGAACTT	MSTRG.12592.2	4	670-691	681	0	50	25

1532	osa-miR396c-5p	TTCCACAGCTTTCTTGAACTT	LOC_Os04g51190.3	4	535-556	546	0	6	1
1533	osa-miR396c-5p	TTCCACAGCTTTCTTGAACTT	LOC_Os04g51190.1	4	535-556	546	0	6	1
1534	osa-miR396c-5p	TTCCACAGCTTTCTTGAACTT	LOC_Os04g51190.2	4	535-556	546	0	6	1
1535	osa-miR396c-5p	TTCCACAGCTTTCTTGAACTT	MSTRG.18786.2	4	720-741	731	0	6	1
1536	osa-miR396c-5p	TTCCACAGCTTTCTTGAACTT	MSTRG.18786.4	4	1504-1525	1515	0	6	1
1537	osa-miR396c-5p	TTCCACAGCTTTCTTGAACTT	MSTRG.18786.1	4	1541-1562	1552	0	6	1
1538	osa-miR396c-5p	TTCCACAGCTTTCTTGAACTT	MSTRG.21999.5	4	469-490	480	0	26	5.2
1539	osa-miR396c-5p	TTCCACAGCTTTCTTGAACTT	LOC_Os06g02560.2	4	605-626	616	0	26	5.2
1540	osa-miR396c-5p	TTCCACAGCTTTCTTGAACTT	MSTRG.21999.2	4	509-530	520	0	26	5.2
1541	osa-miR396c-5p	TTCCACAGCTTTCTTGAACTT	LOC_Os06g02560.1	4	605-626	616	0	26	5.2
1542	osa-miR396c-5p	TTCCACAGCTTTCTTGAACTT	LOC_Os06g02560.3	4	625-646	636	0	26	5.2
1543	osa-miR396c-5p	TTCCACAGCTTTCTTGAACTT	LOC_Os11g35030.1	4	869-890	880	0	32	16
1544	osa-miR396c-5p	TTCCACAGCTTTCTTGAACTT	LOC_Os11g35030.2	4	1556-1577	1567	0	32	16
1545	osa-miR396c-5p	TTCCACAGCTTTCTTGAACTT	LOC_Os12g29980.2	4	732-753	743	2	4	2
1546	osa-miR396c-5p	TTCCACAGCTTTCTTGAACTT	LOC_Os12g29980.1	4	774-795	785	2	4	2
1547	osa-miR396c-5p	TTCCACAGCTTTCTTGAACTT	MSTRG.533.4	3.5	1997-2017	2008	2	2	0.5
1548	osa-miR396c-5p	TTCCACAGCTTTCTTGAACTT	LOC_Os01g08560.2	3.5	1991-2011	2002	2	2	0.5
1549	osa-miR396c-5p	TTCCACAGCTTTCTTGAACTT	LOC_Os01g08560.1	3.5	1995-2015	2006	2	2	0.5
1550	osa-miR396c-5p	TTCCACAGCTTTCTTGAACTT	MSTRG.533.3	3.5	2421-2441	2432	2	2	0.5
1551	osa-miR396c-5p	TTCCACAGCTTTCTTGAACTT	LOC_Os03g51970.1	4	419-440	430	0	6	3
1552	osa-miR396c-5p	TTCCACAGCTTTCTTGAACTT	LOC_Os03g47140.1	4	947-968	958	0	20	5
1553	osa-miR396c-5p	TTCCACAGCTTTCTTGAACTT	MSTRG.15768.1	4	696-717	707	0	6	3
1554	osa-miR396c-5p	TTCCACAGCTTTCTTGAACTT	MSTRG.15456.4	4	1382-1403	1393	0	20	5
1555	osa-miR396c-5p	TTCCACAGCTTTCTTGAACTT	MSTRG.15456.3	4	1359-1380	1370	0	20	5
1556	osa-miR396c-5p	TTCCACAGCTTTCTTGAACTT	MSTRG.15456.2	4	1386-1407	1397	0	20	5
1557	osa-miR396c-5p	TTCCACAGCTTTCTTGAACTT	LOC_Os06g10310.1	4	423-444	434	0	3	3
1558	osa-miR396d	TCCACAGGCTTTCTTGAACGG	LOC_Os02g45570.1	0	629-649	640	0	120	40
1559	osa-miR396d	TCCACAGGCTTTCTTGAACGG	LOC_Os02g45570.2	0	838-858	849	0	120	40
1560	osa-miR396d	TCCACAGGCTTTCTTGAACGG	MSTRG.12043.2	0	879-899	890	0	120	40
1561	osa-miR396d	TCCACAGGCTTTCTTGAACGG	LOC_Os02g47280.2	0	570-590	581	0	12	6
1562	osa-miR396d	TCCACAGGCTTTCTTGAACGG	LOC_Os02g47280.1	0	667-687	678	0	12	6
1563	osa-miR396d	TCCACAGGCTTTCTTGAACGG	LOC_Os02g53690.1	0	570-590	581	0	50	25
1564	osa-miR396d	TCCACAGGCTTTCTTGAACGG	MSTRG.12592.2	0	670-690	681	0	50	25
1565	osa-miR396d	TCCACAGGCTTTCTTGAACGG	LOC_Os04g51190.3	0	535-555	546	0	6	1
1566	osa-miR396d	TCCACAGGCTTTCTTGAACGG	LOC_Os04g51190.1	0	535-555	546	0	6	1
1567	osa-miR396d	TCCACAGGCTTTCTTGAACGG	LOC_Os04g51190.2	0	535-555	546	0	6	1
1568	osa-miR396d	TCCACAGGCTTTCTTGAACGG	MSTRG.18786.2	0	720-740	731	0	6	1
1569	osa-miR396d	TCCACAGGCTTTCTTGAACGG	MSTRG.18786.4	0	1504-1524	1515	0	6	1
1570	osa-miR396d	TCCACAGGCTTTCTTGAACGG	MSTRG.18786.1	0	1541-1561	1552	0	6	1
1571	osa-miR396d	TCCACAGGCTTTCTTGAACGG	MSTRG.21999.5	0	469-489	480	0	26	5.2
1572	osa-miR396d	TCCACAGGCTTTCTTGAACGG	LOC_Os06g02560.2	0	605-625	616	0	26	5.2
1573	osa-miR396d	TCCACAGGCTTTCTTGAACGG	MSTRG.21999.2	0	509-529	520	0	26	5.2
1574	osa-miR396d	TCCACAGGCTTTCTTGAACGG	LOC_Os06g02560.1	0	605-625	616	0	26	5.2
1575	osa-miR396d	TCCACAGGCTTTCTTGAACGG	LOC_Os06g02560.3	0	625-645	636	0	26	5.2
1576	osa-miR396d	TCCACAGGCTTTCTTGAACGG	LOC_Os11g35030.1	2.5	869-889	880	0	32	16
1577	osa-miR396d	TCCACAGGCTTTCTTGAACGG	LOC_Os11g35030.2	2.5	1556-1576	1567	0	32	16
1578	osa-miR396d	TCCACAGGCTTTCTTGAACGG	LOC_Os12g29980.2	2	732-752	743	2	4	2
1579	osa-miR396d	TCCACAGGCTTTCTTGAACGG	LOC_Os12g29980.1	2	774-794	785	2	4	2
1580	osa-miR396d	TCCACAGGCTTTCTTGAACGG	LOC_Os03g47140.1	0	947-967	958	0	20	5
1581	osa-miR396d	TCCACAGGCTTTCTTGAACGG	MSTRG.15456.4	0	1382-1402	1393	0	20	5
1582	osa-miR396d	TCCACAGGCTTTCTTGAACGG	MSTRG.15456.3	0	1359-1379	1370	0	20	5
1583	osa-miR396d	TCCACAGGCTTTCTTGAACGG	MSTRG.15456.2	0	1386-1406	1397	0	20	5
1584	osa-miR396d	TCCACAGGCTTTCTTGAACGG	LOC_Os03g51970.1	2	419-439	430	0	6	3
1585	osa-miR396d	TCCACAGGCTTTCTTGAACGG	MSTRG.15768.1	2	696-716	707	0	6	3
1586	osa-miR396d	TCCACAGGCTTTCTTGAACGG	LOC_Os06g10310.1	0	423-443	434	0	3	3
1587	osa-miR396e-5p	TCCACAGGCTTTCTTGAACTG	LOC_Os02g45570.1	1	629-649	640	0	120	40
1588	osa-miR396e-5p	TCCACAGGCTTTCTTGAACTG	LOC_Os02g45570.2	1	838-858	849	0	120	40
1589	osa-miR396e-5p	TCCACAGGCTTTCTTGAACTG	MSTRG.12043.2	1	879-899	890	0	120	40
1590	osa-miR396e-5p	TCCACAGGCTTTCTTGAACTG	LOC_Os02g47280.2	1	570-590	581	0	12	6

1591	osa-miR396e-5p	TCCACAGGCTTTCTTGAACTG	LOC_Os02g47280.1	1	667-687	678	0	12	6
1592	osa-miR396e-5p	TCCACAGGCTTTCTTGAACTG	LOC_Os02g53690.1	1	570-590	581	0	50	25
1593	osa-miR396e-5p	TCCACAGGCTTTCTTGAACTG	MSTRG.12592.2	1	670-690	681	0	50	25
1594	osa-miR396e-5p	TCCACAGGCTTTCTTGAACTG	LOC_Os04g51190.3	1	535-555	546	0	6	1
1595	osa-miR396e-5p	TCCACAGGCTTTCTTGAACTG	LOC_Os04g51190.1	1	535-555	546	0	6	1
1596	osa-miR396e-5p	TCCACAGGCTTTCTTGAACTG	LOC_Os04g51190.2	1	535-555	546	0	6	1
1597	osa-miR396e-5p	TCCACAGGCTTTCTTGAACTG	MSTRG.18786.2	1	720-740	731	0	6	1
1598	osa-miR396e-5p	TCCACAGGCTTTCTTGAACTG	MSTRG.18786.4	1	1504-1524	1515	0	6	1
1599	osa-miR396e-5p	TCCACAGGCTTTCTTGAACTG	MSTRG.18786.1	1	1541-1561	1552	0	6	1
1600	osa-miR396e-5p	TCCACAGGCTTTCTTGAACTG	MSTRG.21999.5	1	469-489	480	0	26	5.2
1601	osa-miR396e-5p	TCCACAGGCTTTCTTGAACTG	LOC_Os06g02560.2	1	605-625	616	0	26	5.2
1602	osa-miR396e-5p	TCCACAGGCTTTCTTGAACTG	MSTRG.21999.2	1	509-529	520	0	26	5.2
1603	osa-miR396e-5p	TCCACAGGCTTTCTTGAACTG	LOC_Os06g02560.1	1	605-625	616	0	26	5.2
1604	osa-miR396e-5p	TCCACAGGCTTTCTTGAACTG	LOC_Os06g02560.3	1	625-645	636	0	26	5.2
1605	osa-miR396e-5p	TCCACAGGCTTTCTTGAACTG	LOC_Os11g35030.1	3.5	869-889	880	0	32	16
1606	osa-miR396e-5p	TCCACAGGCTTTCTTGAACTG	LOC_Os11g35030.2	3.5	1556-1576	1567	0	32	16
1607	osa-miR396e-5p	TCCACAGGCTTTCTTGAACTG	LOC_Os12g29980.2	3	732-752	743	2	4	2
1608	osa-miR396e-5p	TCCACAGGCTTTCTTGAACTG	LOC_Os12g29980.1	3	774-794	785	2	4	2
1609	osa-miR396e-5p	TCCACAGGCTTTCTTGAACTG	LOC_Os03g47140.1	1	947-967	958	0	20	5
1610	osa-miR396e-5p	TCCACAGGCTTTCTTGAACTG	MSTRG.15456.4	1	1382-1402	1393	0	20	5
1611	osa-miR396e-5p	TCCACAGGCTTTCTTGAACTG	MSTRG.15456.3	1	1359-1379	1370	0	20	5
1612	osa-miR396e-5p	TCCACAGGCTTTCTTGAACTG	MSTRG.15456.2	1	1386-1406	1397	0	20	5
1613	osa-miR396e-5p	TCCACAGGCTTTCTTGAACTG	LOC_Os03g51970.1	3	419-439	430	0	6	3
1614	osa-miR396e-5p	TCCACAGGCTTTCTTGAACTG	MSTRG.15768.1	3	696-716	707	0	6	3
1615	osa-miR396e-5p	TCCACAGGCTTTCTTGAACTG	LOC_Os06g10310.1	1	423-443	434	0	3	3
1616	osa-miR396f-3p_L-3	GTTCAAGAAAGTCCTTGGAAA	LOC_Os06g39480.1	3.5	2206-2225	2216	2	8	4
1617	osa-miR396f-3p_L-3	GTTCAAGAAAGTCCTTGGAAA	MSTRG.23643.1	3.5	2236-2255	2246	2	8	4
1618	osa-miR3979-3p	CTTCGGGGGAGGAGAGAAGC	LOC_Os07g08530.1	3	1157-1177	1168	4	1	1
1619	osa-miR3979-5p_R+1	TCTCTCTCTCCCTTGAAGGCT	LOC_Os09g32150.1	3.5	435-454	446	3	2	2
1620	osa-miR3979-5p_R+1	TCTCTCTCTCCCTTGAAGGCT	LOC_Os07g04330.1	4	28-47	38	4	1	1
1621	osa-miR3979-5p_R+1	TCTCTCTCTCCCTTGAAGGCT	LOC_Os12g14070.1	3	1560-1579	1570	4	1	1
1622	osa-miR3980a-3p	CTGGCCGAGGCCGTCGATTCT	LOC_Os02g03750.1	3.5	1147-1167	1158	2	2	0.666666667
1623	osa-miR3980a-3p	CTGGCCGAGGCCGTCGATTCT	MSTRG.9805.2	3.5	1308-1328	1319	2	2	0.666666667
1624	osa-miR3980a-3p	CTGGCCGAGGCCGTCGATTCT	MSTRG.9805.3	3.5	3720-3740	3731	2	2	0.666666667
1625	osa-miR398b	TGTGTTCTCAGGTCGCCCCTG	LOC_Os07g46990.1	2.5	124-144	135	2	19	9.5
1626	osa-miR398b	TGTGTTCTCAGGTCGCCCCTG	MSTRG.26623.1	2.5	162-182	173	2	19	9.5
1627	osa-miR399a	TGCCAAAGGAGAATTGCCCTG	LOC_Os07g40620.1	4	1036-1057	1048	4	1	1
1628	osa-miR408-3p	CTGCACTGCCTCTTCCCTGGC	LOC_Os03g15340.1	1	85-105	96	2	33	33
1629	osa-miR408-3p	CTGCACTGCCTCTTCCCTGGC	LOC_Os08g37670.1	3	657-677	668	0	403	403
1630	osa-miR408-3p	CTGCACTGCCTCTTCCCTGGC	LOC_Os03g50140.1	3	252-272	263	2	2	2
1631	osa-MIR435-p3	CGGTATTGGAGTTGAGGGATG	LOC_Os09g31180.2	4	423-442	433	3	2	0.666666667
1632	osa-MIR435-p3	CGGTATTGGAGTTGAGGGATG	LOC_Os09g31180.1	4	423-442	433	3	2	0.666666667
1633	osa-MIR435-p3	CGGTATTGGAGTTGAGGGATG	LOC_Os02g01332.1	4	402-421	412	3	2	0.666666667
1634	osa-miR444a-3p.2_R+2	TGCAGTTGCTGCCTCAAGCTTCT	LOC_Os08g06510.1	3	1369-1391	1382	2	14	14
1635	osa-MIR5079a-p5_1ss18AT	TACCCATCTAAATGATTTCAT	MSTRG.13482.2	3	174-194	185	4	1	0.5
1636	osa-MIR5079a-p5_1ss18AT	TACCCATCTAAATGATTTCAT	LOC_Os03g08000.1	3	537-557	548	4	1	0.5
1637	osa-miR5144-3p_L-1R+1	CTCCTCAGCAGCACAAGAAGA	MSTRG.22607.1	4	998-1018	1009	4	1	0.5
1638	osa-miR5144-3p_L-1R+1	CTCCTCAGCAGCACAAGAAGA	LOC_Os06g12100.1	4	998-1018	1009	4	1	0.5
1639	osa-miR5150-3p_L-1R+1	GAAGCTGCAGCTGTCAGAAGCTCC	LOC_Os05g51500.1	3.5	459-481	472	2	2	1
1640	osa-miR5150-3p_L-1R+1	GAAGCTGCAGCTGTCAGAAGCTCC	MSTRG.21861.2	3.5	757-779	770	2	2	1
1641	osa-miR528-3p	CCTGTGCTTGCCTCTTCCATT	LOC_Os05g04220.1	4	527-547	538	4	1	0.333333333
1642	osa-miR528-3p	CCTGTGCTTGCCTCTTCCATT	LOC_Os05g04220.2	4	620-640	631	4	1	0.333333333
1643	osa-miR528-3p	CCTGTGCTTGCCTCTTCCATT	MSTRG.19621.1	4	656-676	667	4	1	0.333333333
1644	osa-miR528-5p	TGGAAGGGGCATGCAGAGGAG	LOC_Os03g03724.2	4	146-166	157	2	2	1
1645	osa-miR528-5p	TGGAAGGGGCATGCAGAGGAG	LOC_Os03g03724.1	4	146-166	157	2	2	1
1646	osa-miR528-5p	TGGAAGGGGCATGCAGAGGAG	LOC_Os06g06050.1	3.5	2648-2668	2659	2	9	1.8
1647	osa-miR528-5p	TGGAAGGGGCATGCAGAGGAG	MSTRG.22228.1	3.5	3217-3237	3228	2	9	1.8
1648	osa-miR528-5p	TGGAAGGGGCATGCAGAGGAG	MSTRG.22228.4	3.5	3147-3167	3158	2	9	1.8
1649	osa-miR528-5p	TGGAAGGGGCATGCAGAGGAG	MSTRG.22228.3	3.5	3213-3233	3224	2	9	1.8

1650	osa-miR528-5p	TGGAAGGGGCATGCAGAGGAG	MSTRG.22228.2	3.5	3285-3305	3296	2	9	1.8
1651	osa-miR528-5p	TGGAAGGGGCATGCAGAGGAG	MSTRG.26074.2	2	651-670	661	0	5187	1729
1652	osa-miR528-5p	TGGAAGGGGCATGCAGAGGAG	LOC_Os07g38290.1	2	528-547	538	0	5187	1729
1653	osa-miR528-5p	TGGAAGGGGCATGCAGAGGAG	MSTRG.26074.3	2	2460-2479	2470	0	5187	1729
1654	osa-miR528-5p	TGGAAGGGGCATGCAGAGGAG	LOC_Os09g20090.1	4	229-248	239	2	3	3
1655	osa-miR528-5p	TGGAAGGGGCATGCAGAGGAG	MSTRG.9083.3	4	2837-2856	2847	4	1	0.5
1656	osa-miR528-5p	TGGAAGGGGCATGCAGAGGAG	MSTRG.9083.2	4	2787-2806	2797	4	1	0.5
1657	osa-miR528-5p	TGGAAGGGGCATGCAGAGGAG	LOC_Os03g50160.1	4	173-192	184	3	2	1
1658	osa-miR528-5p	TGGAAGGGGCATGCAGAGGAG	MSTRG.15647.1	4	228-247	239	3	2	1
1659	osa-miR529a_L+1	GCTGTACCCTCTCTCTTCTTC	LOC_Os03g11890.1	4	2774-2794	2785	4	1	0.5
1660	osa-miR529a_L+1	GCTGTACCCTCTCTCTTCTTC	MSTRG.13785.1	4	2774-2794	2785	4	1	0.5
1661	osa-MIR529a-p5	AGAAGAGAGAGAGTACAGCCT	LOC_Os09g31438.1	2.5	804-824	815	4	1	1
1662	osa-MIR529a-p5	AGAAGAGAGAGAGTACAGCCT	LOC_Os06g49010.2	2.5	1490-1509	1500	4	1	0.1
1663	osa-MIR529a-p5	AGAAGAGAGAGAGTACAGCCT	MSTRG.24320.18	2.5	1935-1954	1945	4	1	0.1
1664	osa-MIR529a-p5	AGAAGAGAGAGAGTACAGCCT	MSTRG.24320.21	2.5	2021-2040	2031	4	1	0.1
1665	osa-MIR529a-p5	AGAAGAGAGAGAGTACAGCCT	MSTRG.24320.23	2.5	2311-2330	2321	4	1	0.1
1666	osa-MIR529a-p5	AGAAGAGAGAGAGTACAGCCT	MSTRG.24320.16	2.5	2275-2294	2285	4	1	0.1
1667	osa-MIR529a-p5	AGAAGAGAGAGAGTACAGCCT	MSTRG.24320.19	2.5	2409-2428	2419	4	1	0.1
1668	osa-MIR529a-p5	AGAAGAGAGAGAGTACAGCCT	MSTRG.24320.20	2.5	2680-2699	2690	4	1	0.1
1669	osa-MIR529a-p5	AGAAGAGAGAGAGTACAGCCT	MSTRG.24320.24	2.5	2739-2758	2749	4	1	0.1
1670	osa-MIR529a-p5	AGAAGAGAGAGAGTACAGCCT	MSTRG.24320.17	2.5	2669-2688	2679	4	1	0.1
1671	osa-MIR529a-p5	AGAAGAGAGAGAGTACAGCCT	MSTRG.24320.22	2.5	2777-2796	2787	4	1	0.1
1672	osa-miR530-5p_R+1	TGCATTTGCACCTGCACCTAC	LOC_Os01g56780.1	3	429-449	440	4	1	0.5
1673	osa-miR530-5p_R+1	TGCATTTGCACCTGCACCTAC	MSTRG.25982.2	3	966-987	978	4	1	0.25
1674	osa-miR530-5p_R+1	TGCATTTGCACCTGCACCTAC	MSTRG.25982.1	3	1047-1068	1059	4	1	0.25
1675	osa-miR530-5p_R+1	TGCATTTGCACCTGCACCTAC	LOC_Os07g36820.1	3	1089-1110	1101	4	1	0.25
1676	osa-miR530-5p_R+1	TGCATTTGCACCTGCACCTAC	LOC_Os07g36820.2	3	2330-2351	2342	4	1	0.25
1677	osa-miR531a_R-2	CTCGCCGGGGCTGCGTGCCGCC	LOC_Os04g45370.1	3.5	393-413	404	2	16	16
1678	osa-miR535-5p	TGACAACGAGAGAGAGCACGC	LOC_Os06g49010.4	4	1636-1657	1647	4	1	0.1
1679	osa-miR535-5p	TGACAACGAGAGAGAGCACGC	LOC_Os06g49010.6	4	1840-1861	1851	4	1	0.1
1680	osa-miR535-5p	TGACAACGAGAGAGAGCACGC	MSTRG.24320.23	4	2313-2334	2324	4	1	0.1
1681	osa-miR535-5p	TGACAACGAGAGAGAGCACGC	MSTRG.24320.16	4	2277-2298	2288	4	1	0.1
1682	osa-miR535-5p	TGACAACGAGAGAGAGCACGC	MSTRG.24320.19	4	2411-2432	2422	4	1	0.1
1683	osa-miR535-5p	TGACAACGAGAGAGAGCACGC	MSTRG.24320.5	4	2358-2379	2369	4	1	0.1
1684	osa-miR535-5p	TGACAACGAGAGAGAGCACGC	MSTRG.24320.20	4	2682-2703	2693	4	1	0.1
1685	osa-miR535-5p	TGACAACGAGAGAGAGCACGC	MSTRG.24320.17	4	2671-2692	2682	4	1	0.1
1686	osa-miR535-5p	TGACAACGAGAGAGAGCACGC	MSTRG.24320.15	4	2762-2783	2773	4	1	0.1
1687	osa-miR535-5p	TGACAACGAGAGAGAGCACGC	MSTRG.24320.2	4	2766-2787	2777	4	1	0.1
1688	osa-miR5487	AAAGATGTGCATGTAGTTCCG	MSTRG.7143.4	4	493-513	504	2	3	0.6
1689	osa-miR5487	AAAGATGTGCATGTAGTTCCG	LOC_Os11g38810.2	4	957-977	968	2	3	0.6
1690	osa-miR5487	AAAGATGTGCATGTAGTTCCG	LOC_Os11g38810.1	4	962-982	973	2	3	0.6
1691	osa-miR5487	AAAGATGTGCATGTAGTTCCG	MSTRG.7143.5	4	1093-1113	1104	2	3	0.6
1692	osa-miR5487	AAAGATGTGCATGTAGTTCCG	MSTRG.7143.1	4	2171-2191	2182	2	3	0.6
1693	osa-miR5488	TGAAGGCGACTGATGATTTCA	MSTRG.24813.2	4	2462-2481	2472	2	2	1
1694	osa-miR5488	TGAAGGCGACTGATGATTTCA	LOC_Os07g07080.1	4	2544-2563	2554	2	2	1
1695	osa-MIR5489-p3	TGAGCTAGAGAGATCAAAGAG	LOC_Os10g35550.1	3.5	2560-2581	2572	4	1	1
1696	osa-miR5490	TTGGATTTTTATTTAGGACGG	LOC_Os04g48230.2	3.5	1459-1478	1469	2	2	1
1697	osa-miR5490	TTGGATTTTTATTTAGGACGG	LOC_Os04g48230.1	3.5	1459-1478	1469	2	2	1
1698	osa-MIR5494-p5	CCACGTCACTTTCTTTTTGCC	LOC_Os04g21320.3	3.5	529-549	540	3	3	1.5
1699	osa-MIR5494-p5	CCACGTCACTTTCTTTTTGCC	LOC_Os04g21320.1	3.5	568-588	579	3	3	1.5
1700	osa-MIR5495-p3	CCATGGAGATCATTGTGACCT	LOC_Os04g57200.1	4	308-328	319	2	2	1
1701	osa-MIR5495-p3	CCATGGAGATCATTGTGACCT	LOC_Os04g57200.2	4	611-631	622	2	2	1
1702	osa-MIR5495-p3	CCATGGAGATCATTGTGACCT	MSTRG.6084.4	4	1877-1897	1888	2	10	2.5
1703	osa-MIR5495-p3	CCATGGAGATCATTGTGACCT	LOC_Os11g07440.3	4	1918-1938	1929	2	10	2.5
1704	osa-MIR5495-p3	CCATGGAGATCATTGTGACCT	MSTRG.6084.1	4	1978-1998	1989	2	10	2.5
1705	osa-MIR5495-p3	CCATGGAGATCATTGTGACCT	LOC_Os11g07440.1	4	1995-2015	2006	2	10	2.5
1706	osa-miR5497	CAGAATATCTGGGACGAGCAT	LOC_Os03g16824.1	3.5	761-780	771	4	1	1
1707	osa-MIR5497-p3_1ss20CT	ATGTTGGCTCTCCCAATGCTT	MSTRG.23419.4	4	2589-2610	2601	2	2	0.4
1708	osa-MIR5497-p3_1ss20CT	ATGTTGGCTCTCCCAATGCTT	MSTRG.23419.5	4	2847-2868	2859	2	2	0.4

1709	osa-MIR5497-p3_1ss20CT	ATGTTGGCTCTCCCAATGCTT	LOC_Os06g34440.1	4	2966-2987	2978	2	2	0.4
1710	osa-MIR5497-p3_1ss20CT	ATGTTGGCTCTCCCAATGCTT	MSTRG.23419.3	4	2933-2954	2945	2	2	0.4
1711	osa-MIR5497-p3_1ss20CT	ATGTTGGCTCTCCCAATGCTT	MSTRG.23419.1	4	2966-2987	2978	2	2	0.4
1712	osa-miR5504_R-3	AGTGACGGGAGGACTGCA	LOC_Os08g44290.1	4	1105-1121	1112	2	3	3
1713	osa-miR5504_R-3	AGTGACGGGAGGACTGCA	LOC_Os06g40550.1	4	1970-1988	1978	2	11	3.666666667
1714	osa-miR5504_R-3	AGTGACGGGAGGACTGCA	MSTRG.23706.1	4	2108-2126	2116	2	11	3.666666667
1715	osa-miR5504_R-3	AGTGACGGGAGGACTGCA	MSTRG.23706.3	4	2364-2382	2372	2	11	3.666666667
1716	osa-miR5509	TAGGCATTTTCTCTTGGCATG	LOC_Os05g04940.1	2.5	636-656	647	2	5	2.5
1717	osa-miR5509	TAGGCATTTTCTCTTGGCATG	MSTRG.19661.2	2.5	714-734	725	2	5	2.5
1718	osa-miR5509	TAGGCATTTTCTCTTGGCATG	LOC_Os03g48010.1	4	2184-2204	2195	4	1	0.333333333
1719	osa-miR5509	TAGGCATTTTCTCTTGGCATG	MSTRG.15517.1	4	2184-2204	2195	4	1	0.333333333
1720	osa-miR5509	TAGGCATTTTCTCTTGGCATG	MSTRG.15517.2	4	2184-2204	2195	4	1	0.333333333
1721	osa-MIR5509-p3	CATTTGGGATAGCAGGGATAC	LOC_Os02g05410.1	3.5	3772-3792	3783	4	1	1
1722	osa-miR5513	TAACAAAGGACAACAGACTGA	LOC_Os05g51000.3	4	1314-1333	1324	4	1	0.166666667
1723	osa-miR5513	TAACAAAGGACAACAGACTGA	LOC_Os05g51000.2	4	1347-1366	1357	4	1	0.166666667
1724	osa-miR5513	TAACAAAGGACAACAGACTGA	LOC_Os05g51000.4	4	1395-1414	1405	4	1	0.166666667
1725	osa-miR5513	TAACAAAGGACAACAGACTGA	LOC_Os05g51000.1	4	1447-1466	1457	4	1	0.166666667
1726	osa-miR5513	TAACAAAGGACAACAGACTGA	MSTRG.21840.2	4	1377-1396	1387	4	1	0.166666667
1727	osa-miR5513	TAACAAAGGACAACAGACTGA	MSTRG.21840.1	4	1416-1435	1426	4	1	0.166666667
1728	osa-MIR5521-p5	TGTTCTGCTTCCGTGCCTCTC	LOC_Os05g23700.1	4	947-966	957	4	1	1
1729	osa-MIR5522-p3	TTTCCTCCATGTTCCCATTCC	MSTRG.5757.4	4	3177-3197	3188	2	4	0.5
1730	osa-MIR5522-p3	TTTCCTCCATGTTCCCATTCC	LOC_Os11g02320.2	4	3055-3075	3066	2	4	0.5
1731	osa-MIR5522-p3	TTTCCTCCATGTTCCCATTCC	LOC_Os11g02320.1	4	3065-3085	3076	2	4	0.5
1732	osa-MIR5522-p3	TTTCCTCCATGTTCCCATTCC	LOC_Os11g02320.3	4	3369-3389	3380	2	4	0.5
1733	osa-MIR5522-p3	TTTCCTCCATGTTCCCATTCC	MSTRG.7674.3	4	2978-2998	2989	2	5	0.75
1734	osa-MIR5522-p3	TTTCCTCCATGTTCCCATTCC	LOC_Os12g02260.2	4	3037-3057	3048	2	5	0.75
1735	osa-MIR5522-p3	TTTCCTCCATGTTCCCATTCC	LOC_Os12g02260.1	4	3101-3121	3112	2	5	0.75
1736	osa-MIR5522-p3	TTTCCTCCATGTTCCCATTCC	MSTRG.7674.4	4	3688-3708	3699	2	5	0.75
1737	osa-MIR5788-p3_1ss20AG	AGATTCGTAGTACTAGGATGTGTC	MSTRG.26498.1	4	1113-1136	1127	2	10	1.25
1738	osa-MIR5788-p3_1ss20AG	AGATTCGTAGTACTAGGATGTGTC	LOC_Os07g45350.2	4	1263-1286	1277	2	10	1.25
1739	osa-MIR5788-p3_1ss20AG	AGATTCGTAGTACTAGGATGTGTC	LOC_Os07g45350.4	4	1331-1354	1345	2	10	1.25
1740	osa-MIR5788-p3_1ss20AG	AGATTCGTAGTACTAGGATGTGTC	LOC_Os07g45350.5	4	1354-1377	1368	2	10	1.25
1741	osa-MIR5788-p3_1ss20AG	AGATTCGTAGTACTAGGATGTGTC	LOC_Os07g45350.1	4	1358-1381	1372	2	10	1.25
1742	osa-MIR5788-p3_1ss20AG	AGATTCGTAGTACTAGGATGTGTC	MSTRG.26498.8	4	1445-1468	1459	2	10	1.25
1743	osa-MIR5788-p3_1ss20AG	AGATTCGTAGTACTAGGATGTGTC	MSTRG.26498.2	4	1397-1420	1411	2	10	1.25
1744	osa-MIR5788-p3_1ss20AG	AGATTCGTAGTACTAGGATGTGTC	LOC_Os07g45350.3	4	1761-1784	1775	2	10	1.25
1745	osa-miR5789	TGACTGAGCTTCGTTCGGTAT	LOC_Os08g42980.1	4	664-683	674	2	4	4
1746	osa-miR5797	TCGTGGGATTTATGCAGTTAA	MSTRG.26797.2	3.5	2976-2996	2987	4	1	0.25
1747	osa-miR5797	TCGTGGGATTTATGCAGTTAA	LOC_Os07g49480.1	3.5	3858-3878	3869	4	1	0.25
1748	osa-miR5797	TCGTGGGATTTATGCAGTTAA	MSTRG.26797.4	3.5	3870-3890	3881	4	1	0.25
1749	osa-miR5797	TCGTGGGATTTATGCAGTTAA	LOC_Os07g49480.2	3.5	3858-3878	3869	0	2	2
1750	osa-miR5797	TCGTGGGATTTATGCAGTTAA	MSTRG.26797.1	3.5	3943-3963	3954	4	1	0.25
1751	osa-miR5808_L-2R-1_1ss3TC	CAAATCGTTTCTGATCGTTGG	LOC_Os08g12840.1	4	545-565	556	4	1	1
1752	osa-MIR5808-p5_2ss8TC20TC	TCGTTTCCGATCGTTGGATCT	MSTRG.9243.1	0	74-94	85	4	1	1
1753	osa-miR5818	TCGAACTAGAAGGGCCAGGTT	LOC_Os01g73880.1	3.5	267-287	278	2	5	5
1754	osa-MIR5818-p3	TGTATTTGTGCTTCTCCTGGC	LOC_Os03g54930.1	4	1295-1315	1306	2	2	1
1755	osa-MIR5818-p3	TGTATTTGTGCTTCTCCTGGC	MSTRG.15926.1	4	1465-1485	1476	2	2	1
1756	osa-MIR6251-p3	CTTGCAGCGTGGCTACATGTG	LOC_Os07g03050.1	3.5	1-21	12	4	1	0.5
1757	osa-MIR6251-p3	CTTGCAGCGTGGCTACATGTG	MSTRG.24603.1	3.5	602-622	613	4	1	0.5
1758	osa-MIR6252-p5_1ss10CT	CCCTCCTAATATAACAAATCT	MSTRG.30326.1	2	2283-2303	2294	4	1	1
1759	osa-MIR6253-p3_1ss18AT	ATCATTTTCCTCTCATTT	MSTRG.2544.22	2.5	2301-2318	2309	4	1	0.111111111
1760	osa-MIR6253-p3_1ss18AT	ATCATTTTCCTCTCATTT	MSTRG.2544.21	2.5	3253-3270	3261	4	1	0.111111111
1761	osa-MIR6253-p3_1ss18AT	ATCATTTTCCTCTCATTT	MSTRG.2544.5	2.5	3777-3794	3785	4	1	0.111111111
1762	osa-MIR6253-p3_1ss18AT	ATCATTTTCCTCTCATTT	MSTRG.2544.15	2.5	3350-3367	3358	4	1	0.111111111
1763	osa-MIR6253-p3_1ss18AT	ATCATTTTCCTCTCATTT	MSTRG.2544.6	2.5	3357-3374	3365	4	1	0.111111111
1764	osa-MIR6253-p3_1ss18AT	ATCATTTTCCTCTCATTT	MSTRG.2544.3	2.5	3305-3322	3313	4	1	0.111111111
1765	osa-MIR6253-p3_1ss18AT	ATCATTTTCCTCTCATTT	MSTRG.2544.4	2.5	3385-3402	3393	4	1	0.111111111
1766	osa-MIR6253-p3_1ss18AT	ATCATTTTCCTCTCATTT	MSTRG.2544.13	2.5	3418-3435	3426	4	1	0.111111111
1767	osa-MIR6253-p3_1ss18AT	ATCATTTTCCTCTCATTT	MSTRG.2544.1	2.5	3411-3428	3419	4	1	0.111111111

1768	osa-MIR6253-p3_1ss18AT	ATCATTTTCCTCTCATTT	MSTRG.12892.2	4	888-905	896	2	3	0.75
1769	osa-MIR6253-p3_1ss18AT	ATCATTTTCCTCTCATTT	LOC_Os02g57640.1	4	970-987	978	2	3	0.75
1770	osa-MIR6253-p3_1ss18AT	ATCATTTTCCTCTCATTT	MSTRG.12892.3	4	1228-1245	1236	2	3	0.75
1771	osa-MIR6253-p3_1ss18AT	ATCATTTTCCTCTCATTT	LOC_Os03g18490.1	4	484-501	492	2	2	0.666666667
1772	osa-MIR6253-p3_1ss18AT	ATCATTTTCCTCTCATTT	MSTRG.14245.1	4	770-787	778	2	2	0.666666667
1773	osa-MIR6253-p3_1ss18AT	ATCATTTTCCTCTCATTT	MSTRG.14245.2	4	796-813	804	2	2	0.666666667
1774	osa-MIR6253-p3_1ss18AT	ATCATTTTCCTCTCATTT	LOC_Os04g04890.1	4	736-753	744	4	1	1
1775	osa-MIR6253-p3_1ss18AT	ATCATTTTCCTCTCATTT	LOC_Os04g30780.1	4	360-376	368	2	2	1
1776	osa-MIR6253-p3_1ss18AT	ATCATTTTCCTCTCATTT	LOC_Os04g30780.2	4	360-376	368	2	2	1
1777	osa-MIR6253-p3_1ss18AT	ATCATTTTCCTCTCATTT	LOC_Os05g23620.2	4	941-958	949	2	3	1
1778	osa-MIR6253-p3_1ss18AT	ATCATTTTCCTCTCATTT	LOC_Os05g23620.3	4	959-976	967	2	3	1
1779	osa-MIR6253-p3_1ss18AT	ATCATTTTCCTCTCATTT	LOC_Os05g23620.1	4	986-1003	994	2	3	1
1780	osa-MIR6253-p3_1ss18AT	ATCATTTTCCTCTCATTT	LOC_Os06g07020.1	3.5	1124-1142	1133	4	1	1
1781	osa-MIR6253-p3_1ss18AT	ATCATTTTCCTCTCATTT	LOC_Os07g32340.2	4	2525-2542	2533	4	1	0.333333333
1782	osa-MIR6253-p3_1ss18AT	ATCATTTTCCTCTCATTT	LOC_Os07g32340.1	4	2529-2546	2537	4	1	0.333333333
1783	osa-MIR6253-p3_1ss18AT	ATCATTTTCCTCTCATTT	LOC_Os07g32340.3	4	2568-2585	2576	4	1	0.333333333
1784	osa-MIR6253-p3_1ss18AT	ATCATTTTCCTCTCATTT	LOC_Os07g37570.1	4	250-267	258	4	1	1
1785	osa-MIR6253-p3_1ss18AT	ATCATTTTCCTCTCATTT	LOC_Os08g39090.1	3.5	775-792	783	2	3	0.6
1786	osa-MIR6253-p3_1ss18AT	ATCATTTTCCTCTCATTT	MSTRG.28539.2	3.5	961-978	969	2	3	0.6
1787	osa-MIR6253-p3_1ss18AT	ATCATTTTCCTCTCATTT	MSTRG.28539.4	3.5	1235-1252	1243	2	3	0.6
1788	osa-MIR6253-p3_1ss18AT	ATCATTTTCCTCTCATTT	MSTRG.28539.5	3.5	1376-1393	1384	2	3	0.6
1789	osa-MIR6253-p3_1ss18AT	ATCATTTTCCTCTCATTT	MSTRG.28539.3	3.5	1384-1401	1392	2	3	0.6
1790	osa-MIR6253-p3_1ss18AT	ATCATTTTCCTCTCATTT	MSTRG.5909.2	3.5	1184-1201	1192	2	4	0.444444444
1791	osa-MIR6253-p3_1ss18AT	ATCATTTTCCTCTCATTT	LOC_Os11g04520.2	3.5	1174-1191	1182	2	4	0.444444444
1792	osa-MIR6253-p3_1ss18AT	ATCATTTTCCTCTCATTT	LOC_Os11g04520.3	3.5	1177-1194	1185	2	4	0.444444444
1793	osa-MIR6253-p3_1ss18AT	ATCATTTTCCTCTCATTT	LOC_Os11g04520.1	3.5	1174-1191	1182	2	4	0.444444444
1794	osa-MIR6253-p3_1ss18AT	ATCATTTTCCTCTCATTT	MSTRG.5909.3	3.5	1309-1326	1317	2	4	0.444444444
1795	osa-MIR6253-p3_1ss18AT	ATCATTTTCCTCTCATTT	MSTRG.5909.1	3.5	1336-1353	1344	2	4	0.444444444
1796	osa-MIR6253-p3_1ss18AT	ATCATTTTCCTCTCATTT	MSTRG.9061.1	4	2288-2305	2296	2	3	1.5
1797	osa-MIR6253-p3_1ss18AT	ATCATTTTCCTCTCATTT	MSTRG.9901.1	4	857-873	864	2	2	1
1798	osa-MIR6253-p3_1ss18AT	ATCATTTTCCTCTCATTT	LOC_Os02g05500.1	4	1029-1045	1036	2	2	1
1799	osa-MIR6253-p3_1ss18AT	ATCATTTTCCTCTCATTT	LOC_Os01g07960.3	4	1013-1030	1021	2	9	1.8
1800	osa-MIR6253-p3_1ss18AT	ATCATTTTCCTCTCATTT	LOC_Os01g07960.1	4	1042-1059	1050	2	9	1.8
1801	osa-MIR6253-p3_1ss18AT	ATCATTTTCCTCTCATTT	MSTRG.485.5	4	1245-1262	1253	2	9	1.8
1802	osa-MIR6253-p3_1ss18AT	ATCATTTTCCTCTCATTT	MSTRG.485.4	4	1420-1437	1428	2	9	1.8
1803	osa-MIR6253-p3_1ss18AT	ATCATTTTCCTCTCATTT	LOC_Os01g07960.2	4	1403-1420	1411	2	9	1.8
1804	osa-MIR6253-p3_1ss18AT	ATCATTTTCCTCTCATTT	LOC_Os06g19470.4	3	1661-1678	1669	2	2	0.2
1805	osa-MIR6253-p3_1ss18AT	ATCATTTTCCTCTCATTT	LOC_Os06g19470.2	3	1376-1393	1384	2	2	0.2
1806	osa-MIR6253-p3_1ss18AT	ATCATTTTCCTCTCATTT	LOC_Os06g19470.1	3	1376-1393	1384	2	2	0.2
1807	osa-MIR6253-p3_1ss18AT	ATCATTTTCCTCTCATTT	MSTRG.22929.6	3	1462-1479	1470	4	1	0.1
1808	osa-MIR6253-p3_1ss18AT	ATCATTTTCCTCTCATTT	MSTRG.22929.8	3	1581-1598	1589	4	1	0.1
1809	osa-MIR6253-p3_1ss18AT	ATCATTTTCCTCTCATTT	LOC_Os06g19470.3	3	1661-1678	1669	4	1	0.1
1810	osa-MIR6253-p3_1ss18AT	ATCATTTTCCTCTCATTT	MSTRG.22929.4	3	1729-1746	1737	4	1	0.1
1811	osa-MIR6253-p3_1ss18AT	ATCATTTTCCTCTCATTT	MSTRG.22929.7	3	1447-1464	1455	4	1	0.1
1812	osa-MIR6253-p3_1ss18AT	ATCATTTTCCTCTCATTT	MSTRG.22929.11	3	1715-1732	1723	4	1	0.1
1813	osa-MIR6253-p3_1ss18AT	ATCATTTTCCTCTCATTT	MSTRG.22929.9	3	1444-1461	1452	4	1	0.1
1814	osa-MIR6253-p3_1ss18AT	ATCATTTTCCTCTCATTT	MSTRG.22929.5	3	1733-1750	1741	4	1	0.1
1815	osa-MIR6253-p3_1ss18AT	ATCATTTTCCTCTCATTT	MSTRG.22929.2	3	2052-2069	2060	2	2	0.2
1816	osa-MIR6253-p3_1ss18AT	ATCATTTTCCTCTCATTT	MSTRG.22929.10	3	1933-1950	1941	2	2	0.2
1817	osa-MIR6253-p3_1ss18AT	ATCATTTTCCTCTCATTT	MSTRG.22929.1	3	2052-2069	2060	2	2	0.2
1818	osa-MIR6253-p3_1ss18AT	ATCATTTTCCTCTCATTT	MSTRG.23965.9	4	1535-1552	1543	2	3	3
1819	osa-MIR6253-p3_1ss18AT	ATCATTTTCCTCTCATTT	LOC_Os08g25460.1	4	1449-1466	1457	2	3	3
1820	osa-MIR6253-p3_1ss18AT	ATCATTTTCCTCTCATTT	MSTRG.27859.5	4	1216-1232	1224	4	1	0.166666667
1821	osa-MIR6253-p3_1ss18AT	ATCATTTTCCTCTCATTT	MSTRG.27859.4	4	1302-1318	1310	4	1	0.166666667
1822	osa-MIR6253-p3_1ss18AT	ATCATTTTCCTCTCATTT	MSTRG.27859.2	4	1374-1390	1382	4	1	0.166666667
1823	osa-MIR6253-p3_1ss18AT	ATCATTTTCCTCTCATTT	MSTRG.27859.3	4	1374-1390	1382	4	1	0.166666667
1824	osa-MIR6253-p3_1ss18AT	ATCATTTTCCTCTCATTT	MSTRG.27859.1	4	1359-1375	1367	4	1	0.166666667
1825	osa-MIR6253-p3_1ss18AT	ATCATTTTCCTCTCATTT	MSTRG.27859.6	4	279-295	287	4	1	0.166666667
1826	osa-MIR6253-p3_1ss18AT	ATCATTTTCCTCTCATTT	LOC_Os12g04290.1	3.5	1148-1165	1156	2	4	0.444444444

1827	osa-MIR6253-p3_1ss18AT	ATCATTTTCCTCTCATTT	MSTRG.7817.2	3.5	1275-1292	1283	2	4	0.444444444
1828	osa-MIR6253-p3_1ss18AT	ATCATTTTCCTCTCATTT	MSTRG.7817.1	3.5	1313-1330	1321	2	4	0.444444444
1829	osa-miR810a	TCATAAGCCCACCACATGTGG	LOC_Os05g06470.1	4	1825-1845	1836	2	2	0.25
1830	osa-miR810a	TCATAAGCCCACCACATGTGG	MSTRG.19773.7	4	2127-2147	2138	2	2	0.25
1831	osa-miR810a	TCATAAGCCCACCACATGTGG	MSTRG.19773.4	4	2303-2323	2314	2	2	0.25
1832	osa-miR810a	TCATAAGCCCACCACATGTGG	MSTRG.19773.2	4	2334-2354	2345	2	2	0.25
1833	osa-miR810a	TCATAAGCCCACCACATGTGG	MSTRG.19773.6	4	2666-2686	2677	2	2	0.25
1834	osa-miR810a	TCATAAGCCCACCACATGTGG	MSTRG.19773.3	4	2562-2582	2573	2	2	0.25
1835	osa-miR810a	TCATAAGCCCACCACATGTGG	MSTRG.19773.1	4	2570-2590	2581	2	2	0.25
1836	osa-miR810a	TCATAAGCCCACCACATGTGG	MSTRG.19773.5	4	2649-2669	2660	2	2	0.25
1837	osa-miR810a	TCATAAGCCCACCACATGTGG	MSTRG.5804.2	4	919-939	930	4	1	0.5
1838	osa-miR810a	TCATAAGCCCACCACATGTGG	MSTRG.5804.1	4	965-985	976	4	1	0.5
1839	osa-miR812a_L+2_2ss13TC23AG	AAGACGGACGGTCAAACGTTGGGC	LOC_Os10g07340.1	2.5	977-1000	991	4	1	0.25
1840	osa-miR812a_L+2_2ss13TC23AG	AAGACGGACGGTCAAACGTTGGGC	MSTRG.4275.3	2.5	2775-2798	2789	4	1	0.25
1841	osa-miR812a_L+2_2ss13TC23AG	AAGACGGACGGTCAAACGTTGGGC	LOC_Os02g23823.2	0.5	388-411	402	4	1	1
1842	osa-miR812a_L+2_2ss13TC23AG	AAGACGGACGGTCAAACGTTGGGC	MSTRG.12236.1	3.5	218-240	231	0	2	2
1843	osa-miR812a_L+2_2ss13TC23AG	AAGACGGACGGTCAAACGTTGGGC	LOC_Os03g12620.2	1	1596-1619	1610	4	1	0.25
1844	osa-miR812a_L+2_2ss13TC23AG	AAGACGGACGGTCAAACGTTGGGC	LOC_Os03g12620.1	1	1608-1631	1622	4	1	0.25
1845	osa-miR812a_L+2_2ss13TC23AG	AAGACGGACGGTCAAACGTTGGGC	MSTRG.16394.2	3.5	523-545	536	4	1	0.142857143
1846	osa-miR812a_L+2_2ss13TC23AG	AAGACGGACGGTCAAACGTTGGGC	MSTRG.16394.1	3.5	519-541	532	4	1	0.142857143
1847	osa-miR812a_L+2_2ss13TC23AG	AAGACGGACGGTCAAACGTTGGGC	LOC_Os03g61950.1	4	2211-2234	2225	4	1	0.125
1848	osa-miR812a_L+2_2ss13TC23AG	AAGACGGACGGTCAAACGTTGGGC	LOC_Os03g61950.2	4	2616-2639	2630	4	1	0.125
1849	osa-miR812a_L+2_2ss13TC23AG	AAGACGGACGGTCAAACGTTGGGC	MSTRG.16459.3	4	3131-3154	3145	4	1	0.125
1850	osa-miR812a_L+2_2ss13TC23AG	AAGACGGACGGTCAAACGTTGGGC	LOC_Os06g04830.1	1.5	3587-3610	3601	4	1	0.25
1851	osa-miR812a_L+2_2ss13TC23AG	AAGACGGACGGTCAAACGTTGGGC	MSTRG.22131.5	1.5	4556-4579	4570	4	1	0.25
1852	osa-miR812a_L+2_2ss13TC23AG	AAGACGGACGGTCAAACGTTGGGC	MSTRG.22131.4	1.5	4716-4739	4730	4	1	0.25
1853	osa-miR812a_L+2_2ss13TC23AG	AAGACGGACGGTCAAACGTTGGGC	MSTRG.6812.3	2.5	3472-3495	3486	3	2	0.267857143
1854	osa-miR812a_L+2_2ss13TC23AG	AAGACGGACGGTCAAACGTTGGGC	MSTRG.6812.5	2.5	3529-3552	3543	3	2	0.267857143
1855	osa-miR812a_L+2_2ss13TC23AG	AAGACGGACGGTCAAACGTTGGGC	MSTRG.6812.4	2.5	3619-3642	3633	3	2	0.267857143
1856	osa-miR812a_L+2_2ss13TC23AG	AAGACGGACGGTCAAACGTTGGGC	MSTRG.14882.3	2.5	12-36	27	2	2	0.267857143
1857	osa-miR812a_L+2_2ss13TC23AG	AAGACGGACGGTCAAACGTTGGGC	LOC_Os03g50620.1	4	1116-1138	1129	2	5	2.5
1858	osa-miR812a_L+2_2ss13TC23AG	AAGACGGACGGTCAAACGTTGGGC	MSTRG.15681.3	4	2151-2173	2164	2	5	2.5
1859	osa-miR812a_L+2_2ss13TC23AG	AAGACGGACGGTCAAACGTTGGGC	MSTRG.8517.1	0.5	2043-2066	2057	4	1	0.25
1860	osa-miR812a_L+2_2ss13TC23AG	AAGACGGACGGTCAAACGTTGGGC	LOC_Os12g09570.2	2.5	2768-2792	2783	3	2	0.267857143
1861	osa-MIR812b-p5_1ss1CT	TGTTTGACCGTTCGTCTTATT	MSTRG.2528.4	1	991-1011	1002	4	1	0.125
1862	osa-MIR812b-p5_1ss1CT	TGTTTGACCGTTCGTCTTATT	MSTRG.2528.3	1	1058-1078	1069	4	1	0.125
1863	osa-MIR812b-p5_1ss1CT	TGTTTGACCGTTCGTCTTATT	MSTRG.2528.2	1	1573-1593	1584	4	1	0.125
1864	osa-MIR812b-p5_1ss1CT	TGTTTGACCGTTCGTCTTATT	MSTRG.2528.1	1	1730-1750	1741	4	1	0.125
1865	osa-MIR812b-p5_1ss1CT	TGTTTGACCGTTCGTCTTATT	LOC_Os02g13210.1	0	376-396	387	4	1	0.5
1866	osa-MIR812b-p5_1ss1CT	TGTTTGACCGTTCGTCTTATT	MSTRG.10472.3	1	2977-2997	2988	4	1	0.125
1867	osa-MIR812b-p5_1ss1CT	TGTTTGACCGTTCGTCTTATT	LOC_Os02g09990.1	2	908-928	919	4	1	0.5
1868	osa-MIR812b-p5_1ss1CT	TGTTTGACCGTTCGTCTTATT	LOC_Os03g29540.2	0	1014-1034	1025	2	2	0.4
1869	osa-MIR812b-p5_1ss1CT	TGTTTGACCGTTCGTCTTATT	MSTRG.20541.2	1	1289-1309	1300	4	1	0.125
1870	osa-MIR812b-p5_1ss1CT	TGTTTGACCGTTCGTCTTATT	MSTRG.29871.1	0	810-830	821	2	2	0.4
1871	osa-MIR812b-p5_1ss1CT	TGTTTGACCGTTCGTCTTATT	MSTRG.29871.2	0	955-975	966	2	2	0.4
1872	osa-MIR812b-p5_1ss1CT	TGTTTGACCGTTCGTCTTATT	MSTRG.29871.3	0	4054-4074	4065	2	2	0.4
1873	osa-MIR812b-p5_1ss1CT	TGTTTGACCGTTCGTCTTATT	MSTRG.599.2	1	77-97	88	4	1	0.5
1874	osa-MIR812b-p5_1ss1CT	TGTTTGACCGTTCGTCTTATT	MSTRG.1476.16	3	2561-2581	2572	4	1	0.5
1875	osa-MIR812b-p5_1ss1CT	TGTTTGACCGTTCGTCTTATT	LOC_Os06g14780.1	0	1173-1193	1184	3	2	0.4
1876	osa-MIR812b-p5_1ss1CT	TGTTTGACCGTTCGTCTTATT	MSTRG.23726.3	4	772-792	783	2	3	1
1877	osa-MIR812b-p5_1ss1CT	TGTTTGACCGTTCGTCTTATT	MSTRG.23726.1	4	1684-1704	1695	2	3	1
1878	osa-MIR812b-p5_1ss1CT	TGTTTGACCGTTCGTCTTATT	LOC_Os06g50146.1	2	1369-1389	1380	2	3	1.5
1879	osa-MIR812b-p5_1ss1CT	TGTTTGACCGTTCGTCTTATT	MSTRG.24397.1	2	1380-1400	1391	2	3	1.5
1880	osa-MIR812b-p5_1ss1CT	TGTTTGACCGTTCGTCTTATT	MSTRG.26800.4	1	1622-1642	1633	4	1	0.125
1881	osa-MIR812b-p5_1ss1CT	TGTTTGACCGTTCGTCTTATT	MSTRG.26800.1	1	6259-6279	6270	4	1	0.125
1882	osa-MIR812c-p5_1ss13AG	GTTTGACCGTCCGTCTTATTTGAA	LOC_Os01g70190.1	4	1899-1922	1913	4	1	0.333333333
1883	osa-MIR812c-p5_1ss13AG	GTTTGACCGTCCGTCTTATTTGAA	LOC_Os02g13210.1	3.5	372-395	386	4	1	0.333333333
1884	osa-MIR812c-p5_1ss13AG	GTTTGACCGTCCGTCTTATTTGAA	LOC_Os03g29540.2	3	1010-1033	1024	4	1	0.142857143
1885	osa-MIR812c-p5_1ss13AG	GTTTGACCGTCCGTCTTATTTGAA	LOC_Os03g60890.1	4	1463-1486	1477	2	3	0.452380952

1886	osa-MIR812c-p5_1ss13AG	GTTTGACCGTCCGTCTTATTTGAA	MSTRG.16379.2	4	2297-2320	2311	2	3	0.452380952
1887	osa-MIR812c-p5_1ss13AG	GTTTGACCGTCCGTCTTATTTGAA	MSTRG.16379.4	4	2312-2335	2326	2	3	0.452380952
1888	osa-MIR812c-p5_1ss13AG	GTTTGACCGTCCGTCTTATTTGAA	MSTRG.16379.6	4	2883-2906	2897	2	3	0.452380952
1889	osa-MIR812c-p5_1ss13AG	GTTTGACCGTCCGTCTTATTTGAA	MSTRG.16379.3	4	3097-3120	3111	2	3	0.452380952
1890	osa-MIR812c-p5_1ss13AG	GTTTGACCGTCCGTCTTATTTGAA	MSTRG.16379.5	4	3788-3811	3802	2	3	0.452380952
1891	osa-MIR812c-p5_1ss13AG	GTTTGACCGTCCGTCTTATTTGAA	MSTRG.29871.1	3	806-829	820	4	1	0.142857143
1892	osa-MIR812c-p5_1ss13AG	GTTTGACCGTCCGTCTTATTTGAA	MSTRG.29871.2	3	951-974	965	4	1	0.142857143
1893	osa-MIR812c-p5_1ss13AG	GTTTGACCGTCCGTCTTATTTGAA	MSTRG.29871.3	3	4050-4073	4064	4	1	0.142857143
1894	osa-MIR812c-p5_1ss13AG	GTTTGACCGTCCGTCTTATTTGAA	MSTRG.9971.1	4	1499-1522	1513	3	2	0.285714286
1895	osa-MIR812c-p5_1ss13AG	GTTTGACCGTCCGTCTTATTTGAA	LOC_Os06g14780.1	2.5	1169-1192	1183	4	1	0.142857143
1896	osa-MIR812c-p5_1ss13AG	GTTTGACCGTCCGTCTTATTTGAA	MSTRG.24115.2	4	52-75	66	4	1	0.142857143
1897	osa-MIR812g-p3_1ss11TC	ATAAGACGGACGATTAAAGTTGGA	LOC_Os02g23823.2	3.5	389-413	404	2	2	2
1898	osa-MIR812g-p3_1ss11TC	ATAAGACGGACGATTAAAGTTGGA	LOC_Os03g12620.2	4	1597-1621	1612	4	1	0.2
1899	osa-MIR812g-p3_1ss11TC	ATAAGACGGACGATTAAAGTTGGA	LOC_Os03g12620.1	4	1609-1633	1624	4	1	0.2
1900	osa-MIR812g-p3_1ss11TC	ATAAGACGGACGATTAAAGTTGGA	MSTRG.16394.2	1.5	524-547	538	4	1	0.5
1901	osa-MIR812g-p3_1ss11TC	ATAAGACGGACGATTAAAGTTGGA	MSTRG.16394.1	1.5	520-543	534	4	1	0.5
1902	osa-MIR812h-p3_1ss9AG	AGTTGGACGCGAAAACTCATGGCT	LOC_Os06g50146.1	3.5	1224-1247	1238	4	1	0.142857143
1903	osa-MIR812h-p3_1ss9AG	AGTTGGACGCGAAAACTCATGGCT	MSTRG.24397.1	3.5	1235-1258	1249	4	1	0.142857143
1904	osa-MIR812k-p3_1ss18CT	AGTTGGACGCGGAAAACTATGGCT	MSTRG.13772.2	3.5	1792-1815	1806	4	1	0.142857143
1905	osa-MIR812k-p3_1ss18CT	AGTTGGACGCGGAAAACTATGGCT	MSTRG.13772.1	3.5	2064-2087	2078	4	1	0.142857143
1906	osa-MIR812k-p3_1ss18CT	AGTTGGACGCGGAAAACTATGGCT	MSTRG.23507.1	3.5	23-46	37	4	1	0.142857143
1907	osa-MIR812m-p3_1ss21AG	AGTTGGACGCGGAAAACTATGGCT	MSTRG.13772.2	3.5	1792-1815	1806	4	1	0.142857143
1908	osa-MIR812m-p3_1ss21AG	AGTTGGACGCGGAAAACTATGGCT	MSTRG.13772.1	3.5	2064-2087	2078	4	1	0.142857143
1909	osa-MIR812m-p3_1ss21AG	AGTTGGACGCGGAAAACTATGGCT	MSTRG.23507.1	3.5	23-46	37	4	1	0.142857143
1910	osa-MIR812p-p3_1ss16TC	AATAAGACGGACGATCAAAGTTGG	LOC_Os03g50620.1	3.5	1118-1141	1132	2	2	1
1911	osa-MIR812p-p3_1ss16TC	AATAAGACGGACGATCAAAGTTGG	MSTRG.15681.3	3.5	2153-2176	2167	2	2	1
1912	osa-MIR812r-p3_1ss18CT	TAAGACGGACGGTCAAATGTT	LOC_Os02g23823.2	0.5	392-412	403	2	6	6
1913	osa-MIR812r-p3_1ss18CT	TAAGACGGACGGTCAAATGTT	MSTRG.21443.1	2	34-53	44	2	2	0.4
1914	osa-MIR812r-p3_1ss18CT	TAAGACGGACGGTCAAATGTT	LOC_Os11g34910.1	3.5	2653-2673	2664	4	1	1
1915	osa-MIR812r-p3_1ss18CT	TAAGACGGACGGTCAAATGTT	LOC_Os12g38051.1	3	758-778	769	2	2	1
1916	osa-MIR812r-p3_1ss18CT	TAAGACGGACGGTCAAATGTT	LOC_Os01g06740.1	1.5	1203-1223	1214	2	2	0.4
1917	osa-MIR812r-p3_1ss18CT	TAAGACGGACGGTCAAATGTT	LOC_Os03g50620.1	3	1120-1139	1130	2	5	2.5
1918	osa-MIR812r-p3_1ss18CT	TAAGACGGACGGTCAAATGTT	MSTRG.15681.3	3	2155-2174	2165	2	5	2.5
1919	osa-MIR812r-p3_1ss18CT	TAAGACGGACGGTCAAATGTT	MSTRG.23507.1	2	43-62	53	2	2	0.4
1920	osa-MIR812r-p3_1ss18CT	TAAGACGGACGGTCAAATGTT	LOC_Os06g50146.1	4	1244-1263	1254	3	2	0.4
1921	osa-MIR812r-p3_1ss18CT	TAAGACGGACGGTCAAATGTT	MSTRG.24397.1	4	1255-1274	1265	3	2	0.4
1922	osa-MIR812r-p5_1ss1CT	TGTTTGACCGTTCGTCTTATT	MSTRG.2528.4	1	991-1011	1002	4	1	0.125
1923	osa-MIR812r-p5_1ss1CT	TGTTTGACCGTTCGTCTTATT	MSTRG.2528.3	1	1058-1078	1069	4	1	0.125
1924	osa-MIR812r-p5_1ss1CT	TGTTTGACCGTTCGTCTTATT	MSTRG.2528.2	1	1573-1593	1584	4	1	0.125
1925	osa-MIR812r-p5_1ss1CT	TGTTTGACCGTTCGTCTTATT	MSTRG.2528.1	1	1730-1750	1741	4	1	0.125
1926	osa-MIR812r-p5_1ss1CT	TGTTTGACCGTTCGTCTTATT	LOC_Os02g13210.1	0	376-396	387	4	1	0.5
1927	osa-MIR812r-p5_1ss1CT	TGTTTGACCGTTCGTCTTATT	MSTRG.10472.3	1	2977-2997	2988	4	1	0.125
1928	osa-MIR812r-p5_1ss1CT	TGTTTGACCGTTCGTCTTATT	LOC_Os02g09990.1	2	908-928	919	4	1	0.5
1929	osa-MIR812r-p5_1ss1CT	TGTTTGACCGTTCGTCTTATT	LOC_Os03g29540.2	0	1014-1034	1025	2	2	0.4
1930	osa-MIR812r-p5_1ss1CT	TGTTTGACCGTTCGTCTTATT	MSTRG.20541.2	1	1289-1309	1300	4	1	0.125
1931	osa-MIR812r-p5_1ss1CT	TGTTTGACCGTTCGTCTTATT	MSTRG.29871.1	0	810-830	821	2	2	0.4
1932	osa-MIR812r-p5_1ss1CT	TGTTTGACCGTTCGTCTTATT	MSTRG.29871.2	0	955-975	966	2	2	0.4
1933	osa-MIR812r-p5_1ss1CT	TGTTTGACCGTTCGTCTTATT	MSTRG.29871.3	0	4054-4074	4065	2	2	0.4
1934	osa-MIR812r-p5_1ss1CT	TGTTTGACCGTTCGTCTTATT	MSTRG.599.2	1	77-97	88	4	1	0.5
1935	osa-MIR812r-p5_1ss1CT	TGTTTGACCGTTCGTCTTATT	MSTRG.1476.16	3	2561-2581	2572	4	1	0.5
1936	osa-MIR812r-p5_1ss1CT	TGTTTGACCGTTCGTCTTATT	LOC_Os06g14780.1	0	1173-1193	1184	3	2	0.4
1937	osa-MIR812r-p5_1ss1CT	TGTTTGACCGTTCGTCTTATT	MSTRG.23726.3	4	772-792	783	2	3	1
1938	osa-MIR812r-p5_1ss1CT	TGTTTGACCGTTCGTCTTATT	MSTRG.23726.1	4	1684-1704	1695	2	3	1
1939	osa-MIR812r-p5_1ss1CT	TGTTTGACCGTTCGTCTTATT	LOC_Os06g50146.1	2	1369-1389	1380	2	3	1.5
1940	osa-MIR812r-p5_1ss1CT	TGTTTGACCGTTCGTCTTATT	MSTRG.24397.1	2	1380-1400	1391	2	3	1.5
1941	osa-MIR812r-p5_1ss1CT	TGTTTGACCGTTCGTCTTATT	MSTRG.26800.4	1	1622-1642	1633	4	1	0.125
1942	osa-MIR812r-p5_1ss1CT	TGTTTGACCGTTCGTCTTATT	MSTRG.26800.1	1	6259-6279	6270	4	1	0.125
1943	osa-miR812s_2ss10AG23AG	AAGACGGACGATCAAACGTTGGGC	LOC_Os02g23823.2	2.5	388-411	402	4	1	1
1944	osa-miR812s_2ss10AG23AG	AAGACGGACGATCAAACGTTGGGC	LOC_Os03g12620.2	3	1596-1619	1610	4	1	0.25

1945	osa-miR812s_2ss10AG23AG	AAGACGGACGATCAAACGTTGGGC	LOC_Os03g12620.1	3	1608-1631	1622	4	1	0.25
1946	osa-miR812s_2ss10AG23AG	AAGACGGACGATCAAACGTTGGGC	MSTRG.16394.2	2.5	523-545	536	4	1	0.142857143
1947	osa-miR812s_2ss10AG23AG	AAGACGGACGATCAAACGTTGGGC	MSTRG.16394.1	2.5	519-541	532	4	1	0.142857143
1948	osa-miR812s_2ss10AG23AG	AAGACGGACGATCAAACGTTGGGC	LOC_Os06g04830.1	3.5	3587-3610	3601	4	1	0.25
1949	osa-miR812s_2ss10AG23AG	AAGACGGACGATCAAACGTTGGGC	MSTRG.22131.5	3.5	4556-4579	4570	4	1	0.25
1950	osa-miR812s_2ss10AG23AG	AAGACGGACGATCAAACGTTGGGC	MSTRG.22131.4	3.5	4716-4739	4730	4	1	0.25
1951	osa-miR812s_2ss10AG23AG	AAGACGGACGATCAAACGTTGGGC	MSTRG.6812.3	1.5	3472-3495	3486	3	2	0.267857143
1952	osa-miR812s_2ss10AG23AG	AAGACGGACGATCAAACGTTGGGC	MSTRG.6812.5	1.5	3529-3552	3543	3	2	0.267857143
1953	osa-miR812s_2ss10AG23AG	AAGACGGACGATCAAACGTTGGGC	MSTRG.6812.4	1.5	3619-3642	3633	3	2	0.267857143
1954	osa-miR812s_2ss10AG23AG	AAGACGGACGATCAAACGTTGGGC	MSTRG.8517.1	2.5	2043-2066	2057	4	1	0.25
1955	osa-MIR814a-p3_2ss20TG24AT	GATTCGTTGTACTATGAAGGGTCT	MSTRG.26498.1	3	1112-1135	1126	2	2	0.25
1956	osa-MIR814a-p3_2ss20TG24AT	GATTCGTTGTACTATGAAGGGTCT	LOC_Os07g45350.2	3	1262-1285	1276	2	2	0.25
1957	osa-MIR814a-p3_2ss20TG24AT	GATTCGTTGTACTATGAAGGGTCT	LOC_Os07g45350.4	3	1330-1353	1344	2	2	0.25
1958	osa-MIR814a-p3_2ss20TG24AT	GATTCGTTGTACTATGAAGGGTCT	LOC_Os07g45350.5	3	1353-1376	1367	2	2	0.25
1959	osa-MIR814a-p3_2ss20TG24AT	GATTCGTTGTACTATGAAGGGTCT	LOC_Os07g45350.1	3	1357-1380	1371	2	2	0.25
1960	osa-MIR814a-p3_2ss20TG24AT	GATTCGTTGTACTATGAAGGGTCT	MSTRG.26498.8	3	1444-1467	1458	2	2	0.25
1961	osa-MIR814a-p3_2ss20TG24AT	GATTCGTTGTACTATGAAGGGTCT	MSTRG.26498.2	3	1396-1419	1410	2	2	0.25
1962	osa-MIR814a-p3_2ss20TG24AT	GATTCGTTGTACTATGAAGGGTCT	LOC_Os07g45350.3	3	1760-1783	1774	2	2	0.25
1963	osa-MIR815a-p3_1ss24GA	TAGGGTGTGTTTGAGGAGAAGGGA	MSTRG.21470.2	4	27-50	41	4	1	0.333333333
1964	osa-MIR815a-p3_1ss24GA	TAGGGTGTGTTTGAGGAGAAGGGA	MSTRG.21470.3	4	25-48	39	4	1	0.333333333
1965	osa-MIR815a-p3_1ss24GA	TAGGGTGTGTTTGAGGAGAAGGGA	LOC_Os05g45660.2	4	47-70	61	4	1	0.333333333
1966	osa-MIR815a-p3_1ss24GA	TAGGGTGTGTTTGAGGAGAAGGGA	MSTRG.15210.6	2.5	1269-1292	1283	2	4	1.333333333
1967	osa-MIR815a-p3_1ss24GA	TAGGGTGTGTTTGAGGAGAAGGGA	MSTRG.15210.7	2.5	1328-1351	1342	2	4	1.333333333
1968	osa-MIR815a-p3_1ss24GA	TAGGGTGTGTTTGAGGAGAAGGGA	MSTRG.15210.1	2.5	2034-2057	2048	2	4	1.333333333
1969	osa-MIR816-p3_1ss11TC	ATTCGTAGTACTAGGATGTGTCAC	LOC_Os04g31120.1	4	1923-1946	1937	4	1	0.1
1970	osa-MIR816-p3_1ss11TC	ATTCGTAGTACTAGGATGTGTCAC	LOC_Os04g31120.6	4	1982-2005	1996	4	1	0.1
1971	osa-MIR816-p3_1ss11TC	ATTCGTAGTACTAGGATGTGTCAC	MSTRG.17563.4	4	1819-1842	1833	4	1	0.1
1972	osa-MIR816-p3_1ss11TC	ATTCGTAGTACTAGGATGTGTCAC	LOC_Os04g31120.2	4	2057-2080	2071	4	1	0.1
1973	osa-MIR816-p3_1ss11TC	ATTCGTAGTACTAGGATGTGTCAC	MSTRG.17563.2	4	1899-1922	1913	4	1	0.1
1974	osa-MIR816-p3_1ss11TC	ATTCGTAGTACTAGGATGTGTCAC	MSTRG.17563.1	4	1968-1991	1982	4	1	0.1
1975	osa-MIR816-p3_1ss11TC	ATTCGTAGTACTAGGATGTGTCAC	LOC_Os04g31120.4	4	2415-2438	2429	4	1	0.1
1976	osa-MIR816-p3_1ss11TC	ATTCGTAGTACTAGGATGTGTCAC	MSTRG.17563.3	4	2226-2249	2240	4	1	0.1
1977	osa-MIR816-p3_1ss11TC	ATTCGTAGTACTAGGATGTGTCAC	LOC_Os04g31120.3	4	2492-2515	2506	4	1	0.1
1978	osa-MIR816-p3_1ss11TC	ATTCGTAGTACTAGGATGTGTCAC	LOC_Os04g31120.5	4	3951-3974	3965	4	1	0.1
1979	osa-miR818a_L+1R+1_2ss6CT17GA	AAATCTCTTATATTATAGGACGGA	LOC_Os02g14290.1	4	975-998	989	2	3	0.333333333
1980	osa-miR818a_L+1R+1_2ss6CT17GA	AAATCTCTTATATTATAGGACGGA	MSTRG.10528.6	4	1062-1085	1076	2	3	0.333333333
1981	osa-miR818a_L+1R+1_2ss6CT17GA	AAATCTCTTATATTATAGGACGGA	MSTRG.10528.3	4	1170-1193	1184	2	3	0.333333333
1982	osa-miR818a_L+1R+1_2ss6CT17GA	AAATCTCTTATATTATAGGACGGA	MSTRG.10528.7	4	1437-1460	1451	2	3	0.333333333
1983	osa-miR818a_L+1R+1_2ss6CT17GA	AAATCTCTTATATTATAGGACGGA	MSTRG.10528.4	4	1260-1283	1274	2	3	0.333333333
1984	osa-miR818a_L+1R+1_2ss6CT17GA	AAATCTCTTATATTATAGGACGGA	MSTRG.10528.8	4	1649-1672	1663	2	3	0.333333333
1985	osa-miR818a_L+1R+1_2ss6CT17GA	AAATCTCTTATATTATAGGACGGA	MSTRG.10528.5	4	1477-1500	1491	2	3	0.333333333
1986	osa-miR818a_L+1R+1_2ss6CT17GA	AAATCTCTTATATTATAGGACGGA	MSTRG.10528.1	4	1323-1346	1337	2	3	0.333333333
1987	osa-miR818a_L+1R+1_2ss6CT17GA	AAATCTCTTATATTATAGGACGGA	MSTRG.10528.2	4	1595-1618	1609	2	3	0.333333333
1988	osa-miR818a_L+1R+1_2ss6CT17GA	AAATCTCTTATATTATAGGACGGA	MSTRG.19222.8	3	1180-1203	1194	2	2	0.285714286
1989	osa-miR818a_L+1R+1_2ss6CT17GA	AAATCTCTTATATTATAGGACGGA	MSTRG.19222.2	3	1233-1256	1247	2	2	0.285714286
1990	osa-miR818a_L+1R+1_2ss6CT17GA	AAATCTCTTATATTATAGGACGGA	MSTRG.19222.5	3	1508-1531	1522	2	2	0.285714286
1991	osa-miR818a_L+1R+1_2ss6CT17GA	AAATCTCTTATATTATAGGACGGA	MSTRG.19222.4	3	1553-1576	1567	2	2	0.285714286
1992	osa-miR818a_L+1R+1_2ss6CT17GA	AAATCTCTTATATTATAGGACGGA	MSTRG.19222.1	3	1243-1266	1257	2	2	0.285714286
1993	osa-miR818a_L+1R+1_2ss6CT17GA	AAATCTCTTATATTATAGGACGGA	MSTRG.19222.9	3	2342-2365	2356	2	2	0.285714286
1994	osa-miR818a_L+1R+1_2ss6CT17GA	AAATCTCTTATATTATAGGACGGA	MSTRG.19222.3	3	2705-2728	2719	2	2	0.285714286
1995	osa-miR818a_L+1R+1_2ss6CT17GA	AAATCTCTTATATTATAGGACGGA	MSTRG.21548.3	2	1793-1816	1807	4	1	1
1996	osa-miR818a_L+1R+1_2ss6CT17GA	AAATCTCTTATATTATAGGACGGA	MSTRG.28819.1	3	785-808	799	3	4	4
1997	osa-miR818a_L+1R+1_2ss6CT17GA	AAATCTCTTATATTATAGGACGGA	LOC_Os01g15970.1	1	545-568	559	2	2	2
1998	osa-miR818a_L+1R+1_2ss6CT17GA	AAATCTCTTATATTATAGGACGGA	MSTRG.794.4	3.5	1426-1449	1440	2	2	0.5
1999	osa-miR818a_L+1R+1_2ss6CT17GA	AAATCTCTTATATTATAGGACGGA	MSTRG.794.3	3.5	1287-1310	1301	2	2	0.5
2000	osa-miR818a_L+1R+1_2ss6CT17GA	AAATCTCTTATATTATAGGACGGA	LOC_Os01g12820.1	3.5	1389-1412	1403	2	2	0.5
2001	osa-miR818a_L+1R+1_2ss6CT17GA	AAATCTCTTATATTATAGGACGGA	MSTRG.794.1	3.5	2002-2025	2016	2	2	0.5
2002	osa-miR818a_L+1R+1_2ss6CT17GA	AAATCTCTTATATTATAGGACGGA	MSTRG.22572.16	2	1025-1048	1039	4	1	0.125
2003	osa-miR818a_L+1R+1_2ss6CT17GA	AAATCTCTTATATTATAGGACGGA	MSTRG.22572.7	2	2343-2366	2357	4	1	0.125

2004	osa-miR818a_L+1R+1_2ss6CT17GA	AAATCTCTTATATTATAGGACGGA	MSTRG.22572.6	2	2352-2375	2366	4	1	0.125
2005	osa-miR818a_L+1R+1_2ss6CT17GA	AAATCTCTTATATTATAGGACGGA	MSTRG.22572.1	2	2352-2375	2366	4	1	0.125
2006	osa-miR818a_L+1R+1_2ss6CT17GA	AAATCTCTTATATTATAGGACGGA	MSTRG.22572.3	2	2351-2374	2365	4	1	0.125
2007	osa-miR818a_L+1R+1_2ss6CT17GA	AAATCTCTTATATTATAGGACGGA	LOC_Os06g11500.1	2	2484-2507	2498	4	1	0.125
2008	osa-miR818a_L+1R+1_2ss6CT17GA	AAATCTCTTATATTATAGGACGGA	MSTRG.22572.15	2	3422-3445	3436	4	1	0.125
2009	osa-miR818a_L+1R+1_2ss6CT17GA	AAATCTCTTATATTATAGGACGGA	MSTRG.22572.13	2	3422-3445	3436	4	1	0.125
2010	osa-miR818a_L-1R+3	ATCCCTTATATTATGGGACGGAGG	MSTRG.2373.2	0.5	4484-4507	4498	2	2	0.666666667
2011	osa-miR818a_L-1R+3	ATCCCTTATATTATGGGACGGAGG	MSTRG.2373.1	0.5	4534-4557	4548	2	2	0.666666667
2012	osa-miR818a_L-1R+3	ATCCCTTATATTATGGGACGGAGG	LOC_Os02g14290.1	2	973-996	987	2	3	0.333333333
2013	osa-miR818a_L-1R+3	ATCCCTTATATTATGGGACGGAGG	MSTRG.10528.6	2	1060-1083	1074	2	3	0.333333333
2014	osa-miR818a_L-1R+3	ATCCCTTATATTATGGGACGGAGG	MSTRG.10528.3	2	1168-1191	1182	2	3	0.333333333
2015	osa-miR818a_L-1R+3	ATCCCTTATATTATGGGACGGAGG	MSTRG.10528.7	2	1435-1458	1449	2	3	0.333333333
2016	osa-miR818a_L-1R+3	ATCCCTTATATTATGGGACGGAGG	MSTRG.10528.4	2	1258-1281	1272	2	3	0.333333333
2017	osa-miR818a_L-1R+3	ATCCCTTATATTATGGGACGGAGG	MSTRG.10528.8	2	1647-1670	1661	2	3	0.333333333
2018	osa-miR818a_L-1R+3	ATCCCTTATATTATGGGACGGAGG	MSTRG.10528.5	2	1475-1498	1489	2	3	0.333333333
2019	osa-miR818a_L-1R+3	ATCCCTTATATTATGGGACGGAGG	MSTRG.10528.1	2	1321-1344	1335	2	3	0.333333333
2020	osa-miR818a_L-1R+3	ATCCCTTATATTATGGGACGGAGG	MSTRG.10528.2	2	1593-1616	1607	2	3	0.333333333
2021	osa-miR818a_L-1R+3	ATCCCTTATATTATGGGACGGAGG	MSTRG.18087.4	0.5	41-64	55	2	4	1.75
2022	osa-miR818a_L-1R+3	ATCCCTTATATTATGGGACGGAGG	LOC_Os07g40450.2	0	88-111	102	2	2	0.75
2023	osa-miR818a_L-1R+3	ATCCCTTATATTATGGGACGGAGG	LOC_Os07g40450.1	0	88-111	102	3	2	0.75
2024	osa-miR818a_L-1R+3	ATCCCTTATATTATGGGACGGAGG	MSTRG.28819.1	2	783-806	797	3	4	1.75
2025	osa-miR818a_L-1R+3	ATCCCTTATATTATGGGACGGAGG	MSTRG.794.4	1.5	1424-1447	1438	4	1	0.25
2026	osa-miR818a_L-1R+3	ATCCCTTATATTATGGGACGGAGG	MSTRG.794.3	1.5	1285-1308	1299	4	1	0.25
2027	osa-miR818a_L-1R+3	ATCCCTTATATTATGGGACGGAGG	LOC_Os01g12820.1	1.5	1387-1410	1401	4	1	0.25
2028	osa-miR818a_L-1R+3	ATCCCTTATATTATGGGACGGAGG	MSTRG.794.1	1.5	2000-2023	2014	4	1	0.25
2029	osa-miR818a_L-1R+3	ATCCCTTATATTATGGGACGGAGG	LOC_Os08g29760.1	2.5	1902-1925	1916	4	1	1
2030	osa-MIR818b-p5_2ss16AG19TG	AATATAAGAGATTTTGGAGGGATG	MSTRG.20874.3	4	1275-1298	1289	4	1	0.333333333
2031	osa-MIR818b-p5_2ss16AG19TG	AATATAAGAGATTTTGGAGGGATG	MSTRG.20874.4	4	1278-1301	1292	4	1	0.333333333
2032	osa-MIR818b-p5_2ss16AG19TG	AATATAAGAGATTTTGGAGGGATG	LOC_Os05g34700.1	4	1372-1395	1386	4	1	0.333333333
2033	osa-MIR818d-p3_2ss13GT17GT	TATTATGGGACGTAGGTAGTA	MSTRG.2373.3	4	4345-4365	4356	4	1	0.333333333
2034	osa-MIR818d-p3_2ss13GT17GT	TATTATGGGACGTAGGTAGTA	MSTRG.2373.2	4	4479-4499	4490	4	1	0.333333333
2035	osa-MIR818d-p3_2ss13GT17GT	TATTATGGGACGTAGGTAGTA	MSTRG.2373.1	4	4529-4549	4540	4	1	0.333333333
2036	osa-MIR818d-p3_2ss13GT17GT	TATTATGGGACGTAGGTAGTA	MSTRG.13526.2	4	1790-1810	1801	4	1	0.5
2037	osa-MIR818d-p3_2ss13GT17GT	TATTATGGGACGTAGGTAGTA	LOC_Os03g08530.1	4	1871-1891	1882	4	1	0.5
2038	osa-MIR818e-p5	AAGGGATTTTGAGTTTTTGCTTGC	MSTRG.22572.16	2	1213-1236	1227	2	2	0.25
2039	osa-MIR818e-p5	AAGGGATTTTGAGTTTTTGCTTGC	MSTRG.22572.7	2	2531-2554	2545	2	2	0.25
2040	osa-MIR818e-p5	AAGGGATTTTGAGTTTTTGCTTGC	MSTRG.22572.6	2	2540-2563	2554	2	2	0.25
2041	osa-MIR818e-p5	AAGGGATTTTGAGTTTTTGCTTGC	MSTRG.22572.1	2	2540-2563	2554	2	2	0.25
2042	osa-MIR818e-p5	AAGGGATTTTGAGTTTTTGCTTGC	MSTRG.22572.3	2	2539-2562	2553	2	2	0.25
2043	osa-MIR818e-p5	AAGGGATTTTGAGTTTTTGCTTGC	LOC_Os06g11500.1	2	2672-2695	2686	2	2	0.25
2044	osa-MIR818e-p5	AAGGGATTTTGAGTTTTTGCTTGC	MSTRG.22572.15	2	3610-3633	3624	2	2	0.25
2045	osa-MIR818e-p5	AAGGGATTTTGAGTTTTTGCTTGC	MSTRG.22572.13	2	3610-3633	3624	2	2	0.25
2046	osa-MIR818e-p5	AAGGGATTTTGAGTTTTTGCTTGC	MSTRG.8243.2	0	1748-1771	1762	4	1	0.2
2047	osa-MIR818e-p5	AAGGGATTTTGAGTTTTTGCTTGC	MSTRG.8243.9	0	1665-1688	1679	4	1	0.2
2048	osa-MIR818e-p5	AAGGGATTTTGAGTTTTTGCTTGC	MSTRG.8243.4	0	1111-1134	1125	4	1	0.2
2049	osa-MIR818e-p5	AAGGGATTTTGAGTTTTTGCTTGC	MSTRG.8243.1	0	1748-1771	1762	4	1	0.2
2050	osa-MIR818e-p5	AAGGGATTTTGAGTTTTTGCTTGC	MSTRG.8243.6	0	1992-2015	2006	4	1	0.2
2051	osa-MIR818f-p3_1ss15TC	ATAAGACGAGTGGTCAAACAGTGC	MSTRG.2528.4	3	863-887	878	2	3	0.666666667
2052	osa-MIR818f-p3_1ss15TC	ATAAGACGAGTGGTCAAACAGTGC	MSTRG.2528.3	3	930-954	945	2	3	0.666666667
2053	osa-MIR818f-p3_1ss15TC	ATAAGACGAGTGGTCAAACAGTGC	MSTRG.2528.2	3	1445-1469	1460	2	2	0.416666667
2054	osa-MIR818f-p3_1ss15TC	ATAAGACGAGTGGTCAAACAGTGC	MSTRG.2528.1	3	1602-1626	1617	2	3	0.666666667
2055	osa-MIR818f-p3_1ss15TC	ATAAGACGAGTGGTCAAACAGTGC	MSTRG.3085.4	3	2769-2793	2784	4	1	0.25
2056	osa-MIR818f-p3_1ss15TC	ATAAGACGAGTGGTCAAACAGTGC	MSTRG.11039.10	2.5	11761-11785	11776	4	1	0.5
2057	osa-MIR818f-p3_1ss15TC	ATAAGACGAGTGGTCAAACAGTGC	MSTRG.10472.3	3	2849-2873	2864	2	5	0.714285714
2058	osa-MIR818f-p3_1ss15TC	ATAAGACGAGTGGTCAAACAGTGC	LOC_Os05g49120.2	3.5	1620-1644	1635	4	1	0.25
2059	osa-MIR818f-p3_1ss15TC	ATAAGACGAGTGGTCAAACAGTGC	LOC_Os05g49120.1	3.5	1735-1759	1750	4	1	0.25
2060	osa-MIR818f-p3_1ss15TC	ATAAGACGAGTGGTCAAACAGTGC	LOC_Os07g39290.1	2.5	999-1023	1014	4	1	0.5
2061	osa-MIR818f-p3_1ss15TC	ATAAGACGAGTGGTCAAACAGTGC	MSTRG.6867.3	3	2429-2453	2444	2	5	0.714285714
2062	osa-MIR818f-p3_1ss15TC	ATAAGACGAGTGGTCAAACAGTGC	MSTRG.6867.6	3	2624-2648	2639	2	5	0.714285714

2063	osa-MIR818f-p3_1ss15TC	ATAAGACGAGTGGTCAAACAGTGC	MSTRG.6867.7	3	2630-2654	2645	2	5	0.714285714
2064	osa-MIR818f-p3_1ss15TC	ATAAGACGAGTGGTCAAACAGTGC	MSTRG.6867.5	3	2641-2665	2656	2	5	0.714285714
2065	osa-MIR818f-p3_1ss15TC	ATAAGACGAGTGGTCAAACAGTGC	MSTRG.6867.4	3	2635-2659	2650	2	5	0.714285714
2066	osa-MIR818f-p3_1ss15TC	ATAAGACGAGTGGTCAAACAGTGC	MSTRG.28044.8	4	737-761	752	2	2	0.416666667
2067	osa-MIR818f-p3_1ss15TC	ATAAGACGAGTGGTCAAACAGTGC	MSTRG.28044.2	4	927-951	942	4	1	0.166666667
2068	osa-MIR818f-p3_1ss15TC	ATAAGACGAGTGGTCAAACAGTGC	MSTRG.26800.1	4	6131-6155	6146	4	1	0.25
2069	PC-3p-102885_34	TGTAGGAAATGCTAGAAAGTC	LOC_Os04g58070.1	4	1519-1539	1530	4	1	0.5
2070	PC-3p-107648_32	AAGACGGACGGTCAAATATTGGAC	LOC_Os10g07340.1	3.5	977-1000	991	4	1	0.25
2071	PC-3p-107648_32	AAGACGGACGGTCAAATATTGGAC	MSTRG.4275.3	3.5	2775-2798	2789	4	1	0.25
2072	PC-3p-107648_32	AAGACGGACGGTCAAATATTGGAC	LOC_Os02g23823.2	1.5	388-411	402	4	1	1
2073	PC-3p-107648_32	AAGACGGACGGTCAAATATTGGAC	LOC_Os03g12620.2	2	1596-1619	1610	4	1	0.25
2074	PC-3p-107648_32	AAGACGGACGGTCAAATATTGGAC	LOC_Os03g12620.1	2	1608-1631	1622	4	1	0.25
2075	PC-3p-107648_32	AAGACGGACGGTCAAATATTGGAC	MSTRG.16394.2	4	523-545	536	4	1	0.142857143
2076	PC-3p-107648_32	AAGACGGACGGTCAAATATTGGAC	MSTRG.16394.1	4	519-541	532	4	1	0.142857143
2077	PC-3p-107648_32	AAGACGGACGGTCAAATATTGGAC	LOC_Os06g04830.1	2.5	3587-3610	3601	4	1	0.25
2078	PC-3p-107648_32	AAGACGGACGGTCAAATATTGGAC	MSTRG.22131.5	2.5	4556-4579	4570	4	1	0.25
2079	PC-3p-107648_32	AAGACGGACGGTCAAATATTGGAC	MSTRG.22131.4	2.5	4716-4739	4730	4	1	0.25
2080	PC-3p-107648_32	AAGACGGACGGTCAAATATTGGAC	MSTRG.21356.2	4	536-558	549	4	1	0.5
2081	PC-3p-107648_32	AAGACGGACGGTCAAATATTGGAC	MSTRG.6812.3	3.5	3472-3495	3486	3	2	0.267857143
2082	PC-3p-107648_32	AAGACGGACGGTCAAATATTGGAC	MSTRG.6812.5	3.5	3529-3552	3543	3	2	0.267857143
2083	PC-3p-107648_32	AAGACGGACGGTCAAATATTGGAC	MSTRG.6812.4	3.5	3619-3642	3633	3	2	0.267857143
2084	PC-3p-107648_32	AAGACGGACGGTCAAATATTGGAC	MSTRG.14882.3	3.5	12-36	27	2	2	0.267857143
2085	PC-3p-107648_32	AAGACGGACGGTCAAATATTGGAC	MSTRG.8517.1	1.5	2043-2066	2057	4	1	0.25
2086	PC-3p-107648_32	AAGACGGACGGTCAAATATTGGAC	LOC_Os12g09570.2	3.5	2768-2792	2783	3	2	0.267857143
2087	PC-3p-130484_22	ACATCAACCTGTCATACACACAAC	MSTRG.3644.2	4	1493-1516	1507	2	4	0.4
2088	PC-3p-130484_22	ACATCAACCTGTCATACACACAAC	MSTRG.3644.5	4	1574-1597	1588	2	4	0.4
2089	PC-3p-130484_22	ACATCAACCTGTCATACACACAAC	MSTRG.3644.4	4	1589-1612	1603	2	4	0.4
2090	PC-3p-130484_22	ACATCAACCTGTCATACACACAAC	MSTRG.3644.3	4	1596-1619	1610	2	4	0.4
2091	PC-3p-130484_22	ACATCAACCTGTCATACACACAAC	MSTRG.3644.1	4	1646-1669	1660	2	4	0.4
2092	PC-3p-130484_22	ACATCAACCTGTCATACACACAAC	LOC_Os02g26550.3	4	3253-3276	3267	2	4	0.4
2093	PC-3p-130484_22	ACATCAACCTGTCATACACACAAC	LOC_Os02g26550.1	4	3836-3859	3850	2	4	0.4
2094	PC-3p-130484_22	ACATCAACCTGTCATACACACAAC	LOC_Os02g26550.2	4	3868-3891	3882	2	4	0.4
2095	PC-3p-130484_22	ACATCAACCTGTCATACACACAAC	MSTRG.10999.2	4	3921-3944	3935	2	4	0.4
2096	PC-3p-130484_22	ACATCAACCTGTCATACACACAAC	MSTRG.10999.1	4	4560-4583	4574	2	4	0.4
2097	PC-3p-133613_21	AAGACGGACGGTCAAAGTTGGGCG	LOC_Os10g07340.1	4	976-1000	991	4	1	0.25
2098	PC-3p-133613_21	AAGACGGACGGTCAAAGTTGGGCG	MSTRG.4275.3	4	2774-2798	2789	4	1	0.25
2099	PC-3p-133613_21	AAGACGGACGGTCAAAGTTGGGCG	LOC_Os02g23823.2	2	387-411	402	4	1	1
2100	PC-3p-133613_21	AAGACGGACGGTCAAAGTTGGGCG	LOC_Os03g12620.2	2.5	1595-1619	1610	4	1	0.25
2101	PC-3p-133613_21	AAGACGGACGGTCAAAGTTGGGCG	LOC_Os03g12620.1	2.5	1607-1631	1622	4	1	0.25
2102	PC-3p-133613_21	AAGACGGACGGTCAAAGTTGGGCG	MSTRG.16394.2	3	522-545	536	4	1	0.142857143
2103	PC-3p-133613_21	AAGACGGACGGTCAAAGTTGGGCG	MSTRG.16394.1	3	518-541	532	4	1	0.142857143
2104	PC-3p-133613_21	AAGACGGACGGTCAAAGTTGGGCG	LOC_Os06g04830.1	3	3586-3610	3601	4	1	0.25
2105	PC-3p-133613_21	AAGACGGACGGTCAAAGTTGGGCG	MSTRG.22131.5	3	4555-4579	4570	4	1	0.25
2106	PC-3p-133613_21	AAGACGGACGGTCAAAGTTGGGCG	MSTRG.22131.4	3	4715-4739	4730	4	1	0.25
2107	PC-3p-133613_21	AAGACGGACGGTCAAAGTTGGGCG	MSTRG.6812.3	4	3471-3495	3486	3	2	0.267857143
2108	PC-3p-133613_21	AAGACGGACGGTCAAAGTTGGGCG	MSTRG.6812.5	4	3528-3552	3543	3	2	0.267857143
2109	PC-3p-133613_21	AAGACGGACGGTCAAAGTTGGGCG	MSTRG.6812.4	4	3618-3642	3633	3	2	0.267857143
2110	PC-3p-133613_21	AAGACGGACGGTCAAAGTTGGGCG	LOC_Os03g50620.1	3	1115-1138	1129	2	5	2.5
2111	PC-3p-133613_21	AAGACGGACGGTCAAAGTTGGGCG	MSTRG.15681.3	3	2150-2173	2164	2	5	2.5
2112	PC-3p-133613_21	AAGACGGACGGTCAAAGTTGGGCG	MSTRG.8517.1	2	2042-2066	2057	4	1	0.25
2113	PC-3p-136330_20	AGATTCGTTGTCTTAGAAGGGGTC	MSTRG.26498.1	4	1113-1136	1127	2	10	1.25
2114	PC-3p-136330_20	AGATTCGTTGTCTTAGAAGGGGTC	LOC_Os07g45350.2	4	1263-1286	1277	2	10	1.25
2115	PC-3p-136330_20	AGATTCGTTGTCTTAGAAGGGGTC	LOC_Os07g45350.4	4	1331-1354	1345	2	10	1.25
2116	PC-3p-136330_20	AGATTCGTTGTCTTAGAAGGGGTC	LOC_Os07g45350.5	4	1354-1377	1368	2	10	1.25
2117	PC-3p-136330_20	AGATTCGTTGTCTTAGAAGGGGTC	LOC_Os07g45350.1	4	1358-1381	1372	2	10	1.25
2118	PC-3p-136330_20	AGATTCGTTGTCTTAGAAGGGGTC	MSTRG.26498.8	4	1445-1468	1459	2	10	1.25
2119	PC-3p-136330_20	AGATTCGTTGTCTTAGAAGGGGTC	MSTRG.26498.2	4	1397-1420	1411	2	10	1.25
2120	PC-3p-136330_20	AGATTCGTTGTCTTAGAAGGGGTC	LOC_Os07g45350.3	4	1761-1784	1775	2	10	1.25
2121	PC-3p-136330_20	AGATTCGTTGTCTTAGAAGGGGTC	MSTRG.30326.1	0	2235-2258	2249	4	1	1

2122	PC-3p-136944_20	TCAATATGCATGTGGGAAATA	LOC_Os02g44990.1	3	1809-1829	1820	3	2	0.833333333
2123	PC-3p-136944_20	TCAATATGCATGTGGGAAATA	MSTRG.19239.4	3.5	1768-1788	1779	4	1	0.333333333
2124	PC-3p-136944_20	TCAATATGCATGTGGGAAATA	MSTRG.28410.3	3.5	1685-1705	1696	2	3	0.6
2125	PC-3p-136944_20	TCAATATGCATGTGGGAAATA	LOC_Os08g36840.1	3.5	1777-1797	1788	2	3	0.6
2126	PC-3p-136944_20	TCAATATGCATGTGGGAAATA	MSTRG.28410.5	3.5	1945-1965	1956	2	3	0.6
2127	PC-3p-136944_20	TCAATATGCATGTGGGAAATA	MSTRG.28410.4	3.5	2615-2635	2626	2	3	0.6
2128	PC-3p-136944_20	TCAATATGCATGTGGGAAATA	MSTRG.28410.1	3.5	2677-2697	2688	2	3	0.6
2129	PC-3p-136944_20	TCAATATGCATGTGGGAAATA	LOC_Os10g26720.1	2	1396-1416	1407	2	2	2
2130	PC-3p-136944_20	TCAATATGCATGTGGGAAATA	MSTRG.23412.2	3	717-737	728	2	2	0.833333333
2131	PC-3p-138895_19	CAAATTGCACAAAGTCAGGGG	LOC_Os07g04240.1	4	2125-2146	2137	2	4	4
2132	PC-3p-139016_19	AGATTCATTGTACTAGGAGGGGTC	MSTRG.26498.1	2	1113-1136	1127	2	10	1.25
2133	PC-3p-139016_19	AGATTCATTGTACTAGGAGGGGTC	LOC_Os07g45350.2	2	1263-1286	1277	2	10	1.25
2134	PC-3p-139016_19	AGATTCATTGTACTAGGAGGGGTC	LOC_Os07g45350.4	2	1331-1354	1345	2	10	1.25
2135	PC-3p-139016_19	AGATTCATTGTACTAGGAGGGGTC	LOC_Os07g45350.5	2	1354-1377	1368	2	10	1.25
2136	PC-3p-139016_19	AGATTCATTGTACTAGGAGGGGTC	LOC_Os07g45350.1	2	1358-1381	1372	2	10	1.25
2137	PC-3p-139016_19	AGATTCATTGTACTAGGAGGGGTC	MSTRG.26498.8	2	1445-1468	1459	2	10	1.25
2138	PC-3p-139016_19	AGATTCATTGTACTAGGAGGGGTC	MSTRG.26498.2	2	1397-1420	1411	2	10	1.25
2139	PC-3p-139016_19	AGATTCATTGTACTAGGAGGGGTC	LOC_Os07g45350.3	2	1761-1784	1775	2	10	1.25
2140	PC-3p-139016_19	AGATTCATTGTACTAGGAGGGGTC	LOC_Os12g06650.1	3	2122-2145	2136	2	2	2
2141	PC-3p-143939_18	TGACCTATAAGTGGATTCGCA	MSTRG.8714.3	3.5	1027-1048	1038	2	2	0.666666667
2142	PC-3p-143939_18	TGACCTATAAGTGGATTCGCA	LOC_Os12g28270.1	3.5	1360-1381	1371	2	2	0.666666667
2143	PC-3p-143939_18	TGACCTATAAGTGGATTCGCA	LOC_Os12g28270.2	3.5	1360-1381	1371	2	2	0.666666667
2144	PC-3p-157596_14	TTTTGGGATGGAGGGAGTGCT	LOC_Os01g69120.3	2.5	1557-1578	1569	4	1	0.5
2145	PC-3p-157596_14	TTTTGGGATGGAGGGAGTGCT	LOC_Os01g69120.2	2.5	1557-1578	1569	4	1	0.5
2146	PC-3p-157596_14	TTTTGGGATGGAGGGAGTGCT	MSTRG.12623.10	3	2061-2081	2072	4	1	0.125
2147	PC-3p-157596_14	TTTTGGGATGGAGGGAGTGCT	MSTRG.12623.6	3	2099-2119	2110	4	1	0.125
2148	PC-3p-157596_14	TTTTGGGATGGAGGGAGTGCT	MSTRG.12623.9	3	2123-2143	2134	4	1	0.125
2149	PC-3p-157596_14	TTTTGGGATGGAGGGAGTGCT	MSTRG.12623.8	3	2099-2119	2110	4	1	0.125
2150	PC-3p-157596_14	TTTTGGGATGGAGGGAGTGCT	MSTRG.12623.5	3	2149-2169	2160	4	1	0.125
2151	PC-3p-157596_14	TTTTGGGATGGAGGGAGTGCT	LOC_Os02g54120.2	3	2131-2151	2142	4	1	0.125
2152	PC-3p-157596_14	TTTTGGGATGGAGGGAGTGCT	LOC_Os02g54120.1	3	2131-2151	2142	4	1	0.125
2153	PC-3p-157596_14	TTTTGGGATGGAGGGAGTGCT	MSTRG.12623.11	3	2762-2782	2773	4	1	0.125
2154	PC-3p-157596_14	TTTTGGGATGGAGGGAGTGCT	LOC_Os05g15510.2	4	1653-1673	1664	4	1	1
2155	PC-3p-157596_14	TTTTGGGATGGAGGGAGTGCT	MSTRG.9744.5	3.5	1885-1905	1896	2	6	0.857142857
2156	PC-3p-157596_14	TTTTGGGATGGAGGGAGTGCT	MSTRG.9744.4	3.5	1934-1954	1945	2	6	0.857142857
2157	PC-3p-157596_14	TTTTGGGATGGAGGGAGTGCT	LOC_Os02g02980.1	3.5	1935-1955	1946	2	6	0.857142857
2158	PC-3p-157596_14	TTTTGGGATGGAGGGAGTGCT	MSTRG.9744.1	3.5	1928-1948	1939	2	6	0.857142857
2159	PC-3p-157596_14	TTTTGGGATGGAGGGAGTGCT	MSTRG.9744.6	3.5	2458-2478	2469	2	6	0.857142857
2160	PC-3p-157596_14	TTTTGGGATGGAGGGAGTGCT	MSTRG.9744.7	3.5	2587-2607	2598	2	6	0.857142857
2161	PC-3p-157596_14	TTTTGGGATGGAGGGAGTGCT	MSTRG.9744.2	3.5	2547-2567	2558	2	6	0.857142857
2162	PC-3p-157596_14	TTTTGGGATGGAGGGAGTGCT	MSTRG.23211.1	3.5	1340-1360	1351	4	1	1
2163	PC-3p-157978_14	ATTCGTTTTTTATGGGACGGAGGG	LOC_Os02g30730.1	4	2703-2726	2717	2	5	1
2164	PC-3p-157978_14	ATTCGTTTTTTATGGGACGGAGGG	LOC_Os02g30730.2	4	2779-2802	2793	2	5	1
2165	PC-3p-157978_14	ATTCGTTTTTTATGGGACGGAGGG	MSTRG.11196.3	4	2916-2939	2930	2	5	1
2166	PC-3p-157978_14	ATTCGTTTTTTATGGGACGGAGGG	MSTRG.11196.1	4	2918-2941	2932	2	5	1
2167	PC-3p-157978_14	ATTCGTTTTTTATGGGACGGAGGG	MSTRG.11196.2	4	2960-2983	2974	2	5	1
2168	PC-3p-157978_14	ATTCGTTTTTTATGGGACGGAGGG	LOC_Os04g31120.1	2.5	1887-1910	1901	4	1	0.1
2169	PC-3p-157978_14	ATTCGTTTTTTATGGGACGGAGGG	LOC_Os04g31120.6	2.5	1946-1969	1960	4	1	0.1
2170	PC-3p-157978_14	ATTCGTTTTTTATGGGACGGAGGG	MSTRG.17563.4	2.5	1783-1806	1797	4	1	0.1
2171	PC-3p-157978_14	ATTCGTTTTTTATGGGACGGAGGG	LOC_Os04g31120.2	2.5	2021-2044	2035	4	1	0.1
2172	PC-3p-157978_14	ATTCGTTTTTTATGGGACGGAGGG	MSTRG.17563.2	2.5	1863-1886	1877	4	1	0.1
2173	PC-3p-157978_14	ATTCGTTTTTTATGGGACGGAGGG	MSTRG.17563.1	2.5	1932-1955	1946	4	1	0.1
2174	PC-3p-157978_14	ATTCGTTTTTTATGGGACGGAGGG	LOC_Os04g31120.4	2.5	2379-2402	2393	4	1	0.1
2175	PC-3p-157978_14	ATTCGTTTTTTATGGGACGGAGGG	MSTRG.17563.3	2.5	2190-2213	2204	4	1	0.1
2176	PC-3p-157978_14	ATTCGTTTTTTATGGGACGGAGGG	LOC_Os04g31120.3	2.5	2456-2479	2470	4	1	0.1
2177	PC-3p-157978_14	ATTCGTTTTTTATGGGACGGAGGG	LOC_Os04g31120.5	2.5	3915-3938	3929	4	1	0.1
2178	PC-3p-162808_13	TGGTAGAATAACTTACATTAT	LOC_Os02g43560.1	4	2739-2759	2750	4	1	0.5
2179	PC-3p-165261_13	AGATTCATTGTACTGGGATGTGCC	LOC_Os12g06650.1	4	2122-2145	2136	2	2	2
2180	PC-3p-166253_13	TAACTCATGGTGACGTGGACG	MSTRG.22550.3	4	1269-1289	1280	2	3	3

2181	PC-3p-166855_13	ATAAATGTGGGAAATGGTAGAATG	MSTRG.23207.6	2	1262-1285	1276	4	1	0.25
2182	PC-3p-166855_13	ATAAATGTGGGAAATGGTAGAATG	MSTRG.23207.5	2	1579-1602	1593	4	1	0.25
2183	PC-3p-166855_13	ATAAATGTGGGAAATGGTAGAATG	MSTRG.23207.2	2	1707-1730	1721	4	1	0.25
2184	PC-3p-166855_13	ATAAATGTGGGAAATGGTAGAATG	MSTRG.23207.1	2	1718-1741	1732	4	1	0.25
2185	PC-3p-1751_4294	CTCAGTTTTCTCCAACATCTTA	LOC_Os05g49970.3	3	568-589	580	0	39	13
2186	PC-3p-1751_4294	CTCAGTTTTCTCCAACATCTTA	LOC_Os05g49970.1	3	568-589	580	0	39	13
2187	PC-3p-1751_4294	CTCAGTTTTCTCCAACATCTTA	MSTRG.21762.3	3	566-587	578	0	39	13
2188	PC-3p-1751_4294	CTCAGTTTTCTCCAACATCTTA	MSTRG.22061.1	4	65-86	77	0	285	285
2189	PC-3p-176329_11	AAGACGGACGATTAAATATTGGGC	MSTRG.6812.3	4	3472-3495	3486	3	2	0.267857143
2190	PC-3p-176329_11	AAGACGGACGATTAAATATTGGGC	MSTRG.6812.5	4	3529-3552	3543	3	2	0.267857143
2191	PC-3p-176329_11	AAGACGGACGATTAAATATTGGGC	MSTRG.6812.4	4	3619-3642	3633	3	2	0.267857143
2192	PC-3p-176909_11	CATGGTGACGTGGACGAATCT	MSTRG.22150.2	4	243-262	253	2	6	3
2193	PC-3p-176909_11	CATGGTGACGTGGACGAATCT	MSTRG.22150.3	4	308-327	318	2	6	3
2194	PC-3p-19443_427	CTTATATTTTGAGACGGGGGA	MSTRG.9744.5	3.5	1891-1911	1902	4	1	0.142857143
2195	PC-3p-19443_427	CTTATATTTTGAGACGGGGGA	MSTRG.9744.4	3.5	1940-1960	1951	4	1	0.142857143
2196	PC-3p-19443_427	CTTATATTTTGAGACGGGGGA	LOC_Os02g02980.1	3.5	1941-1961	1952	4	1	0.142857143
2197	PC-3p-19443_427	CTTATATTTTGAGACGGGGGA	MSTRG.9744.1	3.5	1934-1954	1945	4	1	0.142857143
2198	PC-3p-19443_427	CTTATATTTTGAGACGGGGGA	MSTRG.9744.6	3.5	2464-2484	2475	4	1	0.142857143
2199	PC-3p-19443_427	CTTATATTTTGAGACGGGGGA	MSTRG.9744.7	3.5	2593-2613	2604	4	1	0.142857143
2200	PC-3p-19443_427	CTTATATTTTGAGACGGGGGA	MSTRG.9744.2	3.5	2553-2573	2564	4	1	0.142857143
2201	PC-3p-203816_7	ATTCAGATTCGTTGTACTAGGAGG	MSTRG.26498.1	2	1117-1140	1131	2	7	0.875
2202	PC-3p-203816_7	ATTCAGATTCGTTGTACTAGGAGG	LOC_Os07g45350.2	2	1267-1290	1281	2	7	0.875
2203	PC-3p-203816_7	ATTCAGATTCGTTGTACTAGGAGG	LOC_Os07g45350.4	2	1335-1358	1349	2	7	0.875
2204	PC-3p-203816_7	ATTCAGATTCGTTGTACTAGGAGG	LOC_Os07g45350.5	2	1358-1381	1372	2	7	0.875
2205	PC-3p-203816_7	ATTCAGATTCGTTGTACTAGGAGG	LOC_Os07g45350.1	2	1362-1385	1376	2	7	0.875
2206	PC-3p-203816_7	ATTCAGATTCGTTGTACTAGGAGG	MSTRG.26498.8	2	1449-1472	1463	2	7	0.875
2207	PC-3p-203816_7	ATTCAGATTCGTTGTACTAGGAGG	MSTRG.26498.2	2	1401-1424	1415	2	7	0.875
2208	PC-3p-203816_7	ATTCAGATTCGTTGTACTAGGAGG	LOC_Os07g45350.3	2	1765-1788	1779	2	7	0.875
2209	PC-3p-223607_6	AGATTCATTGTGCTAGGATATGTC	LOC_Os12g06650.1	3	2122-2145	2136	2	2	2
2210	PC-3p-238099_6	AGATTCGTTGTACTATGAGGGGAT	MSTRG.26498.1	2.5	1113-1136	1127	2	10	1.25
2211	PC-3p-238099_6	AGATTCGTTGTACTATGAGGGGAT	LOC_Os07g45350.2	2.5	1263-1286	1277	2	10	1.25
2212	PC-3p-238099_6	AGATTCGTTGTACTATGAGGGGAT	LOC_Os07g45350.4	2.5	1331-1354	1345	2	10	1.25
2213	PC-3p-238099_6	AGATTCGTTGTACTATGAGGGGAT	LOC_Os07g45350.5	2.5	1354-1377	1368	2	10	1.25
2214	PC-3p-238099_6	AGATTCGTTGTACTATGAGGGGAT	LOC_Os07g45350.1	2.5	1358-1381	1372	2	10	1.25
2215	PC-3p-238099_6	AGATTCGTTGTACTATGAGGGGAT	MSTRG.26498.8	2.5	1445-1468	1459	2	10	1.25
2216	PC-3p-238099_6	AGATTCGTTGTACTATGAGGGGAT	MSTRG.26498.2	2.5	1397-1420	1411	2	10	1.25
2217	PC-3p-238099_6	AGATTCGTTGTACTATGAGGGGAT	LOC_Os07g45350.3	2.5	1761-1784	1775	2	10	1.25
2218	PC-3p-2416_3415	TTCAGTTTCCTCCAACATCTTA	LOC_Os03g13750.1	4	513-534	525	4	1	1
2219	PC-3p-2416_3415	TTCAGTTTCCTCCAACATCTTA	MSTRG.22061.1	3	65-86	77	0	285	285
2220	PC-3p-2416_3415	TTCAGTTTCCTCCAACATCTTA	MSTRG.7148.1	3	1669-1690	1681	0	6011	6011
2221	PC-3p-25552_293	TAGCTCGGCTCGGCTCATTTC	MSTRG.20436.3	3	1490-1510	1501	4	1	0.333333333
2222	PC-3p-25552_293	TAGCTCGGCTCGGCTCATTTC	MSTRG.20436.2	3	1491-1511	1502	4	1	0.333333333
2223	PC-3p-25552_293	TAGCTCGGCTCGGCTCATTTC	LOC_Os05g27730.1	3	1581-1601	1592	4	1	0.333333333
2224	PC-3p-273284_5	ATTACTTATATTATGGGACGGGGG	MSTRG.2373.2	4	4484-4507	4498	2	2	0.666666667
2225	PC-3p-273284_5	ATTACTTATATTATGGGACGGGGG	MSTRG.2373.1	4	4534-4557	4548	2	2	0.666666667
2226	PC-3p-273284_5	ATTACTTATATTATGGGACGGGGG	MSTRG.18087.4	4	41-64	55	2	4	1.75
2227	PC-3p-273284_5	ATTACTTATATTATGGGACGGGGG	LOC_Os07g40450.2	3.5	88-111	102	2	2	0.75
2228	PC-3p-273284_5	ATTACTTATATTATGGGACGGGGG	LOC_Os07g40450.1	3.5	88-111	102	3	2	0.75
2229	PC-3p-293478_5	TGAGTAAATTGGTTCCCGATC	MSTRG.6470.4	4	258-277	268	4	1	0.5
2230	PC-3p-293478_5	TGAGTAAATTGGTTCCCGATC	LOC_Os11g16280.1	4	308-327	318	4	1	0.5
2231	PC-3p-30267_229	TTGCGGGACGGAGGGAGTACC	MSTRG.19222.8	4	1171-1191	1182	2	3	0.428571429
2232	PC-3p-30267_229	TTGCGGGACGGAGGGAGTACC	MSTRG.19222.2	4	1224-1244	1235	2	3	0.428571429
2233	PC-3p-30267_229	TTGCGGGACGGAGGGAGTACC	MSTRG.19222.5	4	1499-1519	1510	2	3	0.428571429
2234	PC-3p-30267_229	TTGCGGGACGGAGGGAGTACC	MSTRG.19222.4	4	1544-1564	1555	2	3	0.428571429
2235	PC-3p-30267_229	TTGCGGGACGGAGGGAGTACC	MSTRG.19222.1	4	1234-1254	1245	2	3	0.428571429
2236	PC-3p-30267_229	TTGCGGGACGGAGGGAGTACC	MSTRG.19222.9	4	2333-2353	2344	2	3	0.428571429
2237	PC-3p-30267_229	TTGCGGGACGGAGGGAGTACC	MSTRG.19222.3	4	2696-2716	2707	2	3	0.428571429
2238	PC-3p-30267_229	TTGCGGGACGGAGGGAGTACC	MSTRG.28819.1	4	776-796	787	4	1	1
2239	PC-3p-37383_169	TTTTCTTCAAACTTCAAAGTT	MSTRG.20090.1	4	1606-1626	1617	4	1	0.333333333

2240	PC-3p-37383_169	TTTTCTTCAAACTTCAAAGTT	MSTRG.25773.10	4	1857-1877	1868	4	1	0.2
2241	PC-3p-37383_169	TTTTCTTCAAACTTCAAAGTT	MSTRG.25773.6	4	3091-3111	3102	4	1	0.2
2242	PC-3p-37383_169	TTTTCTTCAAACTTCAAAGTT	MSTRG.25773.4	4	3117-3137	3128	4	1	0.2
2243	PC-3p-37383_169	TTTTCTTCAAACTTCAAAGTT	MSTRG.25773.7	4	3232-3252	3243	4	1	0.2
2244	PC-3p-37383_169	TTTTCTTCAAACTTCAAAGTT	MSTRG.25773.1	4	3259-3279	3270	4	1	0.2
2245	PC-3p-37383_169	TTTTCTTCAAACTTCAAAGTT	LOC_Os01g12870.1	4	1564-1584	1575	2	2	2
2246	PC-3p-71892_63	AGATTCGTTGTACTGGGATCTGTC	MSTRG.26498.1	3.5	1113-1136	1127	2	10	1.25
2247	PC-3p-71892_63	AGATTCGTTGTACTGGGATCTGTC	LOC_Os07g45350.2	3.5	1263-1286	1277	2	10	1.25
2248	PC-3p-71892_63	AGATTCGTTGTACTGGGATCTGTC	LOC_Os07g45350.4	3.5	1331-1354	1345	2	10	1.25
2249	PC-3p-71892_63	AGATTCGTTGTACTGGGATCTGTC	LOC_Os07g45350.5	3.5	1354-1377	1368	2	10	1.25
2250	PC-3p-71892_63	AGATTCGTTGTACTGGGATCTGTC	LOC_Os07g45350.1	3.5	1358-1381	1372	2	10	1.25
2251	PC-3p-71892_63	AGATTCGTTGTACTGGGATCTGTC	MSTRG.26498.8	3.5	1445-1468	1459	2	10	1.25
2252	PC-3p-71892_63	AGATTCGTTGTACTGGGATCTGTC	MSTRG.26498.2	3.5	1397-1420	1411	2	10	1.25
2253	PC-3p-71892_63	AGATTCGTTGTACTGGGATCTGTC	LOC_Os07g45350.3	3.5	1761-1784	1775	2	10	1.25
2254	PC-3p-71892_63	AGATTCGTTGTACTGGGATCTGTC	MSTRG.24976.5	4	1364-1387	1378	2	3	0.428571429
2255	PC-3p-71892_63	AGATTCGTTGTACTGGGATCTGTC	MSTRG.24976.6	4	1403-1426	1417	2	3	0.428571429
2256	PC-3p-71892_63	AGATTCGTTGTACTGGGATCTGTC	MSTRG.24976.1	4	1432-1455	1446	2	3	0.428571429
2257	PC-3p-71892_63	AGATTCGTTGTACTGGGATCTGTC	MSTRG.24976.7	4	2849-2872	2863	2	3	0.428571429
2258	PC-3p-71892_63	AGATTCGTTGTACTGGGATCTGTC	MSTRG.24976.2	4	2860-2883	2874	2	3	0.428571429
2259	PC-3p-71892_63	AGATTCGTTGTACTGGGATCTGTC	LOC_Os07g09460.2	4	541-564	555	2	3	0.428571429
2260	PC-3p-71892_63	AGATTCGTTGTACTGGGATCTGTC	LOC_Os07g09460.1	4	487-510	501	2	3	0.428571429
2261	PC-3p-7526_1294	TTGGGGAACGCGCCGATCGTC	MSTRG.4642.1	0	1857-1877	1868	4	1	1
2262	PC-3p-87370_46	TTAACGGTCAACACACGGTCA	MSTRG.16462.6	4	699-720	711	2	2	0.285714286
2263	PC-3p-87370_46	TTAACGGTCAACACACGGTCA	MSTRG.16462.7	4	701-722	713	2	2	0.285714286
2264	PC-3p-87370_46	TTAACGGTCAACACACGGTCA	LOC_Os03g61990.1	4	673-694	685	2	2	0.285714286
2265	PC-3p-87370_46	TTAACGGTCAACACACGGTCA	LOC_Os03g61990.5	4	653-674	665	2	2	0.285714286
2266	PC-3p-87370_46	TTAACGGTCAACACACGGTCA	LOC_Os03g61990.2	4	673-694	685	2	2	0.285714286
2267	PC-3p-87370_46	TTAACGGTCAACACACGGTCA	LOC_Os03g61990.4	4	673-694	685	2	2	0.285714286
2268	PC-3p-87370_46	TTAACGGTCAACACACGGTCA	LOC_Os03g61990.3	4	1340-1361	1352	2	2	0.285714286
2269	PC-3p-87370_46	TTAACGGTCAACACACGGTCA	LOC_Os03g47800.1	3.5	897-918	909	2	6	2
2270	PC-3p-87370_46	TTAACGGTCAACACACGGTCA	MSTRG.15500.2	3.5	2697-2718	2709	2	6	2
2271	PC-3p-87370_46	TTAACGGTCAACACACGGTCA	MSTRG.15500.1	3.5	3227-3248	3239	2	6	2
2272	PC-3p-87391_46	CATGTCGCTAATGTTGCAGCA	MSTRG.29169.3	4	197-217	208	4	1	0.142857143
2273	PC-3p-87391_46	CATGTCGCTAATGTTGCAGCA	MSTRG.29169.4	4	805-825	816	4	1	0.142857143
2274	PC-3p-87391_46	CATGTCGCTAATGTTGCAGCA	MSTRG.29169.2	4	835-855	846	4	1	0.142857143
2275	PC-3p-88439_45	ATTCAAAGTTTGGATTTTGGT	LOC_Os05g34770.1	4	195-214	206	2	4	4
2276	PC-3p-88439_45	ATTCAAAGTTTGGATTTTGGT	LOC_Os07g10660.1	4	916-936	927	2	2	2
2277	PC-3p-89060_44	TTGTACTAGGTTGTATCACA	MSTRG.12140.1	4	1266-1285	1276	3	4	1.333333333
2278	PC-3p-89060_44	TTGTACTAGGTTGTATCACA	MSTRG.28982.1	4	101-120	111	2	4	1.333333333
2279	PC-3p-89060_44	TTGTACTAGGTTGTATCACA	LOC_Os09g01000.1	4	2646-2665	2656	3	4	1.333333333
2280	PC-3p-89662_44	CTCGTCCTAGGTGAAACAGCC	MSTRG.25708.8	3.5	2746-2766	2757	2	3	0.333333333
2281	PC-3p-89662_44	CTCGTCCTAGGTGAAACAGCC	LOC_Os07g31450.1	3.5	6357-6377	6368	2	3	0.333333333
2282	PC-3p-89662_44	CTCGTCCTAGGTGAAACAGCC	MSTRG.25708.7	3.5	6646-6666	6657	2	3	0.333333333
2283	PC-3p-89662_44	CTCGTCCTAGGTGAAACAGCC	MSTRG.25708.4	3.5	6653-6673	6664	2	3	0.333333333
2284	PC-3p-89662_44	CTCGTCCTAGGTGAAACAGCC	MSTRG.25708.6	3.5	6661-6681	6672	2	3	0.333333333
2285	PC-3p-89662_44	CTCGTCCTAGGTGAAACAGCC	MSTRG.25708.5	3.5	6752-6772	6763	2	3	0.333333333
2286	PC-3p-89662_44	CTCGTCCTAGGTGAAACAGCC	MSTRG.25708.1	3.5	6758-6778	6769	2	3	0.333333333
2287	PC-3p-89662_44	CTCGTCCTAGGTGAAACAGCC	MSTRG.25708.3	3.5	6759-6779	6770	2	3	0.333333333
2288	PC-3p-89662_44	CTCGTCCTAGGTGAAACAGCC	MSTRG.25708.2	3.5	6767-6787	6778	2	3	0.333333333
2289	PC-3p-98913_37	AGATTCATTGTATTAGGAGGGGTC	MSTRG.26498.1	3	1113-1136	1127	2	10	1.25
2290	PC-3p-98913_37	AGATTCATTGTATTAGGAGGGGTC	LOC_Os07g45350.2	3	1263-1286	1277	2	10	1.25
2291	PC-3p-98913_37	AGATTCATTGTATTAGGAGGGGTC	LOC_Os07g45350.4	3	1331-1354	1345	2	10	1.25
2292	PC-3p-98913_37	AGATTCATTGTATTAGGAGGGGTC	LOC_Os07g45350.5	3	1354-1377	1368	2	10	1.25
2293	PC-3p-98913_37	AGATTCATTGTATTAGGAGGGGTC	LOC_Os07g45350.1	3	1358-1381	1372	2	10	1.25
2294	PC-3p-98913_37	AGATTCATTGTATTAGGAGGGGTC	MSTRG.26498.8	3	1445-1468	1459	2	10	1.25
2295	PC-3p-98913_37	AGATTCATTGTATTAGGAGGGGTC	MSTRG.26498.2	3	1397-1420	1411	2	10	1.25
2296	PC-3p-98913_37	AGATTCATTGTATTAGGAGGGGTC	LOC_Os07g45350.3	3	1761-1784	1775	2	10	1.25
2297	PC-3p-98913_37	AGATTCATTGTATTAGGAGGGGTC	LOC_Os12g06650.1	4	2122-2145	2136	2	2	2
2298	PC-5p-102706_34	TTCATCTAGGACGAGGATGTG	LOC_Os11g34810.1	3	1138-1157	1148	4	1	0.5

2299	PC-5p-102706_34	TTCATCTAGGACGAGGATGTG	LOC_Os11g34810.2	3	1138-1157	1148	4	1	0.5
2300	PC-5p-114783_28	CTAAAAATAGTTTTTAGTCCC	MSTRG.10459.2	4	876-895	887	4	1	0.5
2301	PC-5p-114783_28	CTAAAAATAGTTTTTAGTCCC	MSTRG.10459.1	4	876-895	887	4	1	0.5
2302	PC-5p-114783_28	CTAAAAATAGTTTTTAGTCCC	MSTRG.29090.2	4	677-696	688	4	1	0.5
2303	PC-5p-114783_28	CTAAAAATAGTTTTTAGTCCC	MSTRG.29090.1	4	951-970	962	4	1	0.5
2304	PC-5p-128789_22	ATATCCTAGTACTACGAATCTGGA	LOC_Os05g07940.4	2.5	1061-1084	1075	1	2	1
2305	PC-5p-128789_22	ATATCCTAGTACTACGAATCTGGA	LOC_Os05g07940.3	2.5	1098-1121	1112	1	2	1
2306	PC-5p-130195_22	AGATTTGAAATAAGTTTTGGCAGC	LOC_Os04g55060.1	3.5	1350-1372	1363	2	2	1
2307	PC-5p-130195_22	AGATTTGAAATAAGTTTTGGCAGC	MSTRG.19080.2	3.5	1926-1948	1939	2	2	1
2308	PC-5p-138986_19	TAGTCCCGGTTGGTGTCTTTA	MSTRG.10459.2	3	942-962	953	4	1	0.5
2309	PC-5p-138986_19	TAGTCCCGGTTGGTGTCTTTA	MSTRG.10459.1	3	942-962	953	4	1	0.5
2310	PC-5p-149326_16	TCTTTCTCTCTCTGCTCGTGG	LOC_Os01g69250.1	4	39-58	49	2	3	1
2311	PC-5p-149326_16	TCTTTCTCTCTCTGCTCGTGG	LOC_Os01g69250.4	4	39-58	49	2	3	1
2312	PC-5p-149326_16	TCTTTCTCTCTCTGCTCGTGG	LOC_Os01g69250.2	4	39-58	49	2	3	1
2313	PC-5p-149326_16	TCTTTCTCTCTCTGCTCGTGG	LOC_Os02g31290.2	3.5	3635-3655	3646	2	5	1.666666667
2314	PC-5p-149326_16	TCTTTCTCTCTCTGCTCGTGG	MSTRG.11223.3	3.5	3783-3803	3794	2	5	1.666666667
2315	PC-5p-149326_16	TCTTTCTCTCTCTGCTCGTGG	LOC_Os02g31290.1	3.5	3952-3972	3963	2	5	1.666666667
2316	PC-5p-149326_16	TCTTTCTCTCTCTGCTCGTGG	LOC_Os02g32570.2	4	1580-1600	1591	4	1	0.2
2317	PC-5p-149326_16	TCTTTCTCTCTCTGCTCGTGG	LOC_Os02g32570.1	4	2296-2316	2307	4	1	0.2
2318	PC-5p-149326_16	TCTTTCTCTCTCTGCTCGTGG	MSTRG.11266.3	4	2846-2866	2857	4	1	0.2
2319	PC-5p-149326_16	TCTTTCTCTCTCTGCTCGTGG	MSTRG.11266.2	4	2885-2905	2896	4	1	0.2
2320	PC-5p-149326_16	TCTTTCTCTCTCTGCTCGTGG	MSTRG.11266.1	4	3051-3071	3062	4	1	0.2
2321	PC-5p-149326_16	TCTTTCTCTCTCTGCTCGTGG	LOC_Os03g04430.1	3.5	187-206	197	3	2	0.2
2322	PC-5p-149326_16	TCTTTCTCTCTCTGCTCGTGG	LOC_Os03g04430.2	3.5	187-206	197	3	2	0.2
2323	PC-5p-149326_16	TCTTTCTCTCTCTGCTCGTGG	MSTRG.13245.1	3.5	362-381	372	3	2	0.2
2324	PC-5p-149326_16	TCTTTCTCTCTCTGCTCGTGG	MSTRG.13245.2	3.5	323-342	333	3	2	0.2
2325	PC-5p-149326_16	TCTTTCTCTCTCTGCTCGTGG	LOC_Os03g23020.1	3	44-63	54	2	21	21
2326	PC-5p-149326_16	TCTTTCTCTCTCTGCTCGTGG	LOC_Os03g60950.2	3.5	54-73	64	2	5	2.5
2327	PC-5p-149326_16	TCTTTCTCTCTCTGCTCGTGG	LOC_Os03g60950.1	3.5	37-56	47	2	5	2.5
2328	PC-5p-149326_16	TCTTTCTCTCTCTGCTCGTGG	LOC_Os04g56939.1	3.5	35-56	47	2	5	5
2329	PC-5p-149326_16	TCTTTCTCTCTCTGCTCGTGG	LOC_Os04g37690.1	3.5	1358-1377	1368	3	3	3
2330	PC-5p-149326_16	TCTTTCTCTCTCTGCTCGTGG	LOC_Os04g32950.4	4	6-26	17	4	1	0.333333333
2331	PC-5p-149326_16	TCTTTCTCTCTCTGCTCGTGG	LOC_Os04g32950.1	4	6-26	17	4	1	0.333333333
2332	PC-5p-149326_16	TCTTTCTCTCTCTGCTCGTGG	LOC_Os04g32950.3	4	6-26	17	4	1	0.333333333
2333	PC-5p-149326_16	TCTTTCTCTCTCTGCTCGTGG	LOC_Os07g42260.2	4	75-96	87	2	2	0.5
2334	PC-5p-149326_16	TCTTTCTCTCTCTGCTCGTGG	LOC_Os07g42260.1	4	75-96	87	2	2	0.5
2335	PC-5p-149326_16	TCTTTCTCTCTCTGCTCGTGG	MSTRG.26313.1	4	82-103	94	3	2	0.5
2336	PC-5p-149326_16	TCTTTCTCTCTCTGCTCGTGG	MSTRG.26313.2	4	63-84	75	2	2	0.5
2337	PC-5p-149326_16	TCTTTCTCTCTCTGCTCGTGG	LOC_Os07g39320.1	4	101-120	111	3	2	0.2
2338	PC-5p-149326_16	TCTTTCTCTCTCTGCTCGTGG	MSTRG.26149.1	4	136-155	146	3	2	0.2
2339	PC-5p-149326_16	TCTTTCTCTCTCTGCTCGTGG	MSTRG.28922.9	3	20-39	30	4	1	0.2
2340	PC-5p-149326_16	TCTTTCTCTCTCTGCTCGTGG	LOC_Os08g44470.1	3	53-72	63	4	1	0.2
2341	PC-5p-149326_16	TCTTTCTCTCTCTGCTCGTGG	MSTRG.28922.4	3	20-39	30	4	1	0.2
2342	PC-5p-149326_16	TCTTTCTCTCTCTGCTCGTGG	MSTRG.28922.1	3	20-39	30	4	1	0.2
2343	PC-5p-149326_16	TCTTTCTCTCTCTGCTCGTGG	MSTRG.29764.1	4	123-142	133	2	2	1
2344	PC-5p-149326_16	TCTTTCTCTCTCTGCTCGTGG	LOC_Os09g29460.1	4	276-295	286	2	2	1
2345	PC-5p-149326_16	TCTTTCTCTCTCTGCTCGTGG	MSTRG.30002.1	4	315-334	325	2	2	1
2346	PC-5p-149326_16	TCTTTCTCTCTCTGCTCGTGG	MSTRG.5673.3	3.5	53-72	63	4	1	0.25
2347	PC-5p-149326_16	TCTTTCTCTCTCTGCTCGTGG	LOC_Os11g01170.1	3.5	151-170	161	4	1	0.25
2348	PC-5p-149326_16	TCTTTCTCTCTCTGCTCGTGG	MSTRG.5673.2	3.5	101-120	111	4	1	0.25
2349	PC-5p-149326_16	TCTTTCTCTCTCTGCTCGTGG	LOC_Os11g38990.1	3.5	31-50	41	4	1	0.2
2350	PC-5p-149326_16	TCTTTCTCTCTCTGCTCGTGG	LOC_Os11g38990.2	3.5	31-50	41	4	1	0.2
2351	PC-5p-149326_16	TCTTTCTCTCTCTGCTCGTGG	MSTRG.7165.5	3.5	14-33	24	4	1	0.2
2352	PC-5p-149326_16	TCTTTCTCTCTCTGCTCGTGG	MSTRG.7165.1	3.5	34-53	44	4	1	0.2
2353	PC-5p-149326_16	TCTTTCTCTCTCTGCTCGTGG	LOC_Os11g38990.3	3.5	31-50	41	4	1	0.2
2354	PC-5p-149326_16	TCTTTCTCTCTCTGCTCGTGG	LOC_Os02g02840.1	4	318-337	328	2	2	2
2355	PC-5p-149326_16	TCTTTCTCTCTCTGCTCGTGG	MSTRG.711.1	3	3578-3597	3588	4	1	0.1
2356	PC-5p-149326_16	TCTTTCTCTCTCTGCTCGTGG	MSTRG.14855.4	4	111-131	122	4	1	0.25
2357	PC-5p-149326_16	TCTTTCTCTCTCTGCTCGTGG	MSTRG.14855.2	4	212-232	223	2	2	0.666666667

2358	PC-5p-149326_16	TCTTTCTCTCTCTGCTCGTGG	LOC_Os03g51470.1	4	44-63	54	2	12	12
2359	PC-5p-149326_16	TCTTTCTCTCTCTGCTCGTGG	LOC_Os08g06040.1	3	29-48	39	2	26	26
2360	PC-5p-154256_15	ACCTTACCAAAATTAGTCATGCC	MSTRG.20792.5	1	3940-3962	3953	4	1	0.2
2361	PC-5p-154256_15	ACCTTACCAAAATTAGTCATGCC	LOC_Os05g33390.1	1	3975-3997	3988	4	1	0.2
2362	PC-5p-154256_15	ACCTTACCAAAATTAGTCATGCC	MSTRG.20792.3	1	3905-3927	3918	4	1	0.2
2363	PC-5p-154256_15	ACCTTACCAAAATTAGTCATGCC	MSTRG.20792.2	1	3918-3940	3931	4	1	0.2
2364	PC-5p-154256_15	ACCTTACCAAAATTAGTCATGCC	MSTRG.20792.1	1	3974-3996	3987	4	1	0.2
2365	PC-5p-15574_566	TTTTTTGGCATTCTGTAACTTG	LOC_Os02g18750.1	2	176-197	188	2	25	25
2366	PC-5p-161424_14	ATGATCCTTGAGAAGGTACCA	LOC_Os06g43710.1	4	421-440	431	4	1	1
2367	PC-5p-177666_11	ATTCGTCCACGTCACCATGAG	MSTRG.7968.2	2.5	1383-1403	1394	4	1	0.5
2368	PC-5p-177666_11	ATTCGTCCACGTCACCATGAG	LOC_Os12g07020.1	2.5	1128-1148	1139	4	1	0.5
2369	PC-5p-190660_10	AAGTTGTGTGTGTATGAAAGGTTT	MSTRG.13311.1	3	2713-2736	2727	2	4	2
2370	PC-5p-190660_10	AAGTTGTGTGTGTATGAAAGGTTT	MSTRG.13311.2	3	2760-2783	2774	2	4	2
2371	PC-5p-190660_10	AAGTTGTGTGTGTATGAAAGGTTT	MSTRG.26074.3	2	673-696	687	4	1	1
2372	PC-5p-221018_6	GACGCTGAACTTAGACACCTCAGC	MSTRG.27030.3	3	1366-1389	1380	2	2	0.666666667
2373	PC-5p-221018_6	GACGCTGAACTTAGACACCTCAGC	MSTRG.27030.1	3	1929-1952	1943	2	2	0.666666667
2374	PC-5p-221018_6	GACGCTGAACTTAGACACCTCAGC	MSTRG.27030.2	3	2279-2302	2293	2	2	0.666666667
2375	PC-5p-236151_6	GAGGGATGTGACATATTCTA	MSTRG.10393.6	4	933-951	942	4	1	1
2376	PC-5p-236151_6	GAGGGATGTGACATATTCTA	MSTRG.6742.2	3	2111-2129	2120	4	1	0.5
2377	PC-5p-236151_6	GAGGGATGTGACATATTCTA	LOC_Os11g29380.1	3	2079-2097	2088	4	1	0.5
2378	PC-5p-244308_5	AGATTCATCCACGTCATCGCG	MSTRG.18817.2	4	1521-1540	1531	4	1	0.5
2379	PC-5p-244308_5	AGATTCATCCACGTCATCGCG	LOC_Os04g51710.1	4	1559-1578	1569	4	1	0.5
2380	PC-5p-28716_248	AAGATCGATCTTCGCATGCGG	LOC_Os07g03160.1	4	723-742	733	2	4	2
2381	PC-5p-28716_248	AAGATCGATCTTCGCATGCGG	MSTRG.24614.1	4	813-832	823	2	4	2
2382	PC-5p-38615_162	ATGACATAAGACGTCGCCTGG	LOC_Os02g39340.1	4	1156-1176	1167	2	2	1
2383	PC-5p-38615_162	ATGACATAAGACGTCGCCTGG	MSTRG.11679.3	4	2339-2359	2350	2	2	1
2384	PC-5p-4750_1996	AAGGTCTTGTTCGGTTAATCC	LOC_Os01g47460.1	4	2041-2061	2052	0	46	15.33333333
2385	PC-5p-4750_1996	AAGGTCTTGTTCGGTTAATCC	MSTRG.2261.1	4	2264-2284	2275	0	46	15.33333333
2386	PC-5p-4750_1996	AAGGTCTTGTTCGGTTAATCC	LOC_Os01g47460.2	4	2462-2482	2473	0	46	15.33333333
2387	PC-5p-52547_103	TTCGTCCACGTCATCACGAGT	LOC_Os11g40550.2	4	1916-1936	1927	0	3	1.5
2388	PC-5p-52547_103	TTCGTCCACGTCATCACGAGT	LOC_Os11g40550.1	4	2262-2282	2273	0	3	1.5
2389	PC-5p-52547_103	TTCGTCCACGTCATCACGAGT	MSTRG.7968.2	3	1382-1402	1393	2	3	1.5
2390	PC-5p-52547_103	TTCGTCCACGTCATCACGAGT	LOC_Os12g07020.1	3	1127-1147	1138	2	3	1.5
2391	PC-5p-58808_87	TTGGTAGGAATAGGAGGCATC	LOC_Os01g61890.4	4	1036-1055	1046	2	9	1.5
2392	PC-5p-58808_87	TTGGTAGGAATAGGAGGCATC	LOC_Os01g61890.1	4	1028-1047	1038	2	9	1.5
2393	PC-5p-58808_87	TTGGTAGGAATAGGAGGCATC	MSTRG.3242.4	4	1036-1055	1046	2	9	1.5
2394	PC-5p-58808_87	TTGGTAGGAATAGGAGGCATC	MSTRG.3242.3	4	1028-1047	1038	2	9	1.5
2395	PC-5p-58808_87	TTGGTAGGAATAGGAGGCATC	LOC_Os01g61890.2	4	1118-1137	1128	2	9	1.5
2396	PC-5p-58808_87	TTGGTAGGAATAGGAGGCATC	LOC_Os01g61890.3	4	2309-2328	2319	2	9	1.5
2397	PC-5p-63036_78	TTGAGTAGATAGCATGAGGTA	LOC_Os02g05692.2	4	1430-1450	1441	4	1	0.333333333
2398	PC-5p-63036_78	TTGAGTAGATAGCATGAGGTA	LOC_Os02g05692.1	4	1432-1452	1443	4	1	0.333333333
2399	PC-5p-63036_78	TTGAGTAGATAGCATGAGGTA	MSTRG.9920.1	4	1497-1517	1508	4	1	0.333333333
2400	PC-5p-67032_71	TTCGTCCACGTCACCATGAGT	LOC_Os01g16860.1	4	1834-1854	1845	1	2	2
2401	PC-5p-67032_71	TTCGTCCACGTCACCATGAGT	MSTRG.7968.2	3.5	1382-1402	1393	2	3	1.5
2402	PC-5p-67032_71	TTCGTCCACGTCACCATGAGT	LOC_Os12g07020.1	3.5	1127-1147	1138	2	3	1.5
2403	PC-5p-82860_50	TTGTTTCATCTAGTATGAGGA	MSTRG.27531.2	4	3039-3060	3051	2	2	1
2404	PC-5p-82860_50	TTGTTTCATCTAGTATGAGGA	LOC_Os08g14640.1	4	1618-1639	1630	2	2	1
2405	PC-5p-87356_46	CAAGTCGTTTCTGATCGTTG	LOC_Os01g64730.1	2	1070-1088	1079	2	4	4
2406	PC-5p-87356_46	CAAGTCGTTTCTGATCGTTG	LOC_Os03g54970.2	4	1029-1047	1038	4	1	0.2
2407	PC-5p-87356_46	CAAGTCGTTTCTGATCGTTG	LOC_Os03g54970.1	4	1029-1047	1038	4	1	0.2
2408	PC-5p-87356_46	CAAGTCGTTTCTGATCGTTG	MSTRG.15928.3	4	2375-2393	2384	4	1	0.2
2409	PC-5p-87356_46	CAAGTCGTTTCTGATCGTTG	MSTRG.15928.2	4	2345-2363	2354	4	1	0.2
2410	PC-5p-87356_46	CAAGTCGTTTCTGATCGTTG	MSTRG.15928.1	4	2369-2387	2378	4	1	0.2
2411	PC-5p-90396_43	TAAGTCATTCTAGCATTGCCT	MSTRG.19239.4	1.5	1846-1866	1857	2	7	7
2412	PC-5p-90396_43	TAAGTCATTCTAGCATTGCCT	LOC_Os08g40440.1	2.5	1386-1406	1397	2	2	2
2413	PC-5p-90396_43	TAAGTCATTCTAGCATTGCCT	LOC_Os10g42060.1	1.5	986-1006	997	2	9	9
2414	PC-5p-90396_43	TAAGTCATTCTAGCATTGCCT	MSTRG.24614.1	1.5	2427-2447	2438	4	1	1
2415	PC-5p-90396_43	TAAGTCATTCTAGCATTGCCT	LOC_Os08g05620.1	2.5	1780-1800	1791	4	1	0.5
2416	PC-5p-90396_43	TAAGTCATTCTAGCATTGCCT	MSTRG.27055.1	2.5	1780-1800	1791	4	1	0.5

2417	PC-5p-90636_43	GTTCGAGCATCAGCCATCTAC	LOC_Os02g47370.1	3	337-356	347	4	1	1
2418	PC-5p-90705_43	TGAGGATGATGATGTGATA	LOC_Os10g04600.1	3	553-572	563	4	1	0.1
2419	PC-5p-90705_43	TGAGGATGATGATGTGATA	LOC_Os10g04600.2	3	553-572	563	4	1	0.1
2420	PC-5p-90705_43	TGAGGATGATGATGTGATA	LOC_Os10g04600.3	3	553-572	563	4	1	0.1
2421	PC-5p-90705_43	TGAGGATGATGATGTGATA	MSTRG.4173.8	3	652-671	662	4	1	0.1
2422	PC-5p-90705_43	TGAGGATGATGATGTGATA	MSTRG.4173.3	3	720-739	730	4	1	0.1
2423	PC-5p-90705_43	TGAGGATGATGATGTGATA	MSTRG.4173.6	3	676-695	686	4	1	0.1
2424	PC-5p-90705_43	TGAGGATGATGATGTGATA	MSTRG.4173.7	3	676-695	686	4	1	0.1
2425	PC-5p-90705_43	TGAGGATGATGATGTGATA	MSTRG.4173.2	3	720-739	730	4	1	0.1
2426	PC-5p-90705_43	TGAGGATGATGATGTGATA	MSTRG.4173.5	3	676-695	686	4	1	0.1
2427	PC-5p-90705_43	TGAGGATGATGATGTGATA	MSTRG.4173.1	3	720-739	730	4	1	0.1
2428	PC-5p-90705_43	TGAGGATGATGATGTGATA	LOC_Os01g67360.1	4	1893-1911	1902	4	1	0.5
2429	PC-5p-90705_43	TGAGGATGATGATGTGATA	MSTRG.3603.1	4	1872-1890	1881	4	1	0.5
2430	PC-5p-90705_43	TGAGGATGATGATGTGATA	LOC_Os02g26860.1	3	1077-1096	1087	4	1	1
2431	PC-5p-90705_43	TGAGGATGATGATGTGATA	LOC_Os02g39390.2	3.5	1026-1045	1036	2	6	1.5
2432	PC-5p-90705_43	TGAGGATGATGATGTGATA	LOC_Os02g39390.1	3.5	1030-1049	1040	2	6	1.5
2433	PC-5p-90705_43	TGAGGATGATGATGTGATA	MSTRG.11685.2	3.5	1183-1202	1193	2	6	1.5
2434	PC-5p-90705_43	TGAGGATGATGATGTGATA	MSTRG.11685.1	3.5	1187-1206	1197	2	6	1.5
2435	PC-5p-90705_43	TGAGGATGATGATGTGATA	LOC_Os02g55140.1	4	1990-2009	2000	4	1	1
2436	PC-5p-90705_43	TGAGGATGATGATGTGATA	LOC_Os03g08440.2	3.5	285-304	295	2	2	1
2437	PC-5p-90705_43	TGAGGATGATGATGTGATA	LOC_Os03g08440.1	3.5	285-304	295	3	2	1
2438	PC-5p-90705_43	TGAGGATGATGATGTGATA	LOC_Os06g07160.1	3	819-836	827	2	2	1
2439	PC-5p-90705_43	TGAGGATGATGATGTGATA	MSTRG.22312.1	3	928-945	936	2	2	1
2440	PC-5p-90705_43	TGAGGATGATGATGTGATA	LOC_Os01g12730.1	2.5	868-885	876	4	1	0.5
2441	PC-5p-90705_43	TGAGGATGATGATGTGATA	LOC_Os01g12730.2	2.5	989-1006	997	4	1	0.5
2442	PC-5p-90705_43	TGAGGATGATGATGTGATA	LOC_Os03g46440.1	3.5	2425-2443	2434	2	3	3
2443	PC-5p-90705_43	TGAGGATGATGATGTGATA	LOC_Os06g51220.3	3.5	698-715	706	2	12	4
2444	PC-5p-90705_43	TGAGGATGATGATGTGATA	LOC_Os06g51220.2	3.5	715-732	723	2	12	4
2445	PC-5p-90705_43	TGAGGATGATGATGTGATA	LOC_Os06g51220.4	3.5	769-786	777	2	12	4
2446	PC-5p-90705_43	TGAGGATGATGATGTGATA	MSTRG.24775.3	4	253-272	263	4	1	0.2
2447	PC-5p-90705_43	TGAGGATGATGATGTGATA	MSTRG.24775.5	4	226-245	236	4	1	0.2
2448	PC-5p-90705_43	TGAGGATGATGATGTGATA	MSTRG.24775.4	4	226-245	236	4	1	0.2
2449	PC-5p-90705_43	TGAGGATGATGATGTGATA	MSTRG.24775.2	4	395-414	405	4	1	0.2
2450	PC-5p-90705_43	TGAGGATGATGATGTGATA	LOC_Os07g06500.1	4	332-351	342	4	1	0.2
2451	PC-5p-90705_43	TGAGGATGATGATGTGATA	MSTRG.26805.9	3.5	740-759	750	4	1	0.25
2452	PC-5p-90705_43	TGAGGATGATGATGTGATA	MSTRG.26805.8	3.5	743-762	753	4	1	0.25
2453	PC-5p-90705_43	TGAGGATGATGATGTGATA	MSTRG.26805.7	3.5	762-781	772	4	1	0.25
2454	PC-5p-90705_43	TGAGGATGATGATGTGATA	MSTRG.26805.3	3.5	5530-5549	5540	4	1	0.25
2455	ptc-miR160g	TGCCTGGCTCCCTGGATGCCA	LOC_Os02g41800.1	2	1484-1504	1495	0	267	89
2456	ptc-miR160g	TGCCTGGCTCCCTGGATGCCA	MSTRG.11804.1	2	1572-1592	1583	0	267	89
2457	ptc-miR160g	TGCCTGGCTCCCTGGATGCCA	LOC_Os02g41800.2	2	1484-1504	1495	0	267	89
2458	ptc-miR160g	TGCCTGGCTCCCTGGATGCCA	LOC_Os04g43910.1	2	1344-1364	1355	2	3	3
2459	ptc-miR160g	TGCCTGGCTCCCTGGATGCCA	LOC_Os04g59430.1	1.5	1334-1354	1345	2	2	2
2460	ptc-miR160g	TGCCTGGCTCCCTGGATGCCA	LOC_Os10g33940.1	2	1635-1655	1646	0	285	142.5
2461	ptc-miR160g	TGCCTGGCTCCCTGGATGCCA	MSTRG.5076.1	2	1903-1923	1914	0	285	142.5
2462	ptc-miR160g	TGCCTGGCTCCCTGGATGCCA	LOC_Os06g47150.3	2	1811-1831	1822	0	336	84
2463	ptc-miR160g	TGCCTGGCTCCCTGGATGCCA	LOC_Os06g47150.2	2	1844-1864	1855	0	336	84
2464	ptc-miR160g	TGCCTGGCTCCCTGGATGCCA	LOC_Os06g47150.1	2	2042-2062	2053	0	336	84
2465	ptc-miR160g	TGCCTGGCTCCCTGGATGCCA	LOC_Os06g47150.4	2	1844-1864	1855	0	336	84
2466	ptc-miR172g-3p	GGAATCTTGATGATGCTGCAG	MSTRG.4023.1	4	518-538	529	2	5	5
2467	ptc-miR172g-3p	GGAATCTTGATGATGCTGCAG	LOC_Os04g55560.4	2	807-827	818	0	29	5.8
2468	ptc-miR172g-3p	GGAATCTTGATGATGCTGCAG	LOC_Os04g55560.2	2	1623-1643	1634	0	29	5.8
2469	ptc-miR172g-3p	GGAATCTTGATGATGCTGCAG	LOC_Os04g55560.3	2	1634-1654	1645	0	29	5.8
2470	ptc-miR172g-3p	GGAATCTTGATGATGCTGCAG	MSTRG.19117.4	2	1909-1929	1920	0	29	5.8
2471	ptc-miR172g-3p	GGAATCTTGATGATGCTGCAG	MSTRG.19117.1	2	2085-2105	2096	0	29	5.8
2472	ptc-miR172g-3p	GGAATCTTGATGATGCTGCAG	LOC_Os05g03040.2	1.5	1552-1572	1563	0	15	5
2473	ptc-miR172g-3p	GGAATCTTGATGATGCTGCAG	LOC_Os05g03040.1	1.5	1976-1996	1987	0	15	5
2474	ptc-miR172g-3p	GGAATCTTGATGATGCTGCAG	LOC_Os05g03040.3	1.5	2089-2109	2100	0	15	5
2475	ptc-miR172g-3p	GGAATCTTGATGATGCTGCAG	LOC_Os05g07070.2	4	1274-1294	1285	2	2	0.2

2476	ptc-miR172g-3p	GGAATCTTGATGATGCTGCAG	LOC_Os05g07070.1	4	1274-1294	1285	2	2	0.2
2477	ptc-miR172g-3p	GGAATCTTGATGATGCTGCAG	MSTRG.19816.2	4	1538-1558	1549	4	1	0.1
2478	ptc-miR172g-3p	GGAATCTTGATGATGCTGCAG	LOC_Os05g07070.4	4	1601-1621	1612	2	2	0.2
2479	ptc-miR172g-3p	GGAATCTTGATGATGCTGCAG	MSTRG.19816.10	4	1672-1692	1683	4	1	0.1
2480	ptc-miR172g-3p	GGAATCTTGATGATGCTGCAG	LOC_Os05g07070.5	4	3942-3962	3953	2	2	0.2
2481	ptc-miR172g-3p	GGAATCTTGATGATGCTGCAG	MSTRG.19816.4	4	3967-3987	3978	2	2	0.2
2482	ptc-miR172g-3p	GGAATCTTGATGATGCTGCAG	MSTRG.19816.3	4	4014-4034	4025	4	1	0.1
2483	ptc-miR172g-3p	GGAATCTTGATGATGCTGCAG	MSTRG.19816.1	4	4278-4298	4289	4	1	0.1
2484	ptc-miR172g-3p	GGAATCTTGATGATGCTGCAG	LOC_Os05g07070.7	4	4362-4382	4373	2	2	0.2
2485	ptc-miR172g-3p	GGAATCTTGATGATGCTGCAG	LOC_Os05g07070.8	4	5150-5170	5161	2	2	0.2
2486	ptc-miR172g-3p	GGAATCTTGATGATGCTGCAG	LOC_Os05g07070.3	4	5590-5610	5601	2	2	0.2
2487	ptc-miR172g-3p	GGAATCTTGATGATGCTGCAG	MSTRG.28588.2	4	2016-2036	2027	4	1	0.5
2488	ptc-miR172g-3p	GGAATCTTGATGATGCTGCAG	LOC_Os08g39630.1	4	1868-1888	1879	4	1	0.5
2489	ptc-miR172g-3p	GGAATCTTGATGATGCTGCAG	LOC_Os03g60430.1	1.5	1755-1775	1766	1	3	0.75
2490	ptc-miR172g-3p	GGAATCTTGATGATGCTGCAG	LOC_Os03g60430.2	1.5	1767-1787	1778	1	3	0.75
2491	ptc-miR172g-3p	GGAATCTTGATGATGCTGCAG	MSTRG.16339.2	1.5	1933-1953	1944	1	3	0.75
2492	ptc-miR172g-3p	GGAATCTTGATGATGCTGCAG	MSTRG.16339.1	1.5	2040-2060	2051	1	3	0.75
2493	ptc-miR172g-3p	GGAATCTTGATGATGCTGCAG	MSTRG.23975.2	1	1427-1447	1438	4	1	0.5
2494	ptc-miR172g-3p	GGAATCTTGATGATGCTGCAG	LOC_Os06g43220.1	1	1480-1500	1491	4	1	0.5
2495	ptc-miR172g-3p	GGAATCTTGATGATGCTGCAG	LOC_Os07g13170.2	1.5	1463-1483	1474	2	3	0.75
2496	ptc-miR172g-3p	GGAATCTTGATGATGCTGCAG	LOC_Os07g13170.1	1.5	1404-1424	1415	2	3	0.75
2497	ptc-miR172g-3p	GGAATCTTGATGATGCTGCAG	MSTRG.25169.4	1.5	1572-1592	1583	2	3	0.75
2498	ptc-miR172g-3p	GGAATCTTGATGATGCTGCAG	MSTRG.25169.1	1.5	1565-1585	1576	2	3	0.75
2499	stu-MIR8005c-p3_1ss12AG	TAGGGTTTAGGGTTTAGGGTTT	LOC_Os01g52140.2	4	90-110	101	4	1	0.25
2500	stu-MIR8005c-p3_1ss12AG	TAGGGTTTAGGGTTTAGGGTTT	LOC_Os01g52140.1	4	90-110	101	4	1	0.25
2501	stu-MIR8005c-p3_1ss12AG	TAGGGTTTAGGGTTTAGGGTTT	MSTRG.2561.2	4	166-186	177	4	1	0.25
2502	stu-MIR8005c-p3_1ss12AG	TAGGGTTTAGGGTTTAGGGTTT	MSTRG.2561.1	4	229-249	240	4	1	0.25
2503	stu-MIR8005c-p3_1ss12AG	TAGGGTTTAGGGTTTAGGGTTT	MSTRG.12084.1	4	144-165	156	4	1	0.5
2504	stu-MIR8005c-p3_1ss12AG	TAGGGTTTAGGGTTTAGGGTTT	LOC_Os02g46150.1	4	119-140	131	4	1	0.5
2505	stu-MIR8005c-p5_1ss12AG	TAGGGTTTAGGGTTTAGGGTT	LOC_Os01g52140.2	4	91-110	101	4	1	0.25
2506	stu-MIR8005c-p5_1ss12AG	TAGGGTTTAGGGTTTAGGGTT	LOC_Os01g52140.1	4	91-110	101	4	1	0.25
2507	stu-MIR8005c-p5_1ss12AG	TAGGGTTTAGGGTTTAGGGTT	MSTRG.2561.2	4	167-186	177	4	1	0.25
2508	stu-MIR8005c-p5_1ss12AG	TAGGGTTTAGGGTTTAGGGTT	MSTRG.2561.1	4	230-249	240	4	1	0.25
2509	stu-MIR8005c-p5_1ss12AG	TAGGGTTTAGGGTTTAGGGTT	MSTRG.12084.1	3	145-165	156	4	1	0.5
2510	stu-MIR8005c-p5_1ss12AG	TAGGGTTTAGGGTTTAGGGTT	LOC_Os02g46150.1	3	120-140	131	4	1	0.5
2511	stu-MIR8005c-p5_1ss12AG	TAGGGTTTAGGGTTTAGGGTT	LOC_Os02g04660.1	3.5	45-65	56	4	1	1
2512	zma-MIR164h-p5_2ss4CG17CG	GTCGGCCGCGCCGGCGGCC	LOC_Os01g54700.1	3.5	726-744	735	2	2	2
2513	zma-MIR164h-p5_2ss4CG17CG	GTCGGCCGCGCCGGCGGCC	MSTRG.10606.1	3	83-101	92	4	1	1
2514	zma-MIR164h-p5_2ss4CG17CG	GTCGGCCGCGCCGGCGGCC	LOC_Os02g57380.2	4	299-317	308	4	1	0.5
2515	zma-MIR164h-p5_2ss4CG17CG	GTCGGCCGCGCCGGCGGCC	LOC_Os02g57380.1	4	299-317	308	4	1	0.5
2516	zma-MIR164h-p5_2ss4CG17CG	GTCGGCCGCGCCGGCGGCC	LOC_Os03g07890.1	3	1078-1096	1087	2	17	8.5
2517	zma-MIR164h-p5_2ss4CG17CG	GTCGGCCGCGCCGGCGGCC	MSTRG.13473.1	3	1214-1232	1223	2	17	8.5
2518	zma-MIR164h-p5_2ss4CG17CG	GTCGGCCGCGCCGGCGGCC	LOC_Os04g32590.1	4	496-513	504	4	1	0.333333333
2519	zma-MIR164h-p5_2ss4CG17CG	GTCGGCCGCGCCGGCGGCC	MSTRG.17641.1	4	571-588	579	4	1	0.333333333
2520	zma-MIR164h-p5_2ss4CG17CG	GTCGGCCGCGCCGGCGGCC	MSTRG.17641.2	4	369-386	377	4	1	0.333333333
2521	zma-MIR164h-p5_2ss4CG17CG	GTCGGCCGCGCCGGCGGCC	LOC_Os04g37680.1	4	666-684	675	3	2	2
2522	zma-MIR164h-p5_2ss4CG17CG	GTCGGCCGCGCCGGCGGCC	LOC_Os05g27730.1	4	1379-1397	1388	4	1	1
2523	zma-MIR164h-p5_2ss4CG17CG	GTCGGCCGCGCCGGCGGCC	LOC_Os06g04070.1	3	2679-2697	2688	2	8	8
2524	zma-MIR164h-p5_2ss4CG17CG	GTCGGCCGCGCCGGCGGCC	LOC_Os06g01700.1	4	108-126	117	3	3	3
2525	zma-MIR164h-p5_2ss4CG17CG	GTCGGCCGCGCCGGCGGCC	MSTRG.26233.1	3	264-283	274	4	1	0.333333333
2526	zma-MIR164h-p5_2ss4CG17CG	GTCGGCCGCGCCGGCGGCC	LOC_Os07g40710.2	3	264-283	274	4	1	0.333333333
2527	zma-MIR164h-p5_2ss4CG17CG	GTCGGCCGCGCCGGCGGCC	LOC_Os07g40710.1	3	264-283	274	4	1	0.333333333
2528	zma-MIR164h-p5_2ss4CG17CG	GTCGGCCGCGCCGGCGGCC	LOC_Os07g47510.1	3.5	764-782	773	2	2	2
2529	zma-MIR164h-p5_2ss4CG17CG	GTCGGCCGCGCCGGCGGCC	LOC_Os07g43230.1	4	349-366	358	3	2	0.25
2530	zma-MIR164h-p5_2ss4CG17CG	GTCGGCCGCGCCGGCGGCC	MSTRG.26376.2	4	391-408	400	3	2	0.25
2531	zma-MIR164h-p5_2ss4CG17CG	GTCGGCCGCGCCGGCGGCC	MSTRG.26376.13	4	510-527	519	3	2	0.25
2532	zma-MIR164h-p5_2ss4CG17CG	GTCGGCCGCGCCGGCGGCC	MSTRG.26376.12	4	555-572	564	3	2	0.25
2533	zma-MIR164h-p5_2ss4CG17CG	GTCGGCCGCGCCGGCGGCC	MSTRG.26376.14	4	372-389	381	3	2	0.25
2534	zma-MIR164h-p5_2ss4CG17CG	GTCGGCCGCGCCGGCGGCC	MSTRG.26376.9	4	451-468	460	3	2	0.25

2535	zma-MIR164h-p5_2ss4CG17CG	GTCGGCCGCGCCGGCGGCC	MSTRG.26376.1	4	542-559	551	3	2	0.25
2536	zma-MIR164h-p5_2ss4CG17CG	GTCGGCCGCGCCGGCGGCC	MSTRG.26376.5	4	494-511	503	3	2	0.25
2537	zma-MIR164h-p5_2ss4CG17CG	GTCGGCCGCGCCGGCGGCC	LOC_Os07g38370.1	4	177-196	186	4	1	1
2538	zma-MIR164h-p5_2ss4CG17CG	GTCGGCCGCGCCGGCGGCC	LOC_Os08g37480.2	4	471-490	481	3	2	0.333333333
2539	zma-MIR164h-p5_2ss4CG17CG	GTCGGCCGCGCCGGCGGCC	LOC_Os08g37480.1	4	471-490	481	3	2	0.333333333
2540	zma-MIR164h-p5_2ss4CG17CG	GTCGGCCGCGCCGGCGGCC	MSTRG.28438.3	4	471-490	481	3	2	0.333333333
2541	zma-MIR164h-p5_2ss4CG17CG	GTCGGCCGCGCCGGCGGCC	MSTRG.28438.1	4	488-507	498	3	2	0.333333333
2542	zma-MIR164h-p5_2ss4CG17CG	GTCGGCCGCGCCGGCGGCC	MSTRG.29573.2	4	303-321	312	4	1	0.5
2543	zma-MIR164h-p5_2ss4CG17CG	GTCGGCCGCGCCGGCGGCC	LOC_Os09g20430.1	4	140-158	149	4	1	0.5
2544	zma-MIR164h-p5_2ss4CG17CG	GTCGGCCGCGCCGGCGGCC	LOC_Os10g37690.1	3.5	296-314	305	4	1	1
2545	zma-MIR164h-p5_2ss4CG17CG	GTCGGCCGCGCCGGCGGCC	MSTRG.4910.2	3.5	401-419	410	2	2	1
2546	zma-MIR164h-p5_2ss4CG17CG	GTCGGCCGCGCCGGCGGCC	LOC_Os10g30450.1	3.5	329-347	338	2	2	1
2547	zma-MIR164h-p5_2ss4CG17CG	GTCGGCCGCGCCGGCGGCC	LOC_Os10g35870.1	4	274-292	283	2	38	12.66666667
2548	zma-MIR164h-p5_2ss4CG17CG	GTCGGCCGCGCCGGCGGCC	MSTRG.5222.2	4	292-310	301	2	38	12.66666667
2549	zma-MIR164h-p5_2ss4CG17CG	GTCGGCCGCGCCGGCGGCC	MSTRG.5222.1	4	292-310	301	2	38	12.66666667
2550	zma-MIR164h-p5_2ss4CG17CG	GTCGGCCGCGCCGGCGGCC	LOC_Os11g16480.2	3	499-516	508	2	2	1
2551	zma-MIR164h-p5_2ss4CG17CG	GTCGGCCGCGCCGGCGGCC	LOC_Os11g16480.1	3	499-516	508	2	2	1
2552	zma-MIR164h-p5_2ss4CG17CG	GTCGGCCGCGCCGGCGGCC	LOC_Os12g43110.1	4	306-325	315	2	34	34
2553	zma-MIR164h-p5_2ss4CG17CG	GTCGGCCGCGCCGGCGGCC	LOC_Os01g07970.1	3	217-234	226	4	1	1
2554	zma-MIR164h-p5_2ss4CG17CG	GTCGGCCGCGCCGGCGGCC	LOC_Os01g06440.1	4	678-696	687	4	1	0.5
2555	zma-MIR164h-p5_2ss4CG17CG	GTCGGCCGCGCCGGCGGCC	MSTRG.396.1	4	1312-1330	1321	4	1	0.5
2556	zma-MIR164h-p5_2ss4CG17CG	GTCGGCCGCGCCGGCGGCC	MSTRG.171.1	4	66-85	76	3	2	0.333333333
2557	zma-MIR164h-p5_2ss4CG17CG	GTCGGCCGCGCCGGCGGCC	LOC_Os01g04110.1	4	79-98	89	3	2	0.333333333
2558	zma-MIR164h-p5_2ss4CG17CG	GTCGGCCGCGCCGGCGGCC	LOC_Os03g31410.1	4	455-473	464	2	2	2
2559	zma-MIR164h-p5_2ss4CG17CG	GTCGGCCGCGCCGGCGGCC	LOC_Os03g52400.1	4	325-343	334	4	1	1
2560	zma-MIR164h-p5_2ss4CG17CG	GTCGGCCGCGCCGGCGGCC	LOC_Os03g59380.1	4	220-239	230	2	2	1
2561	zma-MIR164h-p5_2ss4CG17CG	GTCGGCCGCGCCGGCGGCC	MSTRG.16270.1	4	337-356	347	2	2	1
2562	zma-MIR164h-p5_2ss4CG17CG	GTCGGCCGCGCCGGCGGCC	LOC_Os06g44140.1	3	1102-1121	1111	4	1	0.5
2563	zma-MIR164h-p5_2ss4CG17CG	GTCGGCCGCGCCGGCGGCC	MSTRG.24000.1	3	1160-1179	1169	4	1	0.5
2564	zma-MIR164h-p5_2ss4CG17CG	GTCGGCCGCGCCGGCGGCC	LOC_Os06g44870.1	3.5	251-268	259	4	1	0.333333333
2565	zma-MIR164h-p5_2ss4CG17CG	GTCGGCCGCGCCGGCGGCC	MSTRG.24040.3	3.5	836-853	844	4	1	0.333333333
2566	zma-MIR164h-p5_2ss4CG17CG	GTCGGCCGCGCCGGCGGCC	MSTRG.24040.1	3.5	3247-3264	3255	4	1	0.333333333
2567	zma-MIR164h-p5_2ss4CG17CG	GTCGGCCGCGCCGGCGGCC	LOC_Os11g47670.1	3	327-344	336	2	47	47
2568	zma-MIR164h-p5_2ss4CG17CG	GTCGGCCGCGCCGGCGGCC	LOC_Os11g45400.1	4	1199-1217	1208	4	1	1
2569	zma-miR166j-3p_1ss15AG	TCGGACCAGGCTTCGATCCCT	LOC_Os03g01890.2	2.5	1090-1109	1100	0	59	6
2570	zma-miR166j-3p_1ss15AG	TCGGACCAGGCTTCGATCCCT	MSTRG.13058.1	2.5	764-783	774	0	53	5.4
2571	zma-miR166j-3p_1ss15AG	TCGGACCAGGCTTCGATCCCT	MSTRG.13058.3	2.5	998-1017	1008	0	57	5.8
2572	zma-miR166j-3p_1ss15AG	TCGGACCAGGCTTCGATCCCT	LOC_Os03g01890.1	2.5	1090-1109	1100	0	57	5.8
2573	zma-miR166j-3p_1ss15AG	TCGGACCAGGCTTCGATCCCT	MSTRG.13058.2	2.5	1186-1205	1196	0	63	6.4
2574	zma-miR166j-3p_1ss15AG	TCGGACCAGGCTTCGATCCCT	LOC_Os04g48290.1	3.5	400-420	411	2	2	2
2575	zma-miR166j-3p_1ss15AG	TCGGACCAGGCTTCGATCCCT	LOC_Os10g33960.3	2.5	925-944	935	0	65	6.5
2576	zma-miR166j-3p_1ss15AG	TCGGACCAGGCTTCGATCCCT	LOC_Os10g33960.4	2.5	925-944	935	0	68	6.8
2577	zma-miR166j-3p_1ss15AG	TCGGACCAGGCTTCGATCCCT	LOC_Os10g33960.2	2.5	697-716	707	0	65	6.5
2578	zma-miR166j-3p_1ss15AG	TCGGACCAGGCTTCGATCCCT	LOC_Os10g33960.1	2.5	925-944	935	0	62	6.2
2579	zma-miR166j-3p_1ss15AG	TCGGACCAGGCTTCGATCCCT	MSTRG.5082.2	2.5	1061-1080	1071	0	63	6.3
2580	zma-miR166j-3p_1ss15AG	TCGGACCAGGCTTCGATCCCT	MSTRG.5082.3	2.5	1128-1147	1138	0	67	6.7
2581	zma-miR166j-3p_1ss15AG	TCGGACCAGGCTTCGATCCCT	MSTRG.9425.2	2.5	499-518	509	2	6	0.6
2582	zma-miR166j-3p_1ss15AG	TCGGACCAGGCTTCGATCCCT	LOC_Os12g41860.1	2.5	878-897	888	2	7	0.7
2583	zma-miR166j-3p_1ss15AG	TCGGACCAGGCTTCGATCCCT	LOC_Os01g08520.1	3	577-598	589	2	2	1
2584	zma-miR166j-3p_1ss15AG	TCGGACCAGGCTTCGATCCCT	MSTRG.528.1	3	1092-1113	1104	2	2	1
2585	zma-miR166j-3p_1ss15AG	TCGGACCAGGCTTCGATCCCT	MSTRG.15291.8	2.5	919-938	929	2	6	0.6
2586	zma-miR166j-3p_1ss15AG	TCGGACCAGGCTTCGATCCCT	LOC_Os03g43930.2	2.5	956-975	966	2	8	0.8
2587	zma-miR166j-3p_1ss15AG	TCGGACCAGGCTTCGATCCCT	LOC_Os03g43930.1	2.5	956-975	966	2	7	0.7
2588	zma-miR166j-3p_1ss15AG	TCGGACCAGGCTTCGATCCCT	MSTRG.15291.2	2.5	887-906	897	2	8	0.8
2589	zma-miR166j-3p_1ss15AG	TCGGACCAGGCTTCGATCCCT	MSTRG.15291.9	2.5	918-937	928	2	8	0.8
2590	zma-miR166j-3p_1ss15AG	TCGGACCAGGCTTCGATCCCT	MSTRG.15291.7	2.5	329-348	339	2	7	0.7
2591	zma-miR166j-3p_1ss15AG	TCGGACCAGGCTTCGATCCCT	MSTRG.15291.5	2.5	361-380	371	2	8	0.8
2592	zma-miR166j-3p_1ss15AG	TCGGACCAGGCTTCGATCCCT	MSTRG.15291.1	2.5	541-560	551	2	7	0.7
2593	zma-miR2118c_L+1	CTTCCTAATGCCTCCCATTCCTA	MSTRG.19754.1	4	133-155	146	4	1	1

2594	zma-miR2118d_L+1	CTTCCTGATGCCTCCCATGCCTA	MSTRG.17803.1	4	316-338	329	2	2	2
2595	zma-miR2118d_L+1	CTTCCTGATGCCTCCCATGCCTA	MSTRG.9266.1	4	286-308	299	4	1	1
2596	zma-miR2118g	TTCCTGATGCCTCCTATTCCTA	MSTRG.11559.1	3.5	148-169	160	4	1	1
2597	zma-miR2118g	TTCCTGATGCCTCCTATTCCTA	MSTRG.17341.1	4	269-290	281	0	11	11
2598	zma-miR2118g	TTCCTGATGCCTCCTATTCCTA	MSTRG.17799.1	4	738-759	750	0	36	36
2599	zma-miR2118g	TTCCTGATGCCTCCTATTCCTA	MSTRG.21294.1	3.5	161-182	173	0	6	6
2600	zma-miR2118g	TTCCTGATGCCTCCTATTCCTA	MSTRG.6412.3	3.5	904-925	916	4	1	0.5
2601	zma-miR2118g	TTCCTGATGCCTCCTATTCCTA	MSTRG.6412.1	3.5	1140-1161	1152	4	1	0.5
2602	zma-miR2118g	TTCCTGATGCCTCCTATTCCTA	MSTRG.9266.1	3	286-307	298	0	84	84
2603	zma-miR2118g	TTCCTGATGCCTCCTATTCCTA	MSTRG.9262.1	3.5	182-203	194	2	2	2
2604	zma-miR2118g	TTCCTGATGCCTCCTATTCCTA	MSTRG.9259.1	3.5	784-805	796	0	77	77
2605	zma-miR2118g	TTCCTGATGCCTCCTATTCCTA	MSTRG.9256.1	3.5	114-135	126	0	72	72
2606	zma-miR2118g	TTCCTGATGCCTCCTATTCCTA	MSTRG.9254.1	3.5	401-422	413	2	3	3
2607	zma-miR2118g	TTCCTGATGCCTCCTATTCCTA	MSTRG.8777.1	4	106-127	118	4	1	1
2608	zma-miR2118g	TTCCTGATGCCTCCTATTCCTA	MSTRG.9246.1	4	115-136	127	0	46	46
2609	zma-miR2118g	TTCCTGATGCCTCCTATTCCTA	LOC_Os12g39430.1	4	697-718	709	0	8	8
2610	zma-miR2118g	TTCCTGATGCCTCCTATTCCTA	MSTRG.9308.1	4	49-70	61	0	30	30
2611	zma-miR2118g	TTCCTGATGCCTCCTATTCCTA	LOC_Os12g26270.1	4	86-107	98	1	3	3
2612	zma-miR2118g	TTCCTGATGCCTCCTATTCCTA	MSTRG.23774.1	1	10-31	22	0	29	29
2613	zma-miR2118g	TTCCTGATGCCTCCTATTCCTA	MSTRG.23338.1	3.5	150-171	162	0	12	12
2614	zma-miR2118g	TTCCTGATGCCTCCTATTCCTA	MSTRG.25143.1	3	143-164	155	0	5	5
2615	zma-miR2118g	TTCCTGATGCCTCCTATTCCTA	MSTRG.25110.1	4	712-733	724	0	3	3
2616	zma-miR2275a-3p_L-1	TTGTTTTCCTCCAATATCTCA	MSTRG.10455.1	2.5	1459-1479	1470	2	28	28
2617	zma-miR2275a-3p_L-1	TTGTTTTCCTCCAATATCTCA	MSTRG.10341.1	4	1241-1261	1252	2	2	2
2618	zma-miR2275a-3p_L-1	TTGTTTTCCTCCAATATCTCA	MSTRG.10342.1	4	1920-1940	1931	2	21	10.5
2619	zma-miR2275a-3p_L-1	TTGTTTTCCTCCAATATCTCA	MSTRG.17514.1	3.5	41-61	52	2	162	162
2620	zma-miR2275a-3p_L-1	TTGTTTTCCTCCAATATCTCA	MSTRG.9359.1	3	3138-3158	3149	2	42	42
2621	zma-miR2275a-3p_L-1	TTGTTTTCCTCCAATATCTCA	MSTRG.23853.1	4	210-230	221	2	6	6
2622	zma-miR2275a-3p_L-1	TTGTTTTCCTCCAATATCTCA	MSTRG.7464.1	3	4543-4563	4554	2	2	2
2623	zma-miR2275a-3p_L-1	TTGTTTTCCTCCAATATCTCA	MSTRG.7482.1	3.5	3-23	14	2	3	3

Table S10. The candidate miRNA–target pairs are associated with fertility in the present study.

miRNA ID	Gene ID	Comparison	miRNAs regulation	Targets regulation	Alignment Range	Degradome Category	Note
osa-miR396d	LOC_Os12g29980	H2 vs T44	down*	up	774-794	2	*OsGRF7*
osa-miR3979-3p	LOC_Os07g08530	H2 vs T44	down	up	1157-1177	4	meiosis stage-specific
osa-miR528-5p	LOC_Os03g50160	H2 vs T44	up*	down	173-192	3	PMCs-specific
osa-miR5487	LOC_Os11g38810	H2 vs T44	up*	down*	962-982	2	PMCs-specific
osa-miR5504_R-3	LOC_Os06g40550	H2 vs T44	up	down*	1970-1988	2	*OsABCG15*
PC-5p-90396_43	LOC_Os08g40440	H2 vs T44	up	down	1386-1406	2	meiosis stage-specific
osa-miR528-5p	LOC_Os07g38290	H2 vs T44	up*	down*	528-547	0	
osa-miR1846a-3p	LOC_Os05g15520	H2 vs T44	down*	up	295-315	4	
osa-MIR1846d-p3	LOC_Os05g15520	H2 vs T44	down*	up	298-318	3	
osa-miR2118d	LOC_Os04g30610	H2 vs T44	down*	up	497-518	0	
osa-miR2118p	LOC_Os04g30610	H2 vs T44	down*	up	497-518	0	
osa-miR1425-5p	LOC_Os10g35640	H2 vs T45	up*	down	1376-1396	4	*Rf1b*
osa-miR1425-5p	LOC_Os10g35240	H2 vs T45	up*	down	1296-1316	2	*Rf4|PPR782a*
osa-miR159a.1	LOC_Os01g11430	H2 vs T45	down*	up	1112-1132	2	PMCs-specific
osa-miR172a	LOC_Os05g03040	H2 vs T45	up*	down	1976-1996	0	*RSR1*
osa-miR172b	LOC_Os05g03040	H2 vs T45	up*	down	1976-1996	0	*RSR1*
osa-MIR2873a-p5_1ss6GT	LOC_Os01g13530	H2 vs T45	down	up*	738-755	3	PMCs-specific
osa-miR396c-5p	LOC_Os11g35030	H2 vs T45	up*	down	869-890	0	Neo-tetraploid rice unique genes
osa-miR396e-5p	LOC_Os11g35030	H2 vs T45	up*	down	869-889	0	Neo-tetraploid rice unique genes
osa-miR3979-3p	LOC_Os07g08530	H2 vs T45	down*	up	1157-1177	4	meiosis stage-specific
osa-miR408-3p	LOC_Os03g50140	H2 vs T45	up*	down	252-272	2	*OsUCL8*
osa-miR528-5p	LOC_Os03g50160	H2 vs T45	up*	down	173-192	3	PMCs-specific
osa-MIR5489-p3	LOC_Os10g35550	H2 vs T45	down*	up	2560-2581	4	*Du1*
osa-miR5504_R-3	LOC_Os06g40550	H2 vs T45	down	up	1970-1988	2	*OsABCG15*
osa-MIR812r-p3_1ss18CT	LOC_Os11g34910	H2 vs T45	down*	up*	2653-2673	4	Neo-tetraploid rice unique genes
PC-5p-90396_43	LOC_Os08g40440	H2 vs T45	up	down	1386-1406	2	meiosis stage-specific
bdi-miR167a_R+1	LOC_Os04g57610	H2 vs T45	up*	down*	2657-2679	2	
bdi-miR167a_R+1	LOC_Os07g33790	H2 vs T45	up*	down	3419-3439	2	
osa-miR164a	LOC_Os12g41680	H2 vs T45	up*	down*	911-931	2	
osa-miR167a-5p	LOC_Os04g57610	H2 vs T45	up*	down*	2658-2679	2	
osa-miR167a-5p	LOC_Os07g33790	H2 vs T45	up*	down	3420-3439	2	
osa-miR167d-5p	LOC_Os07g33790	H2 vs T45	up*	down	3420-3439	2	
osa-MIR169g-p3	LOC_Os03g04400	H2 vs T45	up*	down*	825-842	2	
osa-MIR169h-p3	LOC_Os03g04400	H2 vs T45	up*	down*	825-842	2	
osa-MIR169j-p3	LOC_Os03g04400	H2 vs T45	up*	down*	825-842	2	
osa-MIR169l-p3	LOC_Os03g04400	H2 vs T45	up*	down*	825-842	2	
osa-MIR169m-p3	LOC_Os03g04400	H2 vs T45	up*	down*	825-842	2	
osa-miR172a	LOC_Os03g60430	H2 vs T45	up*	down*	1755-1775	1	
osa-miR172b	LOC_Os03g60430	H2 vs T45	up*	down*	1755-1775	1	

osa-miR398b	LOC_Os07g46990	H2 vs T45	up*	down*	124-144	2
osa-miR408-3p	LOC_Os08g37670	H2 vs T45	up*	down*	657-677	0
osa-miR528-5p	LOC_Os09g20090	H2 vs T45	up*	down	229-248	2
PC-5p-28716_248	LOC_Os07g03160	H2 vs T45	up*	down*	723-742	2

Note: "*" indicated the miRNAs show significantly differential expression levels between H21 and T44/T45.

Table S11. The primers used in this study.

ID	Sequence (5'>3')	Primer (5'>3')
osa-miR5792_2ss1GA17CT	AATGACAGCGGTGGTTTGGACATC	AGCGGTGGTTTGGACATCAA
osa-miR528-5p	TGGAAGGGGCATGCAGAGGAG	GAAGGGGCATGCAGAGGAG
osa-miR408-3p	CTGCACTGCCTCTTCCCTGGC	CTGCACTGCCTCTTCCCTG
osa-miR2118p	TTCCCGATGCCTCCCATGCCTA	CCGATGCCTCCCATGCCTA
osa-MIR397b-p3	TTCACCAGCACTGCACCCAATC	ACCAGCACTGCACCCAATC
osa-miR5528	AAGACGGTTTTAGATGTTGCC	AAGACGGTTTTAGATGTTGCC
osa-miR159a.1	TTTGGATTGAAGGGAGCTCTG	TTTGGATTGAAGGGAGCTCTG
osa-MIR5495-p5	TCTTACAGCCTTATAGCACAT	TCTTACAGCCTTATAGCACAT
osa-miR528-3p	CCTGTGCTTGCCTCTTCCATT	CTGTGCTTGCCTCTTCCATT
osa-MIR397a-p5	TTGAGTGCAGCGTTGATGAAC	TGAGTGCAGCGTTGATGAAC
PC-5p-18426_458	TTTGACCGTGTGTTGACCGTTAAC	TGACCGTGTGTTGACCGTTA
osa-miR159f_R-1	CTTGGATTGAAGGGAGCTCT	CTTGGATTGAAGGGAGCTCT
PC-3p-25657_292	TTCTCAAGGACCGTAGAATTA	TTCTCAAGGACCGTAGAATTA
osa-miR398b	TGTGTTCTCAGGTCGCCCCTG	TGTGTTCTCAGGTCGCCC
osa-miR408-5p	CAGGGATGAGGCAGAGCATGG	CAGGGATGAGGCAGAGCAT
osa-MIR5793-p5	CATCGCGGGACGGAAATCTTC	GCGGGACGGAAATCTTCAA
gma-miR408a-3p_L-1R+3	TGCACTGCCTCTTCCCTGGCTTT	CTGCCTCTTCCCTGGCTTT
osa-miR1432-5p_R+1	ATCAGGAGAGATGACACCGACA	AGGAGAGATGACACCGACA
osa-miR5492	AGAAGGAGAATAGATATGGTT	AGAAGGAGAATAGATATGGTTA
osa-miR1425-3p	CAGCAAGAACTGGATCTTAAT	CAGCAAGAACTGGATCTTAAT
osa-miR1874-3p	TATGGATGGAGGTGTAACCCGATG	GATGGAGGTGTAACCCGATG
osa-MIR5806-p5	CTGGAAGTTAGCAAGTTAGAA	CTGGAAGTTAGCAAGTTAGAA
osa-MIR5793-p3	CTTTGCGTGTACCTTCTCTTG	CTTTGCGTGTACCTTCTCTTG
PC-3p-7526_1294	TTGGGGAACGCGCCGATCGTC	TTGGGGAACGCGCCGATC
PC-5p-55332_95	AGAGTTGTAGGAAACTGAACT	AGAGTTGTAGGAAACTGAACT
LOC_Os01g59660	AGCAGGCTGTGGGTTTTGACTA	ACTGAAGGGGTTGCTGCTGGAGACT
LOC_Os01g47530	TCCAGCCACCACCAACCTATCAGCA	TACTGACTTTATTGTAGTTACCTCC
LOC_Os04g30610	TTTGGTCGTGAAAAAGAACTGGAGG	ATTCCGCACCACTCCATCC
LOC_Os07g46990	TGGAGTCTTCCTCATCAGAAATCAG	CCTTGACACCCTCACTGCT
LOC_Os03g15340	TCTCCGAACGAACACACAGGGTAGC	AAGTTCTTGCCCTTGGACC
LOC_Os08g37670	GCACCACCCCAGGAAGCAG	TAGCAAACCACTCATCTCTCTCACA
LOC_Os07g38290	GATGGCGGTGAGCAAGTCG	ATGGAAGGGGCAGCAGAGG
LOC_Os06g06050	TTGCTTCATCTCATCCTCAAACTGT	ATCGTCTGGATTTGCTTTG
osa-MIR397b-p3-STTM	AAGCTTgattgggtgcagCTAtgctggtgaaGTTGTTGTTGTTATG GTCTAATTTAAATATGGTCTAAAGAAGAAGAATgattgg gtgcagCTAtgctggtgaaGGATCC	

osa-MIR5495-p5-STTM	AAGCTTatgtgctataaCTAggctgtaagaGTTGTTGTTGTTATGGTCTAATTTAAATATGGTCTAAAGAAGAAGAATatgtgctataaCTAggctgtaagaGGATCC
osa-miR5492-STTM	AAGCTTaaccatatctaCTAttctccttctGTTGTTGTTGTTATGGTCTAATTTAAATATGGTCTAAAGAAGAAGAATaaccatatctaCTAttctccttctGGATCC
osa-miR528-5p-STTM	GGTACCctcctctgcatCTAgccccttccaGTTGTTGTTGTTATGGTCTAATTTAAATATGGTCTAAAGAAGAAGAATctcctctgcatCTAgccccttccaAAGCTT
osa-MIR818d-p3_2ss13GT17GT-	GGTACCtactacctacgCTAtcccataataGTTGTTGTTGTTATGGTCTAATTTAAATATGGTCTAAAGAAGAAGAATtactacctacgCTAtcccataataAAGCTT
PC-3p-29913_234-STTM	GGTACCactcttaactcCTAtaaactccaaGTTGTTGTTGTTATGGTCTAATTTAAATATGGTCTAAAGAAGAAGAATactcttaactcCTAtaaactccaaAAGCTT
